# Total Synthesis and Anomeric Configuration Revision of Zwitterionic Polysaccharide A2’s Pentasaccharide Repeating Unit from *Bacteroides fragilis*

**DOI:** 10.1021/jacsau.5c01070

**Published:** 2025-10-24

**Authors:** Tianhui Hao, Liangwei Zhang, Tiehai Li

**Affiliations:** † State Key Laboratory of Chemical Biology, Shanghai Institute of Materia Medica, Chinese Academy of Sciences, Shanghai 201203, China; ‡ University of Chinese Academy of Sciences, Beijing 100049, China

**Keywords:** zwitterionic polysaccharide, oligosaccharide synthesis, stereoselective glycosylation, synthesis design, configuration revision

## Abstract

Zwitterionic polysaccharides (ZPSs) represent a distinctive class of bacterial glycans that elicit immune responses via a T-cell-dependent pathway, making them promising immunotherapeutic agents. Herein, we present the first total synthesis of a zwitterionic pentasaccharide repeating unit of the reported polysaccharide A2 (PS A2) structure from *Bacteroides fragilis*, accomplished through a stereocontrolled convergent [2 + 3] glycosylation strategy. The synthetic approach successfully addresses the formidable challenges posed by the complex architecture, which features two rare deoxyamino sugars 2-amino-4-acetamido-2,4,6-trideoxygalactose (AAT) and 3-acetamido-3,6-dideoxyglucose (ADG), and a unique 3-hydroxybutanoic acid-functionalized d-*glycero*-d-*manno*-heptose (d,d-Hep). A critical advancement involves the use of the Arndt–Eistert homologation reaction to selectively introduce the ether-linked 3-hydroxybutanoic acid onto the Hep moiety. Additionally, four oligosaccharide variants with distinct anomeric configurations of ManNAc and Hep residues were synthesized to revisit and confirm their stereochemical assignments. The stereoselective construction of challenging 1,2-*cis*-β-ManNAc and 1,2-*cis*-β-Hep linkages was achieved via Au­(I)-catalyzed glycosyl *ortho*-hexynylbenzoate glycosylation and B­(C_6_F_5_)_3_-promoted glycosyl trichloroacetimidate glycosylation, respectively. Comparative NMR analysis revealed that β-ManNAc signals matched the reported data and β-Hep configuration aligned more closely with native PS A2.

## Graphical abstract

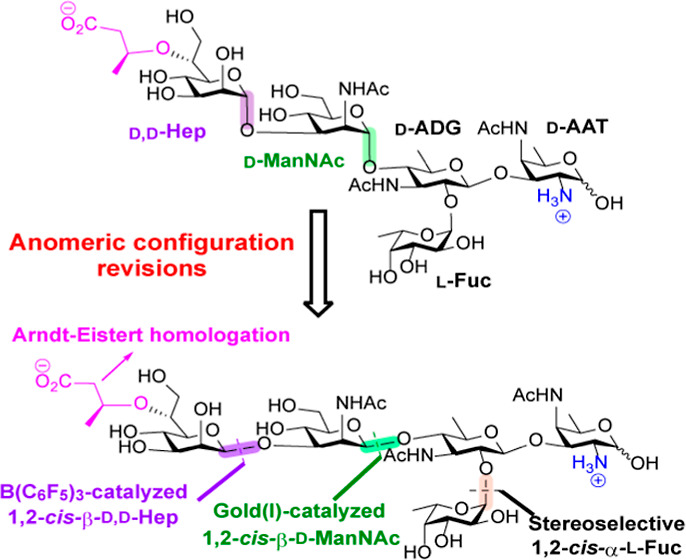

## Introduction

Zwitterionic polysaccharides (ZPSs) are a unique category of immunomodulatory bacterial glycans that defy the conventional paradigm of carbohydrate-based immunity.^1,2^ Unlike typical T cell-independent immune responses elicited by most bacterial polysaccharides, ZPSs can uniquely elicit a T-cell-dependent response without protein conjugation through major histocompatibility complex class II (MHCII) activation.^3,4^ This distinctive immunological property, stemming from their alternating cationic (amine) and anionic (carboxylate or phosphate) groups on adjacent monosaccharide residues, enables ZPSs to modulate adaptive immunity.^5,6^ The zwitterionic motifs are essential for ZPS bioactivity, as chemical neutralization of either positively charged or negatively charged groups completely abrogates their capacity to stimulate T-cell responses.^1,5,6^ Beyond their MHCII-dependent adaptive immune activation, ZPSs also engage the innate immune system via direct recognition by Toll-like receptor 2 (TLR2).^4,7^ This dual-pathway stimulation establishes a critical immunological bridge, where ZPSs coordinately drive both innate and adaptive responses.^7^ These distinctive immunological properties make ZPSs promising candidates for vaccine development and immunotherapy.^4,8^ For example, ZPSs from *Bacteroides fragilis* have been explored as carriers to conjugate with tumor-associated carbohydrate antigen in anticancer vaccines.^9–11^

The unique immunological and structural properties of ZPSs make them attractive synthetic targets.^2,12^ Representative ZPSs are shown in Figure 1, including PS A1, PS A2, and PS B from *B. fragilis*,^2,4,13^ MM-ZPS from *Morganella morganii*,^14^ and specific type 1 polysaccharide (Sp1) from *Streptococcus pneumoniae*.^15^ Despite their biological significance and biomedical potential, the synthesis and structural elucidation of ZPSs remain significantly challenging due to their complex architectures and the presence of rare deoxy amino sugars and carboxylation or phosphorylation modifications. To date, significant synthetic advances have been achieved through the total synthesis of several ZPS repeating units and extended structures from PS A1,^16–19^ PS B,^20^ MM-ZPS,^21^ and Sp1,^22–25^ enabling detailed studies of their secondary structures and structure–activity relationships. In contrast, the synthesis study of PS A2-related oligosaccharides has not yet been reported, thereby hindering a comprehensive understanding of their structural and functional characteristics and limiting their potential in biomedical applications.

**Figure 1:**
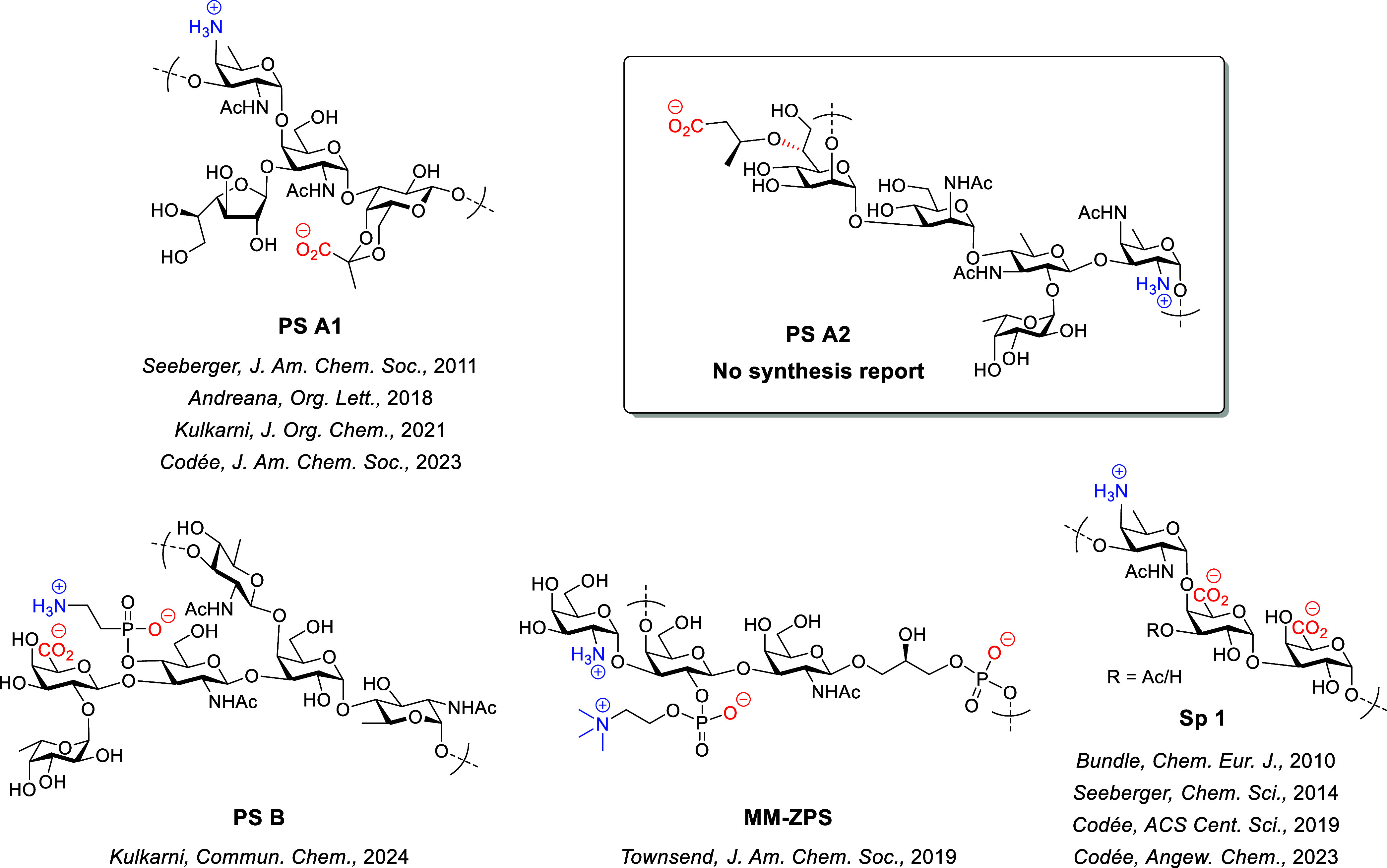
Representative ZPS structures and related synthetic reports.

The PS A2 was isolated by Kasper and co-workers from the capsular polysaccharides of *B. fragilis* 638R,^13^ a clinical pathogen causing intra-abdominal sepsis and abscesses. It is composed of 2-amino-4-acetamido-2,4,6-trideoxygalactose (AAT), 3-acetamido-3,6-dideoxyglucose (ADG), *N*-acetylmannosamine (ManNAc), d-*glycero*-d-*manno*-heptose (d,d-Hep), l-fucose (Fuc), and 3­(*S*)-hydroxybutanoic acid (3Hb) (Figure 1). The chemical structure of the PS A2 repeating unit was elucidated as →2)-[(*S*)-3Hb-(3→6)]-α-d,d-Hep*p*-(1→3)-α-d-Man*p*NAc-(1→4)-[α-l-Fuc*p*-(1→2)]-β-d-ADG*p*-(1→3)-α-d-AAT*p*-(1→.^13^ Compared with other ZPS oligosaccharide repeating units, this structure exhibits exceptional complexity. First, it features a dense arrangement of rare sugars, including the previously unreported aminodideoxyhexose ADG and the higher-carbon sugar d,d-Hep in ZPSs. Notably, the AAT residue in PS A2 carries a C-2 amino group, contrasting with the C-4-NH_2_ substitution observed in PS A1 and Sp1.^2,12^ Additionally, the 3Hb moiety is uniquely attached to the 6-OH of the Hep residue via an ether linkage, which is unprecedented in ZPSs. Collectively, these distinctive structural features pose significant synthetic challenges to the pentasaccharide repeating unit of PS A2.

To overcome the challenges, we report the first total synthesis of pentasaccharide repeating unit **1**, corresponding to the reported structure of PS A2 (Figure 2). The pentasaccharide **1** was efficiently assembled by a stereocontrolled convergent [2 + 3] glycosylation strategy from five orthogonally protected and strategically designed monosaccharide building blocks. A pivotal aspect of this synthesis is the selective introduction of the synthetically challenging ether-linked 3-hydroxybutanoic acid onto the Hep moiety via an Arndt–Eistert homologation reaction.^26^ However, the spectroscopic data obtained for synthetic repeating unit **1** exhibit some deviations when compared with those reported for natural PS A2. Specifically, the chemical shifts of the α-ManNAc and α-Hep residues stand out as notable points of discrepancy. After re-examining the reported NMR data and scrutinizing the original NOESY spectrum, we have formulated a hypothesis that the anomeric configurations of these two sugar residues may have been erroneously assigned previously. It is now more plausible that they should be designated as β-linkages. To test this hypothesis, we designed and synthesized two pentasaccharides **2–3** and two frame-shift trisaccharides **4**–**5** (Figure 2). During synthesis, key features include gold­(I)-catalyzed glycosylation of *ortho*-hexynylbenzoate (OABz) donor (Yu glycosylation)^27^ for stereoselective formation of the challenging 1,2-*cis*-β-ManNAc linkage and B­(C_6_F_5_)_3_-promoted glycosylation of trichloroacetimidate (TCAI) donor^28^ for the construction of the more challenging 1,2-*cis*-β-Hep linkage. Additionally, comprehensive NMR analysis revealed that the β-ManNAc residue displayed remarkable consistency with the previously reported NMR data, and the signals of β-Hep residue aligned more closely with the data obtained from a natural isolate.

**Figure 2:**
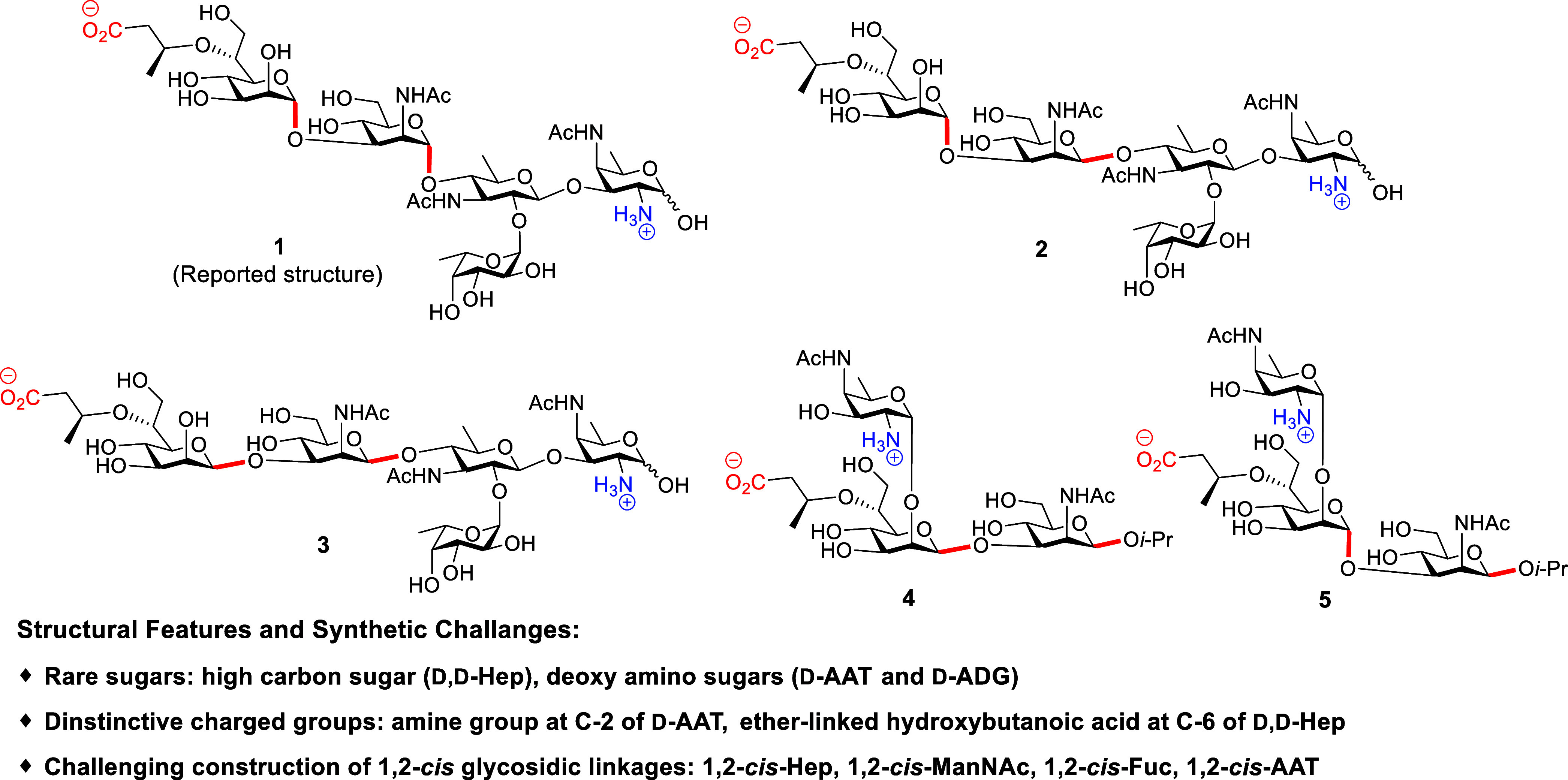
Synthetic pentasaccharide **1** as the reported structure and oligosaccharides **2–5** for structural validation studies. *i*-Pr, isopropyl.

## Results and Discussion

### Synthetic Strategy to Assemble the Reported Structure of PS A2 Pentasaccharide **1**

Given the structural complexity of pentasaccharide **1**, the synthetic challenges include the accessibility of rare monosaccharide building blocks, stereoselective construction of different glycosidic bonds, chemoselective differentiation of amino groups for late-stage acylation, and regioselective installation of the 3Hb group on the Hep residue. In the retrosynthetic analysis, as illustrated in Scheme 1, the pentasaccharide **1** was envisaged to be synthesized after the global deprotection of fully protected compound **6**, which was designed to be prepared by a stereocontrolled convergent [2 + 3] approach utilizing disaccharide donor **7** and trisaccharide acceptor **8**. A well-tailored orthogonal protecting group strategy made it possible to achieve highly stereoselective glycosylations and selective acylation of distinct amino groups. Anchimeric assistance group 2,2,2-trichloroethoxycarbonyl (Troc)^29^ for protecting amine in **7** was chosen to ensure the stereospecific glycosylation with **8** for the construction of 1,2-*trans*-α-ManNAc linkage. Meanwhile, this NHTroc group, along with two azide groups, served as precursors for subsequent conversion to acetamides (NHAc).^30^ The permanent benzyloxycarbonyl (Cbz) protecting group was chosen to mask the amino group at C-2 of the AAT residue. The disaccharide thioglycoside donor **7**, featuring a 1,2-*trans*-α-heptosidic bond, could be stereospecifically constructed by the glycosylation of *N*-phenyl trifluoroacetimidate (PTFAI) donor **9** and thioglycoside acceptor **10,** relying on the neighboring group participation of fluorenylmethoxycarbonyl (Fmoc) in C-2 of **9**. The Hep building block **11** with free 6-OH was chosen to install the crucial 3Hb group. Furthermore, functionalized butanols **12a**–**b** and commercially available 2­(*R*)-bromopropionic acid **12c** were investigated as potential precursors for 3Hb group installation. The trisaccharide acceptor **8** could be prepared by the 1,2-*cis*-α-fucosylation of thioglycoside donor **14** with disaccharide **13** through a combination of anomeric effect and ether solvent effect.^30^ The 2-OH of ADG **15** was orthogonally protected with the transient levulinoyl (Lev) group^31^ for ensuring the formation of 1,2-*trans*-glycosyl bond with AAT acceptor **16** and subsequent selective removal of the Lev group to afford the disaccharide **13**. Additionally, the target pentasaccharide **1** could be obtained by chemoselectively reducing the NHTroc and azide groups to NH_2_ for acetylation, followed by final global deprotection.

**Scheme 1:**
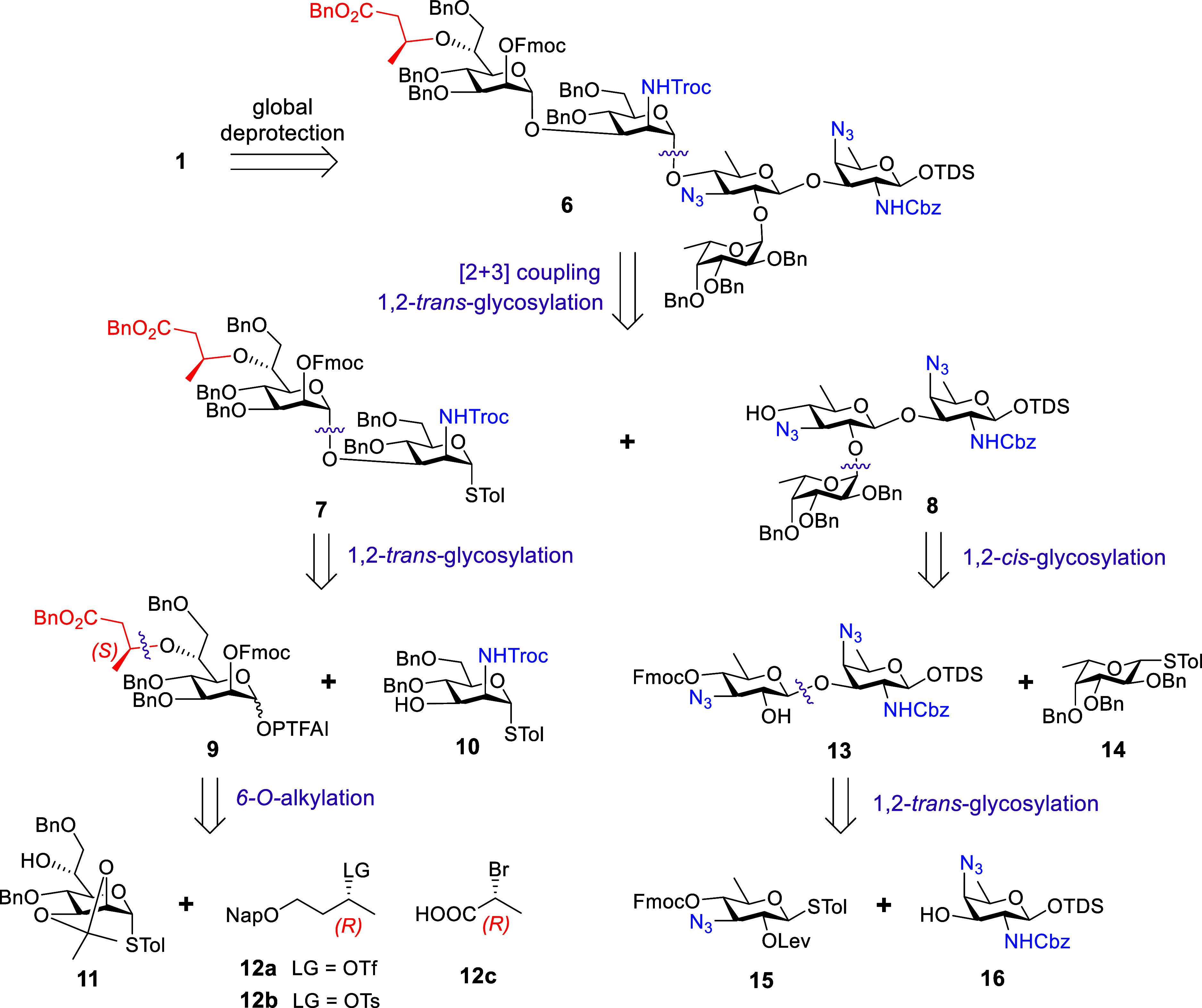
Retrosynthetic Analysis of the Pentasaccharide **1**

### Synthesis of Rare Monosaccharide Building Blocks

The synthesis of building blocks **9**, **15**, and **16** is depicted in Scheme 2. The synthesis of rare higher-carbon building block **9** is challenging due to the construction of the heptose (Hep) skeleton and the regioselective installation of the 3Hb group (Scheme 2A). Gratifyingly, a gram-scale Hep intermediate **11** was efficiently prepared by an 11-step reaction procedure starting from commercially available d-mannose according to our previous report.^32^ Subsequently, we explored potential methods for installing the 3Hb group at C-6 of **11**. Initially, we attempted direct *O*-6 alkylation of **11** with **12a/b** via Williamson ether synthesis, with the plan to selectively unmask and oxidize the 2-naphthylmethyl (Nap)-protected hydroxyl group to a carboxylic acid for subsequent benzylation.^31^ Unfortunately, all attempts failed to form the desired ether bond (Table S6), and β-elimination byproducts from **12a**–**b** were observed, which might be facilitated by the alkoxide and excess base. Inspired by our previous successful installation of a lactic acid moiety in the synthesis of muramyl dipeptide (MDP)^33^ and the Arndt–Eistert homologation reaction,^26^ we devised an indirect strategy to introduce the challenging 3Hb group. First, the lactic acid moiety was installed at the C-6-OH of **11** using 2­(*R*)-bromopropionic acid **12c** and NaH in a mixed solvent of DMF and 1,4-dioxane to afford compound **18** through an S_N_2-type reaction.^33^ The HMBC correlation between C-2 of lactic acid and H-6 of Hep confirmed the formation of a C–O ether linkage in **18**. Our synthetic case further indicated that 2-bromopropionic acid was a viable substrate for coupling with alkoxides under strong-base-promoted Williamson ether synthesis. It is also noted that the types of protecting and functional groups should be carefully considered to tolerate the basic conditions. Next, employing the standard condition of Arndt–Eistert homologation reaction,^26,34^ compound **18** was successfully converted to an α-diazoketone intermediate. The resulting intermediate was subjected to a silver trifluoroacetate (TFAOAg)-mediated Wolff rearrangement in the presence of benzyl alcohol (BnOH), furnishing benzyl ester **19** in an overall yield of 72% in three steps. Subsequently, compound **19** was transformed into **22** through a facile three-step procedure, including the cleavage of an isopropylidene group using 80% acetic acid, dibutyltin oxide (*n-*Bu_2_SnO)-mediated regioselective benzylation of the C-3-OH of **20**, and the protection of the C-2-OH of **21** with an Fmoc group. Lastly, the thioglycoside **22** was converted to a hemiacetal using *N*-iodosuccinimide (NIS) and catalytic trifluoromethanesulfonic acid (TfOH) in wet dichloromethane, and the resulting hemiacetal was treated with CF_3_C­(NPh)Cl and Cs_2_CO_3_ to yield the desired *N*-phenyl trifluoroacetimidate (PTFAI) Hep donor **9**.^35^

**Scheme 2:**
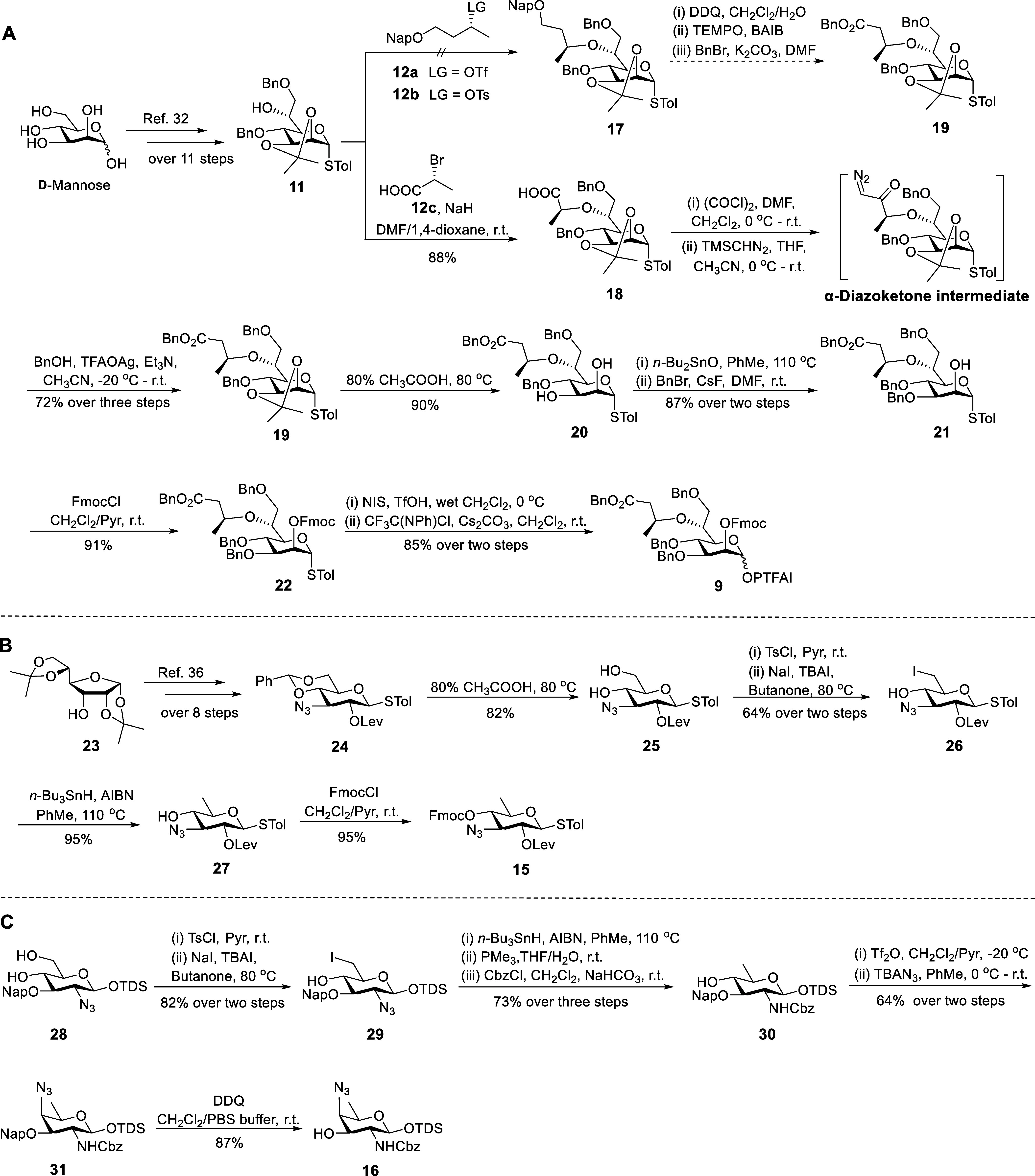
Synthesis of Hep Building Block 9 (A), ADG Building Block 15 (B), and AAT Building Block 16 (C)

Next, ADG building block **15** was synthesized from commercially available 1,2:5,6-di-*O*-isopropylidene-α-d-allofuranose **23** (Scheme 2B). Compound **24** was readily obtained through an eight-step reaction procedure starting from **23** based on a previous report.^36^ The removal of the 4,6-*O*-benzylidene protecting group of **24** with 80% acetic acid provided diol **25**. The 6-hydroxyl group of **25** was selectively tosylated by 4-toluenesulfonyl chloride (TsCl), followed by treatment with sodium iodide (NaI) and catalytic tetrabutylammonium iodide (TBAI) in refluxing butanone to afford iodide **26**.^37^ Subsequently, compound **26** was subjected to radical reduction with tributyltin hydride (*n-*Bu_3_SnH) and azobis­(isobutyronitrile) (AIBN)^38^ to give **27** in a yield of 95%. Finally, the Fmoc protecting group was introduced on C-4-OH of **27** to provide the orthogonally protected ADG building block **15**.

To synthesize AAT building block **16** (Scheme 2C), a readily available 4,6-diol **28** ^39^ was first converted to the iodide **29** using the same method described for the preparation of **26**. Compound **29** was treated with Bu_3_SnH and AIBN^38^ to remove 6-iodine, followed by reduction of the azide group with trimethyl phosphine (PMe_3_)^31,40^ to yield an amine intermediate, which was reacted with CbzCl to afford compound **30**. The 4-hydroxyl group of **30** was triflated using Tf_2_O and pyridine (Pyr), followed by the replacement with tetrabutylammonium azide (TBAN_3_)^41^ in the axial position to yield the azide derivative **31** in an overall yield of 64% for two steps. Finally, the cleavage of Nap ether with 2,3-dichloro-5,6-dicyano-1,4-benzoquinone (DDQ) in a mixed solution of dichloromethane and phosphate buffer (PBS, pH 7.4)^31,42^ provided the desired AAT building block **16** in 87% yield. Additionally, ManNTroc building block **10** was readily synthesized, as shown in Scheme S8, and Fuc building block **14** was easily obtained as previously reported.^43^

### Assembly of Pentasaccharide **1** and ^1^H NMR Data Analysis

With five building blocks in hand, we set out to assemble the fully protected pentasaccharide **6** through a stereocontrolled convergent [2 + 3] strategy (Scheme 3). The ADG thioglycosyl donor **15** and AAT acceptor **16** were condensed to provide disaccharide **32**. After investigating the promoter, solvent, and reaction temperature, disaccharide **32** was obtained in 75% yield using 2.0 equiv of NIS (based on the donor) and 0.4 equiv of TfOH in toluene at 0 °C to room temperature (Table S7). The newly formed stereospecific β-linkage (*J*_C1–H1_ = 161 Hz) is attributed to the anchimeric assistance of the Lev group. Subsequently, the Lev group of **32** was selectively removed by hydrazine acetate^31^ to give glycosyl acceptor **13** in 86% yield. The resulting acceptor **13** was coupled with Fuc thioglycosyl donor **14** in the presence of NIS and TfOH in a mixed solvent of diethyl ether (Et_2_O) and dichloromethane (CH_2_Cl_2_) at −78 °C^30,44^ to afford trisaccharide **33** in 82% yield with excellent α-selectivity (α/β > 20:1, *J*_C1–H1_ = 171 Hz). This highly stereoselective glycosylation resulted from the inherent glycosyl anomeric effect of Fuc and the solvent effect of diethyl ether.^30^ Subsequent removal of the Fmoc group of **33** with Et_3_N^45^ afforded trisaccharide acceptor **8** in 92% yield. Meanwhile, disaccharide **7** was obtained through the coupling of PTFAI donor **9** and ManNTroc acceptor **10**. Under the condition of a catalytic amount of TfOH, the newly formed α-linked disaccharide **7** (*J*_C1–H1_ = 173 Hz) was smoothly generated with a high yield (92%) and specific stereoselectivity due to the neighboring participation effect of the Fmoc group.

**Scheme 3:**
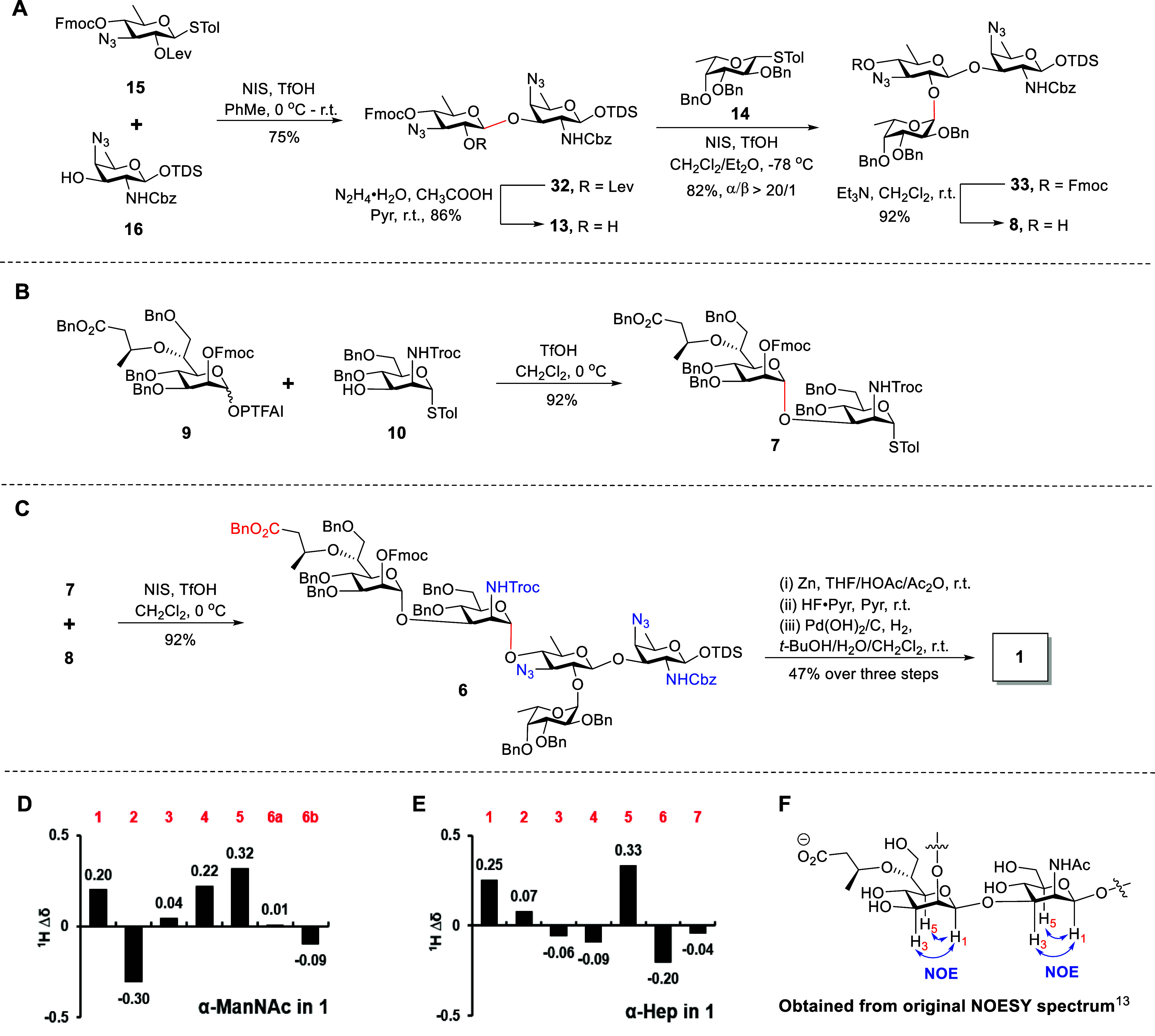
Synthesis of Pentasaccharide **1** (A–C), the Comparison of the ^1^H NMR Chemical Shifts of ManNAc and Hep Residues in 1 (Δδ ppm) with Those of the Reported Data (D,E), and the Characteristic NOE Correlations of Hep and ManNAc Residues in Native PS A2 (F)

Next, the fully protected pentasaccharide **6** was efficiently assembled by glycosylating thioglycoside donor **7** with trisaccharide acceptor **8** using the promoter combination of NIS/TfOH in 92% yield. The exclusive α-anomeric selectivity (*J*_C1–H1_ = 172 Hz) of the glycosylation reaction was attributed to the neighboring participation effect of the Troc group. Finally, the global deprotection of **6** successfully provided the target pentasaccharide **1** by converting NHTroc and azido groups into acetylamino (NHAc) with the Zn/AcOH/Ac_2_O system, followed by removal of the anomeric TDS with HF/pyridine and hydrogenolysis of Bn, Cbz, and Fmoc groups with Pd­(OH)_2_/C as the catalyst in the mixed solvent of *t-*BuOH/H_2_O/CH_2_Cl_2_ (v/v/v, 3/2/0.5). Comprehensive structural analysis of **1** was performed by 1D/2D NMR spectroscopy and high-resolution mass spectrometry (HRMS), confirming the structural integrity and stereochemistry of all glycosidic linkages (Supporting Information Pages S144–S147 and S207).

Unexpectedly, ^1^H NMR data of synthetic pentasaccharide repeating unit **1** displayed apparent deviations from those of natural PS A2 ^13^ (Table S2). The ^1^H NMR data of the ADG residue, located in the central region of the pentasaccharide backbone, were in excellent agreement with the reported data. For the AAT and its proximal Fuc residues, the delicate differences can be rationalized by the synthetic structure possessing a free anomeric hydroxyl group at the reducing end rather than a hindered glycosidic linkage. However, as depicted in Scheme 3D,E, the discrepancies in the signals from the distal Hep and ManNAc residues remained perplexing. Notably, the significant downfield shifts observed in the H-1 protons (Δδ = +0.20 ppm for ManNAc, +0.25 ppm for Hep) and H-5 protons (Δδ = +0.32 ppm for ManNAc, +0.33 ppm for Hep) prompted us to question their characterized anomeric configurations of these two glycosyl units.^13,46^ After re-examining the NOESY spectrum of natural PS A2 provided in the original paper,^13^ we were surprised to find NOE correlations between the H-1 and axially oriented H-5/H-3 protons within both Hep and ManNAc residues (Figure S2). Collectively, these findings suggest that the Hep and ManNAc glycosyl units in natural PS A2 may adopt anomeric β-configurations. To test this hypothesis, we resolved to synthesize the corresponding structures containing β-linked ManNAc or Hep residues.

### Synthesis of β-ManNAc-Containing Pentasaccharide **2**

Given the notorious challenges of constructing two consecutive 1,2-*cis*-glycosyl linkages, we initially focused on the synthesis of pentasaccharide **2** containing a β-ManNAc residue via the [(1 + 3) + 1] assembly strategy. The synthesis commenced with the stereoselective construction of the β-ManN_3_ linkage through glycosylation of trisaccharide acceptor **8** and 4,6-*O*-benzylidenated ManN_3_ donors **34a**–**d** (Table 1), respectively. First, we attempted a hydrogen-bond-mediated aglycone delivery (HAD) approach^47^ using a C-3 picoloyl (Pico)-modified thioglycosyl donor **34a** (Table 1, Entry 1). Unfortunately, no desired product was generated. Subsequently, the thioglycosyl donor **34a** was transformed to a more reactive OPTFAI donor **34b** for coupling with acceptor **8**. However, only a trace amount of product **35** was detected by TLC and mass spectrometry analysis (Table 1, Entry 2). The failure of glycosylation resulted from the reactivity mismatch between the glycosyl donor and the acceptor. Next, we employed Crich β-mannosidation protocol,^48^ which involves Ph_2_SO/Tf_2_O-mediated preactivation glycosylation, to couple Nap-protected donor **34c** with trisaccharide acceptor **8** (Table 1, Entry 3). Activation of donor **34c** with Ph_2_SO and Tf_2_O at −60 °C, followed by the addition of trisaccharide acceptor **8** and gradually warming to room temperature (r.t.), yielded **36** in a poor yield of 30% with β/α selectivity of 2.5/1 (*J*_C1–H1_ = 161 Hz for β-anomer, *J*_C1–H1_ = 171 Hz for α*-*anomer). In this reaction, as the temperature was raised to room temperature, most of the donor decomposed, while the acceptor remained largely unreacted. Alternatively, the gold­(I)-catalyzed glycosyl *ortho*-hexynylbenzoates (OABz) glycosylation method (Yu glycosylation) was also employed to construct this challenging β-linkage.^49,50^ Gratifyingly, the coupling of glycosyl donor **34d** and acceptor **8** proceeded smoothly in the presence of Ph_3_PAuOTf in PhMe at −40–0 °C, giving the tetrasaccharide **8** in a high yield of 89% with a moderate β-selectivity (β/α = 3.5/1, Table 1, Entry 4). When PhCl was used as the solvent, the β/α ratio was improved to 5/1 (Table 1, Entry 5). Additionally, the desired β-isomer **36β** can be easily purified through silica gel column chromatography.

**Table 1:**
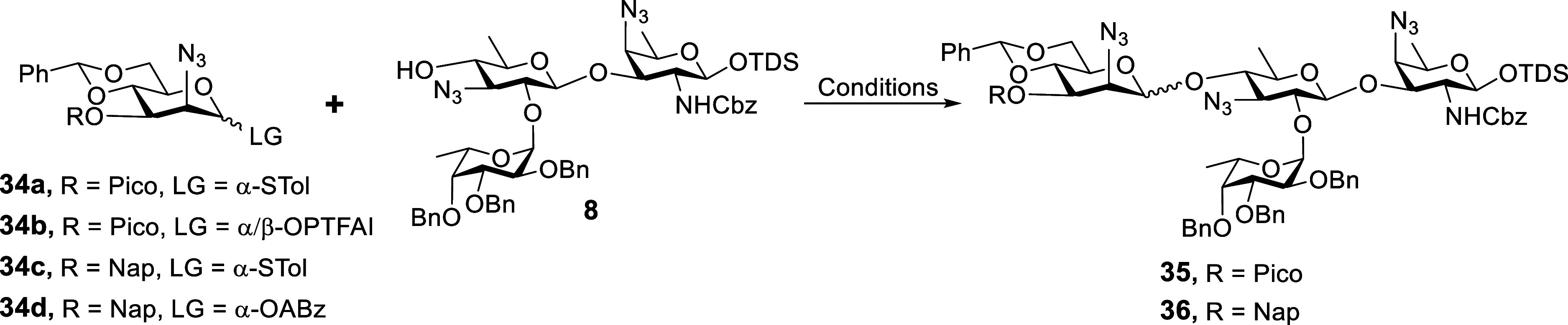
Stereoselective Construction of β-ManN_3_ Linkage

entry	donor	conditions	product	yield^a^ (β/α)^b^
1	**34a**	NIS/TfOH, CH_2_Cl_2_, 0 °C to r.t.	**35**	N.R.
2	**34b**	TBSOTf, CH_2_Cl_2_, 0 °C to r.t.	**35**	trace
3	**34c**	Ph_2_SO, Tf_2_O, TTBP, CH_2_Cl_2_, –60 °C to r.t.	**36**	30% (β/α = 2.5/1)
4	**34d**	Ph_3_PAuOTf, PhMe, –40–0 °C	**36**	89% (β/α = 3.5/1)
5	**34d**	Ph_3_PAuOTf, PhCl, –40–0 °C	**36**	86% (β/α = 5/1)

a Isolated yield.

b The β/α ratio was determined by the isolated yield ratio. N.R., no reaction; Ph_2_SO, diphenylsulfoxide; TTBP, 2,4,6-tri-*tert*-butylpyrimidine.

With the β-tetrasaccharide **36β** in hand, the Nap group was oxidatively cleaved by DDQ in DCM/PBS buffer^31,42^ to afford the glycosyl acceptor **37** in 74% yield. Subsequently, the coupling of acceptor **37** and Hep-PTFAI donor **9** under the activation of TfOH smoothly generated fully protected pentasaccharide **38** as the only α-anomer (*J*_C1–H1_ = 172 Hz) in 75% yield due to the neighboring group participation of Fmoc. The global deprotection of **38** provided the desired pentasaccharide **2** through a similar sequential procedure as described for the preparation of **1**. Next, we conducted a detailed analysis of the NMR spectra of pentasaccharide **2**. ^1^H NMR data of ManNAc residue were excellently identical to the literature values,^13^ with the deviations of chemical shifts not exceeding 0.04 ppm (Scheme 4B and Table S2). These data provide strong evidence for the presence of a β-linked ManNAc residue in natural PS A2. Encouraged by this outcome, we proceeded to synthesize pentasaccharide **3** containing both β-ManNAc and β-Hep linkages.

**Scheme 4:**
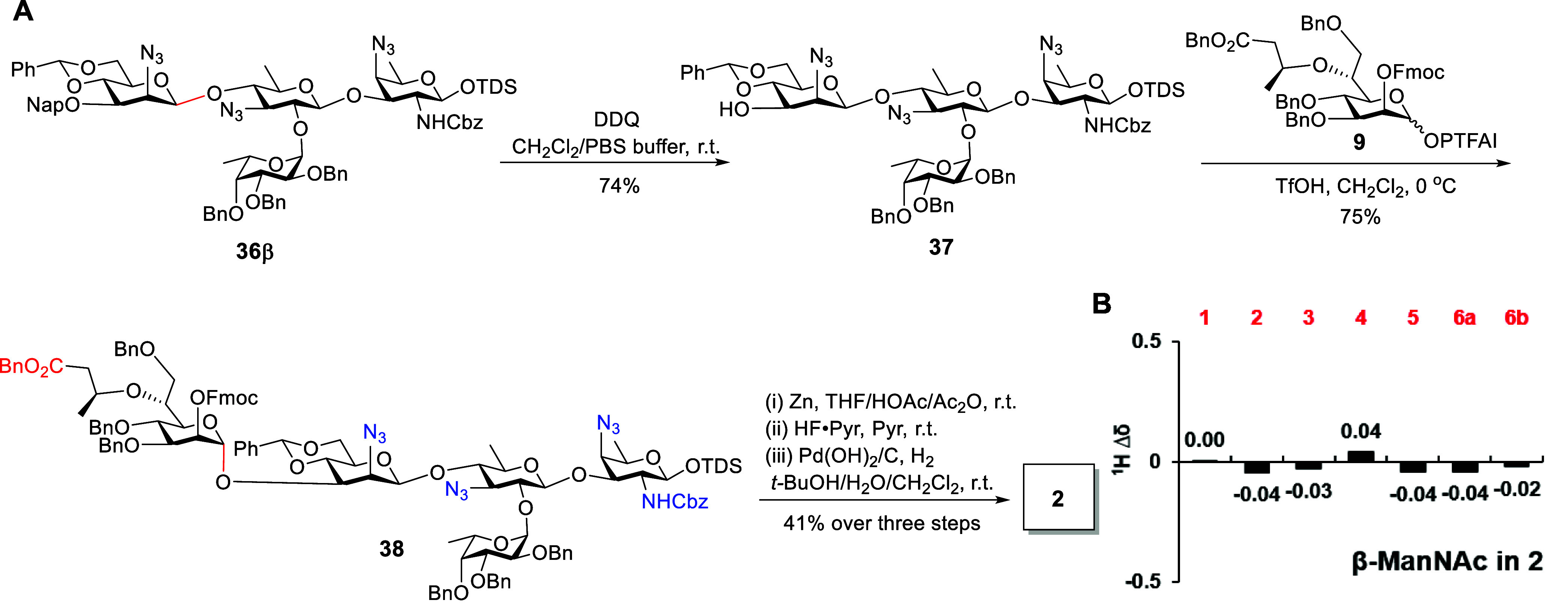
Synthesis of Pentasaccharide **2** (A) and Comparison of ^1^H NMR Chemical Shifts of β-ManNAc Residue in 2 (Δδ ppm) with Those of the Reported Data (B)

### Synthesis of Pentasaccharide **3** Containing Both β-ManNAc and β-Hep Linkages

To synthesize pentasaccharide **3**, we focused on constructing the challenging β-Hep linkage. Initially, we attempted two distinct strategies. First, we investigated silyl-mediated intramolecular aglycone delivery (IAD)^51,52^ for the coupling of Hep donor **21** with model acceptor **39**. Concurrently, we explored an indirect strategy involving C-2 epimerization of **21** followed by glycosylation with **39** (Scheme S14). Regrettably, neither strategy proved successful. The silyl-mediated IAD failed due to the inefficient formation of a silicon-tethered intermediate between the donor and acceptor, while the epimerization route was thwarted by unanticipated stereoselectivity issues during the glycosylation step (Scheme S14). Subsequently, we proceeded to explore direct β-heptosylation reactions via stereoinvertive substitution at the anomeric position of glycosyl Hep donors **40a**–**d** by acceptor **39** (Table 2). Yu glycosylation was first employed to access the β-heptoside.^27,53^ Unfortunately, the coupling of glycosyl ABz donor **40a** with acceptor **39** under different gold­(I) catalysts generated only α-linked product **41** (*J*_C1–H1_ = 171 Hz, Table 2 Entries 1–3). When glycosyl ABz donor **40b** (α/β = 5/1) containing an isopropylidene group at C-2/3-OH was tested, a small amount of β-disaccharide was generated (β/α = 1/7.5, *J*_C1–H1_ = 163 Hz for β*-*anomer, *J*_C1–H1_ = 174 Hz for α-anomer, Table 2 Entry 4). We postulated that the reactions might fail to proceed through S_N_2-type substitution via the 1-glycosyloxyisochromenylium-4-gold­(I) intermediate.^27^ Consequently, the reaction predominantly afforded α-linked products via an S_N_1 pathway, where the stereoselectivity was affected by the anomeric effect of the glycosyl donor. Given the success of tris­(pentafluorophenyl)­borane (B­(C_6_F_5_)_3_)-promoted stereoselective glycosylation of superarmed trichloroacetimidate (TCAI) donor,^28^ particularly with perbenzyl-mannosyl TCAI donors, the α-Hep-TCAI donor **40c** was examined for glycosylation with **39**. To our delight, a slightly improved β-selectivity (β/α = 1/4.5, *J*_C1–H1_ = 157 Hz for β*-*anomer) was observed under a catalytic amount of B­(C_6_F_5_)_3_ in DCM at −10 °C (Table 2 Entry 5). When the reaction temperature was decreased to −78 °C, the disaccharide **41** was obtained in 89% yield with a significant increase in β-selectivity (β/α = 2/1, Table 2, Entry 6). Furthermore, the glycosylation of **40d** and **39** in the presence of B­(C_6_F_5_)_3_ at −78 °C afforded the disaccharide **42** in 82% yield with better β-selectivity (β/α = 4/1, Table 2, Entry 7). This β-selectivity could be attributed to the acid–base catalyst B­(C_6_F_5_)_3_, which forms an adduct with the acceptor and stereoselectively activates the α-OTCAI glycosyl donor through β-face attack to afford β-glycoside.^28^ The β-anomer **42β** can be easily purified by silica gel column chromatography.

**Table 2:**
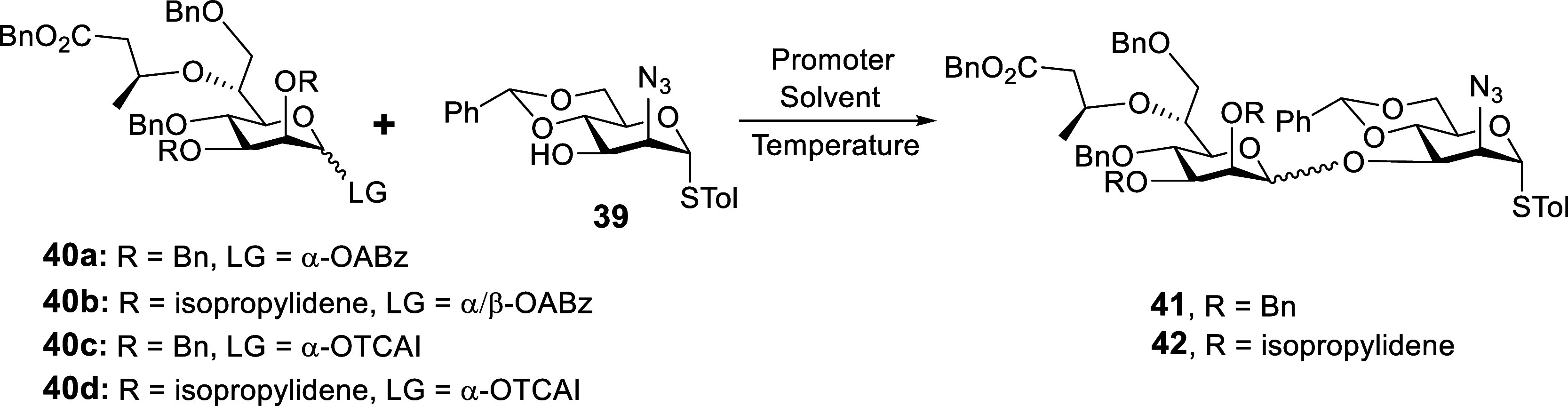
Stereoselective Construction of the β-Heptose Linkage

entry	donor	promoter	solvent	*T* (°C)	product	yield^a^ (β/α)^b^
1	**40a**	Ph_3_PAuOTf	PhCl	–40	**41**	73% (α only)
2	**40a**	Ph_3_PAuBAr_4_^F^	PhCl	–40 to r.t.	**41**	82% (α only)
3	**40a**	SPhosAuNTf_2_	PhCl	–40 to r.t.	**41**	53% (α only)
4	**40b**	Ph_3_PAuOTf	PhCl	–40	**42**	87% (β/α = 1/7.5)
5	**40c**	B(C_6_F_5_)_3_	CH_2_Cl_2_	–10	**41**	88% (β/α = 1/4.5)
6	**40c**	B(C_6_F_5_)_3_	CH_2_Cl_2_	–78	**41**	89% (β/α = 2/1)
7	**40d**	B(C_6_F_5_)_3_	CH_2_Cl_2_	–78	**42**	82% (β/α = 4/1)

a Isolated yield.

b The β/α ratio was determined by the isolated yield ratio. BAr_4_^F^, tetrakis­[3,5-bis­(trifluoromethyl)­phenyl]­borate; SPhos, 2-dicyclohexylphosphino −2′,6′-dimethoxy-1,1′-biphenyl.

With the β-linked heptosidic disaccharide **42β** in hand, we attempted to assemble the fully protected pentasaccharide through a [2 + 3] coupling approach based on Yu’s glycosylation protocol^27,49^ (Scheme S15). Unfortunately, only the α-ManN_3_ linkage (*J*_C1–H1_ = 175 Hz) was formed by the coupling of *ortho*-alkynylbenzoate disaccharide donor **S21** with trisaccharide acceptor **8** under the catalysis of Ph_3_PAuOTf. This outcome might be attributed to the steric hindrance effect of C-3-linked Hep residue in the ManN_3_ moiety, which constrained the β-face attack of acceptor **8**.^54^ Therefore, we designed an alternate synthetic route in which the pentasaccharide **3** would be assembled by a [1 + 4] coupling approach based on the established B­(C_6_F_5_)_3_-promoted β-heptosylation (Scheme 5A). The tetrasaccharide **37** was coupled with TCAI donor **41d** under B­(C_6_F_5_)_3_ activation at −78 °C, smoothly generating the β-linked heptosidic pentasaccharide **43** in an isolated yield of 61% (β/α = 4/1, *J*_C1–H1_ = 163 Hz for β*-*anomer). Next, we proceeded with the global deprotection of **43**. First, all azide groups were successfully transformed to NHAc using thioacetic acid (AcSH).^55^ Subsequently, the anomeric TDS group was cleaved by HF/pyridine, and the isopropylidene and benzylidene acetals were hydrolyzed with 80% HOAc. Finally, the hydrogenolytic cleavage of Bn and Cbz groups with Pd­(OH)_2_/C afforded the desired pentasaccharide **3**. As depicted in Scheme 5B,C, ^1^H NMR signals from the β-ManNAc residue correlated well with the recorded data of the natural PS A2. Compared with the α-linked Hep residue in **1** and **2**, the key H-1 and H-5 protons of β-linked Hep residue in **3** displayed a smaller ^1^H Δδ deviation relative to the reported values (Table S2).

**Scheme 5:**
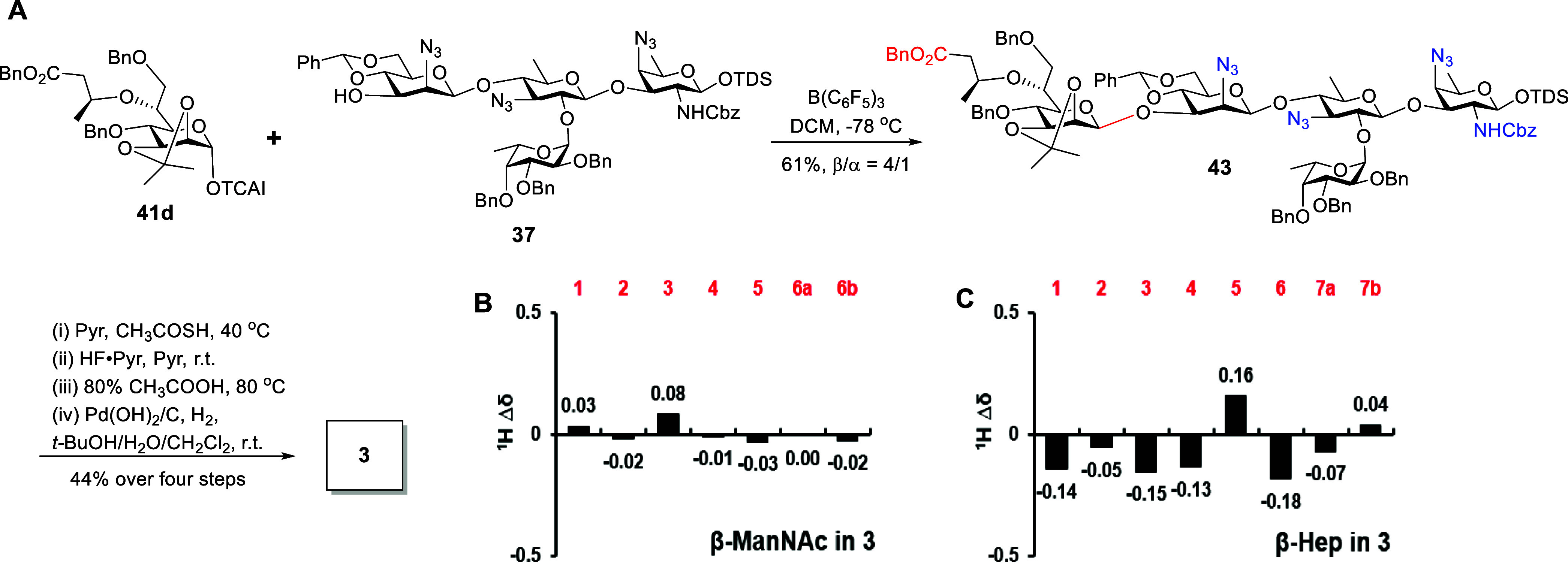
Synthesis of Pentasaccharide **3** (A) and Comparison of ^1^H NMR Chemical Shifts of β-ManNAc and β-Hep Residues in 3 (Δδ ppm) with Those of the Reported Data (B,C)

### Synthesis of Frame-Shift Trisaccharide **4** Containing β-Hep Linkage and Trisaccharide **5** Containing α-Hep Linkage for Comparative NMR Analysis of Hep Residue Deviations

In the native polysaccharide, the C-2-OH of the Hep residue is glycosidically linked to the AAT residue. To investigate the influence of AAT substitution on the NMR chemical shifts of the Hep residue, we synthesized trisaccharide **4** that mimics the natural sequence flanking the Hep unit (Scheme 6). For the introduction of the α-linked AAT residue, a new AAT building block **48** was synthesized, featuring 4-NHTCA and 2-N_3_ groups (Scheme S7). The 4-NHTCA group not only facilitates the formation of α-AAT linkage via remote neighboring group participation,^17,56–59^ but also serves as a precursor for subsequent acetamide (NHAc) installation. First, ManN_3_ acceptor **44** (Scheme S11) and Hep-TCAI donor **41d** were coupled in the presence of B­(C_6_F_5_)_3_, yielding the β-linked heptosidic disaccharide **45** in 63% yield (*J*_C1–H1_ = 160 Hz). Subsequent isopropylidene cleavage and regioselective C-3-OH benzylation of the Hep moiety provided compound **46**. Zinc-mediated reduction of the azide group of **46** generated an amine intermediate, which was acetylated by trichloroacetyl chloride (TCACl) to furnish disaccharide acceptor **47**. TfOH-promoted glycosylation of PTFAI donor **48** and acceptor **47** in a mixed solvent of CH_2_Cl_2_/Et_2_O afforded trisaccharide **49** in 71% yield with excellent α-selectivity (*J*_C1–H1_ = 175 Hz). Next, the target trisaccharide **4** was efficiently obtained via one-step Pd­(OH)_2_/C-catalyzed hydrogenation for converting NHTCA to NHAc^60^ and removing Bn and Nap groups. Meanwhile, trisaccharide **5** containing an α-linked Hep residue was synthesized for comparative NMR analysis (Scheme S18). As depicted in Scheme 6B, the sugar ring proton chemical shifts of β-linked Hep residue in **4** were closer to those of the native structure than β-linked Hep moiety in **3**. Notably, upon glycosylation with AAT, the H-1 proton of the α-linked Hep residue in **5** underwent a significant downfield shift (Scheme 6C), resulting in a larger discrepancy compared to the literature value. These results revealed that α-linked AAT at the C-2-OH of the Hep moiety significantly influenced the ^1^H NMR signals of the Hep residue. Additionally, ^13^C NMR data from the sugar ring carbon of the β-linked Hep residue in compound **3** also aligned more closely with those of the native structure (Table S5). Nevertheless, compared to the native structure, persistent deviations were observed in the H/C-6 signals of the Hep residue and its attached 3-hydroxybutanoic acid (3Hb) side chain across all synthetic oligosaccharides (**1–5**) (Tables S2–S5), which may originate from conformational variations^13,16,19,61,62^ in the native polysaccharide induced by the 3Hb modification. Collectively, the NMR signals of the β-Hep residue aligned more closely with the data obtained from the natural isolate.

**Scheme 6:**
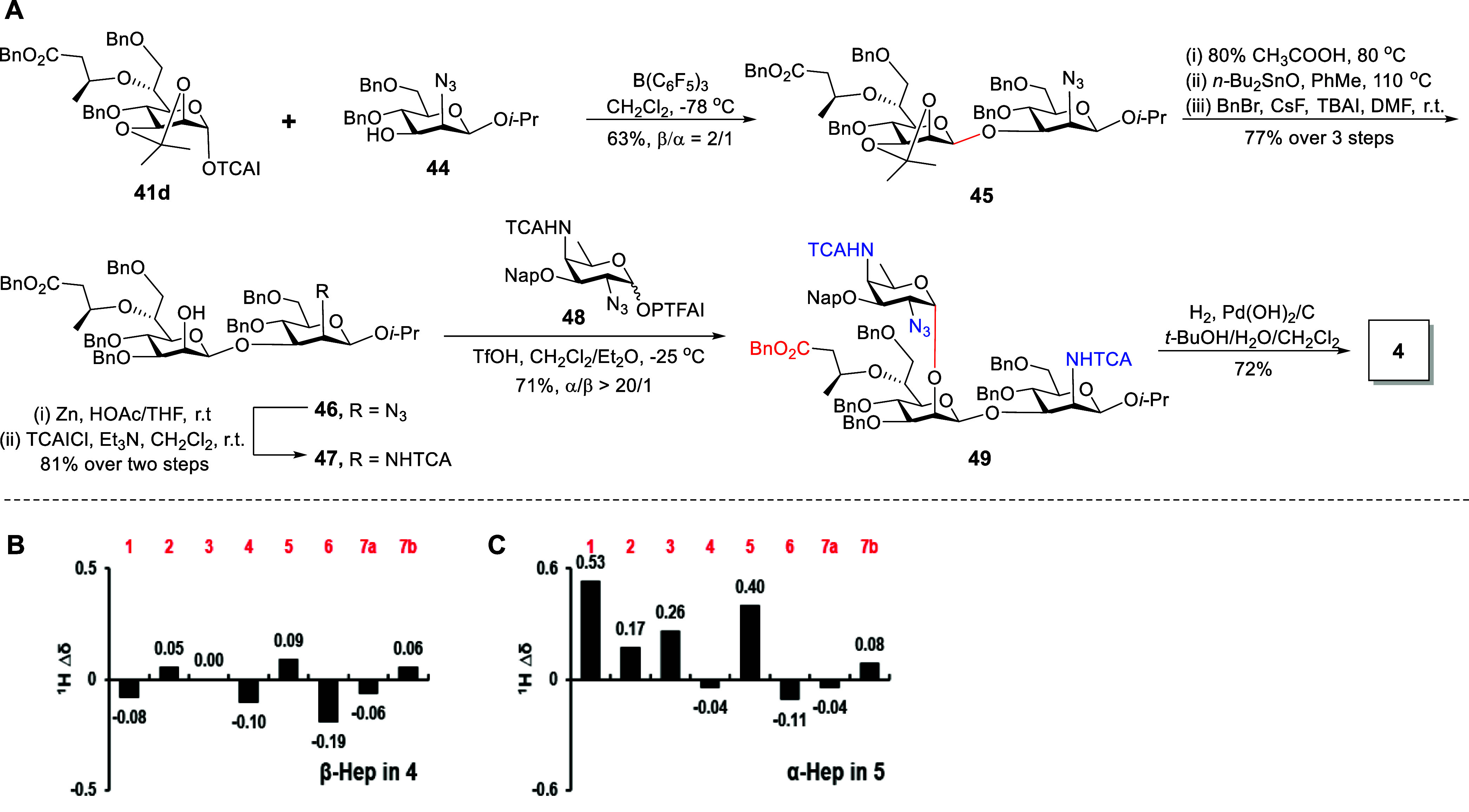
Synthesis of Trisaccharide **4** (A) and Comparison of the ^1^H NMR Chemical Shifts of β-Hep Residue in 4 and α-Hep Residue in 5 (Δδ ppm) with Those of the Reported Data (B, C)

## Conclusion

In summary, we have achieved the first total synthesis of the pentasaccharide repeating unit **1** from the reported structure of zwitterionic PS A2 via a stereocontrolled convergent [2 + 3] strategy. Efficient access to orthogonally protected rare-sugar building blocks made it possible to stereoselectively construct different glycosidic bonds for the assembly of the pentasaccharide and chemoselectively differentiate amino groups for acylation. In particular, the regioselective installation of a distinctive ether-linked 3-hydroxybutanoic acid onto the Hep moiety was realized by the Arndt-Eistert homologation reaction. More importantly, by comparing with the NMR data of synthetic **1** with native PS A2 and re-examining the original NOESY spectrum, we proposed that the anomeric configurations of ManNAc and Hep residues should be β-linkages. Consequently, pentasaccharides **2** and **3** containing β-linked ManNAc and/or Hep residues, as well as the corresponding frame-shift trisaccharides **4** and **5,** have been synthesized to verify our assumption. The stereoselective construction of the challenging 1,2-*cis*-β-ManNAc linkage and 1,2-*cis*-β-Hep linkage was achieved by Yu glycosylation and B­(C_6_F_5_)_3_-promoted glycosylation of the trichloroacetimidate (TCAI) donor, respectively. Additionally, comprehensive NMR analysis revealed that the β-ManNAc residue exhibited excellent agreement with reported NMR data, and the signals of β-Hep residue aligned more closely with the native PS A2 values, thereby revising and confirming anomeric stereochemical assignments of the polysaccharide. Collectively, this work delivers well-defined PS A2-related oligosaccharides for future immunological studies and establishes robust synthetic methodologies for accessing complex zwitterionic glycans, facilitating the development of carbohydrate-based therapeutics.

## Methods

### Materials

Organic reactions were performed under an atmosphere of argon using anhydrous solvents, unless otherwise stated. All chemicals were purchased commercially and used without further purification. Thin-layer chromatography (TLC) was carried out on Merck silica gel 60 F_254_-coated aluminum plates, and visualization was accomplished with 254 nm UV light and by staining with 10% sulfuric acid in ethanol followed by heating. Flash column chromatography was performed on a normal-phase silica column. Reversed phase chromatography was performed on a C18 silica gel column. Size-exclusion chromatography was performed on a Sephadex LH-20 and Bio-Gel P-4 column. Molecular sieves were activated before use.

### General Method

^1^H and ^13^C NMR spectra were recorded on Bruker Avance 400, 500, 600, and 800 spectrometers. Chemical shifts for ^1^H and ^13^C NMR spectra are reported in ppm (δ) relative to residual protium and carbon resonances in the solvent. Multiplicities were given as singlet (s), doublet (d), doublet of doublets (dd), triplet (t), quartet (q), or multiplet (m). Spectra were assigned using COSY, HSQC, TOCSY, HMBC, and NOESY experiments. The stereochemistry of glycosidic linkage was confirmed by the coupling constant between the anomeric proton and C2-proton (*J*_H1–H2_), the coupling constant between the anomeric carbon and proton (*J*_C1–H1_), and the NOE correlations. High-resolution mass spectrometry (HRMS) was measured on an ESI apparatus using an Agilent 1290 G6460A Q-TOF. MALDI-MS data were recorded on an Autoflex II MALDI-TOF (Bruker Daltonics) system instrument.

### Global Deprotection Procedure for the Synthesis of Pentasaccharides **1** and **2**

The fully protected pentasaccharide was dissolved in THF/Ac_2_O/AcOH (3:2:1, v/v/v, 2.4 mL), followed by the addition of Zn dust under an atmosphere of argon. The reaction mixture was stirred vigorously at room temperature overnight. MAIDI-TOF MS analysis showed conversion of the starting material to a major NHAc product. The reaction was quenched with MeOH and filtered through Celite. The filtrate was concentrated, diluted with CH_2_Cl_2_, and washed with a saturated NaHCO_3_ solution. The organic layer was dried over Na_2_SO_4_ and concentrated under reduced pressure to afford an NHAc intermediate. The NHAc intermediate was dissolved in pyridine (10 mL), followed by the addition of HF/Pyridine (70%, 1 mL) at 0 °C. The reaction mixture was stirred at room temperature overnight. TLC analysis showed complete conversion of the starting material to a major hemiacetal product. The reaction was quenched with a saturated NaHCO_3_ solution and diluted with CH_2_Cl_2_. The organic phase was separated, washed with brine, and dried over Na_2_SO_4_. The filtration was concentrated under reduced pressure and purified using preparative thin-layer chromatography to give the hemiacetal intermediate. To a solution of the hemiacetal intermediate in *t*-BuOH/H_2_O/CH_2_Cl_2_ (3/2/0.5, v/v/v, 5.5 mL) was added Pd­(OH)_2_/C (20%). The mixture was stirred at room temperature under a H_2_ atmosphere until ESI-MS analysis showed the complete conversion of the starting material to a major product. The reaction mixture was filtered through Celite, and the filtrate was concentrated under reduced pressure to give a crude product, which was sequentially purified by reverse-phase silica column (C-18) and size-exclusion chromatography (Bio-Gel P-4). The product-containing fractions were combined and lyophilized to afford the target product.

### Global Deprotection Procedure for the Synthesis of Pentasaccharide **3**

The fully protected pentasaccharide was dissolved in a mixed solvent of pyridine/AcSH (1/1, v/v, 1 mL), and the mixture was stirred at 40 °C for 48 h under an atmosphere of argon. The ESI-MS analysis showed conversion of the starting material to a major NHAc product. The reaction was concentrated *in vacuo*, and the crude product was coevaporated three times with toluene. The resulting residue was purified by size exclusion chromatography (Sephadex LH-20) to give the NHAc intermediate. The subsequent deprotection of the TDS group and the hydrogenation are similar to those employed in the synthesis of compounds **1** and **2**.

### Global Deprotection Procedure for the Synthesis of Trisaccharides **4** and **5**

To a solution of fully protected trisaccharide in *t*-BuOH/H_2_O/CH_2_Cl_2_ (4/1/1, v/v/v, 3 mL) was added Pd­(OH)_2_/C (20%). The mixture was stirred at room temperature under H_2_ atmosphere until ESI-MS analysis showed the complete conversion of the starting material to a major product. The reaction mixture was filtered through Celite, and the filtrate was concentrated under reduced pressure to give a crude product, which was sequentially purified by a reverse-phase silica column (C-18) and size-exclusion chromatography (Bio-Gel P-4). The product-containing fractions were combined and lyophilized to afford the target product.

## Supplementary Material



## Notes

Detailed experimental procedures, compound characterization data, additional schemes, figures, and tables, and copies of NMR and HRMS spectra (PDF)
