# Supplementary material for: Total Synthesis and Anomeric Configuration Revision of Zwitterionic Polysaccharide A2’s Pentasaccharide Repeating Unit from Bacteroides fragilis
Source: JACS Au. 2025 Oct 24;5(11):5555–67. doi: 10.1021/jacsau.5c01070 (PMC12648304; doi:10.1021/jacsau.5c01070)
Supplement: Supplementary file 1 [file au5c01070_si_001.pdf]

Supporting Information

Total Synthesis and Anomeric Configuration Revision of Zwitterionic Polysaccharide A2 Pentasaccharide Repeating Unit from *Bacteroides fragilis*

Tianhui Hao[a,b], Liangwei Zhang[a], and Tiehai Li[a,b]*

[a]*State Key Laboratory of Chemical Biology, Shanghai Institute of Materia Medica, Chinese Academy of Sciences, Shanghai 201203, China*

[b]*University of Chinese Academy of Sciences, Beijing 100049, China*

*E-mail: tiehaili@simm.ac.cn

Table of Contents

1. **General materials and methods** **2**
2. **NMR data analysis of the natural PS A2 and synthetic compounds 1-5** **3**
3. **Synthesis of monosaccharide building blocks.** **10**
4. **Synthesis of pentasaccharide 1** **46**
5. **Synthesis of pentasaccharide 2** **55**
6. **Synthesis of pentasaccharide 3** **63**
7. **Synthesis of trisaccharides 4 and 5** **73**
8. **Reference** **83**
9. **NMR and HRMS spectrum** **85**

1. General materials and methods

Organic reactions were performed under an atmosphere of argon using anhydrous solvents unless otherwise noted. Thin-layer chromatography (TLC) was carried out on Merck silica gel 60 F_{254}-coated aluminum sheets. TLC plates were detected with UV absorption (254 nm) and sprayed with 10% sulfuric acid in ethanol (1:9, v/v), followed by heating for visualization. Flash column chromatography was performed on a normal-phase silica column. Reversed phase chromatography was performed on a C18 silica gel column. Size-exclusion chromatography was performed on Sephadex LH-20 and Bio-Gel P4 column. Molecular sieves were activated before use. Chemical reagents were purchased from *J&K* Scientific Ltd. and TCI Shanghai, China. Proton nuclear magnetic resonance (^{1}H NMR) spectra were recorded on a Bruker Avance 400 (at 400 MHz), a Bruker Avance 500 (at 500 MHz), a Bruker Avance 600 (at 600 MHz) and a Bruker Avance 800 (at 800 MHz). Carbon nuclear magnetic resonance (^{13}C NMR) spectra were recorded on a Bruker Avance 400 (at 101 MHz), a Bruker Avance 500 (at 126 MHz), a Bruker Avance 600 (at 151 MHz) and a Bruker Avance 800 (at 201 MHz). The ^{1}H and ^{13}C NMR spectra were calibrated against the proton and carbon signals of the solvents as internal references ($CDCl_3$: δ_H = 7.26 ppm and δ_C = 77.16 ppm; Acetone-d_6: δ_H = 2.05 ppm and δ_C = 29.84 ppm; CD_3OD: δ_H = 3.31 ppm and δ_C = 49.00 ppm; D_2O: δ_H = 4.79 ppm). Multiplicities were given as singlet (s), doublet (d), doublet of doublets (dd), triplet (t), quartet (q), or multiplet (m). Spectra were assigned using COSY, HSQC, TOCSY, HMBC and NOESY experiments. The stereochemistry of glycosidic linkage was confirmed by the coupling constant between the anomeric proton and C2-proton ($J_{H1\text{-}H2}$), coupling constant between the anomeric carbon and proton ($J_{C1\text{–}H1}$), and NOE correlations. High-resolution mass spectrometry (HRMS) was measured on an ESI apparatus using an Agilent 1290 G6460A Q-TOF. MALDI-MS data were recorded on an Autoflex II MALDI-TOF (Bruker Daltonics) system instrument.

2. NMR data analysis of the natural PS A2 and synthetic compounds 1-5

2.1 NMR data of natural PS A2

Table S1. ^{1}H and ^{13}C chemical shift assignments of natural PS A2[1]

Residue			1	2	3	4	5	6	7
**a**	**AAT**	^{1}**H**	5.26	3.99	4.32	4.46	4.59	1.10	
		^{13}**C**	98.41	50.92	74.25	55.27	66.56	17.08	
**b**	**Fuc**	^{1}**H**	4.97	3.69	3.59	3.65	4.14	1.17	
		^{13}**C**	100.18	69.06	71.02	72.97	67.80	16.87	
**c**	**Hep**	^{1}**H**	4.84	3.85	3.75	3.92	3.41	4.08	3.77
		^{13}**C**	97.43	76.21	75.17	67.69	76.33	70.03	64.03
**d**	**ManNAc**	^{1}**H**	4.79	4.60	3.95	3.56	3.44	3.84, 3.94	
		^{13}**C**	100.48	50.85	78.24	66.65	77.77	61.97	
**e**	**ADG**	^{1}**H**	4.70	3.48	4.01	3.43	3.54	1.29	
		^{13}**C**	103.81	76.83	56.60	82.84	73.11	18.14	
	**3Hb**	^{1}**H**		2.56	4.23	1.26			
		^{13}**C**	175.42	45.99	66.34	23.02			

Data from *Proc. Natl. Acad. Sci. U.S.A.* **2000**, *97*, 13478–13483. Copyright (2000) National Academy of Sciences, U.S.A.

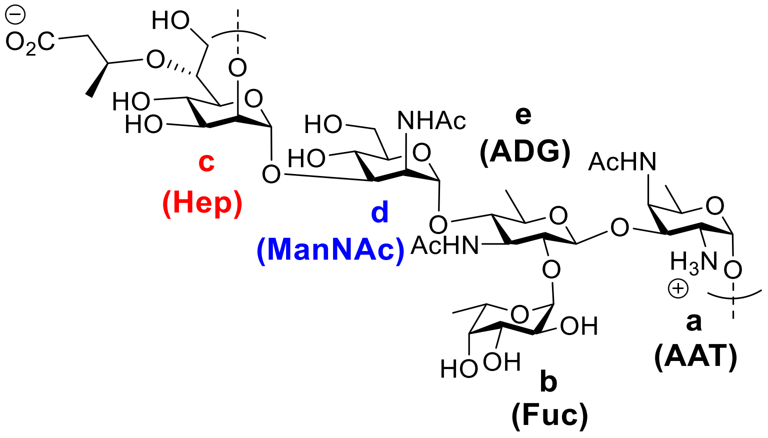

Figure S1. The reported structure of natural PS A2

2.2 The key NOE correlations in ManNAc and Hep residues of the natural PS A2

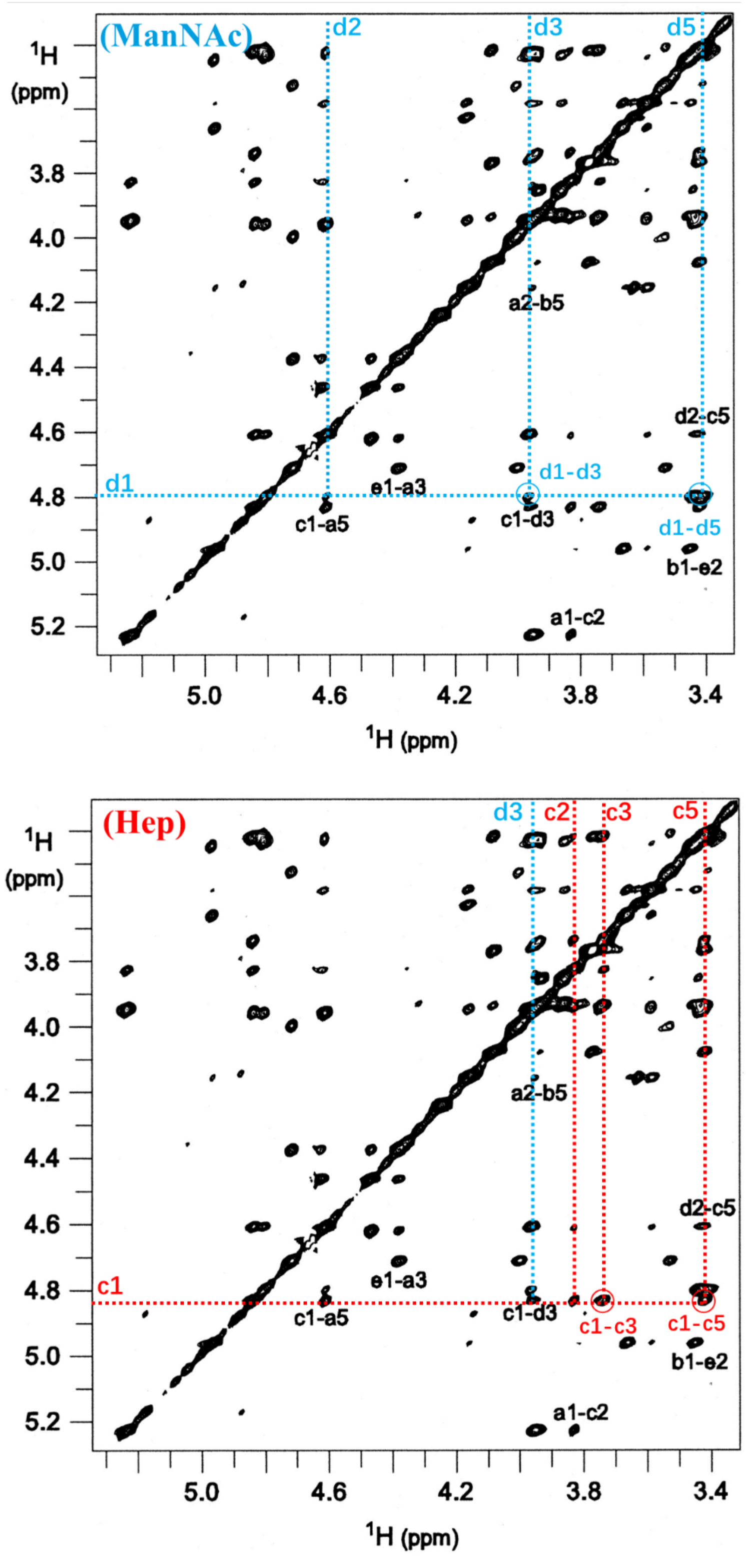

Figure S2. The key NOE correlations in the ManNAc (d) and Hep (c) residues of natural PS A2.

The NOESY spectrum from *Proc. Natl. Acad. Sci. U.S.A.* **2000**, *97*, 13478–13483. Copyright (2000) National Academy of Sciences, U.S.A.

2.3 Comparison of the NMR data of the synthetic compounds 1 – 5 and natural PS A2

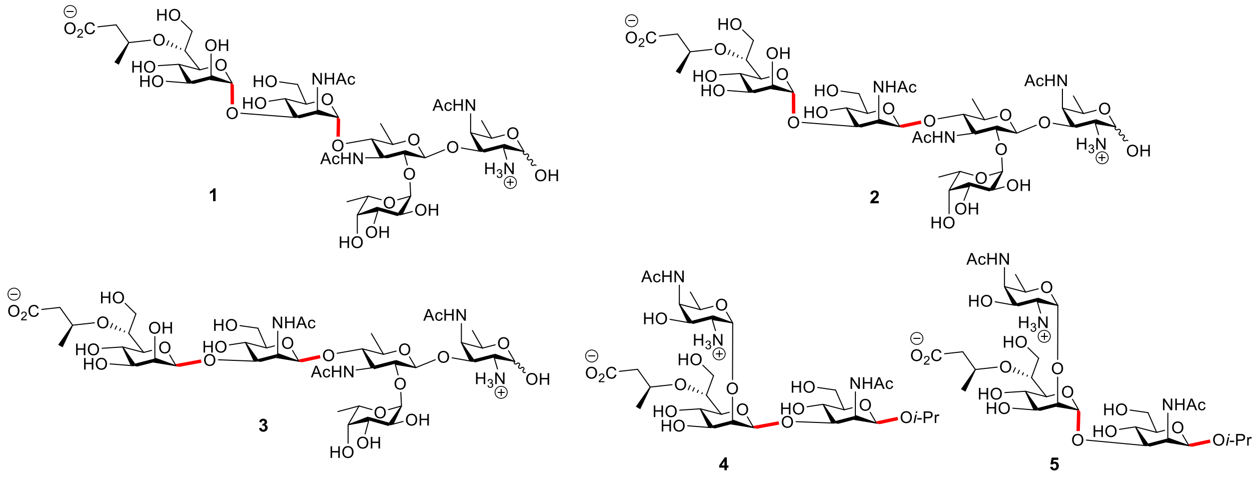

Figure S3. Synthetic compounds **1-5**

Table S2. Comparison of the ^{1}H NMR data of the synthetic compounds **1** – **3** and natural PS A2

		Natural PS A2	1		2		3	
		δ (ppm)	*δ* (ppm)	Δ*δ* (ppm)	*δ* (ppm)	Δ*δ* (ppm)	*δ* (ppm)	Δ*δ* (ppm)
α-AAT	H-1	5.26	5.41	0.15	5.41	0.15	5.41	0.15
	H-2	3.99	3.55	-0.44	3.59	-0.40	3.60	-0.39
	H-3	4.32	4.39	0.07	4.43	0.11	4.42	0.10
	H-4	4.46	4.55	0.09	4.53	0.07	4.43	-0.03
	H-5	4.59	4.42	-0.17	4.40	-0.19	4.40	-0.19
	H-6	1.10	1.10	0.00	1.08	-0.02	1.08	-0.03
ADG	H-1	4.70	4.76	**0.06**	4.73	**0.03**	4.73	**0.03**
	H-2	3.48	3.50	**0.02**	3.42	**-0.06**	3.43	**-0.05**
	H-3	4.01	4.07	**0.06**	3.92	**-0.09**	3.96	**-0.05**
	H-4	3.43	3.41	**-0.02**	3.44	**0.01**	3.43	**0.00**
	H-5	3.54	3.65	**0.11**	3.54	**0.00**	3.53	**-0.01**
	H-6	1.29	1.34	**0.05**	1.30	**0.01**	1.28	**-0.01**
Fuc	H-1	4.97	5.11	0.14	5.09	0.12	5.09	0.12
	H-2	3.69	3.76	0.07	3.72	0.03	3.73	0.04
	H-3	3.59	3.75	0.16	3.71	0.12	3.77	0.18
	H-4	3.65	3.79	0.14	3.76	0.11	3.76	0.11
	H-5	4.14	4.28	0.14	4.24	0.10	4.25	0.11
	H-6	1.17	1.23	0.06	1.20	0.03	1.21	0.04

ManNAc	**H-1**	4.79	4.99	**0.20**	4.79	**0.00**	4.81	**0.02**
	**H-2**	4.60	4.30	**-0.30**	4.55	**-0.05**	4.58	**-0.02**
	**H-3**	3.95	3.99	0.04	3.92	-0.03	4.03	0.08
	**H-4**	3.56	3.78	0.22	3.60	0.04	3.57	0.01
	**H-5**	3.44	3.76	**0.32**	3.40	**-0.04**	3.41	**-0.03**
	**H-6a**	3.84	3.85	0.01	3.80	-0.04	3.83	-0.01
	**H-6b**	3.94	3.85	-0.09	3.90	-0.04	3.92	-0.02
Hep	**H-1**	4.84	5.09	**0.25**	5.12	**0.28**	4.70	**-0.14**
	**H-2**	3.85	3.92	0.07	3.93	0.07	3.80	-0.05
	**H-3**	3.75	3.70	-0.06	3.63	-0.12	3.60	-0.15
	**H-4**	3.92	3.83	-0.09	3.73	-0.19	3.79	-0.13
	**H-5**	3.41	3.74	**0.33**	3.91	**0.50**	3.55	**0.14**
	**H-6**	4.08	3.88	-0.20	3.90	-0.18	3.90	-0.18
	**H-7a**	3.77	3.73	-0.04	3.71	-0.06	3.70	-0.07
	**H-7b**	3.77	3.73	-0.04	3.71	-0.06	3.81	0.04
3Hb	**H-2a**	2.56	2.60	0.04	2.64	0.08	2.53	-0.03
	**H-2b**	2.56	2.32	-0.24	2.28	-0.28	2.30	-0.26
	**H-3**	4.23	4.23	0.00	4.15	-0.08	4.06	-0.17
	**H-4**	1.26	1.26	0.00	1.24	-0.02	1.22	-0.04

$\Delta\delta = \delta$(synthetic compound) – δ(natural PS A2)

Table S3. Comparison of the ^{13}C NMR data of the synthetic compounds **1** – **3** and natural PS A2

		**Natural PS A2**	**1**		**2**		**3**	
		**δ (ppm)**	**δ (ppm)**	**$\Delta\delta$ (ppm)**	**δ (ppm)**	**$\Delta\delta$ (ppm)**	**δ (ppm)**	**$\Delta\delta$ (ppm)**
α-AAT	**C-1**	98.41	89.15	-9.26	89.23	-9.18	89.07	-9.34
	**C-2**	50.92	49.94	-0.98	50.36	-0.56	50.58	-0.34
	**C-3**	74.25	74.29	0.04	73.88	-0.37	74.16	-0.09
	**C-4**	55.27	52.33	-2.94	52.74	-2.53	52.94	-2.33
	**C-5**	66.56	65.03	-1.53	65.40	-1.16	65.55	-1.01
	**C-6**	17.08	15.09	-1.99	15.56	-1.52	15.70	-1.38
ADG	**C-1**	103.81	102.81	-1.00	102.81	-1.00	103.02	-0.79
	**C-2**	76.83	78.49	1.66	79.23	2.40	79.71	2.88

	**C-3**	56.60	55.00	-1.60	54.04	-2.56	53.86	-2.74
	**C-4**	82.84	80.74	-2.10	79.38	-3.46	80.67	-2.17
	**C-5**	73.11	71.53	-1.58	72.39	-0.72	72.33	-0.78
	**C-6**	18.14	17.27	-0.87	17.14	-1.00	16.92	-1.22
**Fuc**	**C-1**	100.18	99.03	-1.15	99.24	-0.94	99.20	-0.98
	**C-2**	69.06	68.66	-0.40	69.07	0.01	69.27	0.21
	**C-3**	71.02	71.56	0.54	71.70	0.68	72.09	1.07
	**C-4**	72.97	71.33	-1.64	71.72	-1.25	71.66	-1.31
	**C-5**	67.80	67.70	-0.10	68.20	0.40	67.97	0.17
	**C-6**	16.87	15.19	-1.68	15.48	-1.39	15.62	-1.25
**ManNAc**	**C-1**	100.48	100.09	-0.39	98.78	-1.70	99.03	-1.45
	**C-2**	50.85	52.06	1.21	52.60	1.75	50.19	-0.66
	**C-3**	78.24	75.21	-3.03	76.83	-1.41	77.63	-0.61
	**C-4**	66.65	65.59	-1.06	67.06	0.41	65.16	-1.49
	**C-5**	77.77	72.63	**-5.14**	76.22	**-1.55**	76.20	**-1.57**
	**C-6**	61.97	59.73	-2.24	60.65	-1.32	60.82	-1.15
**Hep**	**C-1**	97.43	101.27	3.84	101.35	3.92	97.10	-0.33
	**C-2**	76.21	69.58	-6.63	69.88	-6.33	70.60	-5.61
	**C-3**	75.17	70.31	-4.86	70.49	-4.68	73.20	-1.97
	**C-4**	67.69	66.06	-1.63	67.12	-0.57	67.31	-0.38
	**C-5**	76.33	73.32	**-3.01**	73.53	**-2.80**	76.30	**-0.03**
	**C-6**	70.03	78.49	8.46	77.70	7.67	77.40	7.37
	**C-7**	64.03	61.75	-2.28	61.02	-3.01	60.15	-3.88
**3Hb**	**C-1**	175.42	180.02	4.60	179.81	4.39	179.92	4.50
	**C-2**	45.99	45.19	-0.02	45.27	-0.72	45.51	-0.48
	**C-3**	66.34	73.90	7.56	74.03	7.69	74.16	7.82
	**C-4**	23.02	18.90	-4.12	19.31	-3.71	19.61	-3.41

$\Delta\delta = \delta$(synthetic compound) – δ(natural PS A2)

Table S4. Comparison of the ^{1}H NMR data of the synthetic compounds **4 - 5** and natural PS A2

		Natural PS A2	4		5	
		δ (ppm)	δ (ppm)	$\Delta\delta$ (ppm)	δ (ppm)	$\Delta\delta$ (ppm)
AAT	H-1	5.26	5.29	0.03	5.30	0.04
	H-2	3.99	3.39	-0.60	3.41	-0.58
	H-3	4.32	4.37	0.05	4.28	-0.04
	H-4	4.46	4.25	-0.21	4.32	-0.14
	H-5	4.59	4.56	-0.03	4.25	-0.34
	H-6	1.10	1.10	0.00	1.11	0.01
ManNAc	H-1	4.79	4.89	0.10	4.91	0.12
	H-2	4.60	4.57	-0.03	4.53	-0.07
	H-3	3.95	4.01	0.06	3.95	0.00
	H-4	3.56	3.54	-0.02	3.66	0.10
	H-5	3.44	3.43	-0.01	3.44	0.00
	H-6a	3.84	3.81	-0.03	3.81	-0.03
	H-6b	3.94	3.89	-0.05	3.89	-0.05
Hep	H-1	4.84	4.76	-0.08	5.37	0.53
	H-2	3.85	3.90	0.05	4.02	0.17
	H-3	3.75	3.75	0.00	4.01	0.26
	H-4	3.92	3.83	-0.09	3.87	-0.05
	H-5	3.41	3.50	0.09	3.81	0.40
	H-6	4.08	3.89	-0.19	3.97	-0.11
	H-7a	3.77	3.71	-0.06	3.73	-0.04
	H-7b	3.77	3.80	0.03	3.83	0.06
3Hb	H-2a	2.56	2.40	-0.16	2.67	0.11
	H-2b	2.56	2.48	-0.08	2.46	-0.10
	H-3	4.23	4.14	-0.09	4.22	-0.01
	H-4	1.26	1.22	-0.04	1.28	0.02

$\Delta\delta = \delta$(synthetic compound) – δ(natural PS A2)

Table S5. Comparison of the ^{13}C NMR data of the synthetic compounds **4 - 5** and natural PS A2

		**Natural PS A2**	**4**		**5**	
		***δ* (ppm)**	***δ* (ppm)**	**Δ*δ* (ppm)**	***δ* (ppm)**	**Δ*δ* (ppm)**
**AAT**	**C-1**	98.41	97.16	-1.26	97.79	-0.62
	**C-2**	50.92	51.46	0.54	51.24	0.32
	**C-3**	74.25	65.56	-8.69	65.79	-8.46
	**C-4**	55.27	53.19	-2.08	53.12	-2.15
	**C-5**	66.56	65.65	-0.91	66.18	-0.38
	**C-6**	17.08	15.75	-1.33	15.37	-1.71
**ManNAc**	**C-1**	100.48	97.16	-3.32	97.50	-2.98
	**C-2**	50.85	50.31	-0.54	53.24	2.39
	**C-3**	78.24	77.66	-0.58	76.73	-1.51
	**C-4**	66.65	65.09	-1.56	67.23	0.58
	**C-5**	77.77	76.37	-1.40	76.08	-1.69
	**C-6**	61.97	60.20	-1.77	60.10	-1.87
**Hep**	**C-1**	97.43	96.55	**-0.88**	99.52	**2.09**
	**C-2**	76.21	77.58	**1.37**	79.15	**2.94**
	**C-3**	75.17	73.36	**-1.81**	72.95	**-2.22**
	**C-4**	67.69	67.82	**0.13**	67.05	**-0.64**
	**C-5**	76.33	75.34	**-0.99**	70.00	**-6.33**
	**C-6**	70.03	78.69	8.66	77.79	7.76
	**C-7**	64.03	60.51	-3.52	61.26	-2.77
**3Hb**	**C-1**	175.42	179.29	3.87	177.47	2.05
	**C-2**	45.99	44.90	-1.09	43.57	-2.42
	**C-3**	66.34	74.08	7.74	73.04	6.70
	**C-4**	23.02	19.66	-3.36	19.04	-3.98

Δ*δ* = *δ*(synthetic compound) – *δ*(natural PS A2)

3. Synthesis of monosaccharide building blocks

3.1 Synthesis of Hep bilding blocks

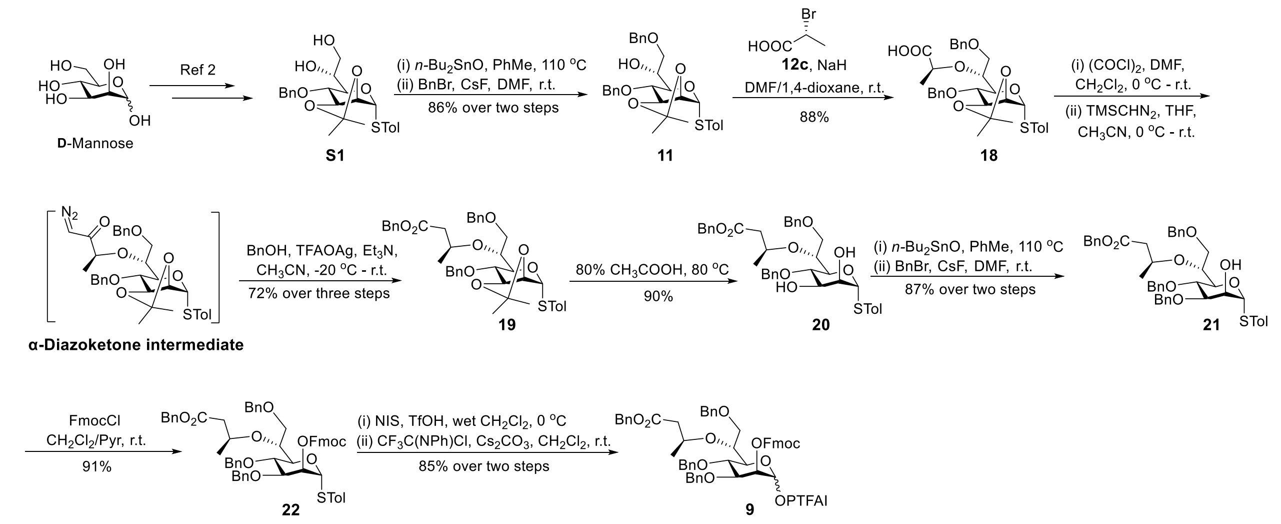

Scheme S1. Synthesis of Hep building block **9**

p-Tolyl 2,3-*O*-isopropylidene-4,7-di-*O*-benzyl-1-thio-D-*glycero*-*α*-D-*manno*-heptopyranoside (11)

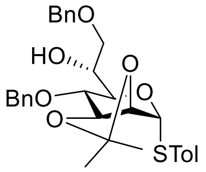

11

A mixture of **S1**[2] (2.3 g, 4.9 mmol) and dibutyltin oxide (n-Bu$_2$SnO, 1.5 g, 5.9 mmol) in PhMe (50 mL) was heated to 110 °C and stirred for 6 h. The reaction mixture was cooled to room temperature, and concentrated under reduced pressure to give a colorless oil for the next step. The resulting oil was dissolved in DMF (50 mL), and the BnBr (0.7 mL, 5.9 mmol) and cesium fluoride (CsF, 0.9 g, 5.9 mmol) were added. The reaction mixture was stirred at room temperature overnight under an atmosphere of argon. TLC analysis showed complete conversion of starting material **S1** to compound **11** (petroleum ether/ethyl acetate = 7/1, R_f = 0.40). The mixture was concentrated *in vacuo*. The resulting residue was dissolved with CH_2Cl_2, and the mixture was washed with 1 M HCl and brine. The organic phase was dried (Na_2SO_4), filtered, and the filtrate was concentrated *in vacuo*. The resulting residue was purified by silica gel column chromatography (petroleum ether/ethyl acetate = 10/1 to 8/1) to give compound **11** (2.4 g, 86%) as a colorless oil. ^{1}H NMR (600 MHz, $CDCl_3$) δ 7.50 – 7.27 (m, 12H, ArH), 7.10 (d, J = 8.0 Hz, 2H, ArH), 5.73 (s, 1H, H-1), 4.92 (d, J = 11.3 Hz, 1H, C*H*H-Bn), 4.61 (d, J = 11.2 Hz, 1H, C*H*H-Bn), 4.45 (d, J = 12.1 Hz, 1H, C*H*H-Bn), 4.38 – 4.33 (m, 3H, C*H*H-Bn, H-2, H-3), 4.15 (dd, J = 9.6, 4.6 Hz, 1H), 4.10 – 4.03 (m, 1H, H-6), 3.78 (dd, J

= 9.6, 6.5 Hz, 1H), 3.45 (dd, J = 10.0, 3.5 Hz, 1H, H-7a), 3.38 (dd, J = 10.0, 7.3 Hz, 1H, H-7b), 2.33 (s, 3H, CH_3-STol), 1.53 (s, 3H, C(CH_3)$_2$), 1.40 (s, 3H, C(CH_3)$_2$); ^{13}C NMR (151 MHz, $CDCl_3$) δ 138.24, 137.89, 137.75, 132.31, 129.93, 129.23, 128.45, 128.34, 128.18, 127.88, 127.69, 127.57, 109.68 (C(CH_3)$_2$), 84.15 (C-1), 78.50, 77.41, 76.12, 73.27, 72.80, 71.75, 71.06, 69.85, 27.99, 26.41, 21.17. HRMS (ESI): m/z calcd for $C_{31}H_{36}NaO_6S^+$ $[M+Na]^+$ 559.2125, found 559.2124.

***p*-Tolyl 2,3-*O*-isopropylidene-4,7-di-*O*-benzyl-6-*O*-[2(*S*)-lactic acid]-1-thio-D-*glycero*-*α*-D-*manno*-heptopyranoside (18)**

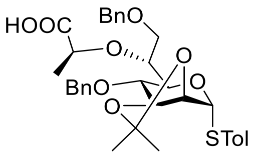

18

To a solution of compound **11** (1.5 g, 2.8 mmol) in anhydrous DMF/1,4-dioxane (v/v, 1/1, 50 mL) was slowly added NaH (60% in mineral oil, 1.1 g, 28 mmol) at 0 °C under an atmosphere of argon. The reaction was vigorously stirred at 0 °C for 20 min. Then, the (*R*)-2-bromopropionic acid (1.3 mL, 14 mmol) was added dropwise using syringe.[3–5]. After being stirred at room temperature for 6 h, TLC analysis showed conversion of starting material **11** to compound **18** (petroleum ether/ethyl acetate = 2/1, R_f = 0.10). The reaction was carefully quenched with H_2O at 0 °C and diluted with CH_2Cl_2. The aqueous phase was re-extracted twice with CH_2Cl_2. The combined organic phase was acidified to pH 3 with 2.5 M aqueous HCl, washed with brine, dried (Na_2SO_4), and filtered. The filtrate was concentrated under reduced pressure and resulting residue was purified by silica gel column chromatography (petroleum ether/ethyl acetate = 20/1 to 10/1) to give compound **18** (1.5 g, 88%) as a light yellow oil. ^{1}H NMR (600 MHz, $CDCl_3$) δ 7.39 – 7.28 (m, 10H, ArH), 7.25 – 7.22 (m, 2H, ArH), 7.12 (d, J = 8.0 Hz, 2H, ArH), 5.75 (s, 1H, H-1-Hep), 4.91 (d, J = 11.4 Hz, 1H, CHH-Bn), 4.55 (d, J = 11.4 Hz, 1H, CHH-Bn), 4.43 – 4.37 (m, 2H), 4.37 – 4.32 (m, 3H), 4.23 (dd, J = 9.9, 2.2 Hz, 1H), 3.86 – 3.81 (m, 1H), 3.81 – 3.73 (m, 1H), 3.63 – 3.49 (m, 2H), 2.34 (s, 3H, CH_3-STol), 1.52 (s, 3H, C(CH_3)$_2$), 1.44 (d, J = 6.9 Hz, 3H, CH_3-lactic acid), 1.39 (s, 3H, C(CH_3)$_2$); ^{13}C NMR (151 MHz, $CDCl_3$) δ 174.45 (C=O-COOH), 138.34, 137.77, 137.74, 132.05, 130.22, 128.82, 128.59, 128.50, 128.27, 127.94, 127.80, 110.04 (C(CH_3)$_2$), 84.30 (C-1-Hep), 81.20, 78.61, 76.40, 75.92, 75.22, 73.57, 72.58, 71.81, 69.93, 28.01, 26.59, 21.25, 18.93. HRMS (ESI): m/z calcd for $C_{34}H_{40}NaO_8S^+$ $[M+Na]^+$ 632.2336, found 632.2334.

p-Tolyl 2,3-O-isopropylidene-4,7-di-O-benzyl-6-O-[benzyl 3(S)-butanoate]-1-thio-D-*glycero*-α-D-*manno*-heptopyranoside (19)[6]

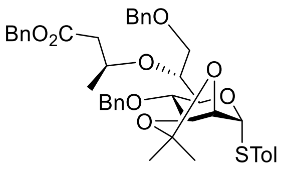

19

To a solution of acid **18** (2.4 g, 3.9 mmol) in anhydrous CH_2Cl_2 (50 mL) was added a drop of anhydrous DMF and $(COCl)_2$ (0.67 mL, 7.9 mmol) at 0 °C under an atmosphere of argon. The reaction was stirred at room temperature for 40 min. [The reaction progress was monitored by taking a drop of reaction mixture and quenching with methanol in a centrifuge tube, converting the acid chloride intermediate to its methyl ester derivative. (methyl ester derivative: petroleum ether/ethyl acetate = 4/1, R_f= 0.55)]. The reaction mixture was concentrated *in vacuo* to give crude acid chloride product as a yellow oil.

To a solution of $TMSCHN_2$ (2 M in hexane, 7.9 mL, 15.8 mmol) in anhydrous THF (10 mL) was slowly added the above acid chloride dissolved in a mixed solvent of anhydrous THF/CH_3CN (v/v, 1/1, 50 mL) at 0 °C under an atmosphere of argon. After being stirred at room temperature for 1 h, TLC analysis showed complete conversion to diazo ketone intermediate (petroleum ether/ethyl acetate = 4/1, R_f= 0.45). The reaction was quenched with 0.5 M aqueous lemon acid at 0 °C until bubbling subsided and diluted with CH_2Cl_2. The aqueous layer was separated and re-extracted twice with CH_2Cl_2, and the combined organic layer was washed with saturated aqueous $NaHCO_3$ and brine. The organic phase was dried (Na_2SO_4), and filtered. The filtrate was concentrated *in vacuo* to give the crude diazo ketone intermediate as a yellow oil for the next step.

To a solution of above diazo ketone and anhydrous BnOH (2.1 mL, 19.7 mmol) in a mixed solvent of anhydrous CH_3CN (50 mL) at -20 °C under an atmosphere of argon and in the dark, was added a solution of AgOTFA (0.26 g, 1.2 mmol) in anhydrous Et_3N (5 mL). The reaction was gradually warmed to room temperature and stirred for another 4 h. TLC analysis showed complete conversion of diazo ketone intermediate to a major product **19** (petroleum ether/ethyl acetate = 4/1, R_f= 0.80) and minor byprocuct **11**. The reaction was quenched with 1 M aqueous HCl and diluted with CH_2Cl_2. The aqueous layer was separated and re-extracted twice with CH_2Cl_2, and the combined organic layer was washed with saturated aqueous $NaHCO_3$ and brine. The organic phase was dried (Na_2SO_4), and filtered. The filtrate was concentrated *in vacuo* to give the crude product. The crude product was purified by silica gel column chromatography (petroleum ether/ethyl acetate = 25/1 to 20/1) to give compound **19** (2.15 g, 72% over three steps) as a colorless oil. ^1H NMR (600 MHz, $CDCl_3$) δ 7.39 (d, J = 8.2 Hz, 2H, ArH), 7.35

– 7.19 (m, 15H, ArH), 7.05 (d, *J* = 8.2 Hz, 2H, ArH), 5.56 (s, 1H, H-1-Hep), 5.12 – 4.93 (m, 2H, C*H*$_2$Bn), 4.83 (d, *J* = 11.4 Hz, 1H, C*H*H-Bn), 4.56 (d, *J* = 11.4 Hz, 1H, C*H*H-Bn), 4.48 – 4.36 (m, 2H, C*H*$_2$Bn), 4.29 – 4.27 (m, 2H, H-2-Hep, H-3-Hep), 4.23 (dd, *J* = 10.0, 1.7 Hz, 1H, H-5-Hep), 4.12 – 4.09 (m, 1H, H-3-3Hb), 3.90 (dd, *J* = 6.9, 4.8 Hz, 1H, H-6), 3.79 – 3.69 (m, 1H, H-4-Hep), 3.57 (dd, *J* = 10.3, 6.9 Hz, 1H, H-7a-Hep), 3.50 (dd, *J* = 10.3, 4.8 Hz, 1H, H-7b-Hep), 2.65 (dd, *J* = 15.1, 6.1 Hz, 1H, H-2a-3Hb), 2.37 (dd, *J* = 15.1, 7.1 Hz, 1H, H-2b-3Hb), 2.28 (s, 3H, C*H*$_3$-STol), 1.46 (s, 3H, C(C*H*$_3$)$_2$), 1.34 (s, 3H, C(C*H*$_3$)$_2$), 1.20 (d, *J* = 6.1 Hz, 3H, C*H*$_3$-3Hb); ^{13}C NMR (151 MHz, CDCl$_3$) δ 171.28 (*C*=O-COOBn), 138.63, 137.96, 136.18, 133.08, 129.81, 128.58, 128.38, 128.35, 128.23, 128.13, 127.59, 109.55 (*C*(CH$_3$)$_2$), 84.90 (C-1-Hep), 78.99, 77.23, 76.37, 76.15, 73.27, 72.94, 72.61, 71.24, 71.10, 66.23, 42.32, 27.99, 26.59, 21.25, 20.68. HRMS (ESI): m/z calcd for $C_{42}H_{48}NaO_8S^+$ [M+Na]$^+$ 735.2962, found 735.2958.

***p*-Tolyl 4,7-di-*O*-benzyl-6-*O*-[benzyl 3(*S*)-butanoate]-1-thio-D-*glycero*-*α*-D-*manno*-heptopyranoside (20)**

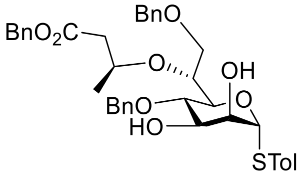

20

The compound **19** (2.0 g, 2.8 mmol) was treated with 80% AcOH in water (40 mL) and the mixture was heated to 80 ºC for 8 h. TLC analysis showed complete conversion of starting material **19** to compound **20** (petroleum ether/ethyl acetate = 2/1, R$_f$ = 0.25). The reaction mixture was cooled to room temperature and concentrated under reduced pressure to give crude product. The resulting crude product was purified by silica gel column chromatography (petroleum ether/ethyl acetate/CH$_2$Cl$_2$= 2.5/1/1) to give compound **20** (1.7 g, 90%) as a colorless oil. ^{1}H NMR (600 MHz, CDCl$_3$) δ 7.38 – 7.25 (m, 17H, ArH), 7.09 (d, *J* = 8.2 Hz, 2H, ArH), 5.44 (d, *J* = 1.8 Hz, 1H, H-1-Hep), 5.17 (d, *J* = 12.3 Hz, 1H, C*H*H-Bn), 5.09 (d, *J* = 12.3 Hz, 1H, C*H*H-Bn), 4.80 (d, *J* = 11.1 Hz, 1H, C*H*H-Bn), 4.54 (d, *J* = 11.0 Hz, 1H, C*H*H-Bn), 4.47 – 4.46 (m, 2H, C*H*$_2$-Bn), 4.29 – 4.27 (m, 2H, H-5-Hep, H-3-3Hb), 4.06 (dd, *J* = 3.6, 1.8 Hz, 1H, H-2-Hep), 3.95 (dd, *J* = 9.0, 3.5 Hz, 1H, H-3-Hep), 3.90 – 3.84 (m, 1H, H-6-Hep), 3.79 (t, *J* = 9.3 Hz, 1H, H-4-Hep), 3.61 (dd, *J* = 10.2, 5.0 Hz, 1H, H-7a-Hep), 3.52 (dd, *J* = 10.2, 7.5 Hz, 1H, H-7b-Hep), 2.63 (dd, *J* = 15.6, 8.7 Hz, 1H, H-2a-3Hb), 2.51 (dd, *J* = 15.6, 3.8 Hz, 1H, H-2b-3Hb), 2.33 (s, 3H, C*H*$_3$-STol), 1.24 (d, *J* = 6.1 Hz, 3H, C*H*$_3$-3Hb); ^{13}C NMR (151 MHz, CDCl$_3$) δ 172.73 (*C*=O-COOBn), 138.52, 138.48, 137.90, 135.78, 132.71, 130.18, 129.93, 128.70, 128.46, 128.41, 128.35, 128.09, 127.70, 127.62, 127.59, 88.80 (C-1-Hep), 78.62, 76.24, 74.54, 73.32, 73.13, 73.07, 72.79, 72.14, 66.77, 42.41, 21.23, 20.45. HRMS (ESI): m/z calcd

for $C_{39}H_{44}NaO_8S^+$ $[M+Na]^+$ 695.2649, found 695.2649.

***p*-Tolyl 3,4,7-tri-*O*-benzyl-6-*O*-[benzyl 3(*S*)-butanoate]-1-thio-D-*glycero*-*α*-D-*manno*-heptopyranoside (21)**

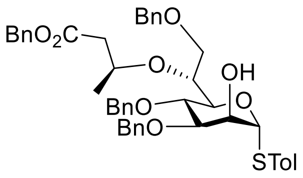

21

A mixture of **20** (1.6 g, 2.7 mmol) and dibutyltin oxide (800 mg, 3.2 mmol) in PhMe (25 mL) was heated to 110 ºC and stirred for 6 h. The reaction mixture was cooled to room temperature, and concentrated under reduced pressure to give a colorless oil for the next step. The resulting oil was dissolved in DMF (50 mL), and the BnBr (0.40 mL, 4.2 mmol) and cesium fluoride (CsF, 611 mg, 4.0 mmol) were added. The reaction mixture was stirred at room temperature overnight under an atmosphere of argon. TLC analysis showed complete conversion of starting material **20** to compound **21** (petroleum ether/ethyl acetate = 2/1, R_f = 0.80). The mixture was concentrated *in vacuo*. The resulting residue was dissolved with CH_2Cl_2, and the mixture was washed with 1 M HCl and brine. The organic phase was dried (Na_2SO_4), filtered, and the filtrate was concentrated *in vacuo*. The resulting residue was purified by silica gel column chromatography (petroleum ether/ethyl acetate = 4:1) to give compound **21** (1.6 g, 87%) as a colorless oil. ^{1}H NMR (400 MHz, $CDCl_3$) δ 7.45 – 7.20 (m, 22H, ArH), 7.09 (d, *J* = 7.7 Hz, 2H, ArH), 5.48 (d, *J* = 1.8 Hz, 1H, H-1-Hep), 5.23 – 5.06 (m, 2H, C*H₂*-Bn), 4.86 (d, *J* = 10.9 Hz, 1H, C*H*H-Bn), 4.79 (d, *J* = 11.5 Hz, 1H, C*H*H-Bn), 4.65 (d, *J* = 11.5 Hz, 1H, C*H*H-Bn), 4.58 (d, *J* = 10.9 Hz, 1H, C*H*H-Bn), 4.47 – 4.45 (m, 2H, C*H₂*-Bn), 4.32 (d, *J* = 8.6 Hz, 1H), 4.30 – 4.24 (m, 2H), 4.01 (t, *J* = 9.5 Hz, 1H), 3.93 – 3.86 (m, 2H), 3.63 (dd, *J* = 10.2, 5.0 Hz, 1H, H-7a-Hep), 3.54 (dd, *J* = 10.1, 7.5 Hz, 1H, H-7b-Hep), 3.38 (s, 1H, OH), 2.67 (dd, *J* = 15.3, 7.9 Hz, 1H, H-2a-3Hb), 2.49 (dd, *J* = 15.2, 4.8 Hz, 1H, H-2b-3Hb), 2.33 (s, 3H, C*H₃*-STol), 1.24 (d, *J* = 6.2 Hz, 3H, C*H₃*-3Hb); ^{13}C NMR (101 MHz, $CDCl_3$) δ 172.38 (*C*=O-COOBn), 138.56, 138.08, 137.80, 135.83, 132.58, 130.34, 129.90, 128.59, 128.39, 128.19, 127.92, 127.54, 88.87 (C-1-Hep), 80.69, 78.29, 74.72, 74.31, 73.41, 73.29, 73.08, 71.99, 71.63, 69.88, 66.73, 42.51, 21.23, 20.41. HRMS (ESI): m/z calcd for $C_{46}H_{50}NaO_8S^+$ $[M+Na]^+$ 785.3119, found 785.3119.

***p*-Tolyl 2-*O*-fluorenylmethoxycarbonyl-3,4,7-tri-*O*-benzyl-6-*O*-[benzyl 3(*S*)-butanoate]-1-thio-D-*glycero*-*α*-D-*manno*-heptopyranoside (22)**

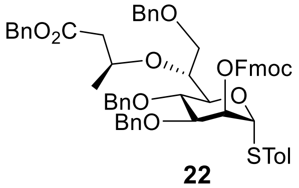

22

To solution of a mixture of **21** (500 mg, 0.66 mmol) in a mixed solvent of pyridine (2 mL) and CH_2Cl_2 (12 mL) was added FmocCl (204 mg, 0.79 mmol) at 0 ºC under an atmosphere of argon. The mixture was stirred at room temperature for 8 h. TLC analysis showed complete conversion of starting material **21** to compound **22** (petroleum ether/ethyl acetate = 4/1, R_f = 0.75). The reaction was quenched with MeOH and concentrated *in vacuo*. The resulting residue was dissolved with CH_2Cl_2, and the mixture was washed with 1 M HCl, saturated aqueous $NaHCO_3$ and brine. The organic phase was dried (Na_2SO_4), and filtered. The filtrate was concentrated *in vacuo* and resulting residue was purified by silica gel column chromatography (petroleum ether/ethyl acetate = 10/1) to give compound **22** (595 mg, 91%) as a colorless oil. ^{1}H NMR (600 MHz, $CDCl_3$) δ 7.78 (dd, *J* = 7.6, 3.1 Hz, 2H, ArH), 7.62 (t, *J* = 8.1 Hz, 2H, ArH), 7.46 – 7.25 (m, 26H, ArH), 7.12 (d, *J* = 8.2 Hz, 2H, ArH), 5.48 (s, 1H, H-1-Hep), 5.44 (dd, *J* = 3.1, 1.8 Hz, 1H, H-2-Hep), 5.06 (d, *J* = 12.3 Hz, 1H, C*H*H-Bn), 5.01 (d, *J* = 12.4 Hz, 1H, C*H*H-Bn), 4.95 (d, *J* = 11.0 Hz, 1H, C*H*H-Bn), 4.79 (d, *J* = 11.4 Hz, 1H, C*H*H-Bn), 4.71 (d, *J* = 11.0 Hz, 1H, C*H*H-Bn), 4.64 (d, *J* = 11.3 Hz, 1H, C*H*H-Bn), 4.53 (s, 2H, C*H₂*-Bn), 4.49 – 4.41 (m, 2H, C*H*H-Fmoc, H-5-Hep), 4.33 (dd, *J* = 10.4, 8.0 Hz, 1H, C*H*H-Fmoc), 4.28 – 4.19 (m, 2H, C*H*-Fmoc, H-3-3Hb), 4.14 (t, *J* = 9.6 Hz, 1H, H-4-Hep), 4.04 – 4.01 (m, 1H, H-6-Hep), 4.00 (dd, *J* = 9.2, 3.0 Hz, 1H, H-3-Hep), 3.77 (dd, *J* = 10.1, 5.2 Hz, 1H, H-7a-Hep), 3.65 (dd, *J* = 10.2, 6.9 Hz, 1H, H-7b-Hep), 2.84 (dd, *J* = 15.1, 5.8 Hz, 1H, H-2a-3Hb), 2.52 (dd, *J* = 15.1, 7.1 Hz, 1H, H-2b-3Hb), 2.34 (s, 3H, CH_3-STol), 1.30 (d, *J* = 6.1 Hz, 3H, CH_3-3Hb); ^{13}C NMR (151 MHz, $CDCl_3$) δ 171.26 (*C*=O-COOBn), 154.78 (*C*=O-Fmoc), 143.62, 143.27, 141.41, 141.33, 138.61, 138.57, 138.28, 137.77, 136.11, 133.11, 129.95, 129.83, 128.51, 128.49, 128.43, 128.41, 128.38, 128.21, 128.16, 128.13, 128.10, 128.00, 127.94, 127.89, 127.69, 127.64, 127.56, 127.25, 125.42, 125.25, 120.13, 120.10, 86.50 (C-1-Hep), 77.47, 75.00, 74.72, 74.39, 73.84, 73.44, 72.75, 72.06, 71.29, 70.24, 66.20, 46.73, 42.31, 21.24, 20.67. HRMS (ESI): m/z calcd for $C_{61}H_{60}NaO_{10}S^+$ $[M+Na]^+$ 1007.3799, found 1007.3799.

***N*-phenyl-trifluoroacetimidoyl 2-*O*-fluorenylmethoxycarbonyl-3,4,7-tri-*O*-benzyl-6-*O*-[benzyl 3(*S*)-butanoate]-D-*glycero*-*α/β*-D-*manno*-heptopyranoside (9)**

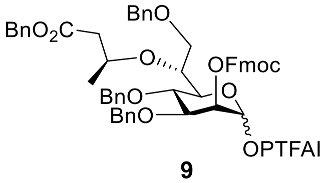

9

To a solution of compound **22** (180 mg, 0.18 mmol) in wet CH_2Cl_2 (5 mL), NIS (82 mg, 0.36 mmol) and TfOH (3 μL, 0.04 mmol) were added. The reaction mixture was stirred for 0.5 h at 0 ºC. TLC analysis showed complete conversion of starting material **9** to a hemiacetal intermediate (petroleum ether/ethyl acetate = 4:1, R_f =0.25). The reaction was quenched with Et_3N and concentrated *in vacuo*. The resulting residue was purified by silica gel column chromatography (petroleum ether/ethyl acetate = 4/1) to give hemiacetal intermediate which was directly used for next step.

The resulting oil was dissolved in anhydrous CH_2Cl_2 (5 mL), and *N*-phenyl trifluoroacetimidoyl chloride (44 μL, 0.27 mmol) and Cs_2CO_3 (89 mg, 0.27 mmol) were added. After being stirred for 2 h at room temperature under an atmosphere of argon, TLC analysis showed complete conversion of hemiacetal intermediate to compound **9** (petroleum ether/ethyl acetate = 4:1, R_f = 0.80). The reaction mixture was filtered through celite, and concentrated *in vacuo*. The resulting residue was purified by silica gel column chromatography (petroleum ether/ethyl acetate = 12:1) to give compound **9** (164 mg, two steps 85%) as a colorless oil that was immediately used for the next glycosylation.

Table S6. The *O*-6 alkylation of Hep **11** using **12a-b**[a]

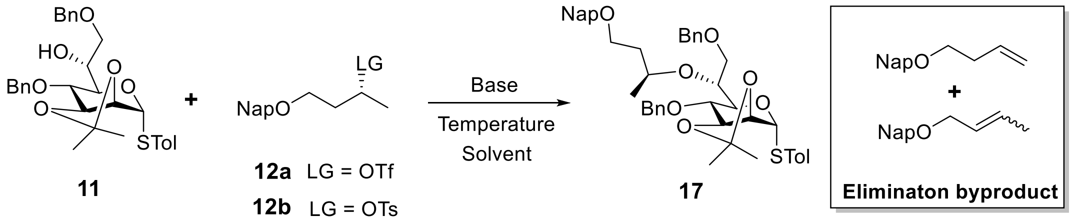

Entry	Substrate	Base	T (ºC)	Solvent	Results[b]
S1	**12a** (1.5 eq.)	NaH (1.5 eq.)	0 to r.t.	DMF	N.D., **12a** was eliminated[c]
S2	**12b** (2 eq.)	NaH (1.5 eq.)	0 to r.t.	DMF	N.D., **12b** was partly eliminated
S3	**12b** (4 eq.)	NaH (1.5 eq.)	0 to r.t.	DMF	N.D., **12b** was partly eliminated
S4	**12b** (4 eq.)	NaH (1.5 eq.)	0 to 80	DMF	Trace, **12b** was partly eliminated
S5	**12b** (4 eq.)	LiHMDS[d] (2.0 eq.)	0 to r.t.	THF	N.D., **12b** was partly eliminated
S6	**12b** (4 eq.)	KHMDS[d] (2.0 eq.)	0 to r.t.	THF	N.D., **12b** was partly eliminated

[a]General Procedure: To a solution of Hep in anhydrous solvent was added base at 0 ºC under an atmosphere of argon. The reaction mixture was vigorously stirred at 0 ºC for 20 min. Next, a solution of **12a/b** disolved in anhydrous solvent was added to the above reaction mixture dropwise using syringe at 0 ºC. Then, the reaction was stirred at room temperature. [b]N.D. = Not detected of product. [c]The elimination byproducts were isolated and identified as a mixture of olefins (See Figure S4). [d]LiHMDS (lithium bis(trimethylsilyl)amide, 1 M in THF); KHMDS (potassium bis(trimethylsilyl)amide, 1 M in THF).

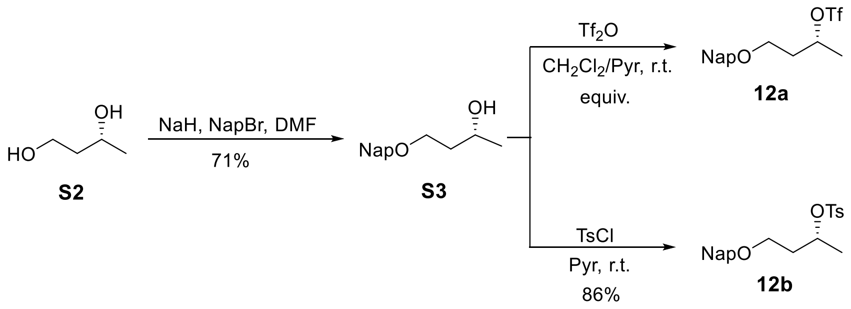

Scheme S2. Synthesis of **12a** and **12b**.

(*R*)-4-*O*-(naphthylmethyl)-butan-2-ol (S3)

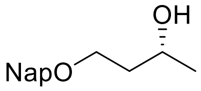

S3

A solution of commercial (*R*)-butane-1,3-diol **S2** (2 g, 22 mmol) in anhydrous DMF (20 mL) was added NapBr (4.9 g, 22 mmol) and NaH (60% dispersion in mineral oil, 0.89 g, 22 mmol) at -20 °C under an atmosphere of argon. The mixture was slowly warmed to room temperature and stirred for 1 h. TLC analysis showed conversion of starting material **S2** to a major product **S3** (petroleum ether/ethyl acetate = 4:1, R_f = 0.30). The reaction mixture was concentrated *in vacuo*. The resulting residue was dissolved with ethyl acetate, and the mixture was washed with 1 M HCl, saturated aqueous $NaHCO_3$ and brine. The organic phase was dried (Na_2SO_4), and filtered. The filtrate was concentrated *in vacuo* and resulting residue was purified by silica gel column chromatography (petroleum ether/ethyl acetate = 4/1) to give compound **S3** (3.6 g, 71%) as light yellow oil. ^{1}H NMR (600 MHz, $CDCl_3$) δ 7.84 (dd, J = 7.3, 4.8 Hz, 3H, ArH), 7.77 (s, 1H, ArH), 7.54 – 7.38 (m, 3H, ArH), 4.69 (s, 2H, CH_2-Nap), 4.10 – 3.95 (m, 1H, H-2), 3.79 – 3.71 (m, 1H, H-4a), 3.71 – 3.62 (m, 1H, H-4b), 2.75 (s, 1H, OH), 1.93 – 1.66 (m, 2H, H-3), 1.22 (d, J = 6.2 Hz, 3H, CH_3); ^{13}C NMR (151 MHz, $CDCl_3$) δ 135.55, 133.37, 133.13, 128.41, 127.98, 127.82, 126.58, 126.27, 126.05, 125.74, 73.51, 69.25, 67.67, 38.31, 23.50. HRMS (ESI): m/z calcd for $C_{15}H_{18}NaO_2^+$ [M+Na]$^+$ 253.1199, found 253.1198.

(*R*)-4-*O*-(naphthylmethyl)-butan-2-yl trifluoromethanesulfonate (12a)

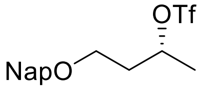

12a

To a solution of **S3** (100 mg, 0.43 mmol) in a mixed solvent of CH_2Cl_2/pyridine (v/v, 2/1, 6 mL) was added Tf_2O (90 μL, 0.52 mmol) at -20 °C under an atmosphere of argon. After being stirred at this temperature for 1 h, TLC analysis showed complete conversion of starting material **S3** to product **12a** (petroleum ether/ethyl acetate = 8/1, R_f = 0.80). The reaction was quenched by adding H_2O. The organic phase was washed with 1 M HCl, saturated aqueous $NaHCO_3$ and brine. The organic phase was dried (Na_2SO_4), and filtered. The filtrate was concentrated *in vacuo* to give the compound **12a**, which was directly used for the next step without further purification.

(*R*)-4-*O*-(naphthylmethyl)-butan-2-yl 4-methylbenzenesulfonate (12b)

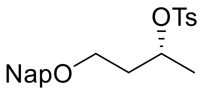

12b

To a solution of **S3** (0.5 g, 2.2 mmol) in pyridine (10 mL) was added *p*-toluenesulfonyl chloride (0.83 g, 4.3 mmol) at 0 ºC. The reaction was stirred at room temperature overnight. TLC analysis showed complete conversion of starting material **S3** to product **12b** (petroleum ether/ethyl acetate = 4/1, R_f = 0.75). The reaction was quenched with MeOH, and the reaction mixture was concentrated *in vacuo*. The resulting residue was dissolved with ethyl acetate, and the mixture was washed with 1 M HCl, saturated aqueous $NaHCO_3$ and brine. The organic phase was dried (Na_2SO_4), and filtered. The filtrate was concentrated *in vacuo* and resulting residue was purified by silica gel column chromatography (petroleum ether/ethyl acetate = 6:1) to give compound **12b** (0.72 g, 86%) as a colorless oil. ^{1}H NMR (500 MHz, $CDCl_3$) δ 7.91 – 7.76 (m, 5H, ArH), 7.76 – 7.68 (m, 1H, ArH), 7.53 – 7.44 (m, 2H, ArH), 7.40 (dd, J = 8.4, 1.8 Hz, 1H, ArH), 7.30 – 7.18 (m, 2H, ArH), 4.98 – 4.78 (m, 1H, H-2), 4.55 – 4.34 (m, 2H, C*H*$_2$-Nap), 3.52 – 3.46 (m, 1H, H-4a), 3.44 – 3.35 (m, 1H, H-4b), 2.36 (s, 3H, C*H*$_3$-Ts), 1.97 – 1.91 (m, 1H, H-3a), 1.88 – 1.77 (m, 1H, H-3b), 1.32 (d, J = 6.3 Hz, 3H, C*H*$_3$); ^{13}C NMR (126 MHz, $CDCl_3$) δ 144.56, 135.80, 134.50, 133.38, 133.08, 129.83, 128.25, 127.98, 127.86, 127.82, 126.35, 126.24, 125.98, 125.73, 77.96, 73.13, 65.97, 36.93, 21.68, 21.35. HRMS (ESI): m/z calcd for $C_{22}H_{24}NaO_4S^+$ $[M+Na]^+$ 407.1288, found 407.1286.

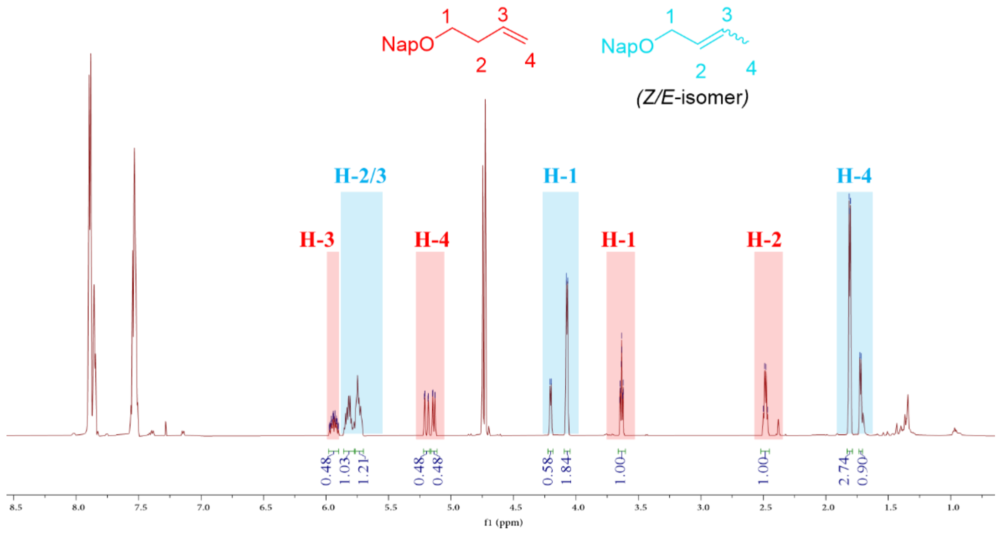

Figure S4. The ^{1}H NMR analysis of olefins byproduct.

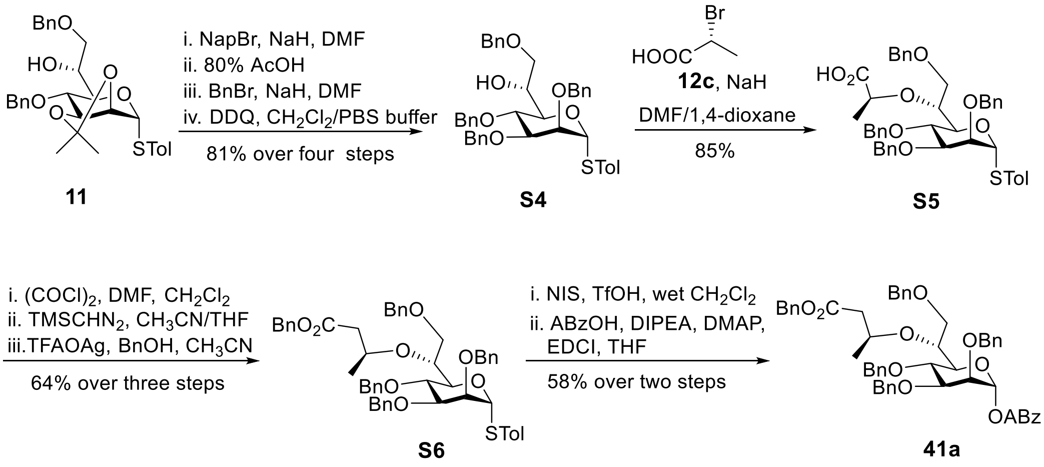

Scheme S3. Synthesis of Hep building block **41a**

***p*-Tolyl 2,3,4,7-tetra-*O*-benzyl-1-thio-D-*glycero*-*α*-D-*manno*-heptopyranoside (S4)**

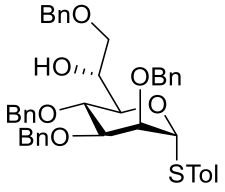

S4

A solution of compound **11** (1.2 g, 2.2 mmol) in anhydrous DMF (20 mL) was added NapBr (593 mg, 2.7 mmol) and NaH (60% dispersion in mineral oil, 107 mg, 2.7 mmol) at 0 ºC. The mixture was stirred for 4 h at room temperature under an atmosphere of argon. TLC analysis showed complete conversion of starting material **11** to a 6-ONap intermediate (petroleum ether/ethyl acetate = 6/1, R_f = 0.75). The reaction was quenched with MeOH (5 mL), and the reaction mixture was concentrated *in vacuo*. The resulting residue was dissolved with ethyl acetate, and the mixture was washed with 1 M HCl, saturated aqueous $NaHCO_3$ and brine. The organic phase was dried (Na_2SO_4), and filtered. The filtrate was concentrated *in vacuo* and resulting residue was used for the next step.

The above 6-ONap intermediate was treated with 80% AcOH in water (50 mL) and the mixture was heated to 80 ºC for 2 h. TLC analysis showed complete conversion of starting material to 2,3-diol intermediate (petroleum ether/ethyl acetate = 4/1, R_f = 0.25). The reaction mixture was cooled to room temperature and concentrated under reduced pressure. The reaction mixture was cooled to room temperature and concentrated under reduced pressure to give crude product. The resulting residue was purified by silica gel column chromatography (petroleum ether/ethyl acetate = 4/1 to 2/1) to give 2,3-diol intermediate (1.4 g) as a colorless oil.

A solution of 2,3-diol intermediate (1.4 g, 2.2 mmol) in anhydrous DMF (50 mL) was added BnBr (0.65 mL, 5.5 mmol) and NaH (60% dispersion in mineral oil, 220 mg, 5.5 mmol) at 0 °C. The mixture was stirred for 2 h at room temperature under an atmosphere of argon. TLC analysis showed complete conversion of starting material to a major 2,3-OBn intermediate (petroleum ether/ethyl acetate = 10/1, R_f = 0.75). The reaction was quenched with MeOH (5 mL), and the reaction mixture was concentrated *in vacuo*. The resulting residue was dissolved with ethyl acetate, and the mixture was washed with 1 M HCl, saturated aqueous $NaHCO_3$ and brine. The organic phase was dried (Na_2SO_4), and filtered. The filtrate was concentrated *in vacuo* and resulting residue was purified by silica gel column chromatography (petroleum ether/ethyl acetate = 25/1 to 20/1) to give 2,3-OBn intermediate (1.5 g) as a colorless oil.

To solution of above 2,3-OBn intermediate (1.5 g, 1.8 mmol) in a mixed solvent of CH_2Cl_2/0.1 M PBS buffer (v/v, 10/1, 55 mL) was added DDQ (0.6 g, 2.8 mmol) at 0 °C. After being stirred at room temperature for 4 h, the reaction was diluted with ethyl acetate, and washed with saturated aqueous $NaHCO_3$ and brine. The organic phase was dried (Na_2SO_4), and filtered. The filtrate was concentrated under reduced pressure to give crude product. The crude product was purified by silica gel column chromatography (petroleum ether/ethyl acetate = 4/1 to 2/1) to give compound **S4** (1.18 g, 81% over four steps) as a colorless oil. ^{1}H NMR (600 MHz, $CDCl_3$) δ 7.41 – 7.20 (m, 22H, ArH), 7.09 (d, J = 8.0 Hz, 2H, ArH), 5.46 (d, J = 1.3 Hz, 1H, H-1), 4.94 (d, J = 10.8 Hz, 1H, C*H*H-Bn), 4.69 (d, J = 12.3 Hz, 1H, C*H*H-Bn), 4.66 – 4.59 (m, 4H, 4 × C*H*H-Bn), 4.51 (d, J = 12.1 Hz, 1H, C*H*H-Bn), 4.45 (d, J = 11.9 Hz, 1H, C*H*H-Bn), 4.20 (dd, J = 9.4, 4.1 Hz, 1H, H-3), 4.19 – 4.13 (m, 1H, H-6), 4.10 (t, J = 9.0 Hz, 1H, H-4), 3.96 (d, J = 3.0 Hz, 1H, H-2), 3.89 (dd, J = 8.8, 3.5 Hz, 1H, H-5), 3.59 (dd, J = 10.0, 3.6 Hz, 1H, H-7a), 3.52 (dd, J = 10.3, 7.0 Hz, 1H, H-7b), 2.33 (s, 3H, C*H*$_3$-STol); ^{13}C NMR (151 MHz, $CDCl_3$) δ 138.32, 138.28, 138.11, 138.00, 137.94, 132.50, 130.28, 129.97, 128.59, 128.54, 128.45, 128.15, 128.04, 128.02, 127.92, 127.85, 127.67, 86.15 (C-1), 80.33, 76.21, 75.84, 74.83, 73.45, 72.92, 72.24, 72.21, 71.88, 71.26, 21.24. HRMS (ESI): m/z calcd for $C_{42}H_{44}NaO_6S^+$ [M+Na]$^+$ 699.2751, found 699.2755.

***p*-Tolyl 2,3,4,7-tetra-*O*-benzyl-6-[2(*S*)-lactic acid]-1-thio-D-*glycero*-*α*-D-*manno*-heptopyranoside (S5)**

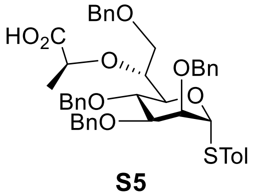

S5

To a solution of compound **S4** (500 mg, 0.74 mmol) in a mixed slovent of anhydrous DMF/1,4-dioxane (v/v, 1/1, 10 mL) was slowly added NaH (60% in mineral oil, 0.3 g, 7.4 mmol) at 0 °C under an atmosphere of argon. The reaction was vigorously stirred at 0 °C for 20 min. Then, the *(R)*-2-bromopropionic acid (0.33 mL, 3.7 mmol) was added dropwise using syringe. After being stirred at room temperature for 8 h. TLC analysis showed complete conversion of starting material **S4** to compound **S5** (petroleum ether/ethyl acetate = 1/1, R_f= 0.20). The reaction was carefully quenched with H_2O at 0 °C and diluted with CH_2Cl_2. The aqueous phase was re-extracted twice with CH_2Cl_2. The combined organic phase was acidified to pH 3 with 2.5 M aqueous HCl, washed with brine, dried (Na_2SO_4), and filtered. The filtrate was concentrated under reduced pressure and resulting residue was purified by silica gel column chromatography (petroleum ether/ethyl acetate = 20/1 to 4/1) to give compound **S5** (470 mg, 85%) as a light yellow oil. ^{1}H NMR (600 MHz, $CDCl_3$) δ 7.39 – 7.27 (m, 16H, ArH), 7.23 – 7.16 (m, 6H, ArH), 7.11 (d, *J* = 8.2 Hz, 2H, ArH), 5.41 (d, *J* = 2.1 Hz, 1H, H-1-Hep), 5.02 (d, *J* = 11.6 Hz, 1H, C*H*H-Bn), 4.71 (d, *J* = 12.5 Hz, 1H, C*H*H-Bn), 4.60 (d, *J* = 12.5 Hz, 1H, C*H*H-Bn), 4.59 – 4.51 (m, 3H, 3 × C*H*H-Bn), 4.46 – 4.33 (m, 2H, H-2-lactic acid, C*H*H-Bn), 4.26 (t, *J* = 9.3 Hz, 1H, H-4-Hep), 4.15 (dd, *J* = 9.2, 1.7 Hz, 1H, H-5-Hep), 3.96 (d, *J* = 2.5 Hz, 1H, H-2-Hep), 3.88 – 3.79 (m, 2H, H-3-Hep, H-6-Hep), 3.62 (dd, *J* = 10.5, 2.4 Hz, 1H, H-7a-Hep), 3.55 (dd, *J* = 10.6, 8.4 Hz, 1H, H-7b-Hep), 2.34 (s, 3H, C*H$_3$*-STol), 1.49 (d, *J* = 7.0 Hz, 3H, C*H$_3$*-lactic acid); ^{13}C NMR (151 MHz, $CDCl_3$) δ 174.74 (*C*=O-COOH), 138.23, 138.19, 137.80, 137.72, 137.49, 131.78, 130.21, 128.65, 128.63, 128.59, 128.55, 128.35, 128.12, 128.10, 128.02, 127.94, 127.85, 127.83, 86.04 (C-1-Hep), 82.79 (C-3-Hep), 80.12 (C-6-Hep), 77.6 (C-2-lactic acid), 75.11 (C-2-Hep), 74.78, 73.58, 73.53 (C-4-Hep), 72.68 (C-5-Hep), 72.44 (C-7-Hep), 72.03, 71.97, 21.26 (*C*H$_3$-STol), 19.25 (*C*H$_3$-lactic acid). HRMS (ESI): m/z calcd for $C_{45}H_{48}NaO_8S^+$ [M+Na]$^+$ 771.2962, found 771.2961.

***p*-Tolyl 2,3,4,7-tetra-*O*-benzyl-6-*O*-[benzyl 3(*S*)-butanoate]-1-thio-D-*glycero*-*α*-D-*manno*-heptopyranoside (S6)**

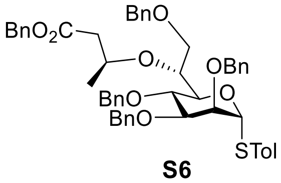

S6

To a solution of acid **S5** (400 mg, 0.53 mmol) in anhydrous CH_2Cl_2 (10 mL) was added a drop of anhydrous DMF and $(COCl)_2$ (90 μL, 1.1 mmol) at 0 °C under an atmosphere of argon. The reaction was stirred at room temperature for 40 min. The reaction progress was monitored by taking a drop of reaction mixture and quenching with methanol

in a centrifuge tube, converting the acid chloride intermediate to its methyl ester derivative. (methyl ester derivative: petroleum ether/ethyl acetate = 4/1, R_f= 0.62). The reaction mixture was concentrated *in vacuo* to give crude acid chloride product as a yellow oil.

To a solution of $TMSCHN_2$ (2 M in hexane, 1.3 mL, 2.7 mmol) in anhydrous THF (5 mL) was slowly added the above acid chloride dissolved in a mixed solvent of anhydrous THF/CH_3CN (v/v, 1/1, 10 mL) at 0 °C under an atmosphere of argon. After being stirred at room temperature for 0.5 h, TLC analysis showed complete conversion to diazo ketone intermediate (petroleum ether/ethyl acetate = 2/1, R_f= 0.60). The reaction was quenched with 0.5 M aqueous lemon acid at 0 °C until bubbling subsided and diluted with CH_2Cl_2. The aqueous layer was separated and re-extracted twice with CH_2Cl_2, and the combined organic layer was washed with saturated aqueous $NaHCO_3$ and brine. The organic phase was dried (Na_2SO_4), and filtered. The filtrate was concentrated *in vacuo* to give the crude diazo ketone intermediate as a yellow oil for the next step.

To a solution of above diazo ketone and anhydrous BnOH (0.55 mL, 5.3 mmol) in a mixed solvent of anhydrous THF/CH_3CN (v/v, 1/1, 50 mL) was added AgOTFA (24 mg, 0.1 mmol, disolved in 0.2 mL anhydrous Et_3N) at -20 °C under an atmosphere of argon. The reaction was gradually warmed to room temperature over 2 h. TLC analysis showed complete conversion of diazo ketone intermediate to a major product **S6** (petroleum ether/ethyl acetate = 5/1, R_f= 0.80). The reaction was quenched with 0.5 mL of aqueous 1 M HCl and diluted with CH_2Cl_2. The aqueous layer was separated and re-extracted twice with CH_2Cl_2, and the combined organic layer was washed with saturated aqueous $NaHCO_3$ and brine. The organic phase was dried (Na_2SO_4), and filtered. The filtrate was concentrated *in vacuo* to give the crude product. The crude product was purified by silica gel column chromatography (petroleum ether/ethyl acetate = 25/1 to 20/1) to give compound **S6** (295 mg, 64% over three steps) as a colorless oil. ^{1}H NMR (600 MHz, $CDCl_3$) δ 7.39 – 7.21 (m, 27H, ArH), 7.07 (d, *J* = 8.3 Hz, 2H, ArH), 5.43 (d, *J* = 2.0 Hz, 1H, H-1), 5.07 – 4.97 (m, 2H, C*H₂*-Bn), 4.92 (d, *J* = 10.9 Hz, 1H, CH*H*-Bn), 4.69 – 4.56 (m, 5H, 5 × CH*H*-Bn), 4.52 – 4.42 (m, 2H,), 4.29 (dd, *J* = 9.8, 1.3 Hz, 1H, H-5-Hep), 4.21 – 4.17 (m, 1H, H-3-3Hb), 4.10 (t, *J* = 9.2 Hz, 1H, H-4-Hep), 4.01 – 3.93 (m, 2H, H-2-Hep, H-6-Hep), 3.86 (dd, *J* = 9.1, 3.0 Hz, 1H, H-3-Hep), 3.71 (dd, *J* = 10.4, 4.5 Hz, 1H, H-7b-Hep), 3.60 (dd, *J* = 10.3, 7.1 Hz, 1H, H-7a-Hep), 2.81 (dd, *J* = 15.1, 5.6 Hz, 1H, H-2b-3Hb), 2.44 (dd, *J* = 15.1, 7.6 Hz, 1H, H-2a-3Hb), 2.32 (s, 3H, C*H₃*-STol), 1.25 (d, *J* = 6.2 Hz, 3H, C*H₃*-3Hb); ^{13}C NMR (151 MHz, $CDCl_3$) δ 171.36 (*C*=O-COOBn), 138.81, 138.71, 138.34, 138.16, 137.82, 136.22, 132.72, 130.76, 129.87, 128.62, 128.57, 128.53, 128.48, 128.45, 128.41, 128.38, 128.28, 128.26, 128.13, 128.02, 128.00, 127.95, 127.91, 127.81, 127.76, 127.61, 127.56, 127.48, 86.19 (C-1-Hep), 80.56, 77.81, 76.35, 75.05, 74.82, 74.00, 73.35, 72.68, 72.19,

71.76, 71.43, 66.16, 42.35, 21.24, 20.66. HRMS (ESI): m/z calcd for $C_{53}H_{56}NaO_8S^+$ $[M+Na]^+$ 875.3588, found 875.3582.

2,3,4,7-tetra-*O*-benzyl-6-*O*-[benzyl 3(*S*)-butanoate]-D-*glycero*-*α*-D-*manno*-heptopyranosyl *ortho*-hexynylbenzoate (41a)

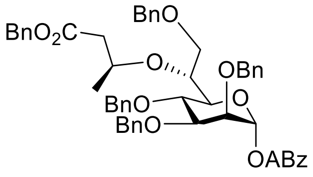

41a

To a solution of compound **S6** (220 mg, 0.26 mmol) in wet CH_2Cl_2 (5 mL) were added NIS (87 mg, 0.38 mmol) and TfOH (4.6 μL, 0.05 mmol) at 0 °C. The reaction mixture was stirred for 0.5 h at 0 °C. TLC analysis showed complete conversion of starting material **S6** to a hemiacetal intermediate (petroleum ether/ethyl acetate = 6/1, R_f =0.25). The reaction was quenched with Et_3N. The organic phase was washed with Na_2SO_3 aqueous, brine, dried (Na_2SO_4), and filtered. The filtrate was concentrated under reduced pressure, and resulting residue was purified by silica gel column chromatography (petroleum ether/ethyl acetate = 4/1) to give hemiacetal intermediate (175 mg) which was used for next step.

A solution of above hemiacetal intermediate (175 mg, 0.23 mmol), *ortho*-hexynylbenzoic acid (71 mg, 0.35 mmol), DMAP (43 mg, 0.35 mmol), EDCI (45 mg, 0.23 mmol) and DIPEA (123 μL, 0.70 mmol) in anhydrous CH_2Cl_2 (5 mL) was stirred at room temperature for 2 h. TLC analysis showed complete conversion of starting material to **41a** (petroleum ether/ethyl acetate =20/1, R_f =0.25) and β-isomer (petroleum ether/ethyl acetate = 6/1, R_f =0.15). The mixture was washed with saturated $NaHCO_3$ solution, H_2O, brine, dried (Na_2SO_4), and filtered. The filtrate was concentrated under reduced pressure, and resulting residue was purified by silica gel column chromatography (petroleum ether/ethyl acetate = 30/1 to 25/1) to **41a** (125 mg, 58% over two steps) as a colorless oil. ^1H NMR (600 MHz, $CDCl_3$) δ 7.89 (d, J = 8.0 Hz, 1H, ArH), 7.55 (d, J = 7.8 Hz, 1H, ArH), 7.51 – 7.42 (m, 3H, ArH), 7.40 – 7.26 (m, 24H, ArH), 6.51 (d, J = 2.0 Hz, 1H, H-1), 5.03 (s, 2H, C*H2*-Bn), 4.99 (d, J = 10.7 Hz, 1H, C*H*H-Bn), 4.85 – 4.74 (m, 2H, C*H2*-Bn), 4.70 (d, J = 10.7 Hz, 1H, C*H*H-Bn), 4.62 (s, 2H, C*H2*-Bn), 4.55 – 4.47 (m, 2H, C*H2*-Bn), 4.30 – 4.19 (m, 2H, H-3-3Hb, H-4-Hep), 4.12 (d, J = 10.0 Hz, 1H, H-5-Hep), 4.09 (dd, J = 9.4, 3.1 Hz, 1H, H-3-Hep), 4.02 (t, J = 5.9 Hz, 1H, H-6-Hep), 3.91 (t, J = 2.7 Hz, 1H, H-2-Hep), 3.74 (dd, J = 10.4, 4.5 Hz, 1H, H-7a-Hep), 3.64 (dd, J = 10.4, 7.1 Hz, 1H, H-7b-Hep), 2.84 (dd, J = 15.3, 6.1 Hz, 1H, H-2a-3Hb), 2.51 – 2.40 (m, 3H,

H-2b-3Hb, CH_2-ABz), 1.62 – 1.54 (m, 2H, CH_2-ABz), 1.49 – 1.39 (m, 2H, CH_2-ABz), 1.28 (d, J = 6.2 Hz, 3H, CH_3-3Hb), 0.94 (t, J = 7.4 Hz, 3H, CH_3-ABz); ^{13}C NMR (151 MHz, $CDCl_3$) δ 171.19 (C=O-COOBn), 164.29 (C=O-ABz), 138.62, 138.59, 138.15, 138.06, 136.21, 134.89, 132.04, 130.84, 130.69, 128.55, 128.48, 128.43, 128.41, 128.35, 128.30, 128.24, 128.22, 128.08, 128.06, 128.04, 127.93, 127.76, 127.58, 127.51, 127.40, 127.22, 125.12, 96.96, 92.54 ($^1J_{C1\text{-}H1}$ = 175 Hz), 79.75, 79.51, 77.73, 75.57, 75.00, 74.26, 73.78, 73.26, 72.61, 72.36, 72.03, 71.15, 66.02, 42.24, 30.82, 22.10, 20.39, 19.64, 13.76. HRMS (ESI): m/z calcd for $C_{59}H_{62}NaO_{10}^+$ $[M+Na]^+$ 953.4235, found 953.4232.

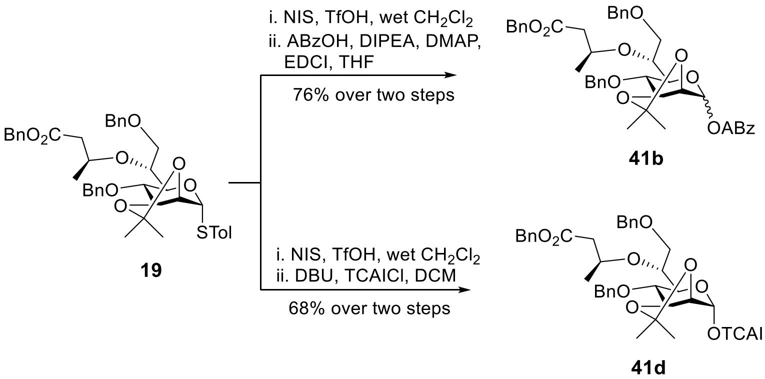

Scheme S4. Synthesis of Hep building blocks **41b** and **41d**

2,3-*O*-isopropylidene-4,7-di-*O*-benzyl-6-*O*-[benzyl 3(*S*)-butanoate]-D-*glycero*-*α/β*-D-*manno*-heptopyranosyl *ortho*-hexynylbenzoate (41b)

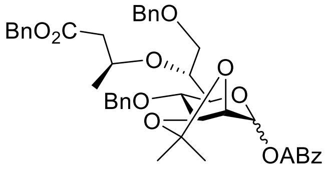

41b

To a solution of compound **19** (50 mg, 0.07 mmol) in wet CH_2Cl_2 (5 mL), NIS (23 mg, 0.11 mmol) and TfOH (1.3 μL, 0.02 mmol) were added at 0 ºC. The reaction mixture was stirred for 0.5 h at 0 ºC. TLC analysis showed complete conversion of starting material **19** to a hemiacetal intermediate (petroleum ether/ethyl acetate = 1/1, R_f =0.70). The reaction was quenched with Et_3N. The organic phase was washed with Na_2SO_3 aqueous, and brine, dried (Na_2SO_4), and filtered. The filtrate was concentrated under reduced pressure, and resulting residue was purified by silica gel column chromatography (petroleum ether/ethyl acetate = 3/1) to give hemiacetal intermediate

which was used for next step.

A solution of above hemiacetal intermediate (40 mg, 0.07 mmol), *ortho*-hexynylbenzoic acid (20 mg, 0.1 mmol), DMAP (12 mg, 0.1 mmol), EDCI (17 mg, 0.1 mmol) and DIPEA (21 μL, 0.1 mmol) in anhydrous CH_2Cl_2 (4 mL) was stirred at room temperature for 2 h. TLC analysis showed complete conversion of starting material to **41b** (petroleum ether/ethyl acetate = 2/1, R_f =0.80). The mixture was washed with saturated $NaHCO_3$ solution, H_2O, brine, dried (Na_2SO_4), and filtered. The filtrate was concentrated under reduced pressure, and resulting residue was purified by silica gel column chromatography (petroleum ether/ethyl acetate = 8/1 to 6/1) to **41b** (42 mg, 76% over two steps, isomer mixture α/β = 5/1) as a colorless oil. ^{1}H NMR (600 MHz, $CDCl_3$) δ 8.26 (d, J = 6.9 Hz, 0.2H, ArH), 7.92 (dd, J = 8.0, 1.5 Hz, 1H, ArH), 7.61 – 7.59 (m, J = 7.5, 1.4 Hz, 0.2 H, ArH), 7.50 (dd, J = 7.8, 1.4 Hz, 1H, ArH), 7.48 – 7.41 (m, 0.2H, ArH), 7.41 – 4.39 (m, J = 7.6, 1.5 Hz, 1H, ArH), 7.36 – 7.22(m, 16H, ArH), 6.57 (s, 1H, H-1α-Hep), 6.25 (s, 0.2H, H-1β-Hep), 5.04 (s, 2H, C*H*$_2$-Bn), 4.88 (d, J = 11.2 Hz, 1H, C*H*H-Bn), 4.62 (d, J = 11.2 Hz, 1H, C*H*H-Bn), 4.49 – 4.38 (m, 3H, 3 × C*H*H-Bn), 4.27 – 4.23 (m, 1H, C*H*H-Bn), 4.19 – 4.15 (m, J = 6.3 Hz, 1H), 4.03 (dd, J = 9.8, 1.9 Hz, 1H), 3.99 – 3.97 (m, 1H), 3.86 (dd, J = 9.9, 6.9 Hz, 1H), 3.63 (dd, J = 10.2, 4.9 Hz, 1H), 3.56 (dd, J = 10.2, 6.8 Hz, 1H), 2.69 (dd, J = 15.1, 6.2 Hz, 1H), 2.54 (t, J = 7.6 Hz, 0.4 H), 2.46 (t, J = 7.2 Hz, 2H), 2.40 (dd, J = 15.1, 6.9 Hz, 1H), 1.75 – 1.67 (m, 0.4H), 1.63 – 1.55 (m, 2H), 1.55 (s, 3H), 1.49 – 1.39 (m, 2.4H), 1.39 (s, 3H), 1.23 (d, J = 6.2 Hz, 3H), 0.96 (t, J = 7.4 Hz, 0.6H), 0.92 (t, J = 7.3 Hz, 3H). ^{13}C NMR (151 MHz, $CDCl_3$) δ 171.14 (*C*=O-COOBn), 164.13 (*C*=O-ABz), 138.56, 138.39, 136.20, 134.84, 132.08, 130.87, 128.59, 128.35, 128.28, 128.17, 128.13, 127.65, 127.56, 127.48, 127.32, 125.05, 109.96, 96.66, 92.18, 79.68, 78.88, 75.31, 74.88, 73.29, 73.18, 72.81, 72.38, 71.00, 66.18, 42.41, 30.87, 27.92, 26.56, 22.19, 20.59, 19.70, 13.77. HRMS (ESI): m/z calcd for $C_{48}H_{54}NaO_{10}^+$ $[M+Na]^+$ 813.3609, found 813.3606.

2,2,2-trichloroacetimidoyl 2,3-*O*-isopropylidene-4,7-di-*O*-benzyl-6-*O*-[benzyl 3(*S*)-butanoate]-D-*glycero*-*α*-D-*manno*-heptopyranoside (41d)

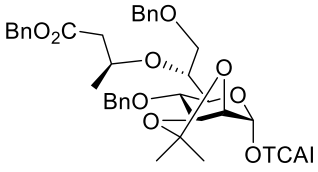

41d

To a solution of compound **19** (100 mg, 0.14 mmol) in wet CH_2Cl_2 (5 mL), NIS (47 mg, 0.21 mmol) and TfOH (2.5 μL, 0.04 mmol) were added at 0 ºC. The reaction mixture was stirred for 0.5 h at 0 ºC. TLC analysis showed

complete conversion of starting material **19** to a hemiacetal intermediate (petroleum ether/ethyl acetate = 1/1, R_f =0.70). The reaction was quenched with Et_3N. The organic phase was washed with Na_2SO_3 aqueous, brine, dried (Na_2SO_4), and filtered. The filtrate was concentrated under reduced pressure, and resulting residue was purified by silica gel column chromatography (petroleum ether/ethyl acetate = 3/1) to give hemiacetal intermediate which was used for next step.

A solution of above hemiacetal intermediate (82 mg, 0.14 mmol), DBU (30 μL, 0.20 mmol), and CCl_3CN (27 μL, 0.27 mmol) in anhydrous CH_2Cl_2 (5 mL) was stirred at room temperature for 2 h. TLC analysis showed complete conversion of starting material to **41d** (petroleum ether/ethyl acetate = 2/1, R_f=0.80). The mixture was concentrated under reduced pressure, and resulting residue was quickly purified by silica gel column chromatography (petroleum ether/ethyl acetate/Et_3N = 2.5/1/0.1) to **41d** (72 mg, 68% over two steps) as a colorless oil. ^{1}H NMR (600 MHz, Acetone) δ 9.33 (s, 1H,N*H*), 7.49 – 7.16 (m, 15H), 6.46 (s, 1H, 1,H-1), 5.17 – 5.05 (m, 2H, C*H₂*-Bn), 4.88 (d, *J* = 11.4 Hz, 1H, C*H*H-Bn), 4.65 (d, *J* = 11.5 Hz, 1H, C*H*H-Bn), 4.48 – 4.42 (m, 2H, C*H₂*-Bn), 4.41 (t, *J* = 6.3 Hz, 1H, H-3), 4.35 (d, *J* = 5.9 Hz, 1H, H-2), 4.19 – 1.16 (m, 1H, H-3Hb), 4.04 (d, *J* = 9.6 Hz, 1H, H-5), 4.00 – 3.99 (m, 1H, H-6), 3.90 (dd, *J* = 9.9, 6.8 Hz, 1H, H-7a-Hep), 3.64 (dd, *J* = 9.9, 5.7 Hz, 1H, H-7b-Hep), 3.55 (dd, *J* = 9.6, 6.8 Hz, 1H, H-4-Hep), 2.67 (dd, *J* = 15.0, 6.2 Hz, 1H, H-2b-3Hb), 2.45 (dd, *J* = 15.0, 6.7 Hz, 1H, H-2a-3Hb), 1.53 (s, 3H, OCH_3), 1.37 (s, 3H, OCH_3), 1.21 (d, *J* = 6.2 Hz, 3H, CH_3-3Hb); ^{13}C NMR (151 MHz, Acetone) δ 170.52 (*C*OOBn), 159.26 (*C*=N), 138.77, 138.63, 136.61, 128.40, 128.38, 128.16, 128.13, 128.07, 127.90, 127.89, 127.40, 127.38, 127.28, 109.62 (*C*(CH_3)$_2$), 103.13 (C-1), 95.27 (*C*Cl_3), 78.73, 77.24, 75.29, 74.32, 72.73, 72.67, 72.18, 70.80, 65.69, 42.11, 27.25, 25.69, 20.06. HRMS (ESI): m/z calcd for $C_{37}H_{42}Cl_3NNaO_9^+$ $[M+Na]^+$ 772.1817, found 772.1814.

3.2 Synthesis of ADG building block.

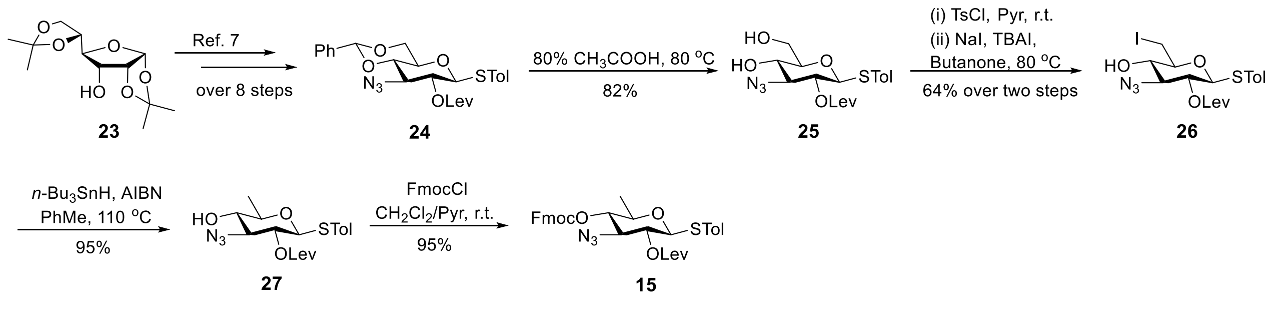

Scheme S5. Synthesis of ADG building block **15**

p-Tolyl 2-O-levulinoyl-3-azide-3-deoxy-1-thio-β-D-glucopyranoside (25)

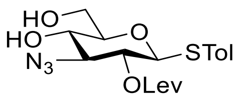

25

The compound **24**[7] (5.0 g, 10.0 mmol) was treated with 80% AcOH in water (10 mL) and the mixture was heated to 80 ºC for 6 h. TLC analysis showed complete conversion of starting material **24** to compound **25** (petroleum ether/ethyl acetate = 1/1, R_f = 0.20). The reaction mixture was cooled to room temperature and concentrated under reduced pressure. The resulting residue was purified by silica gel column chromatography (petroleum ether/ethyl acetate = 1/1) to give compound **25** (3.7 g, 90%) as a white amorphous solid. ^{1}H NMR (600 MHz, $CDCl_3$) δ 7.27 (d, J = 8.1 Hz, 2H, ArH), 7.02 (d, J = 8.2 Hz, 2H, ArH), 4.71 (t, J = 9.7 Hz, 1H, H-2), 4.55 (d, J = 10.0, 1H, H-1), 3.80 (dd, J = 12.2, 3.1 Hz, 1H, H-6a), 3.73 (dd, J = 12.3, 4.2 Hz, 1H, H-6b), 3.61 – 3.45 (m, 4H, H-3, H-4, 2 × OH), 3.30 (dd, J = 8.2, 4.1 Hz, 1H, H-5), 2.74 (t, J = 7.0 Hz, 2H, C*H₂*-Lev), 2.61 (t, J = 6.5 Hz, 2H, CH_2-Lev), 2.23 (s, 3H, CH_3), 2.12 (s, 3H, CH_3); ^{13}C NMR (151 MHz, $CDCl_3$) δ 206.82 (*C*=O-Lev), 171.56 (*C*=O-Lev), 138.45, 132.83, 129.86, 128.62, 86.68, 80.10 (C-1), 70.76, 68.71, 68.30, 61.68, 37.87, 29.85, 28.06, 21.14. HRMS (ESI): m/z calcd for $C_{18}H_{23}N_3NaO_6S^+$ $[M+Na]^+$ 432.1200, found 432.1202.

p-Tolyl 2-O-levulinoyl-3-azide-6-iodo-3,6-di-deoxy-thio-β-D-glucopyranoside (26)

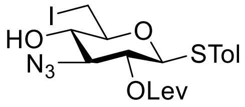

26

To a solution of **25** (3.6 g, 8.8 mmol) in pyridine (100 mL) was added *p*-toluenesulfonyl chloride (1.8 g, 9.7 mmol) at 0 ºC. After being stirred at room temperature for 8 h, the reaction was quenched with MeOH, and the reaction mixture was concentrated *in vacuo* to give a residue. The resulting residue was dissolved with ethyl acetate, and the mixture was washed with 1 M HCl, saturated aqueous $NaHCO_3$ and brine. The organic phase was dried (Na_2SO_4), and filtered. The filtrate was concentrated *in vacuo* and resulting residue was purified by silica gel column chromatography (CH_2Cl_2/MeOH = 60:1 to 40:1) to give compound 6-OTs intermediate as a white amorphous solid. To a solution of obtained 6-OTs intermediate (4.15 g, 7.5 mmol) in butanone (110 mL) were added the NaI (5.5 g, 36.8 mmol) and TBAI (0.54 g, 1.5 mmol). The reaction was stirred at 80 ºC for 8 h under an atmosphere of argon. TLC analysis showed that most of 6-OTs intermediate converted to compound **26** (petroleum ether/ethyl acetate =

2/1, R_f = 0.55). After cooling to room temperature, the mixture was diluted with ethyl acetate and washed with aqueous $Na_2S_2O_3$. The aqueous phase was re-extracted twice with ethyl acetate. The combined organic phase was washed with brine, dried (Na_2SO_4) and filtered, and filtrate was concentrated under reduced pressure. The resulting residue was purified by silica gel column chromatography (petroleum ether/ethyl acetate = 4/1) to give compound **26** (2.9 g, 64% over two steps) as an off-white amorphous solid. ^{1}H NMR (600 MHz, $CDCl_3$) δ 7.47 (d, *J* = 8.1 Hz, 2H, ArH), 7.10 (d, *J* = 8.1 Hz, 2H, ArH), 4.78 (t, *J* = 9.9 Hz, 1H, H-2), 4.61 (d, *J* = 10.0 Hz, 1H, H-1), 3.58 (dd, *J* = 11.2, 2.5 Hz, 1H, H-6a), 3.56 (t, *J* = 9.6 Hz, 1H, H-3), 3.35 (t, *J* = 9.3 Hz, 1H, H-4), 3.31 (dd, *J* = 11.2, 6.8 Hz, 1H, H-6b), 3.23 – 3.20 (m, 1H, H-5), 2.83 (t, *J* = 6.8 Hz, 2H, C*H₂*-Lev), 2.70 – 2.67 (m, 2H, C*H₂*-Lev), 2.31 (s, 3H, C*H₃*), 2.21 (s, 3H, C*H₃*); ^{13}C NMR (151 MHz, $CDCl_3$) δ 207.09 (*C*=O-Lev), 171.38 (*C*=O-Lev), 138.53, 133.74, 129.65, 127.80, 86.21 (C-1), 78.86, 72.47, 70.61, 68.05, 37.83, 29.93, 28.00, 21.20, 5.41 (C-6). HRMS (ESI): m/z calcd for $C_{18}H_{21}IN_3O_5S^-$ [M-H]$^-$ 518.0252, found 518.0251.

_p_-Tolyl 2-_O_-levulinoyl-3-azide-3,6-di-deoxy-thio-_β_-D-glucopyranoside (27)

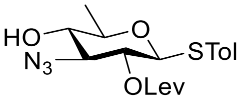

27

To solution of a mixture of **26** (1.2 g, 2.3 mmol) in degassed PhMe (50 mL) were added AIBN (76 mg, 0.46 mmol) and Bu_3SnH (685 μL, 2.5 mmol) at room temperature under an atmosphere of argon. The mixture was heated to 110 °C and stirred for 40 min. TLC analysis showed complete conversion of starting material **26** to compound **27** (petroleum ether/ethyl acetate = 2/1, R_f = 0.50). The reaction mixture was cooled to room temperature and concentrated under reduced pressure to give crude product. The resulting crude product was purified by silica gel column chromatography (petroleum ether/ethyl acetate = 6/1) to give compound **27** (0.83 g, 91%) as a yellow amorphous solid. ^{1}H NMR (600 MHz, $CDCl_3$) δ 7.35 (d, *J* = 8.2 Hz, 2H, ArH), 7.09 (d, *J* = 8.1 Hz, 2H, ArH), 4.79 (t, *J* = 9.9 Hz, 1H, H-2), 4.56 (d, *J* = 10.0 Hz, 1H, H-1), 3.48 (t, *J* = 9.6 Hz, 1H, H-3), 3.42 (s, 1H, OH), 3.40 – 3.33 (m, 1H, H-5), 3.24 – 3.10 (m, 1H, H-4), 2.81 (t, *J* = 6.8 Hz, 2H, C*H₂*-Lev), 2.67 (t, *J* = 6.8 Hz, 2H, C*H₂*-Lev), 2.31 (s, 3H, C*H₃*), 2.19 (s, 3H, C*H₃*), 1.32 (d, *J* = 6.2 Hz, 3H, H-6); ^{13}C NMR (151 MHz, $CDCl_3$) δ 206.84 (*C*=O-Lev), 171.45 (*C*=O-Lev), 138.26, 132.97, 129.67, 128.70, 86.37 (C-1), 76.59, 73.96, 71.02, 68.50, 37.81, 29.84, 27.99, 21.13, 17.76 (C-6). HRMS (ESI): m/z calcd for $C_{18}H_{23}N_3NaO_5S^+$ [M+Na]$^+$ 416.1251, found 416.1254.

***p*-Tolyl 2-*O*-levulinoyl-3-azide-4-*O*-fluorenylmethoxycarbony-3,6-di-deoxy-l-thio-*β*-D-glucopyranoside (15)**

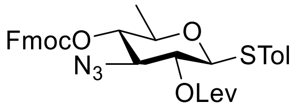

15

To solution of a mixture of **27** (428 mg, 1.1 mmol) in a mixed solvent of pyridine (1 mL) and CH_2Cl_2 (6 mL) was added FmocCl (422 mg, 1.6 mmol) at 0 °C under an atmosphere of argon. The mixture was stirred at room temperature for 4 h. TLC analysis showed complete conversion of starting material **27** to compound **15** (petroleum ether/ethyl acetate = 2/1, R_f = 0.75). The reaction was quenched with MeOH, and the reaction mixture was concentrated *in vacuo*. The resulting residue was dissolved with CH_2Cl_2, and the mixture was washed with 1 M HCl, saturated aqueous $NaHCO_3$ and brine. The organic phase was dried (Na_2SO_4), and filtered. The filtrate was concentrated *in vacuo* and resulting residue was purified by silica gel column chromatography (petroleum ether/ethyl acetate/CH_2Cl_2 = 10/1/1 to 5/1/1) to give compound **15** (595 mg, 89%) as a white amorphous solid. ^{1}H NMR (600 MHz, $CDCl_3$) δ 7.86 – 7.74 (m, 2H, ArH), 7.61 (d, *J* = 7.5 Hz, 2H, ArH), 7.47 – 7.38 (m, 4H, ArH), 7.37 – 7.30 (m, 2H, ArH), 7.14 (d, *J* = 8.1 Hz, 2H, ArH), 4.89 (t, *J* = 9.8 Hz, 1H, H-2), 4.60 – 4.49 (m, 3H, H-1, C*H*2-Fmoc), 4.46 (t, *J* = 9.8 Hz, 1H, H-4), 4.27 (t, *J* = 7.0 Hz, 1H, C*H*-Fmoc), 3.64 (t, *J* = 9.8 Hz, 1H, H-3), 3.56 – 3.51 (m, 1H, H-5), 2.87 – 2.81 (m, 2H, C*H*2-Lev), 2.76 – 2.66 (m, 2H, C*H*2-Lev), 2.36 (s, 3H, C*H*3), 2.22 (s, 3H, C*H*3), 1.25 (d, *J* = 6.2 Hz, 3H, H-6); ^{13}C NMR (151 MHz, $CDCl_3$) δ 205.91 (*C*=O-Lev), 171.11 (*C*=O-Lev), 154.25 (*C*=O-Fmoc), 143.14, 142.98, 141.34, 138.66, 133.64, 129.74, 127.98, 127.22, 125.03, 124.99, 120.14, 86.21 (C-1), 77.00, 74.68, 70.77, 70.21, 65.76, 46.78, 37.79, 29.82, 27.96, 21.19, 17.43 (C-6). HRMS (ESI): m/z calcd for $C_{33}H_{33}N_3NaO_7S^+$ $[M+Na]^+$ 638.1931, found 638.1930.

3.3 Synthesis of AAT building blocks.

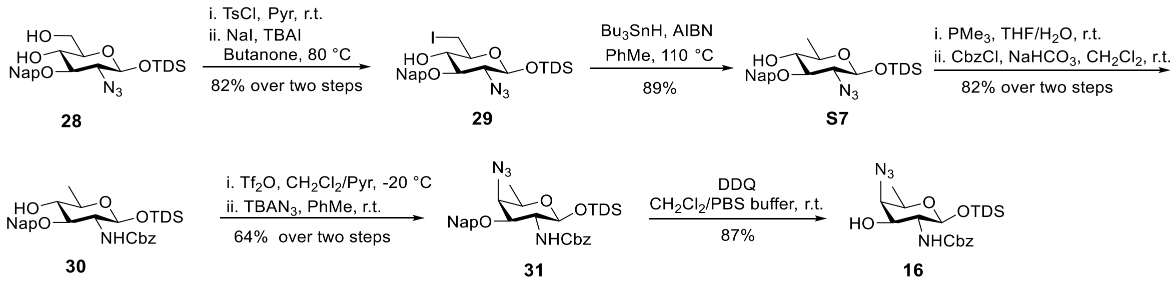

Scheme S6. Synthesis of AAT building block **16**

Dimethylthexylsilyl 2-azide-3-*O*-naphthylmethyl-6-iodo-2,6-di-deoxy-*β*-D-glucopyranoside (29)

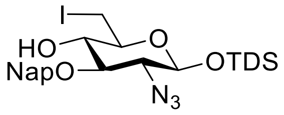

29

To a solution of **28**[8] (2.9 g, 6.0 mmol) in pyridine (50 mL) was added *p*-toluenesulfonyl chloride (2.3 g, 11.9 mmol) at 0 ºC. After being stirred at room temperature for 6 h, TLC analysis showed complete conversion of starting material **28** to an 6-OTs intermediate (petroleum ether/ethyl acetate = 3/1, R_f = 0.60). The reaction was quenched with MeOH, and the reaction mixture was concentrated *in vacuo*. The resulting residue was dissolved with ethyl acetate, and the mixture was washed with 1 M HCl, saturated aqueous $NaHCO_3$ and brine. The organic phase was dried (Na_2SO_4), and filtered. The filtrate was concentrated *in vacuo* and resulting residue was purified by silica gel column chromatography (petroleum ether/ethyl acetate/CH_2Cl_2 = 6/1/1 to 3/1/1) to give compound 6-OTs intermediate (3.5 g) as a colorless oil.

To a solution of obtained 6-OTs intermediate (3.5 g, 5.5 mmol) in butanone (50 mL) were added the NaI (4.1 g, 27.3 mmol) and TBAI (0.40 g, 1.1 mmol). The reaction was stirred at 80 ºC for 8 h under an atmosphere of argon. TLC analysis showed complete conversion of 6-OTs intermediate to compound **29** (petroleum ether/ethyl acetate = 3/1, R_f = 0.85). After cooling to room temperature, the mixture was diluted with ethyl acetate and washed with aqueous $Na_2S_2O_3$. The aqueous phase was re-extracted twice with ethyl acetate. The combined organic phase was washed with brine, dried (Na_2SO_4) and filtered, and filtrate was concentrated under reduced pressure. The resulting residue was purified by silica gel column chromatography (petroleum ether/ethyl acetate = 15/1 to 10/1) to give compound **29** (3.1 g, 82% over two steps) as a colorless oil. ^{1}H NMR (600 MHz, $CDCl_3$) δ 8.01 – 7.76 (m, 4H, ArH), 7.52 – 7.48 (m, 3H, ArH), 5.13 (d, *J* = 11.7 Hz, 1H, C*H*H-Nap), 4.83 (d, *J* = 11.7 Hz, 1H, C*H*H-Nap), 4.58 (d, *J* = 7.6 Hz, 1H, H-1), 3.52 (dd, *J* = 10.6, 2.2 Hz, 1H), 3.44 – 3.33 (m, 2H), 3.28 – 3.17 (m, 2H), 3.16 – 3.11 (m, 1H), 1.76 – 1.67 (m, 1H, C*H*(CH_3)$_2$-TDS), 0.99 – 0.85 (m, 12H, CH(C*H*$_3$)$_2$-TDS, C(C*H*$_3$)$_2$-TDS), 0.29 (s, 3H, SiC*H*$_3$-TDS), 0.27 (s, 3H, SiC*H*$_3$-TDS); ^{13}C NMR (151 MHz, $CDCl_3$) δ 135.37, 133.43, 133.27, 128.87, 128.10, 127.93, 127.25, 126.54, 126.39, 125.86, 97.15 (C-1), 81.87 , 77.37, 75.13, 75.04, 73.66, 68.77, 34.03, 24.95, 20.12, 20.07, 18.63, 18.56, 5.13, -1.61 (Si*C*H_3-TDS), -3.11 (Si*C*H_3-TDS). HRMS (ESI): m/z calcd for $C_{25}H_{36}IN_3NaO_4Si^+$ [M+Na]$^+$ 620.1412, found 620.1412.

Dimethylthexylsilyl 2-azide-3-*O*-naphthylmethyl-2,6-di-deoxy-*β*-D-glucopyranoside (S7)

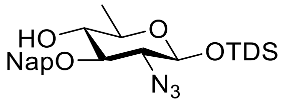

S7

To solution of **29** (400 mg, 0.67 mmol) in degassed PhMe (5 mL) were added AIBN (22 mg, 0.13 mmol) and Bu_3SnH (208 μL, 0.74 mmol) at room temperature under an atmosphere of argon. The mixture was heated to 110 °C and stirred for 40 min. TLC analysis showed complete conversion of starting material **29** to compound **S7** (petroleum ether/ethyl acetate = 2/1, R_f = 0.50). The reaction mixture was cooled to room temperature and concentrated under reduced pressure. The resulting residue was purified by silica gel column chromatography (petroleum ether/ethyl acetate = 15/1 to 10/1) to give compound **S7** (284 mg, 89%) as a colorless oil. ^{1}H NMR (600 MHz, $CDCl_3$) δ 7.97 – 7.75 (m, 4H, ArH), 7.63 – 7.39 (m, 3H, ArH), 5.13 (d, J = 11.7 Hz, 1H, C*H*H-Nap), 4.85 (d, J = 11.7 Hz, 1H, C*H*H-Nap), 4.52 (d, J = 7.6 Hz, 1H, H-1), 3.35 (dd, J = 9.9, 7.6 Hz, 1H), 3.30 – 3.23 (m, 2H), 3.22 – 3.14 (m, 1H), 1.73 – 1.62 (m, 1H, C*H*$(CH_3)_2$-TDS), 1.28 (d, J = 5.7 Hz, 3H, CH_3), 1.06 – 0.83 (m, 12H, CH(C*H*$_3$)$_2$-TDS, C(C*H*$_3$)$_2$-TDS), 0.20 (d, J = 3.8 Hz, 6H, 2 × SiC*H*$_3$-TDS); ^{13}C NMR (151 MHz, $CDCl_3$) δ 135.63, 133.46, 133.26, 128.78, 128.11, 127.91, 127.15, 126.44, 126.28, 125.93, 97.04 (C-1), 82.58, 75.25, 75.04, 71.59, 68.89, 34.02, 24.98, 20.09, 19.99, 18.63, 18.53, 17.83, -1.94 (Si*C*H$_3$-TDS), -3.10 (Si*C*H$_3$-TDS). HRMS (ESI): m/z calcd for $C_{25}H_{37}N_3NaO_4Si^+$ $[M+Na]^+$ 494.2446, found 494.2449.

Dimethylthexylsilyl 2-*N*-benzyloxycarbonyl-3-*O*-naphthylmethyl-2,6-di-deoxy-*β*-D-glucopyranoside (30)

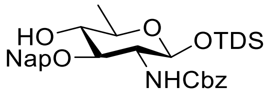

30

To a solution of compound **S7** (500 mg, 1.1 mmol) in a mixed solvent of THF/H_2O (v/v, 5/1, 6 mL) was added PMe_3 (1 M in THF, 1.6 mL). The reaction was stirred at room temperature for 1.5 h. TLC analysis showed complete conversion of starting material **S7** to an amine intermediate (CH_2Cl_2/MeOH = 20/1, R_f = 0.50). The reaction mixture was concentrated under reduced pressure and co-evaporated three times with PhMe.

The resulting residue was dissolved in CH_2Cl_2 (5 mL) and saturated aqueous $NaHCO_3$ (0.5 mL). CbzCl (182 μL, 1.3 mmol) was added at 0 °C. The reaction mixture was stirred at room temperature for 0.5 h. TLC analysis showed complete conversion of amine intermediate to compound **30** (petroleum ether/ethyl acetate = 2/1, R_f = 0.50). The

organic phase was dried (Na_2SO_4), and filtered. The filtrate was concentrated *in vacuo* to give a residue. The resulting residue was purified by silica gel column chromatography (petroleum ether/ethyl acetate/CH_2Cl_2 = 10/1/1 to 5/1/1) to give compound **30** (505 mg, 82% over two steps) as a colorless oil. ^{1}H NMR (500 MHz, $CDCl_3$) δ 7.86 – 7.66 (m, 4H, ArH), 7.50 – 7.28 (m, 8H, ArH), 5.19 – 4.98 (m, 2H), 4.93 – 4.75 (m, 3H), 3.80 (s, 1H), 3.58 – 3.06 (m, 3H), 1.76 – 1.53 (m, 1H, C*H*(CH_3)$_2$-TDS), 1.28 (d, *J* = 5.8 Hz, 3H, C*H$_3$*), 1.09 – 0.78 (m, 12H, CH(C*H$_3$*)$_2$-TDS, C(C*H$_3$*)$_2$-TDS), 0.16 (s, 3H, SiC*H$_3$*-TDS), 0.10 (s, 3H, SiC*H$_3$*-TDS); ^{13}C NMR (126 MHz, $CDCl_3$) δ 155.91 (*C*=O-Cbz), 136.52, 135.82, 133.33, 133.09, 128.58, 128.48, 128.28, 128.21, 128.13, 128.06, 127.80, 126.90, 126.26, 126.10, 126.01, 95.38 (C-1), 80.96, 75.90, 73.75, 71.52, 66.79, 59.66, 34.07, 24.87, 20.12, 20.06, 18.62, 18.59, 17.85, -1.78 (Si*C*H$_3$-TDS), -3.46 (Si*C*H$_3$-TDS). HRMS (ESI): m/z calcd for $C_{33}H_{45}NNaO_6Si^+$ $[M+Na]^+$ 602.2908, found 602.2910.

Dimethylthexylsilyl 4-azide-3-*O*-naphthylmethyl-2-*N*-benzyloxycarbonyl-2,4,6-tri-deoxy-*β*-D-galactopyranoside (31)

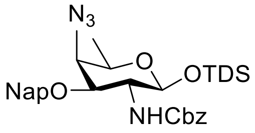

31

To a solution of **30** (450 mg, 0.78 mmol) in a mixed solvent of CH_2Cl_2/pyridine (v/v, 5/1, 6 mL) was added Tf_2O (0.20 mL, 1.16 mmol) at -20 °C under an atmosphere of argon. After being stirred at this temperature for 1.5 h, the reaction was quenched by adding H_2O. The organic phase was washed with 1 M HCl, saturated aqueous $NaHCO_3$ and brine. The organic phase was dried (Na_2SO_4), and filtered. The filtrate was concentrated *in vacuo* to give the 4-OTf intermediate.

To a solution of above 4-OTf intermediate in anhydrous PhMe (5 mL) was added $TBAN_3$ (400 mg, 1.55 mmol) under an atmosphere of argon. The reaction was stirred at room temperature overnight. TLC analysis showed complete conversion of starting material to a major compound **31** (petroleum ether/ethyl acetate = 4/1, R_f = 0.45). The reaction was diluted with CH_2Cl_2, and the mixture was washed with H_2O and brine. The organic phase was dried (Na_2SO_4), and filtered. The filtrate was concentrated *in vacuo* and purified by silica gel column chromatography (petroleum ether/ethyl acetate = 10/1) to give compound **31** (300 mg, 64% over two steps) as a white amorphous solid. ^{1}H NMR (600 MHz, CD_3OD) δ 7.88 – 7.68 (m, 4H, ArH), 7.51 – 7.40 (m, 3H, ArH), 7.35 – 7.20 (m, 5H, ArH), 5.03 (d, *J* = 12.5 Hz, 1H, C*H*H), 4.97 (d, *J* = 12.4 Hz, 1H, C*H*H), 4.88 (d, *J* = 12.0 Hz, 1H,

C*H*H), 4.71 (d, *J* = 12.0 Hz, 1H, C*H*H), 4.62 (d, *J* = 7.9 Hz, 1H, H-1), 3.97 (d, *J* = 4.0 Hz, 1H), 3.83 (d, *J* = 10.3 Hz, 1H), 3.66 – 3.61 (m, 2H), 1.68 – 1.55 (m, 1H, C*H*(CH_3)$_2$-TDS), 1.27 (d, *J* = 6.4 Hz, 3H, C*H$_3$*), 0.95 – 0.76 (m, 12H, CH(C*H$_3$*)$_2$-TDS, C(C*H$_3$*)$_2$-TDS), 0.12 (s, 3H, SiC*H$_3$*-TDS), 0.09 (s, 3H, SiC*H$_3$*-TDS); ^{13}C NMR (151 MHz, CD_3OD) δ 158.66 (*C*=O-Cbz), 138.34, 137.00, 134.72, 134.48, 129.43, 129.04, 128.99, 128.95, 128.93, 128.66, 128.41, 127.44, 127.08, 126.93, 126.84, 97.64 (C-1), 80.45, 73.21, 70.14, 67.34, 64.16, 56.92, 35.30, 25.84, 20.59, 19.07, 19.05, 17.98, -1.68 (Si*C*H_3-TDS), -3.14 (Si*C*H_3-TDS). HRMS (ESI): m/z calcd for $C_{33}H_{44}N_4NaO_5Si^+$ $[M+Na]^+$ 627.2973, found 627.2975.

Dimethylthexylsilyl 4-azide-2-*N*-benzyloxycarbonyl-2,4,6-tri-deoxy-*β*-D-galactopyranoside (16)

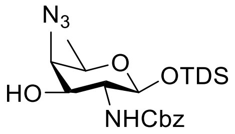

16

To solution of a mixture of **31** (500 mg, 0.83 mmol) in a mixed solvent of CH_2Cl_2/0.1 M PBS buffer (v/v, 10/1, 5.5 mL) was added DDQ (375 mg, 1.65 mmol) at 0 °C. After being stirred at room temperature for 5 h, the reaction was diluted with ethyl acetate, and washed with saturated aqueous $NaHCO_3$ and brine. The organic phase was dried (Na_2SO_4), and filtered. The filtrate was concentrated under reduced pressure to give crude product. The crude product was purified by silica gel column chromatography (petroleum ether/ethyl acetate/CH_2Cl_2= 3/1/1) to give compound **16** (333 mg, 87%) as a colorless oil. ^{1}H NMR (600 MHz, CD_3OD) δ 7.61 – 7.01 (m, 5H. ArH), 5.05 (s, 2H, C*H$_2$*-Cbz), 4.55 (d, *J* = 7.9 Hz, 1H, H-1), 3.85 (dd, *J* = 10.7, 3.8 Hz, 1H), 3.70 – 3.64 (m, 2H), 3.51 (dd, *J* = 10.7, 7.9 Hz, 1H), 1.64 – 1.58 (m, 1H, C*H*(CH_3)$_2$-TDS), 1.28 (d, *J* = 6.4 Hz, 3H, C*H$_3$*), 0.99 – 0.74 (m, 12H, CH(C*H$_3$*)$_2$-TDS, C(C*H$_3$*)$_2$-TDS), 0.13 (s, 3H, SiC*H$_3$*-TDS), 0.10 (s, 3H, SiC*H$_3$*-TDS); ^{13}C NMR (151 MHz, CD_3OD) δ 158.87 (*C*=O-Cbz), 138.24, 129.40, 129.04, 128.93, 97.95, 72.81, 70.41, 67.93, 67.46, 57.76, 35.24, 25.83, 20.57, 19.04, 17.90, -1.65 (Si*C*H_3-TDS), -3.11 (Si*C*H_3-TDS). HRMS (ESI): m/z calcd for $C_{22}H_{36}N_4NaO_5Si^+$ $[M+Na]^+$ 487.2347, found 487.2345.

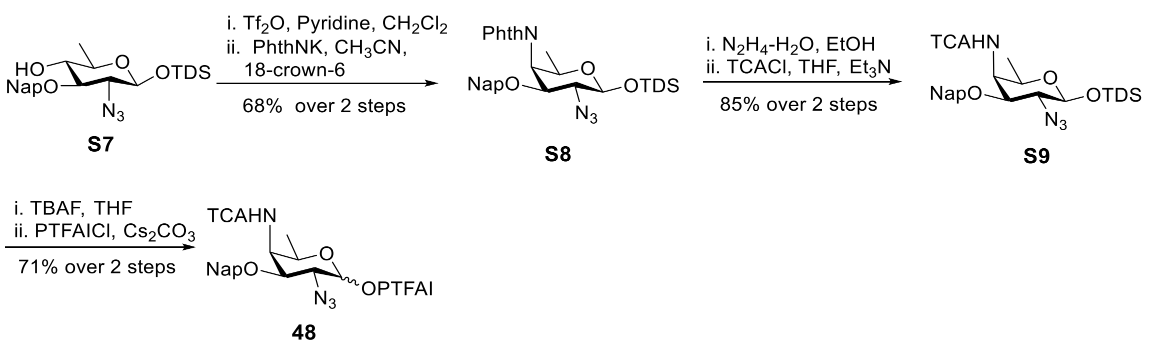

Scheme S7. Synthesis of AAT building block **48**

Dimethylthexylsilyl 2-azide-3-*O*-naphthylmethyl-4-*N*-phthaloyl-2,4,6-tri-deoxy-*β*-D-galactopyranoside (S8)

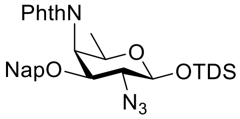

S8

To a solution of **S7** (1.1 g, 2.3 mmol) in a mixed solvent of CH_2Cl_2/pyridine (v/v, 5/1, 12 mL) was added Tf_2O (0.59 mL, 3.5 mmol) at -20 °C under an atmosphere of argon. After being stirred at this temperature for 1.5 h, the reaction was quenched by adding H_2O. The organic phase was washed with 1 M HCl, saturated aqueous $NaHCO_3$ and brine. The organic phase was dried (Na_2SO_4), and filtered. The filtrate was concentrated *in vacuo* to give the 4-OTf intermediate.

To a solution of above 4-OTf intermediate in anhydrous CH_3CN (25 mL) was added PhthK (864 mg, 4.7 mmol) and 18-crown-6 (0.62 g, 2.3 mmol) an atmosphere of argon.[9] The reaction was heated to 40 °C for 8 h. TLC analysis showed complete conversion of starting material to a major compound **S8** (petroleum ether/ethyl acetate = 6/1, R_f = 0.65). The reaction was diluted with CH_2Cl_2, and the mixture was washed with H_2O and brine. The organic phase was dried (Na_2SO_4), and filtered. The filtrate was concentrated *in vacuo* and purified by silica gel column chromatography (petroleum ether/ethyl acetate = 25/1) to give compound **S8** (1.1 g, 68% over two steps) as a yellow oil. ^{1}H NMR (600 MHz, $CDCl_3$) δ 7.88 – 7.59 (m, 8H, ArH), 7.45 – 7.40 (m, 2H, ArH), 7.34 (dd, J = 8.4, 1.8 Hz, 1H, ArH), 4.76 – 4.70 (m, 2H, H-4, C*H*H-Nap), 4.66 (d, J = 11.4 Hz, 1H, C*H*H-Nap), 4.52 – 4.44 (m, 2H, H-1, H-3), 3.75 (dd, J = 6.4, 3.2 Hz, 1H, H-5), 3.64 (dd, J = 9.5, 7.1 Hz, 1H, H-2), 1.83 – 1.63 (m, 1H, C*H*(CH_3)$_2$-TDS), 1.12 (d, J = 6.4 Hz, 3H, H-6), 1.00 – 0.79 (m, 12H, CH(C*H$_3$*)$_2$-TDS, C(C*H$_3$*)$_2$-TDS), 0.19 (d, J = 6.5 Hz, 6H, 2 × SiC*H$_3$*-TDS); ^{13}C NMR (151 MHz, $CDCl_3$) δ 160.65 (*C*=O-Phth), 134.80, 133.22, 133.11, 128.29, 128.01, 127.77,

126.70, 126.17, 126.04, 125.70, 98.02 (C-1), 76.67, 71.94, 69.50, 66.53, 51.30, 34.09, 25.01, 20.23, 20.09, 18.69, 18.59, 16.88, -1.80 (Si*C*H_3-TDS), -2.98 (Si*C*H_3-TDS). HRMS (ESI): m/z calcd for $C_{33}H_{40}N_4NaOSSi^+$ $[M+Na]^+$ 623.2660, found 623.2662.

Dimethylthexylsilyl 2-azide-4-trichloroacetamido-3-*O*-naphthylmethyl-2,4,6-tri-deoxy-*β*-D-galactopyranoside (S9)

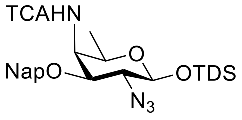

S9

To a solution of **S8** (400 mg, 0.67 mmol) in EtOH (10 mL) was added N_2H_4-H_2O (85%, 0.16 mL, 2.6 mmol). The mixture was stirred at 80 °C overnight. TLC analysis showed complete conversion of starting material to AN amine intermediate (CH_2Cl_2/MeOH = 20/1, R_f = 0.30). The reaction mixture was cooled to room temperature and concentrated under reduced pressure. The resulting residue was co-evaporated three times with PhMe to give amine intermediate for the next step.

To a solution of above amine intermediate in a mixed solvent of THF/Et_3N (v/v, 10/1, 5.5 mL) was added TCACl (0.09 mL, 0.8 mmol) at 0 °C. The reaction was stirred at room temperature for 0.5 h. TLC analysis showed complete conversion of starting material to compound **S9** (petroleum ether/ethyl acetate = 3/1, R_f = 0.72). The reaction was quenched by adding H_2O and diluted with CH_2Cl_2. The organic phase was washed with 1 M HCl, saturated aqueous $NaHCO_3$ and brine. The organic phase was dried (Na_2SO_4), and filtered. The filtrate was concentrated *in vacuo* and was purified by silica gel column chromatography (petroleum ether/ethyl acetate = 15/1) to give compound **S9** (350 mg, 85% over two steps) as a colorless oil. ^{1}H NMR (600 MHz, $CDCl_3$) δ 7.96 – 7.79 (m, 4H, ArH), 7.64 – 7.42 (m, 3H, ArH), 6.88 (d, J = 9.9 Hz, 1H, N*H*), 4.98 (d, J = 11.6 Hz, 1H, C*H*H-Nap), 4.70 (d, J = 11.6 Hz, 1H, C*H*H-Nap), 4.53 – 4.38 (m, 2H, H-1, H-4), 3.67 – 3.60 (m, 1H, H-5), 3.47 (dd, J = 10.3, 4.3 Hz, 1H, H-3), 3.23 (dd, J = 10.3, 7.7 Hz, 1H, H-2), 1.77 – 1.62 (m, 1H, C*H*(CH_3)$_2$-TDS), 1.26 (d, J = 6.4 Hz, 3H, H-6), 1.00 – 0.75 (m, 12H, CH(C*H_3*)$_2$-TDS, C(C*H_3*)$_2$-TDS), 0.18 (s, 6H, 2 × SiC*H_3*-TDS); ^{13}C NMR (126 MHz, $CDCl_3$) δ 162.96 (*C*=O-TCA), 134.70, 133.35, 133.28, 128.39, 128.13, 127.83, 127.36, 126.26, 126.21, 126.13, 97.25 (C-1), 92.71 (*C*Cl_3), 77.41, 71.74, 69.45, 65.83, 51.69, 34.10, 25.03, 20.16, 20.08, 18.62, 18.56, 16.95, -2.01 (Si*C*H_3-TDS), -2.98 (Si*C*H_3-TDS). HRMS (ESI): m/z calcd for $C_{27}H_{36}Cl_3N_4O_4Si^-$ $[M-H]^-$ 613.1577, found 613.1579.

N-phenyl-trifluoroacetimidoyl 2-azide-4-trichloroacetamido-3-O-naphthylmethyl-2,4,6-tri-deoxy-α/β-D-galactopyranoside (48)

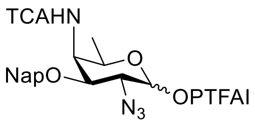

48

To a solution of compound **S9** (350 mg, 0.57 mmol) in THF (5 mL) was added TBAF (1 M in THF, 0.85 mL, 0.85 mmol) at 0 °C. The reaction was sitrred at room temperature for 12 h. TLC analysis showed complete conversion of starting material to hemiacetal intermediate. The reaction was concentrated under reduced pressure and purified by silica gel column chromatography (petroleum ether/ethyl acetate = 3:1) to give the hemiacetal intermediate (217 mg, 81%) as a colorless oil that was used for the next step.

The above hemiacetal intermediate (50 mg, 0.11 mmol) was dissolved in anhydrous CH_2Cl_2 (2 mL), and *N*-phenyl trifluoroacetimidoyl chloride (26 μL, 0.16 mmol) and Cs_2CO_3 (51 mg, 0.16 mmol) were added. After being stirred for 2 h at room temperature, TLC analysis showed complete conversion of hemiacetal intermediate to compound **48** (petroleum ether/ethyl acetate = 4/1, R_f = 0.75). The reaction mixture was filtered through celite, and concentrated *in vacuo*. The resulting residue was purified by silica gel column chromatography (petroleum ether/ethyl acetate = 10:1) to give compound **48** (60 mg, 88%) as a colorless oil that was immediately used for the next glycosylation.

3.4 Synthesis of ManN building blocks

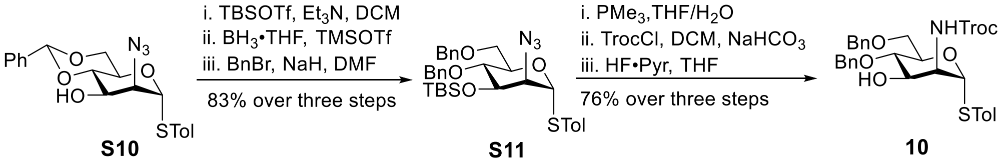

Scheme S8. Synthesis of ManN building block **10**

p-Tolyl 2-azide-3-O-$tert$-butyldimethylsilyl-4,6-di-O-benzyl-2-deoxy-1-thio-α-D-mannopyranoside (S11)

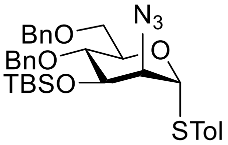

S11

To a solution of a mixture of **S10**[10] (1.65 g, 4.1 mmol) in CH_2Cl_2 (20 mL) was added TBSOTf (1.9 mL, 8.2 mmol) and Et_3N (1.7 mL, 12.4 mmol) at 0 ºC under an atmosphere of argon. The mixture was stirred at room temperature for 1 h. TLC analysis showed complete conversion of starting material **S10** to 3-OTBS intermediate (petroleum ether/ethyl acetate = 4/1, R_f = 0.85). The reaction was quenched with MeOH, and the reaction mixture was concentrated *in vacuo*. The resulting residue was directly used for the next step.

To a solution of above 3-OTBS intermediate in anhydrous CH_2Cl_2 (10 mL) was added BH_3-THF(1 M in THF, 8 mL, 8 mmol) and TMSOTf (0.15 mL, 0.8 mmol) at 0 ºC under an atmosphere of argon. The mixture was stirred at room temperature for 3 h. TLC analysis showed complete conversion of starting material to 6-OH intermediate (petroleum ether/ethyl acetate = 4/1, R_f = 0.70). The reaction was cooled to 0 ˚C and quenched slowly by the addition of MeOH until bubbling subsided. The reaction mixture was concentrated *in vacuo*. The resulting residue was directly used for the next step.

To a solution of above 6-OH intermediate in DMF (10 mL) was added NaH (60% in mineral oil, 192 mg, 4.8 mmol) and BnBr (0.57 mL, 4.8 mmol) at 0 ºC under an atmosphere of argon. After being stirred at room temperature for 2 h, TLC analysis showed complete conversion of starting material to compound **S11** (petroleum ether/ethyl acetate = 6/1, R_f = 0.65). The reaction was quenched by the addition of MeOH and concentrated i*n vacuo*. The residue was dissolved with CH_2Cl_2, and washed with 1 M HCl, saturated aqueous $NaHCO_3$ and brine. The organic phase was dried (Na_2SO_4), and filtered. The filtrate was concentrated *in vacuo* and resulting residue was purified by silica gel column chromatography (petroleum ether/ethyl acetate = 20/1) to give compound **S11** (2.0 g, 83% over three steps) as a colorless oil. ^{1}H NMR (600 MHz, $CDCl_3$) δ 7.50 – 7.27 (m, 12H, ArH), 7.12 (d, J = 8.2 Hz, 2H, ArH), 5.46 (d, J = 1.8 Hz, 1H, H-1), 4.92 (d, J = 11.2 Hz, 1H, C*H*H-Bn), 4.66 (d, J = 12.0 Hz, 1H, C*H*H-Bn), 4.61 (d, J = 11.1 Hz, 1H, C*H*H-Bn), 4.52 (d, J = 12.0 Hz, 1H, C*H*H-Bn), 4.40 – 4.32 (m, 1H, H-5), 4.29 (dd, J = 8.9, 3.6 Hz, 1H, H-3), 4.08 (dd, J = 3.6, 1.8 Hz, 1H,H-2), 3.91 (t, J = 9.3 Hz, 1H, H-4), 3.82 (dd, J = 11.0, 5.0 Hz, 1H, H-6), 3.73 (dd, J = 11.0, 2.0 Hz, 1H, H-6), 2.36 (s, 3H, CH_3-STol), 1.05 (s, 9H, (CH_3)$_3$-TBS), 0.25 (s, 3H, CH_3-TBS), 0.23 (s, 3H, CH_3-TBS); ^{13}C NMR (151 MHz, $CDCl_3$) δ 138.28, 138.20, 138.06, 132.45, 129.95, 129.82, 128.38, 128.34, 127.85, 127.68, 127.57, 86.84 (C-1), 76.95, 75.98, 75.22, 73.58, 73.45, 73.01, 68.95, 66.34, 25.99, 21.18, 18.11, -4.30

(CH_3Si), -4.52 (CH_3Si). HRMS (ESI): m/z calcd for $C_{33}H_{47}N_4O_4SSi^+$ $[M+NH_4]^+$ 623.3082, found 623.3081.

***p*-Tolyl 2-*N*-(2,2,2-trichloroethyloxy)carbonylamino-4,6-di-*O*-benzyl-2-deoxy-1-thio-*α*-D-mannopyranoside (10)**

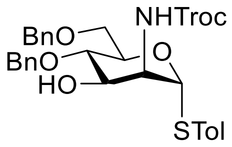

10

To a solution of **S11** (1.6 g, 2.6 mmol) in a mixed solvent of THF/H_2O (v/v, 9/1, 20 mL) was added PMe_3 (1 M in THF, 5 mL) at 0 ºC. The reaction was stirred at room temperature for 2 h. TLC analysis showed complete conversion of starting material to a major amine intermediate. The reaction mixture was concentrated *in vacuo*. The resulting residue was co-evaporated three times with PhMe and used for the next step.

To a solution of above amine intermediate in a mixed solvent of CH_2Cl_2/saturated $NaHCO_3$ solution (v/v, 9/1, 40 mL) was added the TrocCl (0.55 mL, 4.0 mmol). The reaction was stirred at room temperature for 1 h. TLC analysis showed complete conversion of starting material to a 2-NHTroc intermediate (petroleum ether/ethyl acetate = 8/1, R_f = 0.75). The reaction diluted with CH_2Cl_2, and washed with brine and dried over Na_2SO_4. The filtration was concentrated under reduced pressure. The resulting residue was directly used for the next step.

To a solution of above 2-NHTroc intermediate in THF (15 mL) was added HF/pyridine (70%, 1.5 mL) at 0 ºC. The reaction was stirred at room temperature overnight. TLC analysis showed complete conversion of starting material to compound **10** (petroleum ether/ethyl acetate = 4/1, R_f = 0.25). The reaction was quenched with saturated $NaHCO_3$ solution and diluted with CH_2Cl_2. The organic phase was separated and washed with brine and dried over Na_2SO_4. The filtration was concentrated under reduced pressure and purified by silica gel column chromatography (petroleum ether/ethyl acetate = 4/1) to give **10** (1.3 g, 77% over three steps) as a colorless oil. ^{1}H NMR (600 MHz, $CDCl_3$) δ 7.43 – 7.24 (m, 12H, ArH), 7.09 (d, J = 8.2 Hz, 2H, ArH), 5.95 (d, J = 8.4 Hz, 1H, N*H*), 5.48 (s, 1H, H-1), 4.83 (d, J = 11.2 Hz, 1H, C*H*H-Bn), 4.79 (d, J = 12.1 Hz, 1H, C*H*H-Bn), 4.71 (d, J = 12.0 Hz, 1H, C*H*H-Bn), 4.64 (d, J = 11.9 Hz, 1H, C*H*H-Bn), 4.58 (d, J = 11.2 Hz, 1H, C*H*H-Bn), 4.49 (d, J = 11.9 Hz, 1H, C*H*H-Bn), 4.44 – 4.33 (m, 2H), 4.16 (dd, J = 9.0, 4.3 Hz, 1H), 3.83 (dd, J = 10.8, 4.2 Hz, 1H), 3.75 – 3.67 (m, 2H), 2.33 (s, 3H, CH_3-STol); ^{13}C NMR (151 MHz, $CDCl_3$) δ 155.16 (*C*=O-Troc), 138.17, 137.99, 137.72, 132.39, 129.99, 129.92, 128.57, 128.52, 128.07, 127.96, 127.92, 127.86, 95.41 (CCl_3), 87.45 (C-1), 75.92, 74.89, 74.81, 73.61, 71.71, 71.17,

68.84, 56.40, 21.20. HRMS (ESI): m/z calcd for $C_{30}H_{32}Cl_4NO_6S^-$ $[M+Cl]^-$ 674.0710, found 674.0712.

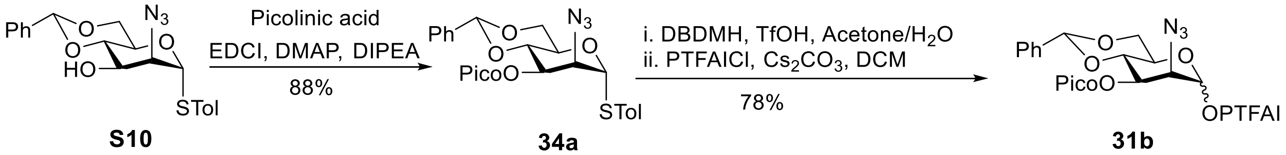

Scheme S9. Synthesis of $ManN_3$ building blocks **34a** and **34b**

p-Tolyl 2-azide-3-*O*-picoloyl-4,6-*O*-benzylidene-2-deoxy-1-thio-*α*-D-mannopyranoside (34a)

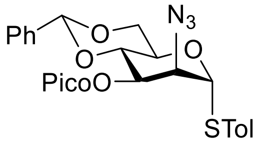

34a

Picolinic acid (93 mg, 0.75 mmol), EDCI (144 mg, 0.75 mmol), DMAP (13 mg, 0.1 mmol), and DIPEA (0.25 mL, 1.5 mmol) were added to a solution of **S10** (200 mg, 0.5 mmol) in CH_2Cl_2 (5 mL) and the resulting mixture was stirred for 2 h at room temperature. TLC analysis showed complete conversion of starting material to compound **34a** (petroleum ether/ethyl acetate = 4/1, R_f = 0.40). The reaction was diluted with CH_2Cl_2 and washed with H_2O and brine. The organic phase was dried (Na_2SO_4), and filtered. The filtrate was concentrated *in vacuo* and purified by silica gel column chromatography (petroleum ether/ethyl acetate = 10/1 to 8/1) to give compound **34a** (224 mg, 88%) as a white amorphous solid. ^{1}H NMR (600 MHz, $CDCl_3$) δ 8.80 – 8.79 (d, J = 4.7, 0.9 Hz, 1H, ArH), 8.17 (d, J = 7.8 Hz, 1H, ArH), 7.85 – 7.82 (m, 1H, ArH), 7.51 – 7.44 (m, 3H, ArH), 7.42 – 7.39 (m, 2H, ArH), 7.36 – 7.30 (m, 3H, ArH), 7.17 (d, J = 7.8 Hz, 2H, ArH), 5.81 (dd, J = 10.2, 3.8 Hz, 1H, H-3), 5.63 (s, 1H, C*H*Ph), 5.49 (d, J = 1.5 Hz, 1H, H-1), 4.65 – 4.47 (m, 2H, H-2, H-5), 4.38 (t, J = 9.8 Hz, 1H, H-4), 4.26 (dd, J = 10.3, 4.9 Hz, 1H, H-6a), 3.88 (t, J = 10.3 Hz, 1H, H-6b), 2.35 (s, 3H, C*H₃*-STol); ^{13}C NMR (151 MHz, $CDCl_3$) δ 164.19 (*C*=O-Pico), 150.31, 147.16, 138.86, 137.11, 137.06, 133.02, 130.28, 129.23, 128.79, 128.35, 127.35, 126.33, 125.75, 102.11 (*C*HPh), 87.27 (C-1), 71.36, 68.49, 65.51, 63.42, 21.28. HRMS (ESI): m/z calcd for $C_{26}H_{25}N_4O_5S^+$ $[M+H]^+$ 505.1540, found 505.1541

N-phenyl-trifluoroacetimidoyl 2-azide-3-O-picoloyl-4,6-O-benzylidene-2-deoxy-1-α/β-D-mannopyranoside (34b)

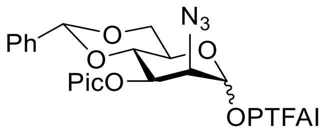

34b

To a solution of compound **34a** (100 mg, 0.18 mmol) in a mixed solvent of acetone/H_2O (v/v, 10/1, 5.5 mL), DBDMH (106 mg, 0.37 mmol) and TfOH (2.5 μL, 0.03 mmol) were added at 0 °C. The reaction mixture was stirred for 0.5 h at 0 °C. TLC analysis showed complete conversion of starting material **34a** to a hemiacetal intermediate (petroleum ether/ethyl acetate = 4/1, R_f =0.30). The reaction was quenched with Et_3N. The organic phase was washed with Na_2SO_3 aqueous, brine, dried (Na_2SO_4), and filtered. The filtrate was concentrated under reduced pressure, and was purified by silica gel column chromatography (petroleum ether/ethyl acetate = 4/1) to give hemiacetal intermediate (75 mg) which was used for next step.

The above hemiacetal intermediate (75 mg, 0.19 mmol) was dissolved in anhydrous CH_2Cl_2 (2 mL), and N-phenyl trifluoroacetimidoyl chloride (46 μL, 0.28 mmol) and Cs_2CO_3 (92 mg, 0.28 mmol) were added. After being stirred for 2 h at room temperature, TLC analysis showed complete conversion of hemiacetal intermediate to compound **34b** (petroleum ether/ethyl acetate = 2/1, R_f = 0.85). The reaction mixture was filtered through celite, and concentrated *in vacuo*. The resulting residue was purified by silica gel column chromatography (petroleum ether/ethyl acetate = 8/1 to 4/1) to give compound **34b**[11] (90 mg, 78% over two steps) as a colorless oil that was immediately used for the glycosylation.

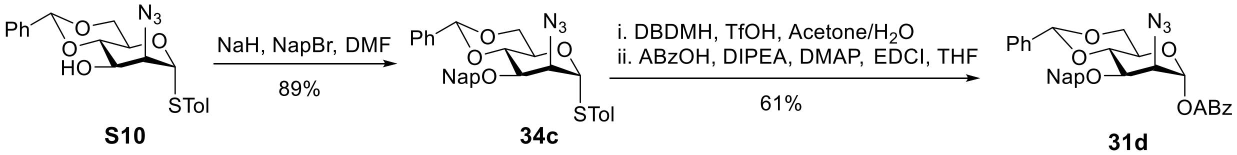

Scheme S10. Synthesis of ManN$_3$ building blocks **34c** and **34d**

***p*-Tolyl 2-azide-3-*O*-naphthylmethyl-4,6-*O*-benzylidene-2-deoxy-1-thio-*α*-D-mannopyranoside (34c)**

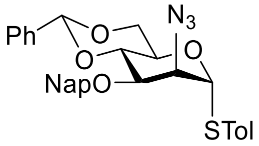

34c

A solution of compound **S10** (200 mg, 0.5 mmol) in anhydrous DMF (5 mL) was added NapBr (133 mg, 0.6 mmol) and NaH (60% dispersion in mineral oil, 24 mg, 0.6 mmol) at 0 ºC. The mixture was stirred for 0.5 h at room temperature under an atmosphere of argon. TLC analysis showed complete conversion of starting material **S10** to a major product **34c** (petroleum ether/ethyl acetate = 8/1, R_f = 0.90). The reaction was quenched with MeOH (10 mL), and the reaction mixture was concentrated *in vacuo*. The resulting residue was dissolved with ethyl acetate, and the mixture was washed with 1 M HCl, saturated aqueous $NaHCO_3$ and brine. The organic phase was dried (Na_2SO_4), and filtered. The filtrate was concentrated *in vacuo* and purified by silica gel column chromatography (petroleum ether/ethyl acetate = 25/1) to give compound **34c** (240 mg, 89%) as light yellow oil. ^{1}H NMR (600 MHz, Acetone-*d*$_6$) *δ* 7.96 – 7.86 (m, 3H, ArH), 7.80 – 7.75 (m, 1H, ArH), 7.59 – 7.53 (m, 3H, ArH), 7.52 – 7.47 (m, 2H, ArH), 7.45 – 7.36 (m, 5H, ArH), 7.17 (d, *J* = 8.1 Hz, 2H, ArH), 5.78 (s, 1H, C*H*Ph), 5.50 (s, 1H, H-1), 5.07 (d, *J* = 12.5 Hz, 1H, C*H*H-Nap), 5.00 (d, *J* = 12.5 Hz, 1H, C*H*H-Nap), 4.64 – 4.51 (m, 1H), 4.28 (dd, *J* = 9.9, 4.6 Hz, 1H), 4.26 – 4.18 (m, 2H), 4.14 (dd, *J* = 10.2, 4.8 Hz, 1H, H-6a), 3.85 (t, *J* = 10.2 Hz, 1H, H-6b), 2.31 (s, 3H, C*H*$_3$-STol); ^{13}C NMR (151 MHz, Acetone-*d*$_6$) *δ* 139.15, 139.05, 136.91, 134.30, 133.99, 133.65, 130.82, 130.20, 129.64, 128.93, 128.85, 128.75, 128.52, 127.20, 126.96, 126.75, 126.51, 102.38 (C*H*Ph), 88.36 (C-1), 79.85, 76.88, 73.28, 68.79, 66.05, 64.57, 21.08. HRMS (ESI): m/z calcd for $C_{31}H_{30}N_3O_4S^+$ $[M+H]^+$ 540.1952, found 540.1952.

2-azide-3-*O*-naphthylmethyl-4,6-*O*-benzylidene-2-deoxy-*α*-D-mannopyranosyl *ortho*-hexynylbenzoate (34d)

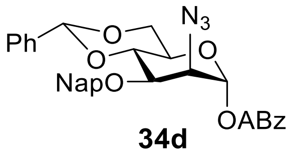

34d

To a solution of compound **34c** (100 mg, 0.18 mmol) in a mixed solvent of acetone/H_2O (v/v, 10/1, 5.5 mL), DBDMH (106 mg, 0.37 mmol) and TfOH (2.5 *μ*L, 0.03 mmol) were added at 0 ºC. The reaction mixture was stirred for 0.5 h at 0 ºC. TLC analysis showed complete conversion of starting material **34c** to a hemiacetal intermediate

(petroleum ether/ethyl acetate = 4/1, R_f =0.30). The reaction was quenched with Et_3N. The organic phase was washed with Na_2SO_3 aqueous, brine, dried (Na_2SO_4), and filtered. The filtrate was concentrated under reduced pressure, and resulting residue was purified by silica gel column chromatography (petroleum ether/ethyl acetate = 4/1) to give hemiacetal intermediate (72 mg) which was used for next step.

A solution of above hemiacetal intermediate (72 mg, 0.17 mmol), *ortho*-hexynylbenzoic acid (50 mg, 0.25 mmol), DMAP (30 mg, 0.25 mmol), EDCI (42 mg, 0.25 mmol) and DIPEA (42 μL, 0.25 mmol) in anhydrous CH_2Cl_2 (5 mL) was stirred at room temperature for 2 h. TLC analysis showed complete conversion of starting material to α-anomer **34d** (petroleum ether/ethyl acetate = 6/1, R_f =0.70) and β-anomer (petroleum ether/ethyl acetate = 6/1, R_f =0.60). The mixture was washed with saturated $NaHCO_3$ solution, H_2O, brine, dried (Na_2SO_4), and filtered. The filtrate was concentrated under reduced pressure, and resulting residue was purified by silica gel column chromatography (petroleum ether/ethyl acetate = 15/1 to 12/1) to α-anomer **34d** (62 mg, 61% over two steps) as a colorless oil. ^{1}H NMR (600 MHz, $CDCl_3$) δ 7.86 – 7.78 (m, 4H, ArH), 7.68 (d, *J* = 7.7 Hz, 1H, ArH), 7.58 – 7.37 (m, 10H, ArH), 7.27 – 7.21 (m, 1H, ArH), 6.32 (d, *J* = 1.9 Hz, 1H, H-1), 5.68 (s, 1H, C*H*Ph), 5.08 (d, *J* = 12.3 Hz, 1H, CH*H*-Nap), 4.94 (d, *J* = 12.3 Hz, 1H, CH*H*-Nap), 4.37 (dd, *J* = 10.2, 3.7 Hz, 1H, H-6a), 4.33 – 4.26 (m, 2H), 4.16 – 4.09 (m, 2H), 3.86 (t, *J* = 10.4 Hz, 1H, H-6a), 2.48 – 2.40 (m, 1H), 2.37 – 2.26 (m, 1H), 1.54 – 1.43 (m, 2H), 1.36 – 1.26 (m, 2H), 0.84 (t, *J* = 7.4 Hz, 3H). ^{13}C NMR (151 MHz, $CDCl_3$) δ 164.16 (*C*=O-ABz), 140.40, 137.35, 135.20, 133.30, 133.11, 132.48, 130.92, 129.94, 129.05, 128.36, 128.29, 128.01, 127.74, 127.41, 126.51, 126.21, 126.13, 126.07, 125.52, 125.00, 101.75, 97.18, 93.52, 79.93, 78.62, 75.35, 73.39, 68.52, 66.56, 61.93, 30.79, 22.04, 19.69, 13.69. HRMS (ESI): m/z calcd for $C_{37}H_{35}N_3NaO_6^+$ $[M+Na]^+$ 640.2418, found 640.2417.

***β*-anomer 34e:** ^{1}H NMR (600 MHz, $CDCl_3$) δ 8.04 (dd, *J* = 7.9, 1.3 Hz, 1H, ArH), 7.87 – 7.83 (m, 3H, ArH), 7.81 – 7.75 (m, 1H, ArH), 7.57 – 7.34 (m, 11H, ArH), 5.99 (d, *J* = 1.6 Hz, 1H, H-1), 5.66 (s, 1H, C*H*Ph), 5.07 (d, *J* = 12.4 Hz, 1H, CH*H*-Nap), 4.95 (d, *J* = 12.4 Hz, 1H, CH*H*-Nap), 4.37 (dd, *J* = 10.3, 4.9 Hz, 1H, H-6b), 4.22 – 4.14 (m, 2H, H-4, H-2), 3.97 (dd, *J* = 9.6, 3.6 Hz, 1H, H-3), 3.89 (t, *J* = 10.3 Hz, 1H, H-6a), 3.58 – 3.54 (m, 1H, H-5), 2.50 (t, *J* = 7.2 Hz, 2H), 1.71 – 1.61 (m, 2H), 1.56 – 1.46 (m, 2H), 0.96 (t, *J* = 7.3 Hz, 3H, C*H*$_3$-ABz). ^{13}C NMR (151 MHz, $CDCl_3$) δ 163.38 (*C*=O-ABz), 137.30, 135.07, 134.76, 133.36, 133.25, 132.72, 130.96, 129.52, 129.22, 128.54, 128.42, 128.10, 127.83, 127.35, 126.80, 126.36, 126.24, 125.93, 125.64, 101.92, 97.46, 92.44, 79.03, 78.36, 76.67, 73.29, 68.36, 68.28, 63.03, 30.78, 22.19, 19.66, 13.79. HRMS (ESI): m/z calcd for $C_{37}H_{35}N_3NaO_6^+$ $[M+Na]^+$ 640.2418, found 640.2417.

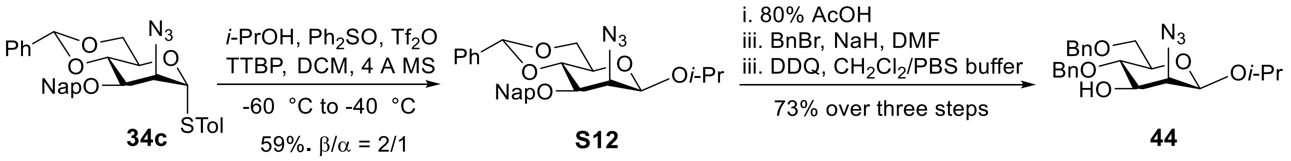

Scheme S11. Synthesis of $ManN_3$ building block **44**

Isopropyl 2-azide-3-*O*-naphthylmethyl-4,6-*O*-benzylidene-2-deoxy-*β*-D-mannopyranoside (S12)

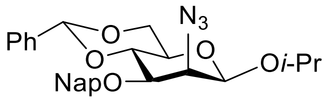

S12

A mixture of ManN donor **34c** (1.0 g, 1.8 mmol), Ph_2SO (0.45 g, 2.2 mmol), and TTBP (0.48 g, 4.6 mmol) was co-evaporated three times with PhMe and dissolved in anhydrous CH_2Cl_2 (10 mL). The mixture was added freshly activated 4 Å molecular sieves and stirred for 15 min at room temperature under an atmosphere of argon. Then, the mixture was cooled to –60 ºC, and the Tf_2O (0.37 mL, 2.2 mmol) was added. After being stirred at this temperature for 5 min, TLC analysis showed complete conversion of **34c**. Then, the anhydrous *i*-PrOH (0.22 mL, 2.8 mmol) was added. The reaction mixture was warmed to –40 ºC over 4 h, TLC analysis showed complete conversion of donor to **S12** (petroleum ether/ethyl acetate = 5/1, R_f =0.60) and *α*-isomer (petroleum ether/ethyl acetate = 5/1, R_f =0.75). The reaction was quenched by the addition of triethylamine and filtered through celite. The filtrate was concentrated under reduced pressure and purified by silica gel column chromatography to afford compound **S12** (520 mg, 59%) as a colorless oil. ^1H NMR (600 MHz, $CDCl_3$) δ 7.89 – 7.81 (m, 3H, ArH), 7.80 – 7.74 (m, 1H, ArH), 7.57 – 7.45 (m, 5H, ArH), 7.45 – 7.37 (m, 3H, ArH), 5.63 (s, 1H, C*H*Ph), 5.03 (d, *J* = 12.7 Hz, 1H, C*H*H-Nap), 4.94 (d, *J* = 12.7 Hz, 1H, C*H*H-Nap), 4.63 (d, *J* = 1.4 Hz, 1H, H-1), 4.31 (dd, *J* = 10.0, 4.9 Hz, 1H, H-6a), 4.08 (t, *J* = 9.9 Hz, 1H, H-6b), 4.04 – 3.98 (m, 1H, OC*H*-Pr), 3.96 (dd, *J* = 3.7, 1.4 Hz, 1H, H-2), 3.90 (t, *J* = 10.3 Hz, 1H, H-4), 3.78 (dd, *J* = 9.6, 3.7 Hz, 1H, H-3), 3.34 – 3.30 (m, 1H, H-5), 1.27 (d, *J* = 6.3 Hz, 3H, CH_3-*i*Pr), 1.17 (d, *J* = 6.1 Hz, 3H, CH_3-*i*Pr); ^{13}C NMR (151 MHz, $CDCl_3$) δ 137.52, 135.37, 133.35, 133.20, 129.15, 128.46, 128.41, 128.08, 127.83, 126.75, 126.31, 126.23, 126.14, 125.75, 101.76 (*C*HPh), 98.47 (C-1), 78.77, 76.38, 72.92, 71.83, 68.65, 67.43, 64.13, 23.47 (*C*H3-*i*Pr), 21.70 (*C*H3-*i*Pr). HRMS (ESI): m/z calcd for $C_{27}H_{30}N_3O_5^+$ [M+H]$^+$ 476.2180, found 476.2183.

Isopropyl 2-azide-4,6-di-*O*-benzyl-2-deoxy-*β*-D-mannopyranoside (44)

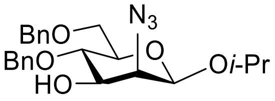

44

The compound **S12** (520 mg, 1.1 mmol) was treated with 80% AcOH in water (10 mL) and the mixture was heated to 80 ºC for 2 h. TLC analysis showed complete conversion of starting material **S12** to a diol intermediate (petroleum ether/ethyl acetate = 3/1, R_f = 0.30). The reaction mixture was cooled to room temperature and concentrated under reduced pressure. The resulting residue was co-evaporated three times with PhMe to give diol intermediate for the next step.

A solution of above diol intermediate in anhydrous DMF (5 mL) was added BnBr (0.33 mL, 2.7 mmol) and NaH (60% dispersion in mineral oil, 110 mg, 2.7 mmol) at 0 ºC. The mixture was stirred for 4 h at room temperature under an atmosphere of argon. TLC analysis showed complete conversion to a major benzyl protected product (petroleum ether/ethyl acetate = 15/1, R_f = 0.40). The reaction was quenched with MeOH, and the reaction mixture was concentrated *in vacuo*. The resulting residue was dissolved with ethyl acetate and washed with 1 M HCl, saturated aqueous $NaHCO_3$ and brine. The organic phase was dried (Na_2SO_4), and filtered. The filtrate was concentrated *in vacuo* and purified by silica gel column chromatography (petroleum ether/ethyl acetate = 25/1 to 15/1) to give 4,6-di-*O*-benzyl ManN intermediate (560 mg) as a colorless oil.

To solution of above intermediate (560 mg, 0.93 mmol) in a mixed solvent of CH_2Cl_2/0.1 M PBS buffer (v/v, 10/1, 11 mL) was added DDQ (318 mg, 1.4 mmol) at 0 ºC. After being stirred at room temperature for 2.5 h, the reaction was diluted with ethyl acetate, and washed with saturated aqueous $NaHCO_3$ and brine. The organic phase was dried (Na_2SO_4), and filtered. The filtrate was concentrated under reduced pressure to give crude product. The crude product was purified by silica gel column chromatography (petroleum ether/ethyl acetate = 10/1 to 8/1) to give compound **44** (345 mg, 73% over two steps) as a colorless oil. ^{1}H NMR (600 MHz, $CDCl_3$) δ 7.43 – 7.16 (m, 10H, ArH), 4.77 (d, J = 11.1 Hz, 1H, C*H*H-Bn), 4.65 (d, J = 12.2 Hz, 1H, C*H*H-Bn), 4.63 (d, J = 1.4 Hz, 1H, H-1), 4.57 (d, J = 11.7 Hz, 2H, 2 × CH*H*-Bn), 4.07 – 4.01 (m, 1H, OC*H*-*i*Pr), 3.82 (dd, J = 3.9, 1.4 Hz, 1H, H-2), 3.76 (dd, J = 10.9, 2.2 Hz, 1H, H-6a), 3.73 – 3.67 (m, 2H, H-3, H-6b), 3.53 (t, J = 9.3 Hz, 1H, H-4), 3.39 – 3.36 (m, 1H, H-5), 1.29 (d, J = 6.2 Hz, 3H, C*H*$_3$-*i*Pr), 1.18 (d, J = 6.2 Hz, 3H, C*H*$_3$-*i*Pr). ^{13}C NMR (151 MHz, $CDCl_3$) δ 138.28, 138.08, 134.25, 129.76, 128.54, 128.39, 128.03, 127.97, 127.89, 127.65, 98.13 (C-1), 76.18, 75.43, 74.93, 73.59, 73.39, 71.31, 69.25, 64.92, 23.48 (*C*H$_3$-*i*Pr), 21.67 (*C*H$_3$-*i*Pr). HRMS (ESI): m/z calcd for $C_{23}H_{29}N_3NaO_5^+$ $[M+Na]^+$

450.1999, found 450.1996.

4. Synthesis of pentasaccharide 1

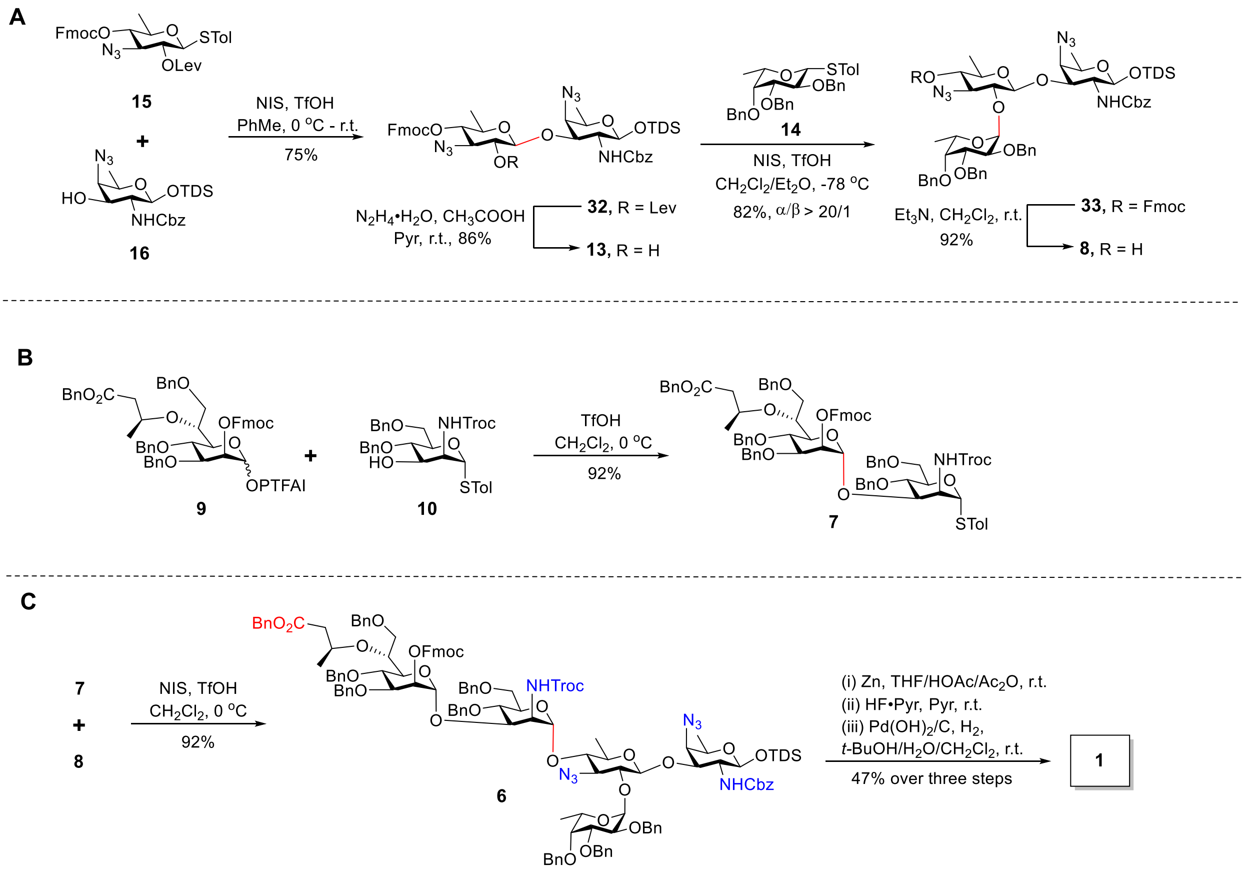

Scheme S12. Synthesis of pentasaccharide **1**

Table S7. Optimized conditions for the coupling of thioglycosyl donor **15** and acceptor **16**

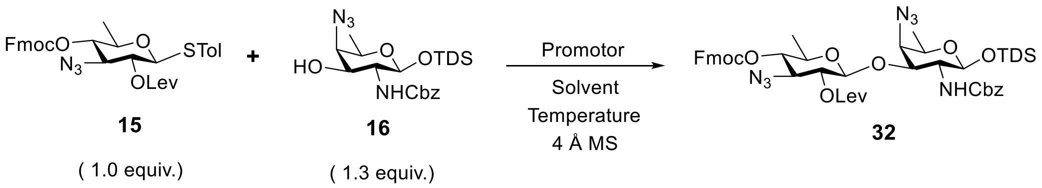

Entry	Promoters	T (°C)	Solvent	Results
S1	NIS (1.5 eq.)/TMSOTf (0.2 eq.)	-20 to r.t.	DCM	No reaction
S2	NIS (1.5 eq.)/TfOH (0.2 eq.)	-20 to r.t.	DCM	<50% (Donor was hydrolysed)
S3	NIS (1.5 eq.)/TfOH (0.2 eq.)	-20 to r.t.	PhMe	65% (Donor was partly activated)
S4	NIS (2.0 eq.)/TfOH (0.2 eq.)	0 to r.t.	PhMe	61% (Donor was partly activated)
S5	NIS (2.0 eq.)/TfOH (0.4 eq.)	0 to r.t.	PhMe	75%

Dimethylthexylsilyl [2-*O*-levulinoyl-3-azide-4-*O*-fluorenylmethoxycarbonyl]-3,6-di-deoxy-*β*-D-glucopyranosyl-(1→3)-2-*N*-benzyloxycarbonyl-4-azide-2,4,6-tri-deoxy-*β*-D-galactopyranoside (32)

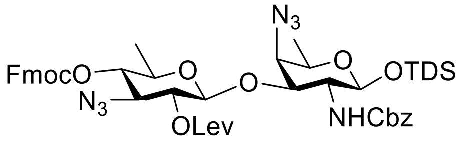

32

Thioglycoside **15** (400 mg, 0.65 mmol) and acceptor **16** (390 mg, 0.84 mmol) were co-evaporated with PhMe. Then, the mixture was dissolved in anhydrous PhMe (50 mL) and added freshly activated 4 Å molecular sieves. The mixture was stirred at room temperature for 15 min under an atmosphere of argon and then cooled to 0 °C. After being stirred at 0 °C for 15 min, NIS (292 mg, 1.3 mmol) and TfOH (22 μL, 0.26 mmol) were added. The reaction was gradually warmed to room temperature and stirred at room temperature for 2 h. TLC analysis showed complete conversion of donor to a major product **32** (petroleum ether/ethyl acetate = 2/1, R_f = 0.80). The reaction was quenched by the addition of pyridine. Then, the reaction was diluted with CH_2Cl_2, filtered and washed with aqueous $Na_2S_2O_3$ and brine. The organic phase was dried (Na_2SO_4), and filtered. The filtrate was concentrated *in vacuo* and purified by silica gel column chromatography (petroleum ether/ethyl acetate/CH_2Cl_2 = 4/1/1) to give compound **32** (465 mg, 75%) as a colorless oil. ^{1}H NMR (600 MHz, $CDCl_3$) δ 7.78 (dd, *J* = 7.5, 2.8 Hz, 2H, ArH), 7.62 (d, *J* = 7.5 Hz, 2H, ArH), 7.50 – 7.30 (m, 8H, ArH), 7.08 (d, *J* = 7.0 Hz, 1H, ArH), 5.30 (d, *J* = 12.3 Hz, 1H, C*H*H-Cbz), 5.13 (d, *J* = 8.0 Hz, 1H, H-1-AAT), 4.90 (d, *J* = 12.3 Hz, 1H, C*H*H-Cbz), 4.83 (dd, *J* = 10.4, 7.8 Hz, 1H, H-2-ADG), 4.66 (dd, *J* = 10.8, 3.9 Hz, 1H, H-3-AAT), 4.60 – 4.50 (m, 2H, CH_2-Fmoc), 4.38 (t, *J* = 9.8 Hz, 1H, H-4-ADG), 4.29 (t, *J* = 7.0 Hz, 1H, C*H*-Fmoc), 4.25 (d, *J* = 7.9 Hz, 1H, H-1-ADG), 3.72 (d, *J* = 4.1 Hz, 1H, H-4-AAT), 3.64 (q, *J* = 6.1 Hz, 1H, H-5-AAT), 3.43 – 3.37 (m, 1H, H-5-ADG), 3.36 (t, *J* = 10.1 Hz, 1H, H-3-AAT), 3.09 – 2.97 (m, 2H, H-2-AAT, C*H*H-Lev), 2.88 – 2.73 (m, 1H, C*H*H-Lev), 2.49 – 2.39 (m, 1H, C*H*H-Lev), 2.31 – 2.21 (m, 1H, C*H*H-Lev), 2.18 (s, 3H, CH_3-Lev), 1.68 – 1.56 (m, 1H, C*H*(CH_3)$_2$-TDS), 1.26 (d, *J* = 6.3 Hz, 3H, CH_3-AAT), 1.17 (d, *J* = 6.1 Hz, 3H, CH_3-ADG), 0.87 (t, *J* = 6.4 Hz, 6H, CH(CH_3)$_2$-TDS), 0.84 (d, *J* = 3.7 Hz, 6H, C(CH_3)$_2$-TDS), 0.13 (s, 3H, SiCH_3-TDS), 0.10 (s, 3H, SiCH_3-TDS); ^{13}C NMR (151 MHz, $CDCl_3$) δ 207.81 (*C*=O-Lev), 170.62 (*C*=O-Lev), 155.91 (*C*=O-Cbz), 154.23 (*C*=O-Fmoc), 143.16, 142.99, 141.37, 137.11, 128.68, 128.51, 128.26, 127.97, 127.22, 127.21, 125.04, 125.00, 120.14, 120.13, 101.99 (C-1-ADG, $J_{C1\text{-}H1}$ = 161 Hz), 93.88 (C-1-AAT, $J_{C1\text{-}H1}$ = 162 Hz), 76.92, 71.88, 70.34, 70.22, 68.39, 66.05, 65.70, 63.66, 56.44, 46.79, 37.31 (*C*H_2-Lev), 34.12 (*C*H(CH_3)$_2$-TDS), 29.90 (*C*H_3-Lev), 27.02 (*C*H_2-Lev), 24.80 (*C*(CH_3)$_2$-TDS), 20.15 (C(*C*H_3)$_2$-TDS), 19.99 (C(*C*H_3)$_2$-TDS), 18.61 (CH(*C*H_3)$_2$-TDS), 18.53 (CH(*C*H_3)$_2$-TDS), 17.68 (C-6-AAT), 17.25 (C-6-ADG), -1.94 (Si*C*H_3-TDS), -3.63 (Si*C*H_3-

TDS). HRMS (ESI): m/z calcd for $C_{48}H_{61}N_7NaO_{12}Si^+$ $[M+Na]^+$ 978.4040, found 978.4043.

Dimethylthexylsilyl [3-azide-4-*O*-fluorenylmethoxycarbonyl-3,6-di-deoxy-*β*-D-glucopyranosyl]-(1→3)-2-*N*-benzyloxycarbonyl-4-azide-2,4,6-tri-deoxy-*β*-D-galactopyranoside (13)

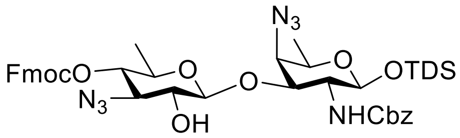

13

To solution of a mixture of **32** (428 mg, 0.45 mmol) in a mixed solvent of pyridine (3 mL) and HOAc (1 mL) was added $N_2H_4{\cdot}H_2O$ (85%, 40 μL, 0.67 mmol) at 0 °C. The mixture was stirred at room temperature for 4.5 h. TLC analysis showed complete conversion of starting material **32** to compound **13** (PhMe/ethyl acetate = 20/1, R_f = 0.35). The reaction mixture was quenched with acetone and concentrated under reduced pressure to give crude product. The crude product was purified by silica gel column chromatography (petroleum ether/ethyl acetate = 8/1) to give compound **13** (370 mg, 86%) as a white amorphous solid. ^{1}H NMR (600 MHz, $CDCl_3$) δ 7.78 (dd, *J* = 7.5, 2.8 Hz, 2H, ArH), 7.62 (d, *J* = 7.5 Hz, 2H, ArH), 7.45 – 7.40 (m, 2H, ArH), 7.40 – 7.31 (m, 7H, ArH), 5.14 (d, *J* = 12.2 Hz, 1H, C*H*H-Cbz), 5.04 (d, *J* = 12.2 Hz, 1H, C*H*H-Cbz), 4.96 (d, *J* = 7.7 Hz, 1H, N*H*), 4.80 – 4.70 (m, 1H, H-1-AAT), 4.57 (dd, *J* = 10.6, 7.0 Hz, 1H, CH*H*-Fmoc), 4.51 (dd, *J* = 10.6, 7.1 Hz, 1H, CH*H*-Fmoc), 4.37 (t, *J* = 9.7 Hz, 1H, H-4-ADG), 4.34 – 4.24 (m, 3H, H-1-ADG, C*H*-Fmoc, H-3-AAT), 3.77 (d, *J* = 4.5 Hz, 1H, H-4-AAT), 3.62 (q, *J* = 6.7 Hz, 1H, H-5-AAT), 3.50 – 3.39 (m, 4H, H-2-ADG, H-3-ADG, H-5-ADG, H-2-AAT), 1.64 – 1.55 (m, 1H, C*H*$(CH_3)_2$-TDS), 1.30 (d, *J* = 6.4 Hz, 3H, C*H*$_3$-AAT), 1.18 (d, *J* = 6.2 Hz, 3H, C*H*$_3$-ADG), 0.84 (dd, *J* = 6.9, 4.4 Hz, 6H, CH(C*H*$_3$)$_2$-TDS), 0.81 (d, *J* = 2.9 Hz, 6H, C(C*H*$_3$)$_2$-TDS), 0.14 (s, 3H, SiC*H*$_3$-TDS), 0.09 (s, 3H, SiC*H*$_3$-TDS); ^{13}C NMR (151 MHz, $CDCl_3$) δ 156.62 (*C*=O-Cbz), 154.53 (*C*=O-Fmoc), 143.32, 143.14, 141.48, 136.31, 128.74, 128.69, 128.50, 128.08, 127.35, 127.33, 125.19, 125.16, 120.25, 120.23, 104.26 (C-1-ADG), 95.19 (C-1-AAT), 76.95, 73.49, 70.72, 70.32, 68.92, 67.13, 65.54, 65.23, 56.34, 46.91 (CH-Fmoc), 34.13 (*C*H$(CH_3)_2$-TDS), 24.92 (*C*$(CH_3)_2$-TDS), 20.22 (C(*C*H$_3$)$_2$-TDS), 20.08 (C(*C*H$_3$)$_2$-TDS), 18.66 (CH(*C*H$_3$)$_2$-TDS), 18.57 (CH(*C*H$_3$)$_2$-TDS), 17.69 (C-6-AAT), 17.46 (C-6-ADG), -1.71 (Si*C*H$_3$-TDS), -3.36 (Si*C*H$_3$-TDS). HRMS (ESI): m/z calcd for $C_{43}H_{55}N_7NaO_{10}Si^+$ $[M+Na]^+$ 880.3672, found 880.3672.

Dimethylthexylsilyl [2,3,4-tri-*O*-benzyl-*α*-L-fucopyranosyl]-(1→2)-[3-azide-4-*O*-fluorenylmethoxycarbonyl-

3,6-di-deoxy-*β*-D-glucopyranosyl]-(1→3)- 2-*N*-benzyloxycarbonyl-4-azide-2,4,6-tri-deoxy-*β*-D-galactopyranoside (33)

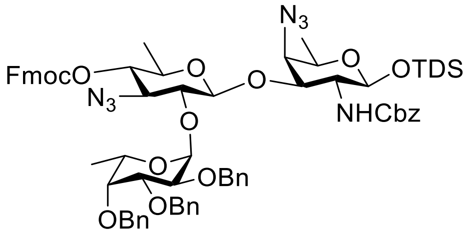

33

Thioglycoside **14**[12] (346 mg, 0.64 mmol) and acceptor **13** (275 mg, 0.32 mmol) were co-evaporated three times with PhMe. Then, the mixture was dissolved in mixed solvent of anhydrous CH_2Cl_2/Et_2O (v/v, 2/1, 3 mL) and added freshly activated 4 Å molecular sieves. The mixture was stirred at room temperature for 15 min under an atmosphere of argon and then cooled to -70 °C. After being stirred at -70 °C for 15 min, NIS (144 mg, 0.64 mmol) and TfOH (5.6 μL, 0.064 mmol) were added. After being stirred at this temperature for 2 h, TLC analysis showed complete conversion of acceptor to a major product **33** (petroleum ether/ethyl acetate = 6/1, R_f = 0.70). The reaction was quenched by the addition of pyridine and filtered. The filtrate was concentrated *in vacuo* and purified by silica gel column chromatography (petroleum ether/ethyl acetate = 20/1-12/1) to give compound **33** (333 mg, 82%) as a yellow oil. ^{1}H NMR (600 MHz, $CDCl_3$) δ 7.81 (d, J = 7.1 Hz, 2H, ArH), 7.66 (d, J = 7.5 Hz, 2H, ArH), 7.48 – 7.42 (m, 2H, ArH), 7.42 – 7.25 (m, 22H, ArH), 6.01 (d, J = 6.8 Hz, 1H, N*H*), 5.59 (d, J = 3.0 Hz, 1H, H-1-Fuc), 5.28 (d, J = 12.1 Hz, 1H, C*H*H-Cbz), 5.21 (d, J = 7.9 Hz, 1H, H-1-AAT), 4.98 (d, J = 11.4 Hz, 1H, C*H*H-Bn), 4.90 – 4.76 (m, 4H, 3 × C*H*H-Bn, C*H*H-Cbz), 4.73 (d, J = 11.7 Hz, 1H, C*H*H-Bn), 4.68 (d, J = 11.5 Hz, 1H, C*H*H-Bn), 4.59 (dd, J = 10.6, 7.1 Hz, 1H, C*H*H-Fmoc), 4.55 – 4.48(m, 2H, C*H*H-Fmoc, H-3-AAT), 4.34 (t, J = 7.1 Hz, 1H, C*H*-Fmoc), 4.28 (t, J = 9.8 Hz, 1H, H-4-ADG), 4.15 – 4.08 (m, 4H, H-2-Fuc, H-5-Fuc, H-1-ADG), 3.72 (dd, J = 3.8, 1.2 Hz, 1H, H-4-AAT), 3.70 (d, J = 2.0 Hz, 1H, H-4-Fuc), 3.65 – 3.61 (m, 2H, H-2-ADG, H-5-AAT), 3.19 – 3.15 (m, 1H, H-5-ADG), 3.13 – 3.05 (m, 2H, H-2-AAT, H-3-ADG), 1.65 – 1.55 (m, 1H, C*H*(CH_3)$_2$-TDS), 1.28 (d, J = 6.3 Hz, 3H, CH_3-AAT), 1.21 (d, J = 6.4 Hz, 3H, CH_3-Fuc), 1.14 (d, J = 6.1 Hz, 3H, CH_3-ADG), 0.86 (dd, J = 6.8, 3.5 Hz, 6H, CH(CH_3)$_2$-TDS), 0.83 (d, J = 2.3 Hz, 6H, C(CH_3)$_2$-TDS), 0.13 (s, 3H, SiCH_3-TDS), 0.10 (s, 3H, SiCH_3-TDS); ^{13}C NMR (151 MHz, $CDCl_3$) δ 155.95 (C=O-Cbz), 154.49 (C=O-Fmoc), 143.36, 143.21, 141.51, 141.49, 139.06, 138.51, 138.48, 136.92, 128.82, 128.78, 128.71, 128.42, 128.38, 128.36, 128.10, 128.08, 127.81, 127.55, 127.52, 127.49, 127.34, 125.16, 125.14, 120.26, 120.24, 103.88 (C-1-ADG, $J_{C1\text{-}H1}$ = 163 Hz), 97.65 (C-1-Fuc, $J_{C1\text{-}H1}$ = 171 Hz), 94.16 (C-1-AAT, $J_{C1\text{-}H1}$ = 162 Hz), 79.20, 77.62, 77.60, 77.08, 76.59, 76.44, 74.94, 73.26, 73.24, 70.42, 70.19, 68.64, 68.56, 66.28, 65.79, 65.60, 57.00, 46.91 (*C*H-Fmoc), 34.12 (*C*H(CH_3)$_2$-TDS), 24.91 (*C*(CH_3)$_2$-

TDS), 20.17 (C(CH_3)$_2$-TDS), 20.11 (C(CH_3)$_2$-TDS), 18.66 (CH(CH_3)$_2$-TDS), 18.62 (CH(CH_3)$_2$-TDS), 17.70 (C-6-AAT), 17.38 (C-6-Fuc), 16.51 (C-6-ADG), -1.80 (Si*C*H$_3$-TDS), -3.54 (Si*C*H$_3$-TDS). HRMS (ESI): m/z calcd for $C_{70}H_{83}N_7NaO_{14}Si^+$ $[M+Na]^+$ 1296.5659, found 1296.5654.

Dimethylthexylsilyl [2,3,4-tri-*O*-benzyl-*α*-L-fucopyranosyl]-(1→2)-[3-azide-3,6-di-deoxy-*β*-D-glucopyranosyl]-(1→3)-2-*N*-benzyloxycarbonyl-4-azide-2,4,6-tri-deoxy-*β*-D-galactopyranoside (8)

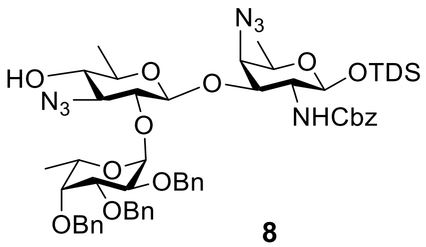

8

To solution of a mixture of **33** (333 mg, 0.26 mmol) in CH_2Cl_2 (3 mL) was added Et_3N (0.5 mL) at 0 °C. The mixture was stirred at room temperature overnight. TLC analysis showed complete conversion of starting material **33** to compound **8** (petroleum ether /ethyl acetate = 6/1, R_f = 0.40). The reaction concentrated under reduced pressure to give crude product. The crude product was purified by silica gel column chromatography (petroleum ether/ethyl acetate/CH_2Cl_2= 4/1/1) to give compound **8** (252 mg, 92%) as a colorless oil. ^{1}H NMR (600 MHz, $CDCl_3$) δ 7.40 – 7.23 (m, 20H, ArH), 6.03 (d, J = 6.7 Hz, 1H, N*H*), 5.61 (d, J = 2.0 Hz, 1H, H-1-Fuc), 5.25 (d, J = 12.3 Hz, 1H, C*H*H-Cbz), 5.21 (d, J = 7.9 Hz, 1H, H-1-AAT), 4.97 (d, J = 11.6 Hz, 1H, C*H*H-Bn), 4.88 – 4.81 (m, 2H, C*H*H-Bn, C*H*H-Cbz), 4.78 (s, 2H, C*H$_2$*-Bn), 4.73 (d, J = 11.8 Hz, 1H, C*H*H-Bn), 4.66 (d, J = 12.5 Hz, 1H, C*H*H-Bn), 4.46 (dd, J = 10.7, 3.9 Hz, 1H, H-3-AAT), 4.13 – 4.06 (m, 4H, H-2-Fuc, H-1-ADG, H-2-Fuc, H-5-Fuc), 3.72 (dd, J = 3.9, 1.3 Hz, 1H, H-4-AAT), 3.68 (s, 1H, H-4-Fuc), 3.64 – 3.58 (m, 1H, H-5-AAT), 3.55 (dd, J = 9.6, 7.7 Hz, 1H, H-2-ADG), 3.21 – 3.03 (m, 2H, H-5-ADG, H-2-AAT), 2.97 (t, J = 9.6 Hz, 1H, H-3-ADG), 2.92 (t, J = 9.3 Hz, 1H, H-4-ADG), 1.66 – 1.55 (m, 1H, C*H*(CH_3)$_2$-TDS), 1.26 (d, J = 6.3 Hz, 3H, C*H$_3$*-AAT), 1.23 (d, J = 6.1 Hz, 3H, C*H$_3$*-ADG), 1.19 (d, J = 6.4 Hz, 3H, C*H$_3$*-Fuc), 0.85 (dd, J = 6.9, 3.6 Hz, 6H, CH(C*H$_3$*)$_2$-TDS), 0.81 (d, J = 2.7 Hz, 6H, C(C*H$_3$*)$_2$-TDS), 0.12 (s, 3H, SiC*H$_3$*-TDS), 0.08 (s, 3H, SiC*H$_3$*-TDS); ^{13}C NMR (151 MHz, D_2O) δ 155.98 (*C*=O-Cbz), 139.13, 138.69, 138.52, 137.01, 128.78, 128.72, 128.65, 128.59, 128.46, 128.43, 128.37, 128.30, 127.81, 127.77, 127.53, 127.52, 127.47, 104.06 (C-1-ADG), 97.66 (C-1-Fuc), 94.23 (C-1-AAT), 79.11, 78.24, 77.60, 76.85, 76.60, 74.98, 73.84, 73.35, 73.13, 72.03, 68.62, 68.58, 66.23, 65.83, 57.00, 34.14 (*C*H(CH_3)$_2$-TDS), 24.94 (*C*(CH_3)$_2$-TDS), 20.19 (C(*C*H$_3$)$_2$-TDS), 20.13 (C(*C*H$_3$)$_2$-TDS), 18.67 (CH(*C*H$_3$)$_2$-TDS), 18.63 (CH(*C*H$_3$)$_2$-TDS),

17.77 (C-6-AAT), 17.72 (C-6-Fuc), 16.56 (C-6-ADG), -1.80 (Si*C*H$_3$-TDS), -3.51 (Si*C*H$_3$-TDS). HRMS (ESI): m/z calcd for $C_{55}H_{73}N_7NaO_{12}Si^+$ [M+Na]$^+$ 1074.4977, found 1074.4979.

***p*-Tolyl 2-*O*-fluorenylmethoxycarbonyl-3,4,7-tri-*O*-benzyl-6-*O*-[benzyl 3(*S*)-butanoate]-D-*glycero*-*α*-D-*manno*-heptopyranosyl-(1→3)-2-*N*-(2,2,2-trichloroethyloxy)carbonylamino-2-deoxy-4,6-di-*O*-benzyl-1-thio-*α*-D-mannopyranoside (7)**

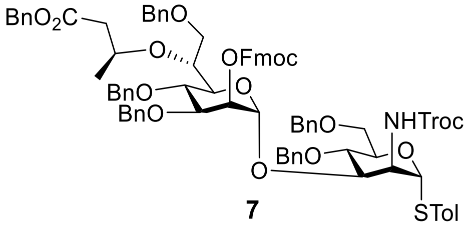

7

The Hep-OPTFAI donor **9** (119 mg, 0.12 mmol) and ManNHTroc acceptor **10** (95 mg, 0.15 mmol) were co-evaporated three times with PhMe. Then, the mixture was dissolved in anhydrous CH_2Cl_2 (5 mL) and added freshly activated 4 Å molecular sieves. The mixture was stirred at room temperature for 15 min under an atmosphere of argon and then cooled to 0 °C. After being stirred at 0 °C for 15 min, TfOH (2.1 *μ*L, 0.024 mmol) was added. After being stirred at this temperature for 30 min, TLC analysis showed complete conversion of donor to a major product **7** (petroleum ether/ethyl acetate = 3/1, R_f = 0.80). The reaction was quenched by the addition of pyridine and filtered. The filtrate was concentrated *in vacuo* and purified by silica gel column chromatography (petroleum ether/ethyl acetate/CH_2Cl_2 = 12/1/1) to give compound **7** (160 mg, 92%) as a colorless oil. ^{1}H NMR (800 MHz, $CDCl_3$) *δ* 7.75 (d, *J* = 7.6 Hz, 2H, ArH), 7.62 – 7.52 (m, 2H, ArH), 7.43 – 7.14 (m, 36H, ArH), 7.02 (d, *J* = 8.2 Hz, 2H, ArH), 5.72 (s, 1H, N*H*), 5.39 (d, *J* = 2.5 Hz, 1H, H-1-ManN), 5.23 – 5.16 (m, 2H, H-1-Hep, H-2-Hep), 5.06 (s, 2H, C*H$_2$*-Bn), 4.83 (d, *J* = 11.1 Hz, 1H, C*H*H-Bn), 4.72 (d, *J* = 10.8 Hz, 1H, C*H*H-Bn), 4.69 – 4.65 (m, 3H, C*H*H-Bn, C*H$_2$*-Troc), 4.66 – 4.59 (m, 2H, 2 × C*H*H-Bn), 4.56 – 4.40 (m, 6H, H-2-ManN, 5 × C*H*H-Bn), 4.36 (dd, *J* = 10.5, 7.4 Hz, 1H, C*H*H-Fmoc), 4.34 – 4.31 (m, 1H, H-5-ManN), 4.26 – 4.19 (m, 3H, C*H*H-Fmoc, H-3-3Hb, H-3-ManN), 4.15 (t, *J* = 7.8 Hz, 1H, C*H*-Fmoc), 4.05 – 3.92 (m, 3H, H-4-Hep, H-6-Hep, H-5-Hep), 3.93 (dd, *J* = 8.6, 3.2 Hz, 1H, H-3-Hep), 3.81 – 3.74 (m, 2H, H-4-ManN, H-6a-ManN), 3.72 (dd, *J* = 10.2, 4.9 Hz, 1H, H-7a-ManN), 3.64 – 3.56 (m, 2H, H-7b-Hep, H-6b-ManN), 2.98 – 2.85 (m, 1H, H-2-3Hb), 2.50 (dd, *J* = 15.1, 8.2 Hz, 1H, H-2-3Hb), 2.28 (s, 3H, C*H$_3$*-STol), 1.30 (d, *J* = 6.2 Hz, 3H, H-4-3Hb); ^{13}C NMR (201 MHz, $CDCl_3$) *δ* 171.54 (*C*=O-3Hb), 154.79 (*C*=O-Fmoc), 154.21 (*C*=O-Troc), 143.64, 143.33, 141.39, 141.32, 138.69, 138.67, 138.07, 137.88, 137.85, 137.68, 136.26, 132.38, 132.33, 130.02, 129.89, 128.56, 128.53, 128.51, 128.41, 128.36, 128.33, 128.24, 128.21, 128.13, 128.11,

128.07, 127.99, 127.97, 127.90, 127.78, 127.72, 127.67, 127.61, 127.48, 127.46, 127.42, 127.29, 127.28, 125.47, 125.29, 120.15, 120.12, 120.09, 99.39 (C-1-Hep, $J_{C1\text{-}H1}$ = 173 Hz), 95.67 (*C*Cl3), 87.26 (C-1-ManN, $J_{C1\text{-}H1}$ = 171 Hz), 78.33, 75.68, 74.98, 74.68, 74.60, 74.33, 74.23, 73.66, 73.40, 73.33, 73.28, 73.18, 73.15, 72.17, 71.88, 71.12, 70.28, 68.77, 66.14, 55.64, 46.82, 46.66, 42.19, 42.08 (*C*H-Fmoc), 21.22 (*C*H3-STol), 21.04 (C-4-3Hb). HRMS (ESI): m/z calcd for $C_{84}H_{88}Cl_3N_2O_{16}S^+$ $[M+NH_4]^+$ 1517.4915, found 1517.4923.

Dimethylthexylsilyl [2-*O*-fluorenylmethoxycarbonyl-3,4,7-tri-*O*-benzyl-6-*O*-[benzyl 3*(S)*-butanoate]-D-*glycero*-*α*-D-*manno*-heptopyranosyl]-(1→3)-[2-*N*-2,2,2-(trichloroethyloxy)carbonylamino-2-deoxy-4,6-di-*O*-benzyl-*α*-D-mannopyranosyl]-(1→4)-[[2,3,4-tri-*O*-benzyl-*α*-L-fucopyranosyl-(1→2)]-3-azide-3,6-di-deoxy-*β*-D-glucopyranosyl]-(1→3)-2-*N*-benzyloxycarbonyl-4-azide-2,4,6-tri-deoxy-*β*-D-galactopyranoside (6)

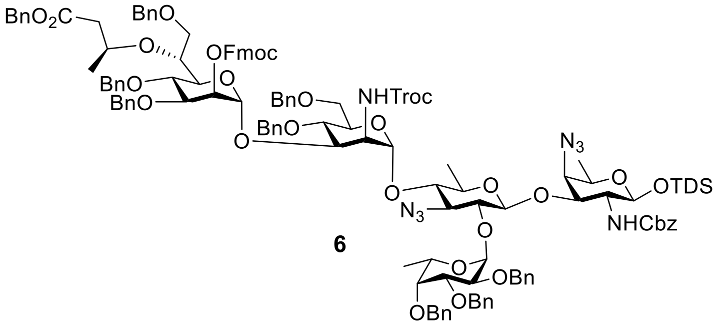

6

Thioglycoside donor **7** (50 mg, 0.048 mmol) and acceptor **8** (86 mg, 0.057 mmol) were co-evaporated three times with PhMe. Then, the mixture was dissolved in anhydrous CH_2Cl_2 (4 mL) and added freshly activated 4 Å molecular sieves. The mixture was stirred at room temperature for 15 min under an atmosphere of argon and then cooled to 0 °C. After being stirred at 0 °C for 15 min, NIS (21 mg, 0.095 mmol) and TfOH (0.8 μL, 0.01 mmol) were added. The reaction was gradually warmed to room temperature and stirred at room temperature for 30 min. TLC analysis showed complete conversion of acceptor to a major product **6** (petroleum ether/ethyl acetate = 3:1, R_f = 0.75). The reaction was quenched by the addition of pyridine and the mixture was filtered. The filtrate was concentrated *in vacuo* and purified by silica gel column chromatography (petroleum ether/ethyl acetate/CH_2Cl_2 = 10/1/1) to give compound **6** (106 mg, 92%) as a colorless oil. ^{1}H NMR (600 MHz, $CDCl_3$) δ 7.84 – 7.76 (m, 2H, Ar-H), 7.60 (t, *J* = 8.3 Hz, 2H, Ar-H), 7.44 – 7.19 (m, 54H, Ar-H), 6.15 (d, *J* = 6.7 Hz, 1H, N*H*Cbz), 5.60 (s, 1H, H-1-Fuc), 5.45 (d, *J* = 8.8 Hz, 1H, N*H*Troc), 5.35 (s, 1H, H-1-Hep), 5.31 (s, 1H, H-1-ManN), 5.28 – 5.23 (m, 3H, H-2-Hep, H-1-AAT, CH*H*-Cbz), 5.00 (d, *J* = 3.8 Hz, 2H, C*H2*-Bn), 4.98 (d, *J* = 11.5 Hz, 1H, C*H*H-Bn), 4.92 (d, *J* = 11.0 Hz, 1H, C*H*H-Bn), 4.87 – 4.64 (m, 12H, C*H2*-Troc, CH*H*-Cbz, 9 ×C*H*H-Bn), 4.59 (d, *J* = 11.3 Hz, 1H, C*H*H-Bn), 4.55 – 4.48 (m, 3H, 3 × C*H*H-Bn), 4.46 – 4.42 (m, 2H, H-3-AAT, C*H*H-Bn), 4.41 – 4.34 (m, 3H, H-2-ManN, 2 × C*H*H-Bn), 4.32

– 4.27 (m, 1H, H-3-3Hb), 4.26 – 4.16 (m, 3H, H-3-ManN, C*H*H-Fmoc, H-5-Hep), 4.14 (t, *J* = 7.7 Hz, 1H, C*H*-Fmoc), 4.12 – 4.07 (m, 2H, H-2-Fuc, H-4-Fuc), 4.07 – 4.03 (m, 1H, H-5-Fuc), 4.02 – 3.95 (m, 3H, H-3-Hep, H-6-Hep, H-1-ADG), 3.90 – 3.85 (m, 1H, H-5-ManN), 3.84 – 3.78 (m, 2H, H-6a-ManN, H-4-ManN), 3.74 – 3.69 (m, 2H, H-7a-Hep, H-4-AAT), 3.67 – 3.61 (m, 4H, H-4-Hep, H-5-ADG, H-3-Fuc, H-6b-ManN), 3.59 (dd, *J* = 10.4, 8.2 Hz, 1H, H-7b-Hep), 3.55 (dd, *J* = 9.7, 7.7 Hz, 1H, H-2-ADG), 3.17 – 3.06 (m, 2H, H-2-AAT, H-3-ADG), 3.08 – 3.01 (m, 2H, H-4-ADG, H-5-ADG), 2.98 (dd, *J* = 14.9, 5.0 Hz, 1H, H-2-3Hb), 2.57 (dd, *J* = 15.0, 8.2 Hz, 1H, H-2-3Hb), 1.68 – 1.58 (m, 1H, C*H*(CH_3)$_2$-TDS), 1.31 – 1.15 (m, 12H, CH_3-AAT, CH_3-Fuc, CH_3-ADG, CH_3-3Hb), 0.88 (dd, *J* = 6.8, 3.5 Hz, 6H, CH(CH_3)$_2$-TDS), 0.85 (d, *J* = 2.4 Hz, 6H, C(CH_3)$_2$-TDS), 0.15 (s, 3H, SiCH_3-TDS), 0.11 (s, 3H, SiCH_3-TDS); ^{13}C NMR (151 MHz, $CDCl_3$) δ 171.44 (*C*=O-3Hb), 155.92 (*C*=O-Cbz), 154.78 (*C*=O-Fmoc), 154.40 (*C*=O-Troc), 143.62, 143.33, 141.32, 141.28, 139.08, 139.05, 138.67, 138.58, 138.49, 137.92, 137.76, 137.53, 136.98, 136.11, 128.98, 128.90, 128.81, 128.75, 128.61, 128.57, 128.53, 128.47, 128.43, 128.38, 128.36, 128.35, 128.28, 128.23, 128.20, 128.17, 128.10, 128.01, 127.97, 127.94, 127.92, 127.87, 127.82, 127.79, 127.76, 127.68, 127.60, 127.49, 127.45, 127.42, 127.39, 127.36, 127.26, 127.23, 127.19, 125.45, 125.28, 120.07, 120.04, 103.87 (C-1-ADG, J_{C1-H1} = 163 Hz), 100.39 (C-1-ManN, J_{C1-H1} = 172 Hz), 99.35 (C-1-Hep, J_{C1-H1} = 173 Hz), 97.71 (C-1-Fuc, J_{C1-H1} = 171 Hz), 95.63 (*C*Cl_3-Troc), 94.13 (C-1-AAT, J_{C1-H1} = 160 Hz), 79.99, 79.31, 79.07, 78.93, 78.25, 77.37, 76.57, 76.47, 75.18, 74.86, 74.73, 74.70, 74.41, 74.18, 73.84, 73.82, 73.66, 73.53, 73.16, 73.09, 72.96, 72.93, 72.41, 72.17, 71.80, 70.75, 70.25, 68.69, 68.53, 68.35, 67.54, 66.21, 65.95, 65.68, 56.85, 54.88, 46.59 (*C*H-Fmoc), 42.17 (*C*H_2-3Hb), 34.09 (*C*H(CH_3)$_2$-TDS), 24.89 (*C*(CH_3)$_2$-TDS), 20.65 (C-4-3Hb), 20.15 (C(*C*H_3)$_2$-TDS), 20.10 (C(*C*H_3)$_2$-TDS), 18.64 (CH(*C*H_3)$_2$-TDS), 18.61 (CH(*C*H_3)$_2$-TDS), 18.45 (C-6-ADG), 17.70 (C-6-AAT), 16.51 (C-6-Fuc), -1.83 (Si*C*H_3-TDS), -3.55 (Si*C*H_3-TDS). HRMS (ESI): m/z calcd for $C_{132}H_{149}Cl_3N_8NaO_{28}Si^+$ [M+Na]$^+$ 2449.9208, found 2449.9202.

[6-*O*-[3*(S)*-butanoic acid]-D-*glycero*-*α*-D-*manno*-heptopyranosyl]-(1→3)-[2-*N*-acetyl-2-deoxy-*α*-D-mannopyranosyl]-(1→4)-[[*α*-L-fucopyranosyl-(1→2)]-3-*N*-acetyl-3,6-di-deoxy-*β*-D-glucopyranosyl]-(1→3)-2-amino-4-*N*-acetyl-2,4,6-tri-deoxy-*α*/*β*-D-galactopyranoside (1)

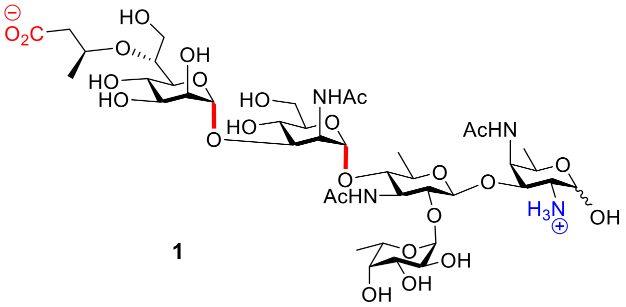

1

The fully protected compound **6** (40 mg, 0.016 mmol) was dissolved in THF/Ac_2O/AcOH (3:2:1, v/v/v, 2.4 mL) followed by addition of Zn dust (100 mg) under an atmosphere of argon. The reaction mixture was stirred vigorously at room temperature overnight. MAIDI-TOF MS analysis showed conversion of starting material to a major NHAc product. The reaction was quenched with MeOH and filtered through celite. The filtrate was concentrated, diluted with CH_2Cl_2, and washed with saturated $NaHCO_3$ solution. The organic layer was dried over Na_2SO_4 and concentrated under reduced pressure to afford NHAc intermediate. The NHAc intermediate was dissolved in pyridine (10 mL), followed by the addition of HF/Pyridine (70%, 1 mL) at 0 ºC. The reaction was stirred at room temperature overnight. TLC analysis showed complete conversion of starting material to a major hemiacetal product (CH_2Cl_2/MeOH/ethyl acetate = 15/0.5/1, R_f = 0.30). The reaction was quenched with saturated $NaHCO_3$ solution and diluted with CH_2Cl_2. The organic phase was separated and washed with brine and dried over Na_2SO_4. The filtration was concentrated under reduced pressure and purified using preparative thin-layer chromatography (CH_2Cl_2/MeOH/ethyl acetate = 15/0.5/1) to give hemiacetal intermediate. To a solution of the hemiacetal intermediate in *t*-BuOH/H_2O/CH_2Cl_2 (3/2/0.5, v/v/v, 5.5 mL) was added $Pd(OH)_2$/C (20%, 20 mg). The mixture was stirred at room temperature for 36 h under H_2 atmosphere. ESI-MS analysis showed the complete conversion of starting material to a major product **1**. The reaction mixture was filtered through celite, and filtration was concentrated under reduced pressure to give a crude product, which was sequentially purified by reverse-phase silica column (C-18, eluent: 1% CH_3CN in H_2O to 20%) and size-exclusion chromatography (BioGel P-4, 45−90 μm, eluent: 0.1 M NH_4HCO_3). The product-containing fractions were combined and lyophilized to afford the product **1** as a white amorphous solid (7.6 mg, 47% over three steps). ^{1}H NMR (600 MHz, D_2O) δ 5.41 (d, J = 3.9 Hz, 0.5H, H-1-AAT-*α*), 5.11 (d, J = 3.4 Hz, 1H, H-1-Fuc), 5.09 (s, 1H, H-1-Hep), 4.99 (d, J = 2.8 Hz, 1H, H-1-ManNAc), 4.78 – 4.73 (m, 1.4 H, H-1-ADG, H-1-AAT-*β*), 4.55 (d, J = 4.7 Hz, 0.5H, H-4-AAT-*α*), 4.51 (d, J = 4.8 Hz, 0.5H,

H-4-AAT-*β*), 4.45 – 4.37 (m, 1H, H-5-AAT-*α*, H-3-AAT-*α*), 4.31 – 4.25 (m, 2H, H-2-ManNAc, H-5-Fuc), 4.25 – 4.20 (m, 1H, H-3-3Hb), 4.14 – 4.09 (m, 0.4H, H-3-AAT-*β*), 4.07 (t, J = 10.3 Hz, 1H, H-3-ADG), 3.99 (dd, J = 9.6, 4.6 Hz, 1H, H-3-ManNAc), 3.96 – 3.90 (m, 1.4 H, H-2-Hep, H-5-AAT-*β*), 3.90 – 3.86 (m, 1H, H-6-Hep), 3.86 – 3.82 (m, 3H, H-6-ManNAc, H-4-Hep), 3.82 – 3.77 (m, 2H), 3.77 – 3.69 (m, 6H), 3.70 (dd, J = 9.7, 3.4 Hz, 1H, H-3-ManNAc), 3.67 – 3.65 (m, 1H, H-5-ADG), 3.56 (d, J = 8.4 Hz, 0.55H, H-2-AAT-*α*), 3.53 – 3.46 (m, 1H, H-2-ADG), 3.41 (t, J = 9.6 Hz, 1H, H-4-ADG), 3.16 (s, 0.5H, H-2-AAT-*β*), 2.60 (dd, J = 13.6, 6.9 Hz, 1H, H-2a-3Hb), 2.32 (dd, J = 13.6, 6.8 Hz, 1H, H-2b-3Hb), 2.10 (d, 3H, CH_3-NHAc-AAT-*α*/*β*), 2.08 (s, 3H, CH_3-NHAc), 2.04 (s, 3H, CH_3-NHAc), 1.35 (d, J = 6.1 Hz, 3H, CH_3-ADG), 1.26 (d, J = 6.1 Hz, 3H, CH_3-3Hb), 1.24 – 1.20 (m, 3H, CH_3-Fuc), 1.15 – 1.10 (m, 3H, CH_3-AAT-*α*/*β*). ^{13}C NMR (151 MHz, D_2O) δ 180.02 (*C*=O-COOH), 174.41 (*C*=O-NHAc), 174.19 (*C*=O-NHAc), 173.15 (*C*=O-NHAc), 102.81 (C-1-ADG, J_{C1-H1} = 163 Hz), 101.27 (C-1-Hep, J_{C1-H1} = 171 Hz), 100.09 (C-1-ManNAc, J_{C1-H1} = 174 Hz), 99.03 (C-1-Fuc, J_{C1-H1} = 169 Hz), 93.92 (C-1-AAT-*β*), 89.15 (C-1-AAT-*α*, J_{C1-H1} = 169 Hz), 80.74, 78.49, 75.21, 74.29, 73.90, 73.32, 72.63, 71.58, 71.53, 71.33, 70.31, 70.07, 69.58, 68.66, 67.70, 66.06, 65.59, 65.03, 61.75, 59.73, 55.00, 53.28, 52.33, 52.06, 49.94, 45.21 (C-2-3Hb), 22.35 (CH_3-NHAc), 21.51 (CH_3-NHAc), 21.34 (CH_3-NHAc), 18.90 (CH_3-3Hb), 17.27 (CH_3-ADG), 15.19 (CH_3-Fuc), 15.09 (CH_3-AAT). HRMS (ESI): m/z calcd for $C_{41}H_{71}N_4O_{25}^+$ $[M+H]^+$ 1019.4402, found 1019.4406.

5. Synthesis of pentasaccharide 2

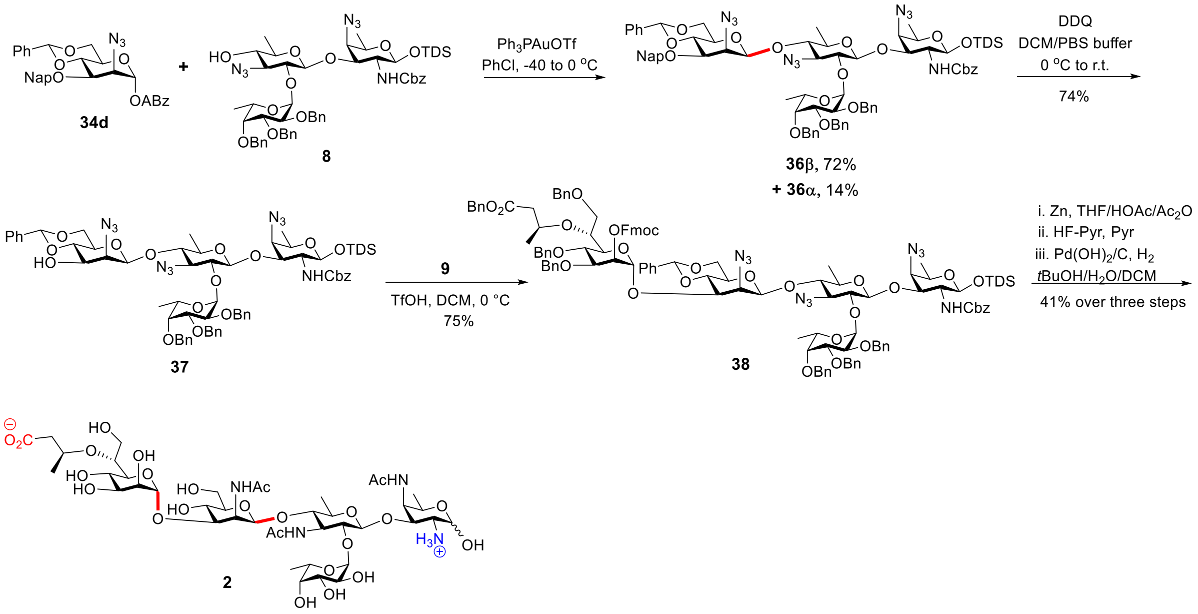

Scheme S13. Synthesis of pentasaccharide **2**

Dimethylthexylsilyl [2-azide-2-deoxy-4,6-*O*-benzylidene-3-*O*-naphthylmethyl-*β*-D-mannopyranosyl]-(1→4)-[[2,3,4-tri-*O*-benzyl-*α*-L-fucopyranosyl-(1→2)]-3-azide-3,6-di-deoxy-*β*-D-glucopyranosyl]-(1→3)-2-*N*-benzyloxycarbonyl-4-azide-2,4,6-tri-deoxy-*β*-D-galactopyranoside (36*β*)

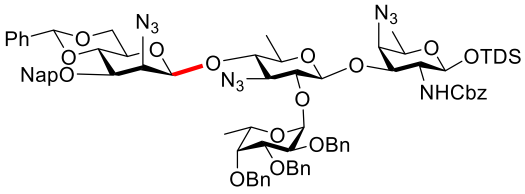

36β

The ABz donor **34d** (97 mg, 0.16 mmol) and acceptor **8** (110 mg, 0.10 mmol) were co-evaporated three times with PhMe. Then, the mixture was dissolved in anhydrous PhCl (2 mL) and added freshly activated 4 Å molecular sieves. The mixture was stirred at room temperature for 15 min under an atmosphere of argon and then cooled to -40 °C. After being stirred at -40 °C for 15 min, a solution of $Ph_3PAuOTf$ in PhCl (0.04 M, 0.5 mL, 0.2 equiv) was added. The reaction was gradually warmed to room temperature over 8 h. TLC analysis showed complete conversion of acceptor to a major product **36*β*** (petroleum ether/ethyl acetate = 3/1, R_f = 0.60) and **36*α*** (petroleum ether/ethyl acetate = 3/1, R_f = 0.75). The reaction was quenched by the addition of Et_3N and filtered. The filtrate was concentrated *in vacuo* and resulting residue was purified by silica gel column chromatography (petroleum ether/ethyl acetate/CH_2Cl_2 = 6/1/1 to 4/1/1) to give compound **36*β*** (110 mg, 72%) and **36*α*** (12 mg, 14%) as colorless oil.

36*β*: ^{1}H NMR (600 MHz, Acetone-*d*$_6$) *δ* 7.96 – 7.84 (m, 3H, ArH), 7.77 – 7.70 (m, 1H, ArH), 7.56 – 7.16 (m, 28H, ArH), 6.52 (d, *J* = 8.9 Hz, 1H, N*H*Cbz), 5.75 (s, 1H, PhC*H*), 5.53 (d, *J* = 3.6 Hz, 1H, H-1-Fuc), 5.21 (d, *J* = 12.7 Hz, 1H, C*H*H-Cbz), 5.14 (d, *J* = 1.5 Hz, 1H, H-1-ManN), 5.06 – 4.92 (m, 4H, C*H*H-Cbz, 3 × C*H*H), 4.86 (t, *J* = 12.1 Hz, 2H, 2 × C*H*H), 4.78 – 4.70 (m, 3H, 2 × C*H*H, H-1-AAT), 4.69 – 4.63 (m, 2H, C*H*H, H-1-ADG), 4.54 (dd, *J* = 3.6, 1.4 Hz, 1H, H-2-ManN), 4.50 – 4.44 (m, 1H, H-5-Fuc), 4.40 – 4.28 (m, 2H, H-3-AAT, H-6a-ManN), 4.14 – 4.05 (m, 3H, H-3-Fuc, H-3-ManN, H-2-Fuc), 4.02 (t, *J* = 9.5 Hz, 1H, H-4-ManN), 3.99 – 3.96 (m, 2H, H-4-AAT, H-4-Fuc), 3.89 (t, *J* = 10.2 Hz, 1H, H-6-ManN), 3.81 – 3.74 (m, 1H, H-5-AAT), 3.64 – 3.57 (m, 1H, H-2-AAT), 3.56 – 3.44 (m, 5H, H-5-ADG, H-4-ADG, H-5-ManN, H-2-ADG, H-3-ADG), 1.65 – 1.59 (m, 1H, C*H*(CH_3)$_2$-TDS), 1.35 (d, *J* = 5.7 Hz, 3H, C*H*$_3$-AAT), 1.33 (d, *J* = 6.4 Hz, 3H, C*H*$_3$-Fuc), 1.27 (d, *J* = 6.3 Hz, 3H, C*H*$_3$-ADG), 0.86 (d, *J* = 6.9 Hz, 6H, CH(C*H*$_3$)$_2$-TDS), 0.83 (d, *J* = 2.4 Hz, 6H, C(C*H*$_3$)$_2$-TDS), 0.14 (s, 3H, SiC*H*$_3$-TDS), 0.11 (s, 3H, SiC*H*$_3$-TDS). ^{13}C NMR (151 MHz, Acetone-*d*$_6$) *δ* 157.06 (C=O-Cbz), 140.52, 140.42, 140.07, 139.08, 138.00, 137.16, 134.24, 133.88, 129.56, 129.24, 129.02, 128.95, 128.92, 128.86, 128.85, 128.77, 128.72, 128.66,

128.58, 128.54, 128.52, 128.48, 128.46, 128.03, 127.96, 127.87, 127.81, 127.16, 126.88, 126.71, 126.63, 126.42, 103.43 (C-1-ADG, $J_{C1\text{-}H1}$ = 163 Hz), 102.26 (PhC*H*), 101.31 (C-1-ManN, $J_{C1\text{-}H1}$ = 161 Hz), 98.08 (C-1-Fuc, $J_{C1\text{-}H1}$ = 171 Hz), 97.53 (C-1-AAT, $J_{C1\text{-}H1}$ = 161 Hz), 82.79, 80.09, 79.58, 79.08, 78.23, 77.59, 77.25, 76.78, 75.68, 73.24, 73.10, 72.51, 72.15, 69.52, 69.05, 68.84, 68.00, 67.98, 67.51, 66.70, 64.25, 56.35, 34.67 (CH(CH_3)$_2$-TDS), 25.48 (C(CH_3)$_2$-TDS), 20.47 (C(CH$_3$)$_2$-TDS), 20.35 (C(CH$_3$)$_2$-TDS), 18.94 (CH(CH$_3$)$_2$-TDS), 18.86 (CH(CH$_3$)$_2$-TDS), 18.20 (C-6-AAT), 17.83 (C-6-Fuc), 16.71 (C-6-ADG), -1.71 (SiCH$_3$-TDS), -2.95 (SiCH$_3$-TDS). HRMS (ESI): m/z calcd for $C_{79}H_{94}N_{10}NaO_{16}Si^+$ [M+Na]$^+$ 1489.6511, found 1489.6514.

36*α*: ^{1}H NMR (600 MHz, Acetone-*d$_6$*) *δ* 7.99 – 7.77 (m, 3H, ArH), 7.77 – 7.68 (m, 1H, ArH), 7.59 – 7.16 (m, 28H, ArH), 6.54 (d, *J* = 8.7 Hz, 1H, N*H*Cbz), 5.80 (s, 1H, PhC*H*), 5.46 (d, *J* = 3.5 Hz, 1H, H-1-Fuc), 5.24 – 5.16 (m, 2H, H-1-ManN, C*H*H-Cbz), 5.10 – 4.93 (m, 4H, C*H*H-Cbz, 3 × C*H*H), 4.86 – 4.70 (m, 5H, H-1-AAT, 4 × C*H*H), 4.65 (d, *J* = 11.5 Hz, 1H, C*H*H), 4.60 (d, *J* = 7.0 Hz, 1H, H-1-ADG), 4.47 (dd, *J* = 3.2, 1.7 Hz, 1H, H-2-ManN), 4.44 – 4.37 (m, 1H, H-5-Fuc), 4.35 – 4.27 (m, 1H, H-3-AAT), 4.24 – 4.15 (m, 3H, H-3-ManN, H-4-ManN, H-6a-ManN), 4.11 (dd, *J* = 10.5, 2.7 Hz, 1H, H-3-Fuc), 4.05 (dd, *J* = 10.3, 3.7 Hz, 1H, H-2-Fuc), 4.01 – 3.94 (m, 2H, H-4-AAT, H-4-Fuc), 3.92 – 3.88 (m, 1H, H-5-ManN), 3.86 (d, *J* = 9.9 Hz, 1H, H-6-ManN), 3.82 – 3.75 (m, 1H, H-5-AAT), 3.61 – 3.43 (m, 4H, H-2-AAT, H-2-ADG, H-3-ADG, H-5-ADG), 3.32 (t, *J* = 9.2 Hz, 1H, H-4-ADG), 1.66 – 1.57 (m, 1H, C*H*(CH_3)$_2$-TDS), 1.35 (d, *J* = 6.1 Hz, 3H, CH_3-AAT), 1.32 (d, *J* = 6.6 Hz, 3H, CH_3-Fuc), 1.27 (d, *J* = 6.3 Hz, 3H, CH_3-ADG), 0.85 (2 × s, 6H, CH(CH_3)$_2$-TDS), 0.82 (s, 6H, C(CH_3)$_2$-TDS), 0.12 (s, 3H, SiCH_3-TDS), 0.10 (s, 3H, SiCH_3-TDS). ^{13}C NMR (151 MHz, Acetone-*d$_6$*) *δ* 157.07 (*C*=O-Cbz), 140.50, 140.36, 140.10, 139.04, 138.13, 136.97, 134.28, 133.94, 129.63, 129.28, 129.01, 128.96, 128.93, 128.90, 128.83, 128.79, 128.70, 128.61, 128.58, 128.55, 128.52, 128.45, 128.07, 128.00, 127.97, 127.14, 126.93, 126.69, 126.65, 126.30, 103.51 (C-1-ADG, $J_{C1\text{-}H1}$ = 163 Hz), 102.28 (Ph*C*H), 101.98 (C-1-ManN, $J_{C1\text{-}H1}$ = 171 Hz), 98.43 (C-1-Fuc, $J_{C1\text{-}H1}$ = 172 Hz), 97.42 (C-1-AAT, $J_{C1\text{-}H1}$ = 159 Hz), 83.49, 80.16, 79.42, 78.39, 77.87, 77.12, 76.55, 75.68, 73.44, 73.18, 73.06, 71.90, 69.91, 69.64, 68.86, 68.39, 67.22, 66.66, 65.80, 63.84, 56.41, 34.73 (CH(CH_3)$_2$-TDS), 25.51 (C(CH_3)$_2$-TDS), 20.48 (C(CH$_3$)$_2$-TDS), 20.38 (C(CH$_3$)$_2$-TDS), 18.96 (CH(CH$_3$)$_2$-TDS), 18.88 (CH(CH$_3$)$_2$-TDS), 18.43 (C-6-AAT), 17.83 (C-6-Fuc), 16.81 (C-6-ADG), -1.70 (SiCH$_3$-TDS), -3.00 (SiCH$_3$-TDS). HRMS (ESI): m/z calcd for $C_{79}H_{94}N_{10}NaO_{16}Si^+$ [M+Na]$^+$ 1489.6511, found 1489.6514.

Table S8. Stereoselective Construction of *β*-ManN$_3$ linkage

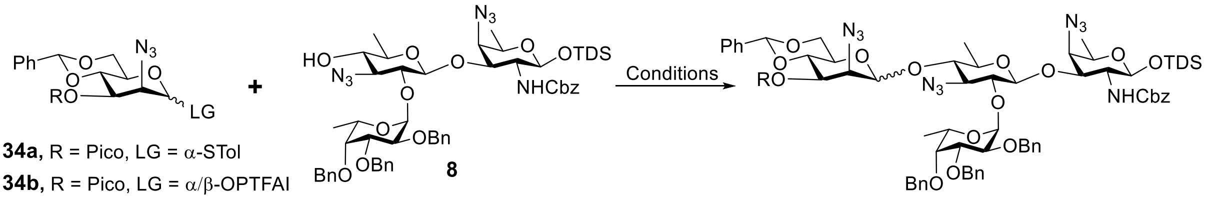

34a, R = Pico, LG = α-STol
34b, R = Pico, LG = α/β-OPTFAI
34c, R = Nap, LG = α-STol
34d, R = Nap, LG = α-OABz
34e, R = Nap, LG = β-OABz

35, R = Pico
36, R = Nap

Entry	Donor	Conditions	Product	Yield[a] (*β*/*α*)[b]
S1	**34a**	NIS/TfOH, CH_2Cl_2, 0 °C to r.t.	**35**	N.R.
S2	**34b**	TBSOTf, CH_2Cl_2, 0 °C to r.t.	**35**	Trace[c]
S3	**34c**	Ph_2SO, Tf_2O, TTBP, CH_2Cl_2, -60 °C to r.t.	**36**	30% (*β*/*α* = 2.5/1)
S4	**34d**	$Ph_3PAuOTf$, PhMe, -40 °C to 0 °C	**36**	89% (*β*/*α* = 3.5/1)
S5	**34e**	$Ph_3PAuOTf$, PhMe, -40 °C to 0 °C	**36**	87% (*β*/*α* = 1.5/1)
S6	**34d**	$Ph_3PAuOTf$, PhCl, -40 °C to 0 °C	**36**	86% (*β*/*α* = 5/1)

[a] Isolated yield. [b] The *β*/*α* ratio was determined by the isolated yield ratio. N.R., no reaction; Ph_2SO, diphenylsulfoxide; TTBP, 2,4,6-tri-*tert*-butylpyrimidine.

Dimethylthexylsilyl [2-azide-2-deoxy-4,6-*O*-benzylidene-*β*-D-mannopyranosyl]-(1→4)-[[2,3,4-tri-*O*-benzyl-*α*-L-fucopyranosyl-(1→2)]-3-azide-3,6-di-deoxy-*β*-D-glucopyranosyl]-(1→3)-2-*N*-benzyloxycarbonyl-4-azide-2,4,6-tri-deoxy-*β*-D-galactopyranoside (37)

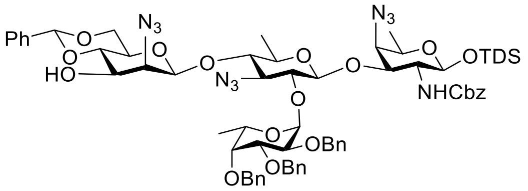

37

To solution of a mixture of **36*β*** (100 mg, 0.068 mmol) in a mixed solvent of CH_2Cl_2/0.1 M PBS buffer (v/v, 10/1, 5.5 mL) was added DDQ (23 mg, 0.102 mmol) at 0 °C. After being stirred at room temperature for 6 h, the reaction was diluted with ethyl acetate, and washed with saturated aqueous $NaHCO_3$ and brine. The organic phase was dried (Na_2SO_4), and filtered. The filtrate was concentrated under reduced pressure to give crude product. The crude product was purified by silica gel column chromatography (petroleum ether/ethyl acetate/CH_2Cl_2= 4/1/1) to give

compound **8** (67 mg, 74%) as a colorless oil. ^{1}H NMR (600 MHz, Acetone-*d$_6$*) δ 7.65 – 7.05 (m, 25H), 6.52 (d, *J* = 8.9 Hz, 1H, N*H*Cbz), 5.63 (s, 1H, PhC*H*), 5.53 (d, *J* = 3.6 Hz, 1H), 5.20 (d, *J* = 12.6 Hz, 1H), 5.14 (d, *J* = 1.5 Hz, 1H), 4.99 (t, *J* = 11.9 Hz, 2H), 4.90 – 4.84 (m, 2H), 4.81 – 4.70 (m, 4H), 4.69 – 4.62 (m, 2H), 4.47 (d, *J* = 6.7 Hz, 1H), 4.38 – 4.33 (m, 1H), 4.30 (dd, *J* = 10.2, 4.9 Hz, 1H), 4.27 – 4.23 (m, 1H), 4.16 – 4.03 (m, 3H), 3.97 (s, 2H), 3.86 – 3.72 (m, 3H), 3.65 – 3.58 (m, 1H), 3.57 – 3.42 (m, 5H), 1.69 – 1.59 (m, 1H, C*H*(CH$_3$)$_2$-TDS), 1.39 (d, *J* = 5.6 Hz, 3H), 1.32 (d, *J* = 6.5 Hz, 3H), 1.27 (d, *J* = 6.3 Hz, 3H), 0.85 (d, *J* = 7.0 Hz, 6H, CH(C*H$_3$*)$_2$-TDS), 0.82 (s, 6H, C(C*H$_3$*)$_2$-TDS), 0.13 (s, 3H, SiC*H$_3$*-TDS), 0.10 (s, 3H, SiC*H$_3$*-TDS). ^{13}C NMR (151 MHz, Acetone-*d$_6$*) δ 157.08 (*C*=O-Cbz), 140.56, 140.45, 140.11, 139.12, 138.03, 129.55, 129.26, 128.96, 128.93, 128.89, 128.87, 128.76, 128.59, 128.57, 128.54, 128.04, 127.97, 127.87, 127.28, 103.47, 102.51, 101.40, 98.09, 97.57, 82.81, 80.12, 79.62, 78.26, 77.29, 76.81, 75.70, 73.27, 73.12, 72.23, 70.73, 70.63, 69.56, 69.04, 68.91, 68.04, 67.55, 66.80, 56.39, 34.70, 25.51, 20.49, 20.38, 18.96, 18.87, 18.26, 17.84, 16.73, -1.69 (Si*C*H$_3$-TDS), -2.93 (Si*C*H$_3$-TDS). HRMS (ESI): m/z calcd for $C_{68}H_{86}N_{10}NaO_{16}Si^+$ [M+Na]$^+$ 1349.5885, found 1349.5887.

Dimethylthexylsilyl [2-*O*-fluorenylmethoxycarbonyl-3,4,7-tri-*O*-benzyl-6-*O*-[benzyl 3(*S*)-butanoate]-D-*glycero*-*α*-D-*manno*-heptopyranosyl]-(1→3)-[2-azide-2-deoxy-4,6-*O*-benzylidene-*β*-D-mannopyranosyl]-(1→4)-[[2,3,4-tri-*O*-benzyl-*α*-L-fucopyranosyl-(1→2)]-3-azide-3,6-di-deoxy-*β*-D-glucopyranosyl]-(1→3)-2-*N*-benzyloxycarbonyl-4-azide-2,4,6-tri-deoxy-*β*-D-galactopyranoside (38)

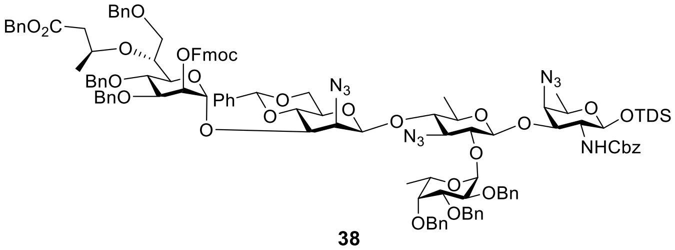

38

The Hep-OPTFAI donor **9** (95 mg, 0.09 mmol) and acceptor **37** (100 mg, 0.075 mmol) were co-evaporated three times with PhMe. Then, the mixture was dissolved in anhydrous CH_2Cl_2 (2.5 mL) and added freshly activated 4 Å molecular sieves. The mixture was stirred at room temperature for 15 min under an atmosphere of argon and then cooled to 0 °C. After being stirred at 0 °C for 15 min, TfOH (1.3 μL, 0.015 mmol) was added. Then, the reaction was gradually warmed to room temperature over 0.5 h. TLC analysis showed complete conversion of acceptor to a major product **38** (petroleum ether/ethyl acetate = 4:1, R$_f$ = 0.75). The reaction was quenched by the addition of pyridine and filtered. The filtrate was concentrated *in vacuo* and purified by silica gel column chromatography

(petroleum ether/ethyl acetate/CH_2Cl_2 = 6/1/1) to give compound **38** (126 mg, 76%) as a colorless oil. ^{1}H NMR (800 MHz, Acetone-*d*$_6$) δ 7.87 (dd, *J* = 7.4, 6.0 Hz, 2H, ArH), 7.69 – 7.64 (m, 2H, ArH), 7.49 – 7.17 (m, 49H), 6.55 (d, *J* = 8.9 Hz, 1H, N*H*Cbz), 5.72 (s, 1H, PhC*H*), 5.53 (d, *J* = 3.7 Hz, 1H, H-1-Fuc), 5.40 (d, *J* = 2.1 Hz, 1H, H-1-Hep), 5.33 (dd, *J* = 3.1, 2.0 Hz, 1H, H-2-Hep), 5.24 – 5.16 (m, 2H, H-1-ManN, C*H*H-Cbz), 5.07 (d, *J* = 12.7 Hz, 1H, C*H*H-Cbz), 5.03 – 4.97 (m, 2H, 2 × C*H*H-Bn), 4.91 – 4.83 (m, 3H, 3 × C*H*H-Bn), 4.78 – 4.73 (m, 4H, H-1-AAT, 3 × C*H*H-Bn), 4.70 – 4.57 (m, 4H, H-1-ADG, 3 × C*H*H-Bn), 4.57 – 4.43 (m, 5H, H-2-ManN, H-5-Fuc, 3 × C*H*H-Bn), 4.39 – 4.32 (m, 4H, H-6a-ManN, H-3-AAT, C*H*$_2$-Fmoc), 4.29 (dd, *J* = 9.8, 3.7 Hz, 1H, H-3-ManN), 4.24 – 4.19 (m, 2H, C*H*-Fmoc, H-3-3Hb), 4.15 – 4.10 (m, 2H, H-3-Fuc, H-2-Fuc), 4.09 – 4.05 (m, 2H, H-5-Hep, H-6-Hep), 4.04 – 4.01 (m, 2H, H-4-Hep, H-4-ManN), 3.99 – 3.97 (m, 1H, H-4-Fuc), 3.97 – 3.95 (m, 1H, H-4-AAT), 3.90 – 3.84 (m, 2H, H-6b-ManN, C*H*H-Bn), 3.81 – 3.77 (m, 1H, H-5-AAT), 3.75 (dd, *J* = 10.2, 4.9 Hz, 1H, H-7a-Hep), 3.63 – 3.54 (m, 2H, H-7b-Hep, H-2-AAT), 3.54 – 3.42 (m, 5H, H-2-ADG, H-3-ADG, H-4-ADG, H-5-ADG, H-5-ManN), 2.73 (dd, *J* = 14.8, 6.9 Hz, 1H, H-2a-3Hb), 2.52 (dd, *J* = 14.8, 6.1 Hz, 1H, H-2b-3Hb), 1.65 – 1.58 (m, 1H, C*H*(CH_3)$_2$-TDS), 1.36 – 1.30 (m, 6H, C*H*$_3$-Fuc, C*H*$_3$-ADG), 1.27 (d, *J* = 6.3 Hz, 3H, C*H*$_3$-AAT), 1.22 (d, *J* = 6.2 Hz, 3H, C*H*$_3$-3Hb), 0.85 (d, *J* = 6.9 Hz, 6H, CH(C*H*$_3$)$_2$-TDS), 0.83 (d, *J* = 3.3 Hz, 6H, C(C*H*$_3$)$_2$-TDS), 0.13 (s, 3H, SiC*H*$_3$-TDS), 0.10 (s, 3H, SiC*H*$_3$-TDS). ^{13}C NMR (151 MHz, Acetone-*d*$_6$) δ 171.84 (*C*=O-3Hb), 166.26 (*C*=O-Cbz), 164.37 (*C*=O-Fmoc), 157.04, 155.27, 144.43, 144.27, 142.09, 142.06, 140.50, 140.40, 140.05, 139.63, 139.57, 139.10, 138.65, 137.98, 137.32, 136.69, 129.59, 129.33, 129.25, 129.16, 129.07, 129.02, 128.99, 128.94, 128.91, 128.84, 128.78, 128.75, 128.71, 128.65, 128.58, 128.53, 128.49, 128.31, 128.16, 128.01, 127.95, 127.81, 126.91, 126.08, 125.94, 120.89, 103.43 (C-1-ADG, J_{C1-H1} = 165 Hz), 101.86 (Ph*C*H), 101.07 (C-1-ManN, J_{C1-H1} = 163 Hz), 98.97 (C-1-Hep, J_{C1-H1} = 172 Hz), 98.08 (C-1-Fuc, J_{C1-H1} = 172 Hz), 97.47 (C-1-AAT, J_{C1-H1} = 159 Hz), 82.59, 80.09, 79.57, 79.43, 79.07, 78.23, 77.27, 77.18, 76.85, 75.66, 75.46, 75.28, 74.68, 73.76, 73.60, 73.25, 73.13, 72.63, 72.29, 72.10, 71.56, 70.57, 69.51, 68.92, 68.85, 68.04, 67.44, 66.69, 66.53, 65.00, 56.36, 46.59 (*C*H-Fmoc), 43.05 (*C*H$_2$-3Hb), 34.67 (*C*H(CH_3)$_2$-TDS), 25.48 (*C*(CH_3)$_2$-TDS), 20.60 (C-4-3Hb), 20.48 (C(*C*H$_3$)$_2$-TDS), 20.36 (C(*C*H$_3$)$_2$-TDS), 18.95 (CH(*C*H$_3$)$_2$-TDS), 18.87 (CH(*C*H$_3$)$_2$-TDS), 18.34 (C-6-ADG), 17.84 (C-6-AAT), 16.72 (C-6-Fuc), -1.70 (Si*C*H$_3$-TDS), -2.94 (Si*C*H$_3$-TDS). MALDI-TOF-MS:[M+Na]$^+$ calcd for $C_{122}H_{138}N_{10}NaO_{26}Si^+$, 2209.9445; found 2209.9789.

[6-*O*-[3-(S)-butanoic acid]-D-*glycero*-*α*-D-*manno*-heptopyranosyl]-(1→3)-[2-*N*-acetyl-2-deoxy-*β*-D-mannopyranosyl]-(1→4)-[*α*-L-fucopyranosyl-(1→2)]-3-*N*-acetyl-3,6-di-deoxy-*β*-D-glucopyranosyl]-(1→3)-

2-amino-4-*N*-acetyl-2,4,6-tri-deoxy-*α*/*β*-D-galactopyranoside (2)

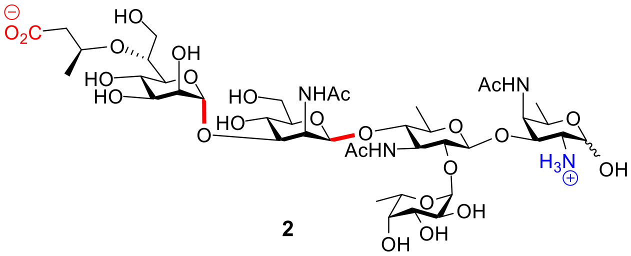

2

The fully protected compound **38** (25 mg, 0.011 mmol) was dissolved in THF/Ac_2O/AcOH (3:2:1, v/v/v, 2.4 mL) followed by addition of Zn dust (50 mg) under an atmosphere of argon. The reaction mixture was stirred vigorously at room temperature overnight. MALDI-TOF MS analysis showed conversion of starting material to a major NHAc product. The reaction was quenched with MeOH and filtered through celite. The filtrate was concentrated, diluted with CH_2Cl_2, and washed with saturated $NaHCO_3$ solution. The organic layer was dried over Na_2SO_4 and concentrated under reduced pressure to afford an NHAc intermediate. The above intermediate was dissolved in pyridine (5 mL), followed by the addition of HF/Pyridine (70%, 0.5 mL) at 0 °C. The reaction was stirred at room temperature overnight. TLC analysis showed complete conversion of starting material to a major hemiacetal product (CH_2Cl_2/MeOH/ethyl acetate = 15/0.5/1, R_f = 0.25). The reaction was quenched with saturated $NaHCO_3$ solution and diluted with CH_2Cl_2. The organic phase was separated and washed with brine and dried over Na_2SO_4. The filtration was concentrated under reduced pressure and purified using preparative thin-layer chromatography (CH_2Cl_2/MeOH/ethyl acetate = 15/0.5/1) to give a hemiacetal intermediate. To a solution of the above hemiacetal intermediate in *t*-BuOH/H_2O/CH_2Cl_2 (3/2/0.5, v/v/v, 5.5 mL) was added $Pd(OH)_2$/C (20%, 20 mg). The mixture was stirred at room temperature for 24 h under H_2 atmosphere. ESI-MS analysis showed the complete conversion of starting material to a major product **2**. The reaction mixture was filtered through celite, and filtration was concentrated under reduced pressure to give a crude product, which was sequentially purified by reverse-phase silica column (C-18, eluent: 1% CH_3CN in H_2O to 20%) and size-exclusion chromatography (BioGel P-2, 45−90 μm, eluent: 0.1 M NH_4HCO_3). The product-containing fractions were combined and lyophilized to afford the product **2** as a white amorphous solid (4.9 mg, 41% over three steps). ^{1}H NMR (600 MHz, D_2O) δ 5.40 (d, J = 3.8 Hz, 0.6 H, H-1-AAT-α), 5.12 (d, J = 2.0 Hz, 1H, 1H, H-1-Hep), 5.09 (s, 1H, H-1-Fuc), 4.85 (d, J = 8.4 Hz, 0.5H, H-1-AAT-β), 4.79 (H-1-ManNAc, obtained from HSQC), 4.77 – 4.71 (m, 1H, H-1-ADG), 4.55 (d, J = 4.8 Hz, 1H, H-2-ManNAc), 4.53 – 4.50 (m, 1H, H-4-AAT), 4.45 – 4.37 (m, 1.3 H, H-3-AAT-α, H-5-AAT-α), 4.24 (dd, J = 7.6, 3.2 Hz, 1H, H-5-Fuc), 4.18 – 4.13 (m, 1.5 H, H-3-AAT-β, H-3-3Hb), 3.95 – 3.87 (m, 6.4H, H-5-AAT-β, H-2-Hep, H-3-ADG, H-3-ManNAc, H-6-Hep, H-6a-ManNAc, H-5-Hep,), 3.85 – 3.78 (m, 2H, H-6b-ManNAc, H-7a-Hep), 3.78 – 3.68 (m,

5H, H-4-Fuc, H-4-Hep, H-2-Fuc, H-7b-Hep, H-3-Fuc), 3.65 – 3.57 (m, 2.6H, H-3-Hep, H-4-ManNAc, H-2-AAT-*α*), 3.56 – 3.52 (m, 1H, H-5-ADG), 3.44 – 3.33 (m, 3H, H-4-ADG, H-2-ADG, H-5-ManNAc), 3.28 – 3.16 (m, 1H, H-2-AAT-*β*), 2.64 (dd, *J* = 13.7, 6.0 Hz, 1H, H-2a-3Hb), 2.28 (dd, *J* = 13.5, 7.7 Hz, 1H, H-2b-3Hb), 2.08 (d, *J* = 4.0 Hz, 3H, CH_3-NHAc-AAT-*α*/*β*), 2.05 (s, 3H, CH_3-NHAc), 2.01 (s, 3H, CH_3-NHAc), 1.30 (d, *J* = 6.2 Hz, 3H, CH_3-ADG), 1.24 (d, *J* = 6.2 Hz, 3H, CH_3-3Hb), 1.23 – 1.19 (m, 3H, CH_3-Fuc), 1.13 – 1.08 (m, 3H, CH_3-AAT-*α*/*β*). ^{13}C NMR (126 MHz, D_2O) δ 179.81 (*C*=O-COOH), 174.66 (*C*=O-NHAc), 174.61 (*C*=O-NHAc), 174.20 (*C*=O-NHAc), 102.81 (C-1-ADG, J_{C1-H1} = 163 Hz), 101.34 (C-1-Hep, J_{C1-H1} = 173 Hz), 99.31 (C-1-Fuc, J_{C1-H1} = 167 Hz), 98.78 (C-1-ManNAc, J_{C1-H1} = 160 Hz), 93.63 (C-1-AAT-*β*), 89.23 (C-1-AAT-*α*, J_{C1-H1} = 174 Hz), 79.38, 79.23, 77.70, 76.83, 76.22, 74.03, 73.88, 73.53, 72.39, 71.98, 71.73, 70.49, 69.88, 69.03, 68.18, 67.12, 67.06, 65.40, 61.02, 60.64, 54.04, 53.70, 52.74, 52.60, 50.36, 45.27 (C-2-3Hb), 22.46 (*C*H_3-NHAc), 21.99 (2 × *C*H_3-NHAc), 19.31 (*C*H_3-3Hb), 17.14 (*C*H_3-ADG), 15.56 (*C*H_3-Fuc), 15.47 (*C*H_3-AAT). HRMS (ESI): m/z calcd for $C_{41}H_{70}N_4NaO_{25}^+$ [M+Na]$^+$ 1041.4221, found 1041.4221.

6. Synthesis of pentasaccharide 3

6.1 Initially strategies for the construction of *β*-Hep linkage

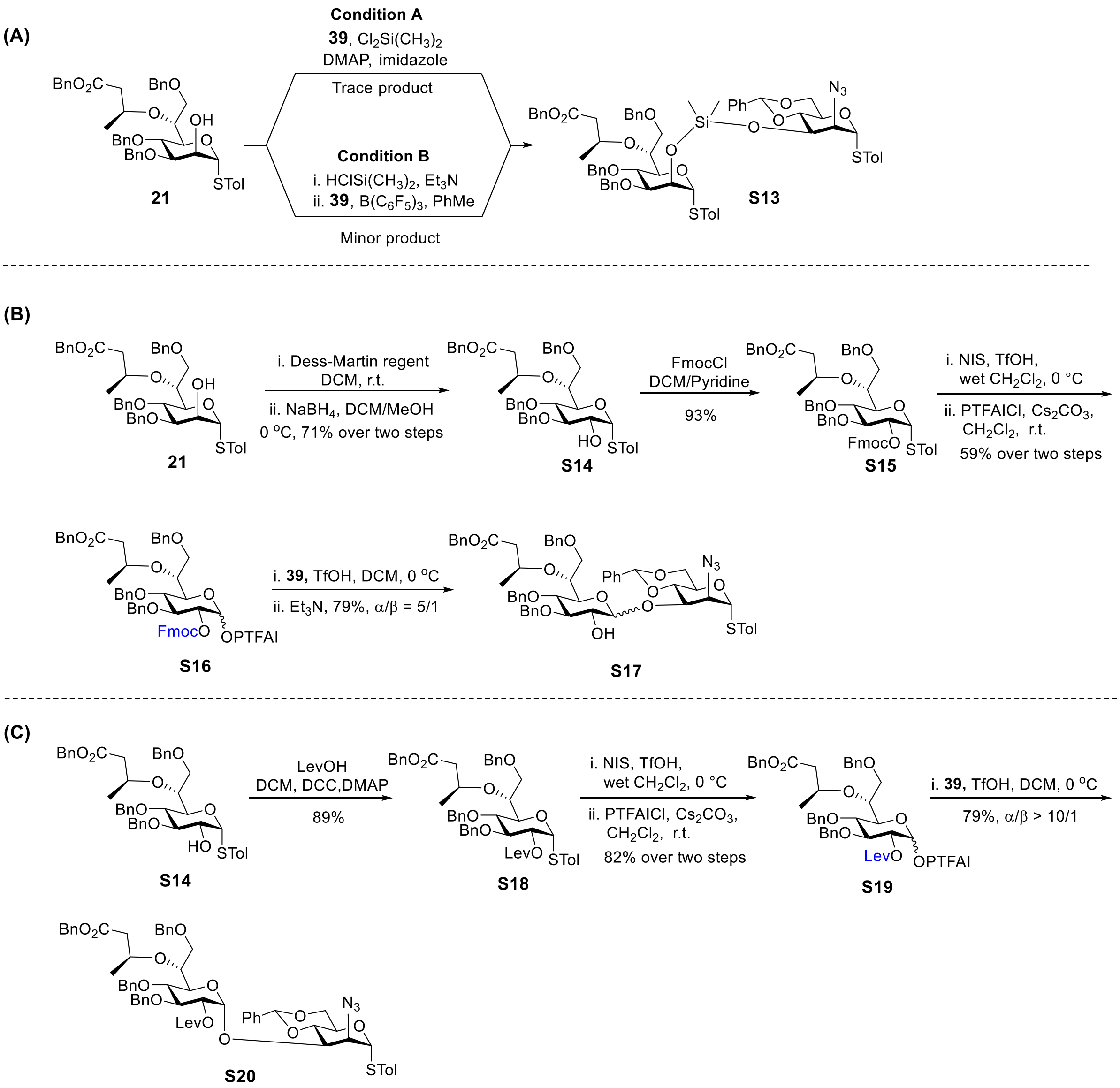

Scheme S14. Failed strategy for the construction of *β*-Hep linkage. **(A)** Silyl-mediated intramolecular aglycone delivery [13,14] **(B-C)** C-2 epimerization of compound **21** followed by neighboring group (Fmoc and Lev) assisted glycosylation.[15]

6.2 Stereoselective glycosylation for the construction of *β*-Hep linkage

Table S9 Stereoselective construction of the *β*-Hep linkage

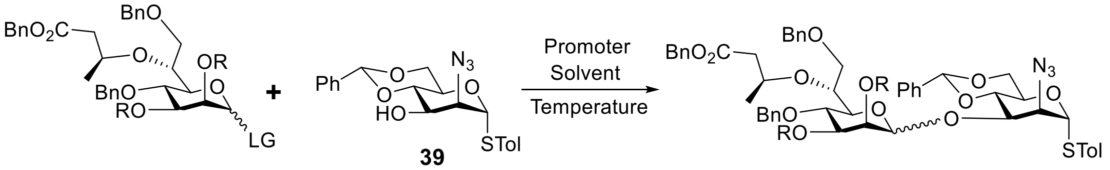

40a: R = Bn, LG = α-OABz
40b: R = isopropylidene, LG = α/β-OABz
40c: R = Bn, LG = α-OTCAI
40d: R = isopropylidene, LG = α-OTCAI

41, R = Bn
42, R = isopropylidene

Entry	Donor	Promoter	Solvent	T (°C)	Product	Yield[a] (β/α)[b]
S1	**40a**	$Ph_3PAuOTf$	PhCl	–40	**41**	73% (*α* only)
S2	**40a**	$Ph_3PAuBAr_4^F$	PhCl	–40 to r.t.	**41**	82% (*α* only)
S3	**40a**	$SPhosAuNTf_2$	PhCl	–40 to r.t.	**41**	53% (α only)
S4	**40b**	$Ph_3PAuOTf$	PhCl	–40	**42**	87% (β/α = 1/7.5)
S5	**40c**	$B(C_6F_5)_3$	DCM	–10	**41**	88% (β/α = 1/4.5)
S6	**40c**	$B(C_6F_5)_3$	DCM	–78	**41**	89% (β/α = 2/1)
S7	**40d**	$B(C_6F_5)_3$	DCM	–78	**42**	82% (β/α = 4/1)

[a]Isolated yield. [b]The *β*/*α* ratio was determined by isolated yield ratio.

The gold(I)-catalyzed glycosylation method (Entries S1-S4)[16-18]**:** The ABz donor **40a-b** (1.5 equiv.) and acceptor **39** (1.0 equiv.) were co-evaporated three times with PhMe. Then, the mixture (75 mM donor and 50 mM acceptor) was dissolved in anhydrous PhCl and added freshly activated 4 Å molecular sieves. The mixture was stirred at room temperature for 15 min under an atmosphere of argon and then cooled to -40 °C. After being stirred at -40 °C for 15 min, the gold-(I) catalyst (0.2 equiv. for $Ph_3PAuOTf$; 0.4 equiv. for $Ph_3PAuBAr_4^F$ and $SphosAuNTf_2$) was added, respectively. Then, the reaction mixture was stirred at conresponding temperature. The reaction was quenched by the addition of Et_3N and filtered. The filtrate was concentrated *in vacuo* and purified by silica gel column chromatography.

The $B(C_6F_5)_3$-promoted glycosylation method (Entries S5-S7)[19]**:** The TCAI donor **40c-d** (1.5 equiv.) and acceptor **39** (1.0 equiv) were co-evaporated three times with PhMe. Then, the mixture (75 mM donor and 50 mM acceptor) was dissolved in anhydrous CH_2Cl_2 and added freshly activated 4 Å molecular sieves. The mixture was stirred at

room temperature for 15 min under an atmosphere of argon and then cooled to -78 °C. After being stirred at -78 °C for 15 min, $B(C_6F_5)_3$ (0.2 equiv.) was added. Then, the reaction was stirred at -78 °C until the TLC analysis showed complete consumption of acceptor. The reaction was quenched by the addition of Et_3N and filtered. The filtrate was concentrated *in vacuo* and resulting residue was purified by silica gel column chromatography.

***p*-Tolyl 2,3,4,7-tetra-*O*-benzyl-6-*O*-[benzyl 3(S)-butanoate]-D-*glycero*-*α*-D-*manno*-heptopyranosyl-(1→3)-2-azide-4,6-*O*-benzylidene-2-deoxy-thio-*α*-D-mannopyranoside (41*α*)**

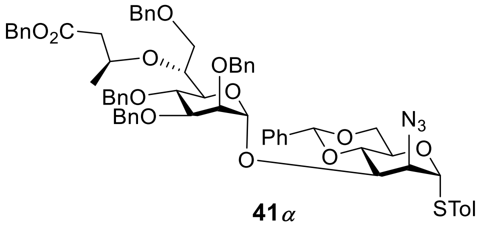

41*α*

^{1}H NMR (600 MHz, Acetone-d_6) δ 7.58 – 7.54 (m, 2H, ArH), 7.46 – 7.13 (m, 32H, ArH), 5.77 (s, 1H, C*H*Ph), 5.38 (d, J = 1.3 Hz, 2H, H-1-Hep, H-1-ManN), 5.15 (d, J = 12.5 Hz, 1H, C*H*H-Bn), 5.05 (d, J = 12.5 Hz, 1H, C*H*H-Bn), 4.86 (d, J = 11.1 Hz, 1H, C*H*H-Bn), 4.67 – 4.60 (m, 3H, 2 × C*H*H-Bn, H-2-ManN), 4.59 – 4.47 (m, 5H, 5 × C*H*H-Bn), 4.43 (dd, J = 9.9, 3.8 Hz, 1H, H-3-ManN), 4.35 – 4.31 (m, 1H, H-5-ManN), 4.25 – 4.19 (m, 1H, H-3-3Hb), 4.17 (t, J = 9.7 Hz, 1H, H-4-ManN), 4.16 – 4.12 (m, 1H, H-6a-ManN), 4.08 – 4.04 (m, 1H, H-6-Hep), 4.03 – 3.94 (m, 4H, H-4-Hep, H-3-Hep, H-5-Hep, H-2-Hep), 3.83 (t, J = 10.2 Hz, 1H, H-6b-ManN), 3.74 (dd, J = 10.0, 5.1 Hz, 1H, H-7a-Hep), 3.62 (dd, J = 10.0, 6.7 Hz, 1H, H-7b-Hep), 2.83 – 2.79 (m, 1H, H-2a-3Hb), 2.53 (dd, J = 14.9, 6.6 Hz, 1H, H-2b-3Hb), 2.32 (s, 3H, C*H$_3$*-STol), 1.26 (d, J = 6.1 Hz, 3H, C*H$_3$*-3Hb); ^{13}C NMR (151 MHz, Acetone-d_6) δ 171.78 (*C*=O-COOBn), 139.91, 139.80, 139.76, 139.56, 139.12, 138.90, 137.53, 133.54, 130.83, 130.23, 129.81, 129.25, 129.08, 129.06, 129.03, 129.00, 128.98, 128.94, 128.76, 128.69, 128.50, 128.37, 128.24, 128.17, 128.15, 128.14, 127.19, 102.53 (*C*HPh), 99.96 (C-1-Hep, $J_{C1\text{-}H1}$ = 171 Hz), 88.29 (C-1-ManN, $J_{C1\text{-}H1}$ = 172 Hz), 80.98, 80.20, 78.19, 75.96, 75.78, 74.97, 74.90, 74.15, 73.95, 73.78, 72.85, 72.56, 71.68, 68.75, 66.57, 65.72, 65.21, 43.13 (C-2-3Hb), 21.38 (*C*H$_3$-STol), 21.09 (*C*H$_3$-3Hb). HRMS (ESI): m/z calcd for $C_{66}H_{69}N_3NaO_{12}S^+$ [M+Na]$^+$ 1150.4494, found 1150.4493.

_p_-Tolyl 2,3,4,7-tetra-_O_-benzyl-6-_O_-[benzyl 3(S)-butanoate]-D-_glycero_-_β_-D-_manno_-heptopyranosyl]-(1→3)-2-azide-4,6-_O_-benzylidene-2-deoxy-thio-_α_-D-mannopyranoside (41_β_)

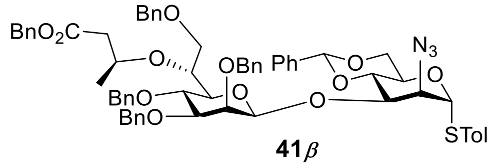

41*β*

^{1}H NMR (500 MHz, Acetone-d_6) δ 7.60 – 7.52 (m, 2H, ArH), 7.47 – 7.19 (m, 32H, ArH), 5.73 (s, 1H, C*H*Ph), 5.56 (d, *J* = 1.4 Hz, 1H, H-1-ManN), 5.15 (d, *J* = 12.6 Hz, 1H, C*H*H-Bn), 5.08 (d, *J* = 12.6 Hz, 1H, C*H*H-Bn), 5.02 (d, *J* = 11.5 Hz, 1H, C*H*H-Bn), 4.96 (s, 1H, H-1-Hep), 4.90 (d, *J* = 11.3 Hz, 1H, C*H*H-Bn), 4.80 – 4.68 (m, 4H, 2 × C*H*H-Bn, H-2-ManN, H-3-ManN), 4.61 (dd, *J* = 13.0, 11.4 Hz, 2H, 2 × C*H*H-Bn), 4.41 (s, 2H, C*H₂*-Bn), 4.38 – 4.27 (m, 1H, H-5-ManN), 4.22 – 4.06 (m, 4H, H-2-Hep, H-3-3Hb, H-4-ManN, H-6a-ManN), 4.05 – 3.99 (m, 2H, H-4-Hep, H-6-Hep), 3.84 (t, *J* = 10.2 Hz, 1H, H-6b-ManN), 3.76 – 3.66 (m, 3H, H-3-Hep, H-5-Hep, H-7a-Hep), 3.62 (dd, *J* = 10.3, 6.9 Hz, 1H, H-7b-Hep), 2.70 (dd, *J* = 15.4, 6.7 Hz, 1H, H-2a-3Hb), 2.50 (dd, *J* = 15.4, 5.9 Hz, 1H, H-2b-3Hb), 2.32 (s, 3H, CH_3-STol), 1.18 (d, *J* = 6.1 Hz, 3H, CH_3-3Hb); ^{13}C NMR (151 MHz, Acetone-d_6) δ 171.63 (*C*=O-COOBn), 140.50, 139.96, 139.90, 139.69, 139.04, 138.99, 137.49, 133.56, 130.79, 130.35, 129.49, 129.23, 129.09, 128.98, 128.95, 128.86, 128.84, 128.75, 128.69, 128.55, 128.52, 128.47, 128.25, 128.19, 128.11, 127.96, 127.78, 127.27, 102.44 (*C*HPh), 98.75 (C-1-Hep, $J_{C1\text{-}H1}$ = 157 Hz), 88.00 (C-1-ManN, $J_{C1\text{-}H1}$ = 172 Hz), 83.58, 78.22, 77.72, 77.49, 76.14, 75.46, 75.09, 74.81, 73.62, 73.20, 72.41, 71.73, 71.56, 68.79, 66.40, 66.12, 63.54, 42.71 (C-2-3Hb), 21.10 (*C*H$_3$-STol), 20.40 (*C*H$_3$-3Hb). HRMS (ESI): m/z calcd for $C_{66}H_{69}N_3NaO_{12}S^+$ [M+Na]$^+$ 1150.4494, found 1150.4494.

_p_-Tolyl 2,3-_O_-isopropylidene-4,7-di-_O_-benzyl-6-_O_-[benzyl 3(_S_)-butanoate]-D-_glycero_-_β_-D-_manno_-heptopyranosyl-(1→3)-2-azide-4,6-_O_-benzylidene-2-deoxy-thio-_α_-D-mannopyranoside (42_β_)

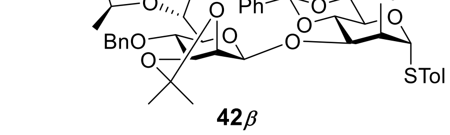

42*β*

^{1}H NMR (600 MHz, Acetone-d_6) δ 7.59 – 7.56 (m, 2H, ArH), 7.43 – 7.37 (m, 2H, ArH), 7.38 – 7.17 (m, 20H, ArH), 5.62 (s, 1H, C*H*Ph), 5.55 (s, 1H, H-1-ManN), 5.16 (d, *J* = 2.6 Hz, 1H, H-1-Hep), 5.12 (s, 2H, CH_2-Bn), 4.76 (d, *J* = 11.7 Hz, 1H, C*H*H-Bn), 4.66 – 4.60 (m, 1H, H-2-ManN), 4.50 (d, *J* = 11.7 Hz, 1H, C*H*H-Bn), 4.46 – 4.41 (m, 3H,

CH_2-Bn, H-3-ManN), 4.35 (t, J = 6.5 Hz, 1H, H-3-Hep), 4.32 – 4.23 (m, 2H. H-2-Hep, H-5-ManN), 4.15 – 4.10 (m, 2H, H-6a-ManN, H-3-3Hb), 4.07 (t, J = 9.7 Hz, 1H, H-4-ManN), 4.05 – 3.95 (m, 2H, H-6-Hep, H-4-Hep), 3.85 – 3.71 (m, 3H, H-6b-ManN, H-7a-Hep, H-5-Hep), 3.66 – 3.58 (m, 1H, H-7b-Hep), 2.64 (dd, J = 15.3, 7.1 Hz, 1H, H-2a-3Hb), 2.47 (dd, J = 15.3, 5.8 Hz, 1H, H-2b-3Hb), 2.32 (s, 3H, CH_3-STol), 1.50 (s, 3H, CH_3-C(CH_3)$_2$), 1.32 (s, 3H, CH_3-C(CH_3)$_2$), 1.19 (d, J = 6.1 Hz, 3H, CH_3-3Hb); ^{13}C NMR (151 MHz, Acetone-d_6) δ 171.66 (C=O-COOBn), 139.93, 139.65, 139.10, 138.95, 137.46, 133.59, 130.81, 130.27, 129.43, 129.29, 129.26, 129.10, 129.03, 128.89, 128.83, 128.78, 128.74, 128.51, 128.47, 128.22, 128.14, 128.03, 127.31, 127.27, 111.01 (C(CH$_3$)$_2$), 102.19 (CHPh), 97.16 (C-1-Hep, J_{C1-H1} = 163 Hz), 88.34 (C-1-ManN, J_{C1-H1} = 170 Hz), 80.07, 78.62, 77.91, 76.40, 76.02, 74.73, 74.55, 73.64, 72.85, 72.51, 71.42, 68.69, 66.50, 66.15, 64.10, 42.69 (C-2-3Hb), 27.32 (C(CH$_3$)$_2$), 25.92 (C(CH$_3$)$_2$), 21.08 (CH$_3$-STol), 20.82 (CH$_3$-3Hb). HRMS (ESI): m/z calcd for $C_{55}H_{61}N_3NaO_{12}S^+$ [M+Na]$^+$ 1010.3868, found 1010.3865.

***p*-Tolyl 2,3-*O*-isopropylidene-4,7-di-*O*-benzyl-6-*O*-[benzyl 3(*S*)-butanoate]-D-*glycero*-*α*-D-*manno*-heptopyranosyl]-(1→3)-2-azide-4,6-*O*-benzylidene-2-deoxy-thio-*α*-D-mannopyranoside (42*α*)**

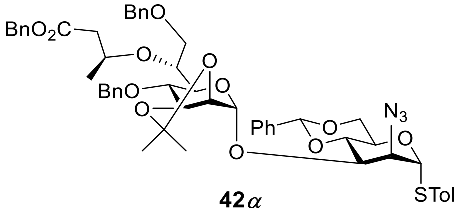

42*α*

^{1}H NMR (600 MHz, Acetone-d_6) δ 7.55 – 7.49 (m, 2H, ArH), 7.46 – 7.23 (m, 20H, ArH), 7.20 (d, J = 8.3 Hz, 2H, ArH), 5.75 (s, 1H, CHPh), 5.38 (s, 1H, H-1-Hep), 5.36 (d, J = 1.3 Hz, 2H, H-1-ManN), 5.16 (d, J = 12.6 Hz, 1H, CHH-Bn), 5.03 (d, J = 12.6 Hz, 1H, CHH-Bn), 4.84 (d, J = 11.6 Hz, 1H, CHH-Bn), 4.74 – 4.62 (m, 2H, CHH-Bn, H-2-ManN), 4.55 (d, J = 11.8 Hz, 1H, CHH-Bn), 4.52 – 4.46 (m, 2H, CHH-Bn, H-3-ManN), 4.37 – 4.31 (m, 1H, H-5-ManN), 4.30 – 4.23 (m, 3H, H-2-Hep, H-5-Hep, H-3-3Hb), 4.18 (t, J = 9.7 Hz, 1H, H-4-ManN), 4.16 – 4.09 (m, 2H, H-6-Hep, H-6a-ManN), 3.95 (dd, J = 10.3, 1.4 Hz, 1H, H-3-Hep), 3.83 (t, J = 10.2 Hz, 1H, H-6b-ManN), 3.73 (dd, J = 9.7, 5.6 Hz, 1H, H-7a-Hep), 3.70 (dd, J = 10.4, 6.6 Hz, 1H, H-4-Hep), 3.65 (dd, J = 9.7, 6.5 Hz, 1H, H-7b-Hep), 2.84 – 2.79 (m, 1H, H-2a-3Hb), 2.57 (dd, J = 14.8, 5.7 Hz, 1H, H-2b-3Hb), 2.32 (s, 3H, CH_3-STol), 1.43 (s, 3H, CH_3-C(CH_3)$_2$), 1.32 (d, J = 6.2 Hz, 3H, CH_3-3Hb), 1.30 (s, 3H, CH_3-C(CH_3)$_2$); ^{13}C NMR (151 MHz, Acetone-d_6) δ 171.77 (C=O-COOBn), 139.55, 138.87, 137.49, 133.55, 130.84, 130.19, 129.71, 129.25, 129.09, 129.02, 128.95, 128.91, 128.80, 128.49, 128.27, 127.14, 110.03 (C(CH$_3$)$_2$), 102.46 (CHPh), 99.42 (C-1-Hep, J_{C1-H1}

= 174 Hz), 88.20 (C-1-ManN, $J_{C1\text{-}H1}$ = 171 Hz), 80.15, 79.89, 78.15, 76.85, 76.42, 74.78, 73.86, 73.72, 73.41, 71.86, 71.52, 68.72, 66.63, 65.59, 64.82, 43.30 (C-2-3Hb), 28.20 (C(CH$_3$)$_2$), 26.52 (C(CH$_3$)$_2$), 21.76 (CH$_3$-STol), 21.10 (CH$_3$-3Hb). HRMS (ESI): m/z calcd for $C_{55}H_{61}N_3NaO_{12}S^+$ [M+Na]$^+$ 1010.3868, found 1010.3864.

6.3 [3+2] coupling strategy for the synthesis of *β*-Hep-containing pentasaccharide

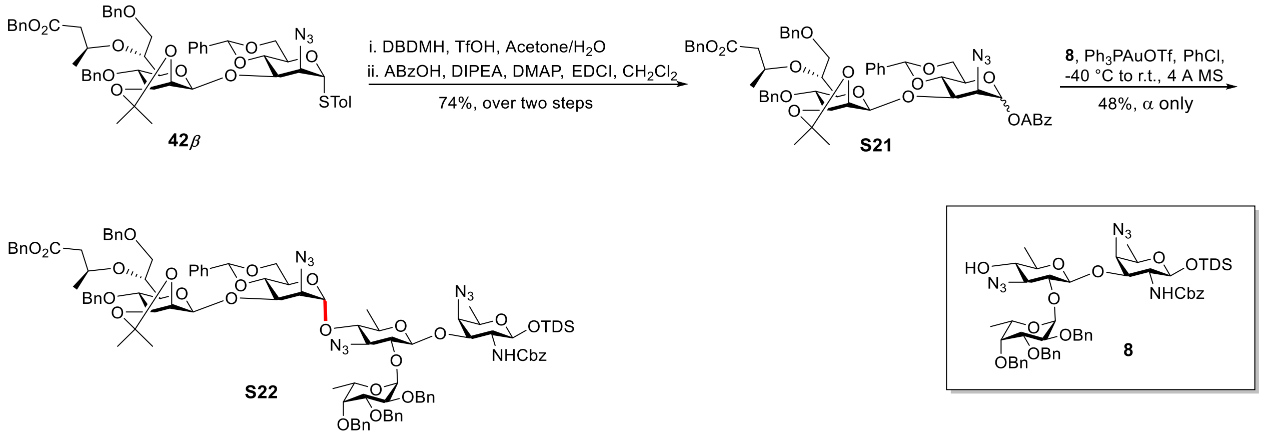

Scheme S15. [3+2] strategy for the synthesis of *β*-Hep-containing pentasaccharide

Dimethylthexylsilyl [2,3-*O*-isopropylidene-4,7-di-*O*-benzyl-6-[benzyl 3*(S)*-butanoate]-D-*glycero*-*β*-D-*manno*-heptopyranosyl]-(1→3)-[2-azide-2-deoxy-4,6-*O*-benzylidene-*α*-D-mannopyranosyl]-(1→4)-[[2,3,4-tri-*O*-benzyl-*α*-L-fucopyranosyl-(1→2)]-3-azide-3,6-di-deoxy-*β*-D-glucopyranosyl]-(1→3)-2-*N*-benzyloxycarbonyl-4-azide-2,4,6-tri-deoxy-*β*-D-galactopyranoside (S22)

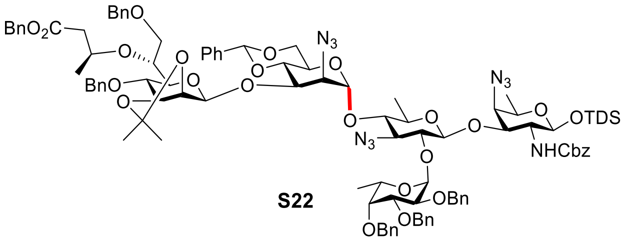

S22

To a solution of compound **42*β*** (42 mg, 42 *μ*mol) in a mixed solvent of acetone/H_2O (v/v, 10/1, 2.2 mL), DBDMH (48 mg, 166 *μ*mol) and TfOH (0.8 *μ*L, 8.4 *μ*mol) were added at 0 °C. The reaction mixture was stirred for 15 min at 0 °C. TLC analysis showed complete conversion of starting material to a hemiacetal intermediate. The reaction was quenched with Et_3N. The organic phase was washed with Na_2SO_3 aqueous, brine, dried (Na_2SO_4), and filtered. The filtrate was concentrated under reduced pressure, and purified by silica gel column chromatography (petroleum ether/ethyl acetate = 2/1) to give hemiacetal intermediate (30 mg) which was used for next step. A solution of

resulting hemiacetal intermediate (30 mg, 34 μmol), *ortho*-hexynylbenzoic acid (10 mg, 51 μmol), DMAP (6 mg, 51 μmol), EDCI (13 mg, 68 μmol) and DIPEA (18 μL, 102 μmol) in anhydrous CH_2Cl_2 (4 mL) was stirred at room temperature for 4 h. TLC analysis showed complete conversion of starting material to ABz donor **S21** (petroleum ether/ethyl acetate = 4/1, R_f =0.75). The mixture was washed with saturated $NaHCO_3$ solution, H_2O, brine, dried (Na_2SO_4), and filtered. The filtrate was concentrated under reduced pressure and purified by preparative thin-layer chromatography (petroleum ether/ethyl acetate = 4/1) to **S21** (32 mg, 74% over two steps) as a colorless oil, which was directly used for the next step.

The ABz donor **S21** (25 mg, 23 μmol) and acceptor **8** (25 mg, 23 μmol) were co-evaporated three times with PhMe. Then, the mixture was dissolved in anhydrous PhCl (1.0 mL) and added freshly activated 4 Å molecular sieves. The mixture was stirred at room temperature for 15 min under an atmosphere of argon and then cooled to -40 °C. After being stirred at -40 °C for 15 min, a solution of $Ph_3PAuOTf$ in PhCl (0.04 M, 0.12 mL, 0.2 equiv) was added. The reaction was gradually warmed to room temperature over 8 h. TLC analysis showed complete conversion of donor to a product **S22** (petroleum ether/ethyl acetate = 2/1, R_f = 0.75) and hydrolysized byproduct. The reaction was quenched by the addition of Et_3N and filtered. The filtrate was concentrated *in vacuo* and purified by size exclusion chromatography (Sephadex LH-20, eluent: CH_2Cl_2/MeOH = 1:1) to give compound **S22** (20 mg, 46%) as a colorless oil. ^{1}H NMR (600 MHz, Acetone-d_6) δ 7.58 (d, J = 6.9 Hz, 2H, ArH), 7.48 – 7.17 (m, 38H, ArH), 6.55 (d, J = 8.9 Hz, 1H, N*H*), 5.60 (s, 1H, C*H*Ph), 5.46 (d, J = 3.5 Hz, 1H, H-1-Fuc), 5.25 – 5.18 (m, 2H, including H-1-ManN), 5.15 – 5.05 (m, 3H, including H-1-Hep), 5.02 – 4.97 (m, 2H), 4.85 – 4.78 (m, 4H, including H-1-AAT), 4.76 – 4.69 (m, 2H), 4.65 (d, J = 11.2 Hz, 1H), 4.59 (d, J = 7.2 Hz, 1H, H-1-ADG), 4.50 (d, J = 11.8 Hz, 1H), 4.45 – 4.38 (m, 5H), 4.36 – 4.30 (m, 3H), 4.25 (dd, J = 7.2, 2.7 Hz, 1H), 4.19 – 4.10 (m, 3H), 4.08 – 3.94 (m, 5H), 3.89 – 3.84 (m, 1H), 3.83 – 3.72 (m, 4H), 3.61 (dd, J = 10.5, 6.8 Hz, 1H), 3.59 – 3.45 (m, 3H), 3.42 (t, J = 7.3 Hz, 1H), 3.30 (t, J = 9.2 Hz, 1H), 2.61 (d, J = 8.2 Hz, 1H, H-2a-3Hb), 2.44 (dd, J = 15.1, 6.0 Hz, 1H, H-2b-3Hb), 1.64 – 1.59 (m, 1H, C*H*(CH_3)$_2$-TDS), 1.50 (s, 3H, C(C*H$_3$*)$_2$), 1.35 – 1.31 (m, 9H, C(C*H$_3$*)$_2$, C*H$_3$*-Fuc, C*H$_3$*-ADG), 1.27 (d, J = 6.3 Hz, 3H, C*H$_3$*-AAT), 1.17 (d, J = 6.1 Hz, 3H, C*H$_3$*-3Hb), 0.88 – 0.69 (m, 12H, CH(C*H$_3$*)$_2$-TDS, C(C*H$_3$*)$_2$-TDS), 0.13 (s, 3H, SiC*H$_3$*-TDS), 0.10 (s, 3H, SiC*H$_3$*-TDS); ^{13}C NMR (126 MHz, Acetone-d_6) δ 171.62 (*C*=O-COOBn), 157.10 (*C*=O-Cbz), 140.51, 140.36, 140.05, 139.66, 138.95, 137.49, 129.32, 129.26, 129.10, 129.04, 129.00, 128.91, 128.83, 128.79, 128.76, 128.66, 128.58, 128.52, 128.44, 128.21, 128.11, 128.07, 128.02, 127.97, 127.21, 110.96 (*C*(CH_3)$_2$), 103.57 (C-1-ADG, $J_{C1\text{-}H1}$ = 163 Hz), 101.99 (*C*HPh; C-1-ManN, $J_{C1\text{-}H1}$ = 175 Hz), 98.45 (C-1-Fuc, $J_{C1\text{-}H1}$ = 171 Hz), 97.60 (C-1-Hep, $J_{C1\text{-}H1}$ = 158 Hz), 97.39 (C-1-AAT, $J_{C1\text{-}H1}$ = 161 Hz), 83.17, 80.17, 79.63, 79.43,

78.47, 78.30, 78.22, 77.93, 77.19, 76.38, 75.67, 75.17, 74.29, 73.61, 73.50, 73.23, 72.80, 72.65, 71.85, 71.53, 69.90, 69.63, 68.72, 68.42, 67.24, 66.68, 66.49, 65.97, 63.57, 56.45, 42.75 (*C*H2-3Hb), 34.75 (C(*C*H3)2-TDS), 27.31 (C(*C*H3)2), 25.79 (C(*C*H3)2), 25.52 (*C*(CH3)2-TDS), 20.98 (*C*H3-3Hb), 20.49 (C(*C*H3)2-TDS), 20.39 (C(*C*H3)2-TDS), 18.96 (CH(*C*H3)2-TDS), 18.89 (CH(*C*H3)2-TDS), 18.49 (C-6-ADG), 17.84 (C-6-AAT), 16.82 (C-6-Fuc), -1.69 (Si*C*H3-TDS), -2.99 (Si*C*H3-TDS). HRMS (ESI): m/z calcd for $C_{103}H_{126}N_{10}NaO_{24}Si^+$ $[M+Na]^+$ 1937.8608, found 1937.8611.

6.4 [4+1] coupling strategy for the synthesis of *β*-Hep-containing pentasaccharide

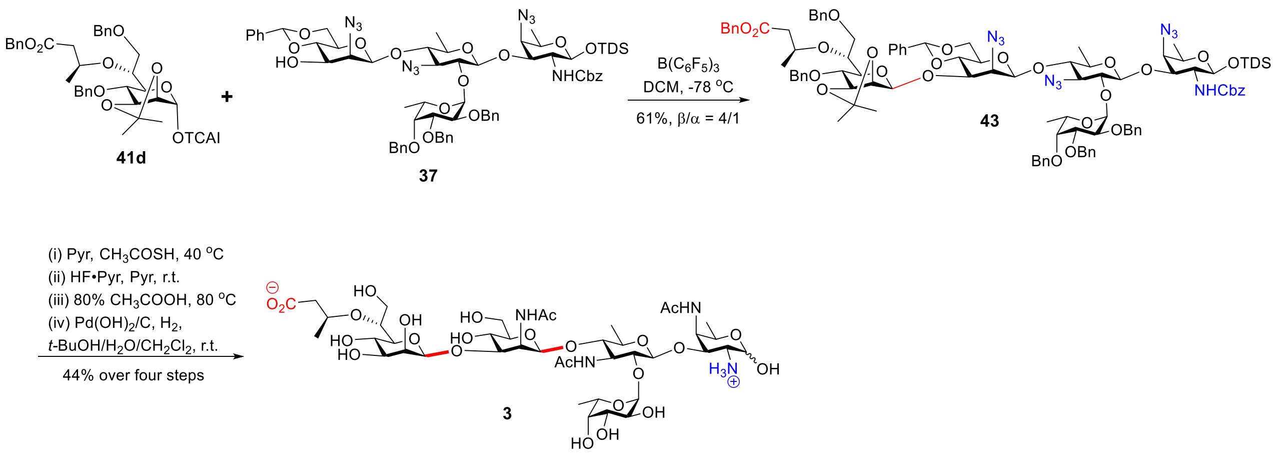

Scheme S16. Synthesis of *β*-Hep-containing pentasaccharide **3**

Dimethylthexylsilyl [2,3-*O*-isopropylidene-4,7-di-*O*-benzyl-6-*O*-[benzyl 3(S)-butanoate]-D-*glycero*-*β*-D-*manno*-heptopyranosyl]-(1→3)-[2-azide-4,6-*O*-benzylidene-2-deoxy-*β*-D-mannopyranosyl]-(1→4)-[[2,3,4-tri-*O*-benzyl-*α*-L-fucopyranosyl-(1→2)]-3-azide-3,6-di-deoxy-*β*-D-glucopyranosyl]-(1→3)-2-*N*-benzyloxycarbonyl-4-azide-2,4,6-tri-deoxy-*β*-D-galactopyranoside (43)

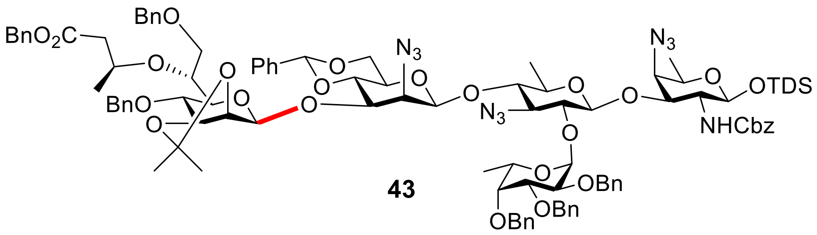

43

The Hep-TCAI donor **41d** (56 mg, 75 *μ*mol) and acceptor **37** (50 mg, 37 *μ*mol) were co-evaporated three times with PhMe. Then, the mixture was dissolved in anhydrous CH_2Cl_2 (1 mL) and added freshly activated 4 Å molecular sieves. The mixture was stirred at room temperature for 15 min under an atmosphere of argon and then cooled to -

78 ºC. After being stirred at -78 ºC for 15 min, $B(C_6F_5)_3$ (7 mg, 15 μmol) was added. Then, the reaction was stirred at -78 ºC for 5 min. TLC analysis showed complete conversion of acceptor to **43** (petroleum ether/ethyl acetate = 4/1, R_f = 0.35) and α-isomer (petroleum ether/ethyl acetate = 4:1, R_f = 0.55). The reaction was quenched by the addition of Et_3N and filtered. The filtrate was concentrated *in vacuo* and purified by silica gel column chromatography (petroleum ether/ethyl acetate/CH_2Cl_2 = 10/1/1 to 6/1/1) to give compound **43** (43 mg, 61%) and α-isomer (11 mg, 15%) as colorless oil. **43**: ^{1}H NMR (400 MHz, Acetone-d_6) δ 7.61 – 7.53 (m, 2H), 7.48 – 7.43 (m, 2H), 7.41 – 7.17 (m, 36H), 6.55 (d, J = 9.0 Hz, 1H, N*H*), 5.60 (s, 1H, C*H*Ph), 5.53 (d, J = 3.3 Hz, 1H, H-1-Fuc), 5.20 (d, J = 12.7 Hz, 1H), 5.16 – 5.06 (m, 4H, including H-1-ManN, H-1-Hep), 4.99 (t, J = 10.9 Hz, 2H), 4.87 (t, J = 10.5 Hz, 2H), 4.81 – 4.71 (m, 4H, including H-1-AAT), 4.68 – 7.64 (m, 2H, including H-1-ADG), 4.53 (d, J = 4.7 Hz, 1H), 4.50 – 4.43 (m, 2H), 4.39 – 4.29 (m, 5H), 4.27 – 4.20 (m, 2H), 4.17 – 4.04 (m, 3H), 4.04 – 3.95 (m, 4H), 3.93 – 3.76 (m, 3H), 3.74 – 3.68 (m, 2H), 3.63 – 3.41 (m, 7H), 2.58 (dd, J = 15.0, 7.3 Hz, 1H, H-2a-3Hb), 2.45 (dd, J = 15.0, 5.6 Hz, 1H, H-2b-3Hb), 1.65 – 1.59 (m, 1H, $CH(CH_3)_2$-TDS), 1.51 (s, 3H, $C(CH_3)_2$), 1.39 – 1.30 (m, 9H, $(CH_3)_2$, CH_3-Fuc, CH_3-ADG), 1.27 (d, J = 6.2 Hz, 3H, CH_3-AAT), 1.17 (d, J = 6.2 Hz, 3H, CH_3-3Hb), 0.94 – 0.77 (m, 12H, $CH(CH_3)_2$-TDS, $C(CH_3)_2$-TDS), 0.13 (s, 3H, $SiCH_3$-TDS), 0.11 (s, 3H, $SiCH_3$-TDS); ^{13}C NMR (126 MHz, Acetone-d_6) δ 171.66 (*C*=O-COOBn), 157.07 (*C*=O-Cbz), 140.54, 140.43, 140.10, 139.98, 139.61, 138.96, 137.44, 129.42, 129.28, 129.25, 129.13, 129.02, 129.00, 128.96, 128.94, 128.86, 128.81, 128.75, 128.66, 128.59, 128.56, 128.53, 128.49, 128.45, 128.29, 128.22, 128.19, 128.10, 128.04, 127.99, 127.97, 127.88, 127.84, 127.23, 111.25 ($C(CH_3)_2$), 103.44 (C-1-ADG, $J_{C1\text{-}H1}$ = 161 Hz), 102.23 (*C*HPh), 101.35 (C-1-ManN, $J_{C1\text{-}H1}$ = 162 Hz), 98.12 (C-1-Fuc, $J_{C1\text{-}H1}$ = 172 Hz), 97.52 (C-1-AAT, $J_{C1\text{-}H1}$ = 160 Hz), 95.82 (C-1-Hep, $J_{C1\text{-}H1}$ = 163 Hz), 82.84 , 80.18, 80.13, 79.59, 78.25, 77.95, 77.83, 77.26, 76.88, 76.32, 76.06, 75.69, 74.74, 74.52, 73.47, 73.26, 73.15, 72.80, 72.69, 72.13, 71.42, 69.54, 68.98, 68.91, 68.13, 68.04, 67.52, 66.70, 66.46, 63.32, 56.37, 42.84 (CH_2-3Hb), 34.68 ($C(CH_3)_2$-TDS), 27.15 ($C(CH_3)_2$), 26.14 ($C(CH_3)_2$), 25.49 ($C(CH_3)_2$-TDS), 20.88 (CH_3-3Hb), 20.48 ($C(CH_3)_2$-TDS), 20.36 ($C(CH_3)_2$-TDS), 18.95 ($CH(CH_3)_2$-TDS), 18.86 ($CH(CH_3)_2$-TDS), 18.30 (C-6-ADG), 17.85 (C-6-AAT), 16.73 (C-6-Fuc), -1.71 ($SiCH_3$-TDS), -2.95 ($SiCH_3$-TDS). HRMS (ESI): m/z calcd for $C_{103}H_{126}N_{10}NaO_{24}Si^+$ $[M+Na]^+$ 1937.8608, found 1937.8612.

[6-*O*-[3(*S*)-butanoic acid]-D-*glycero*-*β*-D-*manno*-heptopyranosyl]-(1→3)-[2-*N*-acetyl-2-deoxy-*β*-D-mannopyranosyl]-(1→4)-[[*α*-L-fucopyranosyl-(1→2)]-3-*N*-acetyl-3,6-di-deoxy-*β*-D-glucopyranosyl]-(1→3)-2-amino-4-*N*-acetyl-2,4,6-tri-deoxy-*α*/*β*-D-galactopyranoside (3)

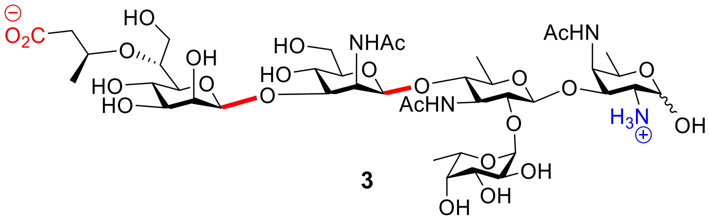

3

The fully protected compound **43** (18 mg, 11 *μ*mol) was dissolved in a mixed solvent of pyridine/AcSH (1/1, v/v, 1 mL) and the mixture was stirred at 40 °C for 48 h under an atmosphere of argon. The ESI-MS analysis showed conversion of starting material to a major NHAc product. The reaction was concentrated *in vacuo* and the crude product was co-evaporated three times with PhMe. The resulting residue was purified by size exclusion chromatography (Sephadex LH-20, eluent: DCM/MeOH = 1:1) to give NHAc intermediate. The NHAc intermediate was dissolved in pyridine (2 mL), followed by the addition of HF/Pyridine (70%, 0.2 mL) at 0 °C. The reaction was stirred at room temperature overnight. TLC analysis showed complete conversion of starting material to a major hemiacetal product (CH_2Cl_2/MeOH/ethyl acetate = 15/0.5/1, R_f = 0.35). The reaction was quenched with saturated $NaHCO_3$ solution and diluted with CH_2Cl_2. The organic phase was separated, and washed with brine and dried over Na_2SO_4. The filtration was concentrated under reduced pressure to give a hemiacetal intermediate. The hemiacetal product was treated with 80% AcOH in water (2 mL) and the mixture was heated to 80 °C for 0.5 h. ESI-MS analysis showed the benzylidene and isopropylidene groups were completely removed. The reaction mixture was cooled to room temperature and concentrated under reduced pressure to give crude product. The resulting crude product was purified by size exclusion chromatography (Sephadex LH-20, eluent: DCM/MeOH = 1/1). A solution of resulting product in *t*-BuOH/H_2O/CH_2Cl_2 (3/2/0.5, v/v/v, 5.5 mL) was added $Pd(OH)_2$/C (20%, 20 mg). The mixture was stirred at room temperature overnight under H_2 atmosphere. ESI-MS analysis showed the complete conversion of starting material to a major product **3**. The reaction mixture was filtered through celite, and filtration was concentrated under reduced pressure to give a crude product, which was sequentially purified by reverse-phase silica column (C-18, eluent: 1% CH_3CN in H_2O to 20%) and size-exclusion chromatography (BioGel P-4, 45−90 *μ*m, eluent: 0.1 M NH_4HCO_3). The product-containing fractions were combined and lyophilized to afford the product **2** as a white amorphous solid (4.2 mg, 44% over four steps).

^{1}H NMR (600 MHz, D_2O) δ 5.41 (d, J = 3.9 Hz, 0.6H, H-1-AAT-α), 5.09 (s, 1H, H-1-Fuc), 4.87 – 4.81 (m, 1.4H, H-1-AAT-β, H-1-ManNAc), 4.73 (d, J = 7.7 Hz, 1H, H-1-ADG), 4.70 (s, 1H, H-1-Hep), 4.58 (dd, J = 4.4, 1.5 Hz, 1H, H-2-ManNAc), 4.55 – 4.49 (m, 1H, H-4-AAT), 4.46 – 4.35 (m, 1.3H, H-3-AAT-α, H-5-AAT-α), 4.28 – 4.21 (m, 1H, H-5-Fuc), 4.18 (dd, J = 11.2, 4.7 Hz, 0.5H, H-3-AAT-β), 4.10 – 4.01 (m, 2H, H-3-ManNAc, H-3-3Hb), 3.99 – 3.85 (m, 3.4H, H-3-ADG, H-6a-ManNAc, H-6-Hep, H-5-AAT-β), 3.84 (dd, J = 12.0, 4.7 Hz, 1H, H-6b-ManNAc), 3.82 – 3.66 (m, 7H), 3.64 – 3.49 (m, 5H), 3.48 – 3.37 (m, 3H, H-2-ADG, H-5-ManNAc, H-4-ADG), 3.29 – 3.20 (m, 0.4H, H-2-AAT-β), 2.53 (dd, J = 13.9, 7.0 Hz, 1H, H-2a-3Hb), 2.30 (dd, J = 13.9, 6.7 Hz, 1H, H-2b-3Hb), 2.08 (d, J = 4.0 Hz, 3H, CH_3-NHAc-AAT-α/β), 2.05 (s, 3H, CH_3-NHAc), 2.02 (s, 3H, CH_3-NHAc), 1.29 – 1.27 (m, 3H, CH_3-ADG), 1.23 – 1.20 (m, 6H, CH_3-3Hb, CH_3-Fuc), 1.14 – 1.07 (m, 3H, CH_3-AAT-α/β); ^{13}C NMR (151 MHz, D_2O) δ 179.92 (C=O-COOH), 174.78 (C=O-NHAc), 174.66 (C=O-NHAc), 174.44 (C=O-NHAc), 103.02 (C-1-ADG, J_{C1-H1} = 163 Hz), 99.20 (C-1-Fuc, J_{C1-H1} = 170 Hz), 99.03 (C-1-ManNAc, J_{C1-H1} = 164 Hz), 97.10 (C-1-Hep, J_{C1-H1} = 157 Hz), 93.12 (C-1-AAT-β), 89.07 (C-1-AAT-α, J_{C1-H1} = 169 Hz), 80.67, 79.71, 77.63, 77.40, 76.30, 76.20, 74.16, 73.20, 72.33, 72.09, 71.66, 70.60, 70.33, 69.27, 67.97, 67.32, 65.55, 65.16, 60.82, 60.15, 54.69, 53.86, 52.94, 50.59, 50.20, 45.51 (C-2-3Hb), 22.03 (CH3-NHAc), 21.98 (CH3-NHAc), 21.96 (CH3-NHAc), 19.61 (CH3-3Hb), 16.91 (CH3-ADG), 15.70 (CH3-AAT), 15.62 (CH3-Fuc). HRMS (ESI): m/z calcd for $C_{41}H_{70}N_4NaO_{25}^+$ [M+Na]$^+$ 1041.4221, found 1041.4222.

7. Synthesis of trisaccharides 4 and 5

7.1 synthesis of compound 4

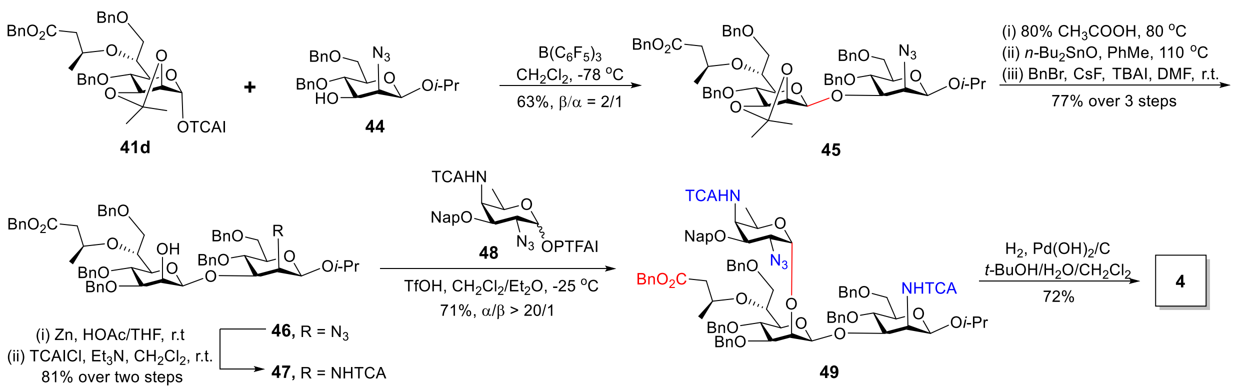

Scheme S17. Synthesis of trisaccharide **4**

Isopropyl 2,3-*O*-isopropylidene-4,7-di-*O*-benzyl-6-*O*-[benzyl 3(*S*)-butanoate]-D-*glycero*-*β*-D-*manno*-heptopyranosyl-(1→3)-2-azide-2-deoxy-4,6-di-*O*-benzyl-*β*-D-mannopyranoside (45)

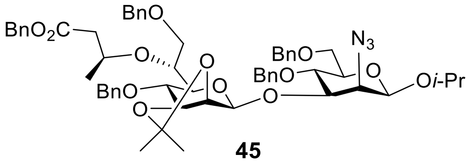

45

The Hep-TCAI donor **41d** (180 mg, 0.24 mmol) and acceptor **44** (123 mg, 0.29 mmol) were co-evaporated three times with PhMe. Then, the mixture was dissolved in anhydrous CH_2Cl_2 (6 mL) and added freshly activated 4 Å molecular sieves. The mixture was stirred at room temperature for 15 min under an atmosphere of argon and then cooled to -78 ºC. After being stirred at -78 ºC for 15 min, $B(C_6F_5)_3$ (24 mg, 0.048 mmol) was added. Then, the reaction was stirred at -78 ºC for 15 min. TLC analysis showed complete conversion of donor to **45** (petroleum ether/ethyl acetate = 4/1, R_f = 0.35) and *α*-isomer (petroleum ether/ethyl acetate = 4/1, R_f = 0.45). The reaction was quenched by the addition of Et_3N and filtered. The filtrate was concentrated *in vacuo* and purified by silica gel column chromatography (petroleum ether/ethyl acetate = 10/1 to 8/1) to give compound **45** (152 mg, 63%) as a colorless oil. ^{1}H NMR (600 MHz, $CDCl_3$) *δ* 7.38 – 7.04 (m, 25H, ArH), 5.09 – 4.95 (m, 3H, 3 × C*H*H-Bn), 4.84 (s, 1H, H-1-Hep), 4.63 (d, *J* = 11.7 Hz, 1H, C*H*H-Bn), 4.56 – 4.41 (m, 5H, H-1-ManN, 4 × C*H*H-Bn), 4.35 – 4.32 (m, 2H, H-3-Hep, C*H*H-Bn), 4.28 (d, *J* = 12.1 Hz, 1H, C*H*H-Bn), 4.24 (d, *J* = 6.7 Hz, 1H, H-2-Hep), 4.09 – 4.00 (m, 2H, H-3-ManN, H-3-3Hb), 3.98 – 3.96 (m, 2H, H-2-ManN, OC*H*-Pr), 3.83 – 3.78 (m, 1H, H-6-Hep), 3.77 – 3.67 (m, 3H, H-4-Hep, H-5-Hep, H-6a-ManN), 3.65 (t, *J* = 9.4 Hz, 1H, H-4-ManN), 3.60 (dd, *J* = 10.8, 6.0 Hz, 1H, H-6b-ManN), 3.56 (dd, *J* = 10.7, 3.2 Hz, 1H, H-7a-Hep), 3.46 (dd, *J* = 10.5, 5.9 Hz, 1H, H-7b-Hep), 3.35 – 3.30 (m, 1H, H-5-ManN), 2.55 (dd, *J* = 15.2, 7.4 Hz, 1H, H-2a-3Hb), 2.30 (dd, *J* = 15.2, 5.3 Hz, 1H, H-2b-3Hb), 1.40 (s, 3H, CH_3-$C(CH_3)_2$), 1.31 (s, 3H, CH_3-$C(CH_3)_2$), 1.22 (d, *J* = 6.3 Hz, 3H, CH_3-3Hb), 1.15 (d, *J* = 6.3 Hz, 3H, CH_3-Pr), 1.11 (d, *J* = 6.1 Hz, 3H, CH_3-Pr); ^{13}C NMR (126 MHz, $CDCl_3$) *δ* 171.18 (*C*=O-COOBn), 138.93, 138.66, 138.51, 138.15, 135.98, 128.63, 128.60, 128.57, 128.53, 128.50, 128.40, 128.32, 128.28, 128.24, 128.16, 128.08, 128.05, 128.02, 128.00, 127.96, 127.88, 127.85, 127.82, 127.77, 127.73, 127.64, 127.56, 127.53, 127.49, 127.37, 110.63 ($C(CH_3)_2$), 97.36 (C-1-ManN, J_{C1-H1} = 155 Hz), 93.94 (C-1-Hep, J_{C1-H1} = 160 Hz), 78.39, 77.92, 77.36, 76.68, 76.20, 75.67, 74.37, 74.00, 73.58, 73.45, 73.21, 73.04, 71.89, 71.63, 71.30, 69.67, 69.61, 66.08, 61.11, 41.99 (C-2-3Hb), 27.06 ($C(CH_3)_2$), 25.69 ($C(CH_3)_2$), 23.47 (CH_3-3Hb), 21.71 (CH_3-Pr), 20.78 (CH_3-Pr). HRMS (ESI): m/z calcd for $C_{58}H_{69}N_3NaO_{13}^+$ $[M+Na]^+$ 1038.4723, found 1038.4728.

Isopropyl 3,4,7-tri-*O*-benzyl-6-*O*-[benzyl 3(*S*)-butanoate]-D-*glycero*-*β*-D-*manno*-heptopyranosyl-(1→3)-2-azide-4,6-di-*O*-benzyl-2-deoxy-*β*-D-mannopyranoside (46)

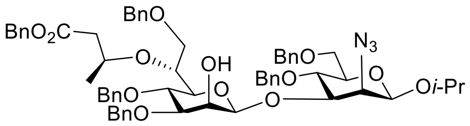

46

The compound **45** (85mg, 84 μmol) was treated with 80% AcOH in water (5 mL) and the mixture was heated to 80 °C for 8 h. TLC analysis showed complete conversion of starting material **45** to diol intermediate (petroleum ether/ethyl acetate = 1/1, R_f = 0.25). The reaction mixture was cooled to room temperature and concentrated under reduced pressure to give crude product. The resulting crude product was purified by silica gel column chromatography (petroleum ether/ethyl acetate = 4/1 to 2/1) to give diol intermediate (75 mg) as a colorless oil.

A mixture of above diol intermediate (75 mg, 77 μmol) and dibutyltin oxide (23 mg, 92 μmol) in PhMe (5 mL) was heated to 110 °C and stirred for 6 h. The reaction mixture was cooled to room temperature, and concentrated under reduced pressure to give a colorless oil for the next step. The resulting oil was dissolved in DMF (5 mL), and the BnBr (10 μL, 92 μmol) and cesium fluoride (CsF, 14 mg, 92 μmol) were added. The reaction mixture was stirred at room temperature overnight under an atmosphere of argon. TLC analysis showed complete conversion to compound **46** (petroleum ether/ethyl acetate = 1/1, R_f = 0.80). The mixture was concentrated *in vacuo*. The resulting residue was dissolved with CH_2Cl_2, and the mixture was washed with 1 M HCl and brine. The organic phase was dried (Na_2SO_4), filtered, and the filtrate was concentrated *in vacuo*. The resulting residue was purified by silica gel column chromatography (petroleum ether/ethyl acetate = 10/1 to 8/1) to give compound **46** (70 mg, 77% over three steps) as a colorless oil. ^1H NMR (500 MHz, $CDCl_3$) δ 7.70 – 6.82 (m, 30H, ArH), 5.18 (s, 2H), 5.08 (d, *J* = 11.5 Hz, 1H), 4.92 (d, *J* = 11.0 Hz, 1H), 4.81 (d, *J* = 11.7 Hz, 1H), 4.72 (s, 1H), 4.70 – 4.52 (m, 6H), 4.42 – 4.29 (m, 3H), 4.19 – 4.04 (m, 3H), 4.01 (d, *J* = 3.5 Hz, 1H), 3.98 – 3.91 (m, 2H), 3.86 – 3.71 (m, 3H), 3.63 – 3.46 (m, 5H), 3.21 (s, 1H), 2.56 (dd, *J* = 15.4, 8.7 Hz, 1H, H-2a-3Hb), 2.27 (dd, *J* = 15.4, 4.2 Hz, 1H, H-2b-3Hb), 1.33 (d, *J* = 6.3 Hz, 3H, CH_3-3Hb), 1.21 (d, *J* = 6.1 Hz, 3H, CH_3-Pr), 1.12 (d, *J* = 6.0 Hz, 3H, CH_3-Pr); ^{13}C NMR (126 MHz, $CDCl_3$) δ 171.80 (*C*=O-COOBn), 138.63, 138.55, 138.43, 138.38, 138.00, 135.90, 128.62, 128.51, 128.38, 128.35, 128.30, 128.28, 128.23, 128.10, 127.91, 127.85, 127.81, 127.61, 127.58, 127.51, 127.47, 97.40, 96.45, 82.00, 76.26, 75.63, 74.73, 74.71, 73.79, 73.75, 73.54, 73.28, 72.27, 71.35, 70.92, 70.78, 69.55, 68.02, 66.45, 61.66, 42.16, 23.51, 21.72, 19.82. HRMS (ESI): m/z calcd for $C_{62}H_{71}N_3NaO_{13}^+$ $[M+Na]^+$ 1088.4879, found 1088.4881.

Isopropyl 2-azide-4-trichloroacetamido-2,4,6-tri-deoxy-*α*-D-galactopyranosyl-(1→2)-[3,4,7-tri-*O*-benzyl-6-*O*-[benzyl 3(*S*)-butanoate]-D-*glycero*-*β*-D-*manno*-heptopyranosyl]-(1→3)-2-azide-2-deoxy-4,6-di-*O*-benzyl-*β*-D-mannopyranoside (49)

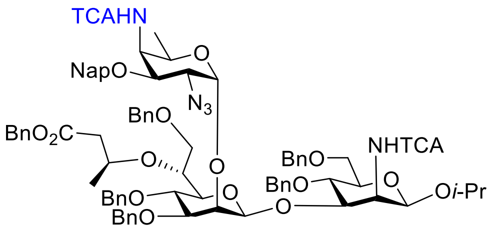

49

To a solution of **46** (20 mg, 19 μmol) in a mixed solvent of THF/HOAc (v/v, 3/1, 2.0 mL) was added Zn powder (50 mg). The reaction was stirred at room temperature for 5 h. TLC analysis showed complete conversion of starting material to amine intermediate (CH_2Cl_2/MeOH = 15/1, R_f = 0.25). The reaction was filtered through celite. The filtrate was concentrated, diluted with CH_2Cl_2, and washed with saturated $NaHCO_3$ solution. The organic layer was dried over Na_2SO_4 and concentrated under reduced pressure to afford amine intermediate for the next step. To a solution of above amine intermediate in a mixed solvent of THF/Et_3N (v/v, 10/1, 5.5 mL) was added TCACl (3.2 μL, 28 μmol) at 0 °C. The reaction was stirred at room temperature for 0.5 h. TLC analysis showed complete conversion to compound **47** (petroleum ether/ethyl acetate = 4/1, R_f = 0.50). The reaction was quenched by adding H_2O and diluted with CH_2Cl_2. The organic phase was washed with 1 M HCl, saturated aqueous $NaHCO_3$ and brine. The organic phase was dried (Na_2SO_4), and filtered. The filtrate was concentrated *in vacuo* and purified by silica gel column chromatography (petroleum ether/ethyl acetate = 8/1 to 6/1) to give compound **47** (18 mg, 81% over two steps) as a colorless oil. HRMS (ESI): m/z calcd for $C_{64}H_{76}Cl_3N_2O_{14}^+$ $[M+NH_4]^+$ 1201.4357, found 1201.4357.
The AAT PTFAI donor **48** (13 mg, 20 μmol) and acceptor **47** (12 mg, 10 μmol) were co-evaporated three times with PhMe. Then, the mixture was dissolved in a mixed solvent of anhydrous CH_2Cl_2/Et_2O(2/1. v/v, 3 mL) and added freshly activated 4 Å molecular sieves. The mixture was stirred at room temperature for 15 min under an atmosphere of argon and then cooled to -25 °C. After being stirred at -25 °C for 15 min, TfOH (0.2 μL, 2.0 μmol) was added. Then, the reaction was stirred at -25 °C for 30 min. TLC analysis showed complete conversion of acceptor **47** to product **49** (petroleum ether/ethyl acetate = 4/1, R_f = 0.55). The reaction was quenched by the addition of Et_3N and filtered. The filtrate was concentrated *in vacuo* and resulting residue was purified by silica gel column chromatography (petroleum ether/ethyl acetate/CH_2Cl_2 = 15/1/1 to 8/1/1) to give compound **49** (12 mg, 72%) as a colorless oil. ^1H NMR (800 MHz, $CDCl_3$) δ 7.80 – 7.70 (m, 3H, ArH), 7.64 – 7.60 (m, 1H, ArH), 7.46 – 7.38 (m, 5H, ArH), 7.36 – 7.19 (m, 28H, ArH), 7.16 (d, J = 8.6 Hz, 1H, N*H*-NHTCA), 6.71 (d, J = 9.8 Hz, 1H, N*H*-NHTCA),

5.38 (d, J = 3.9 Hz, 1H, H-1-AAT), 5.11 – 5.01 (m, 2H, CH_2-Bn), 4.89 (d, J = 11.2 Hz, 1H, CHH-Bn), 4.87 (s, 1H, H-1-Hep), 4.85 (d, J = 10.3 Hz, 1H, CHH-Bn), 4.82 (d, J = 1.4 Hz, 1H, H-1-ManN), 4.78 (d, J = 12.2 Hz, 1H, CHH-Bn), 4.77 – 4.73 (m, 1H, H-5-AAT), 4.71 (d, J = 12.1 Hz, 1H, CHH-Bn), 4.69 – 4.65 (m, 2H, 2 × CHH-Bn), 4.63 – 4.59 (m, 1H, H-2-ManN), 4.56 (d, J = 11.8 Hz, 1H, CHH-Bn), 4.47 – 4.44 (m, 2H, CHH-Bn, H-3-ManN), 4.43 – 4.39 (m, 3H, 2 × CHH-Bn, CHH-Nap), 4.24 (d, J = 3.0 Hz, 1H, H-2-Hep), 4.19 – 4.14 (m, 1H, H-4-AAT), 4.11 – 4.08 (m, 1H, H-3-3Hb), 4.02 (t, J = 9.6 Hz, 1H, H-4-Hep), 4.00 – 3.95 (m, 2H, CH-Pr, H-6-Hep), 3.85 (dd, J = 10.6, 2.9 Hz, 1H, H-6a-ManN), 3.82 (dd, J = 10.8, 3.5 Hz, 1H, H-7a-Hep), 3.80 – 3.76 (m, 2H, H-7b-Hep, H-3-AAT), 3.75 – 3.71 (m, 2H, H-4-ManN, H-6b-ManN), 3.68 – 3.63 (m, 2H, H-3-Hep, CHH-Nap), 3.54 – 3.51 (m, 2H, H-5-ManN, H-5-Hep), 3.21 (dd, J = 10.9, 3.9 Hz, 1H, H-2-AAT), 2.63 (dd, J = 14.6, 8.4 Hz, 1H, H-2a-3Hb), 2.47 (dd, J = 14.6, 4.6 Hz, 1H, H-2b-3Hb), 1.22 (d, J = 6.2 Hz, 3H, CH_3-3Hb), 1.20 (d, J = 6.2 Hz, 3H, CH_3-Pr), 1.13 (d, J = 6.5 Hz, 3H, CH_3-AAT), 1.10 (d, J = 6.2 Hz, 3H, CH_3-Pr). ^{13}C NMR (201 MHz, $CDCl_3$) δ 171.24 (C=O-COOBn), 163.11 (C=O-TCA), 162.80 (C=O-TCA), 138.92, 138.33, 138.20, 137.95, 135.92, 134.90, 133.38, 133.10, 128.84, 128.75, 128.72, 128.63, 128.60, 128.50, 128.45, 128.37, 128.34, 128.32, 128.26, 128.14, 128.12, 128.07, 127.99, 127.93, 127.83, 127.80, 127.75, 127.72, 127.54, 127.41, 127.31, 126.96, 126.21, 125.96, 125.87, 97.36 (C-1-AAT, $J_{C1\text{-}H1}$ = 175 Hz), 96.40 (C-1-Hep, $J_{C1\text{-}H1}$ = 157 Hz), 96.37 (C-1-ManN, $J_{C1\text{-}H1}$ = 160 Hz), 92.70 (CCl$_3$), 92.67 (CCl$_3$), 83.73, 77.36, 76.36, 74.93, 74.88, 74.86, 74.45, 73.88, 73.55, 73.51, 73.30, 71.99, 71.66, 71.53, 71.21, 70.74, 70.54, 68.79, 66.17, 64.07, 59.82, 52.38 (C-3-3Hb), 52.24 (OCH-Pr), 42.77 (C-2-3Hb), 23.35 (CH$_3$-Pr), 21.63 (CH$_3$-Pr), 20.27 (CH$_3$-3Hb), 16.86 (CH$_3$-AAT). HRMS (ESI): m/z calcd for $C_{83}H_{93}Cl_6N_6O_{17}^+$ $[M+NH_4]^+$ 1655.4723, found 1655.4727.

Isopropyl 2-amino-4-*N*-acetyl-2,4,6-tri-deoxy-*α*-D-galactopyranosyl-(1→2)-[6-*O*-[3(*S*)-butanoic acid]-D-*glycero*-*β*-D-*manno*-heptopyranosyl]-(1→3)-2-*N*-acetyl-2-deoxy-*β*-D-mannopyranoside (4)

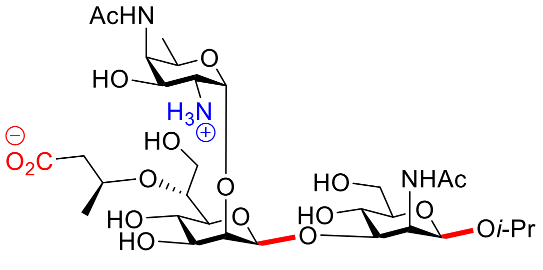

4

To a solution of the **49** (12 mg, 7.3 μmol)in t-BuOH/H_2O/CH_2Cl_2 (4/1/1, v/v/v, 3 mL) was added $Pd(OH)_2$/C (20%,

40 mg). The mixture was stirred at room temperature for 48 h under H_2 atmosphere. ESI-MS analysis showed the complete conversion of starting material to a major product **4**. The reaction mixture was filtered through celite, and filtration was concentrated under reduced pressure to give a crude product, which was sequentially purified by reverse-phase silica column (C-18, eluent: 20% CH_3CN in H_2O to 30%) and size-exclusion chromatography (BioGel P-4, 45−90 μm, eluent: 0.1 M NH_4HCO_3). The product-containing fractions were combined and lyophilized to afford the product **4** as a white amorphous solid (4.3 mg, 72%). ^{1}H NMR (600 MHz, D_2O) δ 5.29 (d, J = 4.1 Hz, 1H, H-1-AAT), 4.90 (d, J = 1.7 Hz, 1H, H-1-ManNAc), 4.76 (s, 1H, H-1-Hep), 4.61 – 4.52 (m, 2H, H-5-AAT, H-2-ManN), 4.37 (dd, J = 11.3, 4.5 Hz, 1H, H-3-AAT), 4.26 (dd, J = 4.5, 1.8 Hz, 1H, H-4-AAT), 4.17 – 4.12 (m, 1H, H-3-3Hb), 4.11 – 4.04 (m, 1H, C*H*-Pr), 4.01 (dd, J = 9.8, 4.3 Hz, 1H, H-3-ManN), 3.92 – 3.86 (m, 3H, H-2-Hep, H-6-Hep, H-6a-ManNAc), 3.85 – 3.78 (m, 3H, H-6b-ManNAc, H-4-Hep, H-7a-Hep), 3.75 (dd, J = 9.6, 3.3 Hz, 1H, H-3-Hep), 3.71 (dd, J = 12.3, 6.5 Hz, 1H, H-7b-Hep), 3.54 (t, J = 9.9 Hz, 1H, H-4-ManNAc), 3.50 (dd, J = 9.9, 3.1 Hz, 1H, H-5-Hep), 3.43 (ddd, J = 9.9, 4.6, 2.2 Hz, 1H, H-5-ManNAc), 3.39 (dd, J = 11.3, 4.1 Hz, 1H, H-2-AAT), 2.49 (dd, J = 14.4, 8.3 Hz, 1H, H-2a-3Hb), 2.40 (dd, J = 14.3, 5.0 Hz, 1H, H-2b-3Hb), 2.09 (s, 3H, CH_3-Ac), 2.05 (s, 3H, CH_3-Ac), 1.22 (d, J = 6.1 Hz, 3H, CH_3-3Hb), 1.20 (d, J = 6.2 Hz, 3H, CH_3-Pr), 1.16 (d, J = 6.1 Hz, 3H, CH_3-Pr), 1.10 (d, J = 6.4 Hz, 3H, CH_3-AAT); ^{13}C NMR (126 MHz, D_2O) δ 179.29 (*C*=O-COOH), 175.67 (C=O-NHAc), 174.78 (C=O-NHAc), 97.53 (C-1-AAT, $J_{C1\text{-}H1}$ = 174 Hz), 97.16 (C-1-ManN, $J_{C1\text{-}H1}$ = 160Hz), 96.55 (C-1-Hep, $J_{C1\text{-}H1}$ = 159 Hz), 78.69, 77.66, 77.58, 76.37, 75.36, 74.08, 73.36, 72.91, 67.82, 65.64, 65.56, 65.09, 60.51, 60.20, 53.19, 51.46, 50.31, 44.90, 22.11 (*C*H$_3$-NHAc), 22.03 (*C*H$_3$-Pr), 21.84 (*C*H$_3$-NHAc), 21.05 (*C*H$_3$-Pr), 19.66 (*C*H$_3$-3Hb), 15.75 (*C*H$_3$-AAT). HRMS (ESI): m/z calcd for $C_{30}H_{54}N_3O_{17}^+$ [M+H]$^+$ 728.3448, found 728.3449.

7.2 Synthesis of compound 5

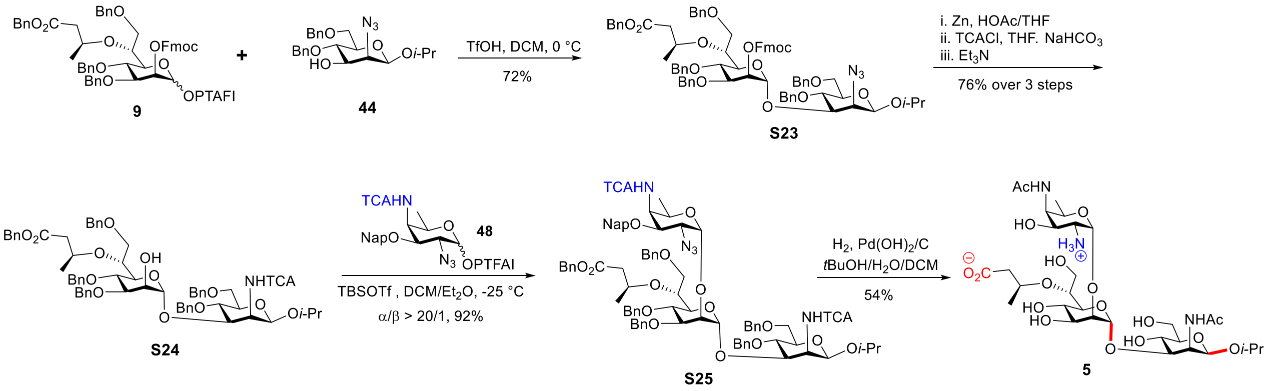

Scheme S18. Synthesis of trisaccharide **5**

Isopropyl 2-*O*-fluorenylmethoxycarbonyl-3,4,7-tri-*O*-benzyl-6-*O*-[benzyl 3(*S*)-butanoate]-D-*glycero*-*α*-D-*manno*-heptopyranosyl-(1→3)-2-azide-2-deoxy-4,6-di-*O*-benzyl-*β*-D-mannopyranoside (S23)

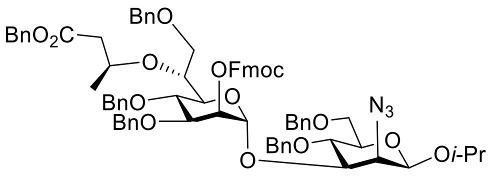

S23

The Hep-PTFAI donor **9** (100 mg, 100 μmol) and acceptor **44** (43 mg, 100 μmol) were co-evaporated three times with PhMe. Then, the mixture was dissolved in anhydrous CH_2Cl_2 (5 mL) and added freshly activated 4 Å molecular sieves. The mixture was stirred at room temperature for 15 min under an atmosphere of argon and then cooled to -25 °C. After being stirred at -25 °C for 15 min, TfOH (1.8 μL, 20 μmol) was added. Then, the reaction was stirred at -25 °C for 15 min. TLC analysis showed conversion of acceptor **44** to a mojor product **S23** (petroleum ether/ethyl acetate = 5/1, R_f = 0.40). The reaction was quenched by the addition of pyridine and filtered. The filtrate was concentrated *in vacuo* and purified by silica gel column chromatography (petroleum ether/ethyl acetate/CH_2Cl_2 = 10/1/1) to give compound **S23** (92 mg, 72%) as a colorless oil. ^{1}H NMR (600 MHz, $CDCl_3$) δ 7.82 – 7.71 (m, 2H), 7.63 – 7.53 (m, 2H), 7.44 – 7.15 (m, 34H), 5.28 (dd, J = 3.1, 1.8 Hz, 1H, H-2-Hep), 5.23 (d, J = 1.9 Hz, 1H, H-1-Hep), 5.14 – 5.06 (m, 2H), 4.90 (d, J = 11.1 Hz, 1H), 4.73 (dd, J = 11.0, 3.6 Hz, 2H), 4.69 – 4.63 (m, 3H), 4.61 (d, J = 11.2 Hz, 1H), 4.58 (d, J = 12.1 Hz, 1H), 4.54 – 4.45 (m, 3H), 4.41 (dd, J = 10.4, 7.2 Hz, 1H), 4.30 – 4.15 (m, 4H), 4.09 (dd, J = 8.8, 3.2 Hz, 1H), 4.07 – 3.99 (m, 4H), 3.83 (dd, J = 9.4, 3.5 Hz, 1H), 3.78 (d, J = 9.4 Hz, 1H), 3.76 – 3.68 (m, 3H), 3.59 (dd, J = 10.0, 6.4 Hz, 1H), 3.37 (dd, J = 9.6, 4.9 Hz, 1H), 2.86 (dd, J = 14.9, 5.2 Hz, 1H, H-2a-3Hb), 2.49 (dd, J = 14.9, 7.9 Hz, 1H, H-2b-3Hb), 1.28 (d, J = 6.1 Hz, 3H, C*H3*-Pr), 1.26 (d, J = 6.1 Hz, 3H, C*H3*-3Hb), 1.16 (d, J = 6.1 Hz, 3H, C*H3*-Pr). ^{13}C NMR (151 MHz, $CDCl_3$) δ 171.29 (*C*=O-COOBn), 154.72 (*C*=O-Fmoc), 143.66, 143.33, 141.40, 141.33, 138.59, 138.42, 137.98, 137.75, 136.14, 128.57, 128.52, 128.46, 128.43, 128.38, 128.30, 128.25, 128.17, 128.14, 128.03, 128.00, 127.98, 127.96, 127.93, 127.75, 127.69, 127.66, 127.61, 127.28, 127.26, 125.46, 125.26, 120.14, 120.11, 99.90 (C-1-Hep, $J_{C1\text{-}H1}$ = 172 Hz), 97.97 (C-1-ManN, $J_{C1\text{-}H1}$ = 157 Hz), 80.38, 78.48, 76.59, 75.74, 75.42, 74.89, 74.83, 74.41, 74.30, 73.65, 73.49, 73.01, 72.90, 72.36, 71.38, 70.50, 70.30, 69.12, 66.25, 63.90, 46.66, 41.94 (C-2-3Hb), 23.58 (*C*H3-Pr), 21.75 (*C*H3-Pr), 21.06 (*C*H3-3Hb). HRMS (ESI): m/z calcd for $C_{77}H_{81}N_3NaO_{15}^+$ [M+Na]$^+$ 1310.5560, found 1310.5564.

Isopropyl 3,4,7-tri-*O*-benzyl-6-*O*-[benzyl 3(*S*)-butanoate]-D-*glycero*-*α*-D-*manno*-heptopyranosyl-(1→3)-2-*N*-trichloroacetamid-2-deoxy-4,6-di-*O*-benzyl-*β*-D-mannopyranoside (S24)

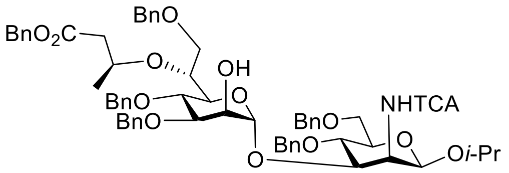

S24

To a solution of **S23** (77 mg, 60 μmol) in a mixed solvent of THF/HOAc (v/v, 3/1, 4 mL) was added Zn powder (50 mg). The reaction was stirred at room temperature overnight. TLC analysis showed complete conversion of starting material to amine intermediate (CH_2Cl_2/MeOH = 15/1, R_f = 0.30). The reaction was filtered through celite. The filtrate was concentrated, diluted with CH_2Cl_2, and washed with saturated $NaHCO_3$ solution. The organic layer was dried over Na_2SO_4 and concentrated under reduced pressure to afford amine intermediate for the next step.

To a solution of above amine intermediate in THF (4 mL) was added TCACl (10 μL, 89 μmol) at 0 °C. The reaction was stirred at room temperature for 0.5 h. TLC analysis showed complete conversion to NHTCA intermediate (petroleum ether/ethyl acetate = 4/1, R_f = 0.65). Then, the reaction was addding Et_3N (1 mL) and stirred at room temperature for 30 min. TLC analysis showed complete deprotection of Fmoc to give product **S24** (petroleum ether/ethyl acetate = 4/1, R_f = 0.20). The reaction mixture was diluted with CH_2Cl_2 and washed with saturated aqueous $NaHCO_3$ and brine. The organic phase was dried (Na_2SO_4), and filtered. The filtrate was concentrated *in vacuo* and purified by silica gel column chromatography (petroleum ether/ethyl acetate/CH_2Cl_2 = 6/1/1 to 4/1/1) to give compound **S24** (54 mg, 76% over three steps) as a colorless oil. ^{1}H NMR (600 MHz, $CDCl_3$) δ 7.42 – 7.22 (m, 30H), 6.96 (d, J = 9.3 Hz, 1H, N*H*-NHTCA), 5.23 – 5.12 (m, 3H), 4.86 (d, J = 11.6 Hz, 1H), 4.75 (d, J = 1.6 Hz, 1H), 4.69 – 4.60 (m, 5H), 4.60 – 4.54 (m, 2H), 4.52 – 4.49 (m, 1H), 4.47 (d, J = 6.2 Hz, 2H), 4.32 (q, J = 6.4 Hz, 1H), 4.25 (d, J = 9.7 Hz, 1H), 4.05 (dd, J = 8.2, 4.1 Hz, 1H), 4.02 – 3.96 (m, 2H), 3.93 (dd, J = 9.3, 3.2 Hz, 1H), 3.89 (t, J = 9.4 Hz, 1H), 3.85 (s, 1H), 3.81 – 3.74 (m, 3H), 3.71 (dd, J = 10.4, 3.9 Hz, 1H), 3.56 (dd, J = 10.4, 7.6 Hz, 1H), 3.48 – 3.46 (m, 1H), 2.79 (dd, J = 15.0, 6.7 Hz, 1H, H-2a-3Hb), 2.51 (dd, J = 14.9, 6.2 Hz, 1H, H-2b-3Hb), 1.27 (d, J = 6.2 Hz, 3H), 1.21 (d, J = 6.3 Hz, 3H), 1.14 (d, J = 6.1 Hz, 3H); ^{13}C NMR (151 MHz, $CDCl_3$) δ 172.08 (*C*=O-COOBn), 162.00 (*C*=O-TCA), 139.07, 138.77, 138.40, 138.21, 137.55, 136.12, 128.66, 128.57, 128.51, 128.47, 128.38, 128.33, 128.32, 128.22, 128.13, 128.02, 127.82, 127.77, 127.62, 127.60, 127.52, 127.38, 127.17, 100.93, 96.69, 93.21, 79.94, 76.47, 75.08, 74.99, 73.85, 73.69, 73.54, 73.48, 73.29, 72.90, 72.11, 71.74, 70.69, 68.96, 68.66, 66.43, 55.12, 42.38, 23.33, 21.62, 20.60. HRMS (ESI): m/z calcd for $C_{64}H_{76}Cl_3N_2O_{14}^+$ $[M+NH_4]^+$ 1201.4357,

found 1201.4347.

Isopropyl 2-azide-4-trichloroacetam58ido-2,4,6-tri-deoxy-*α*-D-galactopyranosyl-(1→2)-[[benzyl 3(S)-hydroxy-butanoate-(3→6)]-3,4,7-tri-*O*-benzyl-D-*glycero*-*α*-D-*manno*-heptopyranosyl]-(1→3)-2-*N*-trichloroacetamid-4,6-di-*O*-benzyl-2-deoxy-*β*-D-mannopyranoside (S25)

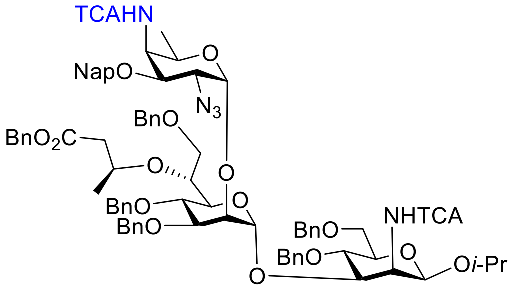

S25

The AAT-PTFAI donor **48** (55 mg, 84 μmol) and acceptor **S24** (50 mg, 42 μmol) were co-evaporated three times with PhMe. Then, the mixture was dissolved in a mixed solvent of anhydrous CH_2Cl_2/Et_2O(v/v, 2/1, 3 mL) and added freshly activated 4 Å molecular sieves. The mixture was stirred at room temperature for 15 min under an atmosphere of argon and then cooled to -25 ºC. After being stirred at -25 ºC for 15 min, TBSOTf (1.5 μL, 2.0 μmol) was added. Then, the reaction was stirred at -25 ºC for 15 min. TLC analysis showed complete conversion of acceptor **S24** to product **S25** (petroleum ether/ethyl acetate = 4/1, R_f = 0.45). The reaction was quenched by the addition of Et_3N, and the mixture was filtered. The filtrate was concentrated *in vacuo* and purified by silica gel column chromatography (petroleum ether/ethyl acetate/CH_2Cl_2 = 8/1/1 to 6/1/1) to give compound **S25** (64 mg, 92%) as a colorless oil. ^{1}H NMR (800 MHz, $CDCl_3$) δ 7.92 – 7.79 (m, 4H, ArH), 7.55 – 7.43 (m, 3H, ArH), 7.41 – 7.21 (m, 30H, ArH), 6.95 (d, J = 9.1 Hz, 1H, N*H*-NHTCA), 6.67 (d, J = 9.8 Hz, 1H, N*H*-NHTCA), 5.18 (d, J = 2.0 Hz, 1H, H-1-Hep), 5.17 – 5.10 (m, 2H), 4.97 (d, J = 10.6 Hz, 1H), 4.93 (d, J = 11.6 Hz, 1H), 4.85 (d, J = 4.1 Hz, 1H, H-1-AAT), 4.77 (d, J = 1.6 Hz, 1H, H-1-ManN), 4.70 – 4.67 (m, 2H), 4.64 (d, J = 11.9 Hz, 1H), 4.62 – 4.59 (m, 2H), 4.57 – 4.49 (m, 4H), 4.49 – 4.43 (m, 2H), 4.43 – 4.39 (m, 1H), 4.31 (d, J = 8.7 Hz, 1H), 4.25 (dt, J = 8.0, 5.6 Hz, 1H), 4.09 – 4.01 (m, 6H), 3.99 – 3.96 (m, 1H), 3.78 (t, J = 9.4 Hz, 1H), 3.76 (t, J = 2.2 Hz, 1H), 3.73 – 3.66 (m, 3H), 3.58 (dd, J = 10.4, 7.4 Hz, 1H), 3.47 – 3.43 (m, J = 9.4, 3.0 Hz, 1H), 3.15 (dd, J = 11.0, 3.9 Hz, 1H), 2.88 (dd, J = 14.8, 5.4 Hz, 1H, H-2a-3Hb), 2.45 (dd, J = 14.8, 8.0 Hz, 1H, H-2b-3Hb), 1.25 (d, J = 6.2 Hz, 3H, C*H₃*-3Hb), 1.20 (d, J = 6.2 Hz, 3H, C*H₃*-Pr), 1.13 (d, J = 6.2 Hz, 3H, C*H₃*-Pr), 0.90 (d, J = 6.5 Hz, 3H, C*H₃*-AAT); ^{13}C NMR (201 MHz, $CDCl_3$) δ 171.37 (*C*=O-COOBn), 162.72 (*C*=O-TCA), 161.99 (*C*=O-TCA), 139.11, 138.83, 138.69,

138.18, 137.92, 136.13, 134.67, 133.39, 133.22, 128.61, 128.55, 128.52, 128.47, 128.45, 128.44, 128.38, 128.35, 128.28, 128.25, 128.21, 128.18, 128.13, 127.86, 127.80, 127.77, 127.75, 127.74, 127.59, 127.55, 127.52, 127.48, 127.46, 127.39, 127.34, 127.20, 126.36, 126.06, 126.00, 99.70 (C-1-Hep, $J_{C1\text{-}H1}$ = 172 Hz), 99.46 (C-1-AAT, $J_{C1\text{-}H1}$ = 171 Hz), 96.68 (C-1-ManN, $J_{C1\text{-}H1}$ = 158 Hz), 93.21 (CCl_3), 92.76 (CCl_3), 79.58, 77.47, 77.24, 76.35, 75.39, 75.02, 74.40, 74.17, 74.13, 73.81, 73.38, 73.32, 72.82, 72.79, 71.66, 71.32, 70.71, 68.57, 66.26, 64.84, 59.53, 55.35, 52.16, 42.52 (C-2-3Hb), 23.31 (CH_3-Pr), 21.60 (CH_3-Pr), 20.65 (CH_3-3Hb), 16.43 (CH_3-AAT). HRMS (ESI): m/z calcd for $C_{83}H_{89}Cl_6N_5NaO_{17}^+$ $[M+Na]^+$ 1660.4277, found 1660.4278.

Isopropyl 2-amino-4-*N*-acetyl-2,4,6-tri-deoxy-*α*-D-galactopyranosyl-(1→2)-[6-*O*-[3(*S*)-butanoic acid]-D-*glycero*-*α*-D-*manno*-heptopyranosyl]-(1→3)-2-*N*-acetyl-2-deoxy-*β*-D-mannopyranoside (5)

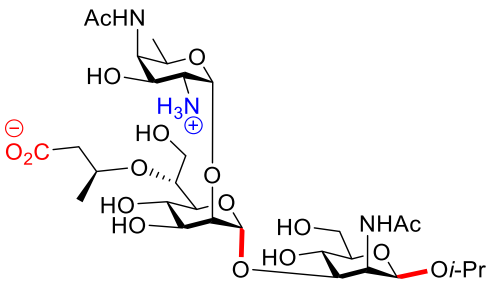

5

To a solution of the **S25** (10 mg, 6.1 μmol) in *t*-BuOH/H_2O/CH_2Cl_2 (4/1/1, v/v/v, 6 mL) was added $Pd(OH)_2$/C (20%, 20 mg). The mixture was stirred at room temperature for 18 h under H_2 atmosphere. ESI-MS analysis showed the complete conversion of starting material to a major product **5**. The reaction mixture was filtered through celite, and filtration was concentrated under reduced pressure to give a crude product, which was sequentially purified by reverse-phase silica column (C-18, eluent: 20% CH_3CN in H_2O to 30%) and size-exclusion chromatography (BioGel P-4, 45–90 μm, eluent: 0.1 M NH_4HCO_3). The product-containing fractions were combined and lyophilized to afford the product **5** as a white amorphous solid (2.7 mg, 54%). ^{1}H NMR (800 MHz, D_2O) δ 5.37 (d, J = 1.7 Hz, 1H, H-1-Hep), 5.30 (d, J = 4.1 Hz, 1H, H-1-AAT), 4.91 (d, J = 1.6 Hz, 1H, H-1-ManNAc), 4.53 (d, J = 3.7 Hz, 1H, H-2-ManNAc), 4.32 (d, J = 3.8 Hz, 1H, H-4-AAT), 4.28 (dd, J = 11.3, 4.4 Hz, 1H, H-3-AAT), 4.26 – 4.20 (m, 2H, H-5-AAT, H-3-3Hb), 4.08 – 4.04 (m, 1H, C*H*-Pr), 4.03 – 3.98 (m, 2H, H-2-Hep, H-3-Hep), 3.98 – 3.95 (m, 1H, H-6-Hep), 3.94 (dd, J = 10.2, 4.4 Hz, 1H, H-3-ManNAc), 3.91 – 3.88 (m, 1H, H-6a-ManNAc), 3.87 (t, J = 10.0 Hz, 1H, H-4-Hep), 3.85 – 3.82 (m, 1H, H-7a-Hep), 3.82 – 3.79 (m, 2H, H-5-Hep, H-6a-ManNAc), 3.73 (dd, J = 12.1, 7.4 Hz, 1H, H-7b-Hep), 3.66 (t, J = 9.9 Hz, 1H, H-4-ManNAc), 3.45 (ddd, J = 10.1, 4.9, 2.3 Hz, 1H, H-5-ManNAc),

2.67 (dd, J = 11.3, 4.0 Hz, 1H, H-2-AAT), 2.69 – 2.66 (m, 1H, H-2a-3Hb), 2.49 – 2.42 (m, 1H, H-2b-3Hb), 2.10 (s, 3H, CH_3-NHAc), 2.08 – 2.03 (m, 3H, CH_3-NHAc), 1.28 (d, J = 6.2 Hz, 3H, CH_3-3Hb), 1.21 (d, J = 6.3 Hz, 3H, CH_3-Pr), 1.17 (d, J = 6.2 Hz, 3H, CH_3-Pr), 1.11 (d, J = 6.5 Hz, 3H, CH_3-AAT); ^{13}C NMR (126 MHz, D_2O) δ 177.47 (*C*=O-COOH), 175.68 (*C*=O-NHAc), 174.75 (*C*=O-NHAc), 99.52 (C-1-Hep, $J_{C1\text{-}H1}$ = 174 Hz), 97.79 (AAT, $J_{C1\text{-}H1}$ = 172 Hz), 97.50 (C-1-ManN, $J_{C1\text{-}H1}$ = 160 Hz), 79.15, 77.79, 76.73, 76.08, 73.04, 72.95, 70.00, 67.23, 67.05, 66.18, 65.79, 61.26, 60.10, 53.04, 51.24, 43.57, 21.98 (*C*H_3-NHAc, *C*H_3-Pr), 21.84 (*C*H_3-NHAc), 21.15 (*C*H_3-Pr), 19.04 (*C*H_3-3Hb), 15.37 (*C*H_3-AAT). HRMS (ESI): m/z calcd for $C_{30}H_{52}N_3O_{17}^-$ [M-H]$^-$ 726.3302, found 726.3307.

8. Reference

1. Wang, Y.; Kalka-Moll, W. M.; Roehrl, M. H.; Kasper, D. L. Structural Basis of the Abscess-Modulating Polysaccharide A2 from *Bacteroides Fragilis*. *Proc. Natl. Acad. Sci. USA* **2000**, *97*, 13478–13483.
2. Li, J.; Gao, R.; Hao, T.; Li, T. Stereocontrolled Synthesis of a Heptose- and Kdo-Containing Common Inner-Core Trisaccharide of Lipopolysaccharides from Multiple Pathogenic Bacteria. *Synlett* **2024**, *36*, 1247–1251.
3. Hesek, D.; Lee, M.; Zhang, W.; Noll, B. C.; Mobashery, S. Total Synthesis of *N*-Acetylglucosamine-1,6-Anhydro-*N*-Acetylmuramylpentapeptide and Evaluation of Its Turnover by AmpD from *Escherichia Coli*. *J. Am. Chem. Soc.* **2009**, *131*, 5187–5193.
4. Xing, S.; Gleason, J. L. A Robust Synthesis of *N*-Glycolyl Muramyl Dipeptide via Azidonitration/Reduction. *Org. Biomol. Chem.* **2015**, *13*, 1515–1520.
5. Ding, D.; Gao, R.; Lei, Y.; Liu, J.; Zhou, C.; Wen, Y.; Zhou, S.; Guo, J.; Li, T. Synergistic Immune Augmentation Enabled by Covalently Conjugating TLR4 and NOD2 Agonists. *Eur. J. Med. Chem.* **2024**, *278*, 116792.
6. Levin, S.; Nani, R. R.; Reisman, S. E. Enantioselective Total Synthesis of (+)-Salvileucalin B. *J. Am. Chem. Soc.* **2011**, *133*, 774–776.
7. Wang, P.; Huo, C.; Lang, S.; Caution, K.; Nick, S. T.; Dubey, P.; Deora, R.; Huang, X. Chemical Synthesis and Immunological Evaluation of a Pentasaccharide Bearing Multiple Rare Sugars as a Potential Anti-*Pertussis* Vaccine. *Angew. Chem. Int. Ed.* **2020**, *59*, 6451–6458.
8. Ding, N.; Li, X.; Chinoy, Z. S.; Boons, G.-J. Synthesis of a Glycosylphosphatidylinositol Anchor Derived from *Leishmania Donovani* That Can Be Functionalized by Cu-Catalyzed Azide–Alkyne Cycloadditions. *Org. Lett.* **2017**, *19*, 3827–3830.
9. Jaeschke, S. O.; Lindhorst, T. K. Versatile Synthesis of Diaminoxylosides via Iodosulfonamidation of Xylal Derivatives. *Eur. J.Org. Chem.* **2021**, 6312–6318.
10. Rai, D.; Kulkarni, S. S. Total Synthesis of Trisaccharide Repeating Unit of *Staphylococcus Aureus* Type 8 (CP8) Capsular Polysaccharide. *Org. Lett.* **2023**, *25*, 1509–1513.
11. Tian, G.; Bao, J.; Chen, G.; Zou, X.; Qin, C.; Hu, J.; Yin, J. Total Synthesis of the Conjugation-Ready

Hexasaccharides of *Pseudomonas Aeruginosa* Serotype O17 O-Antigen via One-Pot Glycosylation. *Chin. J. Chem.* **2025***, 43,* 743–749.

12. Wang, Z.; Zhou, L.; El-Boubbou, K.; Ye, X.; Huang, X. Multi-Component One-Pot Synthesis of the Tumor-Associated Carbohydrate Antigen Globo-H Based on Preactivation of Thioglycosyl Donors. *J. Org. Chem.* **2007**, *72*, 6409–6420.
13. Walk, J. T.; Buchan, Z. A.; Montgomery, J. Sugar Silanes: Versatile Reagents for Stereocontrolled Glycosylation via Intramolecular Aglycone Delivery. *Chem. Sci.* **2015**, *6*, 3448–3453.
14. Stork, G.; La Clair, J. J. Stereoselective Synthesis of *β*-Mannopyranosides via the Temporary Silicon Connection Method. *J. Am. Chem. Soc.* **1996**, *118*, 247–248.
15. Cai, J.; Hu, J.; Qin, C.; Li, L.; Shen, D.; Tian, G.; Zou, X.; Seeberger, P. H.; Yin, J. Chemical Synthesis Elucidates the Key Antigenic Epitope of the Autism-Related Bacterium *Clostridium Bolteae* Capsular Octadecasaccharide. *Angew. Chem. Int. Ed.* **2020**, *59*, 20529–20537.
16. Zhu, Y.; Yu, B. Highly Stereoselective *β*-Mannopyranosylation via the 1-α-Glycosyloxy-Isochromenylium-4-Gold(I) Intermediates. *Chem. Eur. J.* **2015**, *21*, 8771–8780.
17. Chen, Y.; Wu, Z.; Li, X.; Hu, Y.; Yang, Y. Promoter-Assisted Stereoselective Synthesis of the 6-Deoxy-*β*-D-*manno*-Heptopyranose Oligosaccharides. *Org. Lett.* **2021**, *23*, 3216–3220.
18. Wang, X.; Chen, Y.; Wang, J.; Yang, Y. Total Synthesis of the Trisaccharide Antigen of the *Campylobacter Jejuni* RM1221 Capsular Polysaccharide via *de Novo* Synthesis of the 6-Deoxy-D-*manno*-Heptose Building Blocks. *J. Org. Chem.* **2019**, *84*, 2393–2403.
19. Mishra, K. B.; Singh, A. K.; Kandasamy, J. Tris(Pentafluorophenyl)Borane-Promoted Stereoselective Glycosylation with Glycosyl Trichloroacetimidates under Mild Conditions. *J. Org. Chem.* **2018**, *83*, 4204–4212.

9. NMR and HRMS spectrum

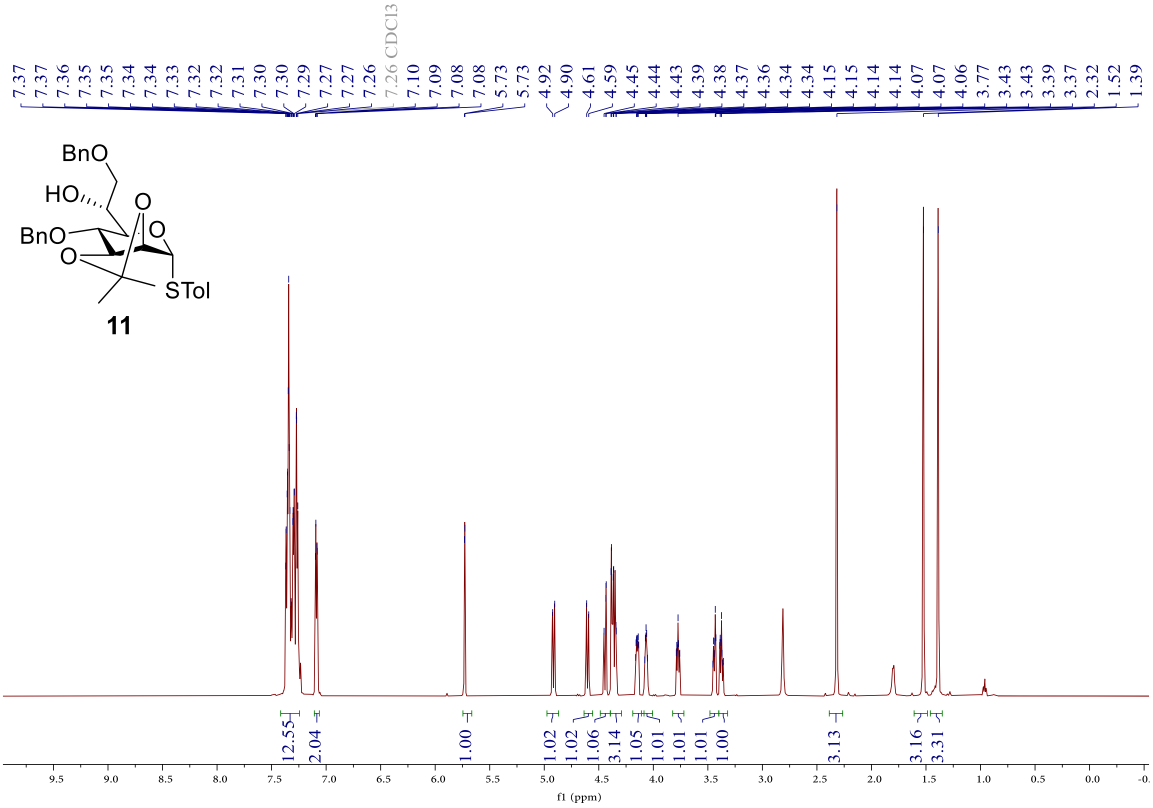

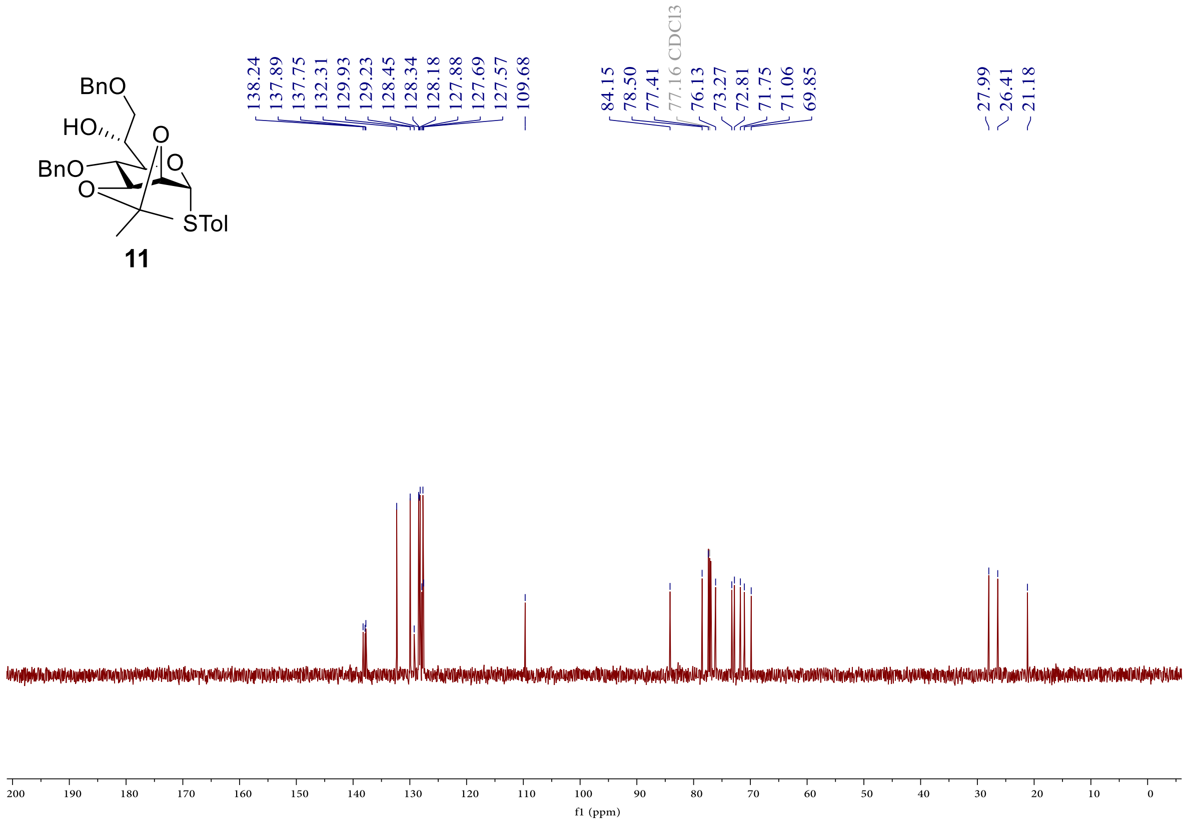

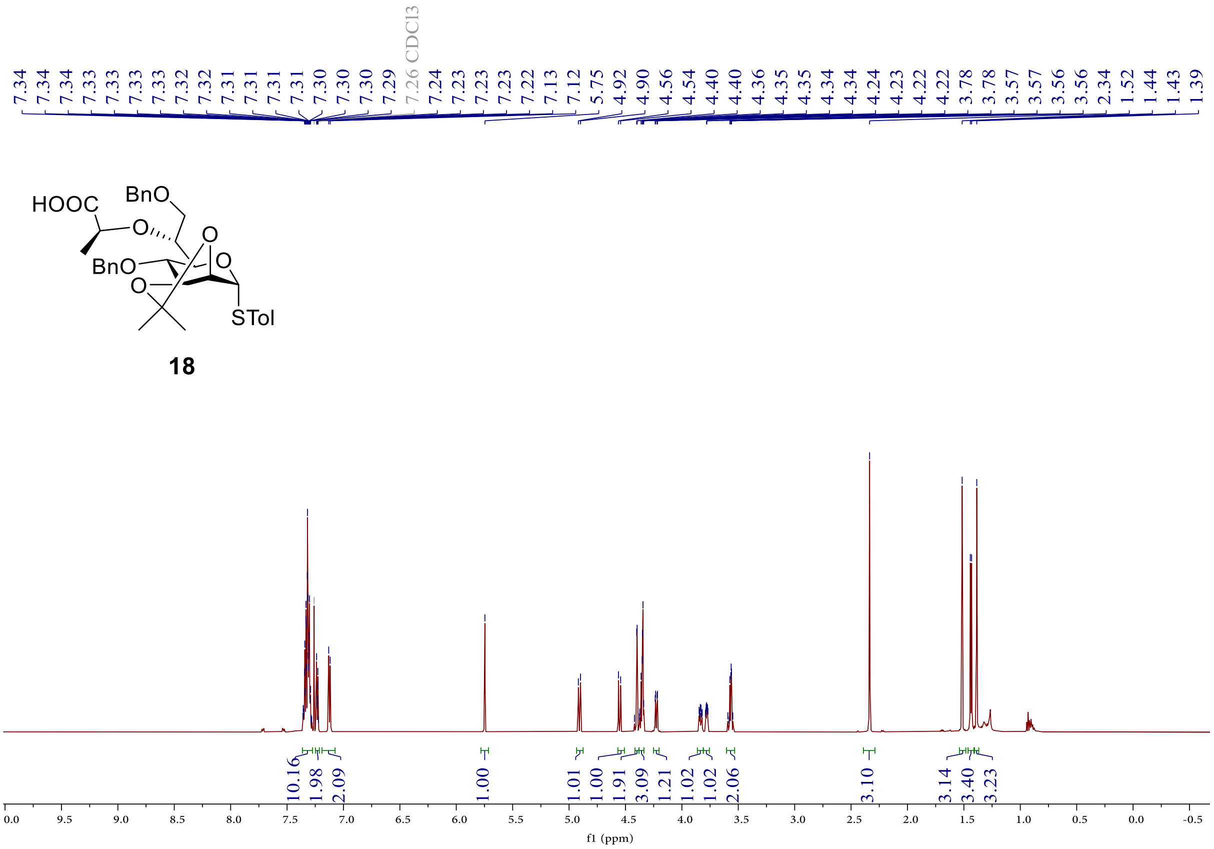

18

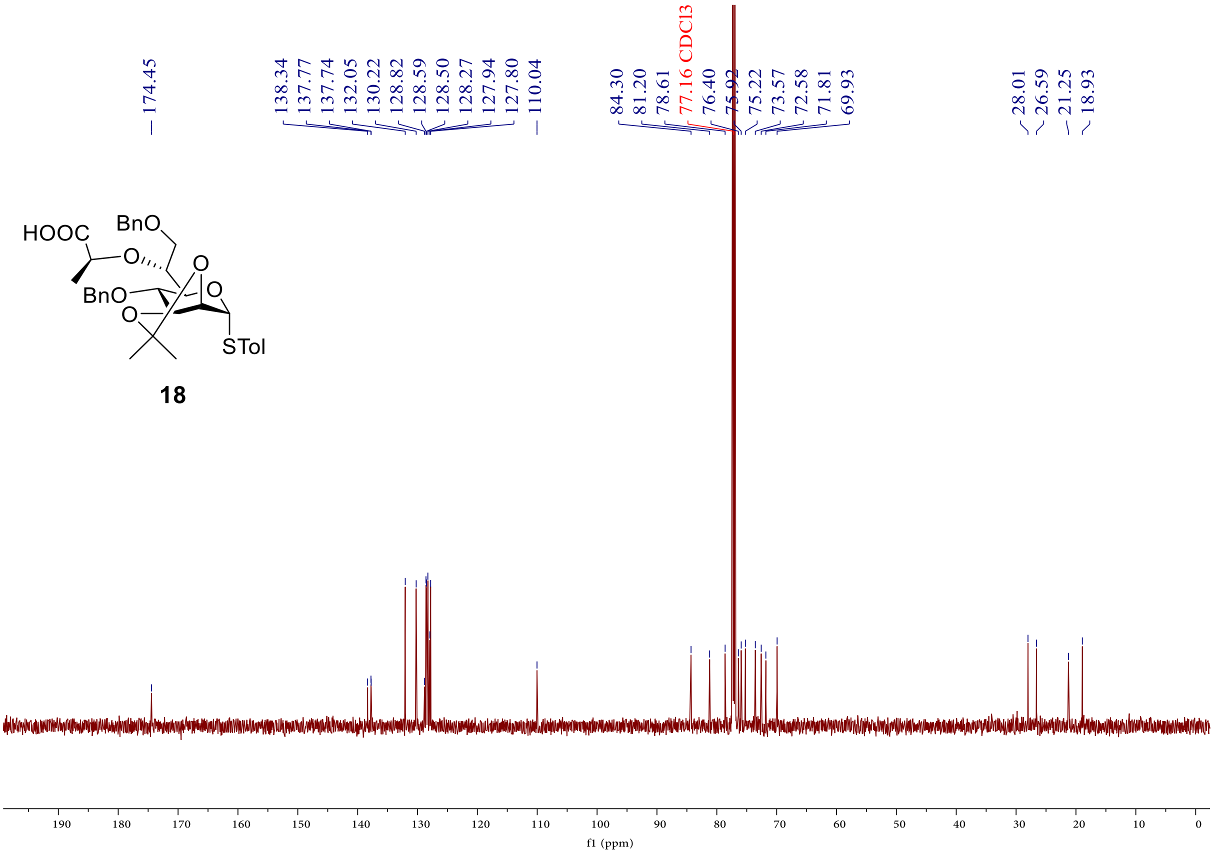

18

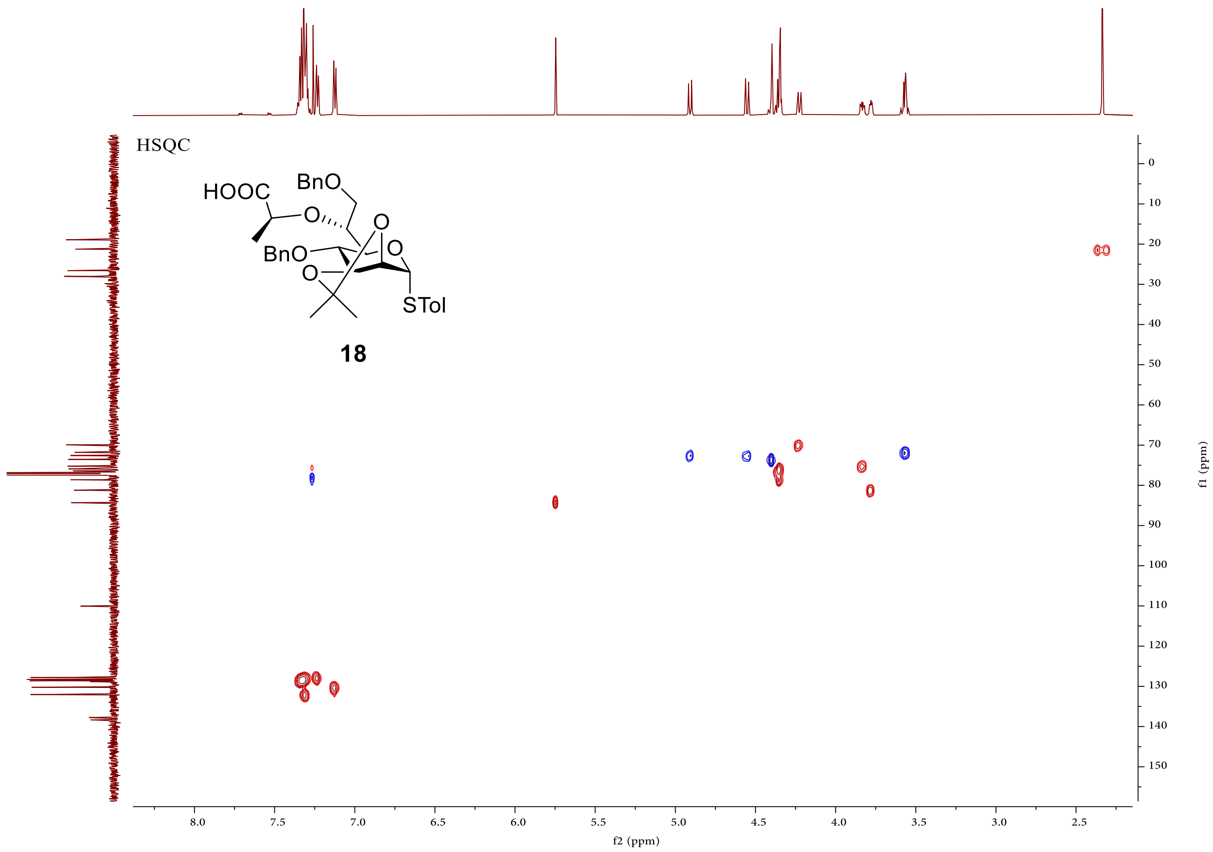
HSQC
HOOC
BnO
BnO
O
STol
18
f2 (ppm)
f1 (ppm)

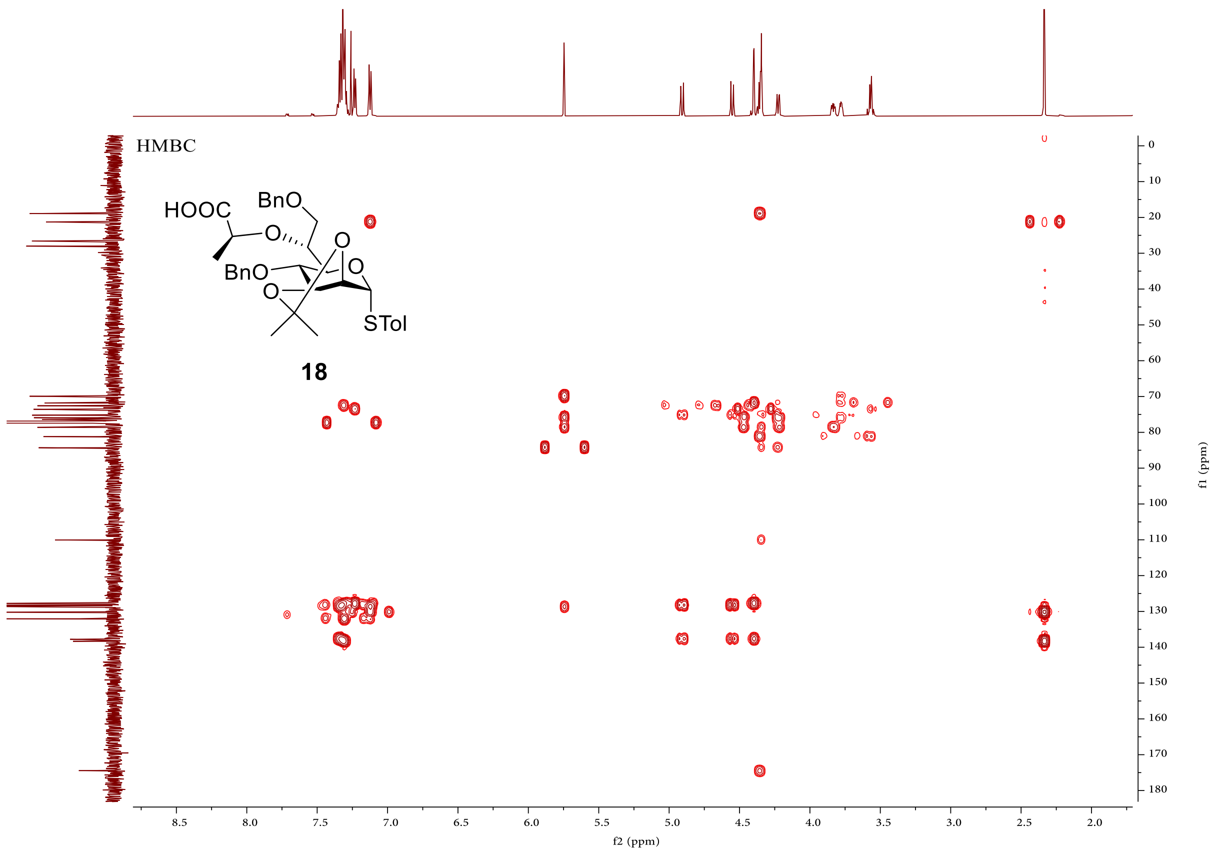
HMBC
HOOC
BnO
BnO
O
STol
18
f2 (ppm)
f1 (ppm)

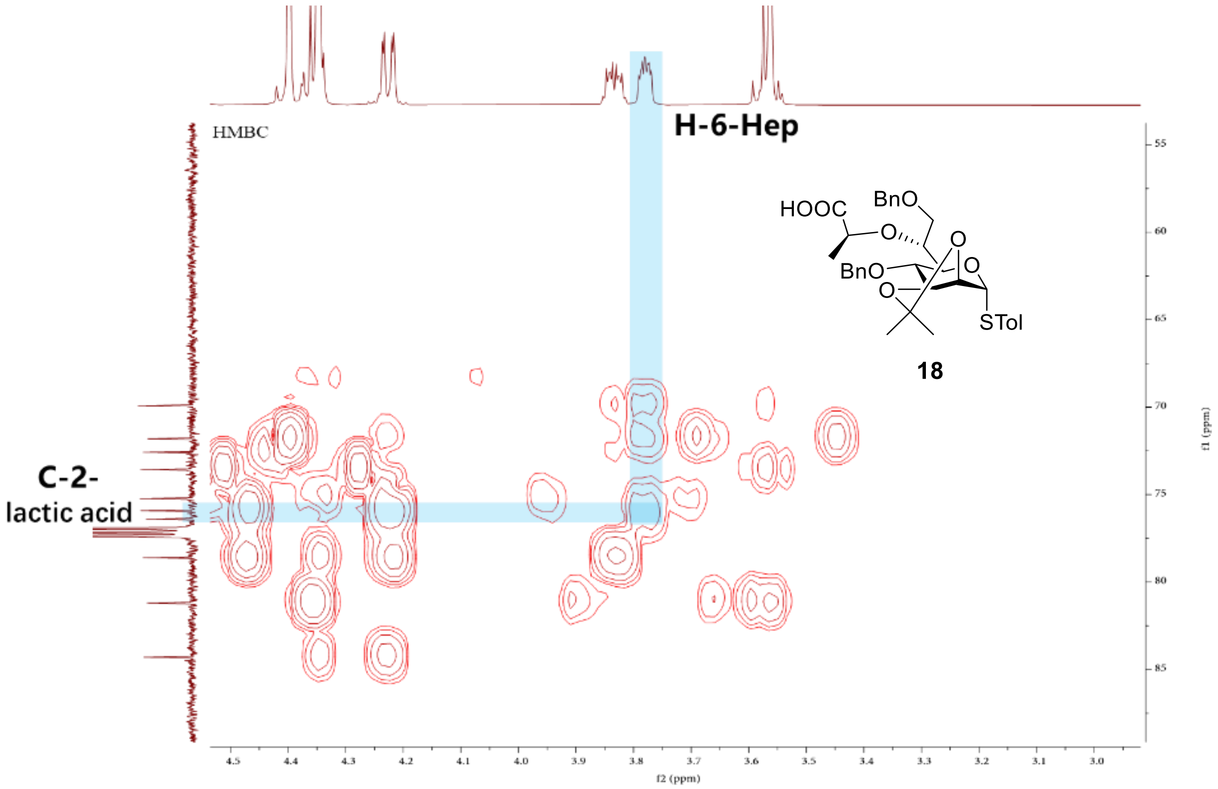
HMBC
H-6-Hep
C-2-
lactic acid
HOOC
BnO
BnO
O
STol
18
f2 (ppm)
f1 (ppm)

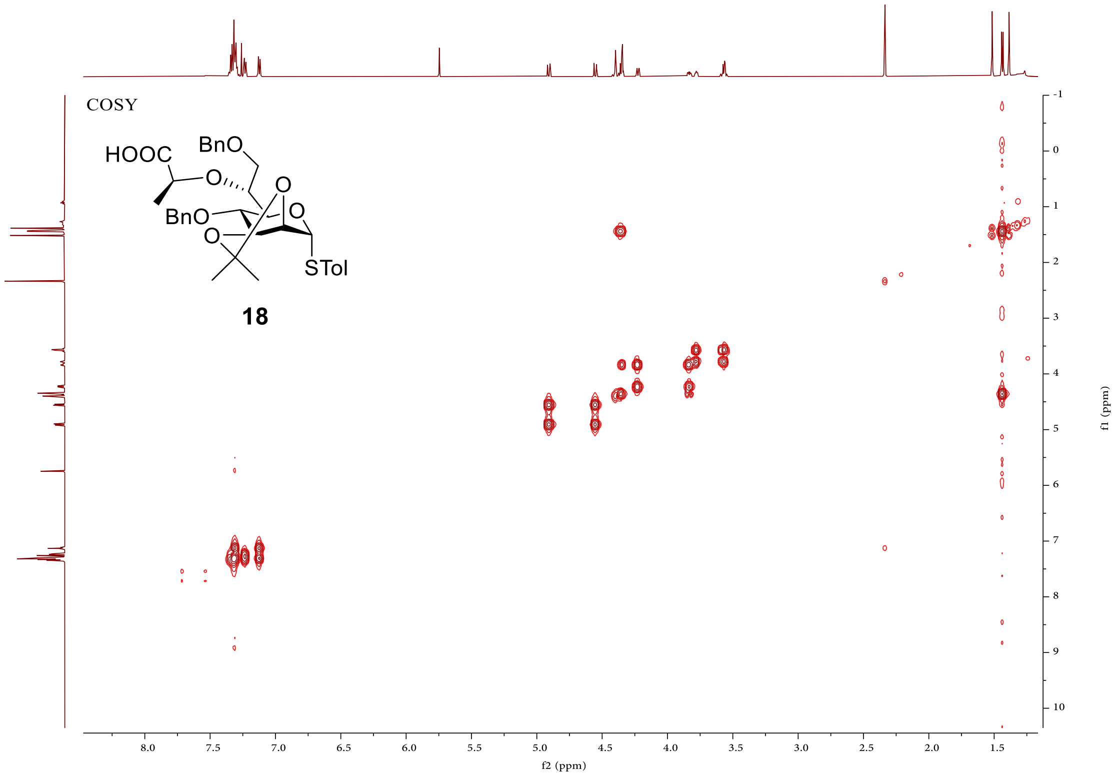
COSY
HOOC
BnO
BnO
O
STol
18
f2 (ppm)
f1 (ppm)

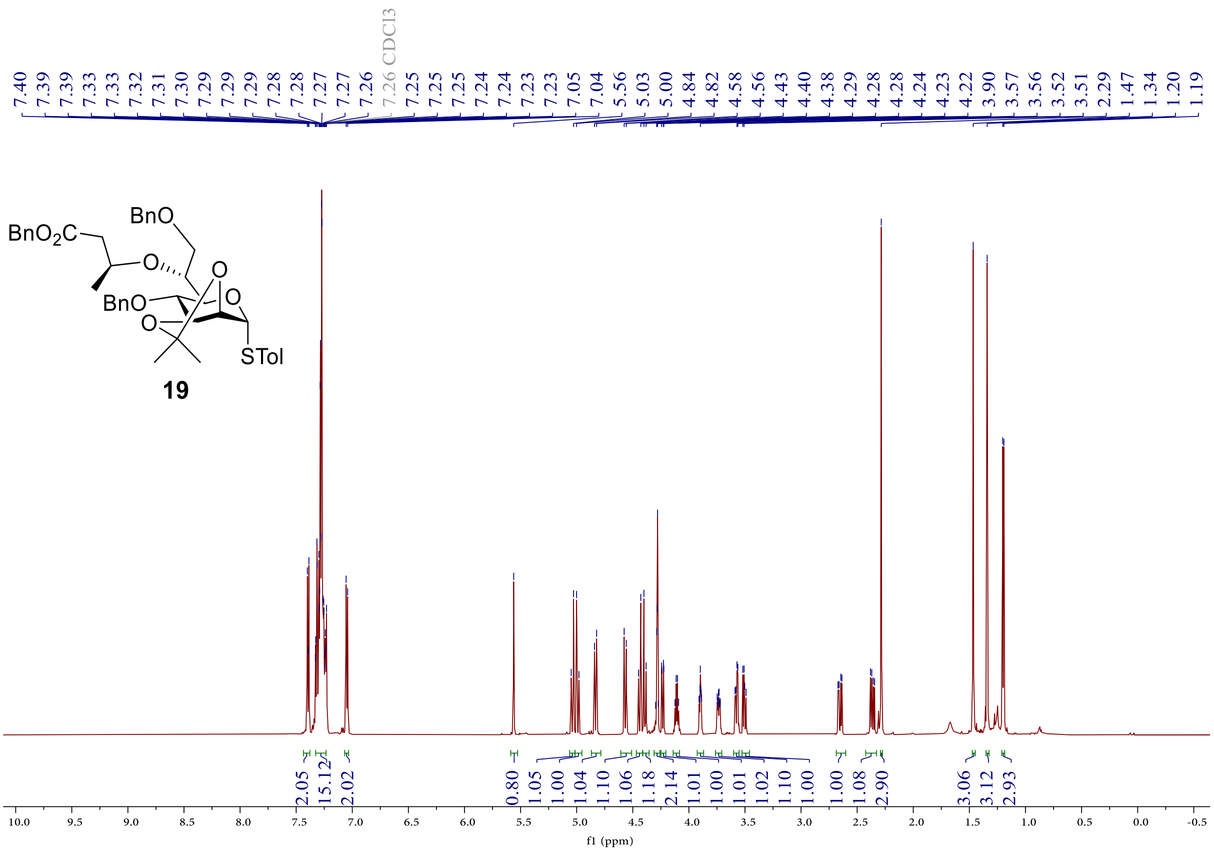
7.40 7.39 7.39 7.33 7.33 7.32 7.31 7.30 7.29 7.29 7.29 7.28 7.27 7.27 7.26 7.26 CDCl3 7.25 7.25 7.25 7.24 7.24 7.23 7.23 7.05 7.04 5.56 5.03 5.00 4.84 4.82 4.58 4.56 4.43 4.40 4.38 4.29 4.28 4.28 4.24 4.23 4.22 3.90 3.57 3.56 3.52 3.51 2.29 1.47 1.34 1.20 1.19
BnO
BnO2C
O
O
BnO
O
O
STol
19
2.05 15.12 2.02 0.80 1.05 1.00 1.04 1.10 1.06 1.18 2.14 1.01 1.00 1.01 1.02 1.10 1.00 1.00 1.08 2.90 3.06 3.12 2.93
f1 (ppm)

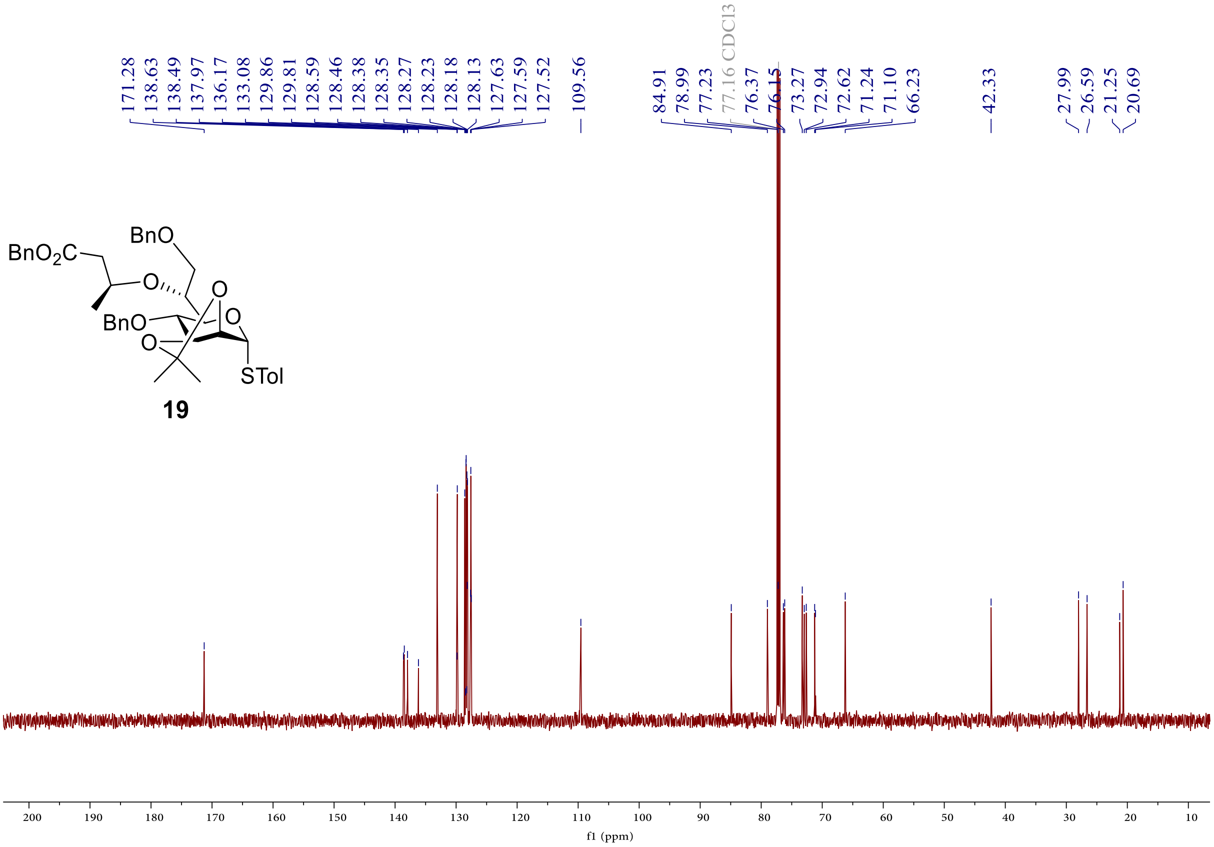
171.28 138.63 138.49 137.97 136.17 133.08 129.86 129.81 128.59 128.46 128.38 128.35 128.27 128.23 128.18 128.13 127.63 127.59 127.52 109.56 84.91 78.99 77.23 77.16 CDCl3 76.37 76.15 73.27 72.94 72.62 71.24 71.10 66.23 42.33 27.99 26.59 21.25 20.69
BnO
BnO2C
O
O
BnO
O
O
STol
19
f1 (ppm)

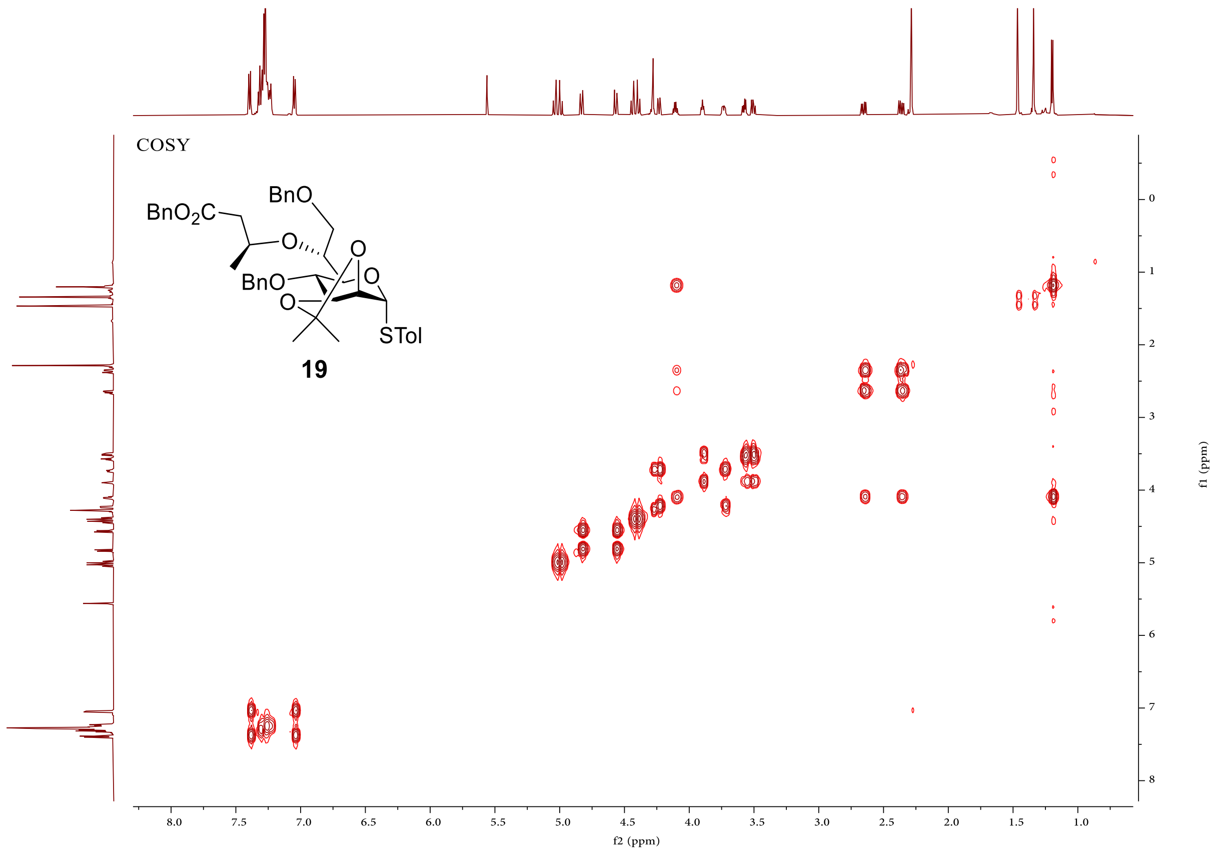

COSY
BnO
BnO2C
O
BnO
O
O
O
STol
19
f2 (ppm)
f1 (ppm)

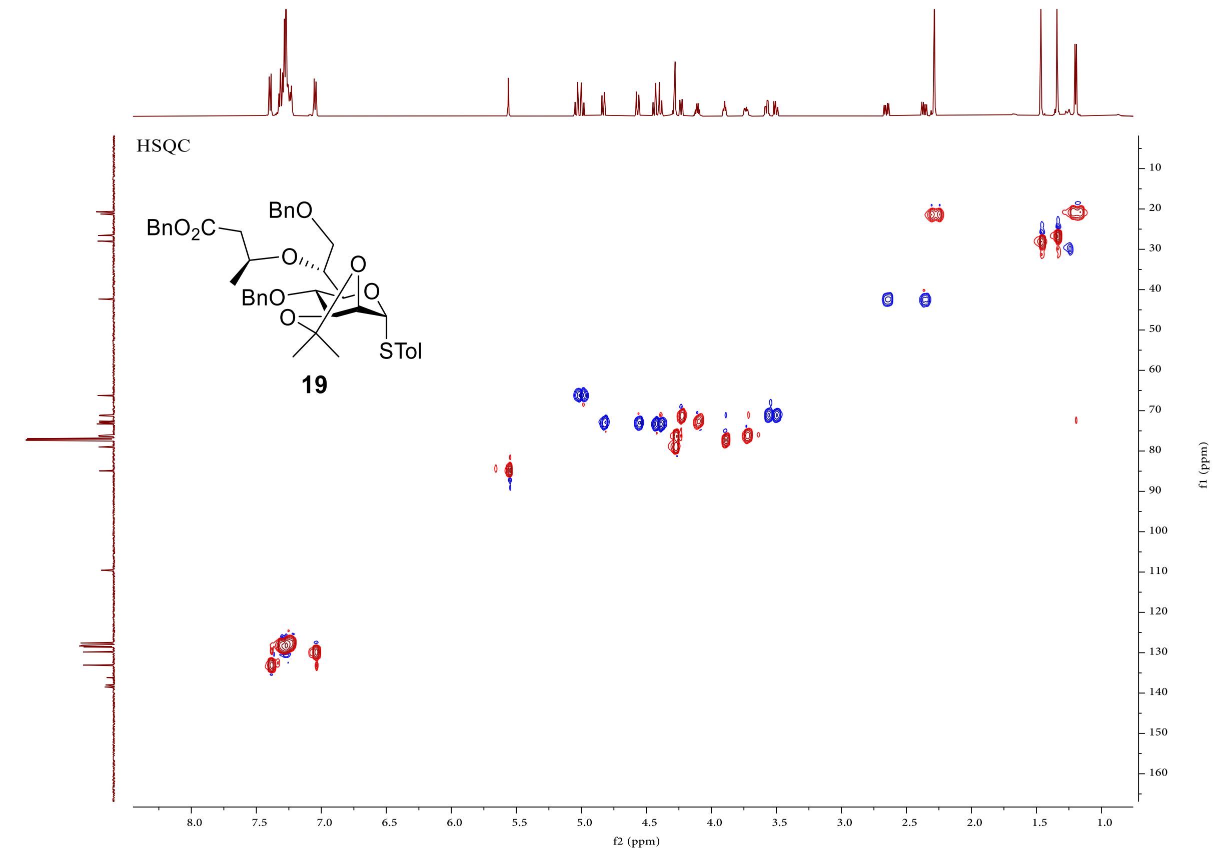

HSQC
BnO
BnO2C
O
BnO
O
O
O
STol
19
f2 (ppm)
f1 (ppm)

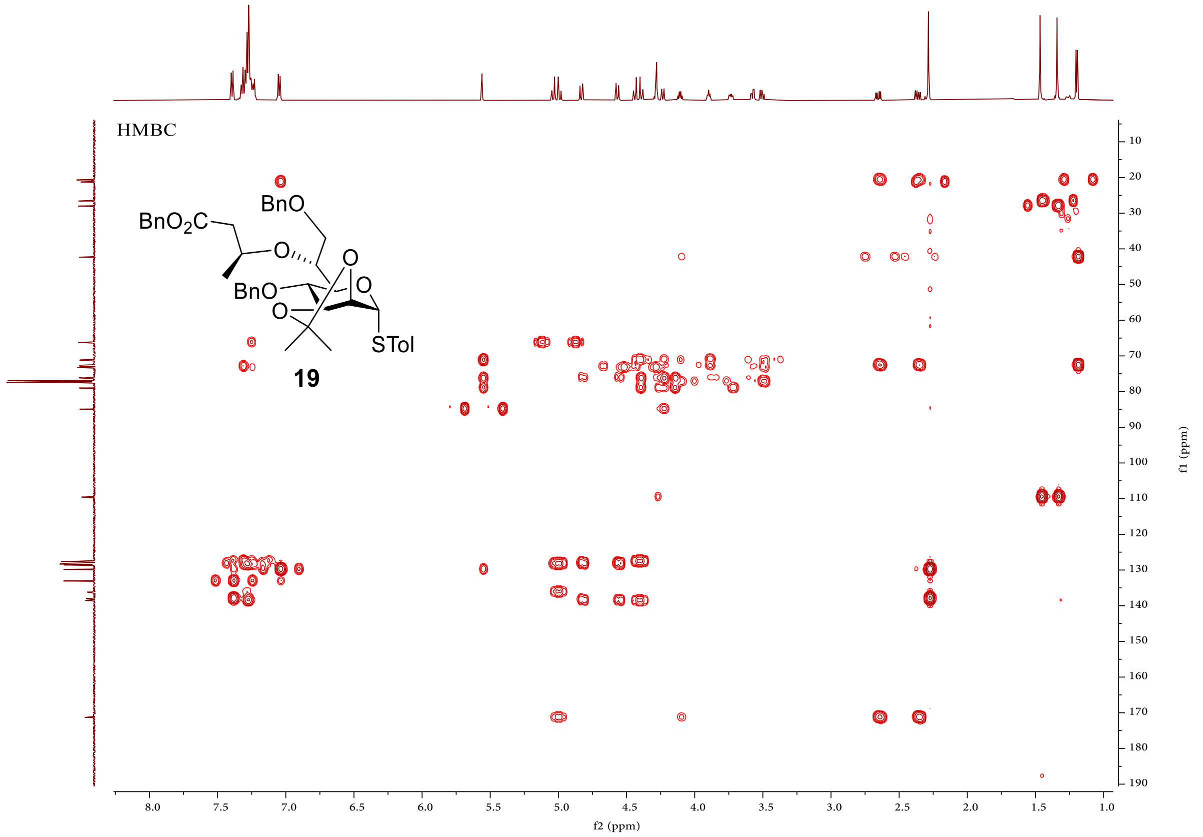
HMBC
BnO
BnO2C
O
BnO
O
O
O
STol
19
f2 (ppm)
f1 (ppm)

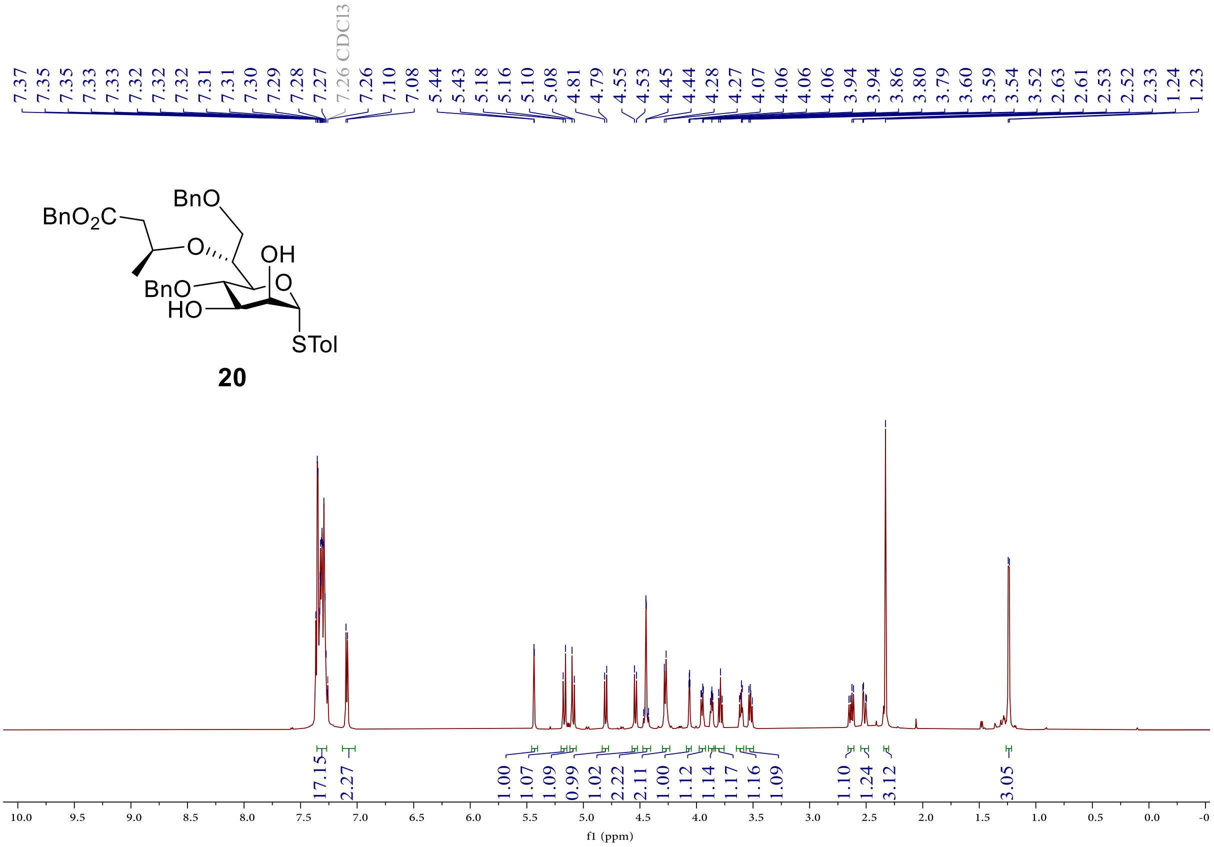
7.37 7.35 7.35 7.33 7.33 7.32 7.32 7.32 7.31 7.31 7.30 7.29 7.28 7.27 7.26 CDCl3 7.26 7.10 7.08 5.44 5.43 5.18 5.16 5.10 5.08 4.81 4.79 4.55 4.53 4.45 4.44 4.28 4.27 4.07 4.06 4.06 4.06 3.94 3.94 3.86 3.80 3.79 3.60 3.59 3.54 3.52 2.63 2.61 2.53 2.52 2.33 1.24 1.23
BnO
BnO2C
O
OH
O
BnO
HO
STol
20
17.15 2.27 1.00 1.07 1.09 0.99 1.02 2.22 2.11 1.00 1.12 1.14 1.17 1.16 1.09 1.10 1.24 3.12 3.05
f1 (ppm)

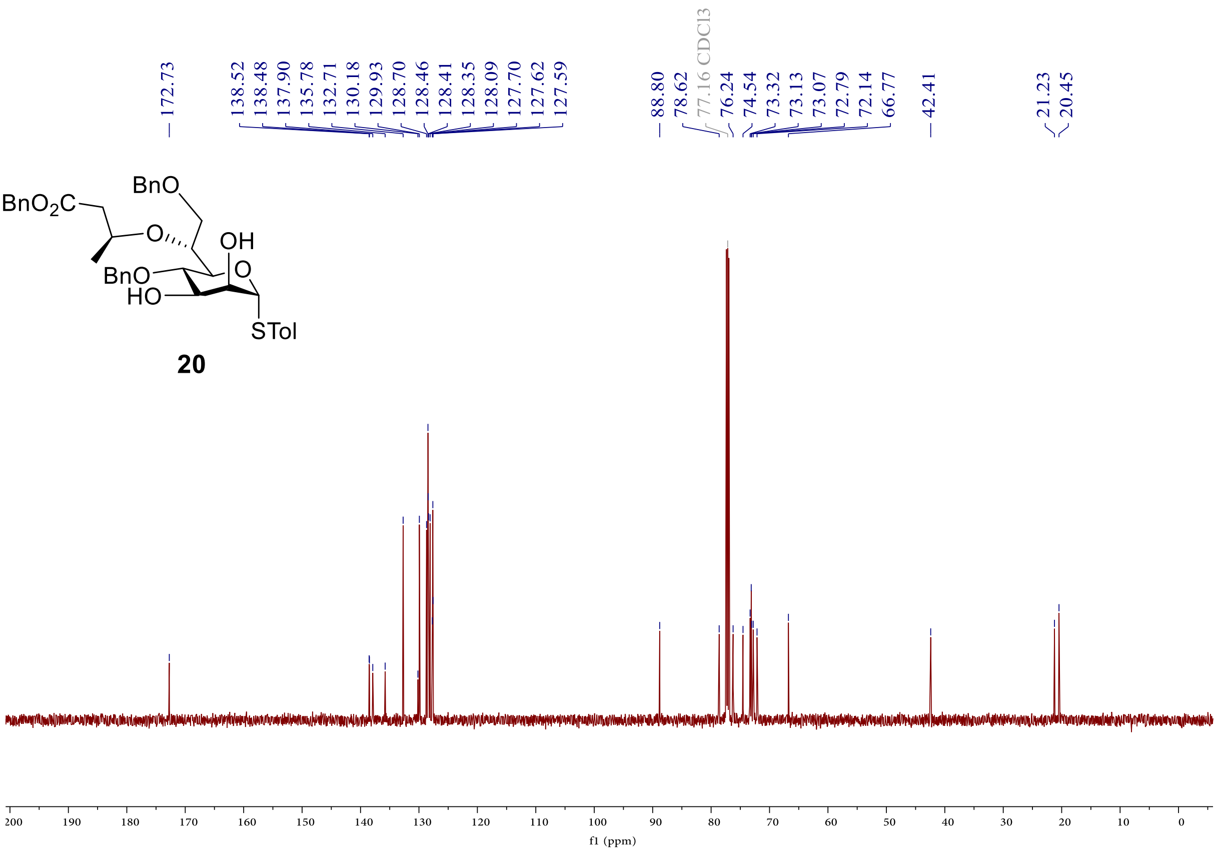
BnO
BnO_2C
O
OH
O
BnO
HO
STol
20
172.73
138.52
138.48
137.90
135.78
132.71
130.18
129.93
128.70
128.46
128.41
128.35
128.09
127.70
127.62
127.59
88.80
78.62
77.16 CDCl3
76.24
74.54
73.32
73.13
73.07
72.79
72.14
66.77
42.41
21.23
20.45
f1 (ppm)

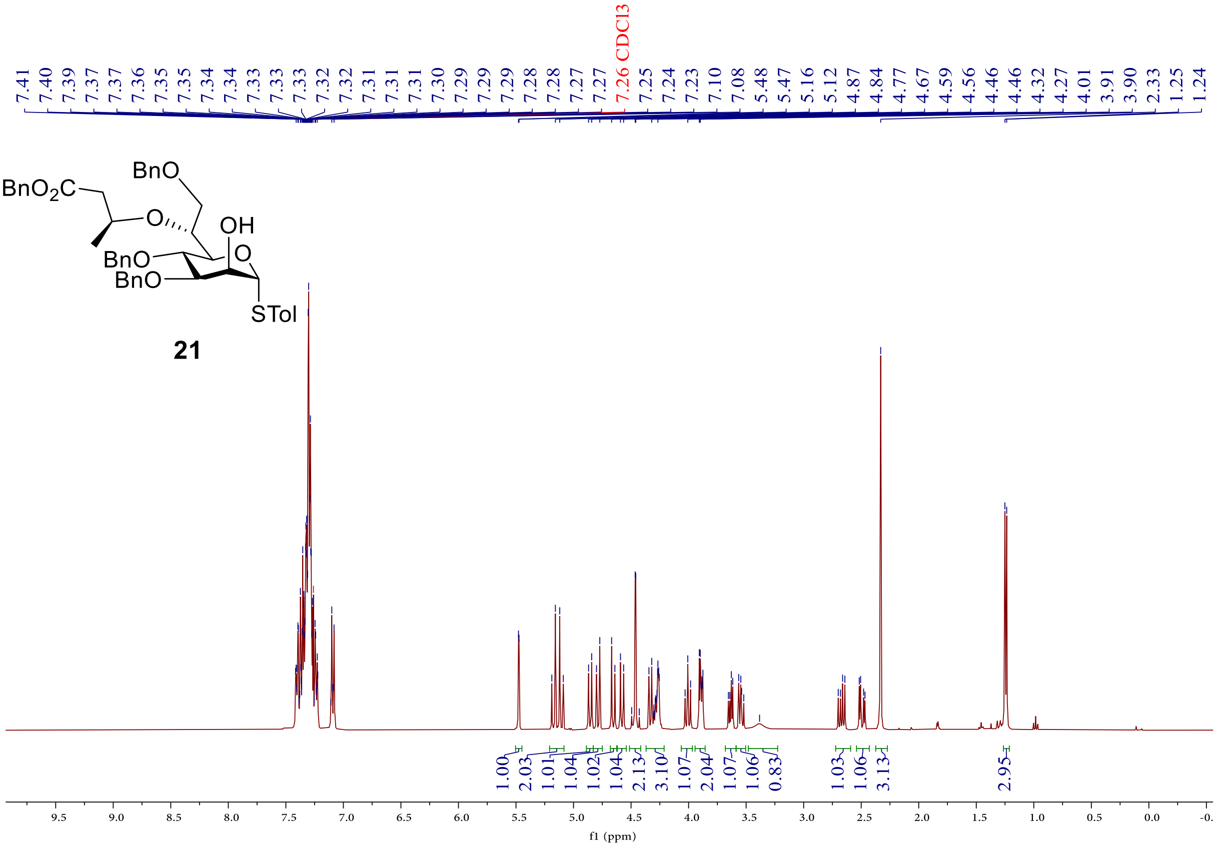
BnO
BnO_2C
O
OH
O
BnO
BnO
STol
21
7.41
7.40
7.39
7.37
7.37
7.36
7.35
7.35
7.34
7.34
7.33
7.33
7.33
7.32
7.32
7.31
7.31
7.31
7.30
7.29
7.29
7.29
7.28
7.28
7.27
7.27
7.26 CDCl3
7.25
7.24
7.23
7.10
7.08
5.48
5.47
5.16
5.12
4.87
4.84
4.77
4.67
4.59
4.56
4.46
4.46
4.32
4.27
4.01
3.91
3.90
2.33
1.25
1.24
1.00
2.03
1.01
1.04
1.02
1.04
2.13
3.10
1.07
2.04
1.07
1.06
0.83
1.03
1.06
3.13
2.95
f1 (ppm)

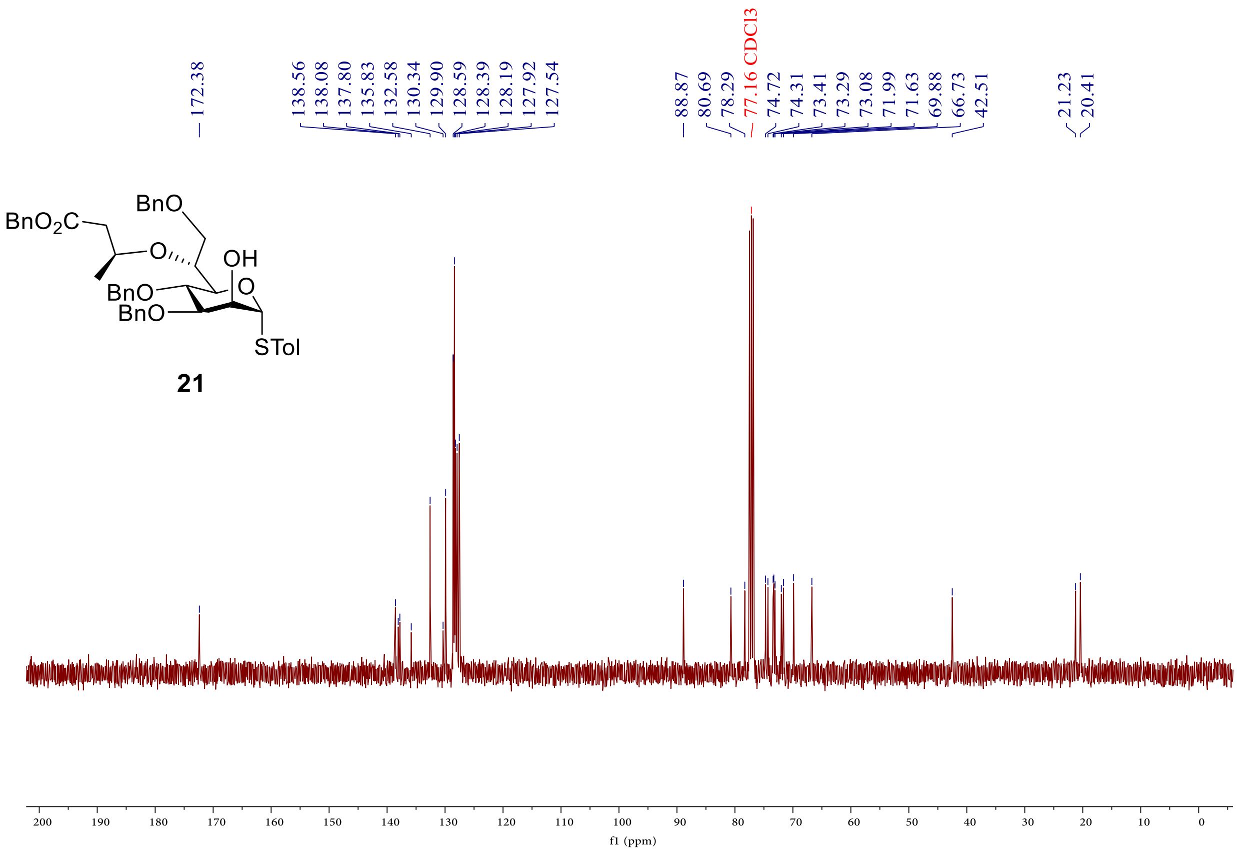

172.38
138.56 138.08 137.80 135.83 132.58 130.34 129.90 128.59 128.39 128.19 127.92 127.54
88.87 80.69 78.29 77.16 CDCl3 74.72 74.31 73.41 73.29 73.08 71.99 71.63 69.88 66.73 42.51
21.23 20.41
BnO
BnO2C
O
OH
O
BnO
BnO
STol
21
200 190 180 170 160 150 140 130 120 110 100 90 80 70 60 50 40 30 20 10 0
f1 (ppm)

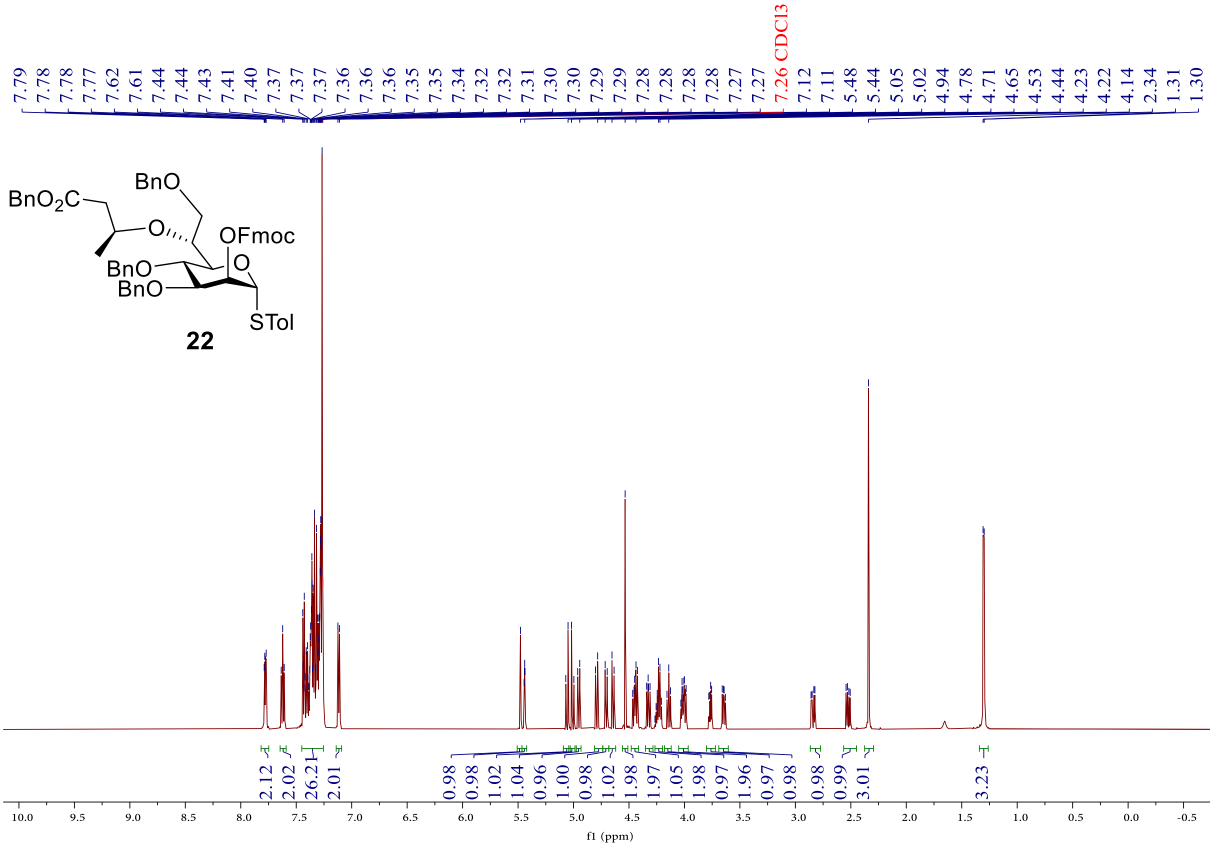

7.79 7.78 7.78 7.77 7.62 7.61 7.44 7.44 7.43 7.41 7.40 7.37 7.37 7.37 7.36 7.36 7.36 7.35 7.34 7.32 7.32 7.31 7.30 7.29 7.29 7.28 7.28 7.28 7.28 7.27 7.27 7.26 CDCl3 7.12 7.11 5.48 5.44 5.05 5.02 4.94 4.78 4.71 4.65 4.53 4.44 4.23 4.22 4.14 2.34 1.31 1.30
BnO
BnO2C
O
OFmoc
O
BnO
BnO
STol
22
2.12 2.02 26.21 2.01 0.98 0.98 1.02 1.04 0.96 1.00 0.98 1.02 1.98 1.97 1.05 1.98 0.97 1.96 0.97 0.98 0.98 0.99 3.01 3.23
10.0 9.5 9.0 8.5 8.0 7.5 7.0 6.5 6.0 5.5 5.0 4.5 4.0 3.5 3.0 2.5 2.0 1.5 1.0 0.5 0.0 -0.5
f1 (ppm)

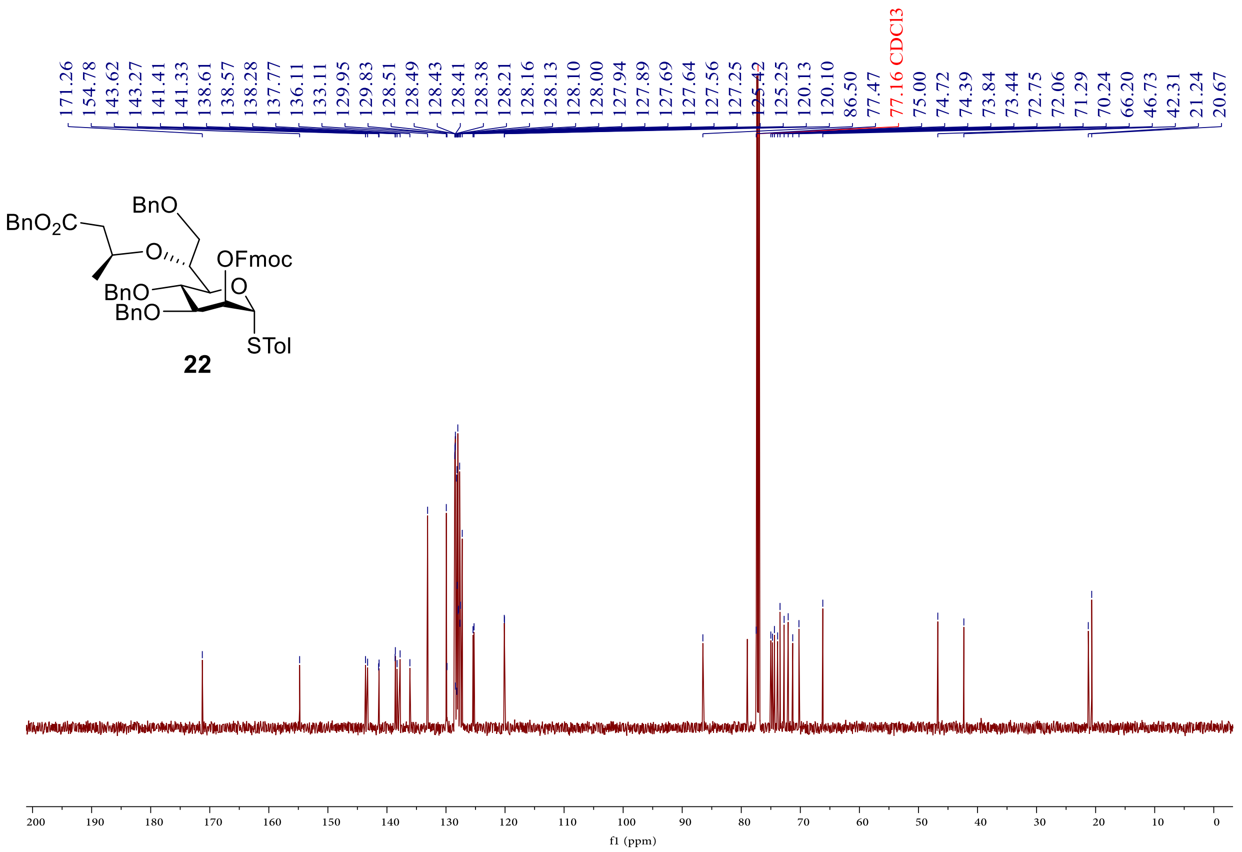
171.26
154.78
143.62
143.27
141.41
141.33
138.61
138.57
138.28
137.77
136.11
133.11
129.95
129.83
128.51
128.49
128.43
128.41
128.38
128.21
128.16
128.13
128.10
128.00
127.94
127.89
127.69
127.64
127.56
127.25
125.42
125.25
120.13
120.10
86.50
77.47
77.16 CDCl13
75.00
74.72
74.39
73.84
73.44
72.75
72.06
71.29
70.24
66.20
46.73
42.31
21.24
20.67
BnO
BnO2C
OFmoc
BnO
BnO
STol
22
f1 (ppm)

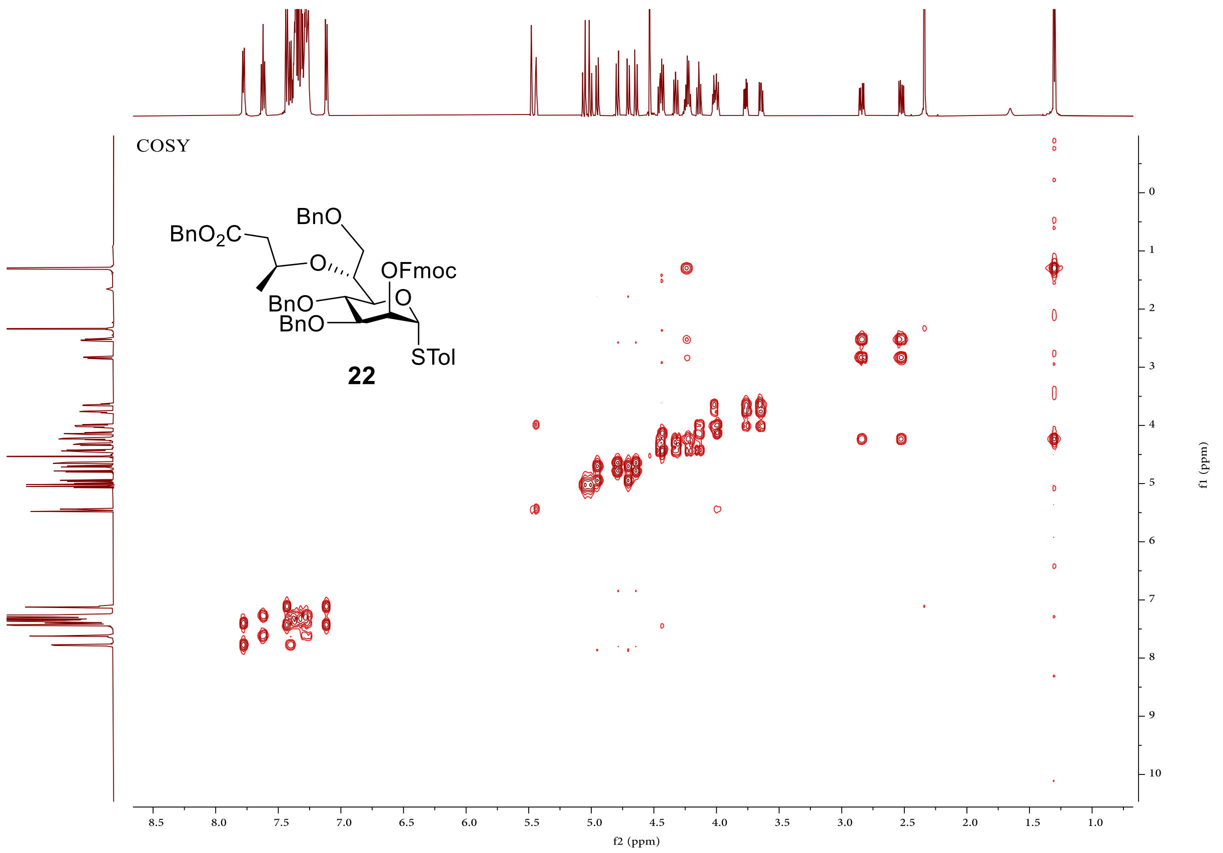
COSY
BnO
BnO2C
OFmoc
BnO
BnO
STol
22
f2 (ppm)
f1 (ppm)

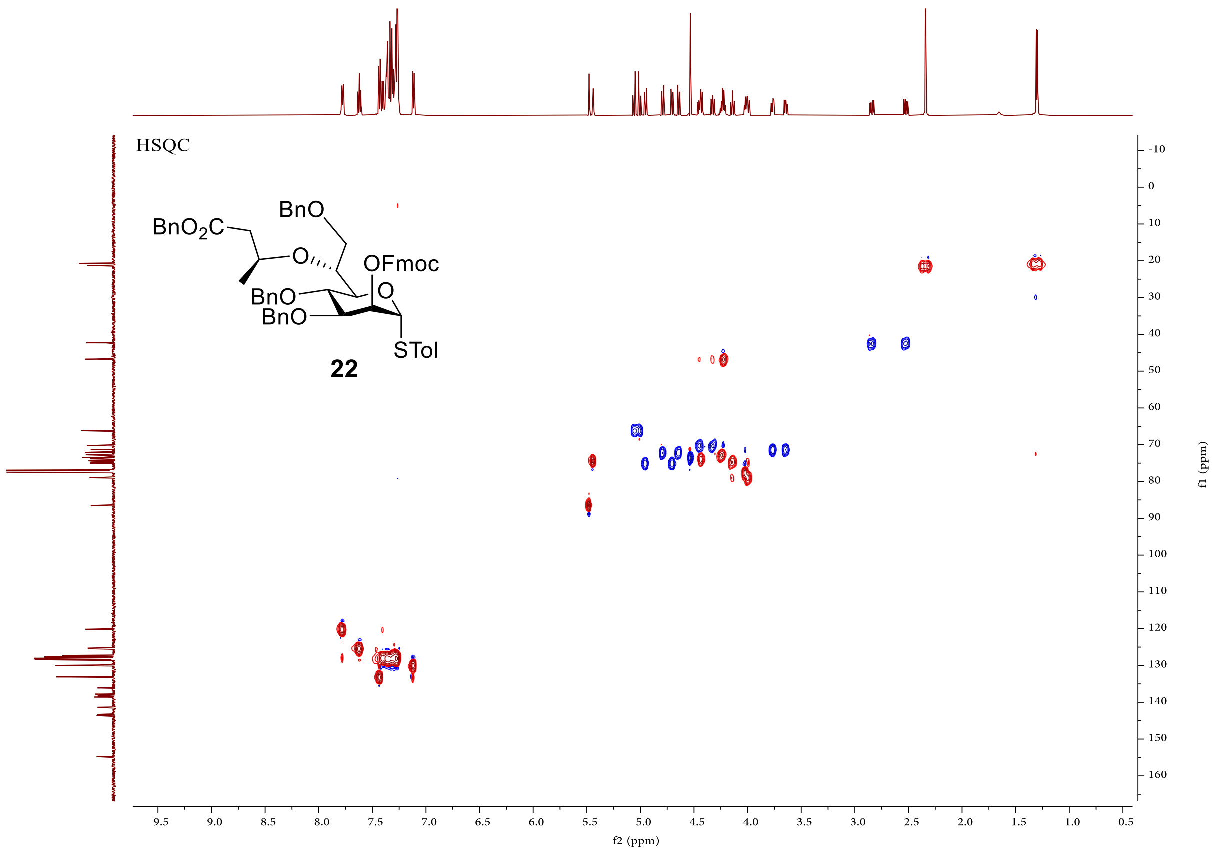
HSQC
BnO
BnO2C
O
OFmoc
O
BnO
BnO
STol
22
f2 (ppm)
f1 (ppm)

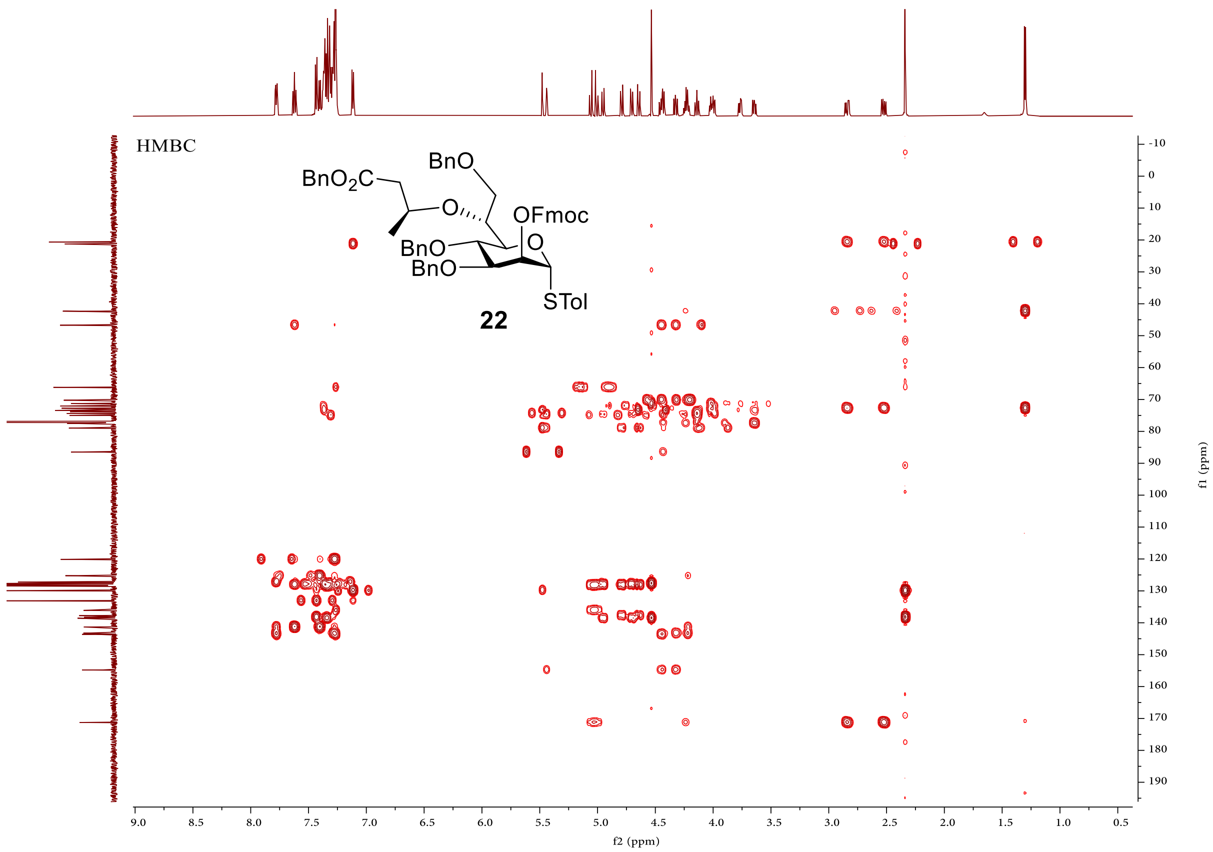
HMBC
BnO
BnO2C
O
OFmoc
O
BnO
BnO
STol
22
f2 (ppm)
f1 (ppm)

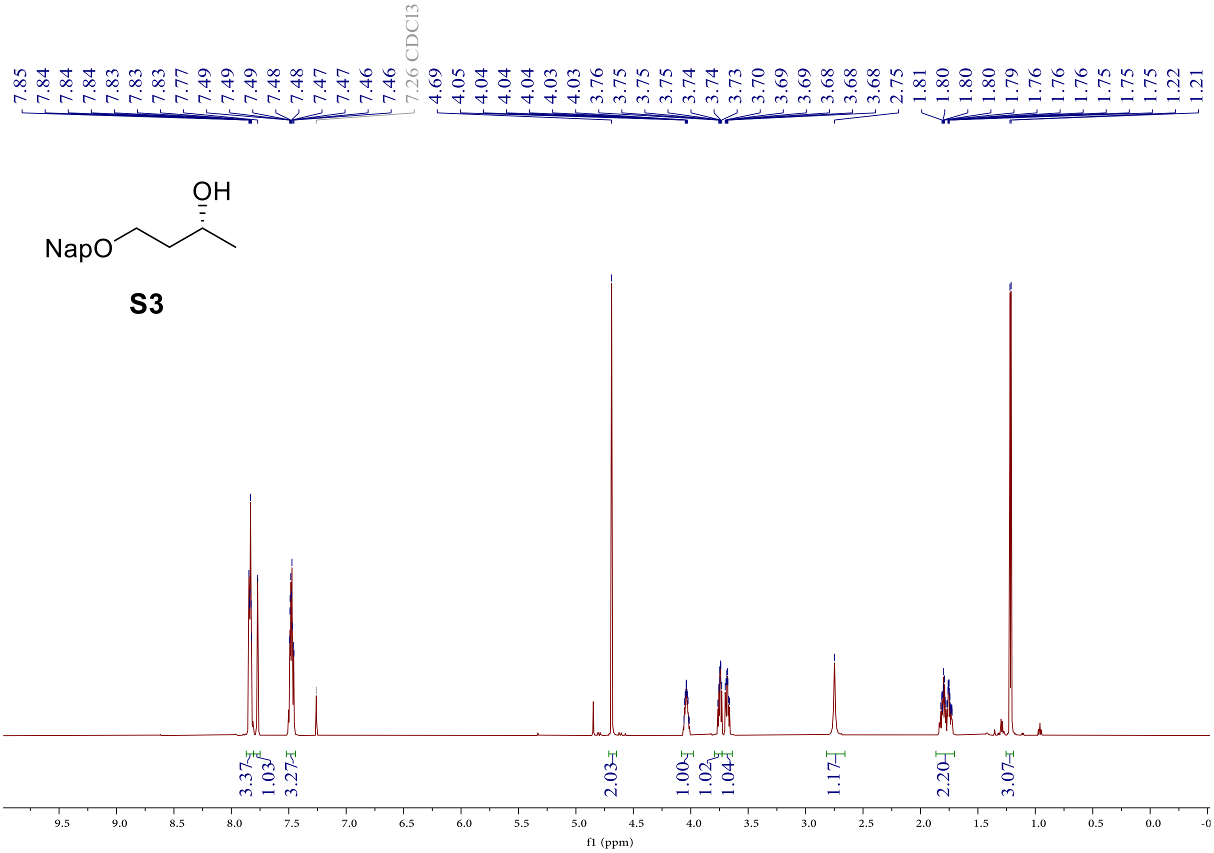
7.85 7.84 7.84 7.84 7.83 7.83 7.83 7.77 7.49 7.49 7.49 7.48 7.48 7.47 7.47 7.46 7.46 7.26 CDCl3 4.69 4.05 4.04 4.04 4.04 4.03 4.03 3.76 3.75 3.75 3.75 3.74 3.74 3.73 3.70 3.69 3.69 3.68 3.68 3.68 2.75 1.81 1.80 1.80 1.80 1.79 1.76 1.76 1.75 1.75 1.75 1.22 1.21
OH
NapO
S3
3.37 1.03 3.27 2.03 1.00 1.02 1.04 1.17 2.20 3.07
f1 (ppm)

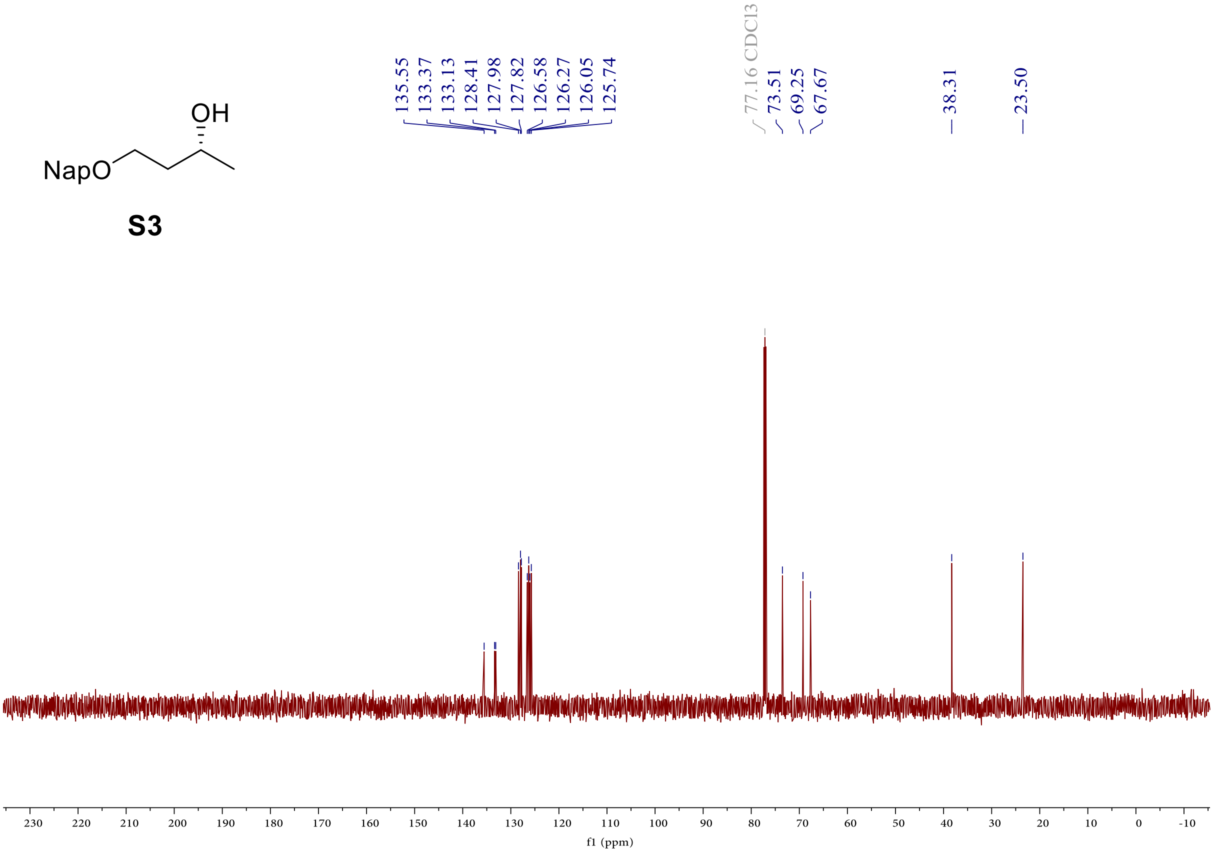
135.55 133.37 133.13 128.41 127.98 127.82 126.58 126.27 126.05 125.74
77.16 CDCl3 73.51 69.25 67.67
38.31
23.50
OH
NapO
S3
f1 (ppm)

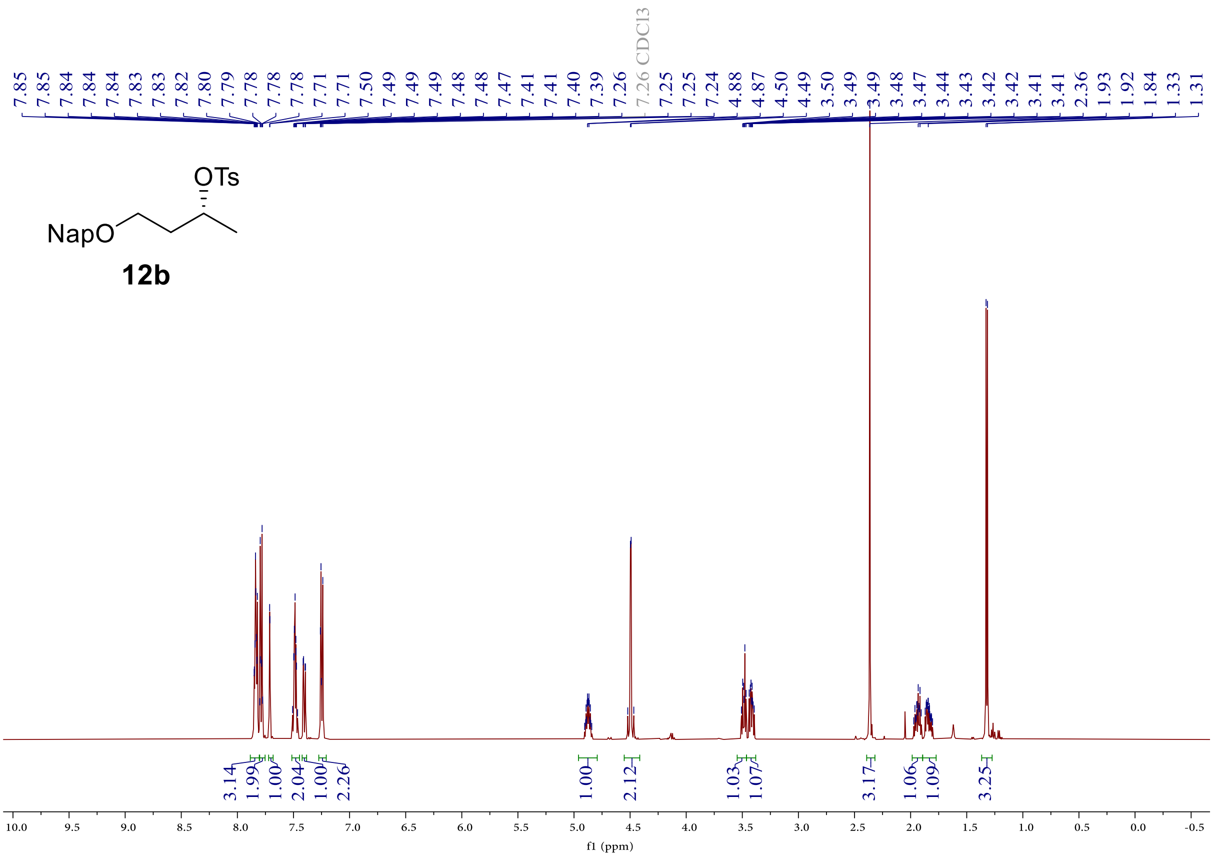
7.85
7.85
7.84
7.84
7.84
7.83
7.83
7.82
7.80
7.79
7.78
7.78
7.78
7.71
7.71
7.50
7.49
7.49
7.49
7.48
7.48
7.47
7.41
7.41
7.40
7.39
7.26
7.26 CDCl3
7.25
7.25
7.24
4.88
4.87
4.50
4.49
3.50
3.49
3.49
3.48
3.47
3.44
3.43
3.42
3.42
3.41
3.41
2.36
1.93
1.92
1.84
1.33
1.31
OTs
NapO
12b
3.14
1.99
1.00
2.04
1.00
2.26
1.00
2.12
1.03
1.07
3.17
1.06
1.09
3.25
f1 (ppm)

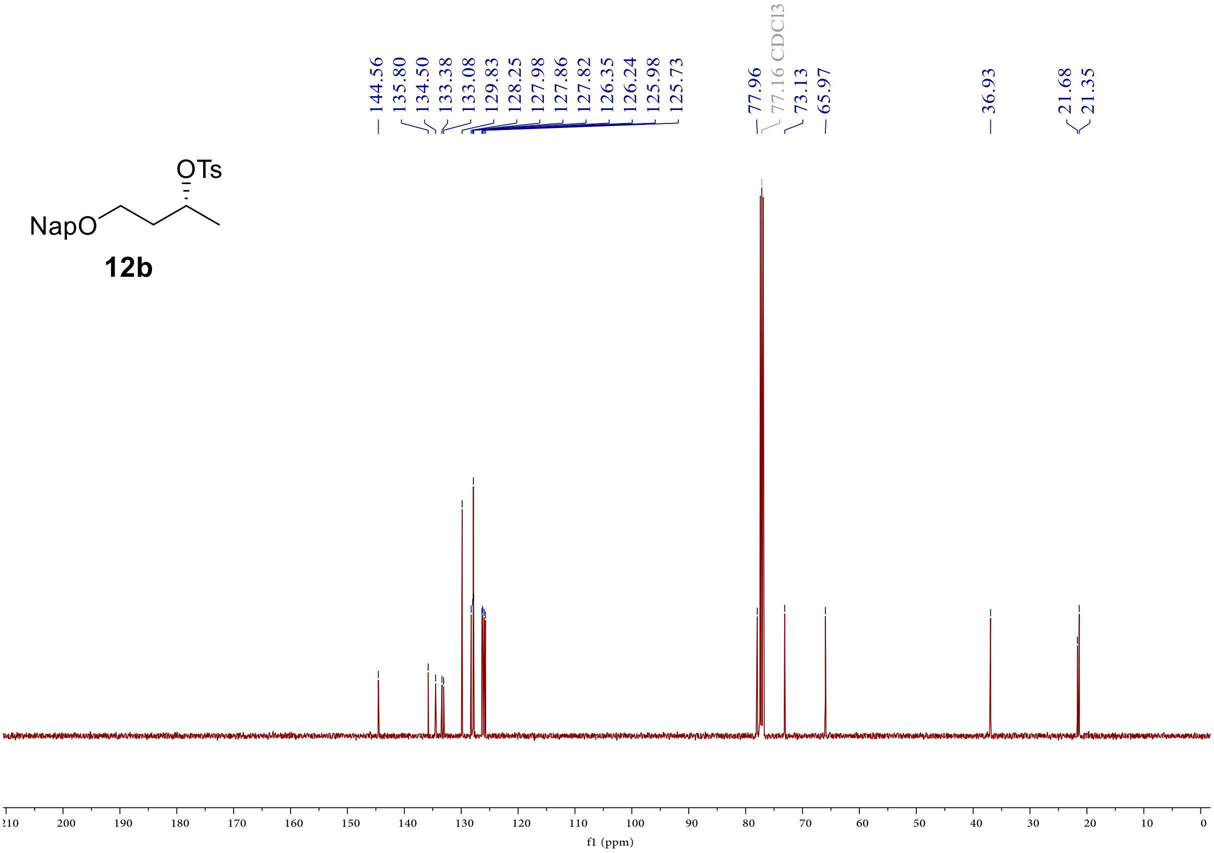
144.56
135.80
134.50
133.38
133.08
129.83
128.25
127.98
127.86
127.82
126.35
126.24
125.98
125.73
77.96
77.16 CDCl3
73.13
65.97
36.93
21.68
21.35
OTs
NapO
12b
f1 (ppm)

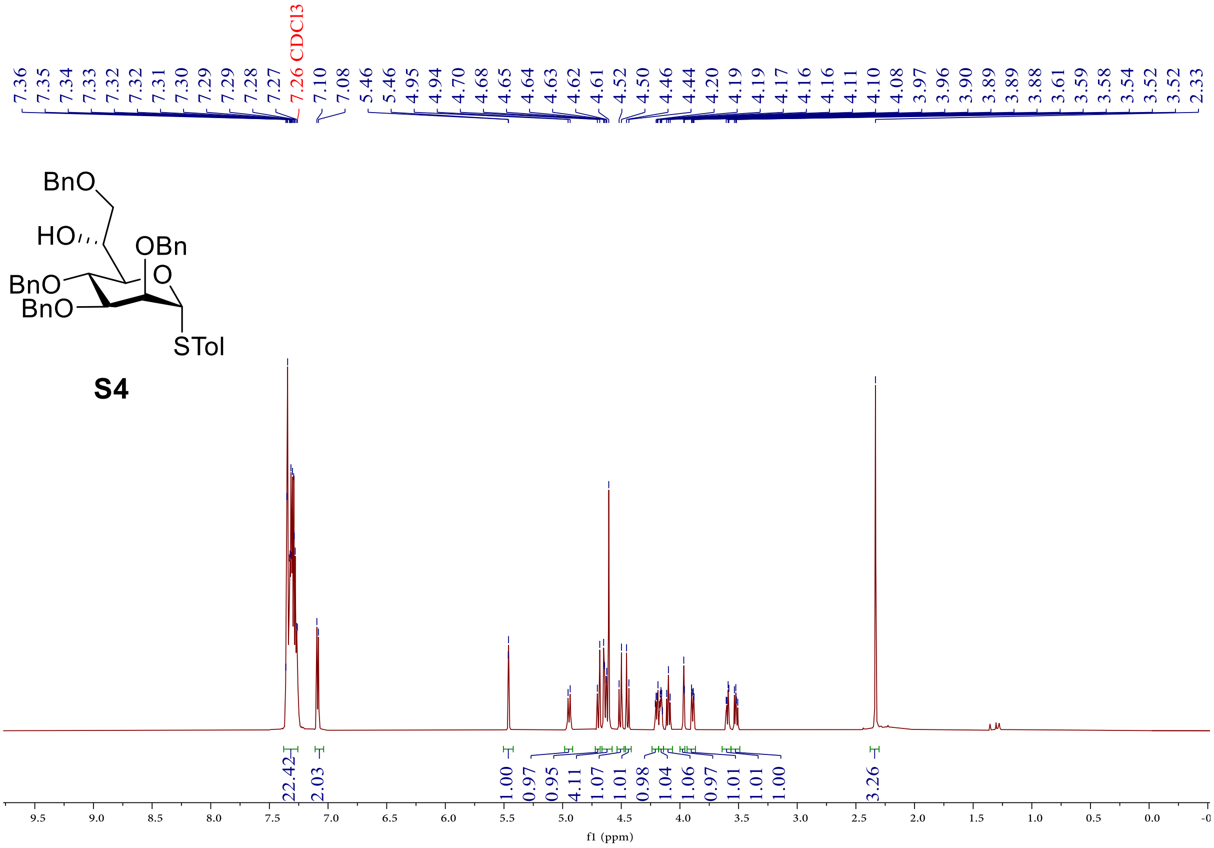

g0-hth-5-35-1108

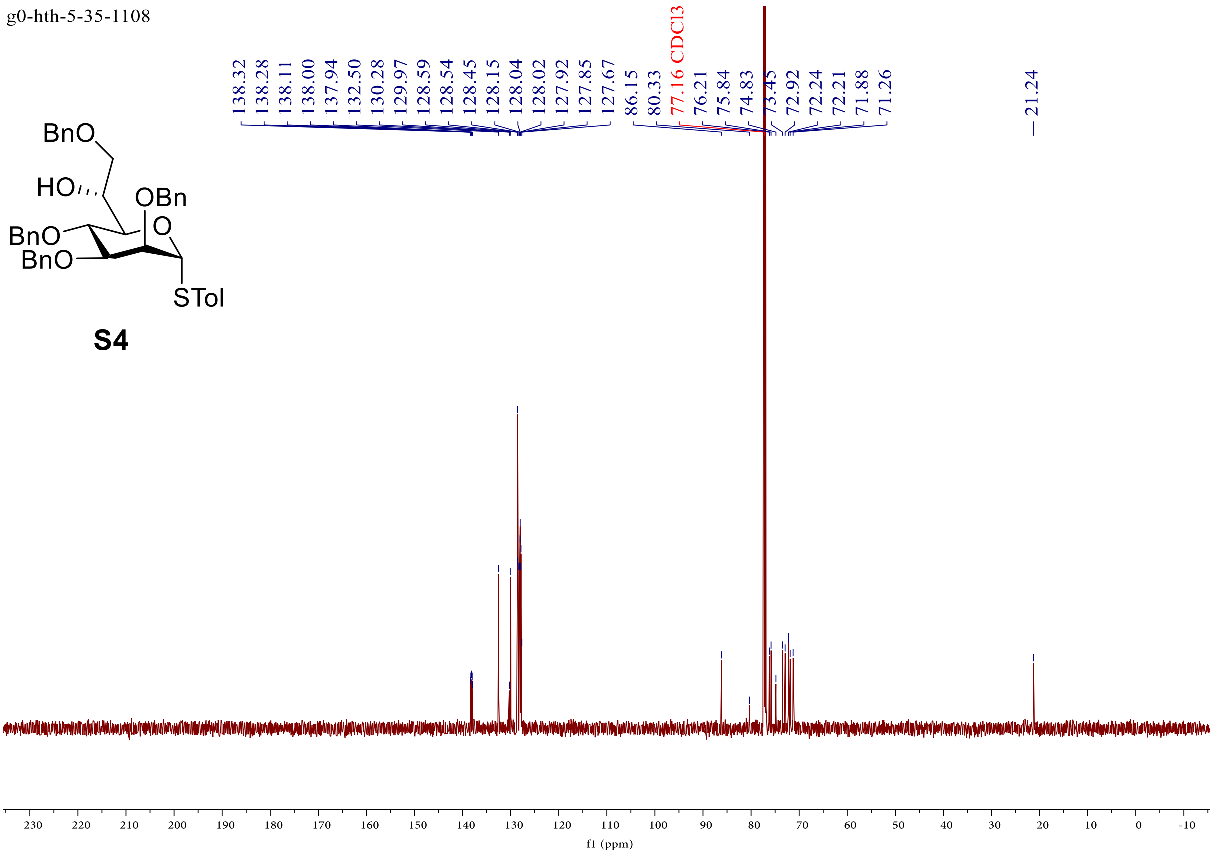

S5

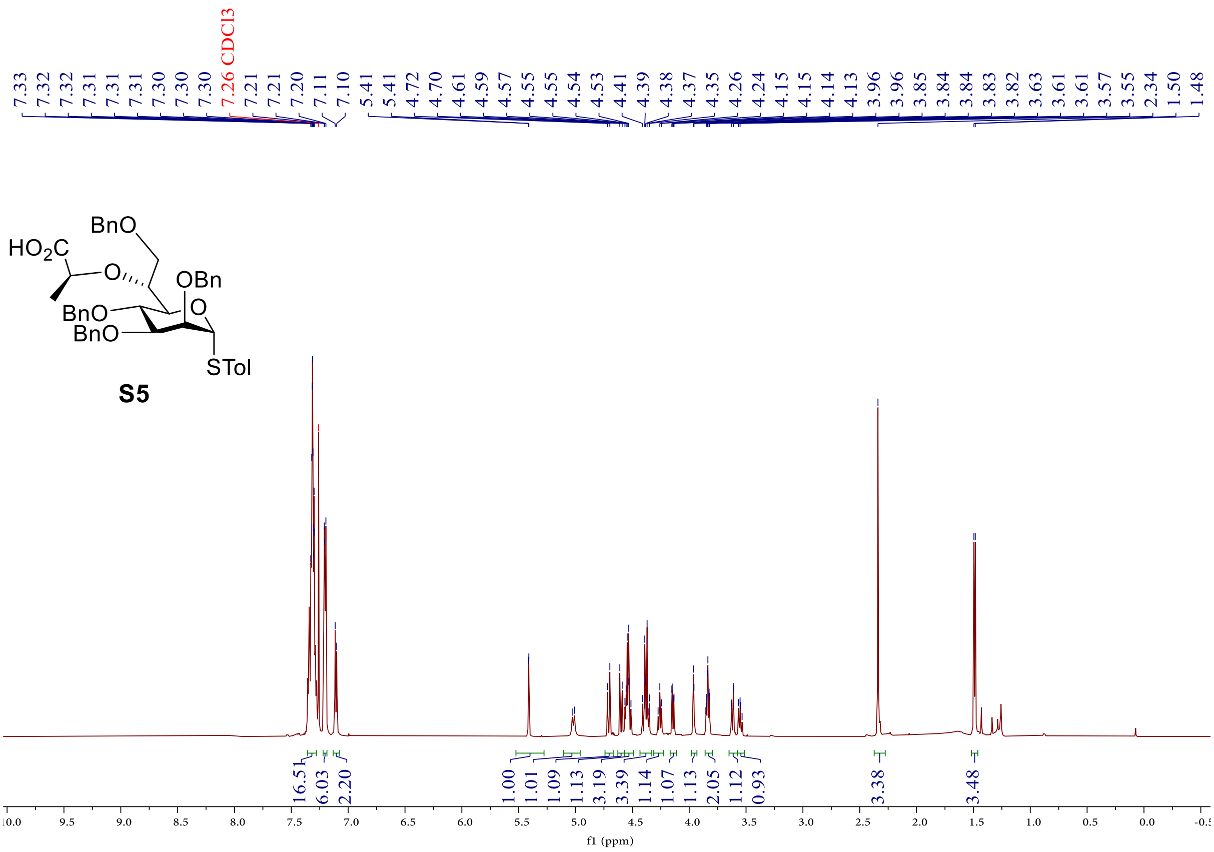
7.33 7.32 7.32 7.31 7.31 7.31 7.30 7.30 7.30 7.26 CDCl3 7.21 7.21 7.20 7.11 7.10 5.41 5.41 4.72 4.70 4.61 4.59 4.57 4.55 4.55 4.54 4.53 4.41 4.39 4.38 4.37 4.35 4.26 4.24 4.15 4.15 4.14 4.13 3.96 3.96 3.85 3.84 3.84 3.83 3.82 3.63 3.61 3.61 3.57 3.55 2.34 1.50 1.48
HO2C
BnO
OBn
BnO
BnO
O
STol
S5
16.51 6.03 2.20 1.00 1.01 1.09 1.13 3.19 3.39 1.14 1.07 1.13 2.05 1.12 0.93 3.38 3.48
f1 (ppm)

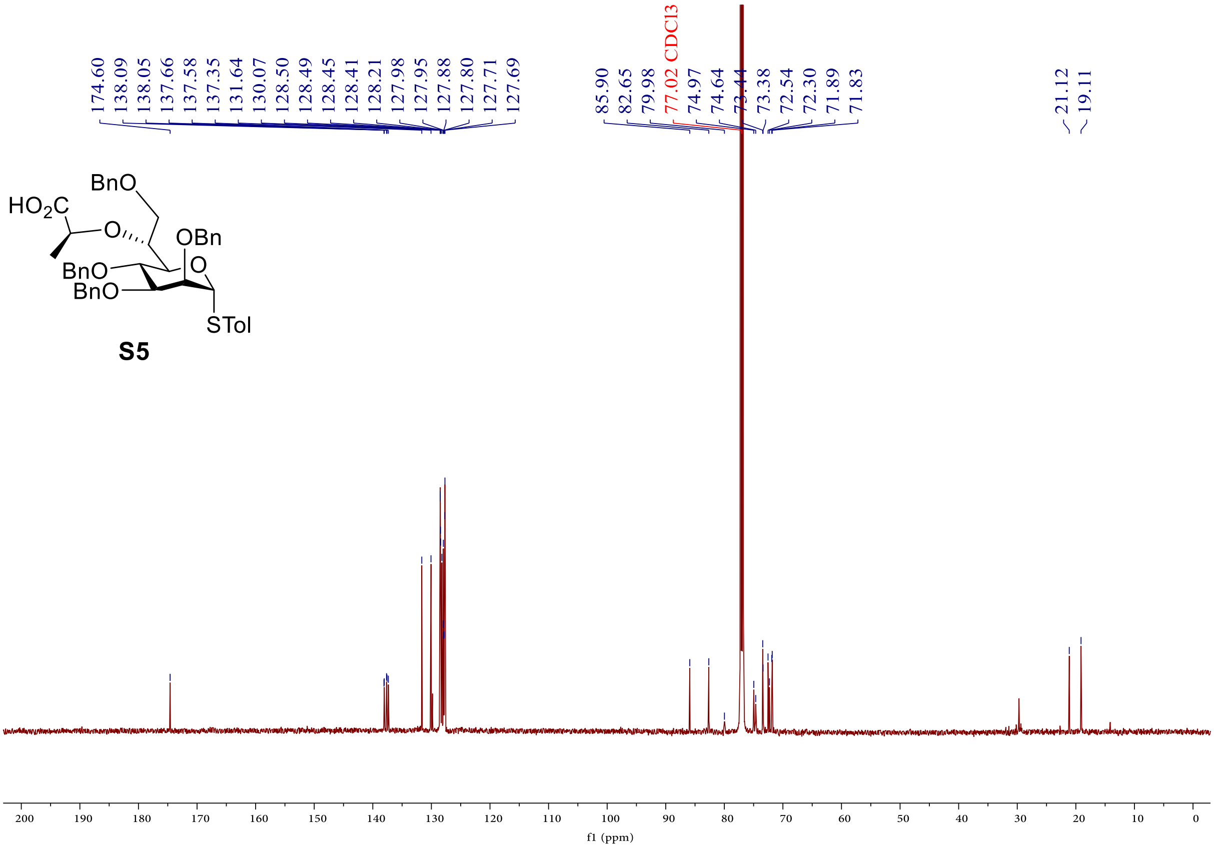
174.60 138.09 138.05 137.66 137.58 137.35 131.64 130.07 128.50 128.49 128.45 128.41 128.21 127.98 127.95 127.88 127.80 127.71 127.69 85.90 82.65 79.98 77.02 CDCl3 74.97 74.64 73.44 73.38 72.54 72.30 71.89 71.83 21.12 19.11
HO2C
BnO
OBn
BnO
BnO
O
STol
S5
f1 (ppm)

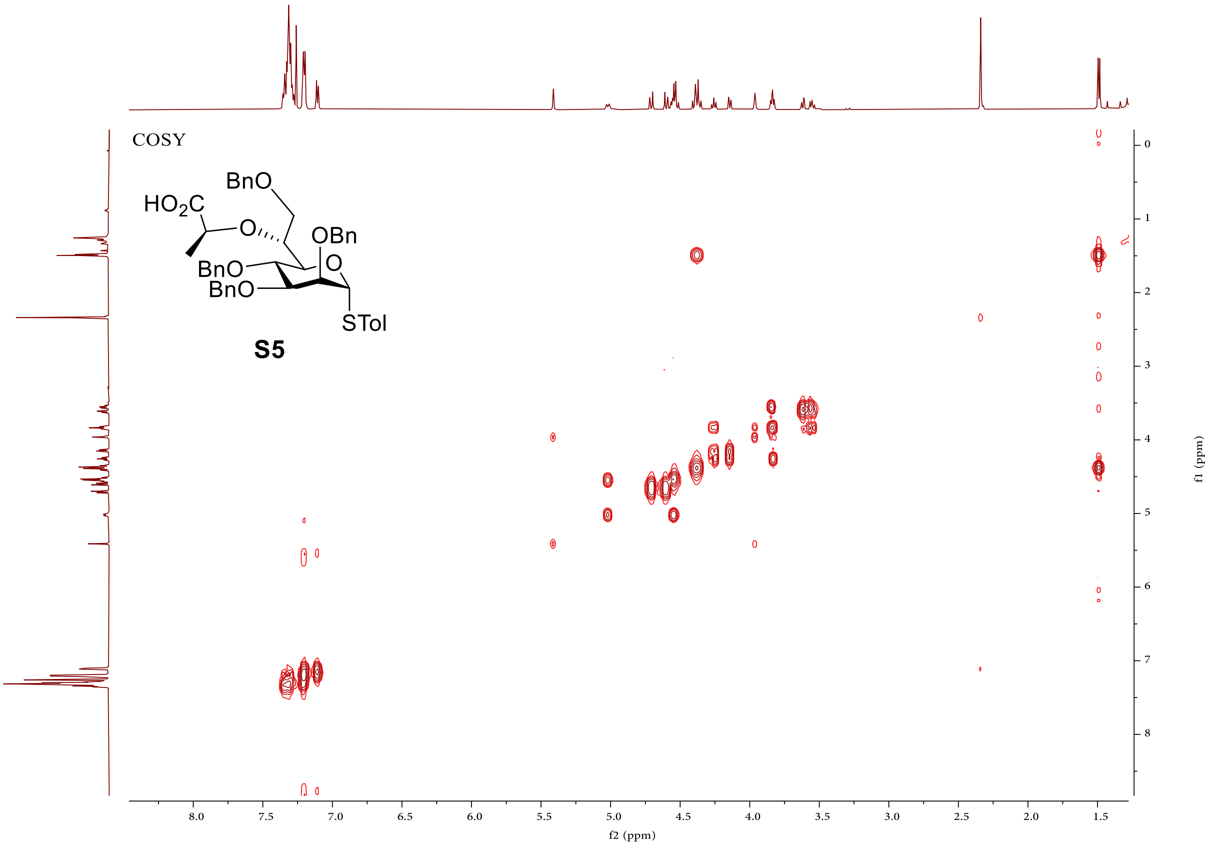
COSY
BnO
HO_2C
O
OBn
O
BnO
BnO
STol
S5
f2 (ppm)
f1 (ppm)

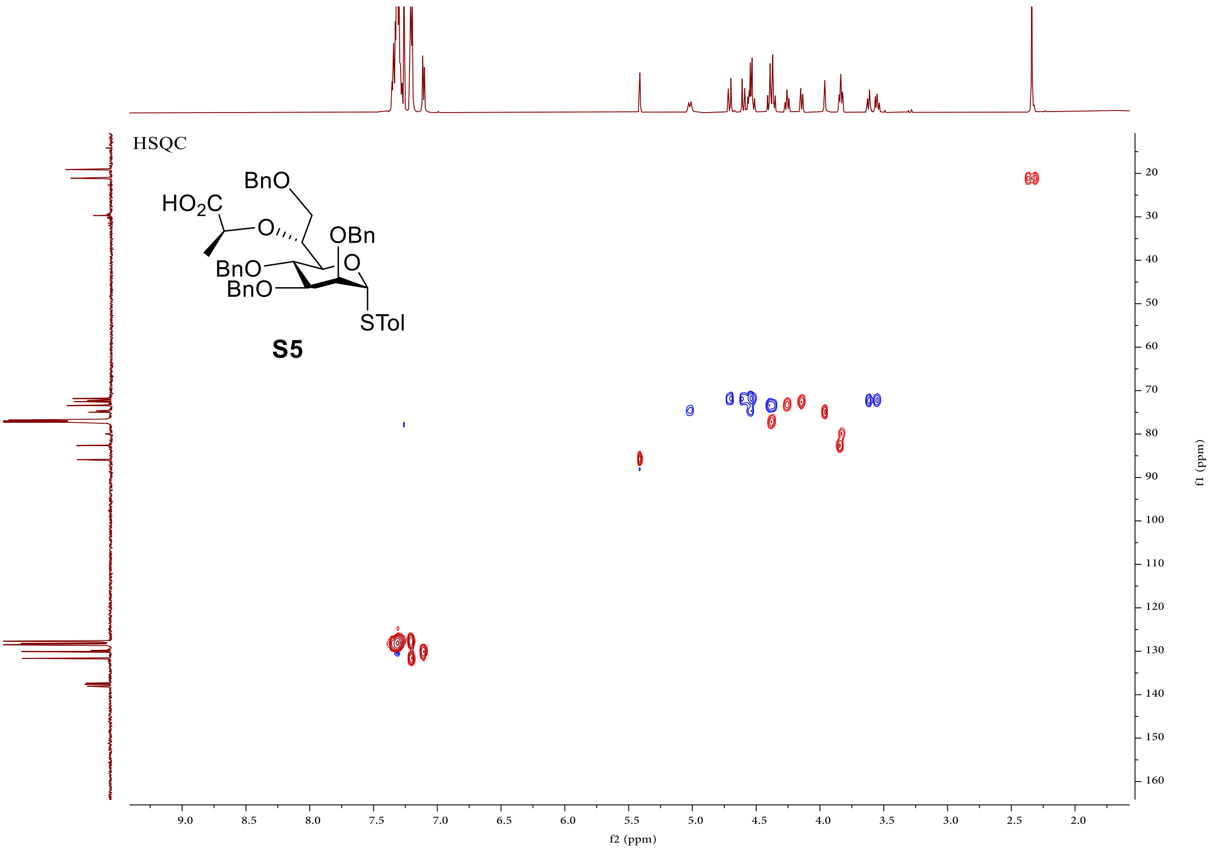
HSQC
BnO
HO_2C
O
OBn
O
BnO
BnO
STol
S5
f2 (ppm)
f1 (ppm)

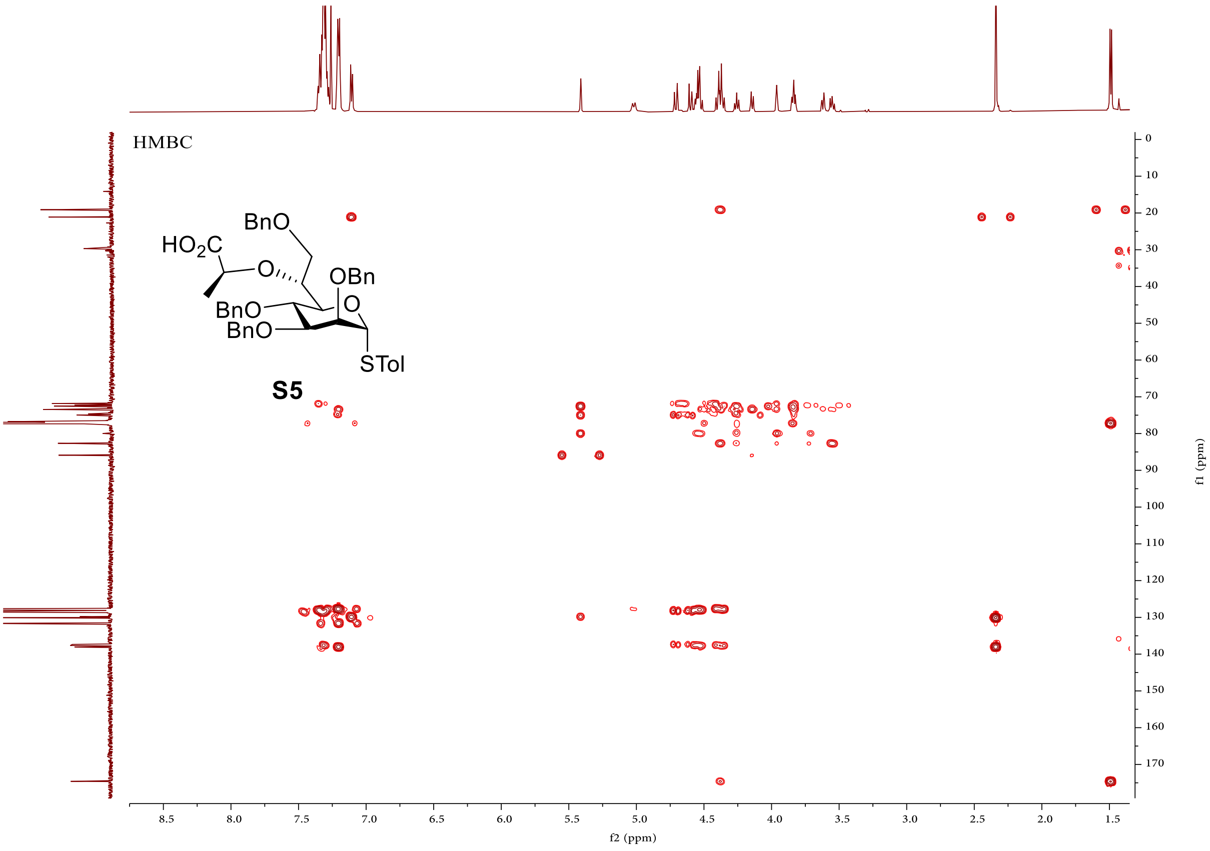
HMBC
BnO
HO_2C
OBn
BnO
BnO
O
STol
S5
f2 (ppm)
f1 (ppm)

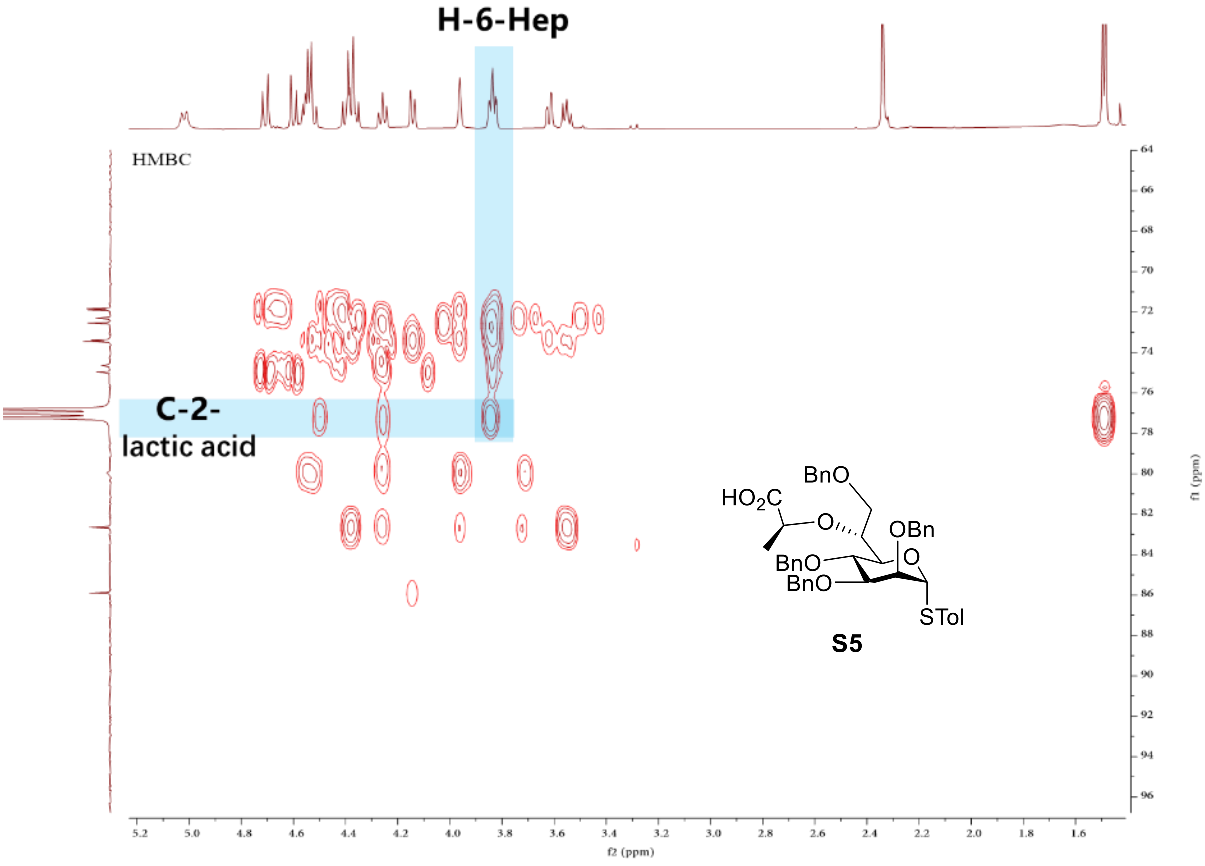
H-6-Hep
HMBC
C-2-
lactic acid
BnO
HO_2C
OBn
BnO
BnO
O
STol
S5
f2 (ppm)
f1 (ppm)

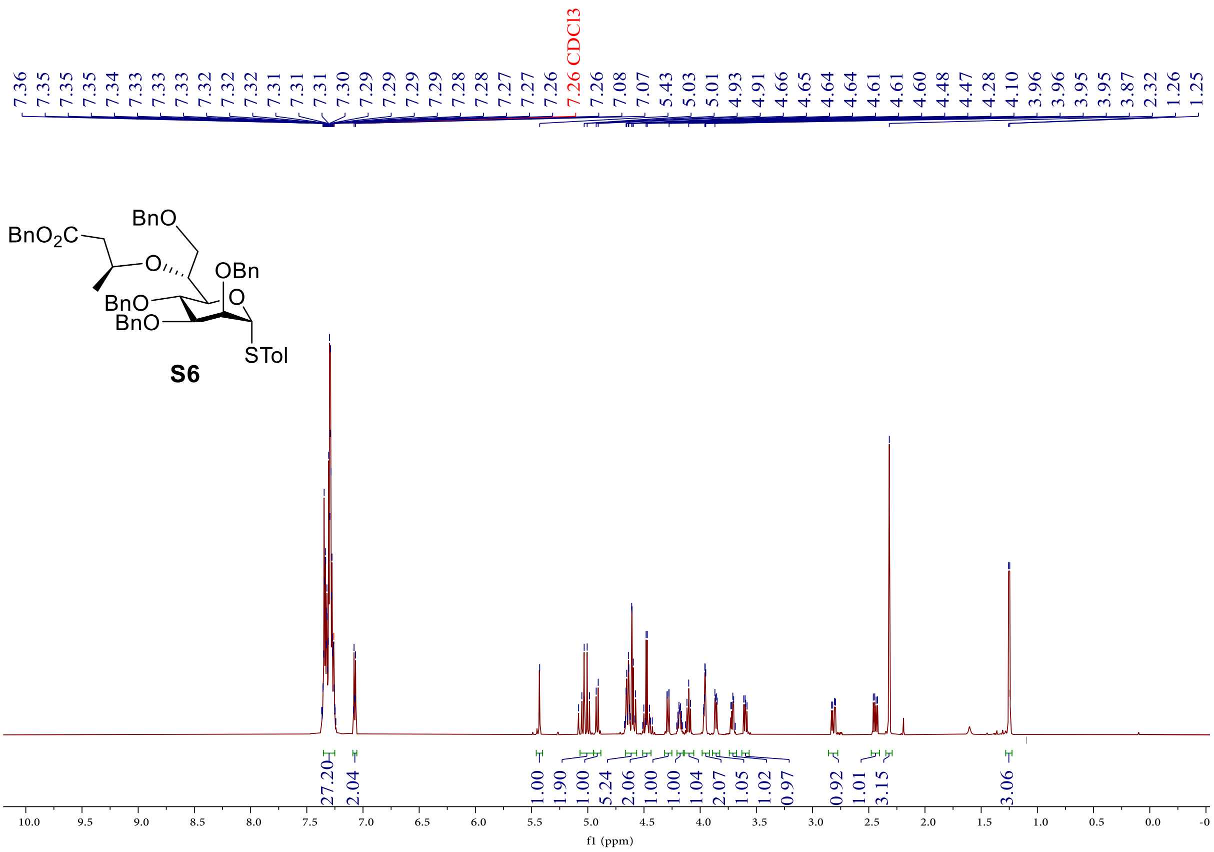
7.36 7.35 7.35 7.35 7.34 7.33 7.33 7.33 7.32 7.32 7.32 7.31 7.31 7.31 7.30 7.29 7.29 7.29 7.29 7.28 7.28 7.27 7.27 7.26 7.26 CDCl3 7.26 7.08 7.07 5.43 5.03 5.01 4.93 4.91 4.66 4.65 4.64 4.64 4.61 4.61 4.60 4.48 4.47 4.28 4.10 3.96 3.96 3.95 3.95 3.87 2.32 1.26 1.25
BnO₂C
BnO
O
OBn
BnO
BnO
O
STol
S6
27.20 2.04 1.00 1.90 1.00 5.24 2.06 1.00 1.00 1.04 2.07 1.05 1.02 0.97 0.92 1.01 3.15 3.06
10.0 9.5 9.0 8.5 8.0 7.5 7.0 6.5 6.0 5.5 5.0 4.5 4.0 3.5 3.0 2.5 2.0 1.5 1.0 0.5 0.0 -0
f1 (ppm)

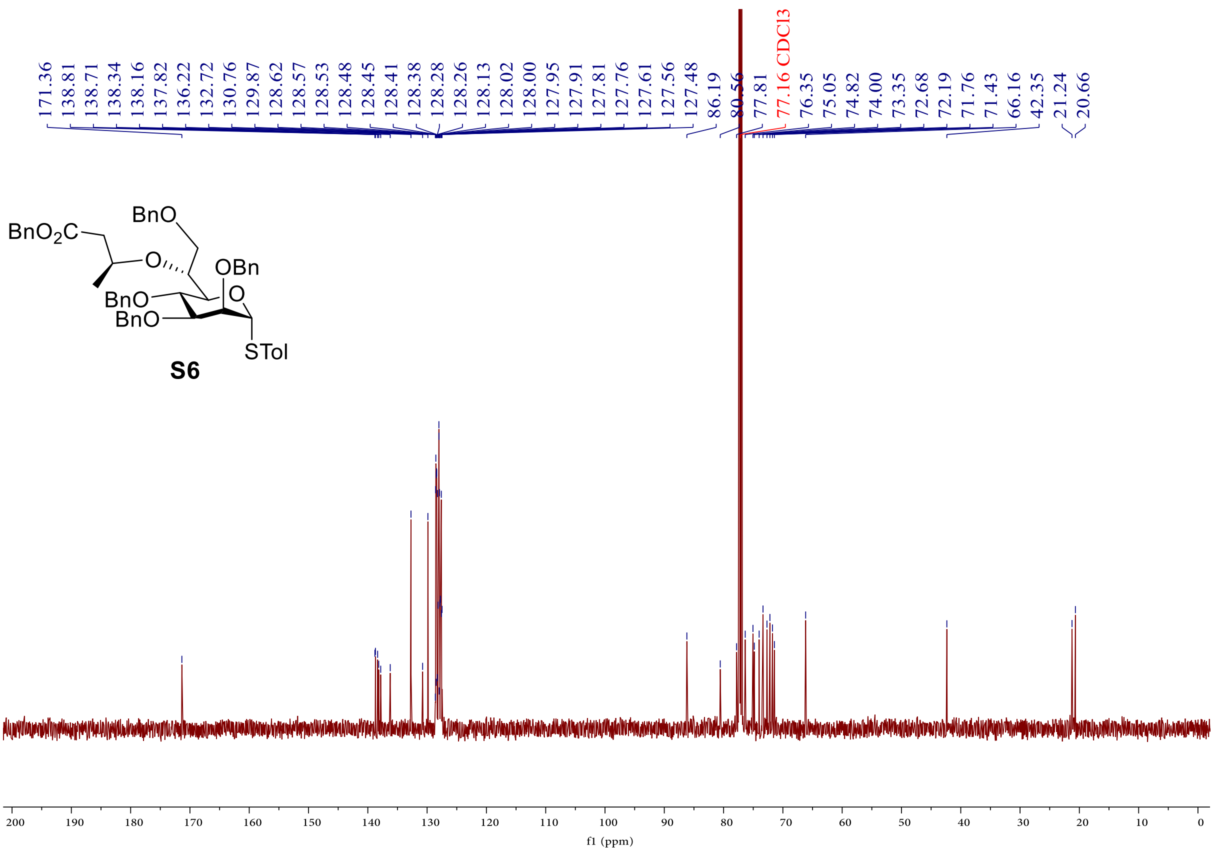
171.36 138.81 138.71 138.34 138.16 137.82 136.22 132.72 130.76 129.87 128.62 128.57 128.53 128.48 128.45 128.41 128.38 128.28 128.26 128.13 128.02 128.00 127.95 127.91 127.81 127.76 127.61 127.56 127.48 86.19 80.56 77.81 77.16 CDCl3 76.35 75.05 74.82 74.00 73.35 72.68 72.19 71.76 71.43 66.16 42.35 21.24 20.66
BnO₂C
BnO
O
OBn
BnO
BnO
O
STol
S6
200 190 180 170 160 150 140 130 120 110 100 90 80 70 60 50 40 30 20 10 0
f1 (ppm)

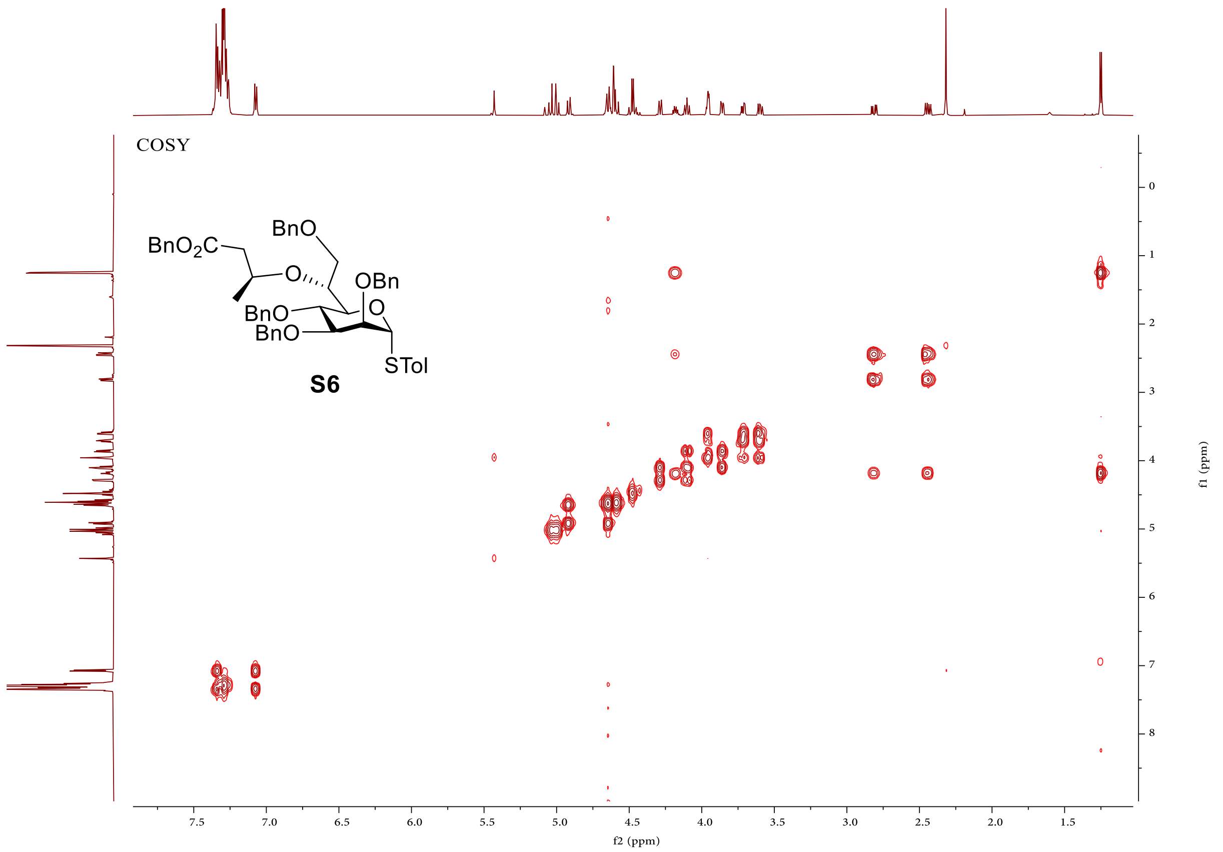
COSY
BnO
BnO2C
O
OBn
BnO
O
BnO
STol
S6
f2 (ppm)
f1 (ppm)

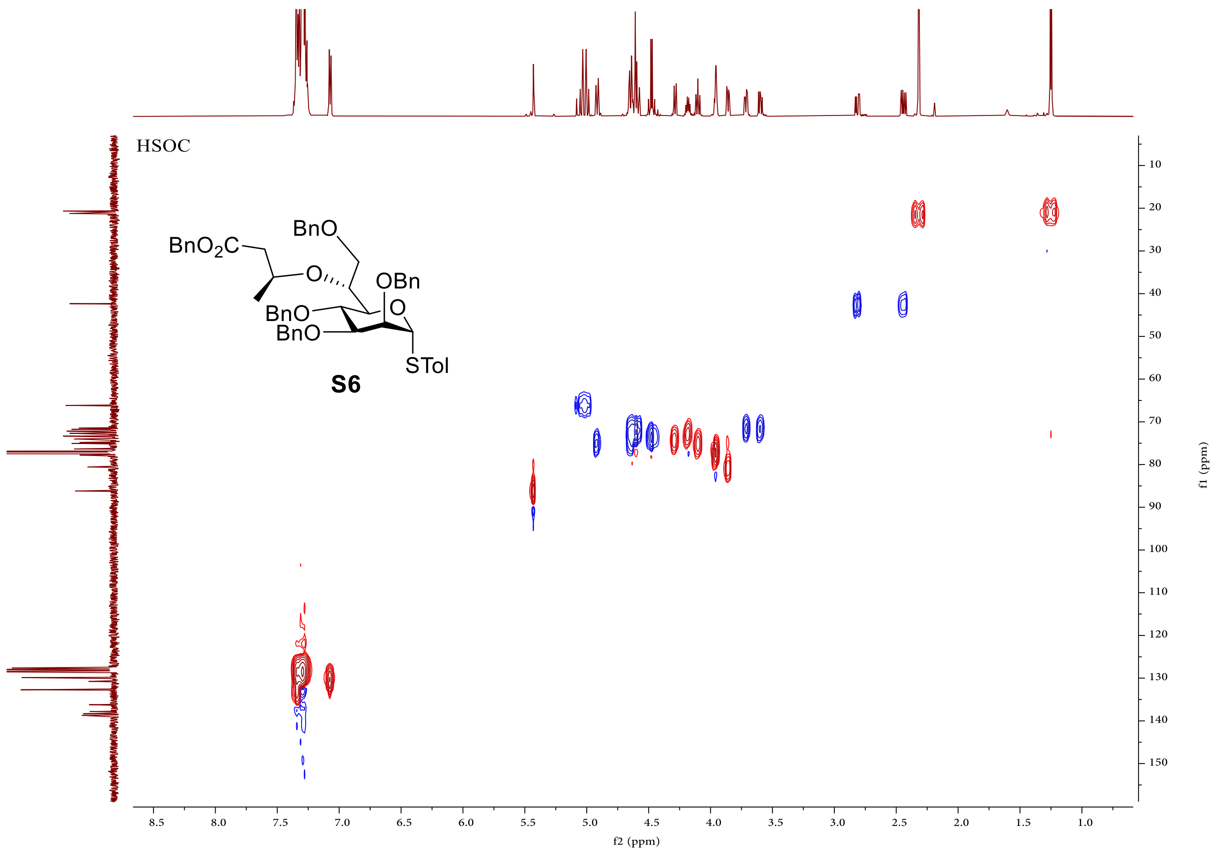
HSQC
BnO
BnO2C
O
OBn
BnO
O
BnO
STol
S6
f2 (ppm)
f1 (ppm)

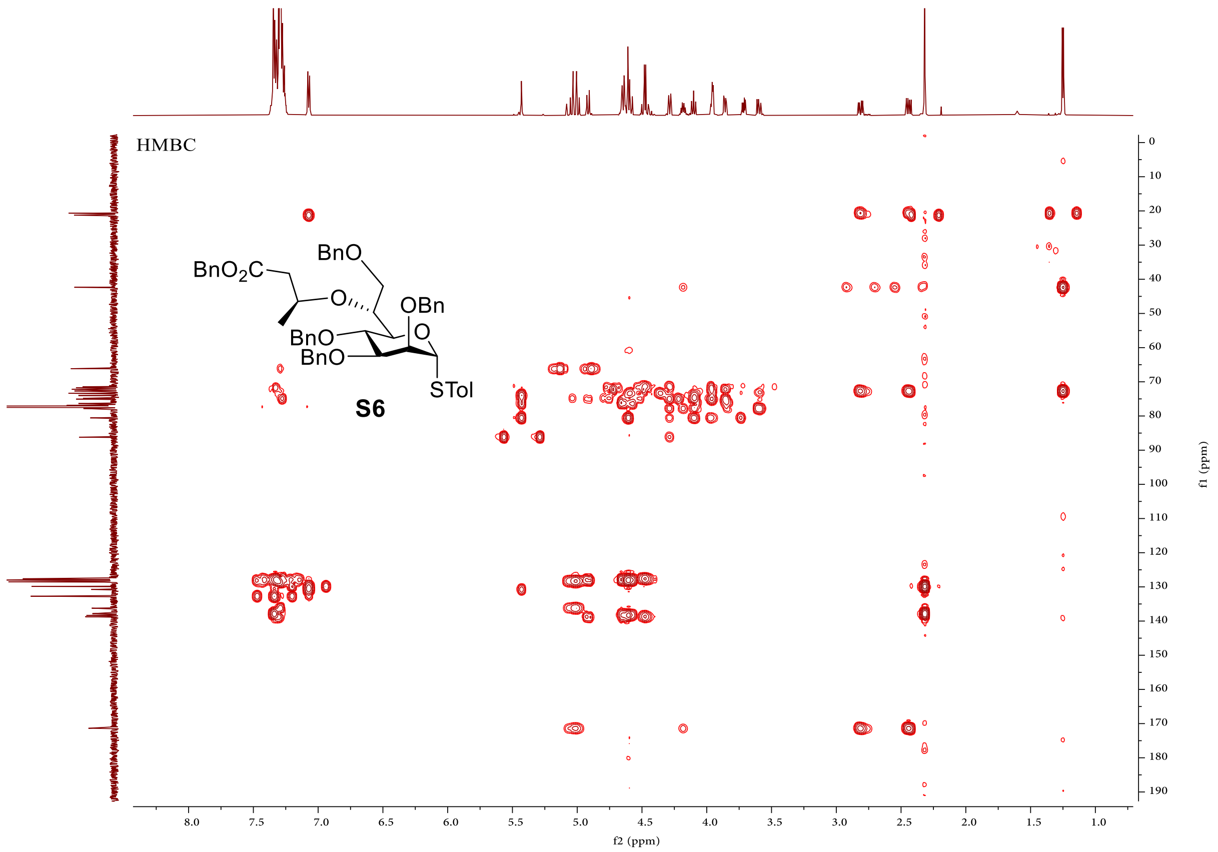
HMBC
BnO
BnO$_2$C
O
OBn
BnO
BnO
O
STol
S6
f2 (ppm)
f1 (ppm)

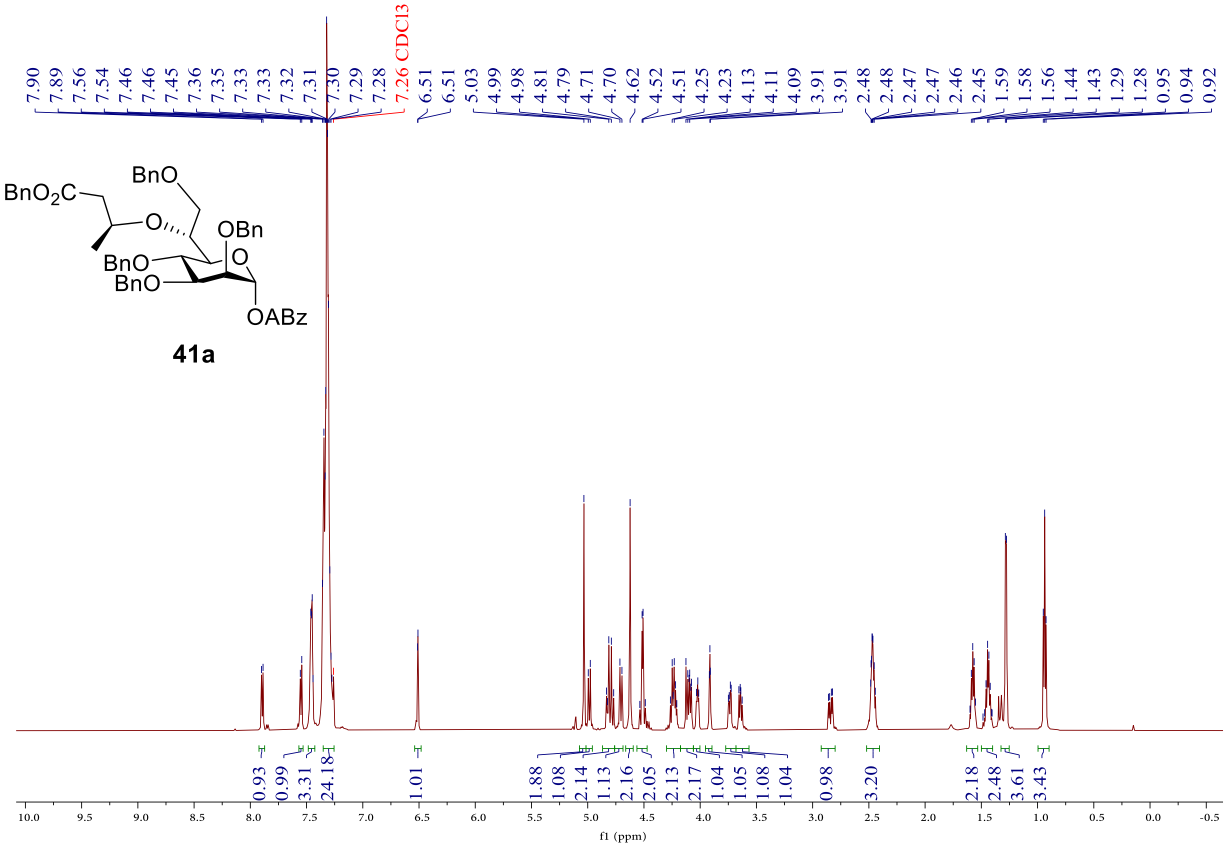
7.90 7.89 7.56 7.54 7.46 7.45 7.36 7.35 7.33 7.33 7.32 7.31 7.30 7.29 7.28 7.26 CDCl3 6.51 6.51 5.03 4.99 4.98 4.81 4.79 4.71 4.70 4.62 4.52 4.51 4.25 4.23 4.13 4.11 4.09 3.91 3.91 2.48 2.48 2.47 2.47 2.46 2.45 1.59 1.58 1.56 1.44 1.43 1.29 1.28 0.95 0.94 0.92
BnO
BnO$_2$C
O
OBn
BnO
BnO
O
OABz
41a
0.93 0.99 3.31 24.18 1.01 1.88 1.08 2.14 1.13 2.16 2.05 2.13 2.17 1.04 1.05 1.08 1.04 0.98 3.20 2.18 2.48 3.61 3.43
f1 (ppm)

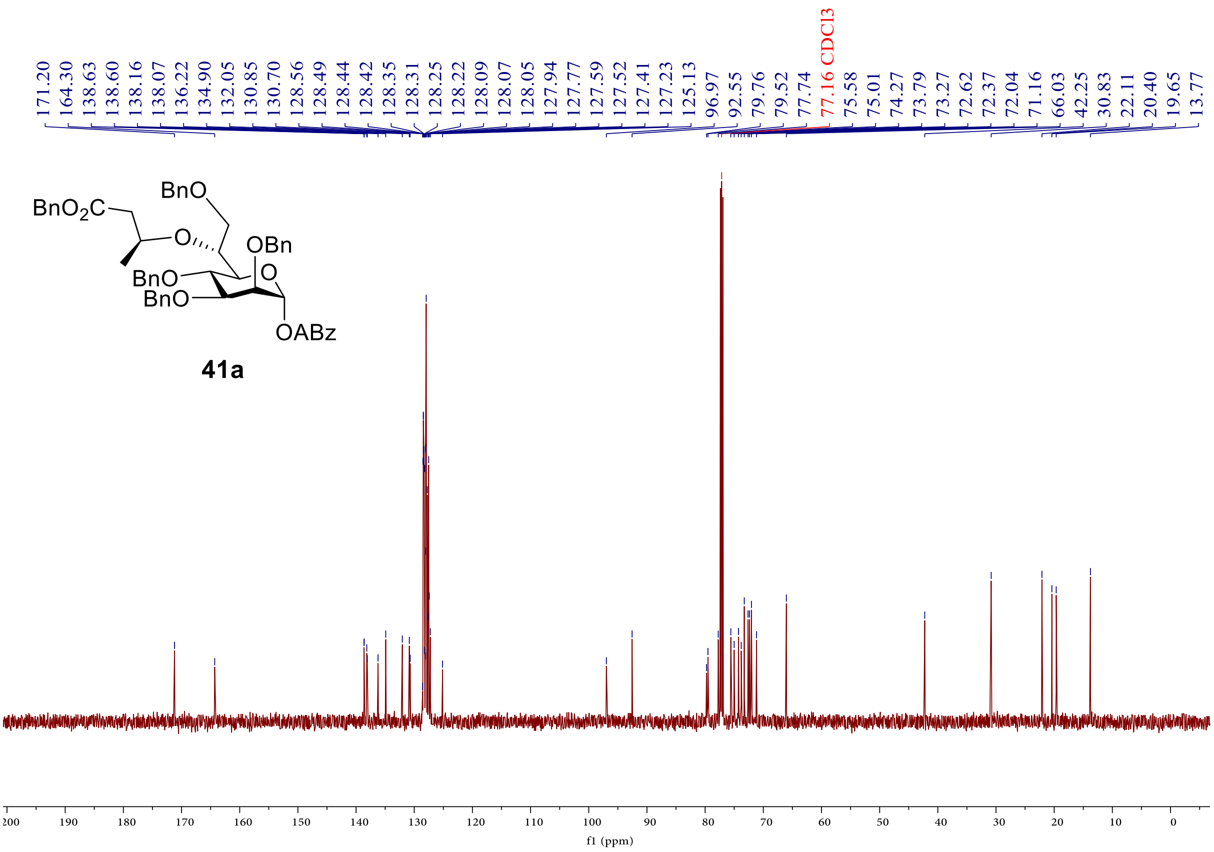
171.20
164.30
138.63
138.60
138.16
138.07
136.22
134.90
132.05
130.85
130.70
128.56
128.49
128.44
128.42
128.35
128.31
128.25
128.22
128.09
128.07
128.05
127.94
127.77
127.59
127.52
127.41
127.23
125.13
96.97
92.55
79.76
79.52
77.74
77.16 CDCl3
75.58
75.01
74.27
73.79
73.27
72.62
72.37
72.04
71.16
66.03
42.25
30.83
22.11
20.40
19.65
13.77
BnO
BnO_2C
O
OBn
O
BnO
BnO
OABz
41a
f1 (ppm)

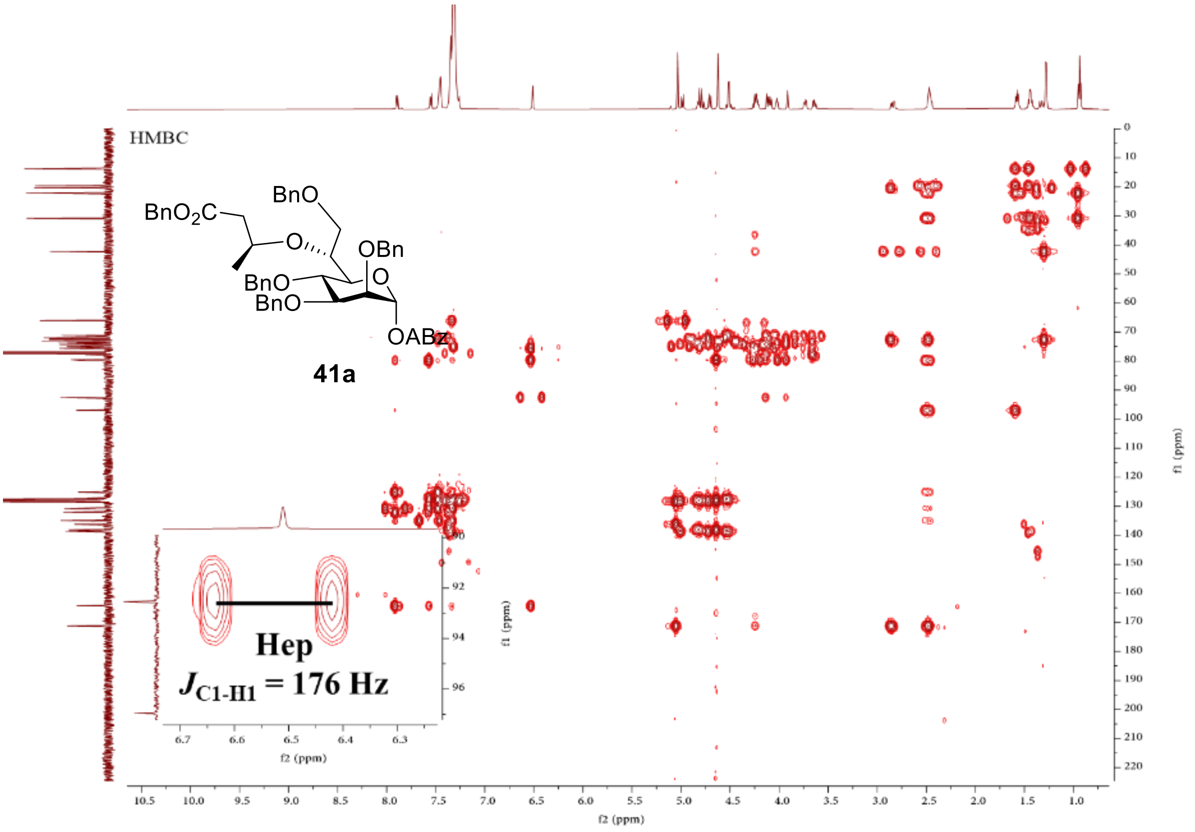
HMBC
BnO
BnO_2C
O
OBn
O
BnO
BnO
OABz
41a
Hep
J_{C1-H1} = 176 Hz
f2 (ppm)
f1 (ppm)

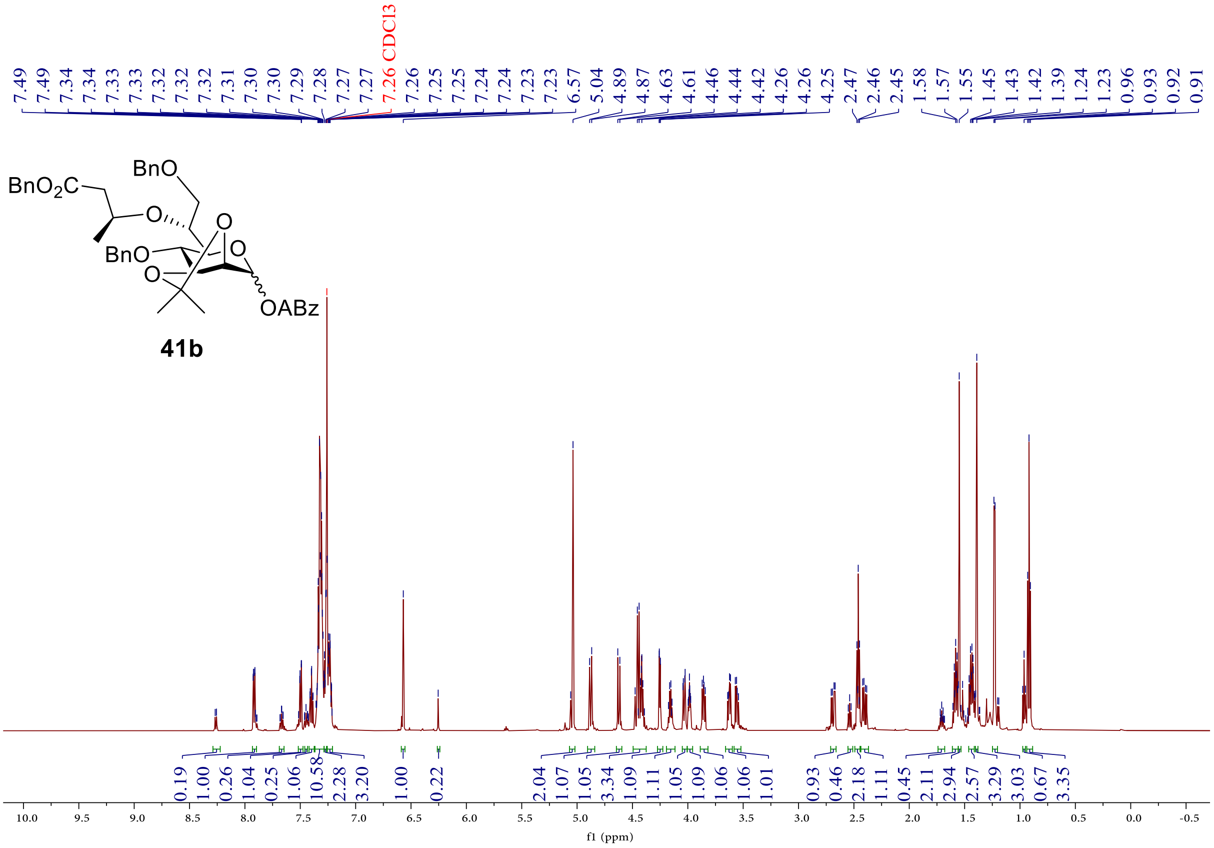

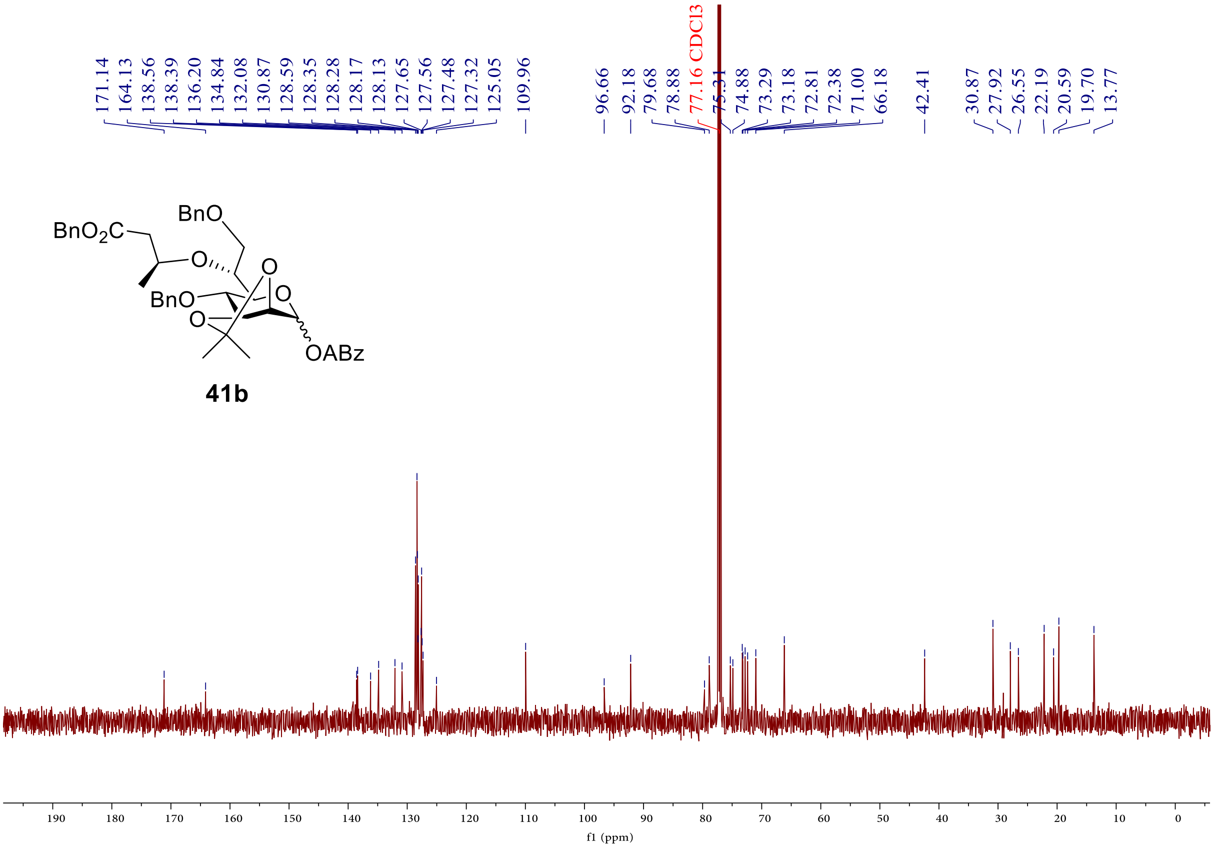

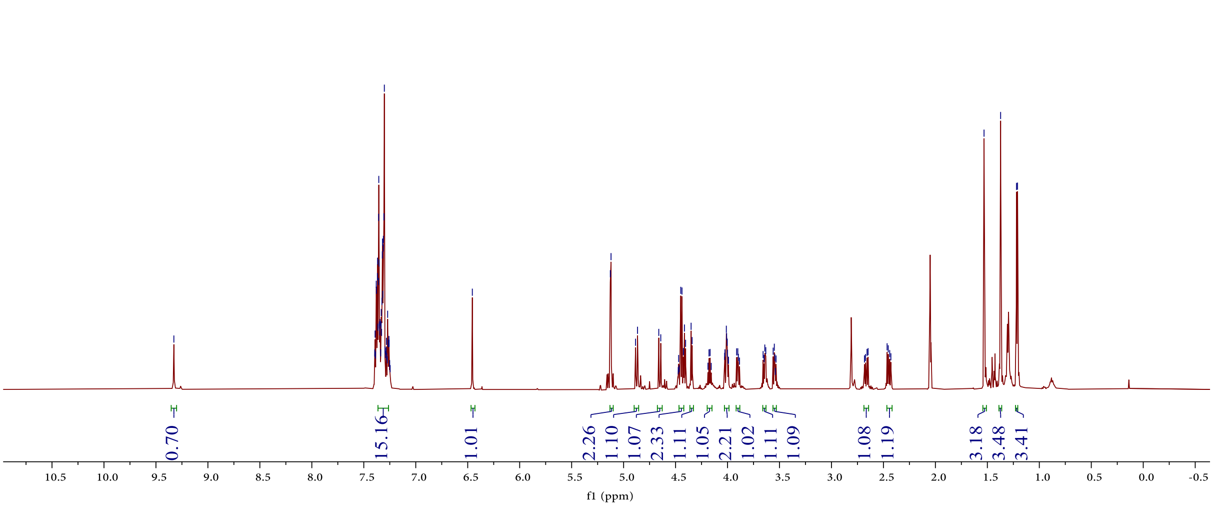
9.33 7.39 7.38 7.38 7.37 7.37 7.36 7.36 7.36 7.36 7.35 7.35 7.35 7.34 7.33 7.33 7.33 7.33 7.32 7.32 7.32 7.31 7.31 7.31 7.30 7.28 7.27 7.27 7.26 7.26 7.26 6.46 5.13 5.12 4.88 4.87 4.66 4.64 4.45 4.44 4.41 4.40 4.35 4.34 4.01 4.01 4.00 2.46 1.53 1.37 1.22 1.21
0.70 15.16 1.01 2.26 1.10 1.07 2.33 1.11 1.05 2.21 1.02 1.11 1.09 1.08 1.19 3.18 3.48 3.41
f1 (ppm)

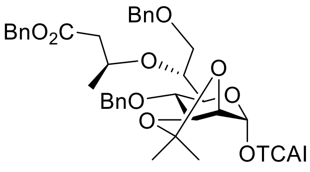
BnO
BnO2C
O
O
BnO
O
O
OTCAI

41d

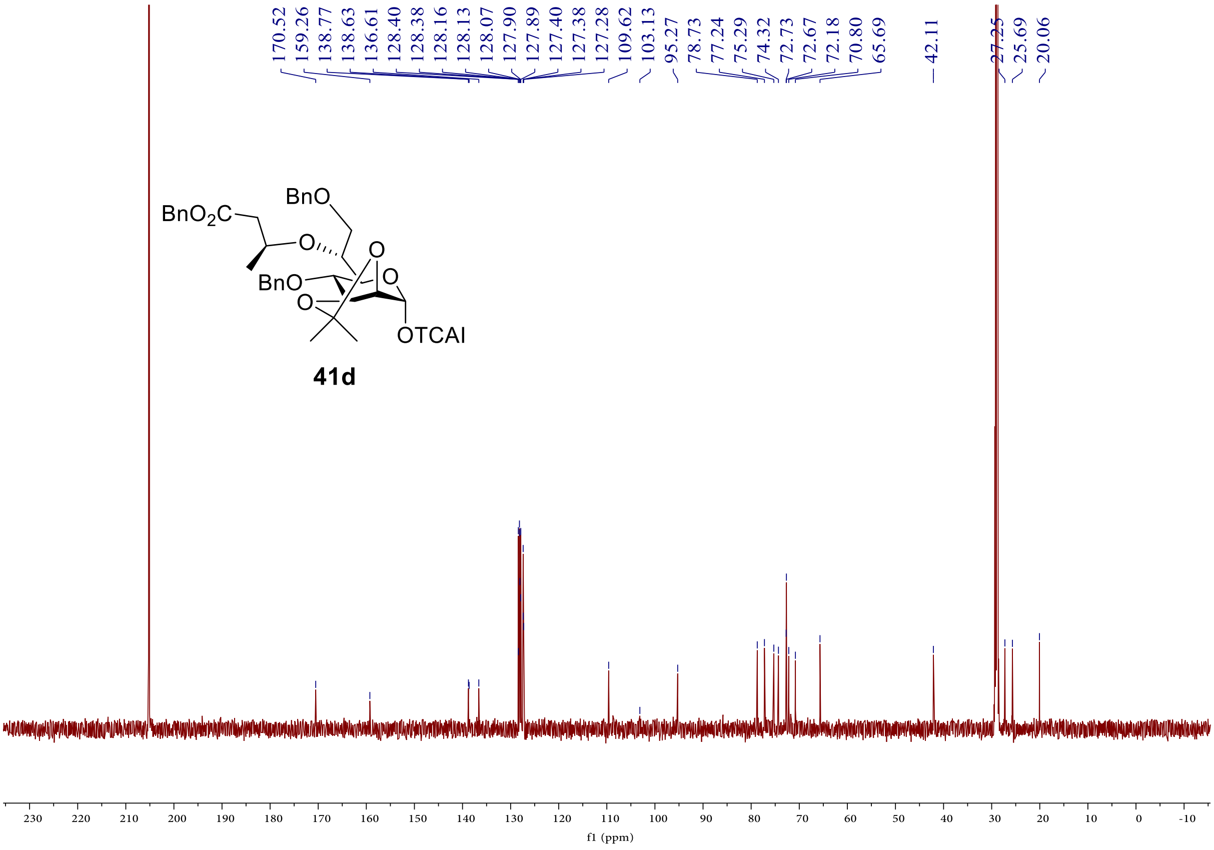
170.52 159.26 138.77 138.63 136.61 128.40 128.38 128.16 128.13 128.07 127.90 127.89 127.40 127.38 127.28 109.62 103.13 95.27 78.73 77.24 75.29 74.32 72.73 72.67 72.18 70.80 65.69 42.11 27.25 25.69 20.06
BnO
BnO2C
O
O
BnO
O
O
OTCAI
41d
f1 (ppm)

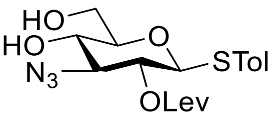

25

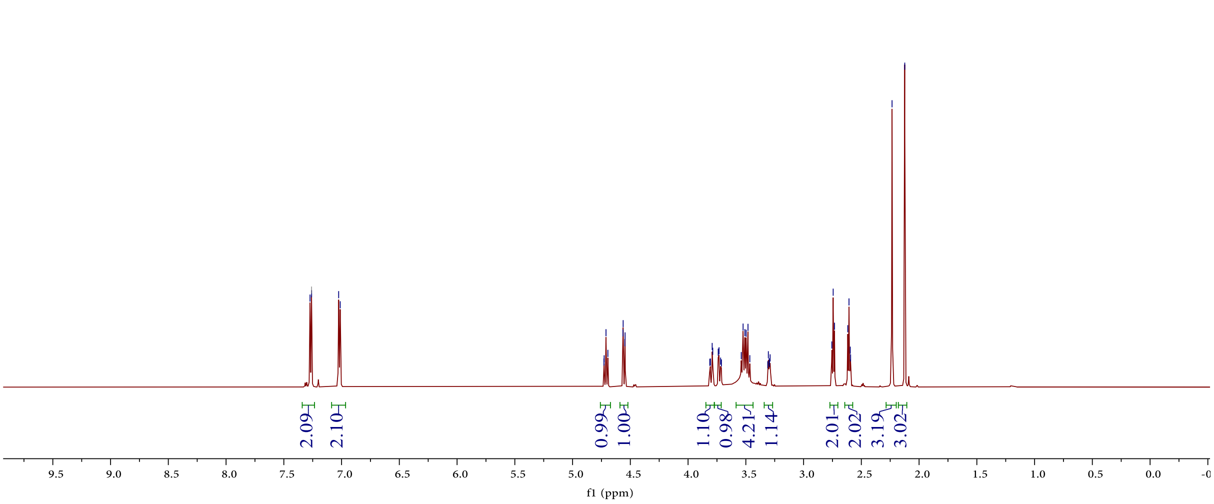

25

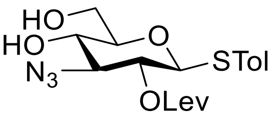

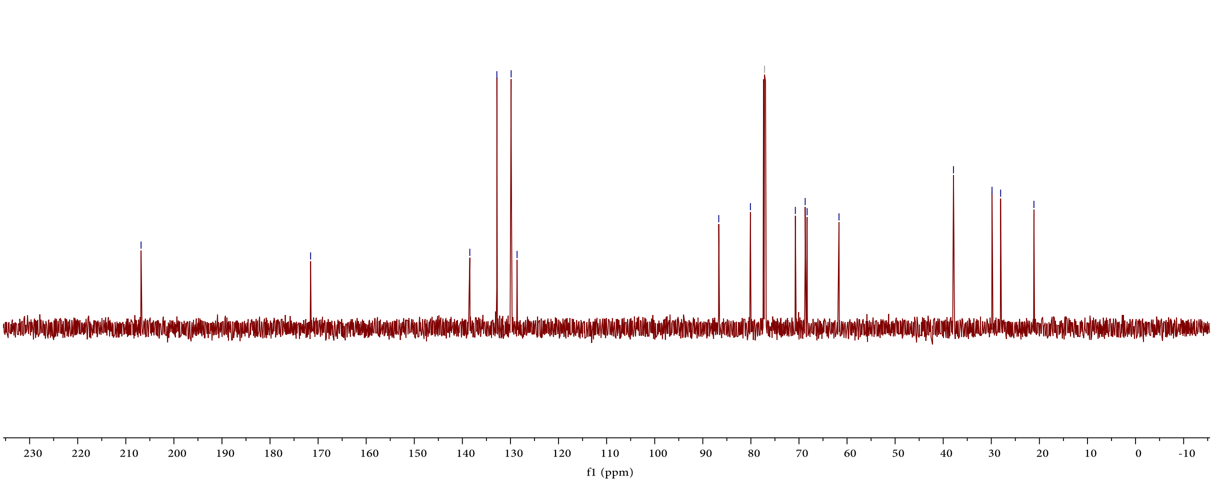

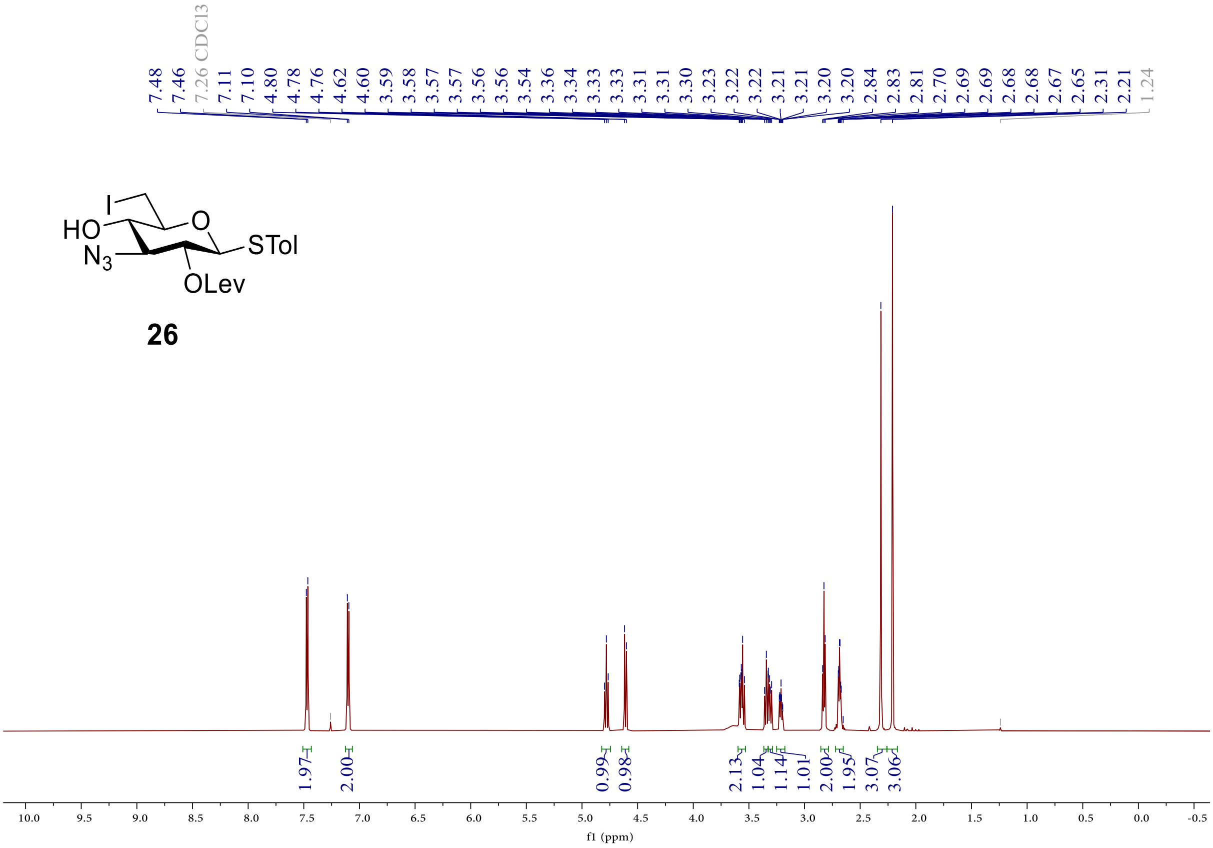
7.48
7.46
7.26 CDCl3
7.11
7.10
4.80
4.78
4.76
4.62
4.60
3.59
3.58
3.57
3.57
3.56
3.56
3.54
3.36
3.34
3.33
3.33
3.31
3.31
3.30
3.23
3.22
3.22
3.21
3.21
3.20
3.20
2.84
2.83
2.81
2.70
2.69
2.69
2.68
2.68
2.67
2.65
2.31
2.21
1.24
HO
I
O
N3
STol
OLev
26
1.97
2.00
0.99
0.98
2.13
1.04
1.14
1.01
2.00
1.95
3.07
3.06
f1 (ppm)

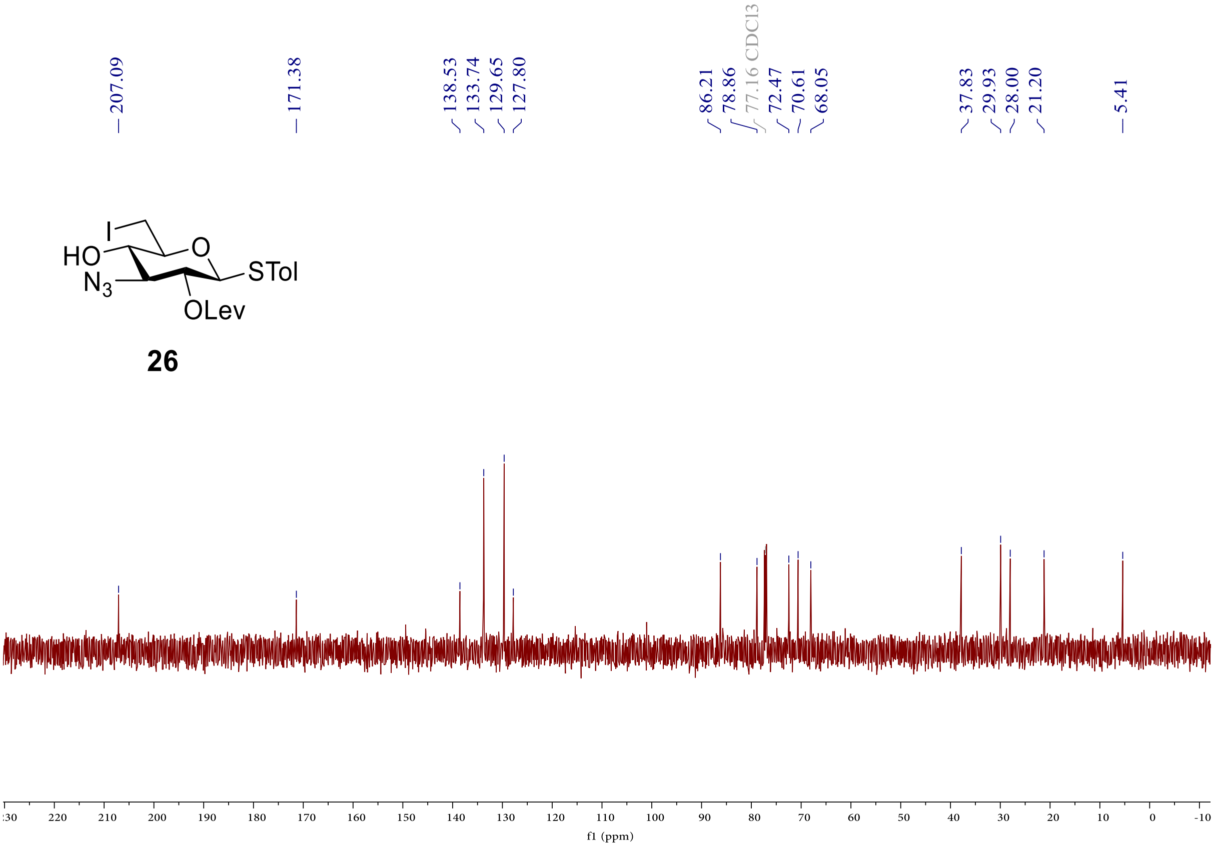
207.09
171.38
138.53
133.74
129.65
127.80
86.21
78.86
77.16 CDCl3
72.47
70.61
68.05
37.83
29.93
28.00
21.20
5.41
HO
I
O
N3
STol
OLev
26
f1 (ppm)

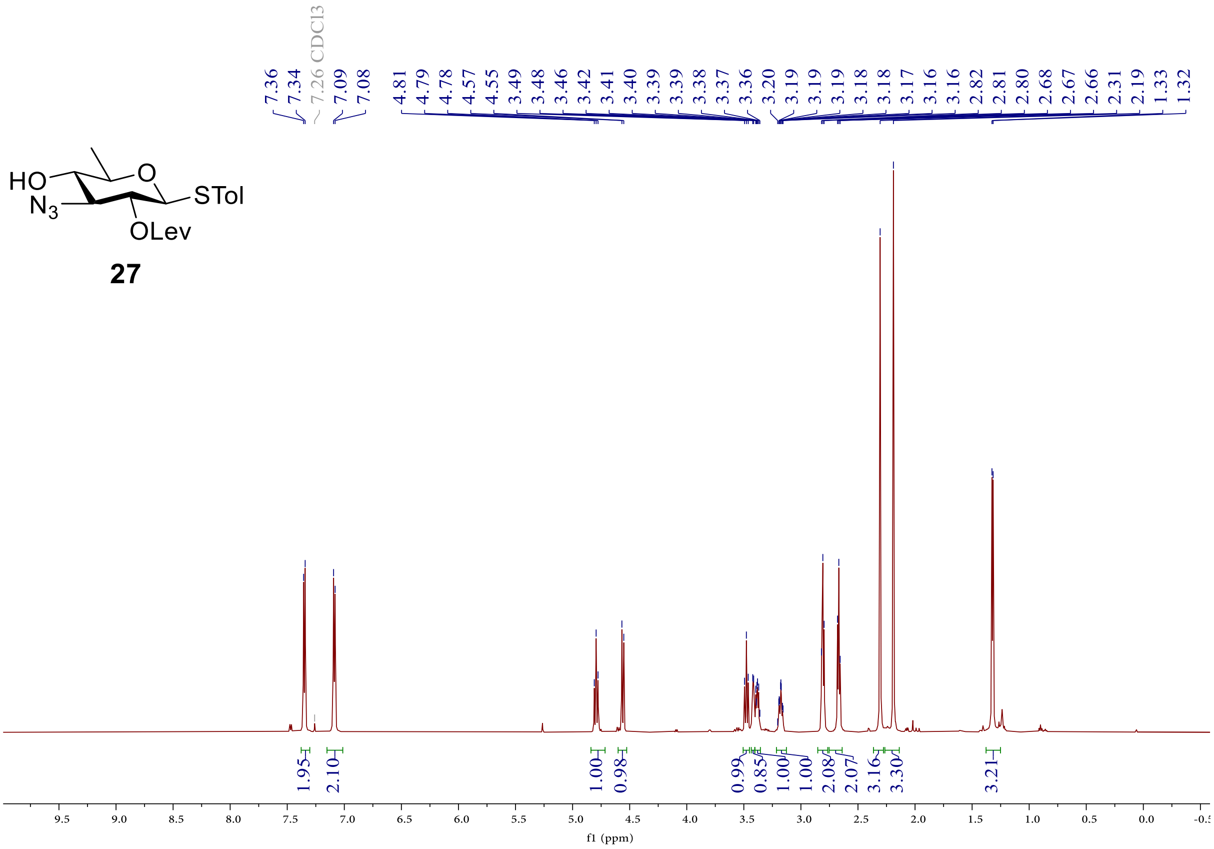
HO
N3
O
STol
OLev
27
7.36
7.34
7.26 CDCl3
7.09
7.08
4.81
4.79
4.78
4.57
4.55
3.49
3.48
3.46
3.42
3.41
3.40
3.39
3.39
3.38
3.37
3.36
3.20
3.19
3.19
3.19
3.18
3.18
3.17
3.16
3.16
2.82
2.81
2.80
2.68
2.67
2.66
2.31
2.19
1.33
1.32
1.95
2.10
1.00
0.98
0.99
0.85
1.00
1.00
2.08
2.07
3.16
3.30
3.21
f1 (ppm)

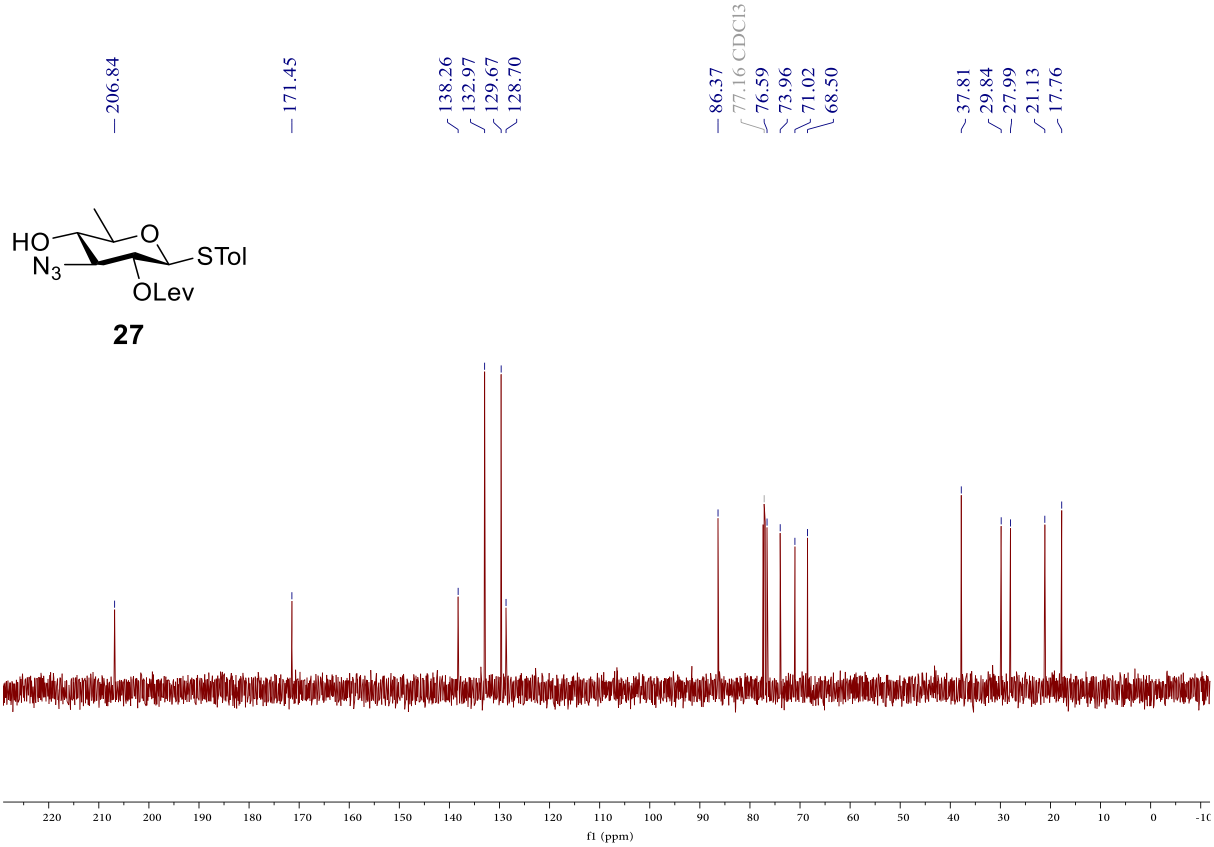
206.84
171.45
138.26
132.97
129.67
128.70
86.37
77.16 CDCl3
76.59
73.96
71.02
68.50
37.81
29.84
27.99
21.13
17.76
HO
N3
O
STol
OLev
27
f1 (ppm)

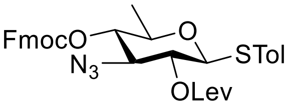
FmocO
N3
O
STol
OLev

15

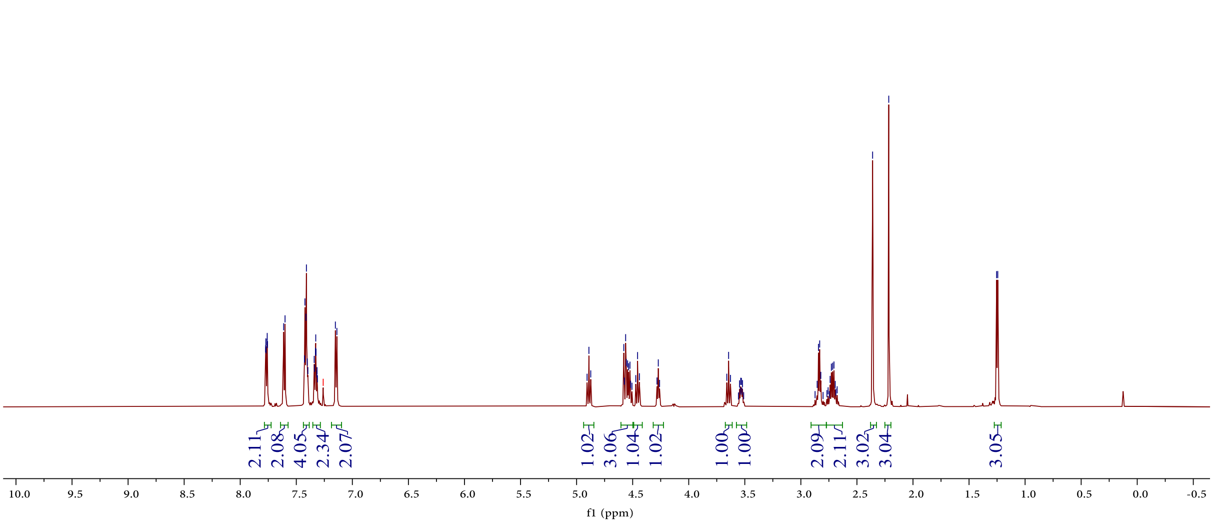
7.77 7.77 7.77 7.76 7.76 7.61 7.60 7.43 7.42 7.41 7.41 7.40 7.40 7.34 7.34 7.33 7.33 7.32 7.32 7.31 7.31 7.15 7.14 4.91 4.89 4.87 4.58 4.56 4.56 4.54 4.54 4.52 4.47 4.46 4.44 4.28 4.27 3.66 3.64 3.63 2.84 2.83 2.82 2.74 2.73 2.72 2.70 2.36 2.22 1.25 1.24
2.11 2.08 4.05 2.34 2.07 1.02 3.06 1.04 1.02 1.00 1.00 2.09 2.11 3.02 3.04 3.05
f1 (ppm)

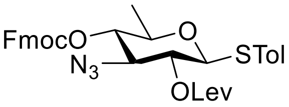
FmocO
N3
O
STol
OLev

15

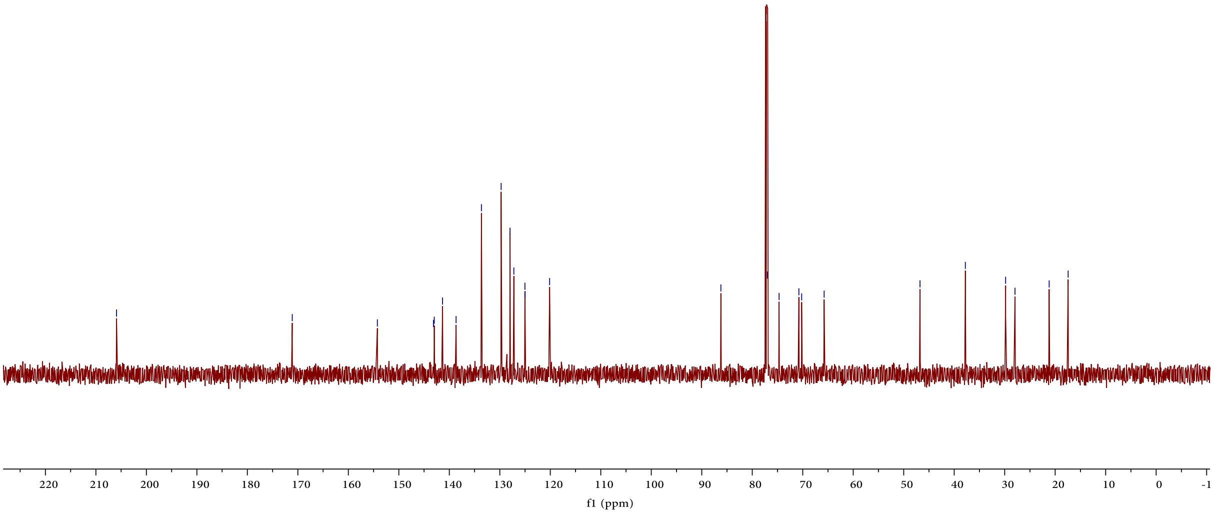
205.91 171.11 154.25 143.14 142.98 141.34 138.66 133.64 129.74 127.98 127.22 125.03 124.99 120.14 86.21 77.16 CDCl3 77.00 74.68 70.77 70.21 65.76 46.78 37.79 29.82 27.96 21.19 17.43
f1 (ppm)

G0-hth-AAT-6I

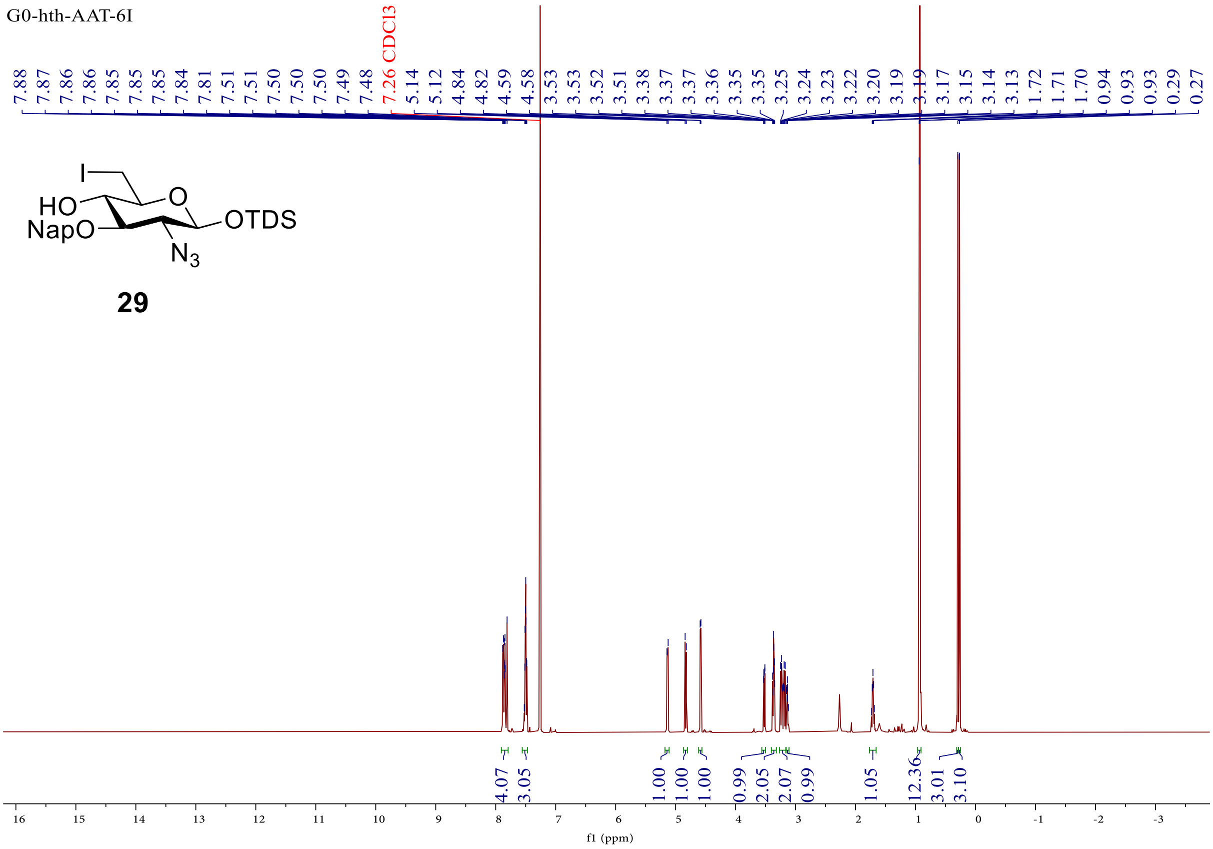

G0-hth-AAT-6I

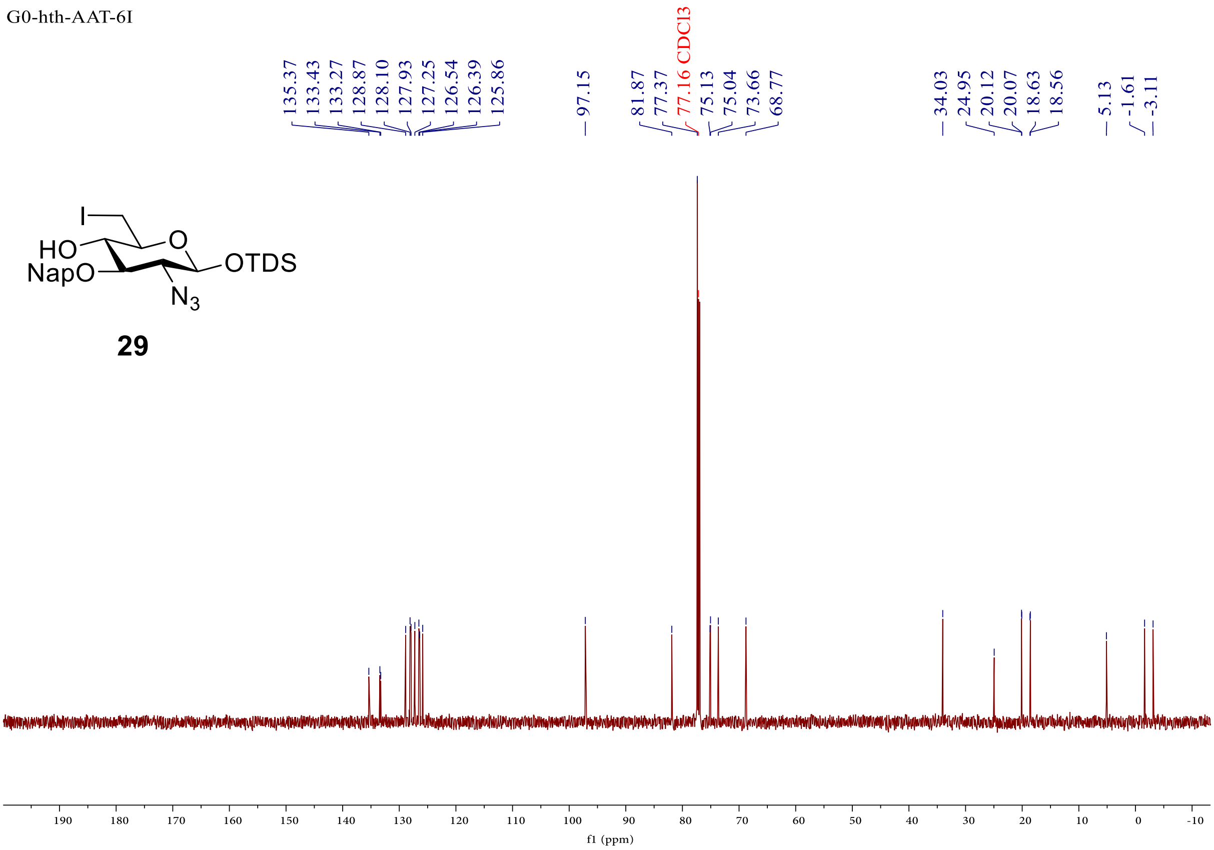

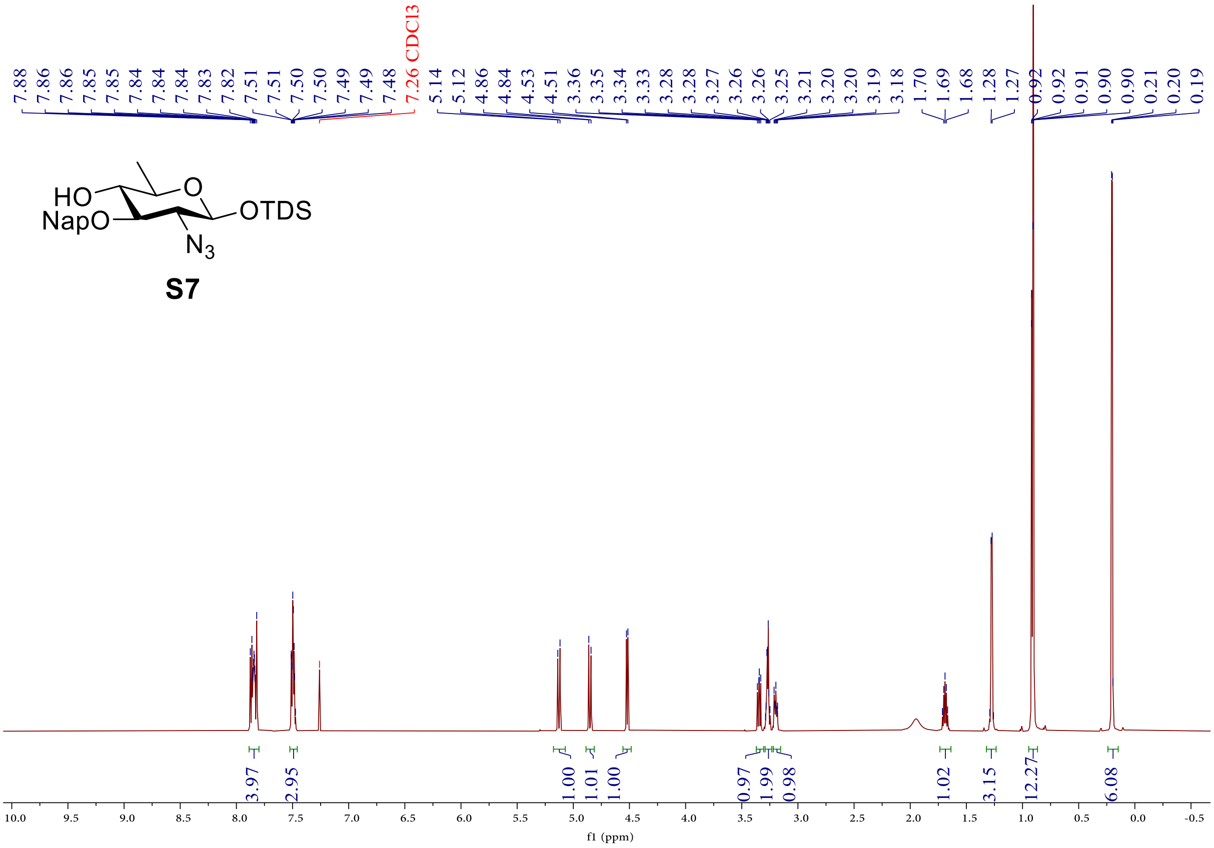
7.88 7.86 7.86 7.85 7.85 7.84 7.84 7.84 7.83 7.82 7.51 7.51 7.50 7.50 7.49 7.48 7.26 CDCl3 5.14 5.12 4.86 4.84 4.53 4.51 3.36 3.35 3.34 3.33 3.28 3.28 3.27 3.26 3.26 3.25 3.21 3.20 3.20 3.19 3.18 1.70 1.69 1.68 1.28 1.27 0.92 0.92 0.91 0.90 0.90 0.21 0.20 0.19
HO
NapO
O
OTDS
N3
S7
3.97
2.95
1.00
1.01
1.00
0.97
1.99
0.98
1.02
3.15
12.27
6.08
10.0 9.5 9.0 8.5 8.0 7.5 7.0 6.5 6.0 5.5 5.0 4.5 4.0 3.5 3.0 2.5 2.0 1.5 1.0 0.5 0.0 -0.5
f1 (ppm)

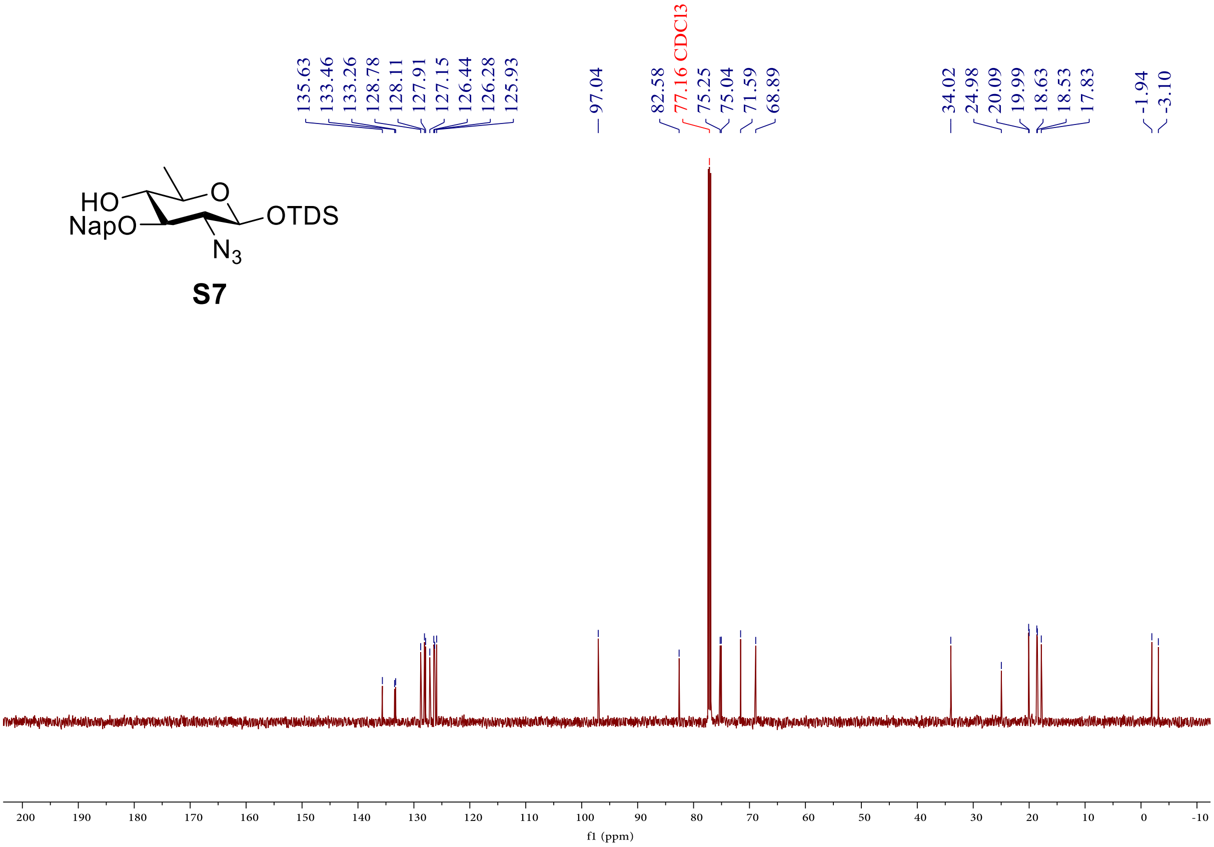
135.63 133.46 133.26 128.78 128.11 127.91 127.15 126.44 126.28 125.93
97.04
82.58 77.16 CDCl3 75.25 75.04 71.59 68.89
34.02 24.98 20.09 19.99 18.63 18.53 17.83
-1.94 -3.10
HO
NapO
O
OTDS
N3
S7
200 190 180 170 160 150 140 130 120 110 100 90 80 70 60 50 40 30 20 10 0 -10
f1 (ppm)

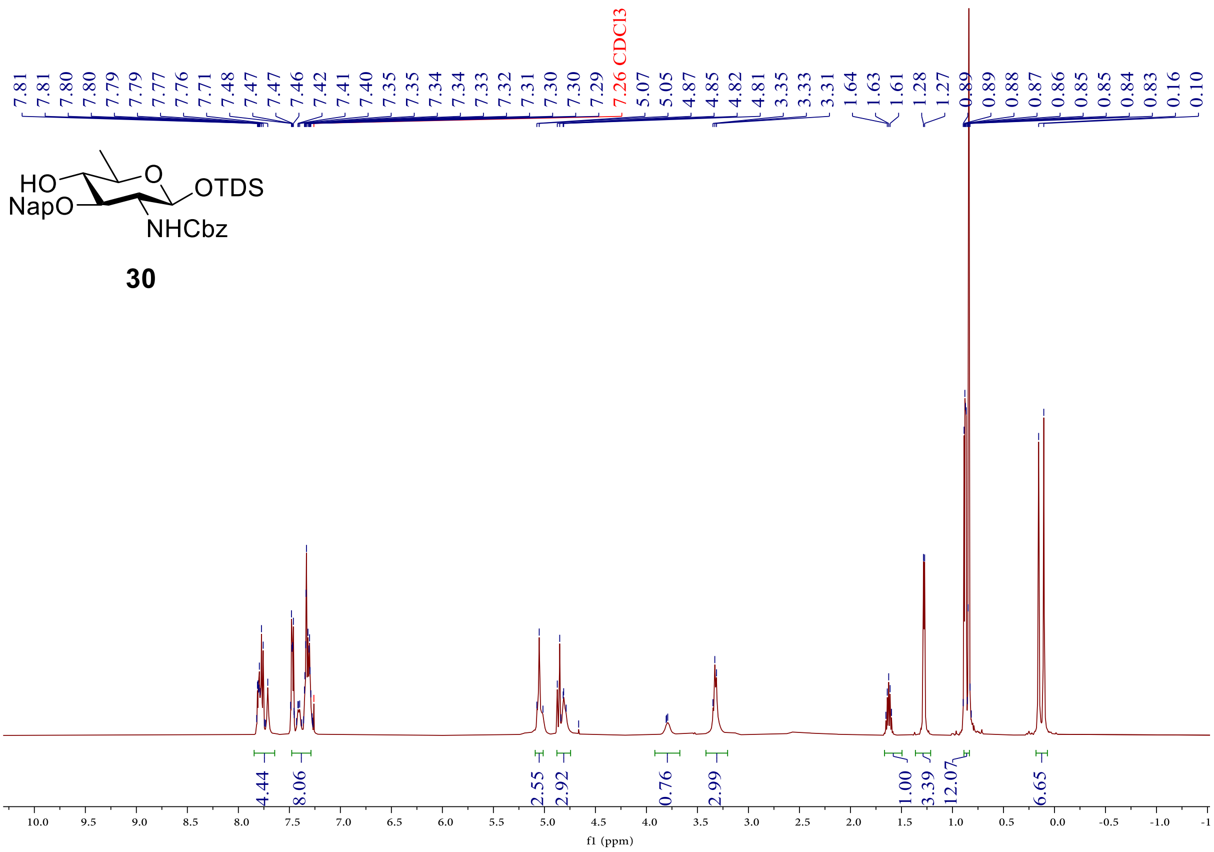
7.81 7.81 7.80 7.80 7.79 7.79 7.77 7.76 7.71 7.48 7.47 7.47 7.46 7.42 7.41 7.40 7.35 7.35 7.34 7.34 7.33 7.32 7.31 7.30 7.30 7.29 7.26 CDCl3 5.07 5.05 4.87 4.85 4.82 4.81 3.35 3.33 3.31 1.64 1.63 1.61 1.28 1.27 0.89 0.89 0.88 0.87 0.86 0.85 0.85 0.84 0.83 0.16 0.10
HO
NapO
OTDS
NHCbz
30
4.44 8.06 2.55 2.92 0.76 2.99 1.00 3.39 12.07 6.65
f1 (ppm)

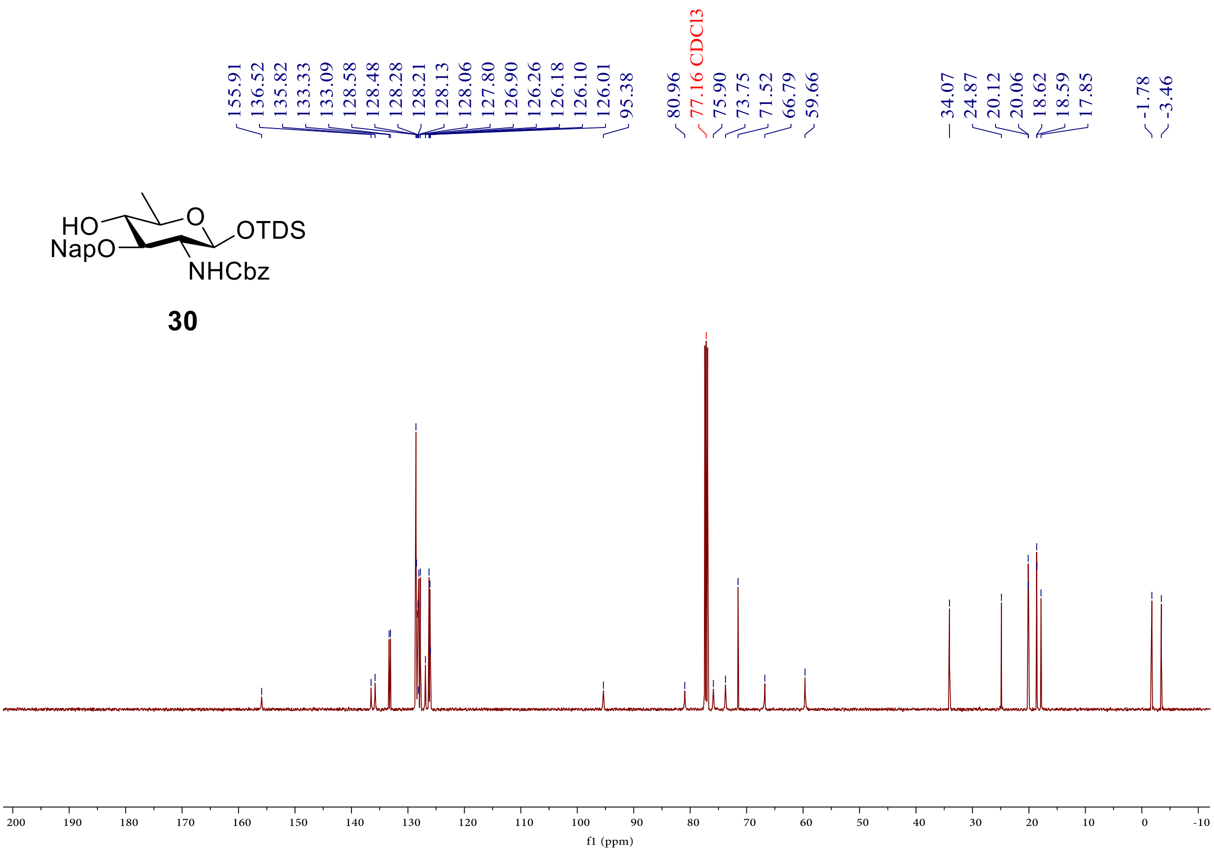
155.91 136.52 135.82 133.33 133.09 128.58 128.48 128.28 128.21 128.13 128.06 127.80 126.90 126.26 126.18 126.10 126.01 95.38 80.96 77.16 CDCl3 75.90 73.75 71.52 66.79 59.66 34.07 24.87 20.12 20.06 18.62 18.59 17.85 -1.78 -3.46
HO
NapO
OTDS
NHCbz
30
f1 (ppm)

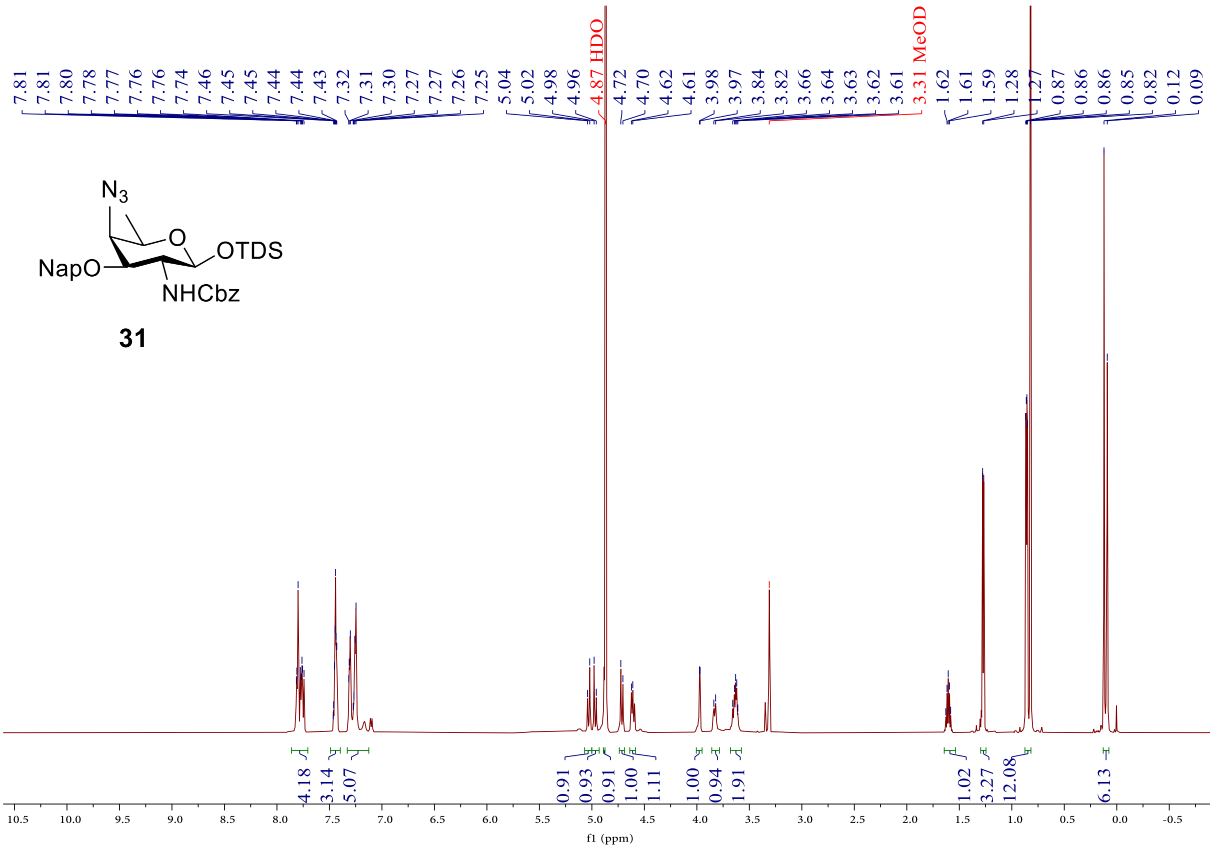
7.81 7.81 7.80 7.78 7.77 7.76 7.76 7.74 7.46 7.45 7.45 7.44 7.44 7.43 7.32 7.31 7.30 7.27 7.27 7.26 7.25 5.04 5.02 4.98 4.96 4.87 HDO 4.72 4.70 4.62 4.61 3.98 3.97 3.84 3.82 3.66 3.64 3.63 3.62 3.61 3.31 MeOD 1.62 1.61 1.59 1.28 1.27 0.87 0.86 0.86 0.85 0.82 0.12 0.09
N3
NapO
O
OTDS
NHCbz
31
4.18 3.14 5.07 0.91 0.93 0.91 1.00 1.11 1.00 0.94 1.91 1.02 3.27 12.08 6.13
f1 (ppm)

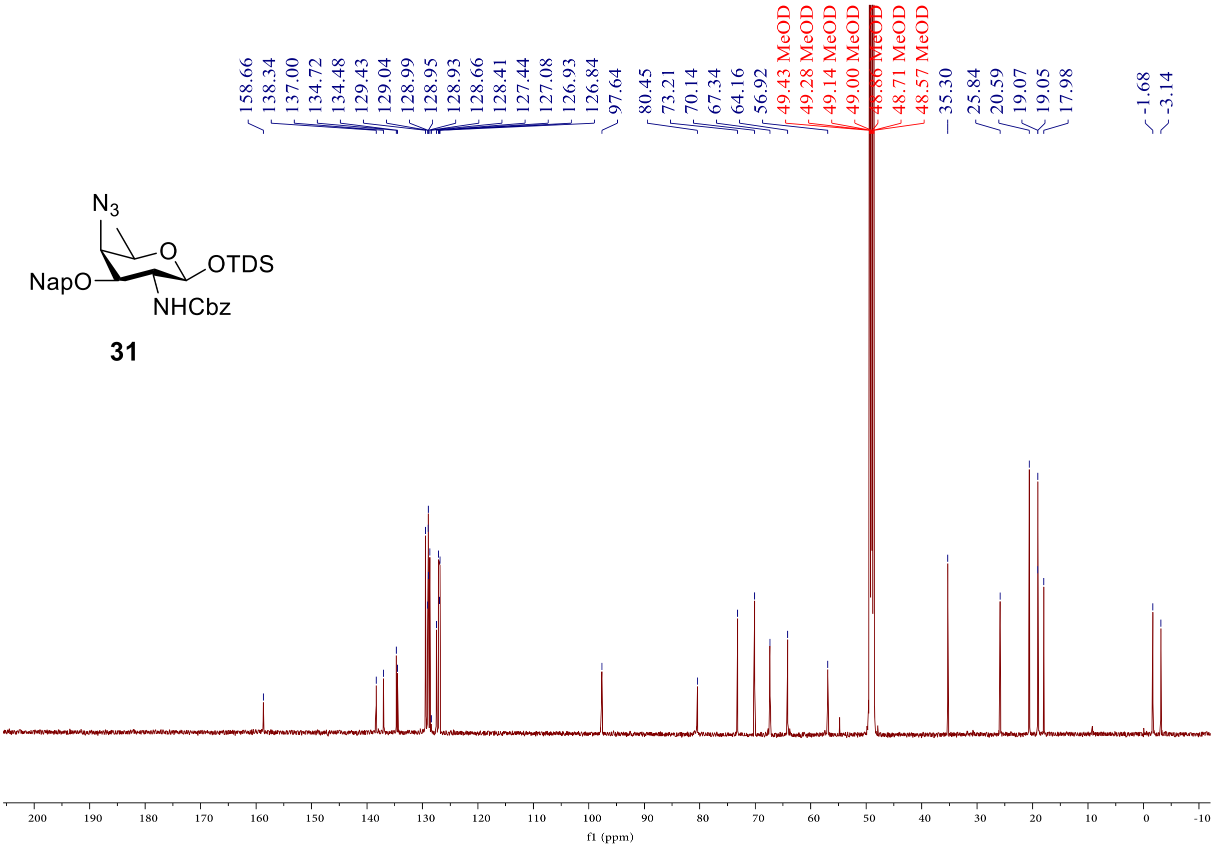
158.66 138.34 137.00 134.72 134.48 129.43 129.04 128.99 128.95 128.93 128.66 128.41 127.44 127.08 126.93 126.84 97.64 80.45 73.21 70.14 67.34 64.16 56.92 49.43 MeOD 49.28 MeOD 49.14 MeOD 49.00 MeOD 48.86 MeOD 48.71 MeOD 48.57 MeOD 35.30 25.84 20.59 19.07 19.05 17.98 -1.68 -3.14
N3
NapO
O
OTDS
NHCbz
31
f1 (ppm)

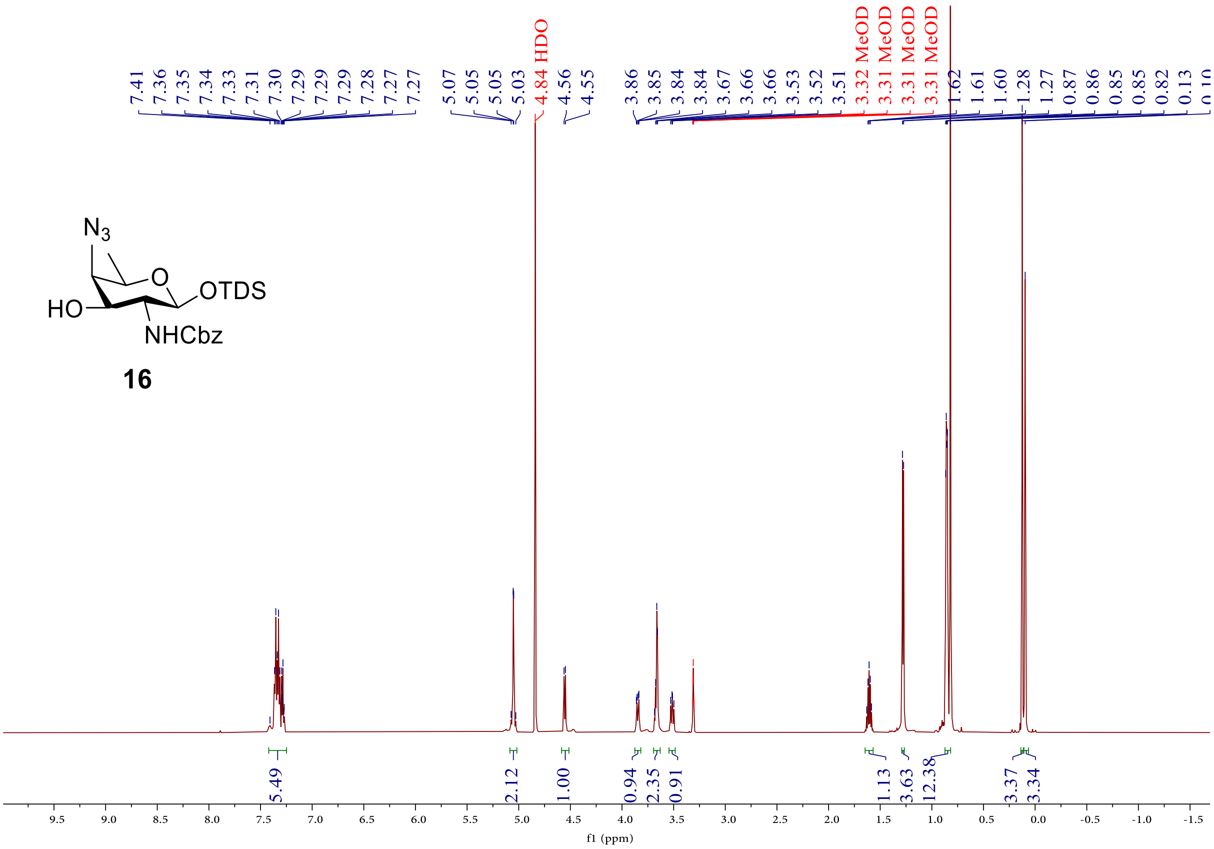
7.41 7.36 7.35 7.34 7.33 7.31 7.30 7.29 7.29 7.29 7.28 7.27 7.27
5.07 5.05 5.05 5.03
4.84 HDO
4.56 4.55
3.86 3.85 3.84 3.84 3.67 3.66 3.66 3.53 3.52 3.51
3.32 MeOD 3.31 MeOD 3.31 MeOD 3.31 MeOD
1.62 1.61 1.60 1.28 1.27 0.87 0.86 0.85 0.85 0.82 0.13
N3
O
OTDS
HO
NHCbz
16
5.49 2.12 1.00 0.94 2.35 0.91 1.13 3.63 12.38 3.37 3.34
9.5 9.0 8.5 8.0 7.5 7.0 6.5 6.0 5.5 5.0 4.5 4.0 3.5 3.0 2.5 2.0 1.5 1.0 0.5 0.0 -0.5 -1.0 -1.5
f1 (ppm)

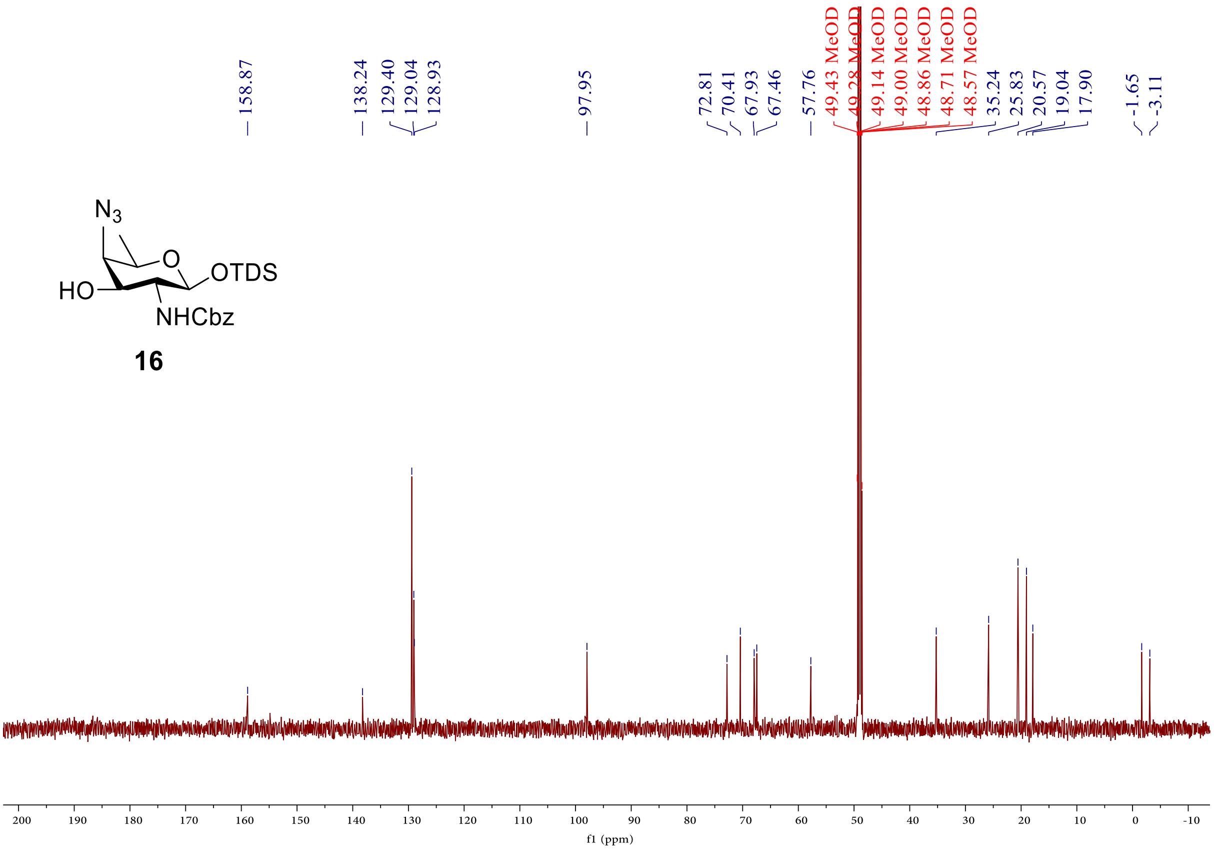
158.87
138.24 129.40 129.04 128.93
97.95
72.81 70.41 67.93 67.46
57.76
49.43 MeOD 49.28 MeOD 49.14 MeOD 49.00 MeOD 48.86 MeOD 48.71 MeOD 48.57 MeOD
35.24 25.83 20.57 19.04 17.90
-1.65 -3.11
N3
O
OTDS
HO
NHCbz
16
200 190 180 170 160 150 140 130 120 110 100 90 80 70 60 50 40 30 20 10 0 -10
f1 (ppm)

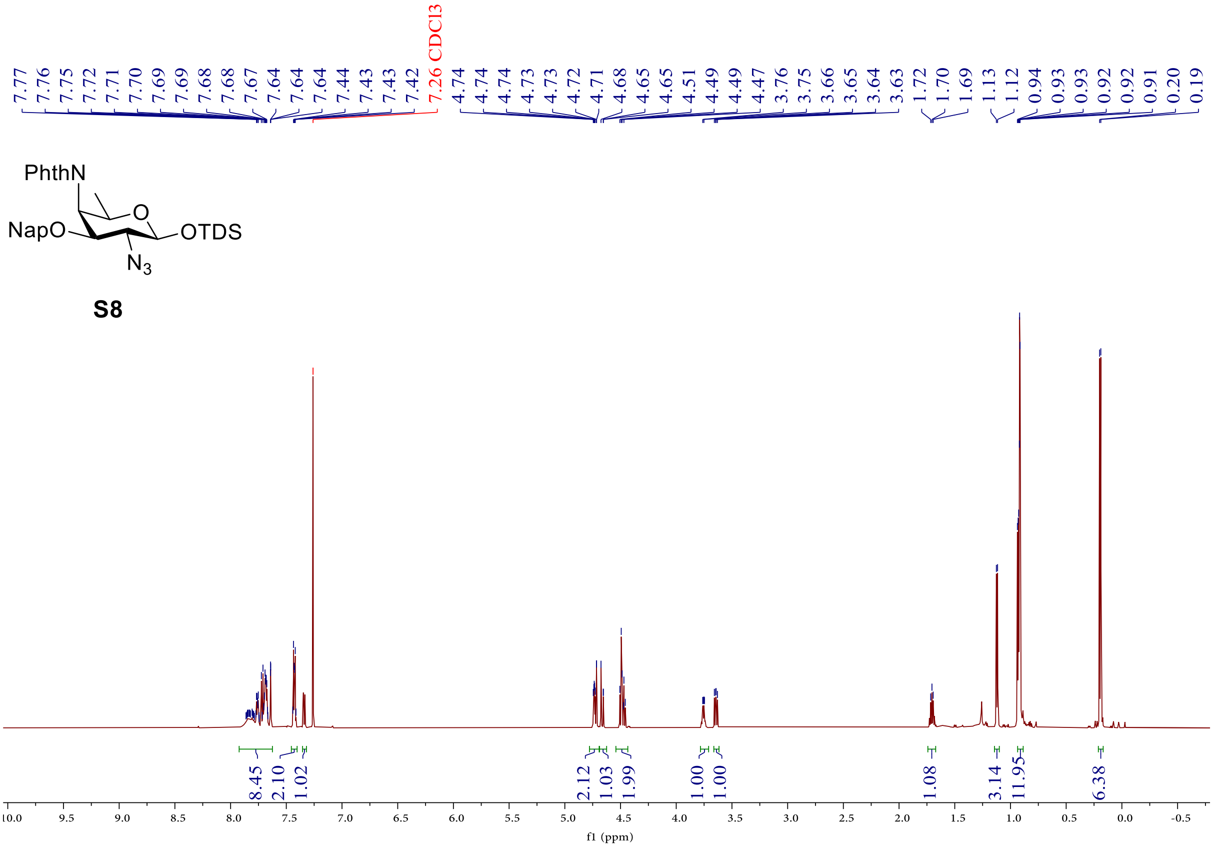
7.77
7.76
7.75
7.72
7.71
7.70
7.69
7.69
7.68
7.68
7.67
7.64
7.64
7.64
7.44
7.43
7.43
7.42
7.26 CDCl3
4.74
4.74
4.74
4.73
4.73
4.72
4.71
4.68
4.65
4.65
4.51
4.49
4.49
4.47
3.76
3.75
3.66
3.65
3.64
3.63
1.72
1.70
1.69
1.13
1.12
0.94
0.93
0.93
0.92
0.92
0.91
0.20
0.19
PhthN
NapO
O
OTDS
N3
S8
8.45
2.10
1.02
2.12
1.03
1.99
1.00
1.00
1.08
3.14
11.95
6.38
f1 (ppm)

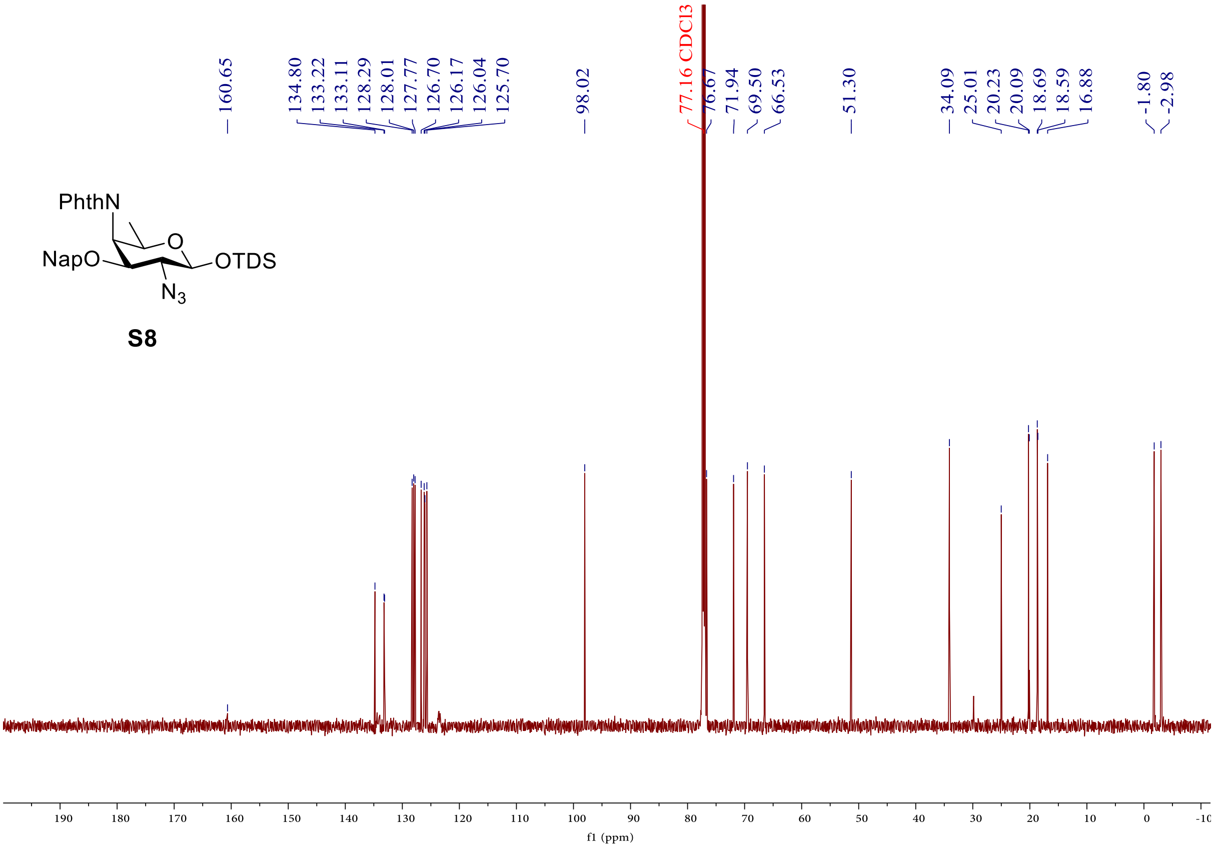
160.65
134.80
133.22
133.11
128.29
128.01
127.77
126.70
126.17
126.04
125.70
98.02
77.16 CDCl3
76.67
71.94
69.50
66.53
51.30
34.09
25.01
20.23
20.09
18.69
18.59
16.88
-1.80
-2.98
PhthN
NapO
O
OTDS
N3
S8
f1 (ppm)

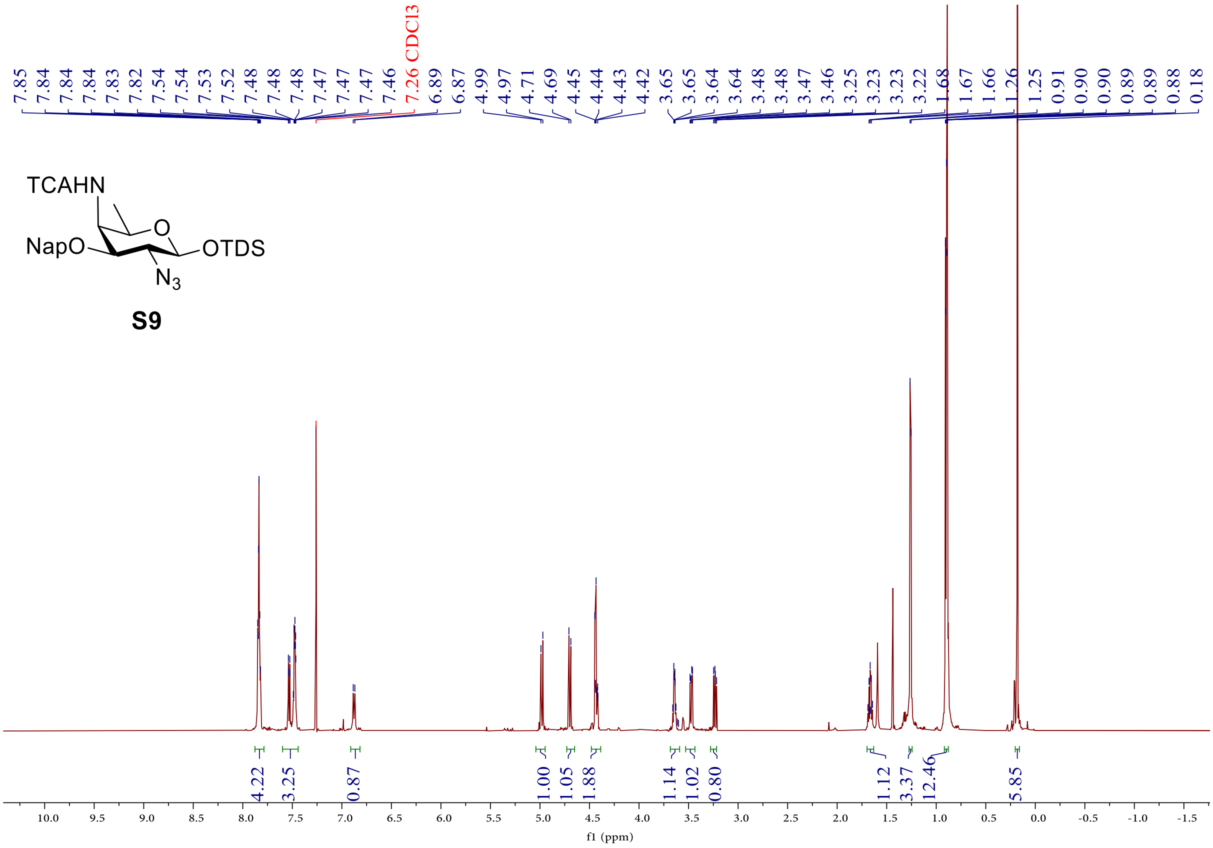
7.85 7.84 7.84 7.84 7.83 7.82 7.54 7.54 7.53 7.52 7.48 7.48 7.47 7.47 7.47 7.46 7.26 CDCl3 6.89 6.87 4.99 4.97 4.71 4.69 4.45 4.44 4.43 4.42 3.65 3.65 3.64 3.48 3.48 3.47 3.46 3.25 3.23 3.23 3.22 1.68 1.67 1.66 1.26 1.25 0.91 0.90 0.90 0.89 0.89 0.88 0.18
TCAHN
O
NapO
OTDS
N_3
S9
4.22 3.25 0.87 1.00 1.05 1.88 1.14 1.02 0.80 1.12 3.37 12.46 5.85
f1 (ppm)

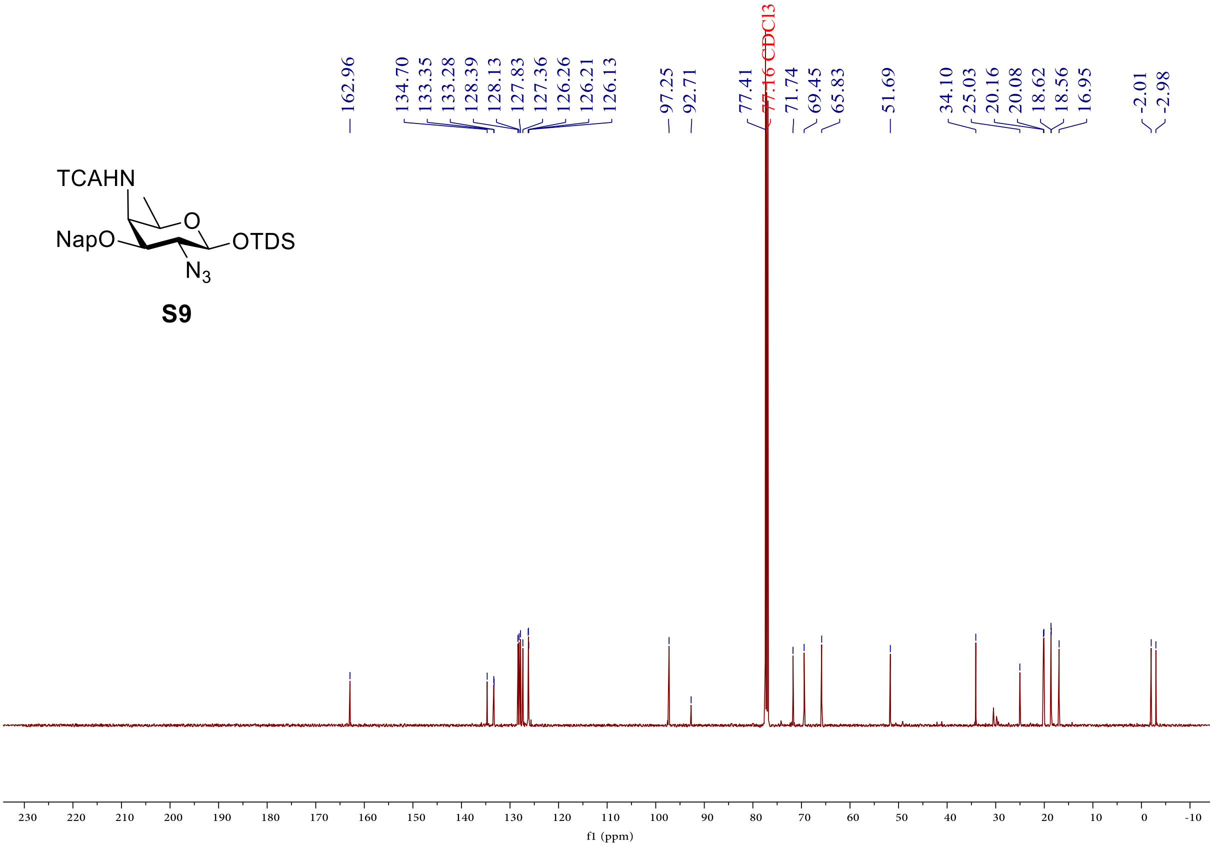
162.96 134.70 133.35 133.28 128.39 128.13 127.83 127.36 126.26 126.21 126.13 97.25 92.71 77.41 77.16 CDCl3 71.74 69.45 65.83 51.69 34.10 25.03 20.16 20.08 18.62 18.56 16.95 -2.01 -2.98
TCAHN
O
NapO
OTDS
N_3
S9
f1 (ppm)

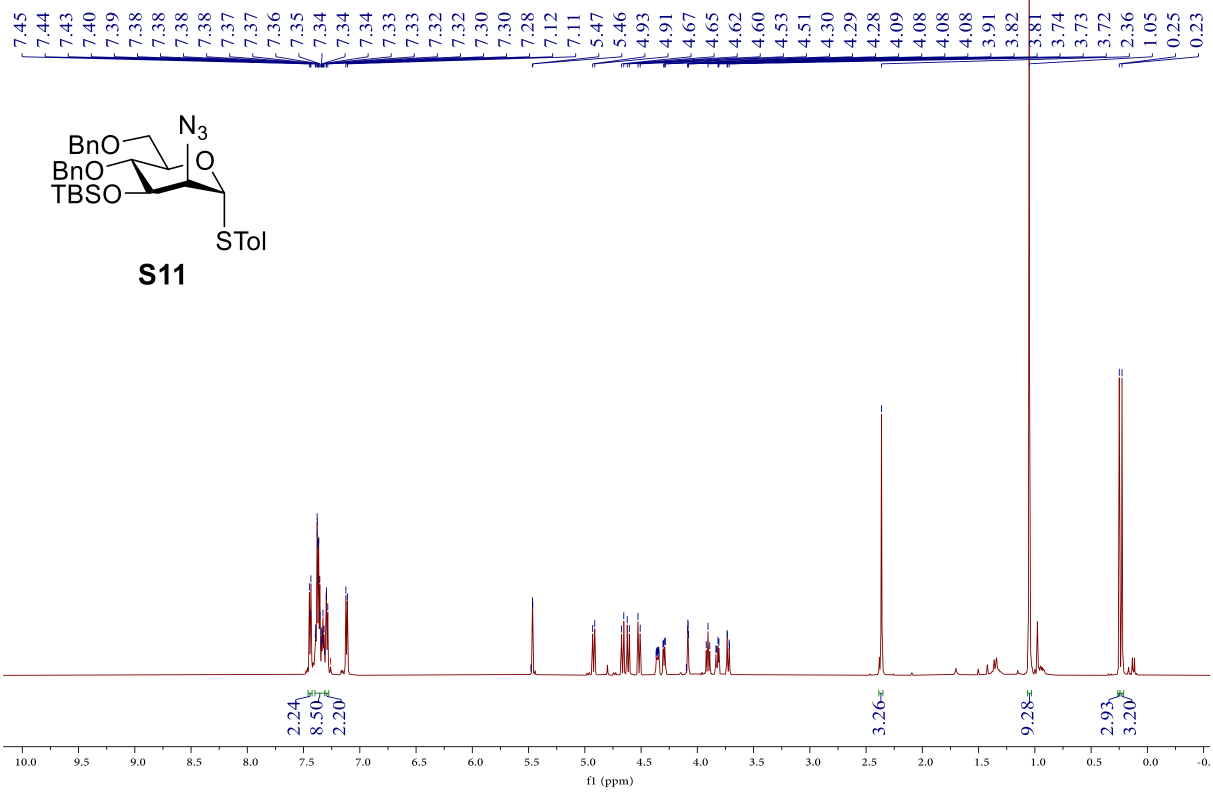
N3
BnO
BnO
TBSO
O
STol
S11
f1 (ppm)

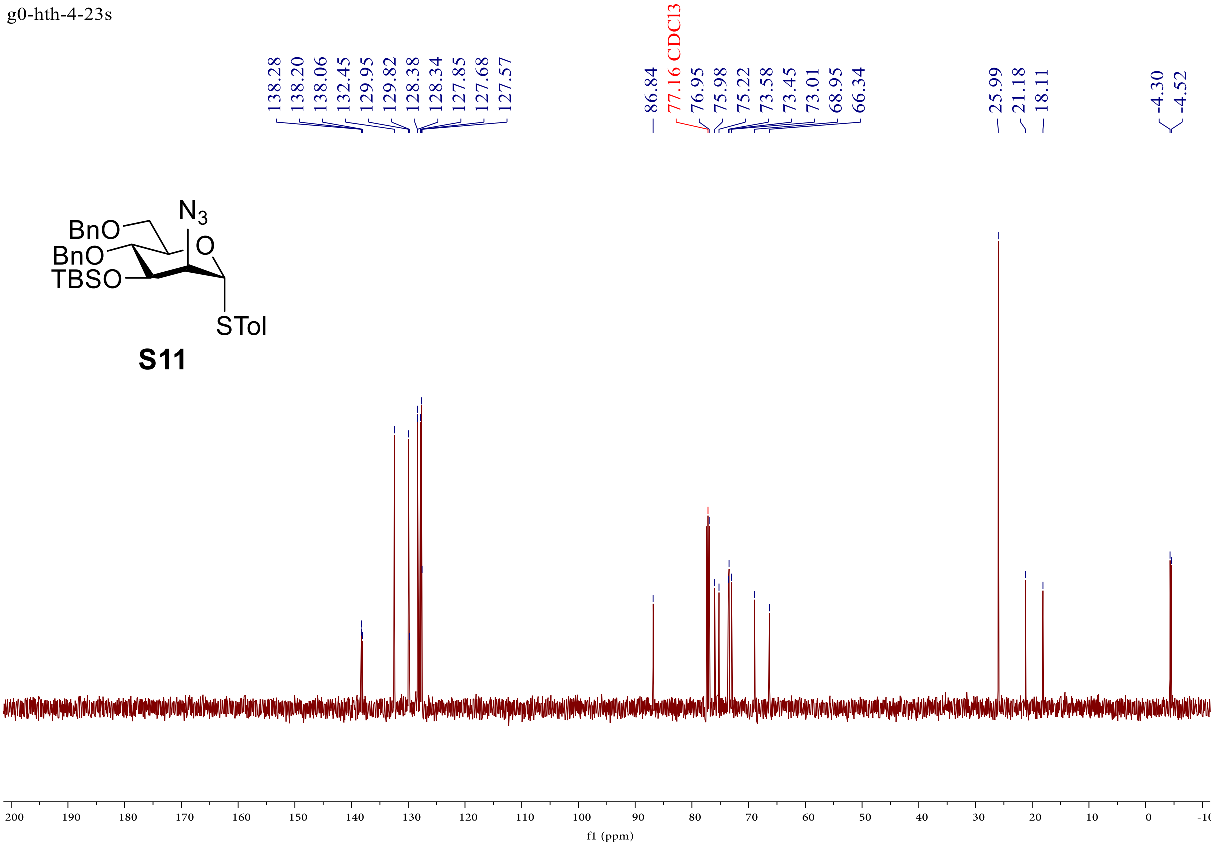
g0-hth-4-23s
77.16 CDCl3
N3
BnO
BnO
TBSO
O
STol
S11
f1 (ppm)

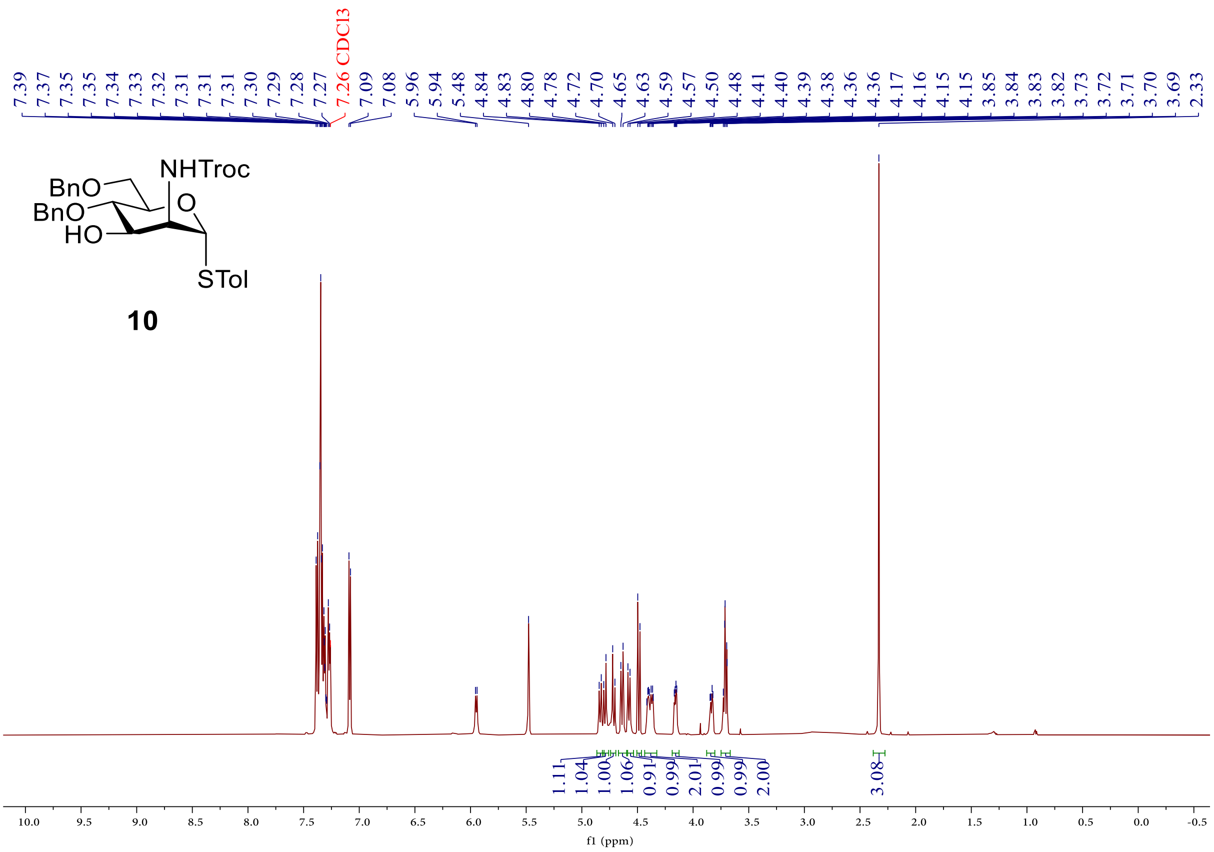
7.39 7.37 7.35 7.35 7.34 7.33 7.32 7.31 7.31 7.31 7.30 7.29 7.28 7.27
7.26 CDCl3
7.09 7.08 5.96 5.94 5.48 4.84 4.83 4.80 4.78 4.72 4.70 4.65 4.63 4.59 4.57 4.50 4.48 4.41 4.40 4.39 4.38 4.36 4.36 4.17 4.16 4.15 4.15 3.85 3.84 3.83 3.82 3.73 3.72 3.71 3.70 3.69 2.33
BnO
BnO
HO
NHTroc
O
STol
10
1.11 1.04 1.00 1.06 0.91 0.99 2.01 0.99 0.99 2.00 3.08
f1 (ppm)

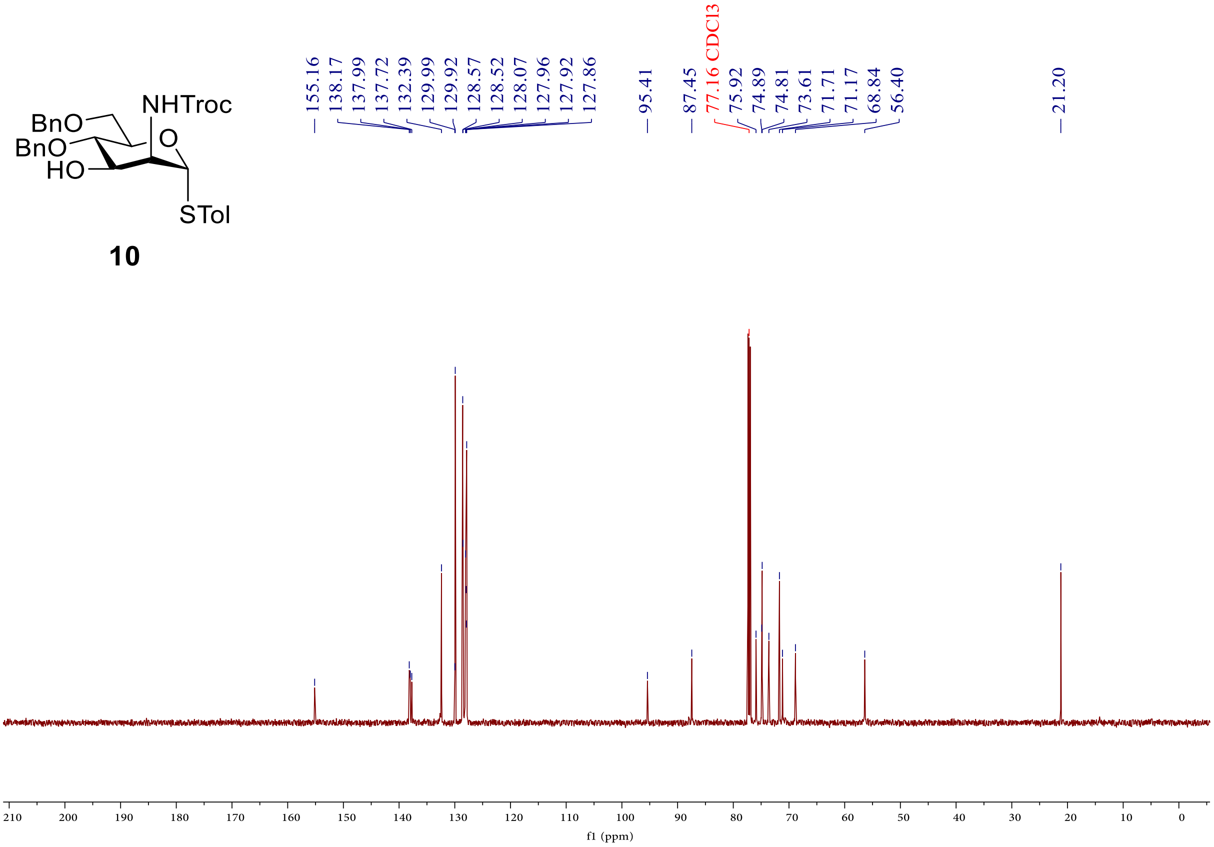
155.16 138.17 137.99 137.72 132.39 129.99 129.92 128.57 128.52 128.07 127.96 127.92 127.86
95.41 87.45
77.16 CDCl3
75.92 74.89 74.81 73.61 71.71 71.17 68.84 56.40 21.20
BnO
BnO
HO
NHTroc
O
STol
10
f1 (ppm)

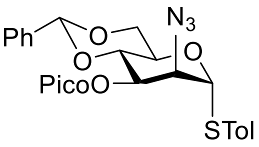

34a

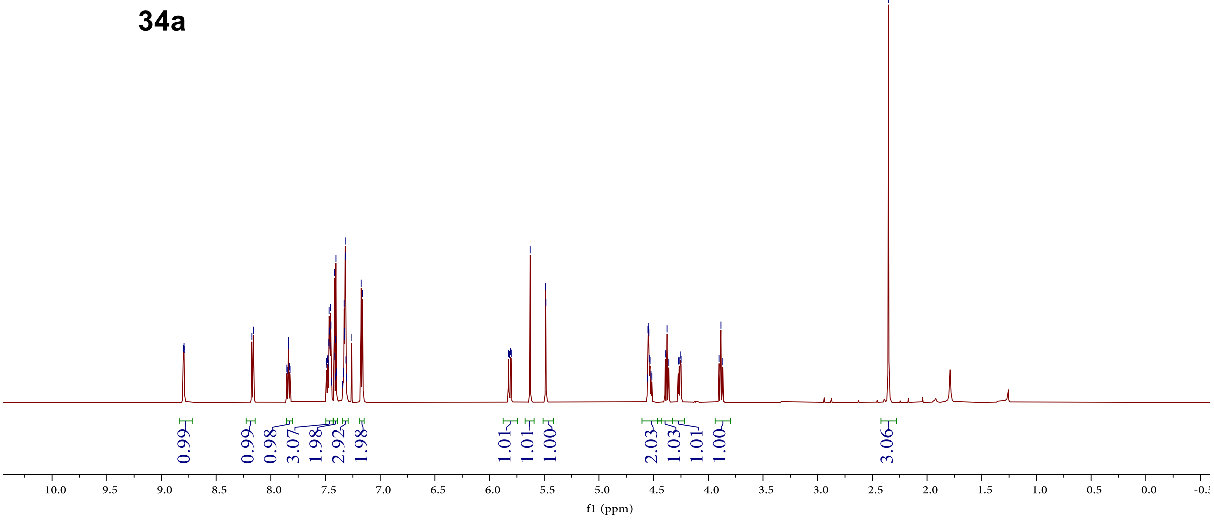

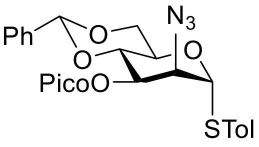

34a

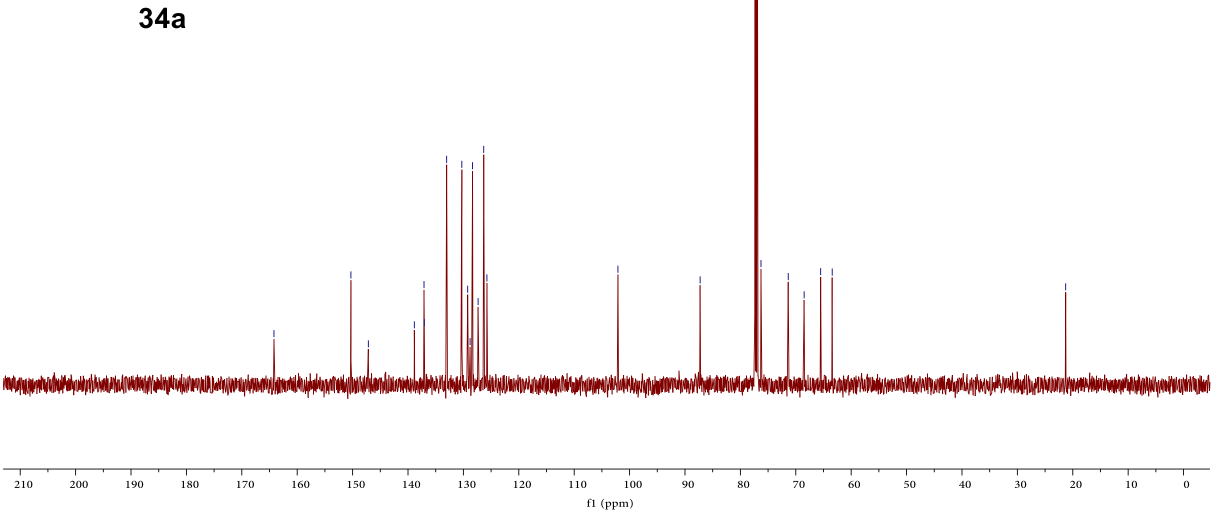

g0-hth-5-2-nap

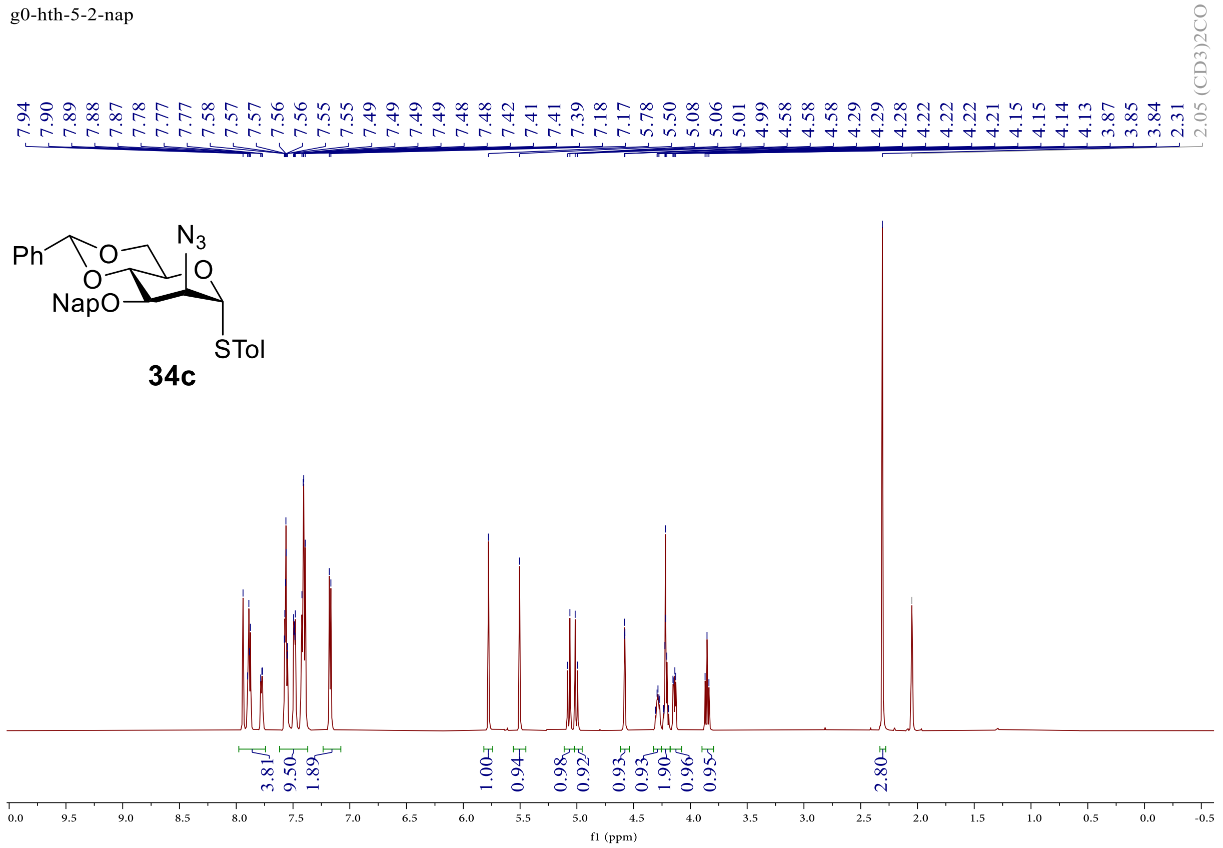
7.94
7.90
7.89
7.88
7.87
7.78
7.77
7.77
7.58
7.57
7.57
7.56
7.56
7.55
7.55
7.49
7.49
7.49
7.49
7.48
7.48
7.42
7.41
7.41
7.39
7.18
7.17
5.78
5.50
5.08
5.06
5.01
4.99
4.58
4.58
4.58
4.58
4.29
4.29
4.28
4.22
4.22
4.22
4.22
4.21
4.15
4.15
4.14
4.13
3.87
3.85
3.84
2.31
2.05 (CD3)2CO
Ph
O
N3
O
O
NapO
STol
34c
3.81
9.50
1.89
1.00
0.94
0.98
0.92
0.93
0.93
1.90
0.96
0.95
2.80
f1 (ppm)

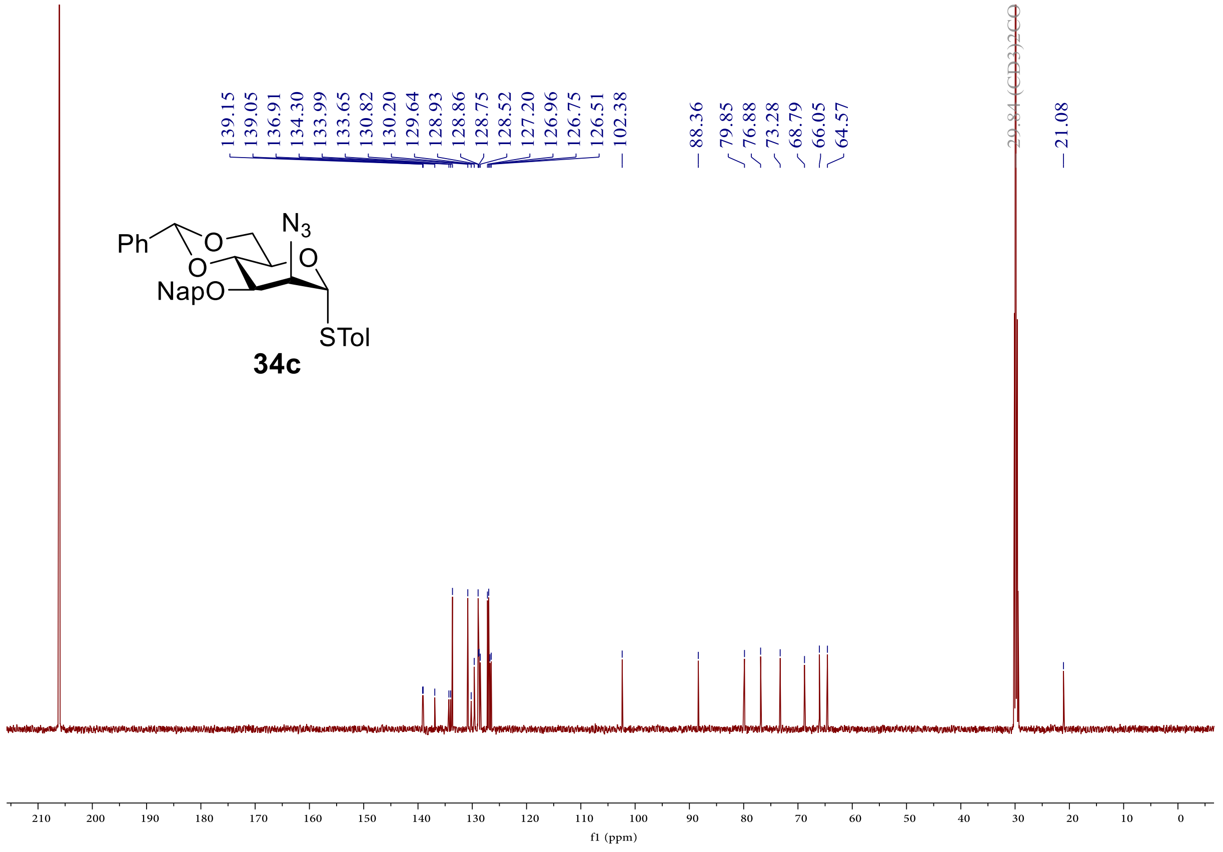
139.15
139.05
136.91
134.30
133.99
133.65
130.82
130.20
129.64
128.93
128.86
128.75
128.52
127.20
126.96
126.75
126.51
102.38
88.36
79.85
76.88
73.28
68.79
66.05
64.57
29.84 (CD3)2CO
21.08
Ph
O
N3
O
O
NapO
STol
34c
f1 (ppm)

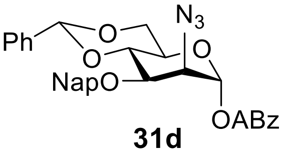

31d

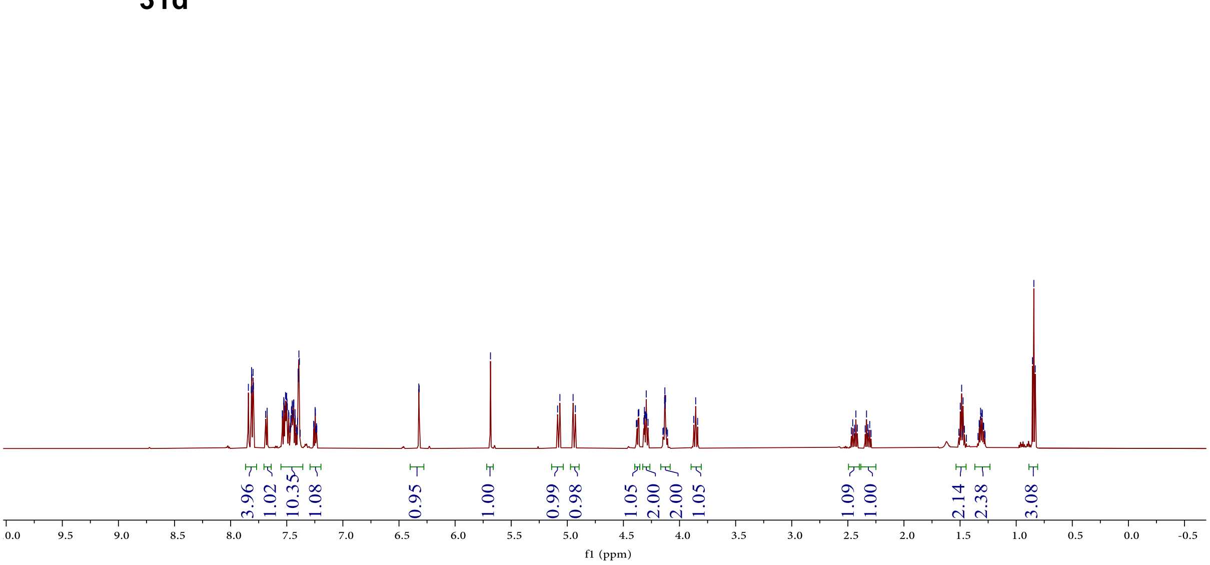

7.84 7.82 7.81 7.81 7.80 7.80 7.80 7.68 7.53 7.52 7.52 7.51 7.51 7.51 7.50 7.50 7.50 7.46 7.46 7.45 7.45 7.44 7.44 7.43 7.40 7.40 7.39 7.39 7.25 7.24 6.32 6.32 5.68 5.09 5.07 4.95 4.93 4.36 4.31 4.30 4.13 4.13 4.13 4.12 3.86 1.50 1.49 1.47 1.32 1.31 0.85 0.84 0.83
3.96 1.02 10.35 1.08 0.95 1.00 0.99 0.98 1.05 2.00 2.00 1.05 1.09 1.00 2.14 2.38 3.08
f1 (ppm)

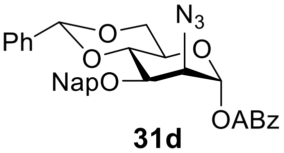

31d

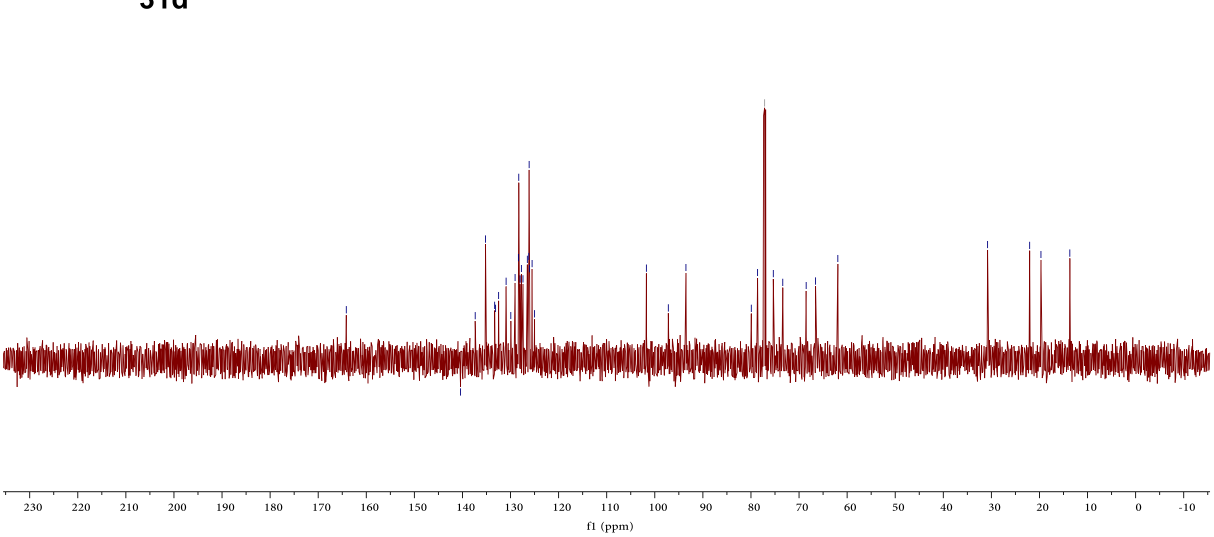

164.16 140.40 137.35 135.20 133.30 133.11 132.48 130.92 129.94 129.05 128.36 128.29 128.01 127.74 127.41 126.51 126.21 126.13 126.07 125.52 125.00 101.75 97.18 93.52 79.93 78.62 77.16 CDCl3 75.35 73.39 68.52 66.56 61.93 30.79 22.04 19.69 13.69
f1 (ppm)

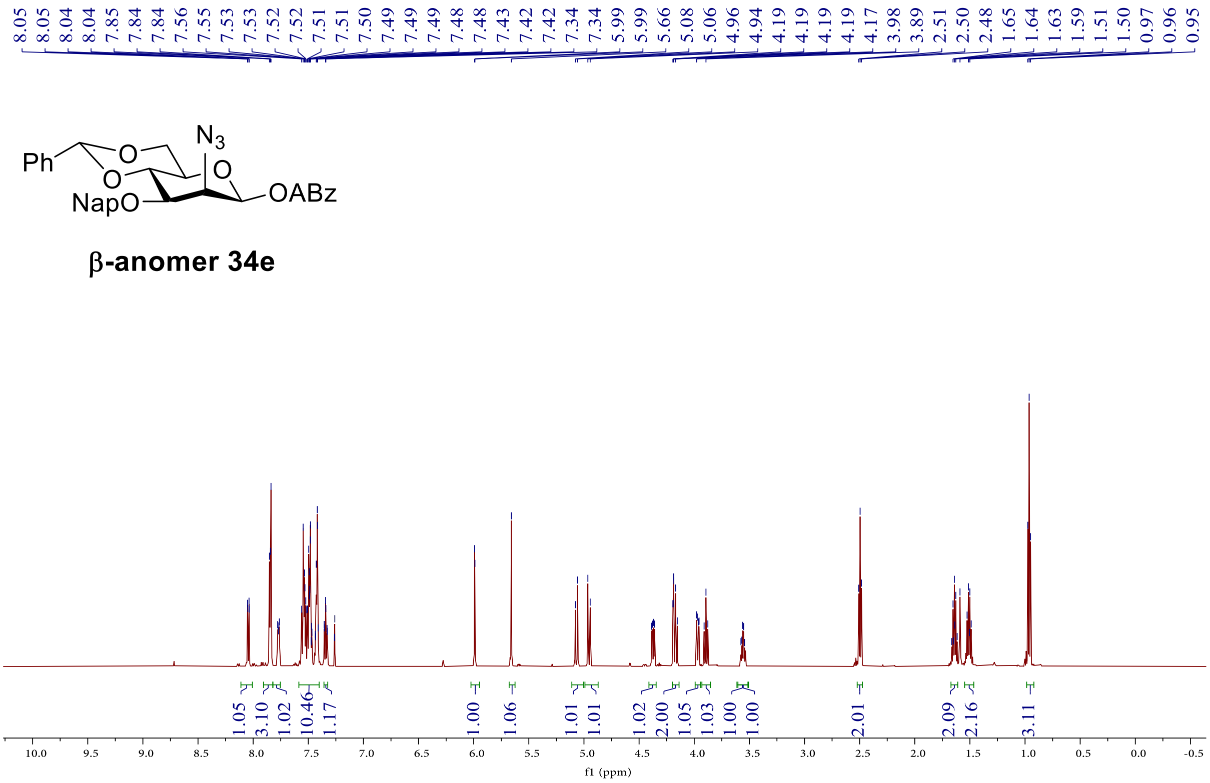
8.05 8.05 8.04 8.04 7.85 7.84 7.84 7.56 7.55 7.53 7.53 7.52 7.51 7.51 7.50 7.49 7.49 7.49 7.48 7.48 7.43 7.42 7.42 7.34 7.34 5.99 5.99 5.66 5.08 5.06 4.96 4.94 4.19 4.19 4.19 4.19 4.17 3.98 3.89 2.51 2.50 2.48 1.65 1.64 1.63 1.59 1.51 1.50 0.97 0.96 0.95
Ph
O
O
N3
O
NapO
OABz
β-anomer 34e
1.05 3.10 1.02 10.46 1.17 1.00 1.06 1.01 1.01 1.02 2.00 1.05 1.03 1.00 1.00 2.01 2.09 2.16 3.11
10.0 9.5 9.0 8.5 8.0 7.5 7.0 6.5 6.0 5.5 5.0 4.5 4.0 3.5 3.0 2.5 2.0 1.5 1.0 0.5 0.0 -0.5
f1 (ppm)

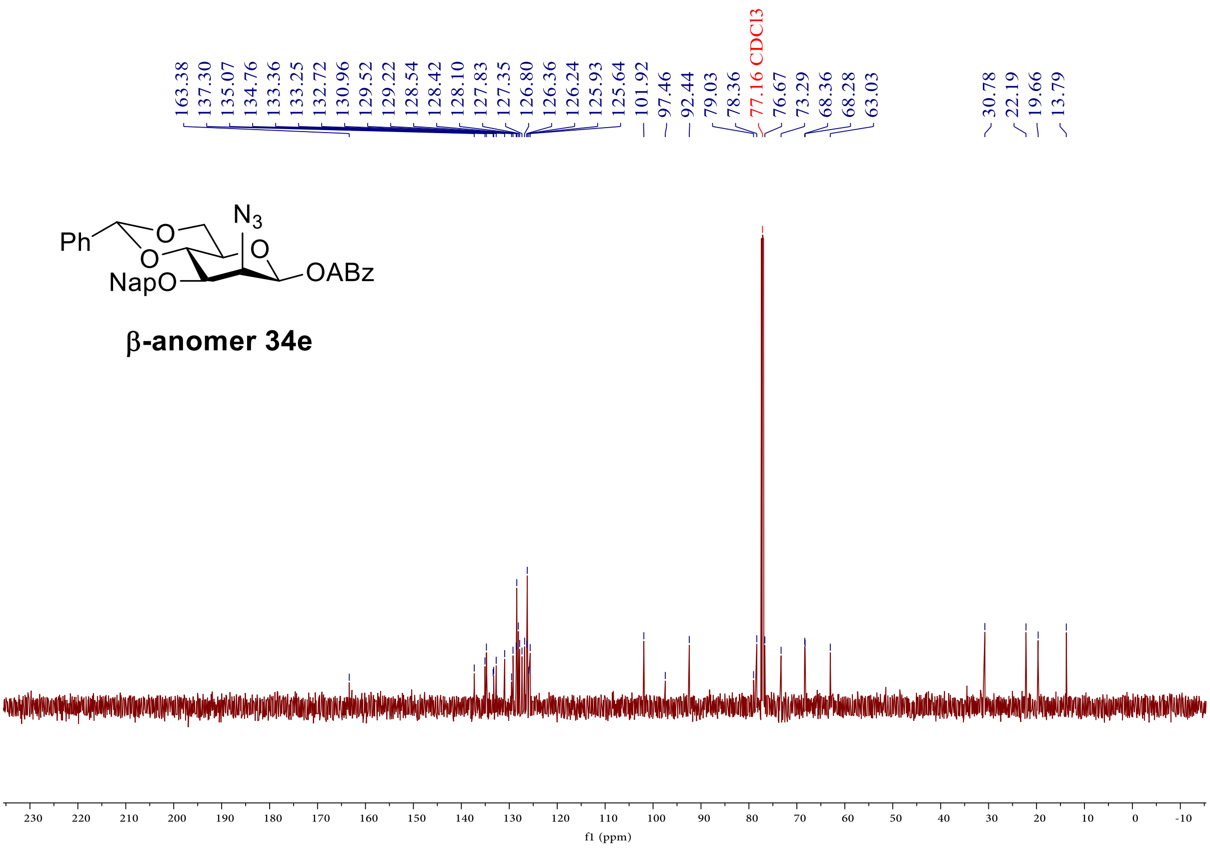
163.38 137.30 135.07 134.76 133.36 133.25 132.72 130.96 129.52 129.22 128.54 128.42 128.10 127.83 127.35 126.80 126.36 126.24 125.93 125.64 101.92 97.46 92.44 79.03 78.36 77.16 CDCl3 76.67 73.29 68.36 68.28 63.03 30.78 22.19 19.66 13.79
Ph
O
O
N3
O
NapO
OABz
β-anomer 34e
230 220 210 200 190 180 170 160 150 140 130 120 110 100 90 80 70 60 50 40 30 20 10 0 -10
f1 (ppm)

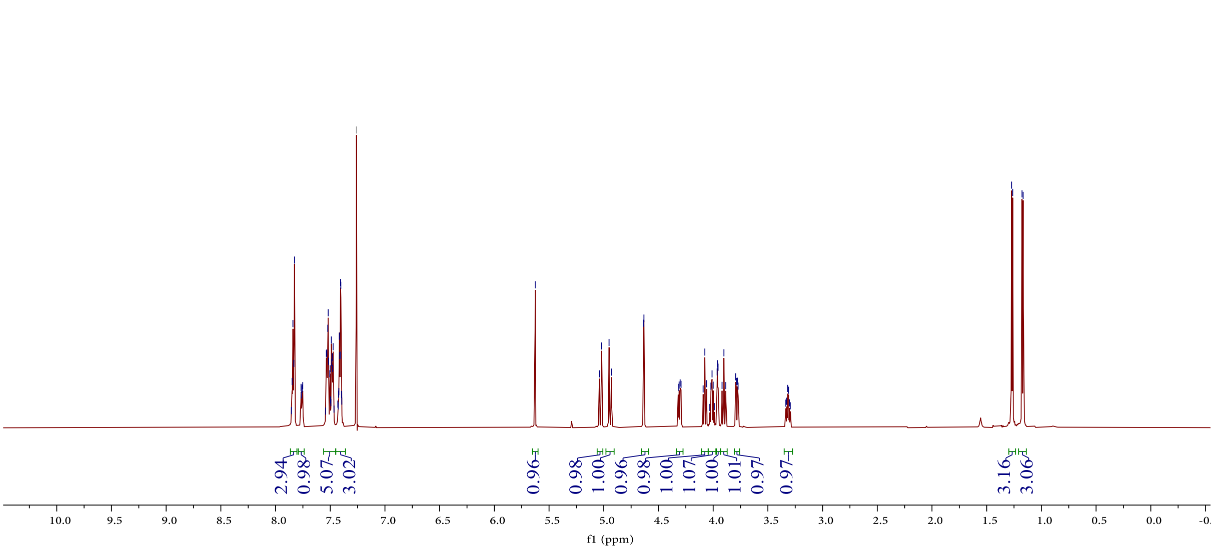
7.85 7.84 7.83 7.83 7.75 7.54 7.53 7.52 7.52 7.51 7.50 7.49 7.49 7.48 7.48 7.47 7.42 7.41 7.41 7.41 7.40 7.26 CDCl3 5.63 5.04 5.02 4.95 4.93 4.64 4.63 4.31 4.30 4.29 4.08 4.06 4.01 4.00 3.96 3.96 3.96 3.92 3.90 3.88 3.79 3.79 3.78 1.27 1.26 1.18 1.17
2.94 0.98 5.07 3.02 0.96 0.98 1.00 0.96 0.98 1.00 1.07 1.00 1.01 0.97 0.97 3.16 3.06
f1 (ppm)

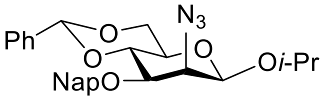
Ph
O
O
N3
O
Oi-Pr
NapO

S12

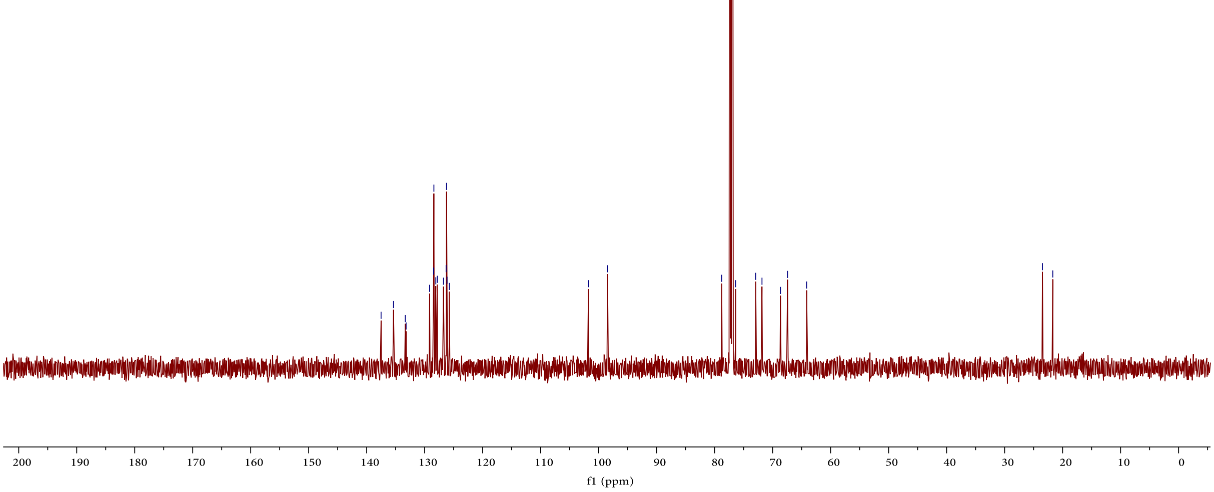
137.52 135.37 133.35 133.20 129.15 128.46 128.41 128.08 127.83 126.75 126.31 126.23 126.15 125.75 101.76 98.47 78.78 77.16 CDCl3 76.38 72.92 71.84 68.65 67.43 64.14 23.47 21.70
f1 (ppm)

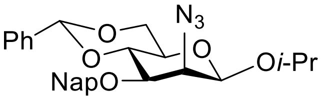
Ph
O
O
N3
O
Oi-Pr
NapO

S12

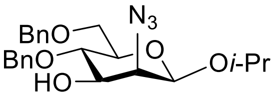
BnO
BnO
HO
N3
O
Oi-Pr

44

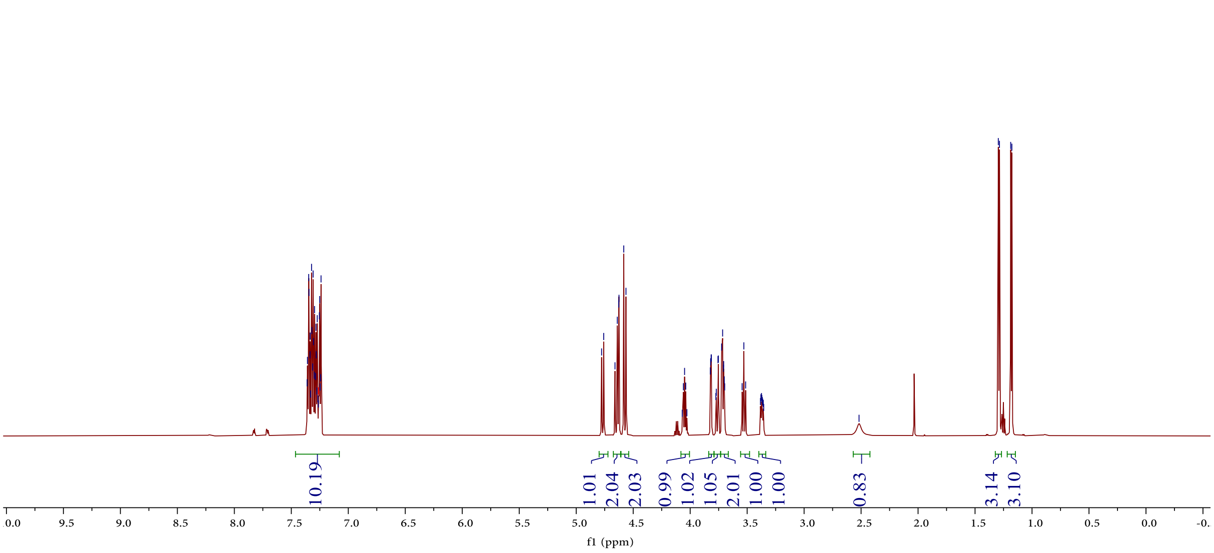
7.36 7.36 7.35 7.34 7.34 7.33 7.32 7.32 7.31 7.31 7.30 7.29 7.28 7.28 7.27 7.25 7.25 7.24 7.24 4.78 4.76 4.66 4.64 4.63 4.62 4.58 4.56 4.06 4.05 4.04 3.82 3.82 3.82 3.81 3.76 3.75 3.72 3.72 3.71 3.71 3.70 3.70 3.54 3.53 3.51 1.30 1.29 1.19 1.18
10.19
1.01
2.04
2.03
0.99
1.02
1.05
2.01
1.00
1.00
0.83
3.14
3.10
f1 (ppm)

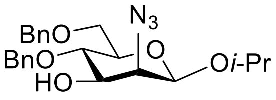
BnO
BnO
HO
N3
O
Oi-Pr

44

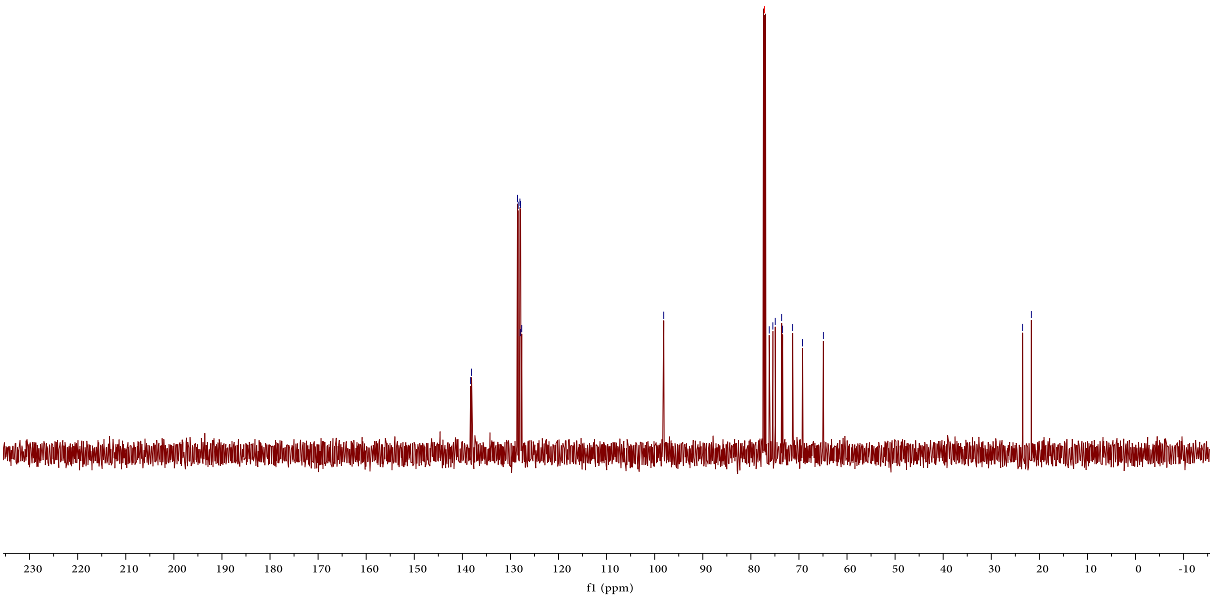
138.28 138.08 128.54 128.39 128.03 127.97 127.89 127.65
98.13
77.16 CDCl3
76.18 75.43 74.93 73.59 73.39 71.31 69.25 64.92
23.48 21.67
f1 (ppm)

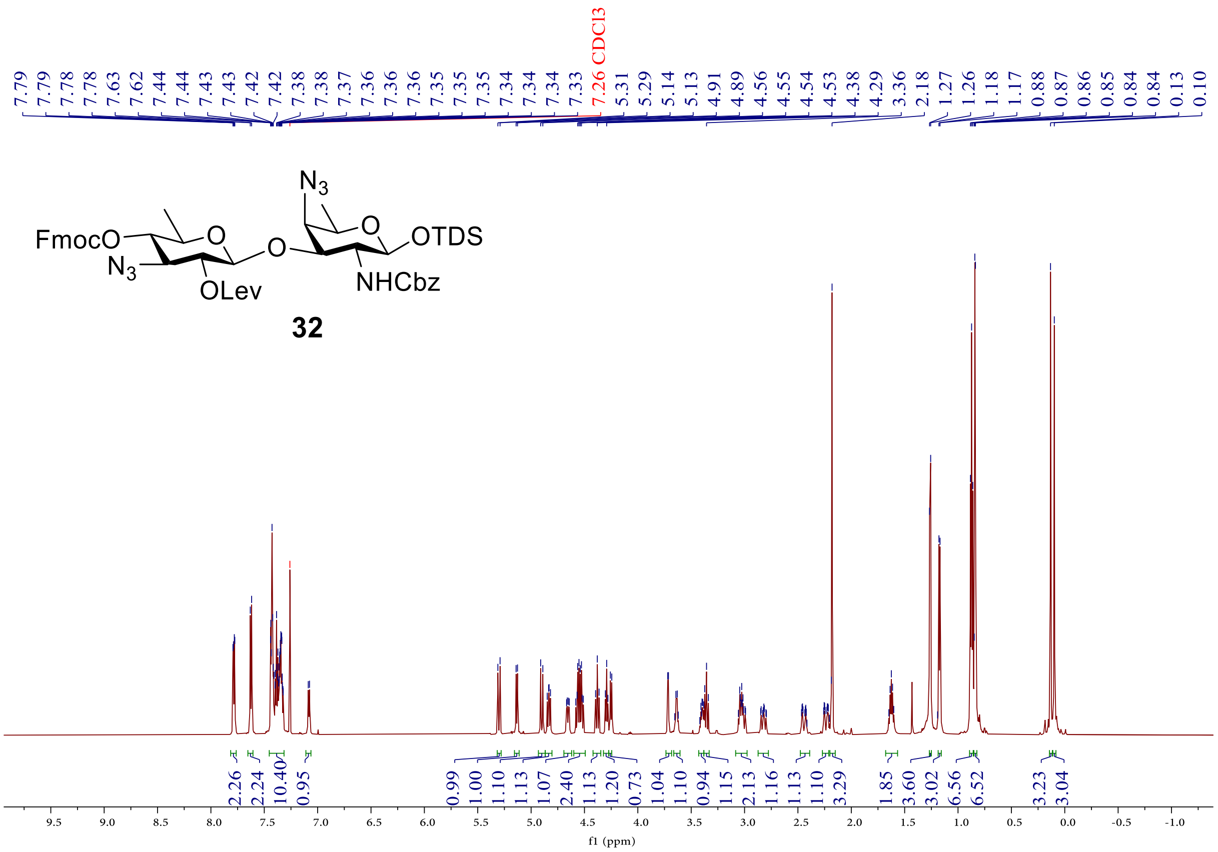
7.79 7.79 7.78 7.78 7.63 7.62 7.44 7.44 7.43 7.43 7.42 7.42 7.38 7.38 7.37 7.36 7.36 7.36 7.35 7.35 7.35 7.34 7.34 7.34 7.33 7.26 CDCl3 5.31 5.29 5.14 5.13 4.91 4.89 4.56 4.55 4.54 4.53 4.38 4.29 3.36 2.18 1.27 1.26 1.18 1.17 0.88 0.87 0.86 0.85 0.84 0.84 0.13 0.10
FmocO
N3
OLev
O
N3
OTDS
NHCbz
32
2.26 2.24 10.40 0.95 0.99 1.00 1.10 1.13 1.07 2.40 1.13 1.20 0.73 1.04 1.10 0.94 1.15 2.13 1.16 1.13 1.10 3.29 1.85 3.60 3.02 6.56 6.52 3.23 3.04
f1 (ppm)

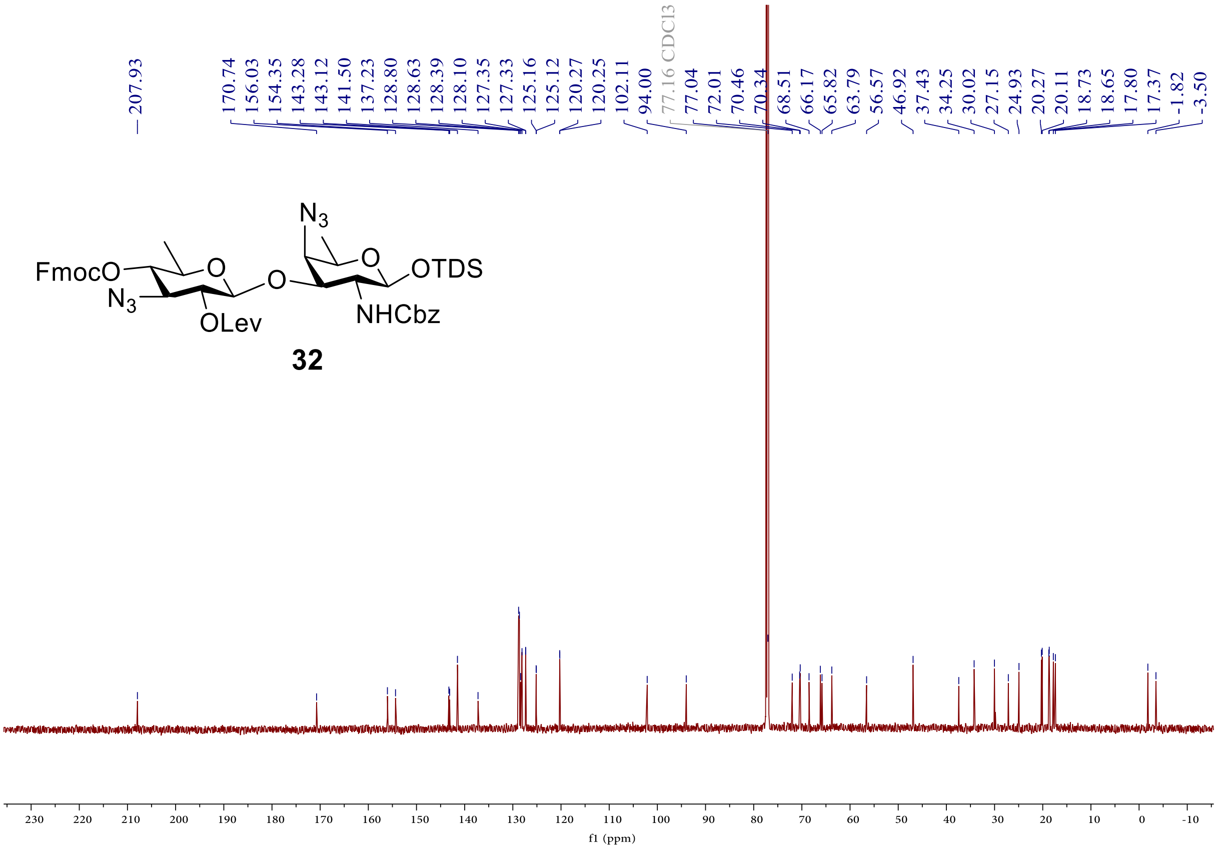
207.93 170.74 156.03 154.35 143.28 143.12 141.50 137.23 128.80 128.63 128.39 128.10 127.35 127.33 125.16 125.12 120.27 120.25 102.11 94.00 77.16 CDCl3 77.04 72.01 70.46 70.34 68.51 66.17 65.82 63.79 56.57 46.92 37.43 34.25 30.02 27.15 24.93 20.27 20.11 18.73 18.65 17.80 17.37 -1.82 -3.50
FmocO
N3
OLev
O
N3
OTDS
NHCbz
32
f1 (ppm)

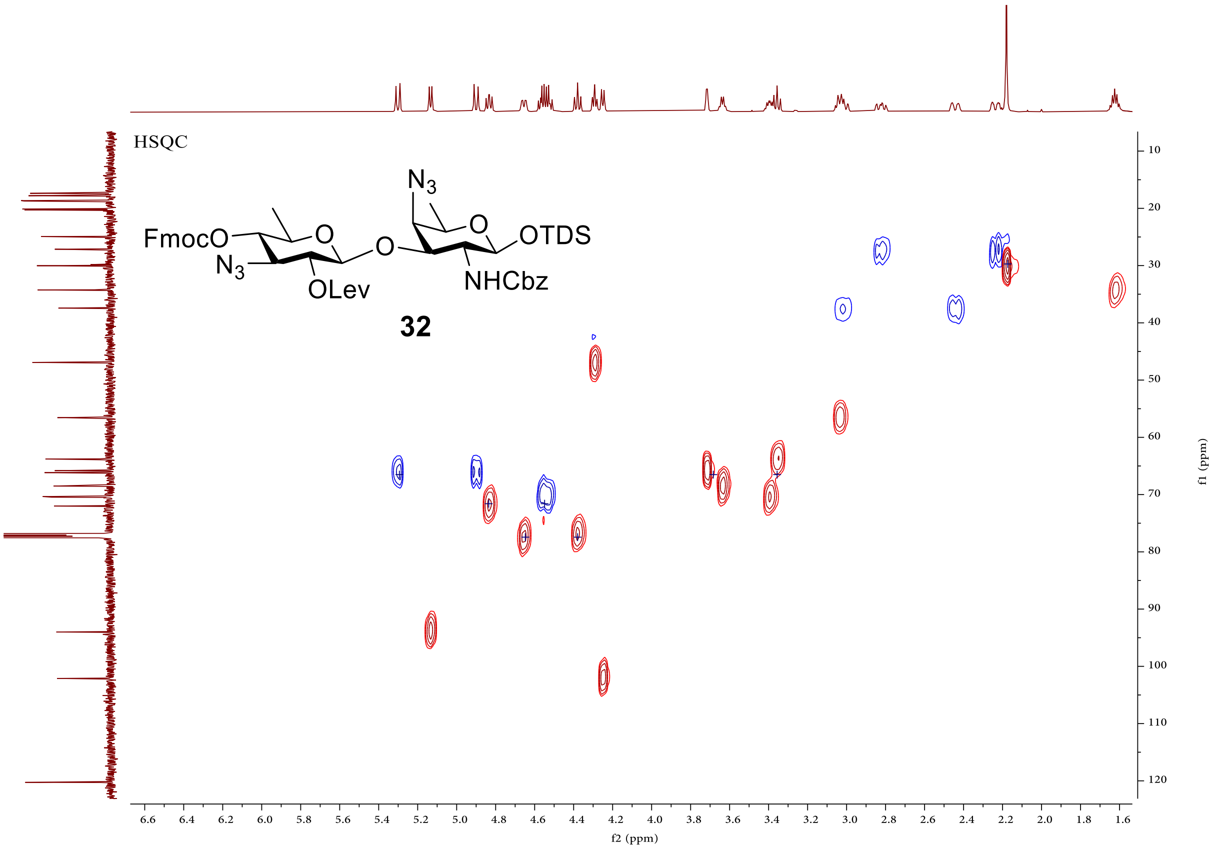
HSQC
FmocO
N3
O
OLev
O
N3
O
OTDS
NHCbz
32
f2 (ppm)
f1 (ppm)

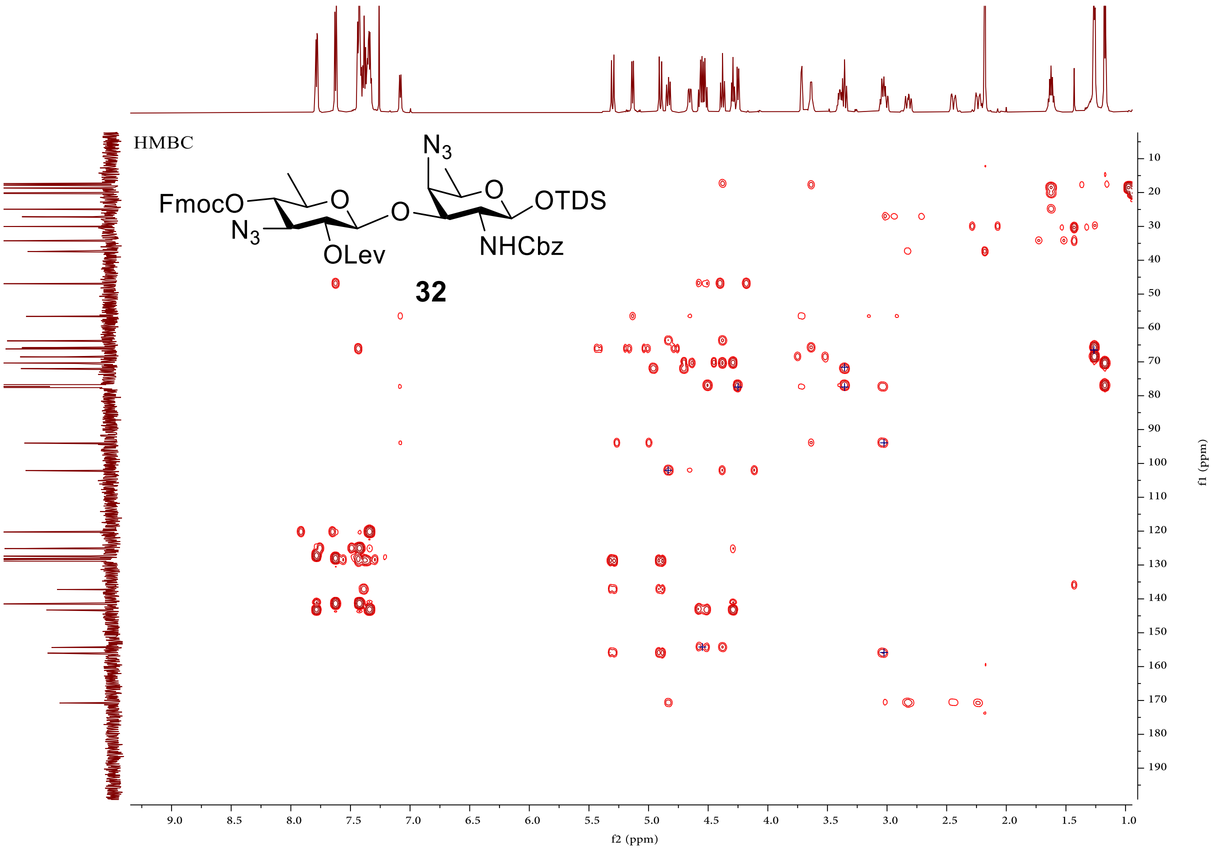
HMBC
FmocO
N3
O
OLev
O
N3
O
OTDS
NHCbz
32
f2 (ppm)
f1 (ppm)

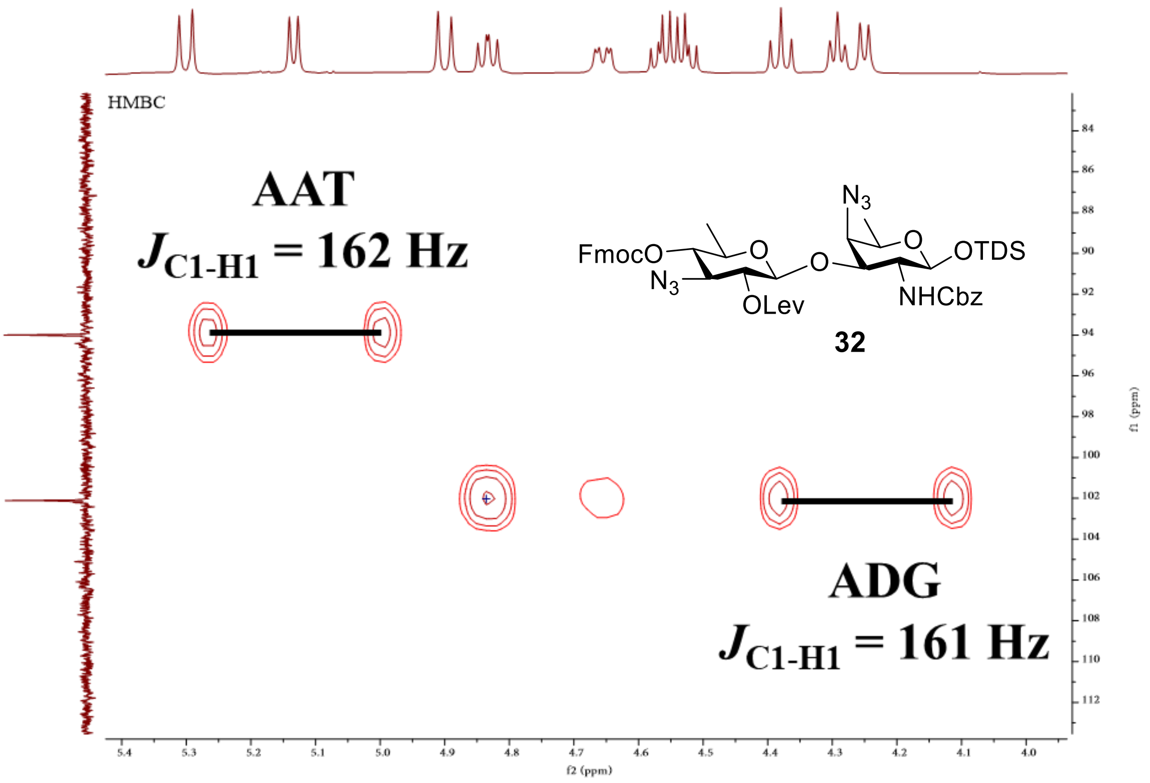
HMBC
AAT
J_{C1-H1} = 162 Hz
FmocO
N3
O
OLev
O
N3
O
OTDS
NHCbz
32
ADG
J_{C1-H1} = 161 Hz
f2 (ppm)
f1 (ppm)

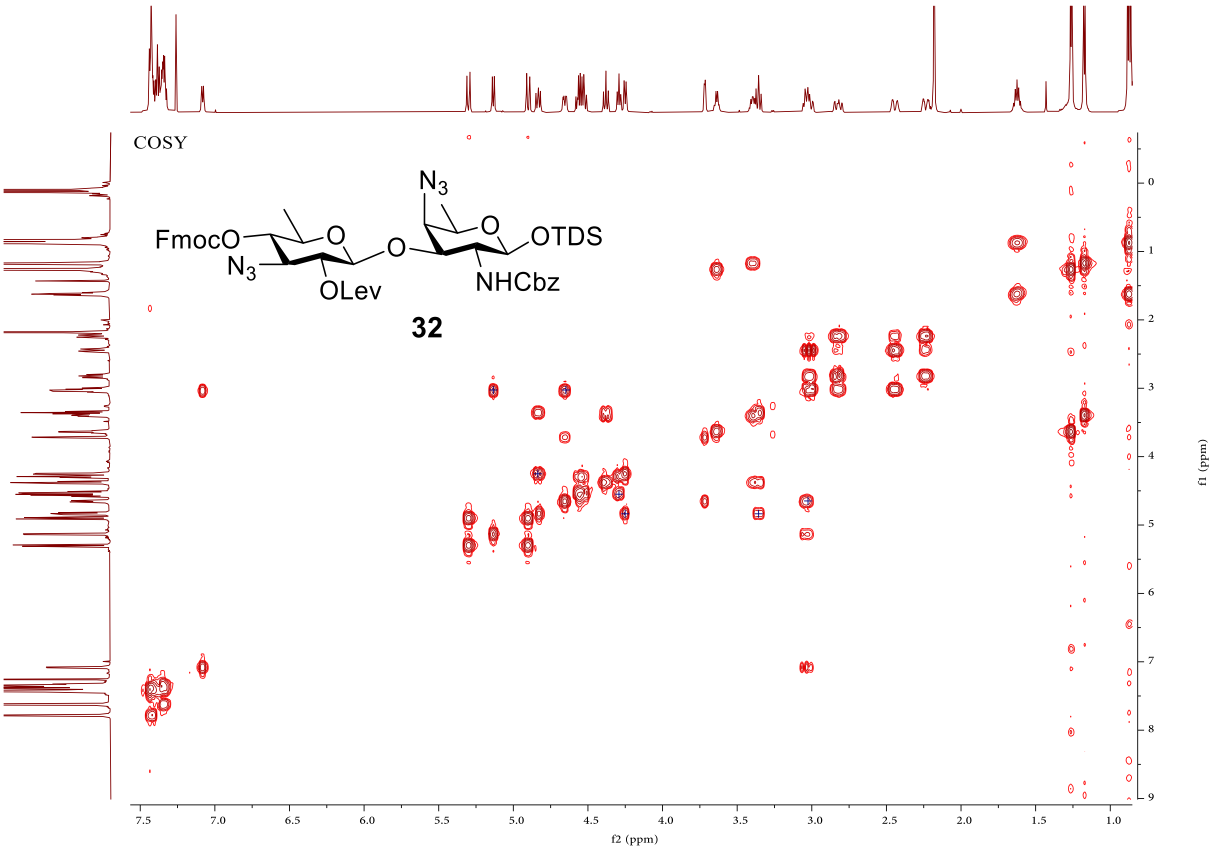
COSY
FmocO
N3
O
OLev
O
N3
O
OTDS
NHCbz
32
f2 (ppm)
f1 (ppm)

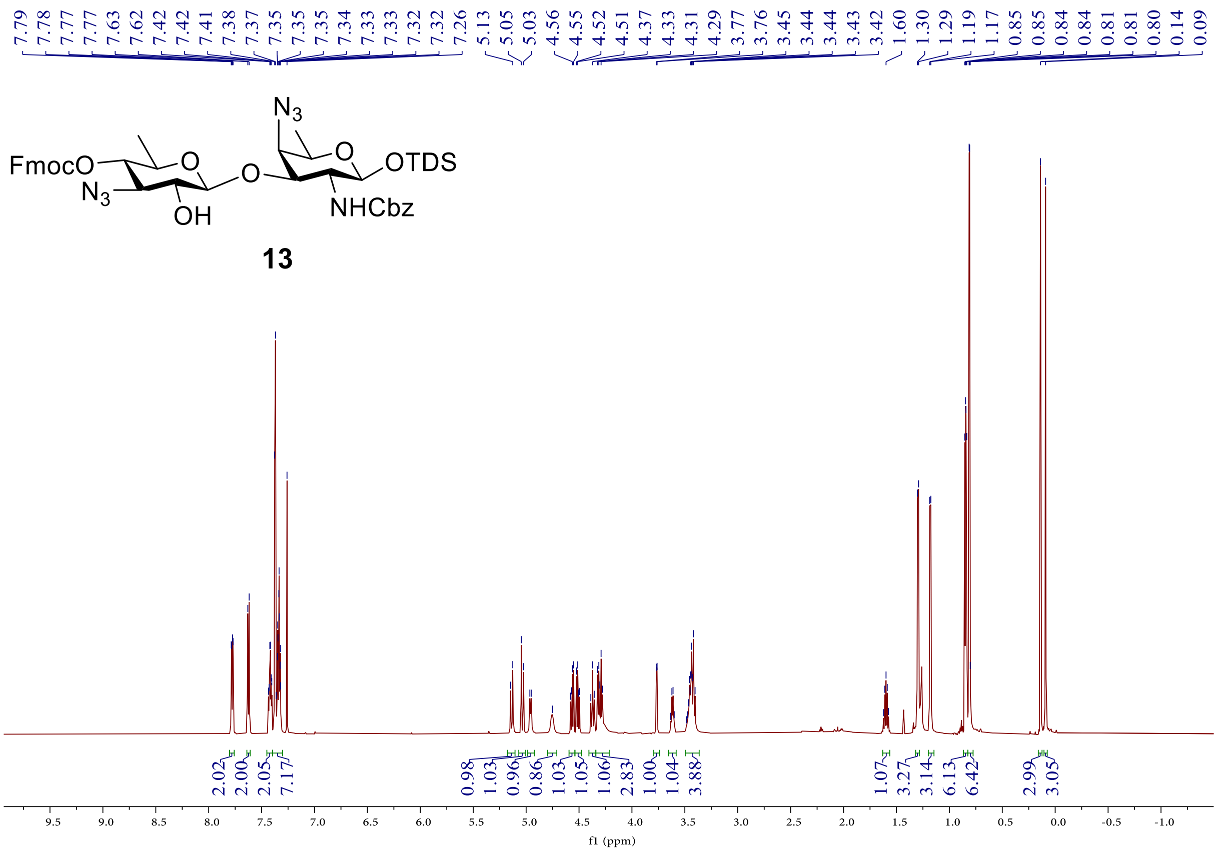

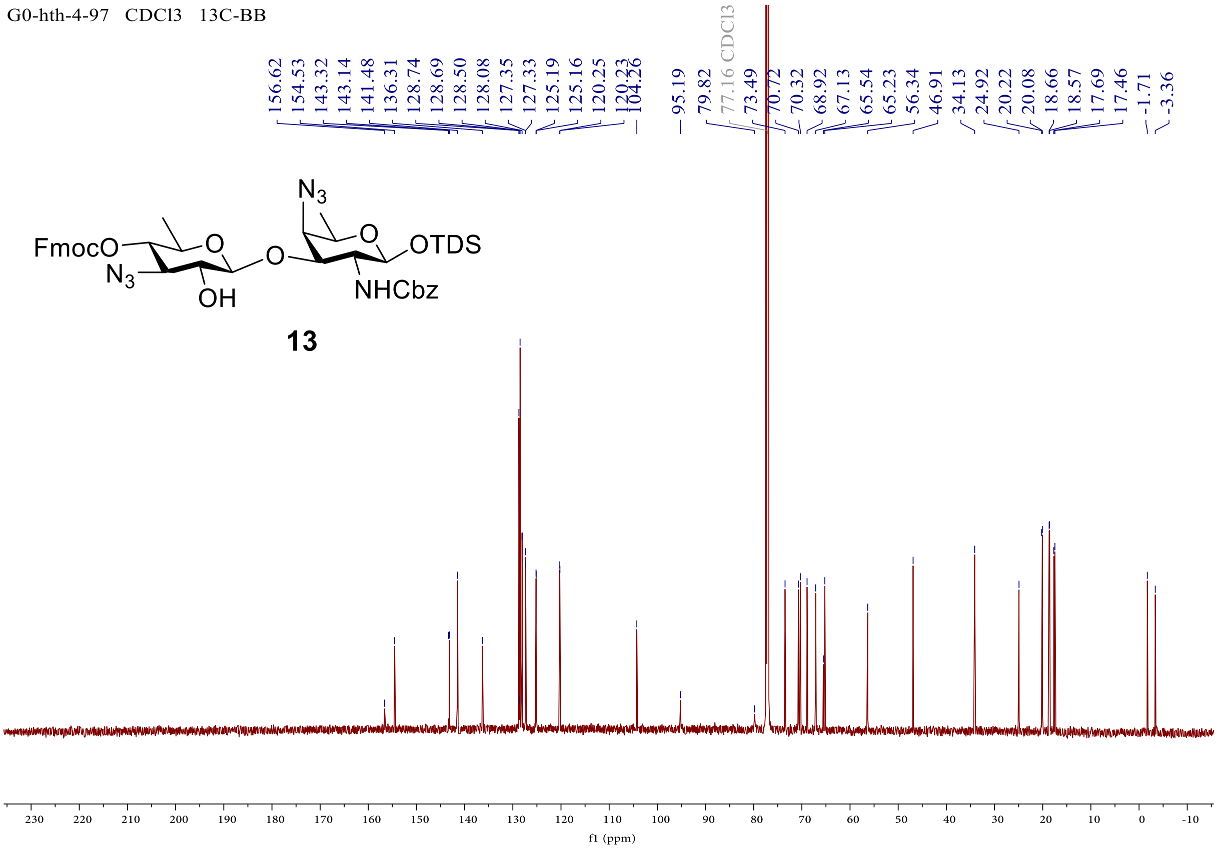
G0-hth-4-97 CDCl3 13C-BB

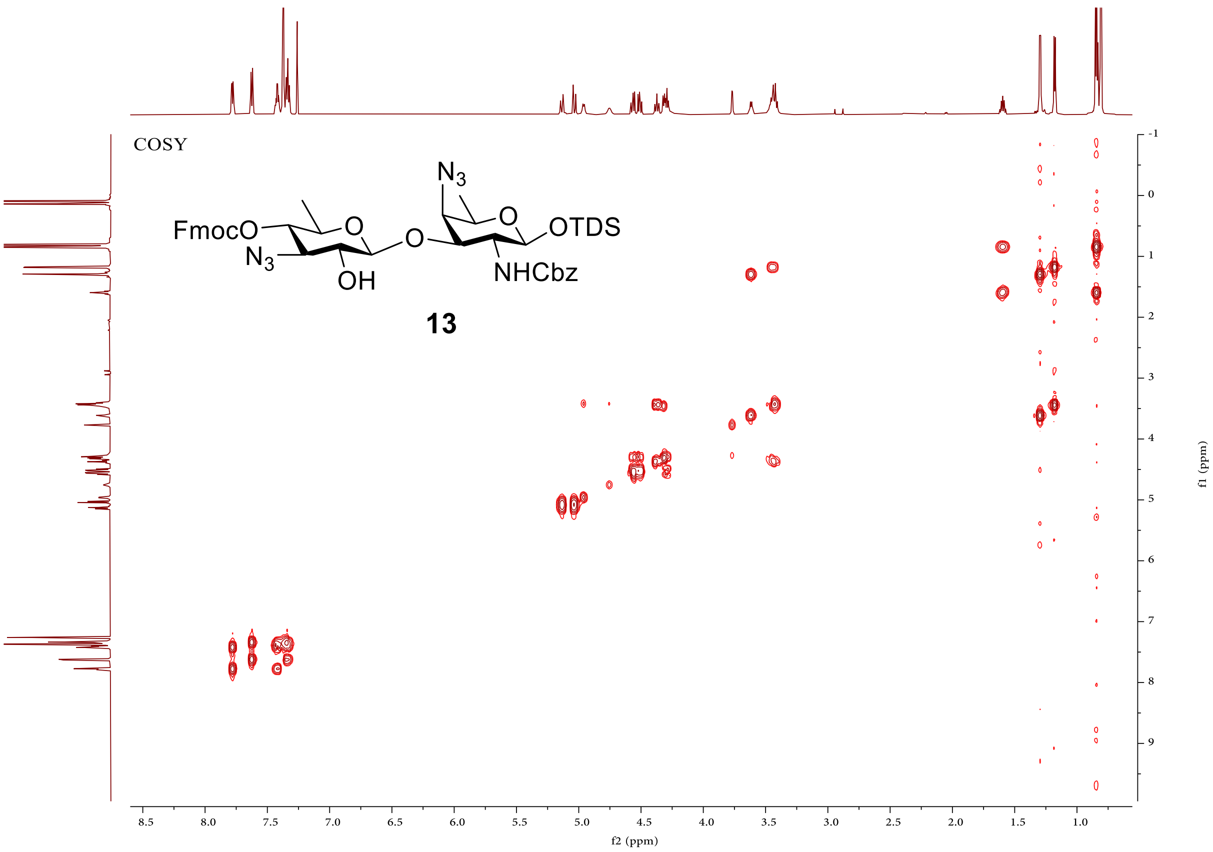
COSY
FmocO
N3
OH
O
N3
O
OTDS
NHCbz
13
f2 (ppm)
f1 (ppm)

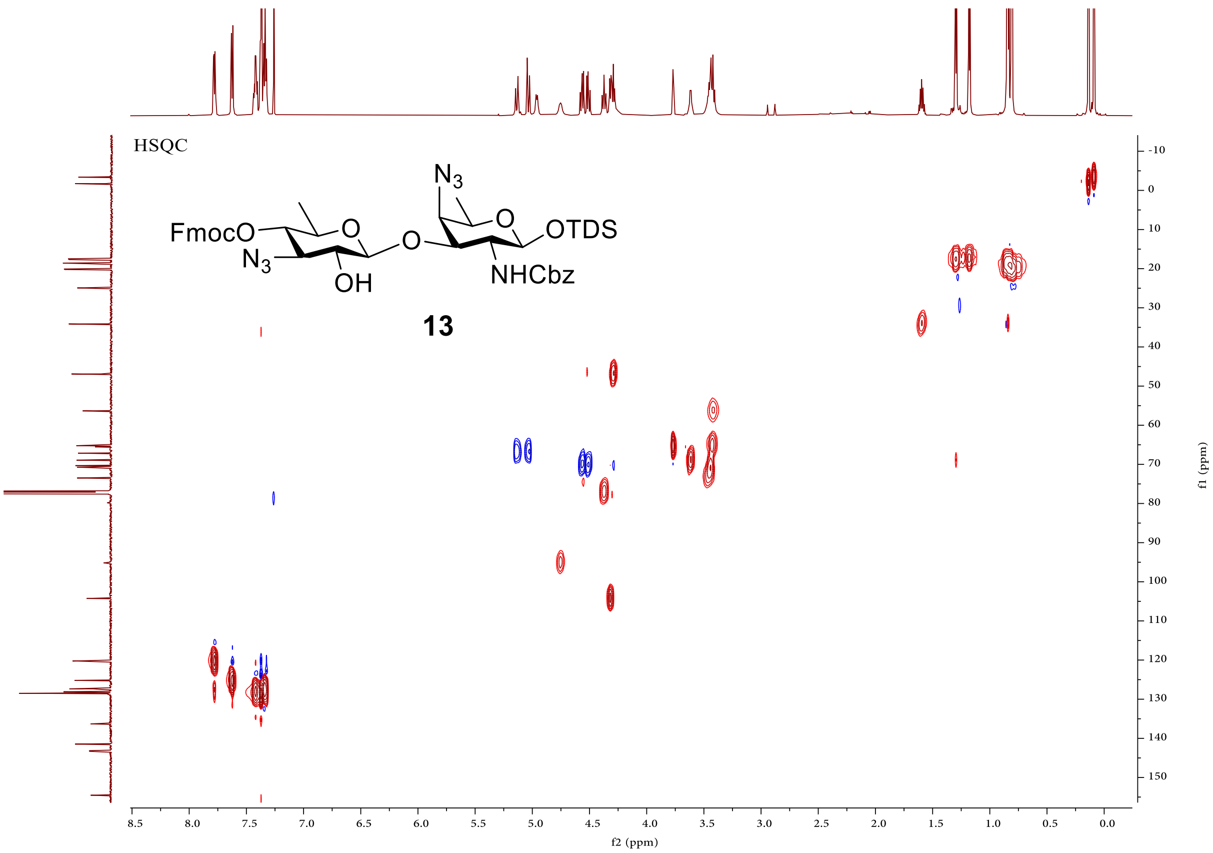
HSQC
FmocO
N3
OH
O
N3
O
OTDS
NHCbz
13
f2 (ppm)
f1 (ppm)

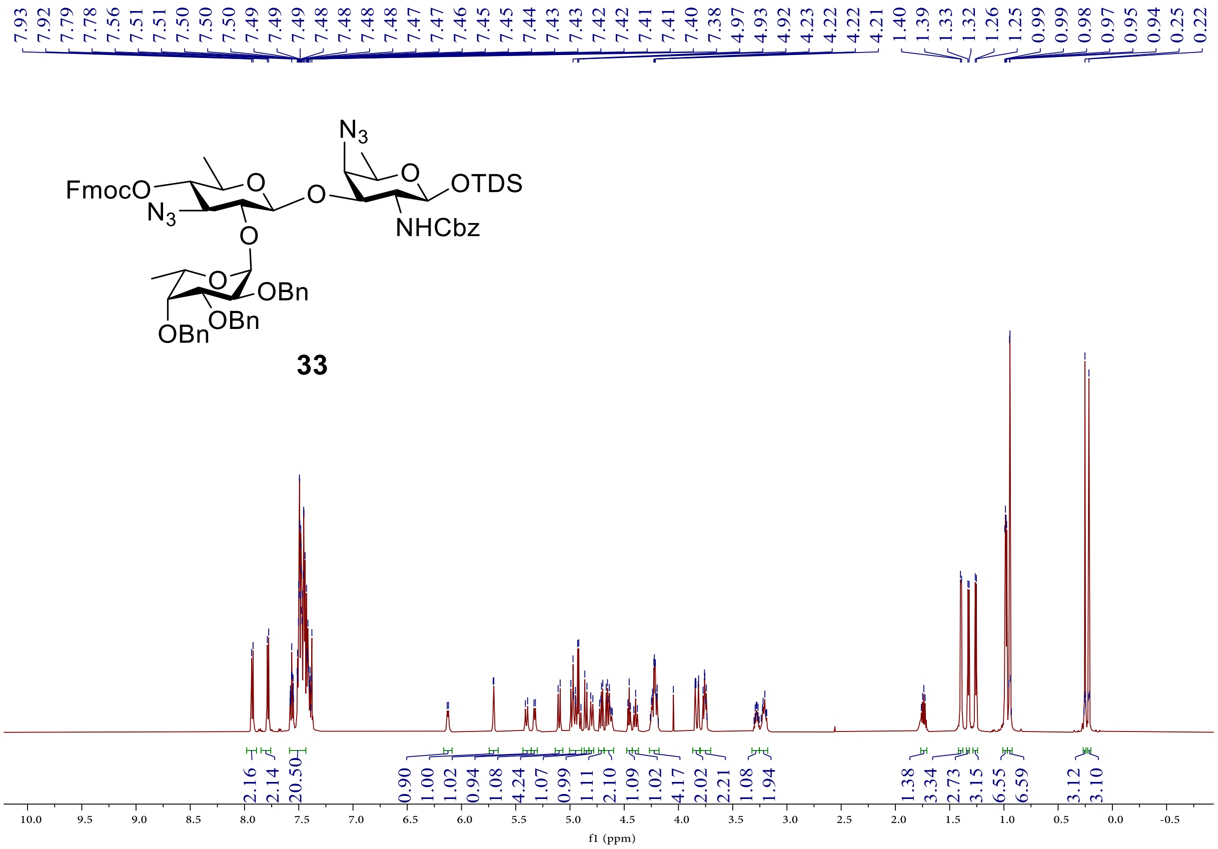
7.93 7.92 7.79 7.78 7.56 7.51 7.50 7.50 7.50 7.49 7.49 7.49 7.48 7.48 7.48 7.48 7.47 7.47 7.46 7.45 7.45 7.44 7.43 7.43 7.42 7.42 7.41 7.41 7.40 7.38 4.97 4.93 4.92 4.23 4.22 4.22 4.21 1.40 1.39 1.33 1.32 1.26 1.25 0.99 0.99 0.98 0.97 0.95 0.94 0.25 0.22
FmocO
N_3
NHCbz
OTDS
OBn
OBn
OBn
33
2.16 2.14 20.50 0.90 1.00 1.02 0.94 1.08 4.24 1.07 0.99 1.11 2.10 1.09 1.02 4.17 2.02 2.21 1.08 1.94 1.38 3.34 2.73 3.15 6.55 6.59 3.12 3.10
f1 (ppm)

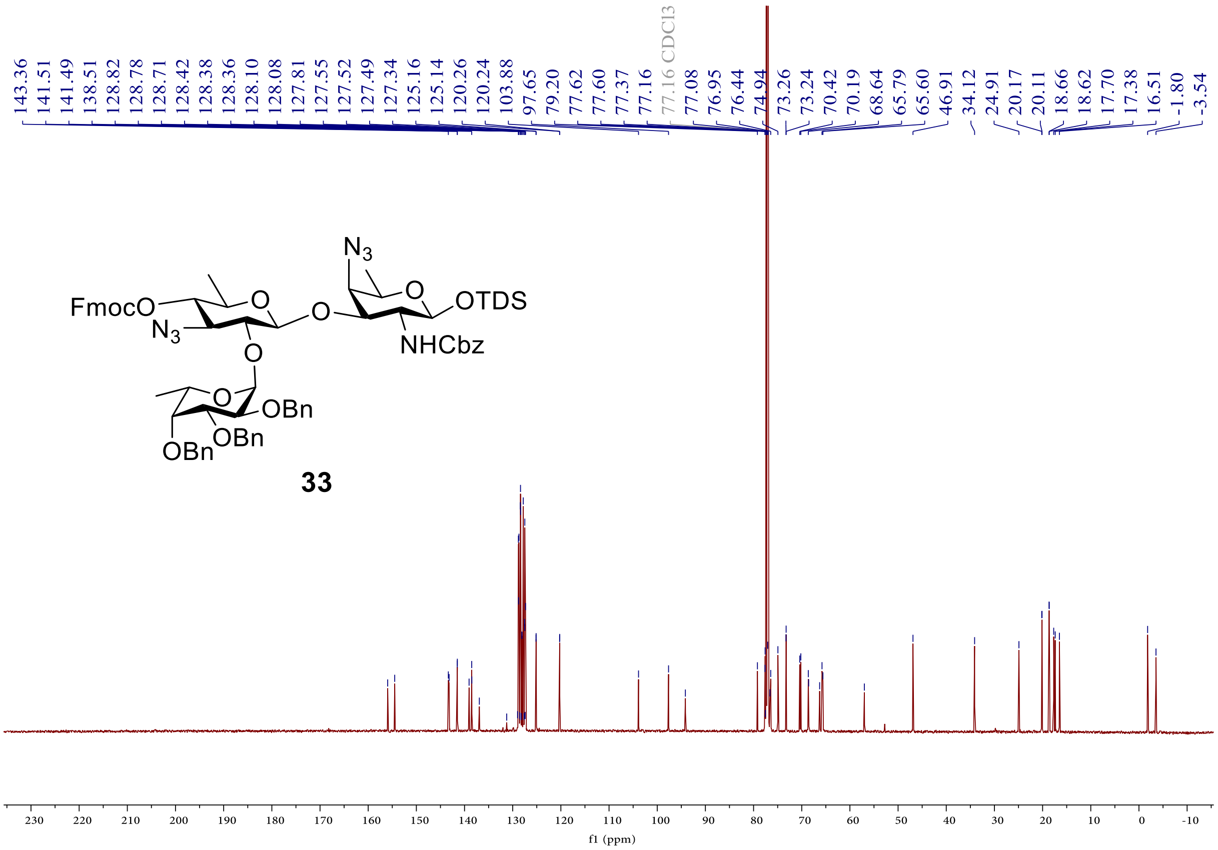
143.36 141.51 141.49 138.51 128.82 128.78 128.71 128.42 128.38 128.36 128.10 128.08 127.81 127.55 127.52 127.49 127.34 125.16 125.14 120.26 120.24 103.88 97.65 79.20 77.62 77.60 77.37 77.16 77.16 CDCl3 77.08 76.95 76.44 74.94 73.26 73.24 70.42 70.19 68.64 65.79 65.60 46.91 34.12 24.91 20.17 20.11 18.66 18.62 17.70 17.38 16.51 -1.80 -3.54
FmocO
N_3
NHCbz
OTDS
OBn
OBn
OBn
33
f1 (ppm)

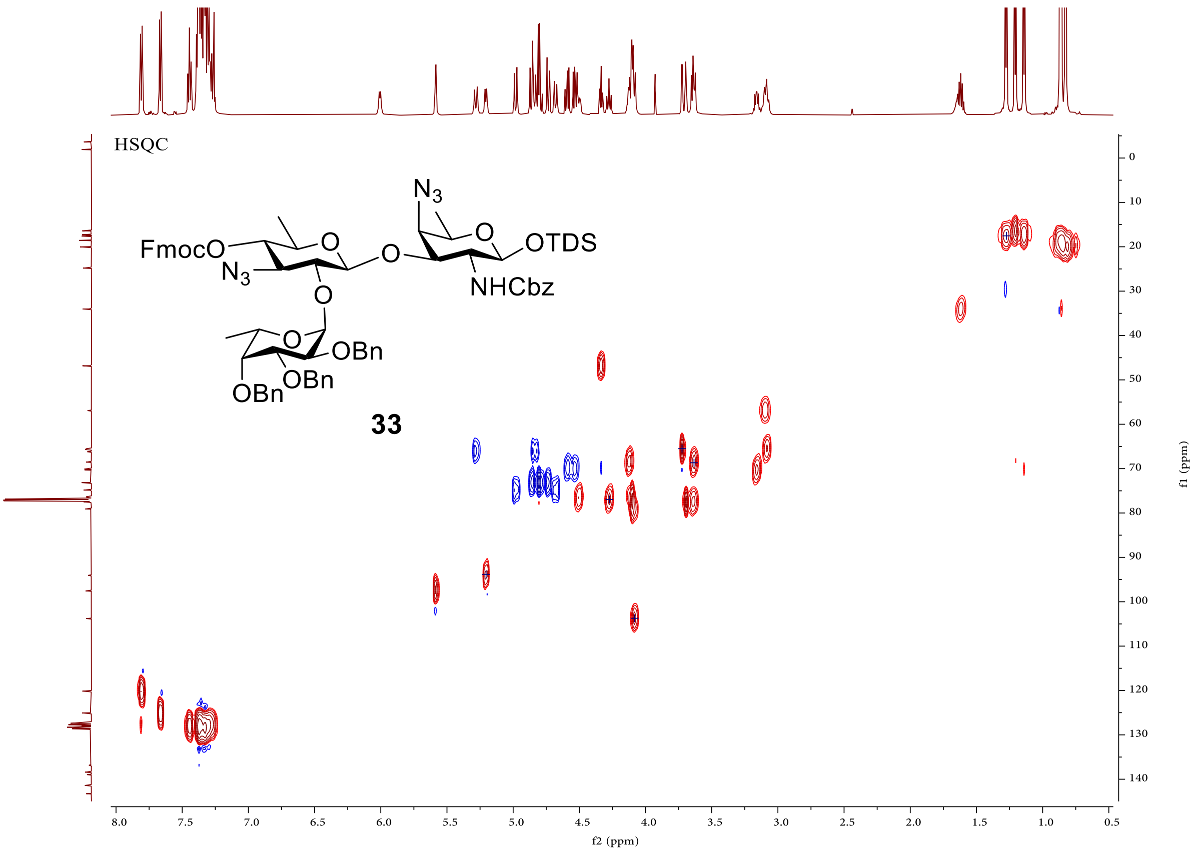
HSQC
FmocO
N3
O
O
N3
O
OTDS
NHCbz
O
O
OBn
OBn
OBn
33
f2 (ppm)
f1 (ppm)

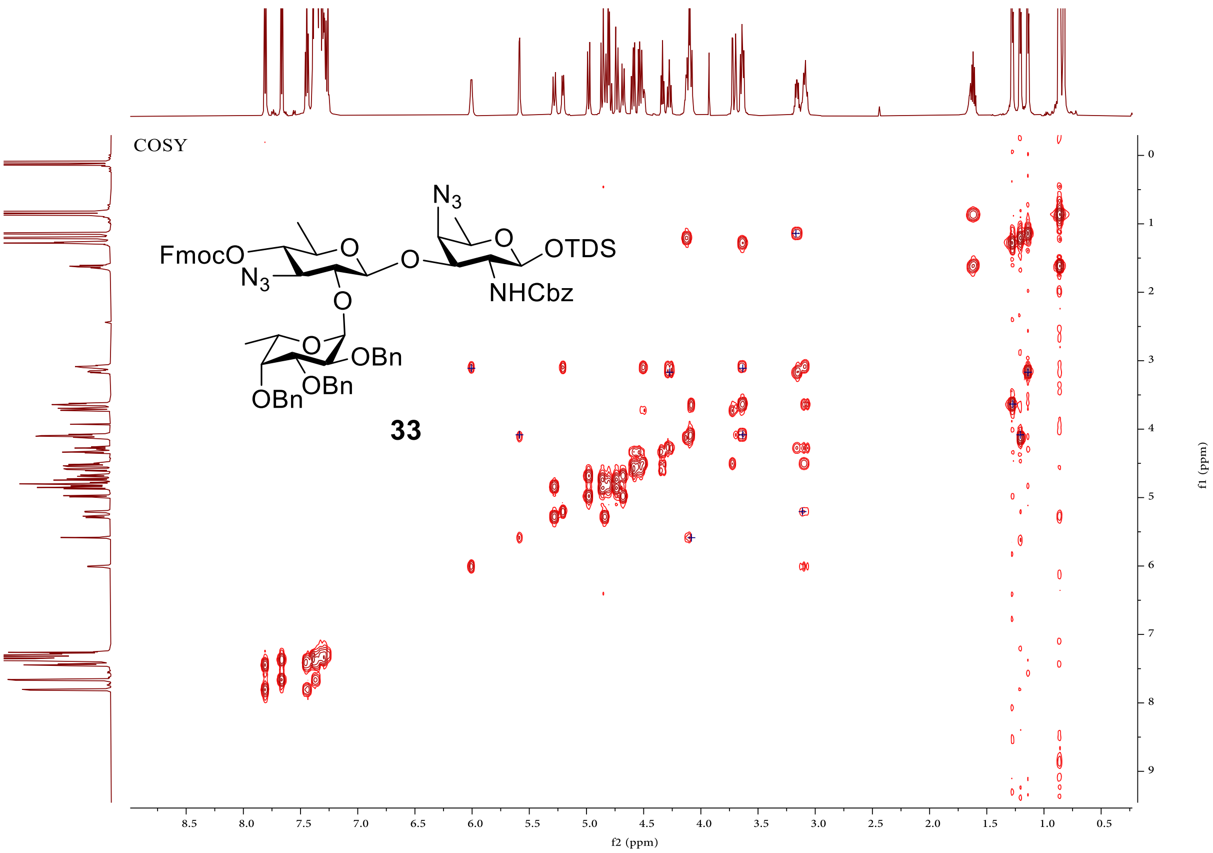
COSY
FmocO
N3
O
O
N3
O
OTDS
NHCbz
O
O
OBn
OBn
OBn
33
f2 (ppm)
f1 (ppm)

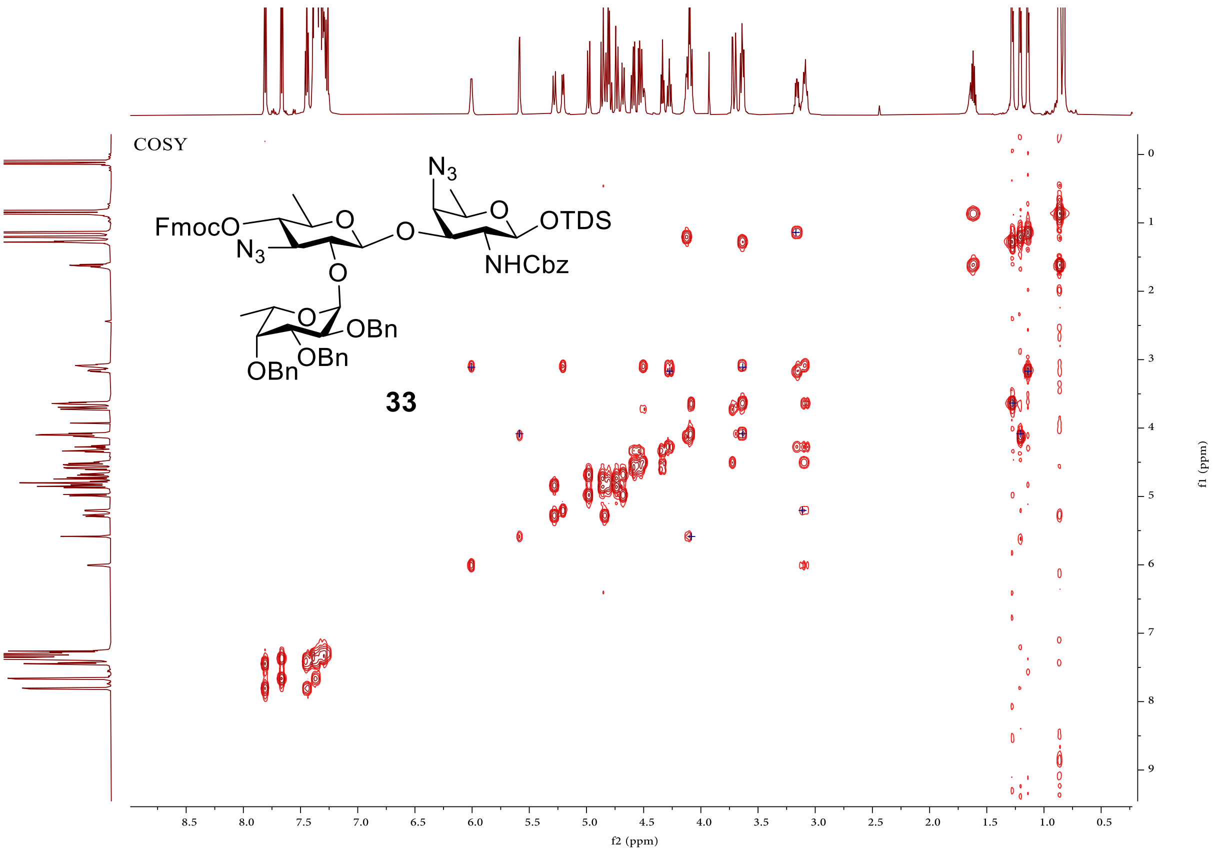
COSY
FmocO
N3
O
O
N3
O
OTDS
NHCbz
O
O
OBn
OBn
OBn
33
f2 (ppm)
f1 (ppm)

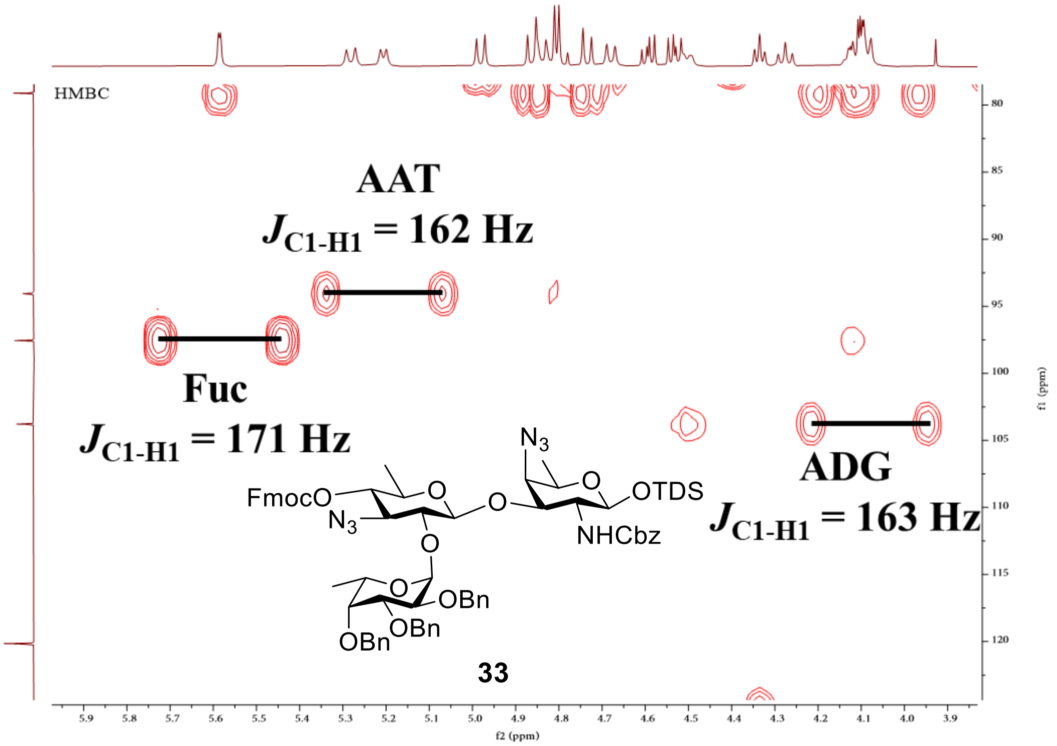
HMBC
AAT
JC1-H1 = 162 Hz
Fuc
JC1-H1 = 171 Hz
ADG
JC1-H1 = 163 Hz
FmocO
N3
O
O
N3
O
OTDS
NHCbz
O
O
OBn
OBn
OBn
33
f2 (ppm)
f1 (ppm)

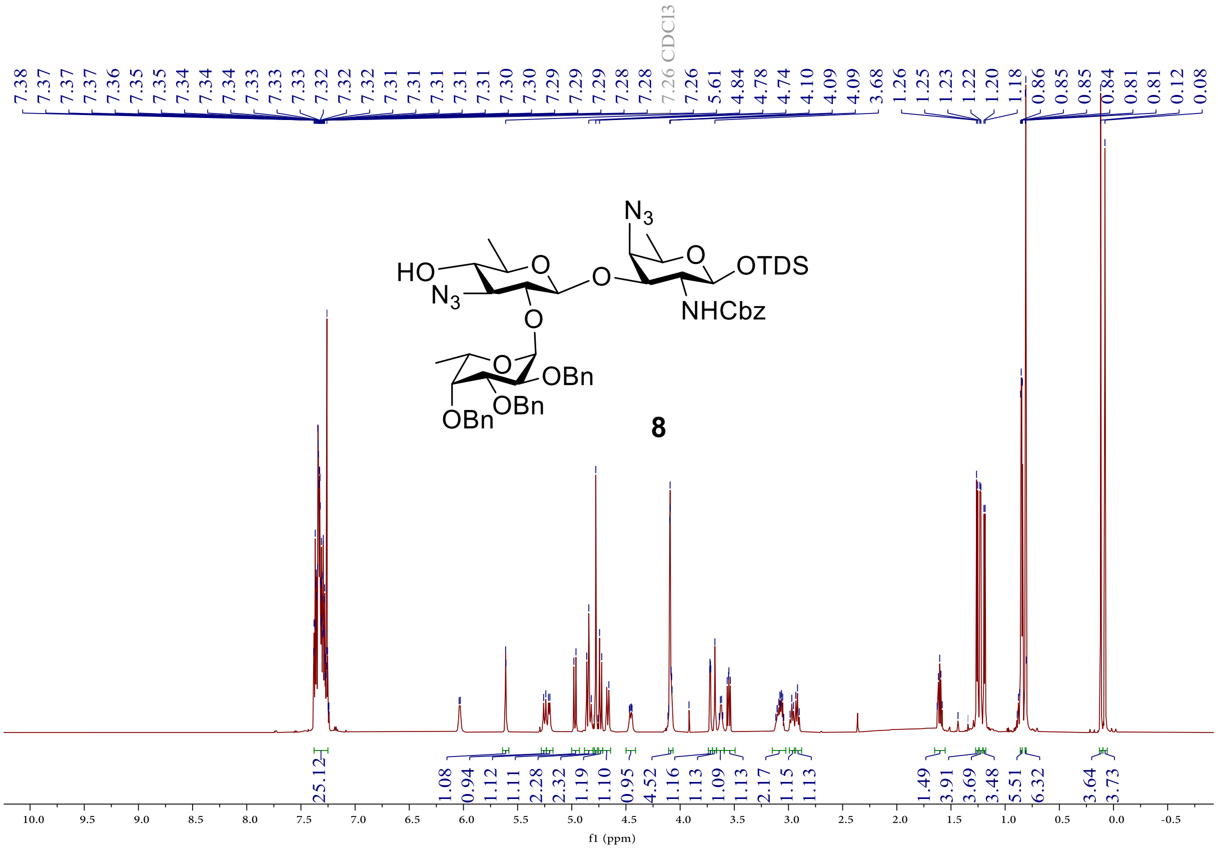
7.38 7.37 7.37 7.37 7.36 7.35 7.35 7.34 7.34 7.34 7.33 7.33 7.33 7.32 7.32 7.32 7.31 7.31 7.31 7.31 7.31 7.30 7.30 7.29 7.29 7.29 7.28 7.28 7.26 CDCl3 7.26 5.61 4.84 4.78 4.74 4.10 4.09 4.09 3.68 1.26 1.25 1.23 1.22 1.20 1.18 0.86 0.85 0.85 0.84 0.81 0.81 0.12 0.08
HO
N3
O
O
N3
O
OTDS
NHCbz
O
O
OBn
OBn
OBn
8
25.12 1.08 0.94 1.12 1.11 2.28 2.32 1.19 1.10 0.95 4.52 1.16 1.13 1.09 1.13 2.17 1.15 1.13 1.49 3.91 3.69 3.48 5.51 6.32 3.64 3.73
f1 (ppm)

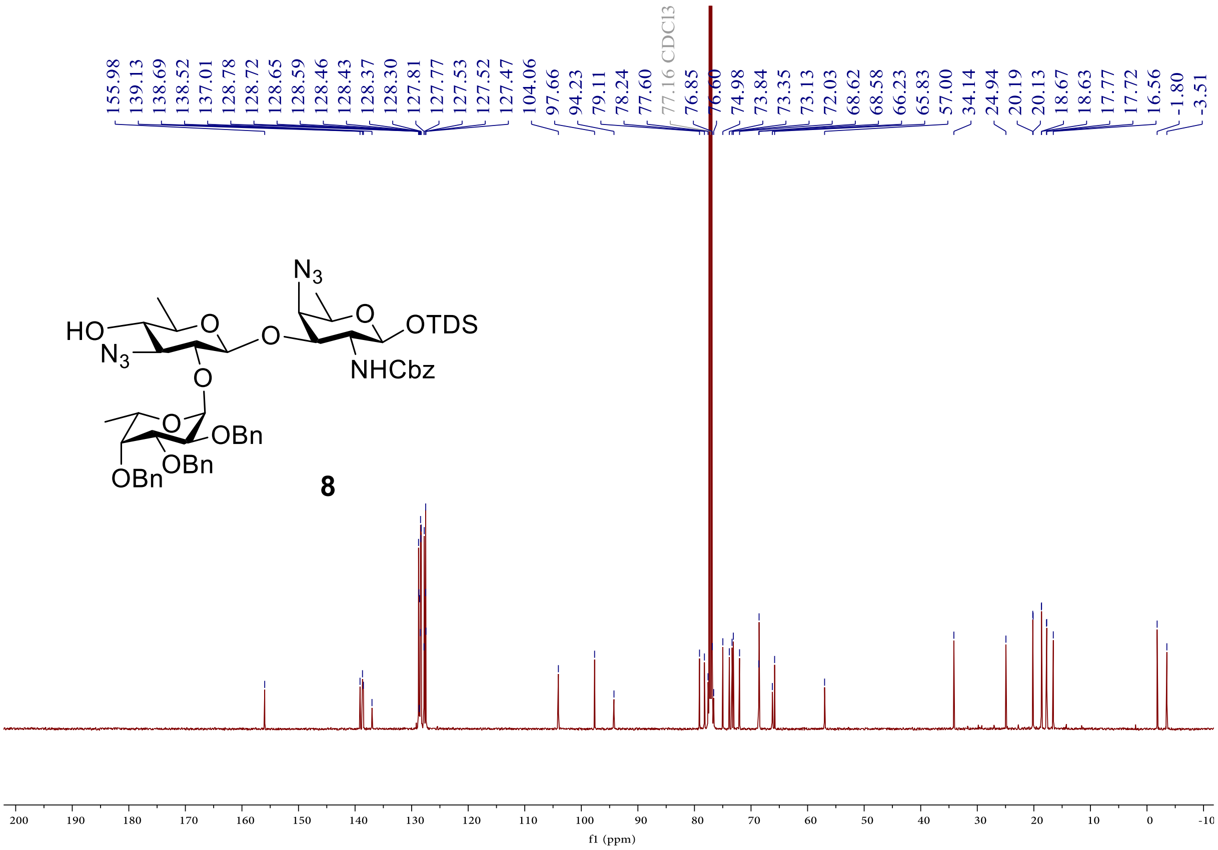
155.98 139.13 138.69 138.52 137.01 128.78 128.72 128.65 128.59 128.46 128.43 128.37 128.30 127.81 127.77 127.53 127.52 127.47 104.06 97.66 94.23 79.11 78.24 77.60 77.16 CDCl3 76.85 76.60 74.98 73.84 73.35 73.13 72.03 68.62 68.58 66.23 65.83 57.00 34.14 24.94 20.19 20.13 18.67 18.63 17.77 17.72 16.56 -1.80 -3.51
HO
N3
O
O
N3
O
OTDS
NHCbz
O
O
OBn
OBn
OBn
8
f1 (ppm)

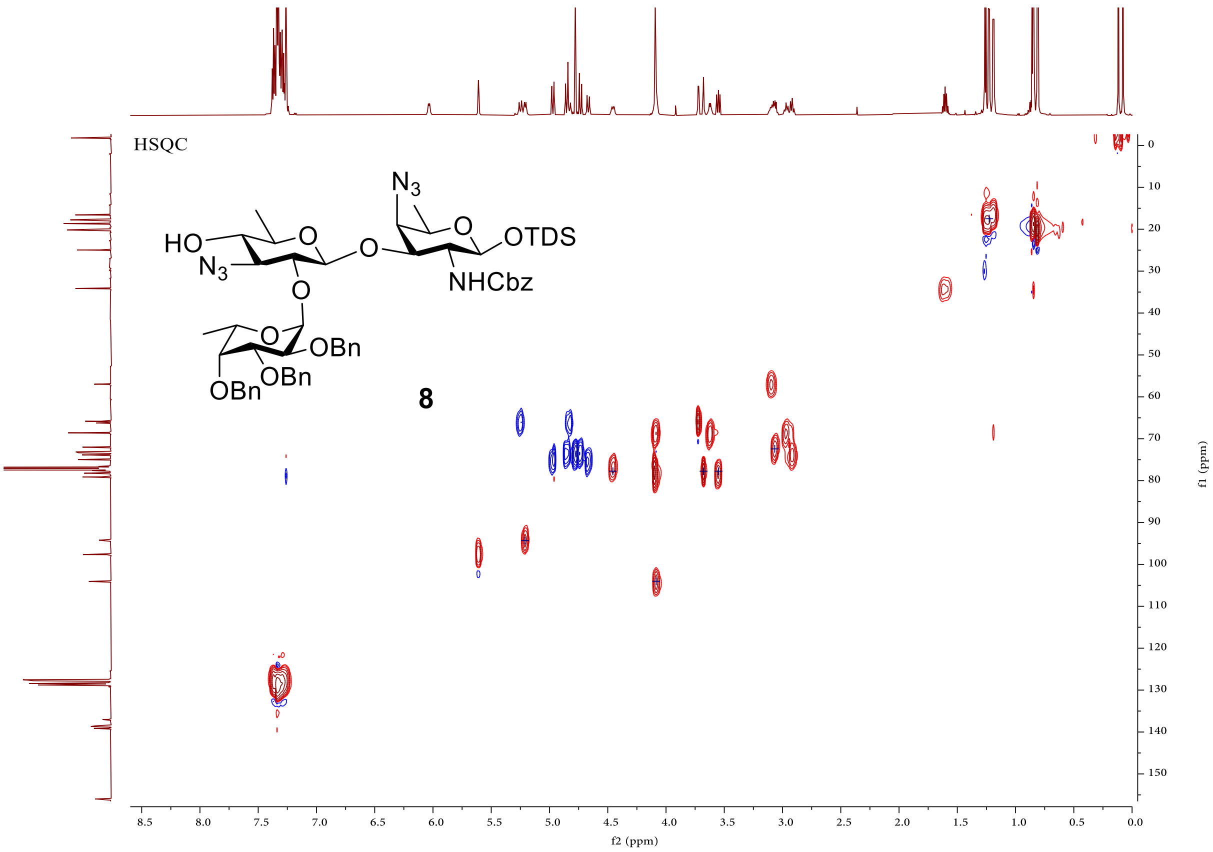

HSQC
HO
N3
O
O
N3
O
OTDS
NHCbz
O
OBn
OBn
OBn
8
f2 (ppm)
f1 (ppm)

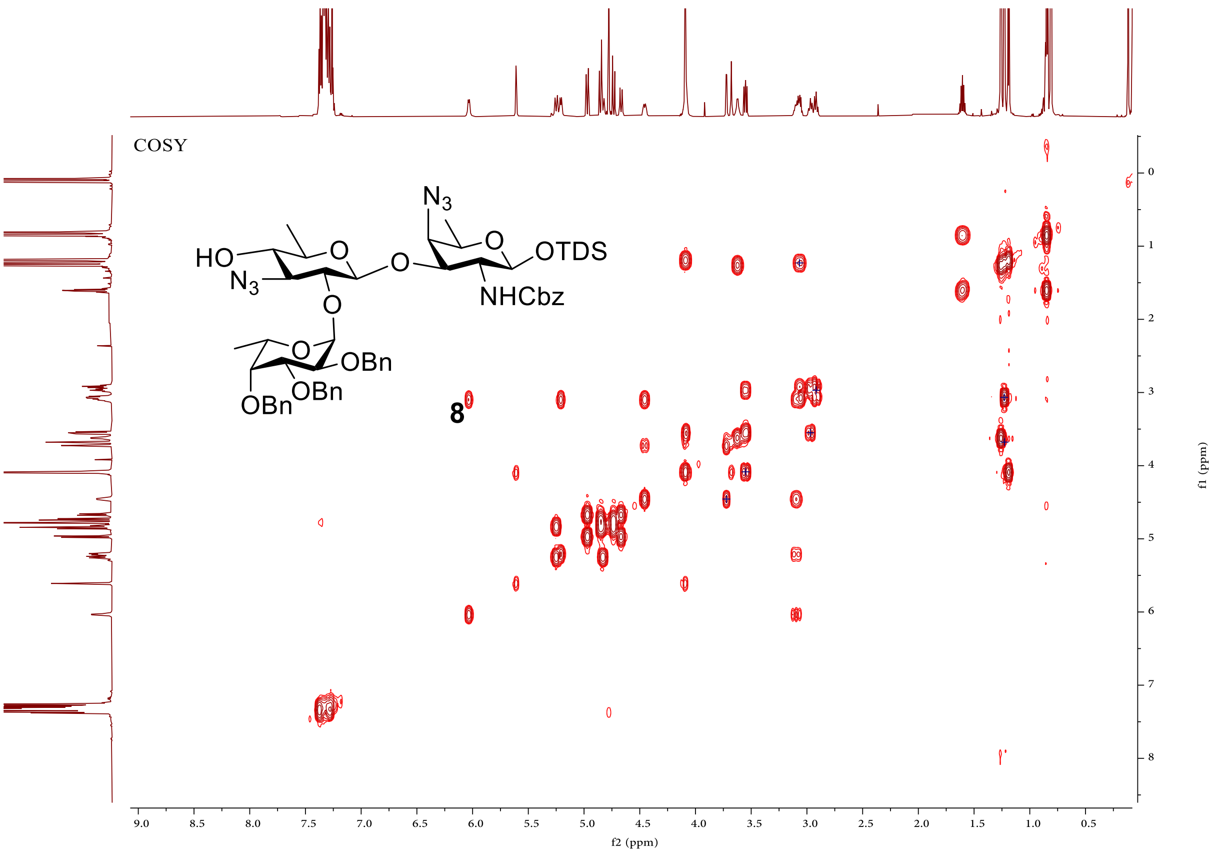

COSY
HO
N3
O
O
N3
O
OTDS
NHCbz
O
OBn
OBn
OBn
8
f2 (ppm)
f1 (ppm)

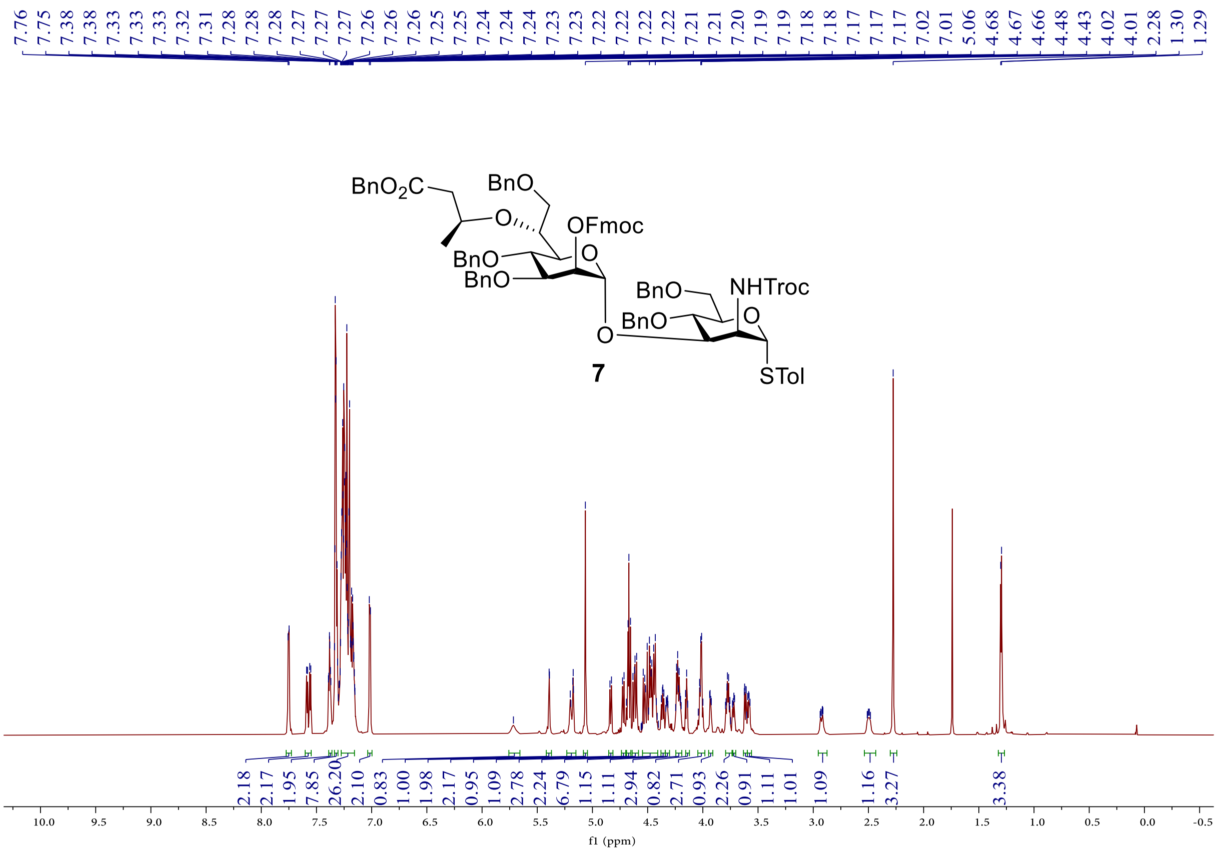
7.76 7.75 7.38 7.38 7.33 7.33 7.33 7.32 7.31 7.28 7.28 7.28 7.27 7.27 7.27 7.26 7.26 7.26 7.25 7.25 7.24 7.24 7.24 7.23 7.23 7.22 7.22 7.22 7.22 7.21 7.21 7.20 7.19 7.19 7.18 7.18 7.17 7.17 7.17 7.02 7.01 5.06 4.68 4.67 4.66 4.48 4.43 4.02 4.01 2.28 1.30 1.29
BnO2C
BnO
OFmoc
BnO
BnO
O
O
BnO
NHTroc
BnO
O
O
7
STol
2.18 2.17 1.95 7.85 26.20 2.10 0.83 1.00 1.98 2.17 0.95 1.09 2.78 2.24 6.79 1.15 1.11 2.94 0.82 2.71 0.93 2.26 0.91 1.11 1.01 1.09 1.16 3.27 3.38
10.0 9.5 9.0 8.5 8.0 7.5 7.0 6.5 6.0 5.5 5.0 4.5 4.0 3.5 3.0 2.5 2.0 1.5 1.0 0.5 0.0 -0.5
f1 (ppm)

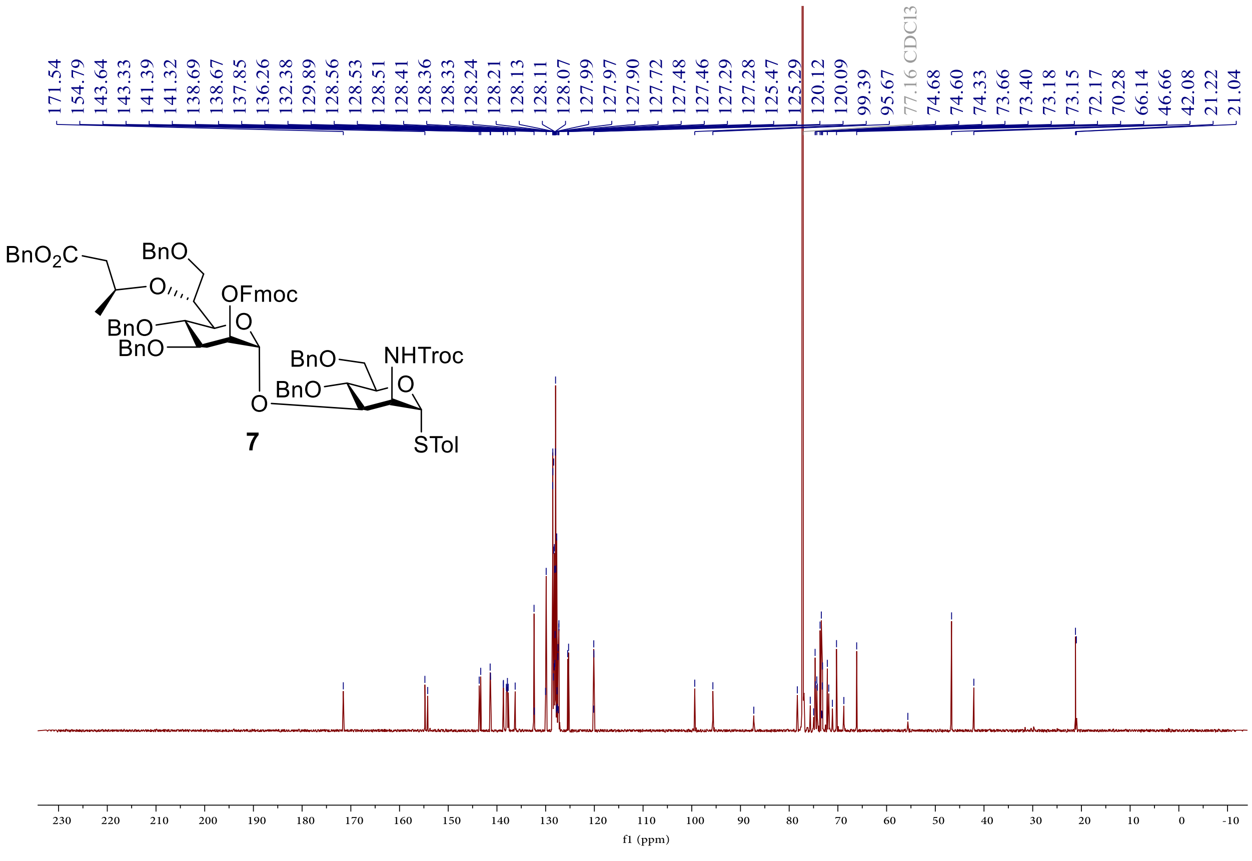
171.54 154.79 143.64 143.33 141.39 141.32 138.69 138.67 137.85 136.26 132.38 129.89 128.56 128.53 128.51 128.41 128.36 128.33 128.24 128.21 128.13 128.11 128.07 127.99 127.97 127.90 127.72 127.48 127.46 127.29 127.28 125.47 125.29 120.12 120.09 99.39 95.67 77.16 CDCl3 74.68 74.60 74.33 73.66 73.40 73.18 73.15 72.17 70.28 66.14 46.66 42.08 21.22 21.04
BnO2C
BnO
OFmoc
BnO
BnO
O
O
BnO
NHTroc
BnO
O
O
7
STol
230 220 210 200 190 180 170 160 150 140 130 120 110 100 90 80 70 60 50 40 30 20 10 0 -10
f1 (ppm)

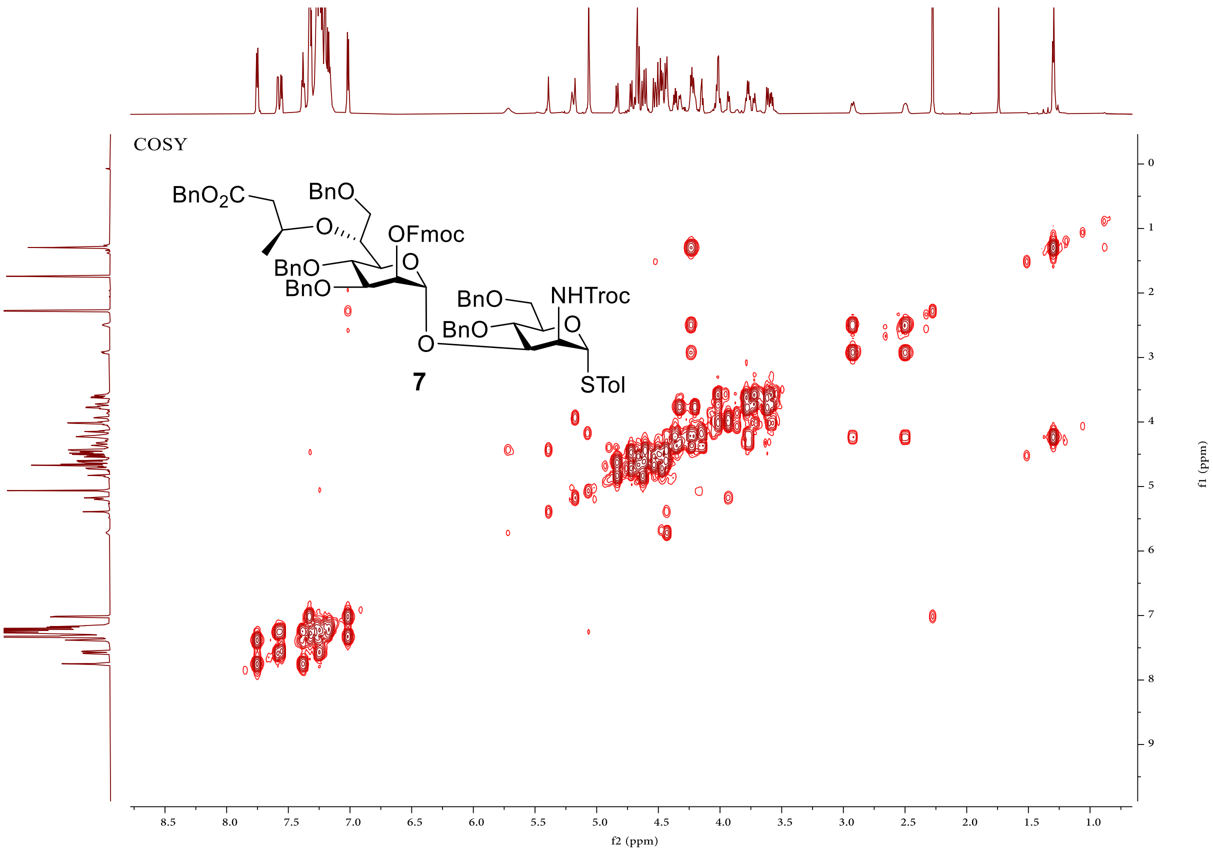
COSY
BnO$_2$C
BnO
OFmoc
O
BnO
BnO
BnO
NHTroc
BnO
O
O
7
STol
f2 (ppm)
f1 (ppm)

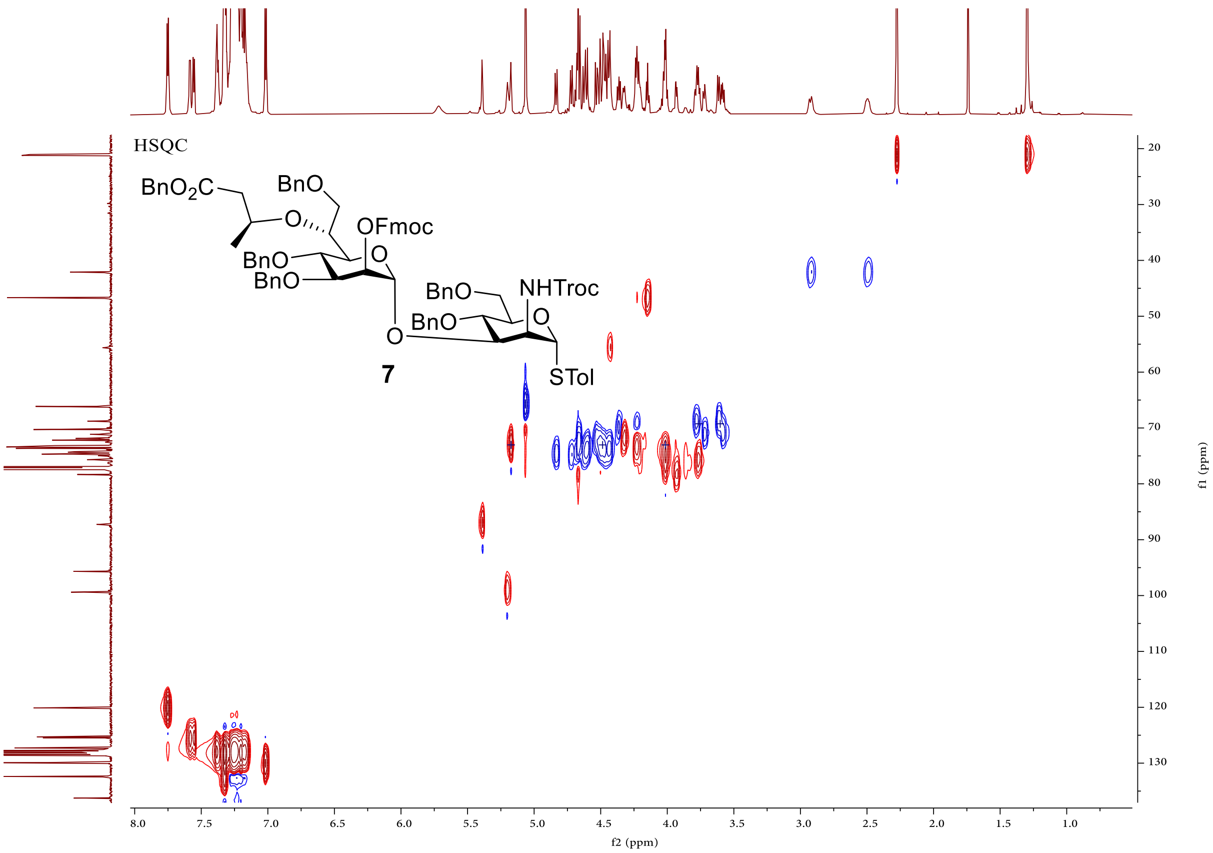
HSQC
BnO$_2$C
BnO
OFmoc
O
BnO
BnO
BnO
NHTroc
BnO
O
O
7
STol
f2 (ppm)
f1 (ppm)

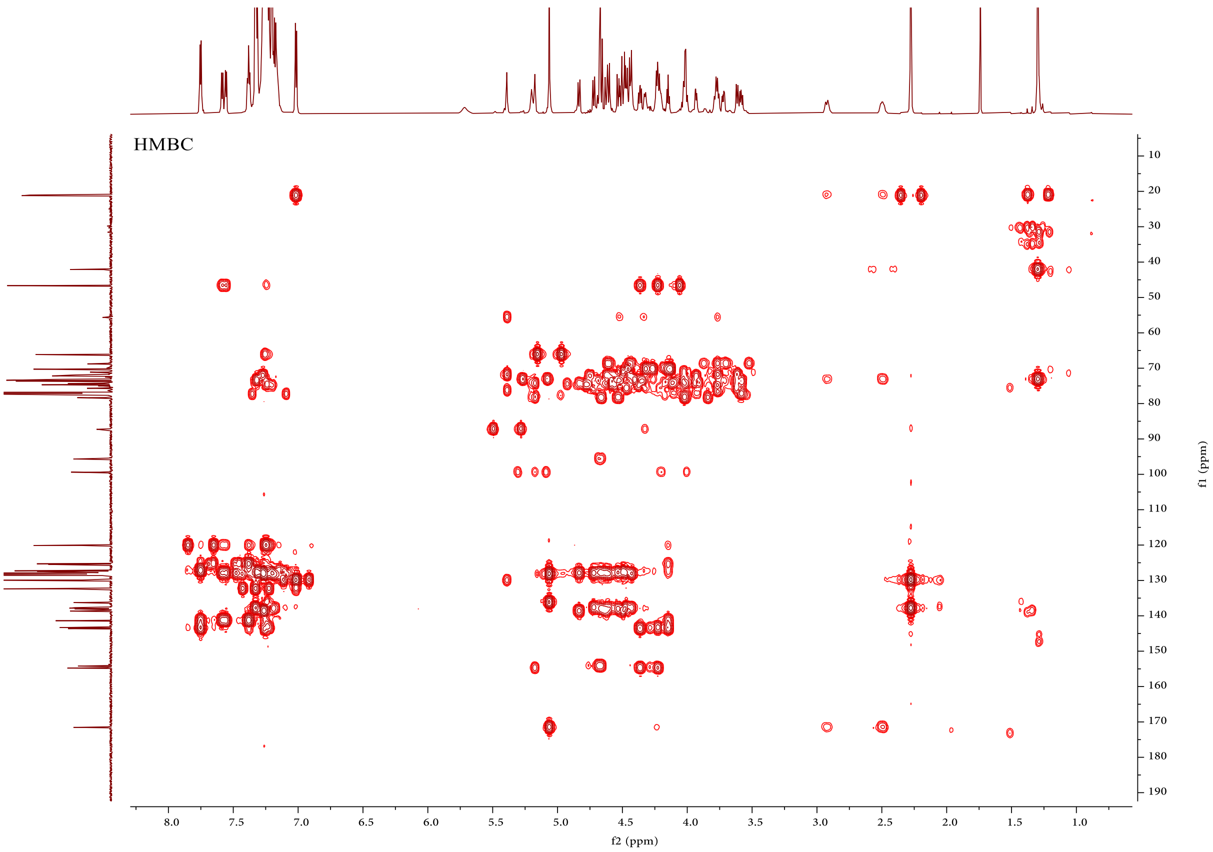
HMBC
f2 (ppm)
f1 (ppm)

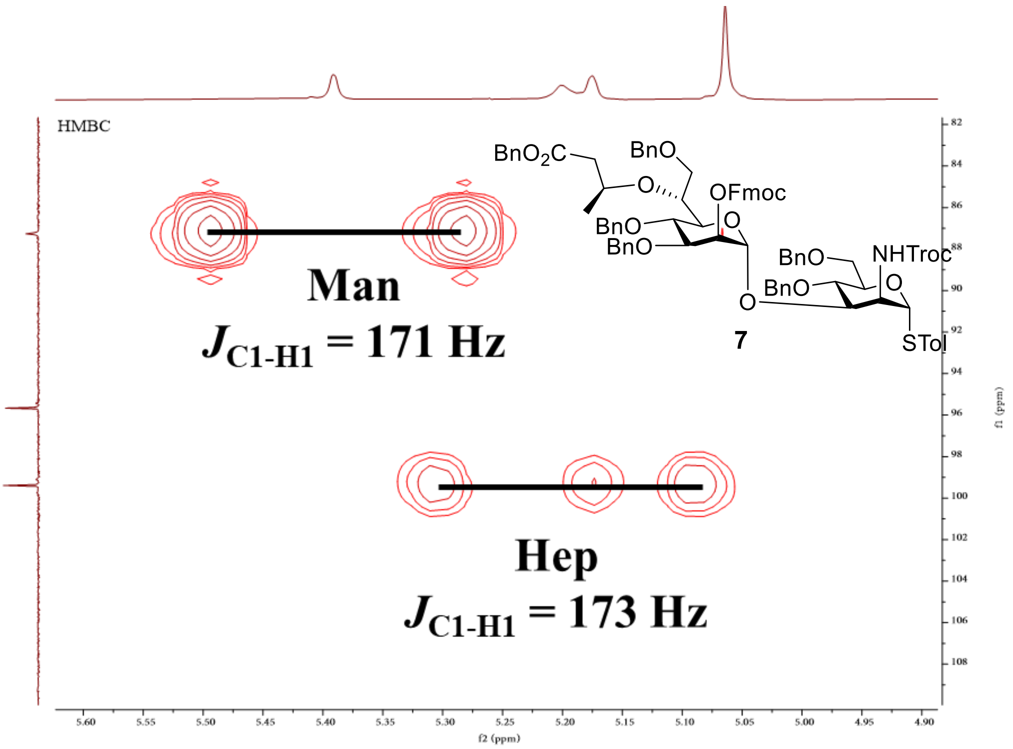
HMBC
Man
J_{C1-H1} = 171 Hz
Hep
J_{C1-H1} = 173 Hz
BnO2C
BnO
OFmoc
BnO
BnO
O
O
BnO
NHTroc
BnO
O
STol
7
f2 (ppm)
f1 (ppm)

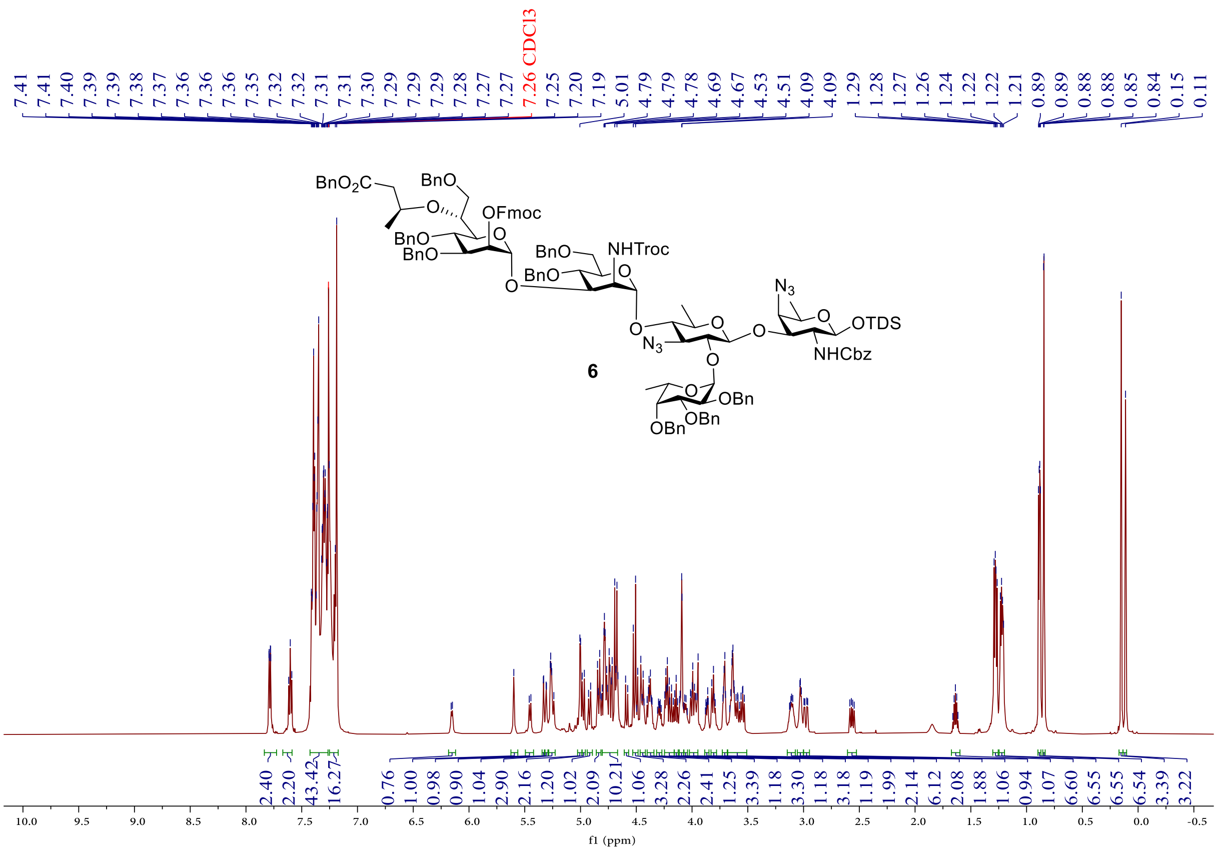
7.41 7.41 7.40 7.39 7.39 7.38 7.37 7.36 7.36 7.36 7.35 7.32 7.31 7.31 7.30 7.29 7.29 7.29 7.28 7.27 7.27 7.26 CDCl3 7.25 7.20 7.19 5.01 4.79 4.79 4.78 4.69 4.67 4.53 4.51 4.09 4.09 1.29 1.28 1.27 1.26 1.24 1.22 1.22 1.21 0.89 0.89 0.88 0.88 0.85 0.84 0.15 0.11
BnO2C
BnO
OFmoc
BnO
BnO
BnO
NHTroc
BnO
N3
OTDS
N3
NHCbz
6
OBn
OBn
OBn
2.40 2.20 43.42 16.27 0.76 1.00 0.98 0.90 1.04 2.90 2.16 1.20 1.02 2.09 10.21 1.06 3.28 2.26 2.41 1.25 3.39 1.18 3.30 1.18 3.18 1.19 1.99 2.14 6.12 2.08 1.88 1.06 0.94 1.07 6.60 6.55 6.55 6.54 3.39 3.22
10.0 9.5 9.0 8.5 8.0 7.5 7.0 6.5 6.0 5.5 5.0 4.5 4.0 3.5 3.0 2.5 2.0 1.5 1.0 0.5 0.0 -0.5
f1 (ppm)

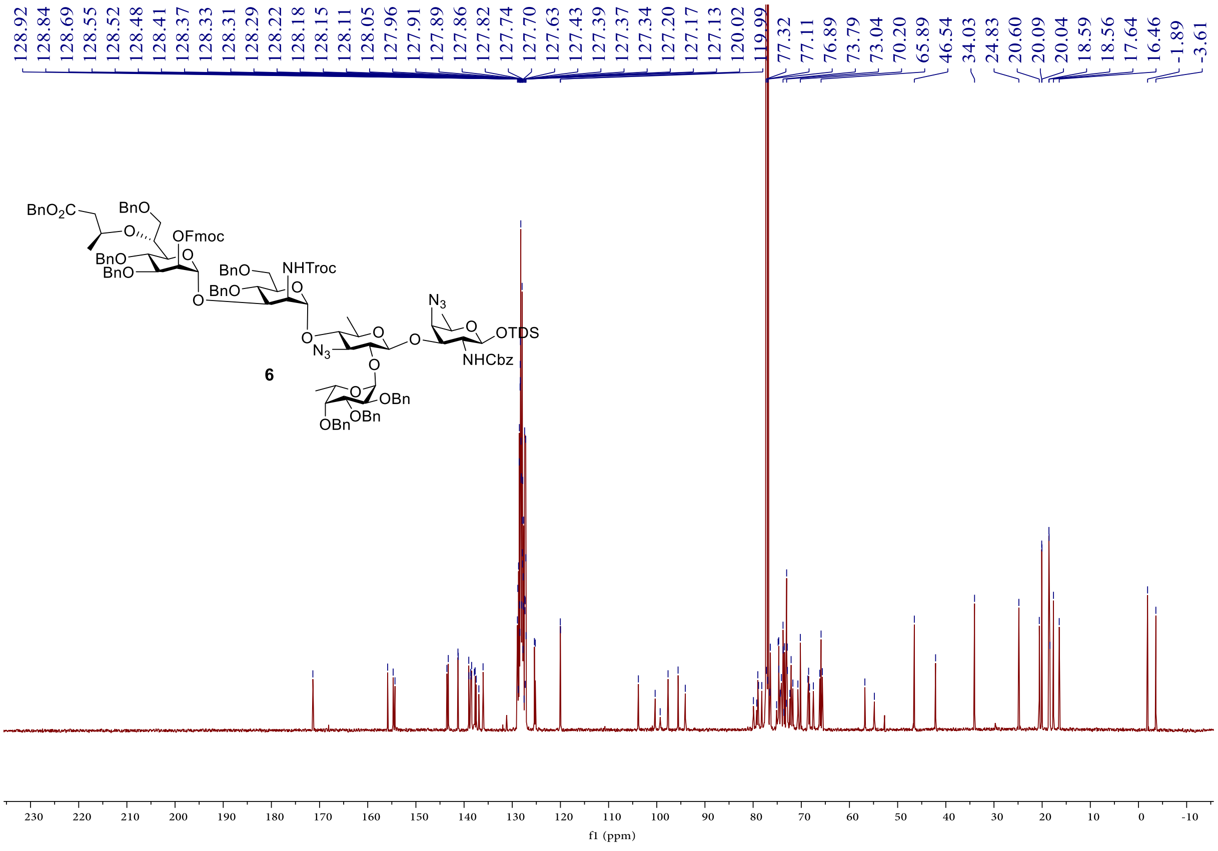
128.92 128.84 128.69 128.55 128.52 128.48 128.41 128.37 128.33 128.31 128.29 128.22 128.18 128.15 128.11 128.05 127.96 127.91 127.89 127.86 127.82 127.74 127.70 127.63 127.43 127.39 127.37 127.34 127.20 127.17 127.13 120.02 119.99 77.32 77.11 76.89 73.79 73.04 70.20 65.89 46.54 34.03 24.83 20.60 20.09 20.04 18.59 18.56 17.64 16.46 -1.89 -3.61
BnO2C
BnO
OFmoc
BnO
BnO
BnO
NHTroc
BnO
N3
OTDS
N3
NHCbz
6
OBn
OBn
OBn
230 220 210 200 190 180 170 160 150 140 130 120 110 100 90 80 70 60 50 40 30 20 10 0 -10
f1 (ppm)

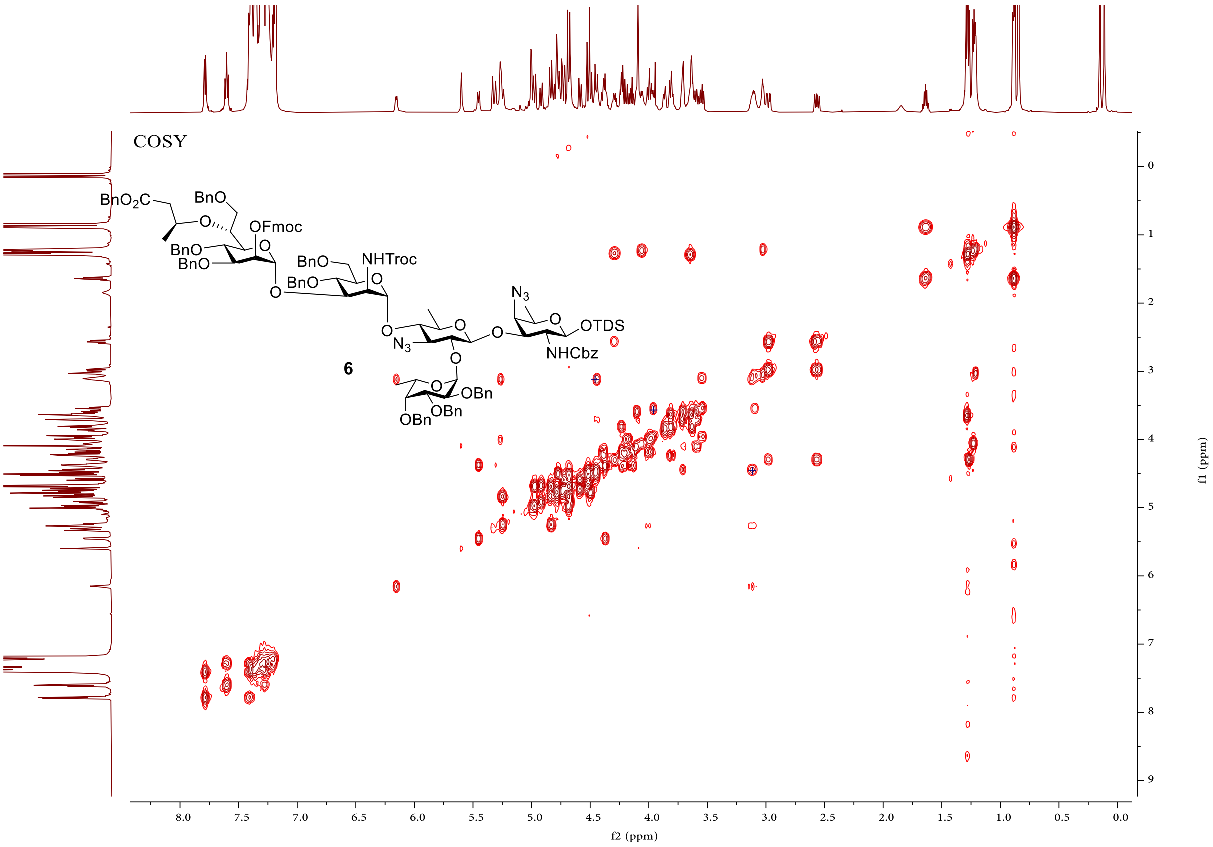
COSY
BnO2C
BnO
OFmoc
NHTroc
N3
OTDS
NHCbz
6
OBn
f2 (ppm)
f1 (ppm)

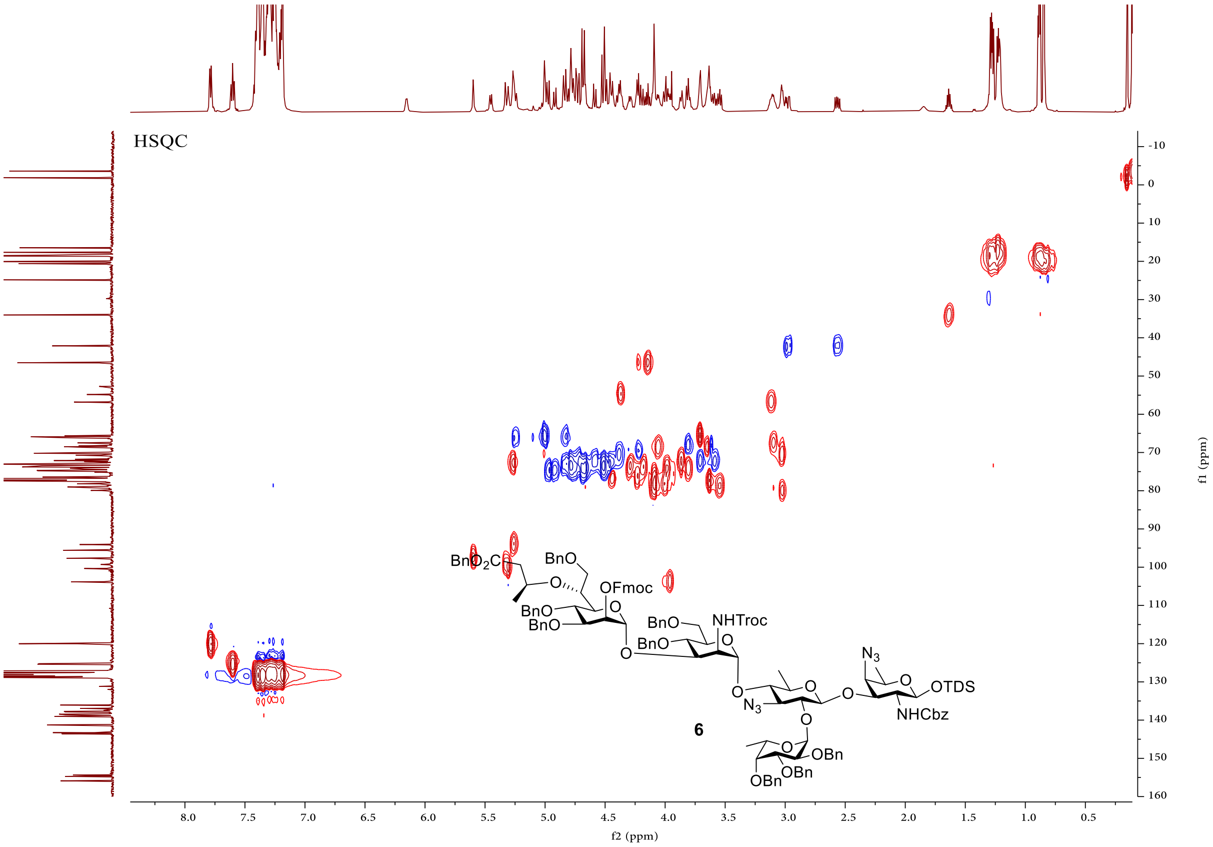
HSQC
BnO2C
BnO
OFmoc
NHTroc
N3
OTDS
NHCbz
6
OBn
f2 (ppm)
f1 (ppm)

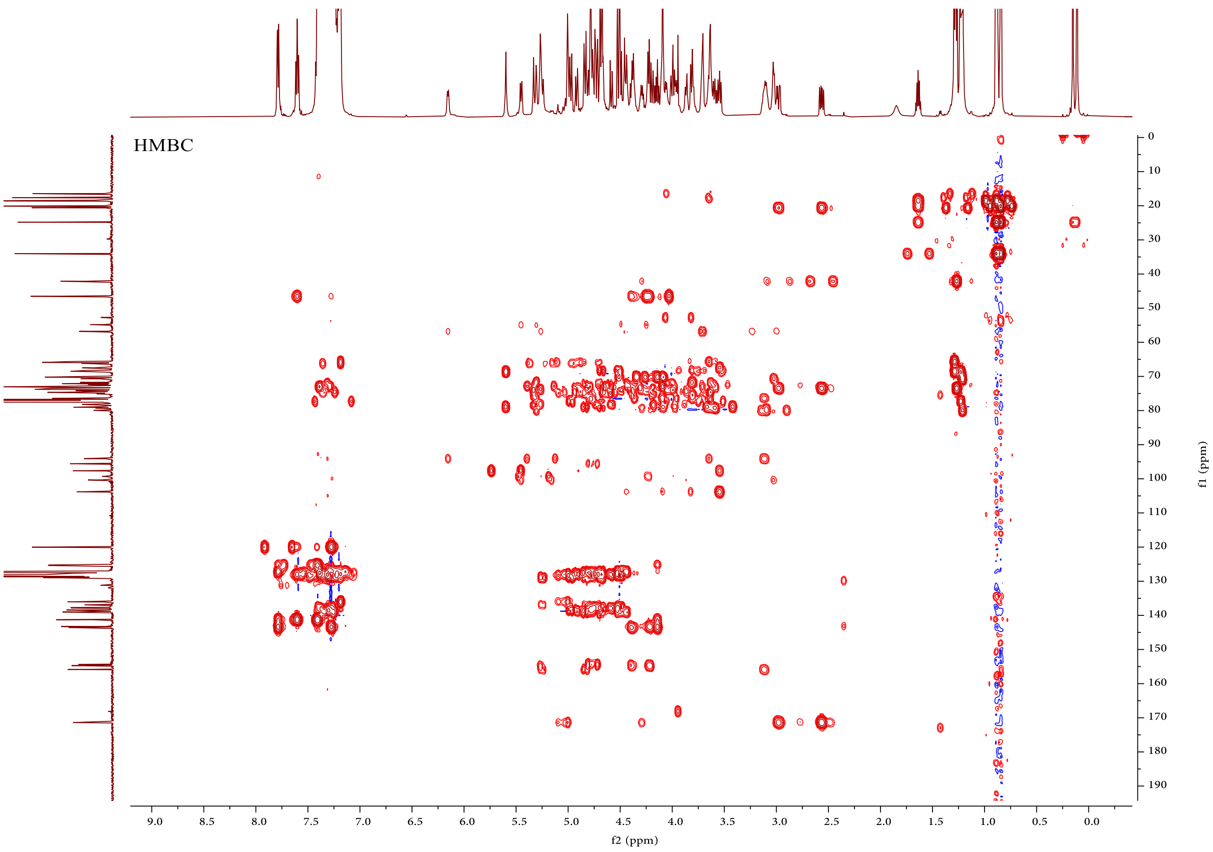
HMBC
f2 (ppm)
f1 (ppm)

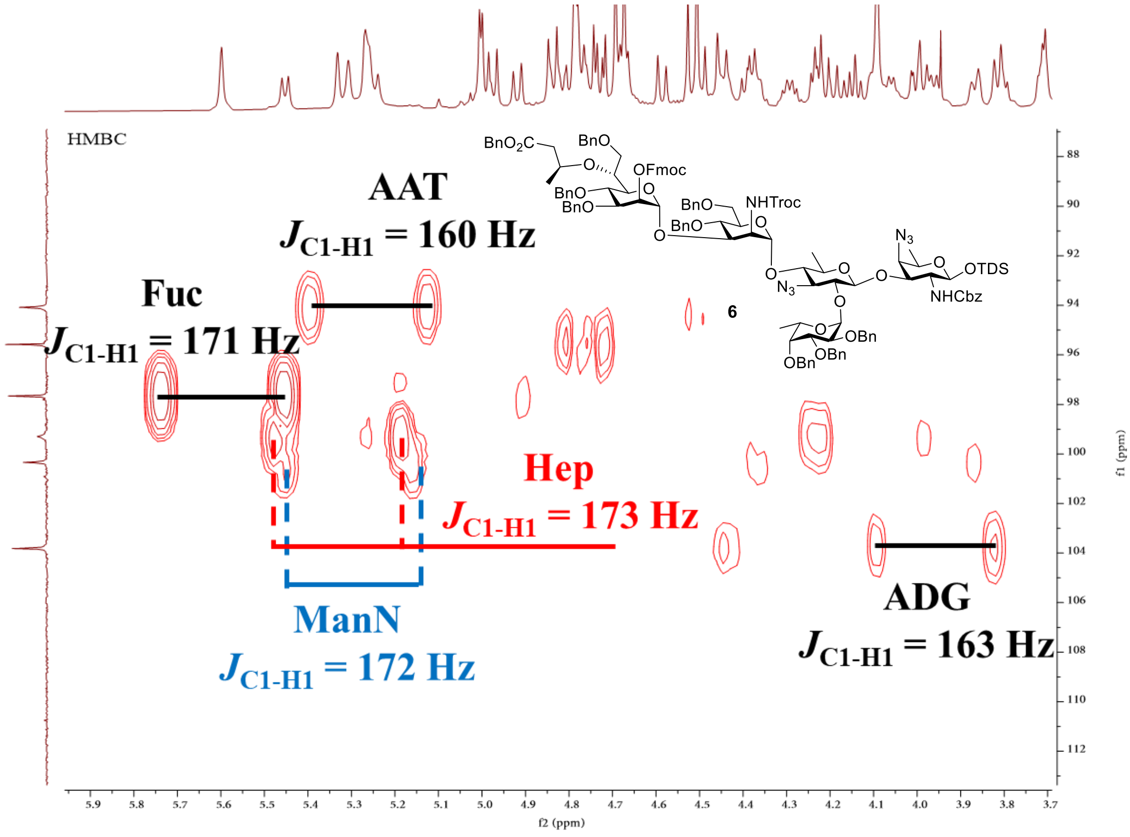
HMBC
AAT
J_{C1-H1} = 160 Hz
Fuc
J_{C1-H1} = 171 Hz
Hep
J_{C1-H1} = 173 Hz
ManN
J_{C1-H1} = 172 Hz
ADG
J_{C1-H1} = 163 Hz
6
f2 (ppm)
f1 (ppm)

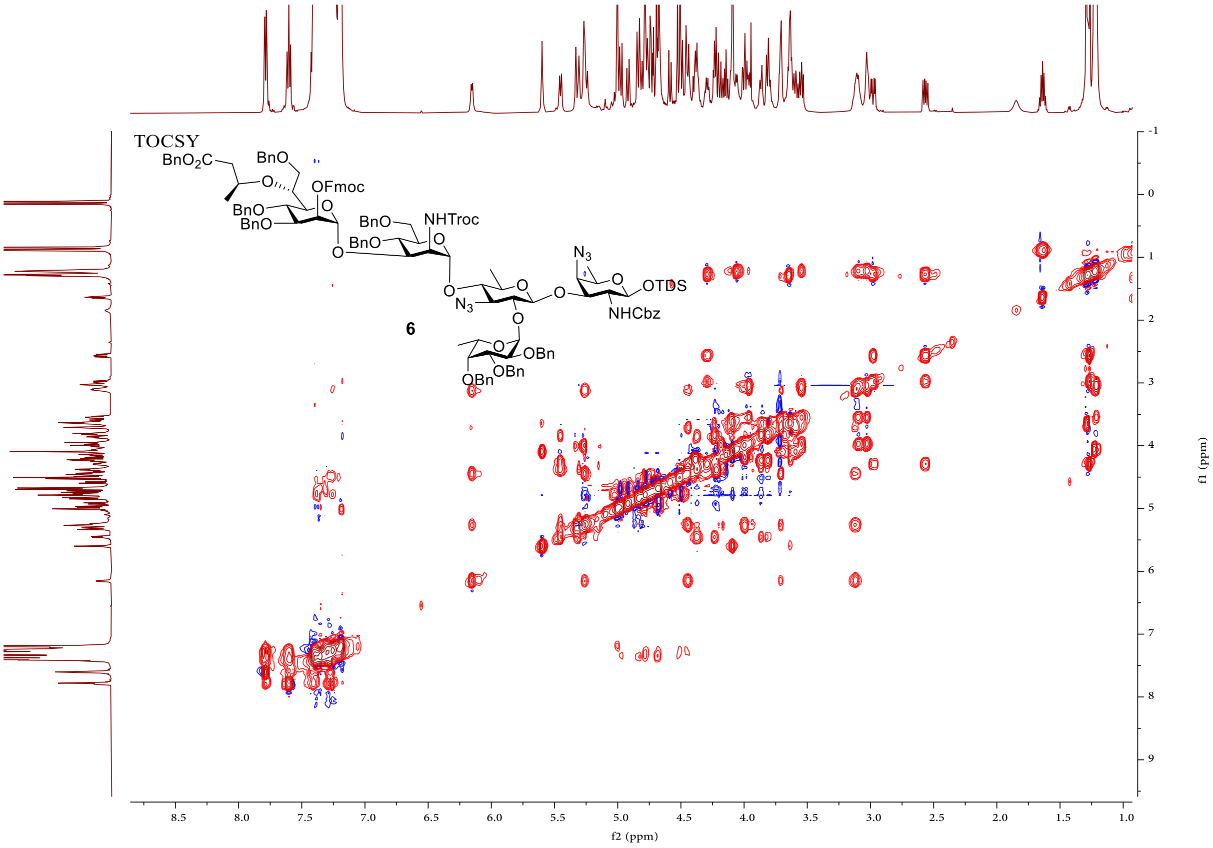

TOCSY
BnO
BnO2C
OFmoc
BnO
BnO
NHTroc
N3
OTDS
NHCbz
6
OBn
OBn
OBn
f2 (ppm)
f1 (ppm)

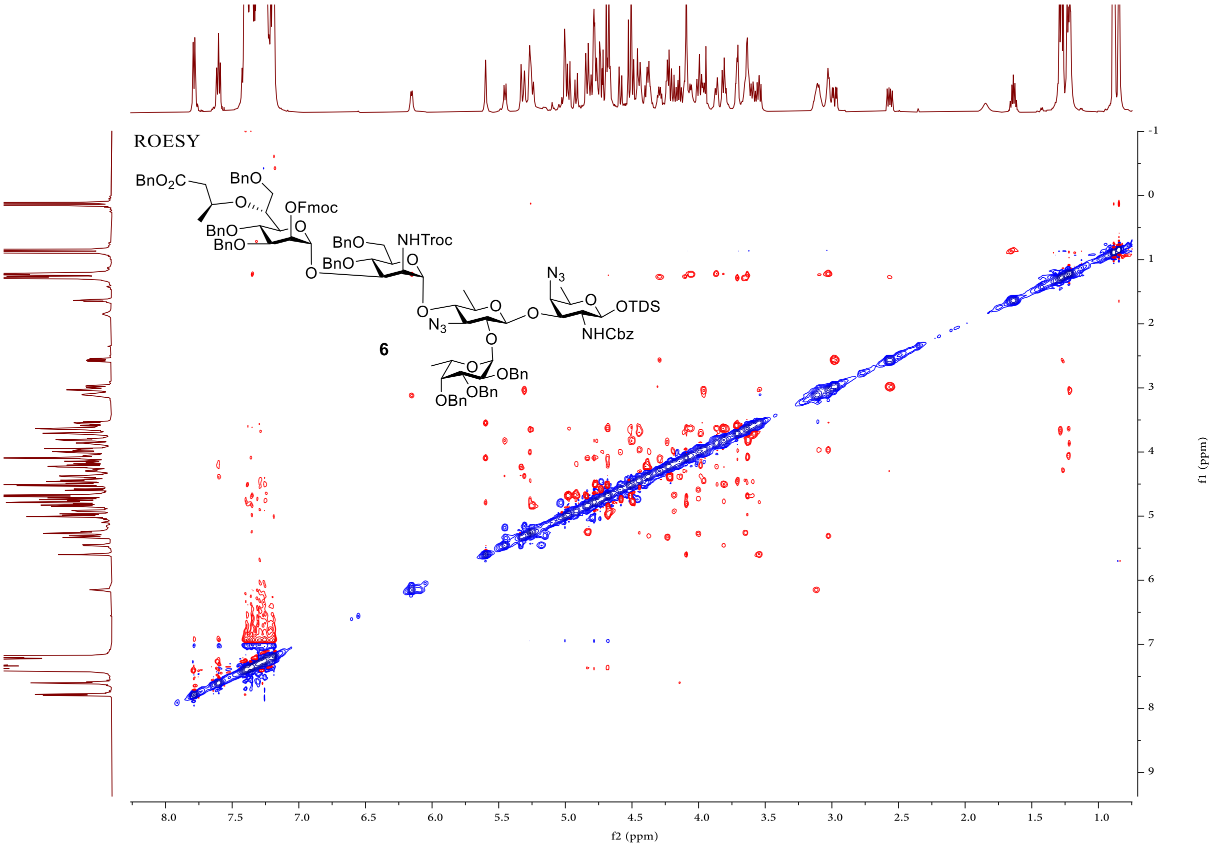

ROESY
BnO
BnO2C
OFmoc
BnO
BnO
NHTroc
N3
OTDS
NHCbz
6
OBn
OBn
OBn
f2 (ppm)
f1 (ppm)

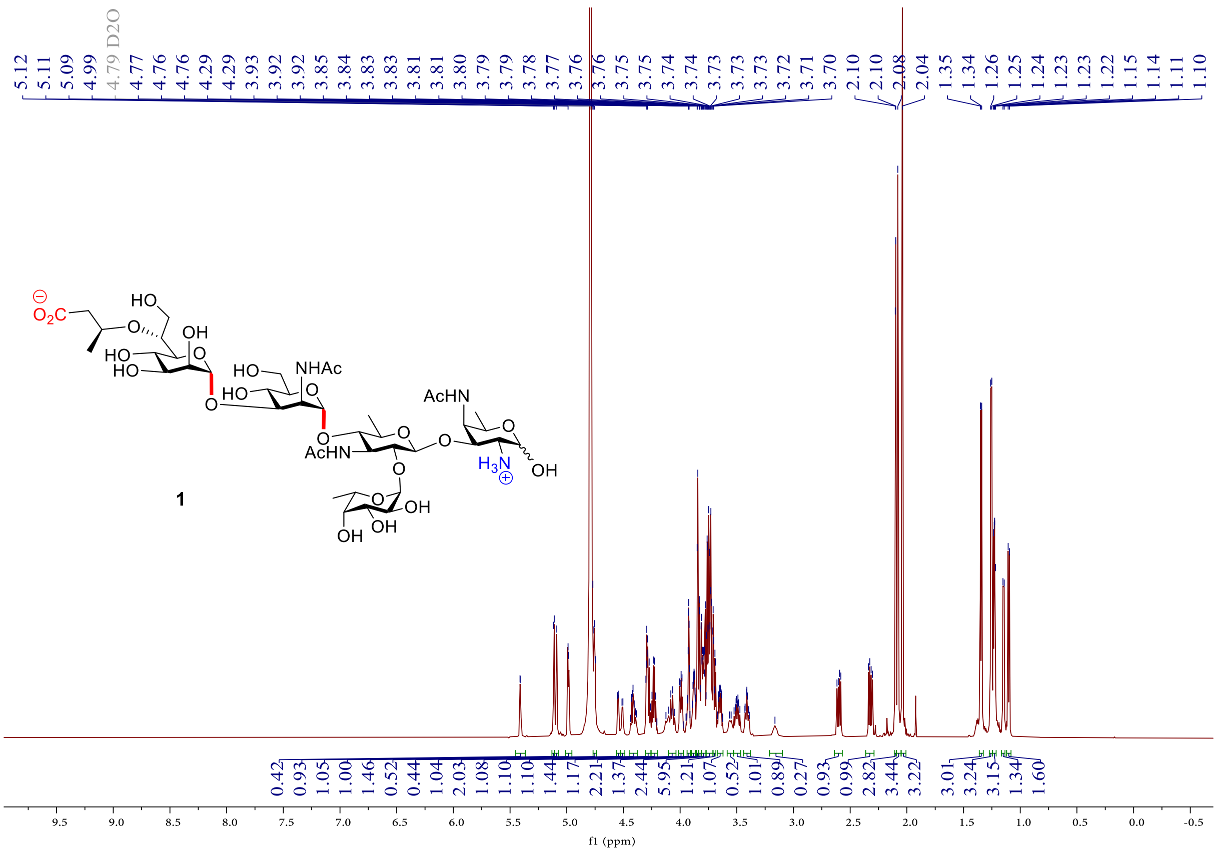

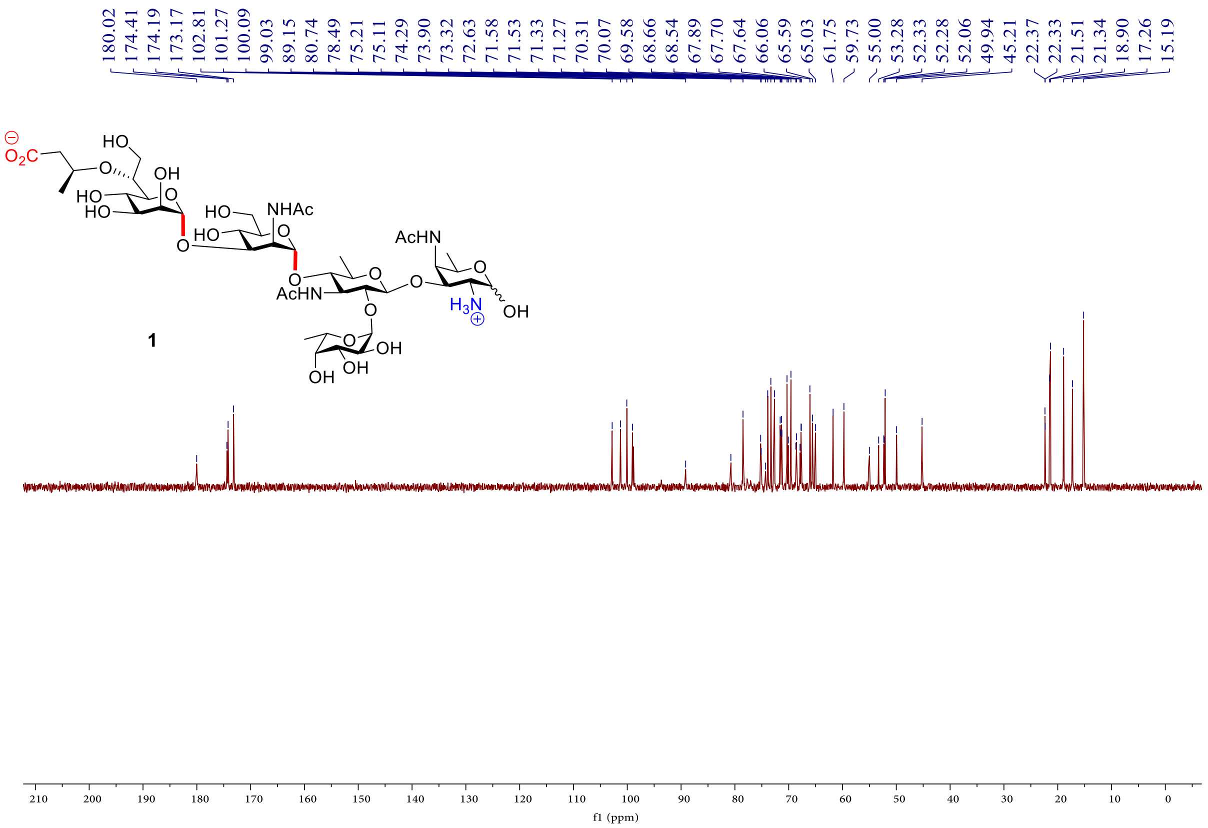

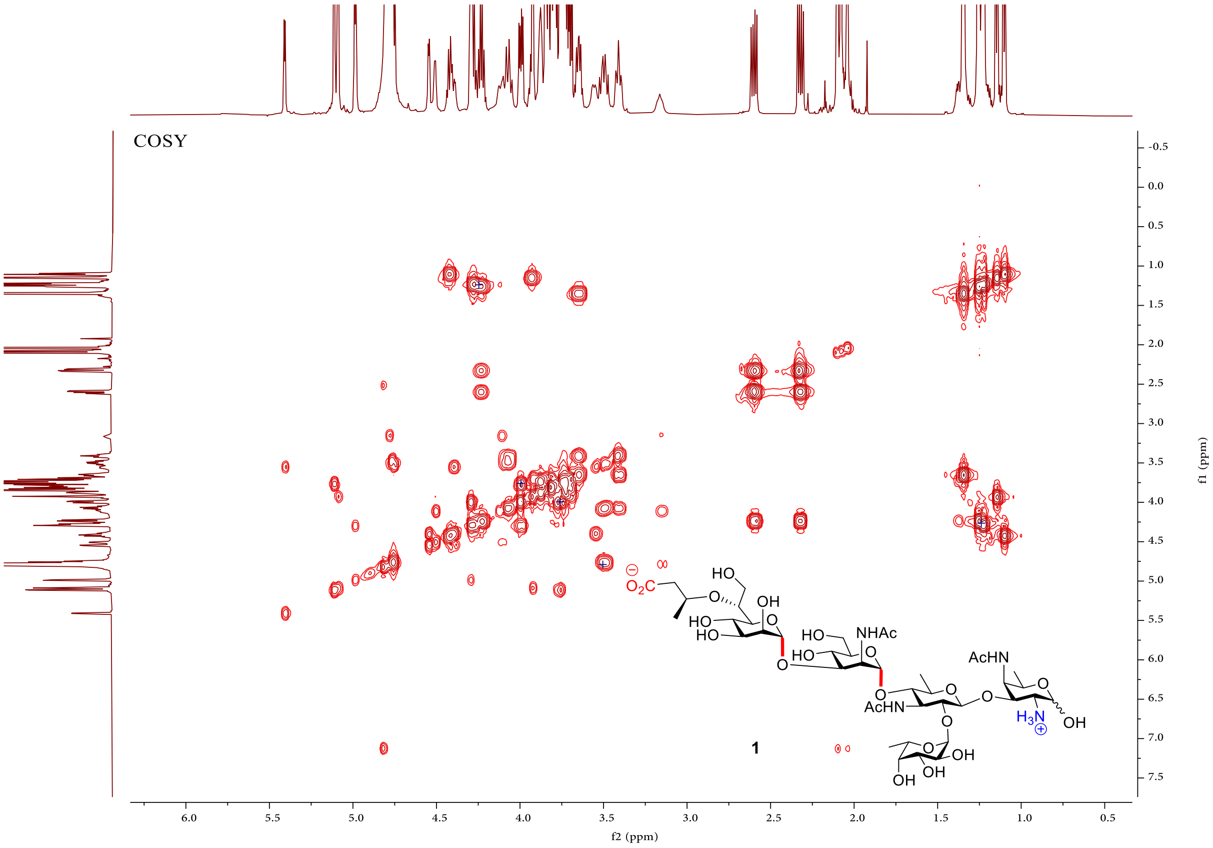
COSY
f2 (ppm)
f1 (ppm)
1

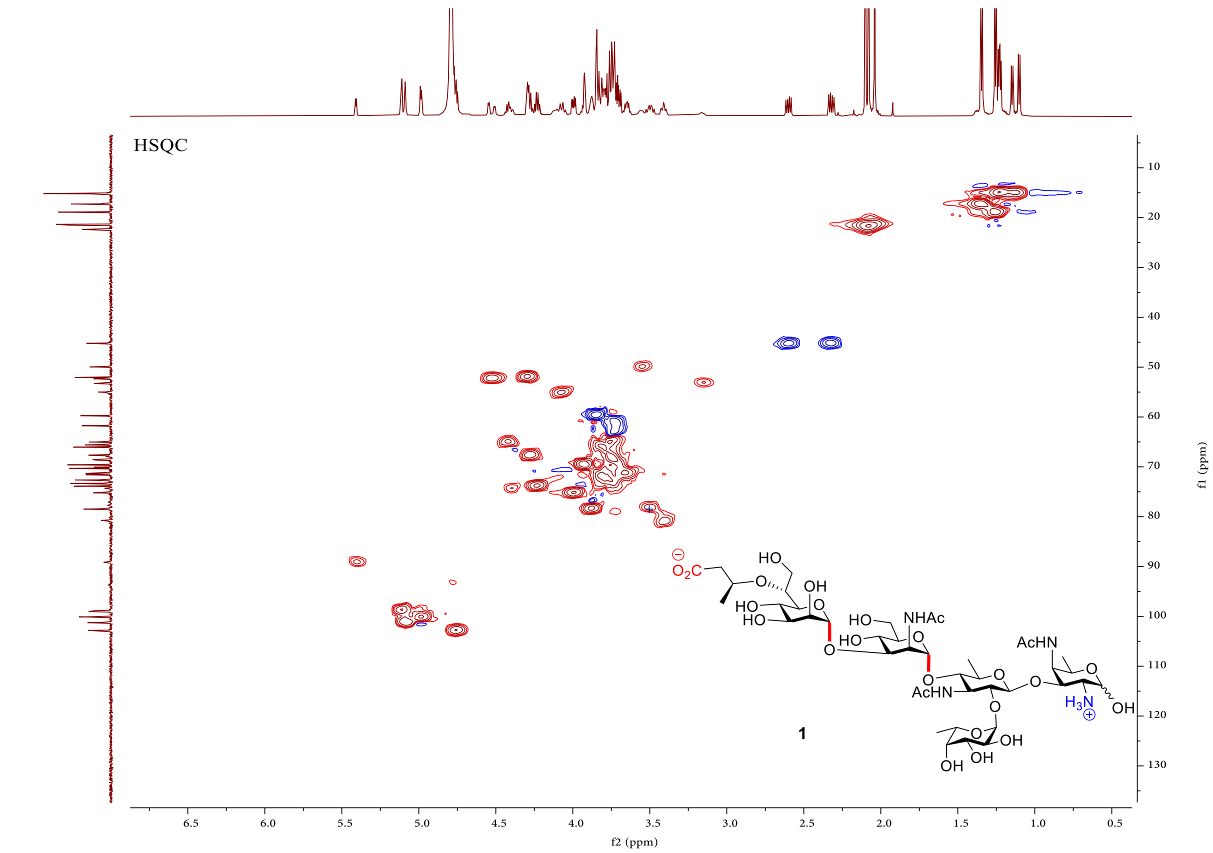
HSQC
f2 (ppm)
f1 (ppm)
1

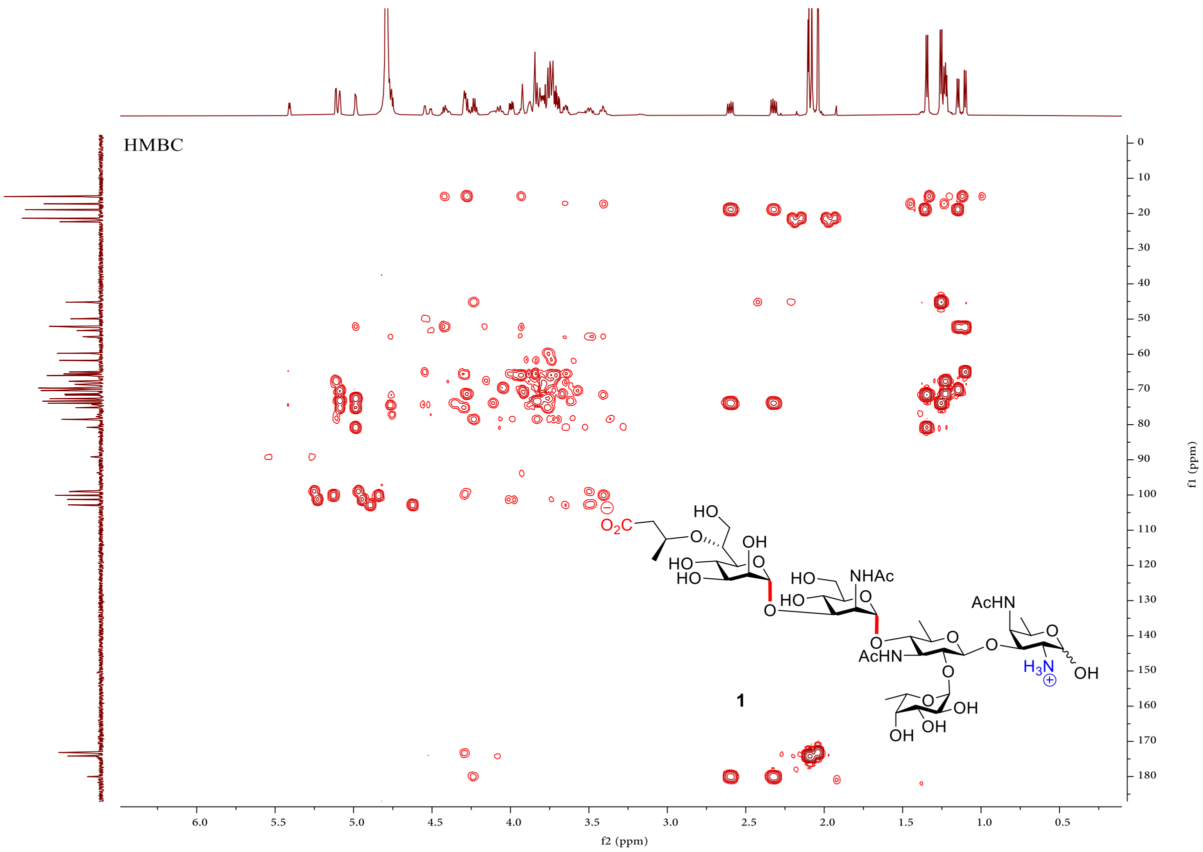
HMBC
f2 (ppm)
f1 (ppm)
1

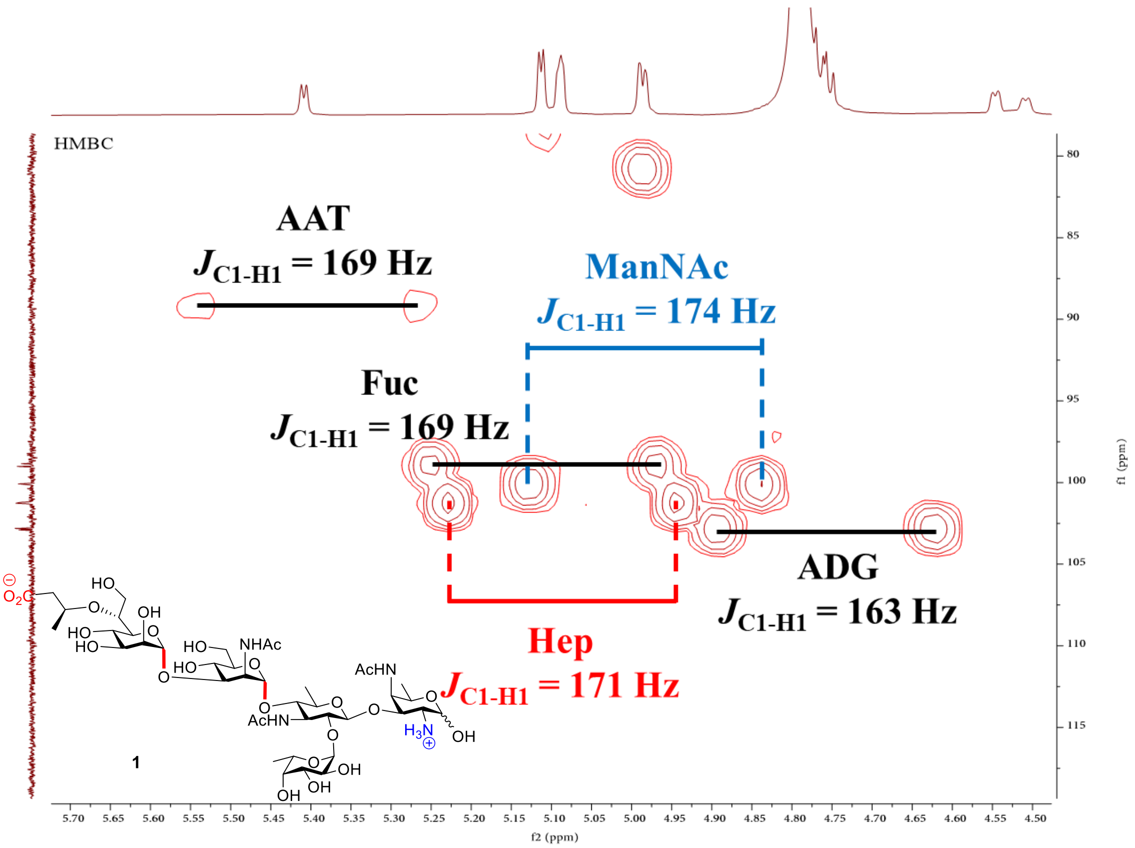
HMBC
AAT
J_{C1-H1} = 169 Hz
ManNAc
J_{C1-H1} = 174 Hz
Fuc
J_{C1-H1} = 169 Hz
ADG
J_{C1-H1} = 163 Hz
Hep
J_{C1-H1} = 171 Hz
1
f2 (ppm)
f1 (ppm)

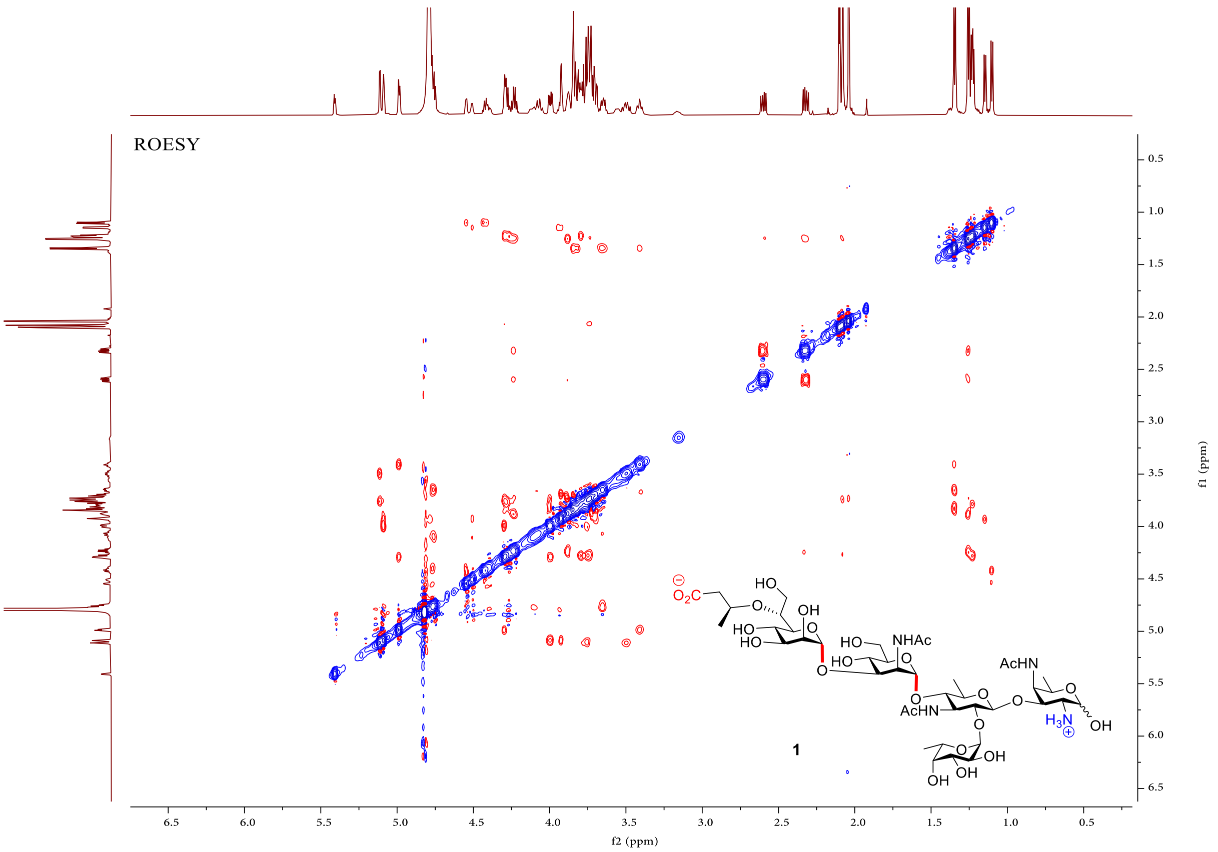
ROESY
f1 (ppm)
f2 (ppm)
HO
OH
HO
HO
O
NHAc
HO
HO
AcHN
AcHN
OH
OH
OH
OH
1

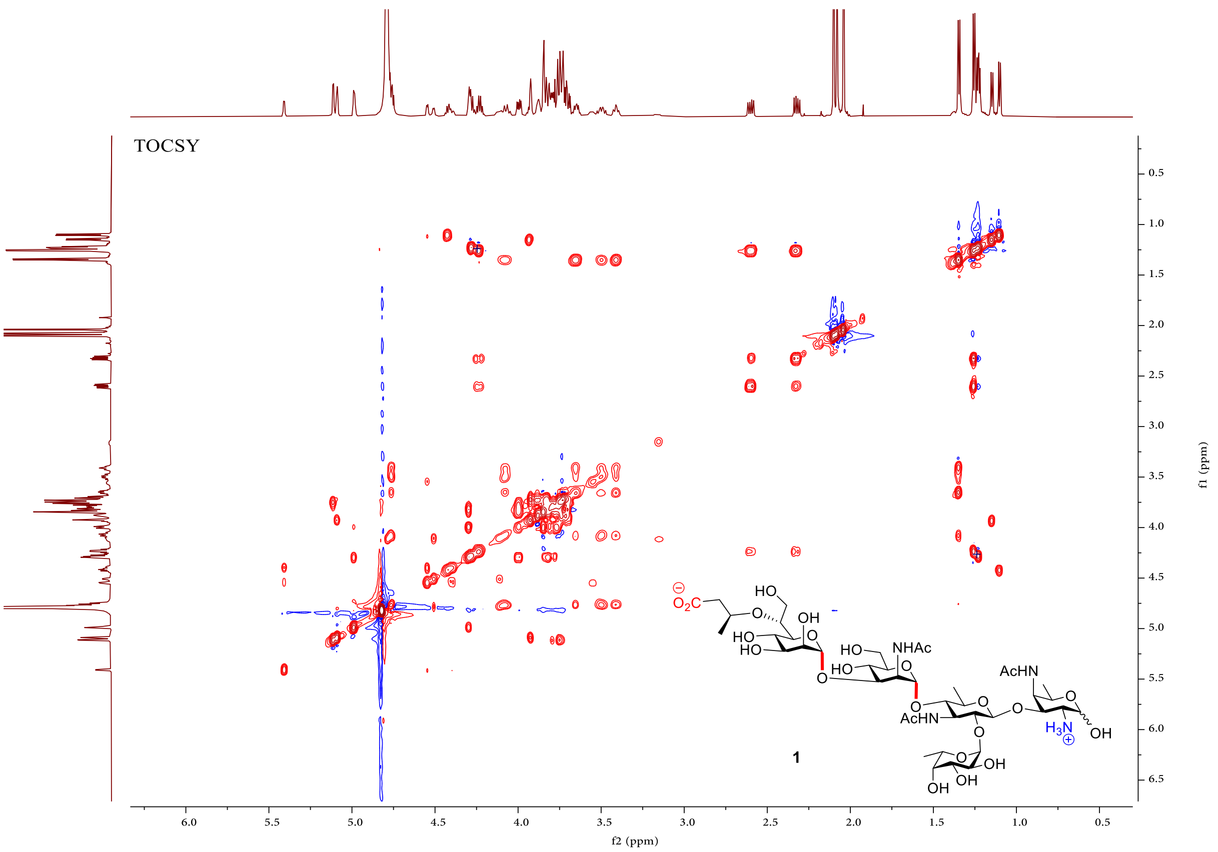
TOCSY
f1 (ppm)
f2 (ppm)
HO
OH
HO
HO
O
NHAc
HO
HO
AcHN
AcHN
OH
OH
OH
OH
1

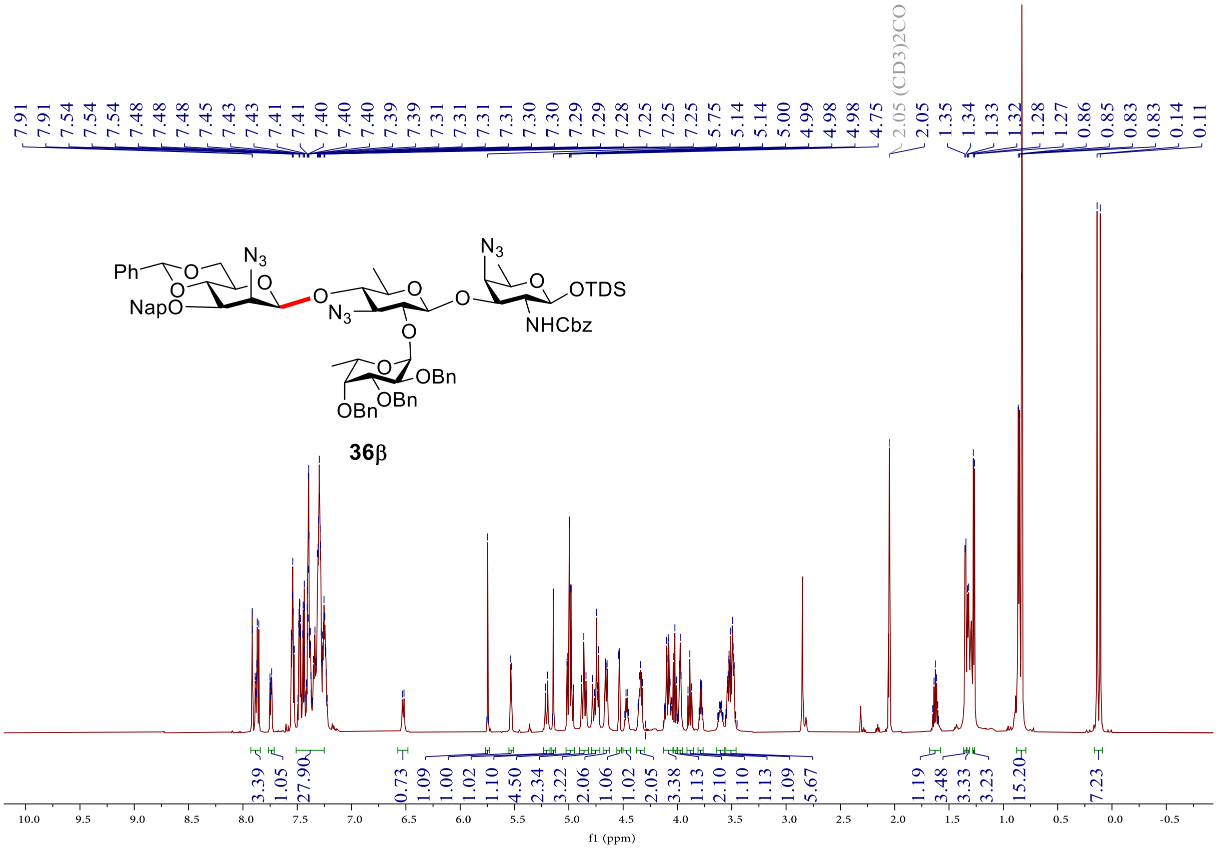
7.91 7.91 7.54 7.54 7.48 7.48 7.48 7.45 7.43 7.43 7.41 7.41 7.40 7.40 7.40 7.39 7.39 7.31 7.31 7.31 7.31 7.30 7.30 7.29 7.29 7.28 7.25 7.25 7.25 5.75 5.14 5.14 5.00 4.99 4.98 4.98 4.75 2.05 (CD3)2CO 2.05 1.35 1.34 1.33 1.32 1.28 1.27 0.86 0.85 0.83 0.14 0.11
Ph
O
O
N3
O
NapO
O
N3
O
O
OTDS
NHCbz
O
O
OBn
OBn
OBn
36β
3.39 1.05 27.90 0.73 1.09 1.00 1.02 1.10 4.50 2.34 3.22 2.06 1.06 1.02 2.05 3.38 1.13 2.10 1.10 1.13 1.09 5.67 1.19 3.48 3.33 3.23 15.20 7.23
10.0 9.5 9.0 8.5 8.0 7.5 7.0 6.5 6.0 5.5 5.0 4.5 4.0 3.5 3.0 2.5 2.0 1.5 1.0 0.5 0.0 -0.5
f1 (ppm)

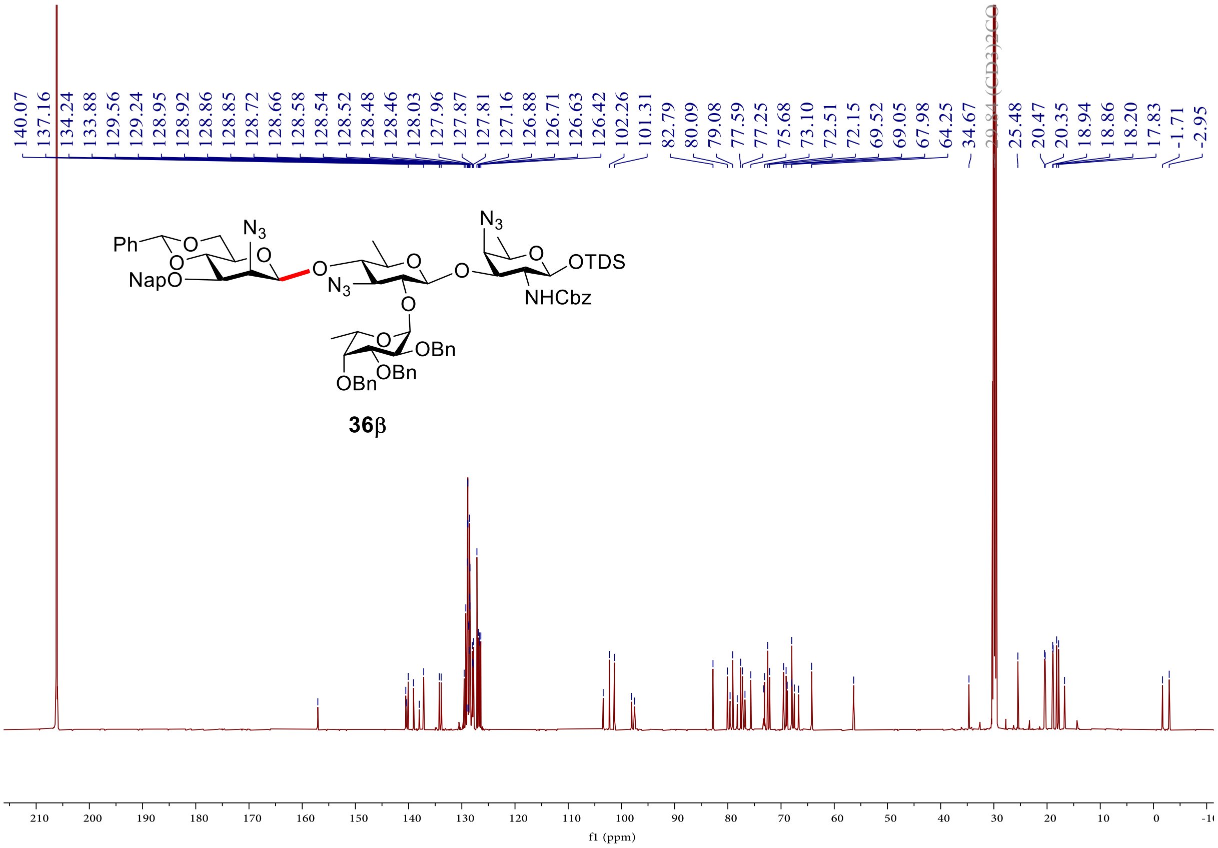
140.07 137.16 134.24 133.88 129.56 129.24 128.95 128.92 128.86 128.85 128.72 128.66 128.58 128.54 128.52 128.48 128.46 128.03 127.96 127.87 127.81 127.16 126.88 126.71 126.63 126.42 102.26 101.31 82.79 80.09 79.08 77.59 77.25 75.68 73.10 72.51 72.15 69.52 69.05 67.98 64.25 34.67 (CD3)2CO 25.48 20.47 20.35 18.94 18.86 18.20 17.83 -1.71 -2.95
Ph
O
O
N3
O
NapO
O
N3
O
O
OTDS
NHCbz
O
O
OBn
OBn
OBn
36β
210 200 190 180 170 160 150 140 130 120 110 100 90 80 70 60 50 40 30 20 10 0
f1 (ppm)

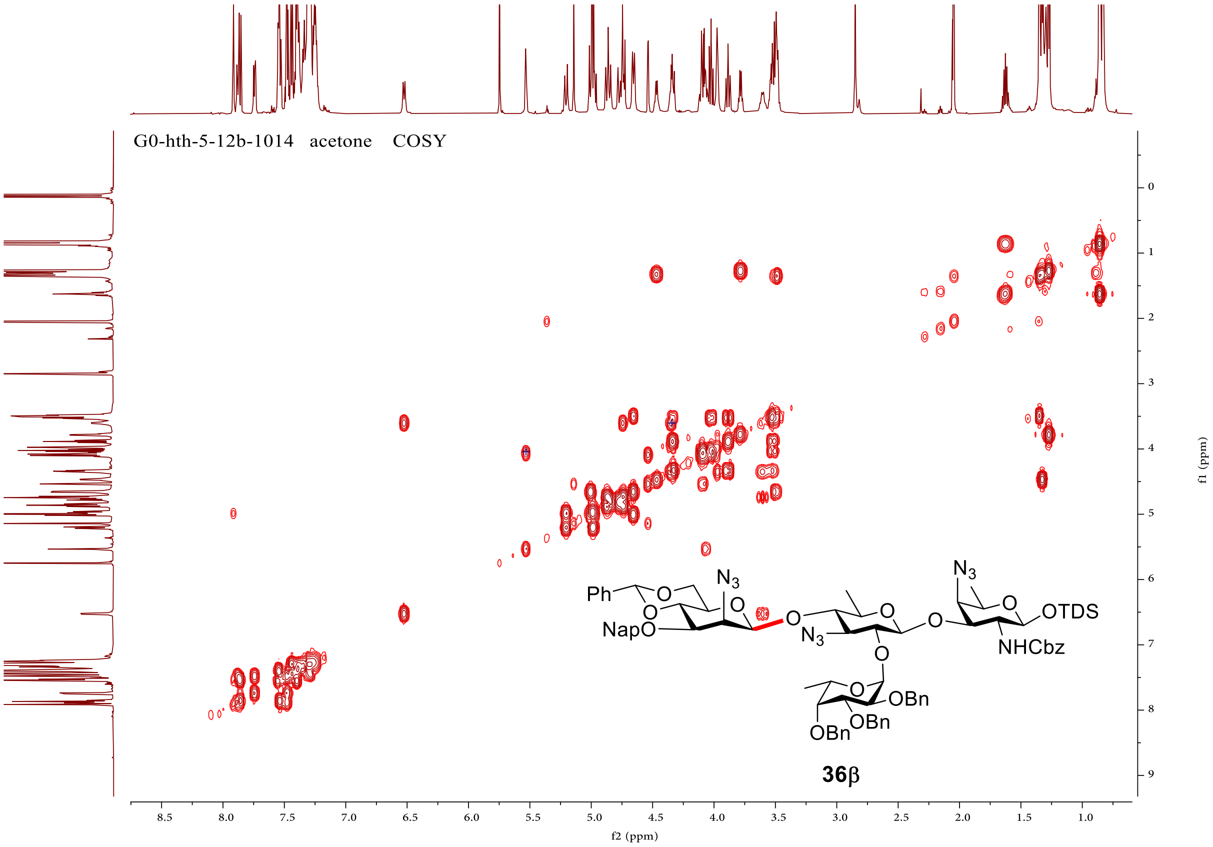
G0-hth-5-12b-1014 acetone COSY
f1 (ppm)
f2 (ppm)
Ph
O
O
NapO
N3
O
O
N3
O
O
O
N3
O
OTDS
NHCbz
O
O
OBn
OBn
OBn
36β

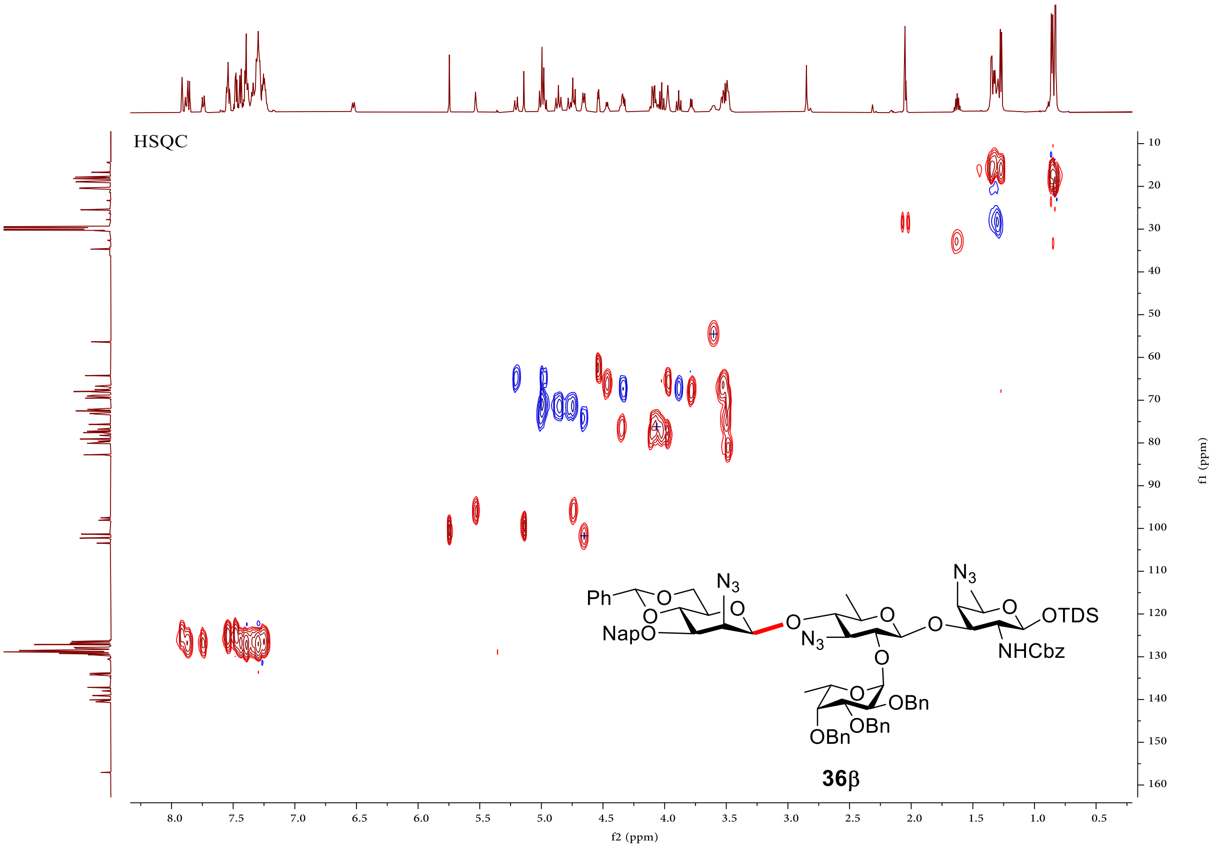
HSQC
f1 (ppm)
f2 (ppm)
Ph
O
O
NapO
N3
O
O
N3
O
O
O
N3
O
OTDS
NHCbz
O
O
OBn
OBn
OBn
36β

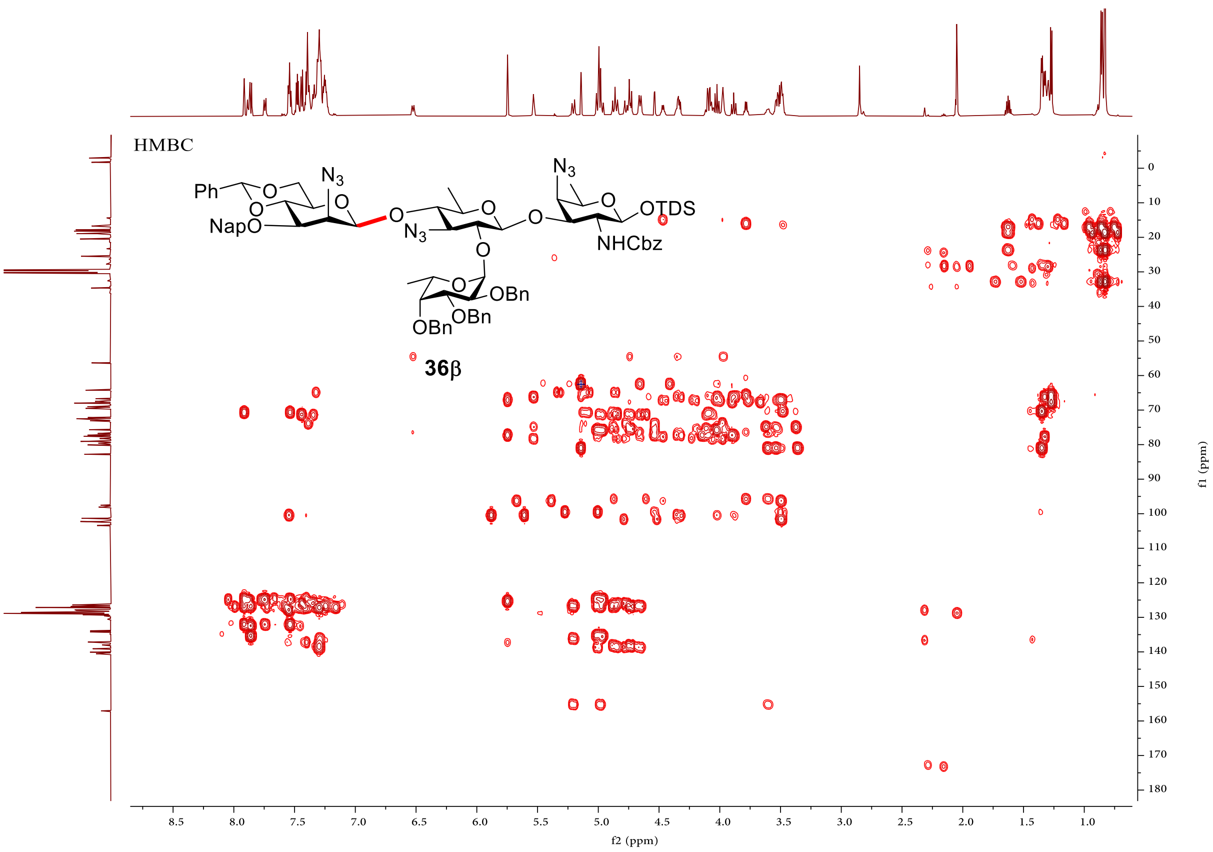
HMBC
36β
f2 (ppm)
f1 (ppm)

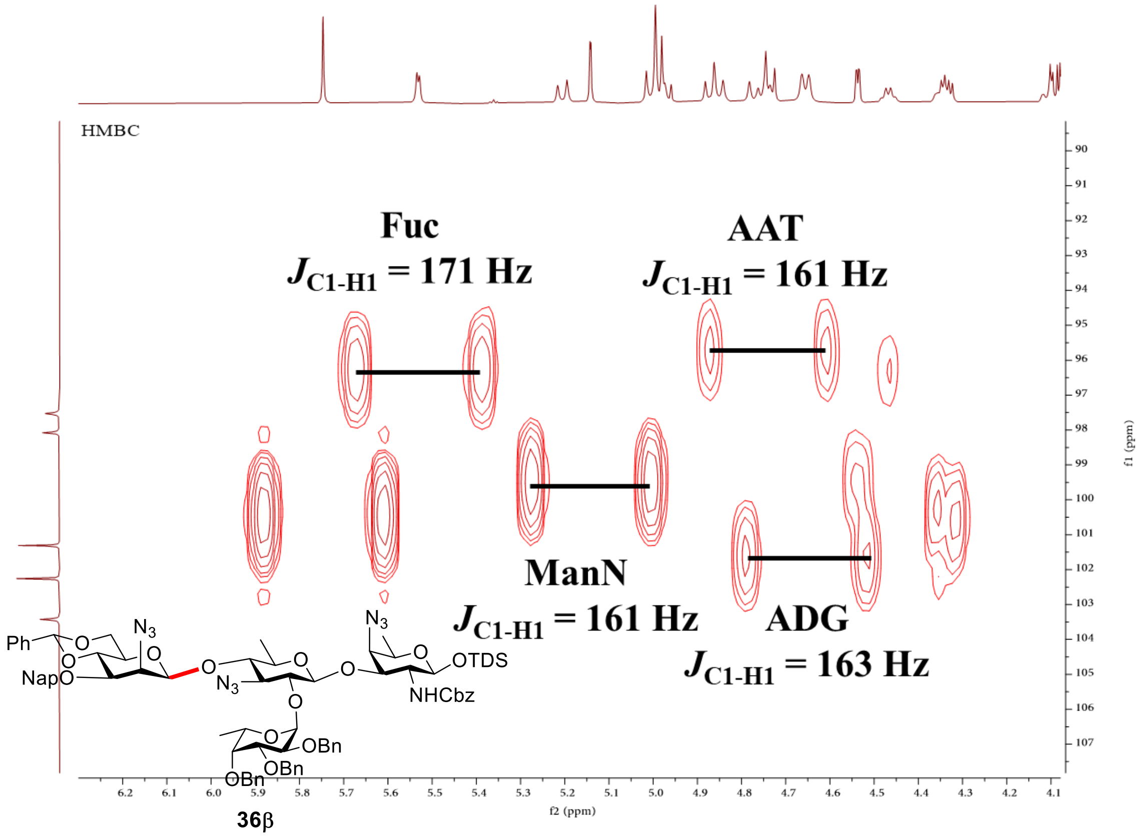
HMBC
Fuc
J_{C1-H1} = 171 Hz
AAT
J_{C1-H1} = 161 Hz
ManN
J_{C1-H1} = 161 Hz
ADG
J_{C1-H1} = 163 Hz
36β
f2 (ppm)
f1 (ppm)

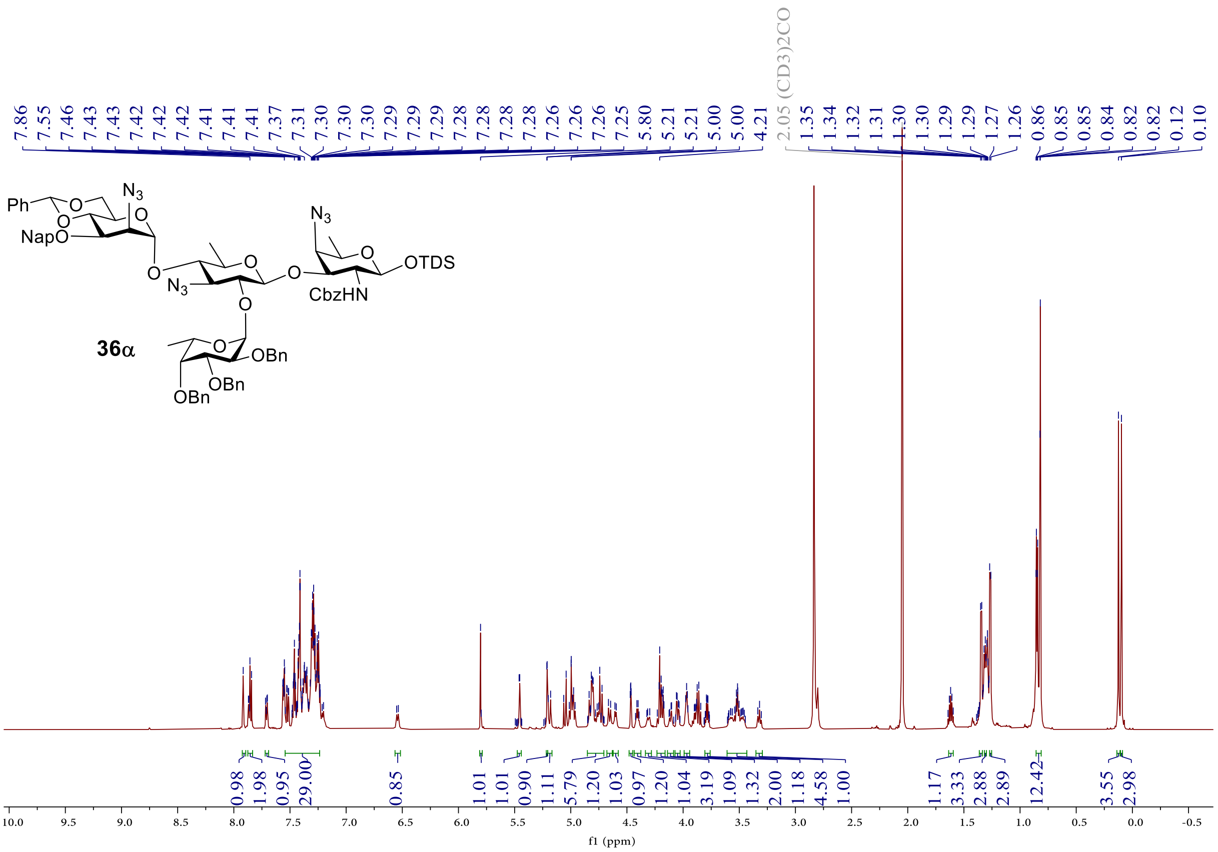
7.86 7.55 7.46 7.43 7.43 7.42 7.42 7.42 7.41 7.41 7.37 7.31 7.30 7.30 7.30 7.29 7.29 7.29 7.28 7.28 7.28 7.28 7.26 7.26 7.26 7.25 5.80 5.21 5.21 5.00 5.00 4.21
2.05 (CD3)2CO
1.35 1.34 1.32 1.31 1.30 1.30 1.29 1.29 1.27 1.26 0.86 0.85 0.85 0.84 0.82 0.82 0.12 0.10
Ph
O
O
N3
O
NapO
O
O
N3
O
N3
O
OTDS
CbzHN
O
36α
O
OBn
OBn
OBn
0.98 1.98 0.95 29.00 0.85 1.01 1.01 0.90 1.11 5.79 1.20 1.03 0.97 1.20 1.04 3.19 1.09 1.32 2.00 1.18 4.58 1.00 1.17 3.33 2.88 2.89 12.42 3.55 2.98
10.0 9.5 9.0 8.5 8.0 7.5 7.0 6.5 6.0 5.5 5.0 4.5 4.0 3.5 3.0 2.5 2.0 1.5 1.0 0.5 0.0 -0.5
f1 (ppm)

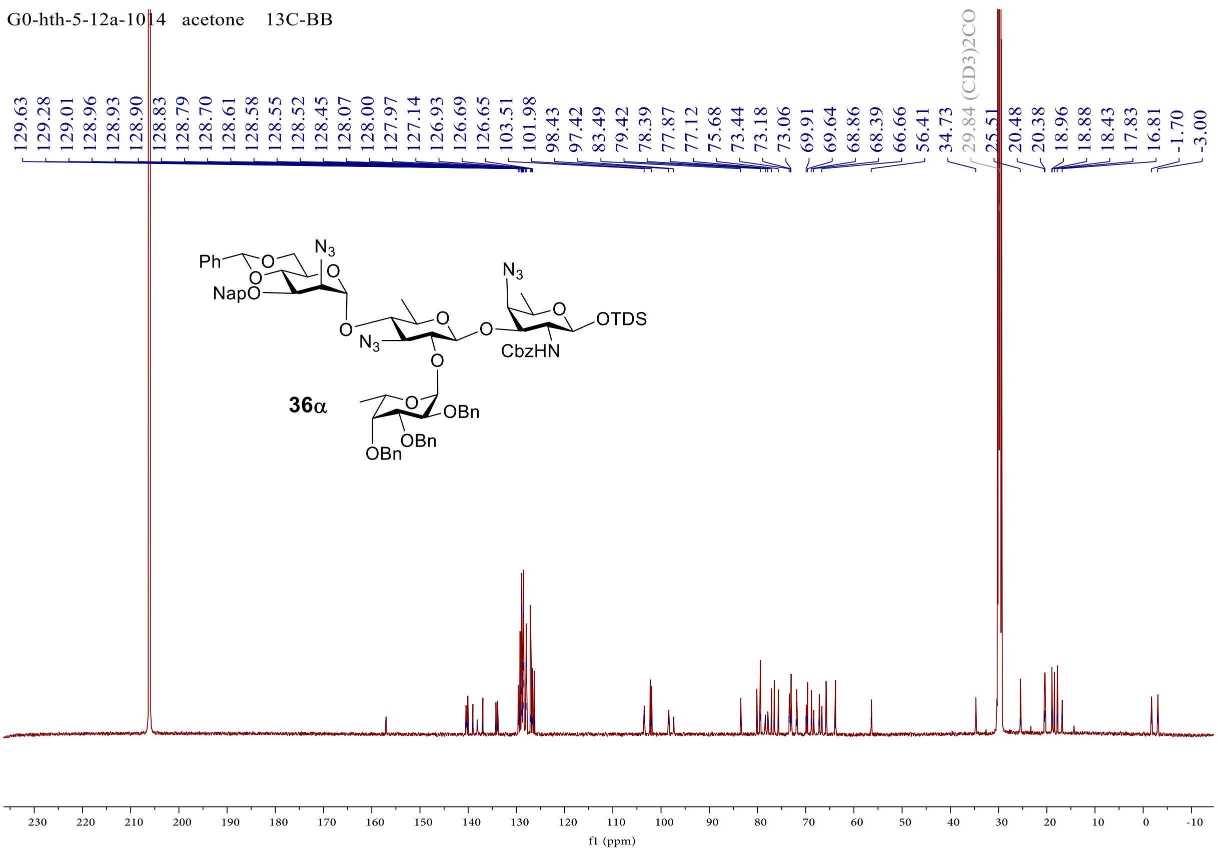
G0-hth-5-12a-1014 acetone 13C-BB
129.63 129.28 129.01 128.96 128.93 128.90 128.83 128.79 128.70 128.61 128.58 128.55 128.52 128.45 128.07 128.00 127.97 127.14 126.93 126.69 126.65 103.51 101.98 98.43 97.42 83.49 79.42 78.39 77.87 77.12 75.68 73.44 73.18 73.06 69.91 69.64 68.86 68.39 66.66 56.41 34.73
29.84 (CD3)2CO
25.51 20.48 20.38 18.96 18.88 18.43 17.83 16.81 -1.70 -3.00
Ph
O
O
N3
O
NapO
O
O
N3
O
N3
O
OTDS
CbzHN
O
36α
O
OBn
OBn
OBn
230 220 210 200 190 180 170 160 150 140 130 120 110 100 90 80 70 60 50 40 30 20 10 0 -10
f1 (ppm)

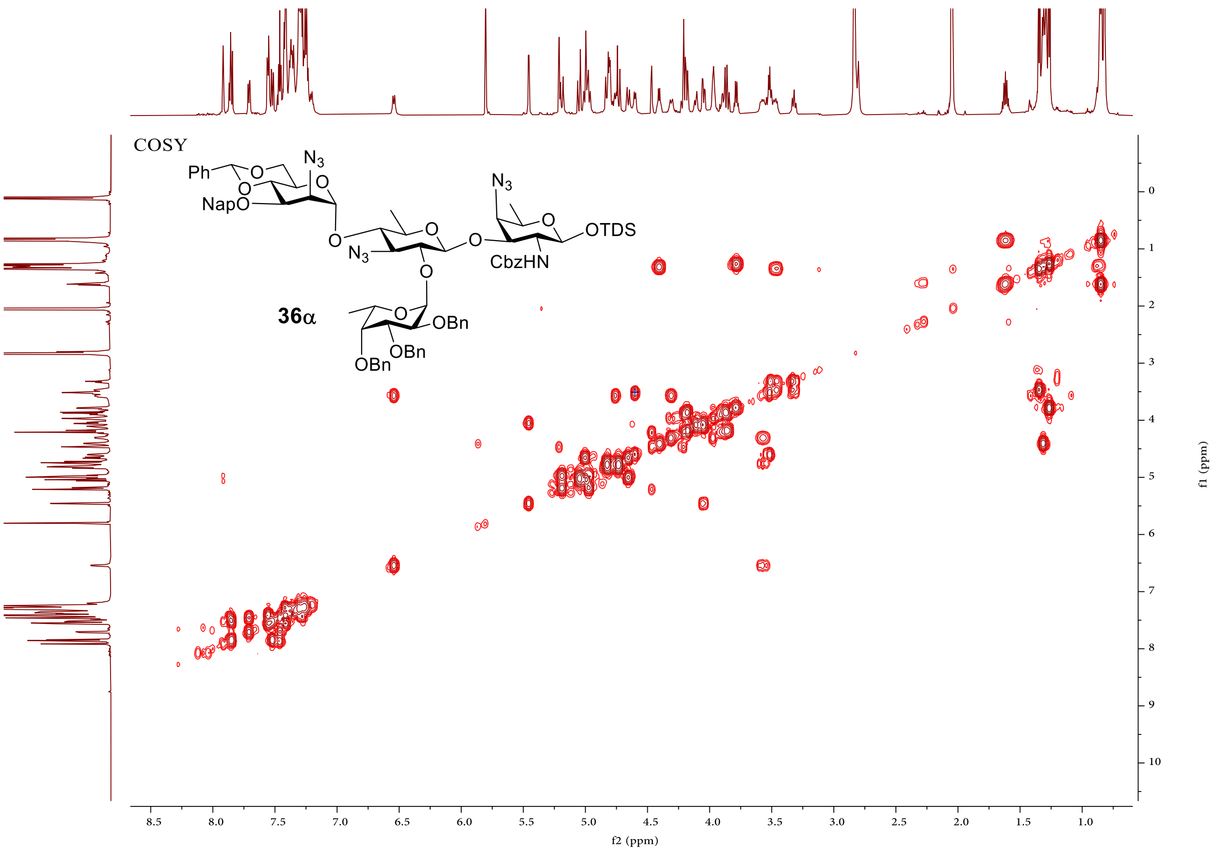
COSY
Ph
O
O
N3
O
NapO
O
N3
O
O
N3
O
OTDS
CbzHN
O
36α
O
OBn
OBn
OBn
f2 (ppm)
f1 (ppm)

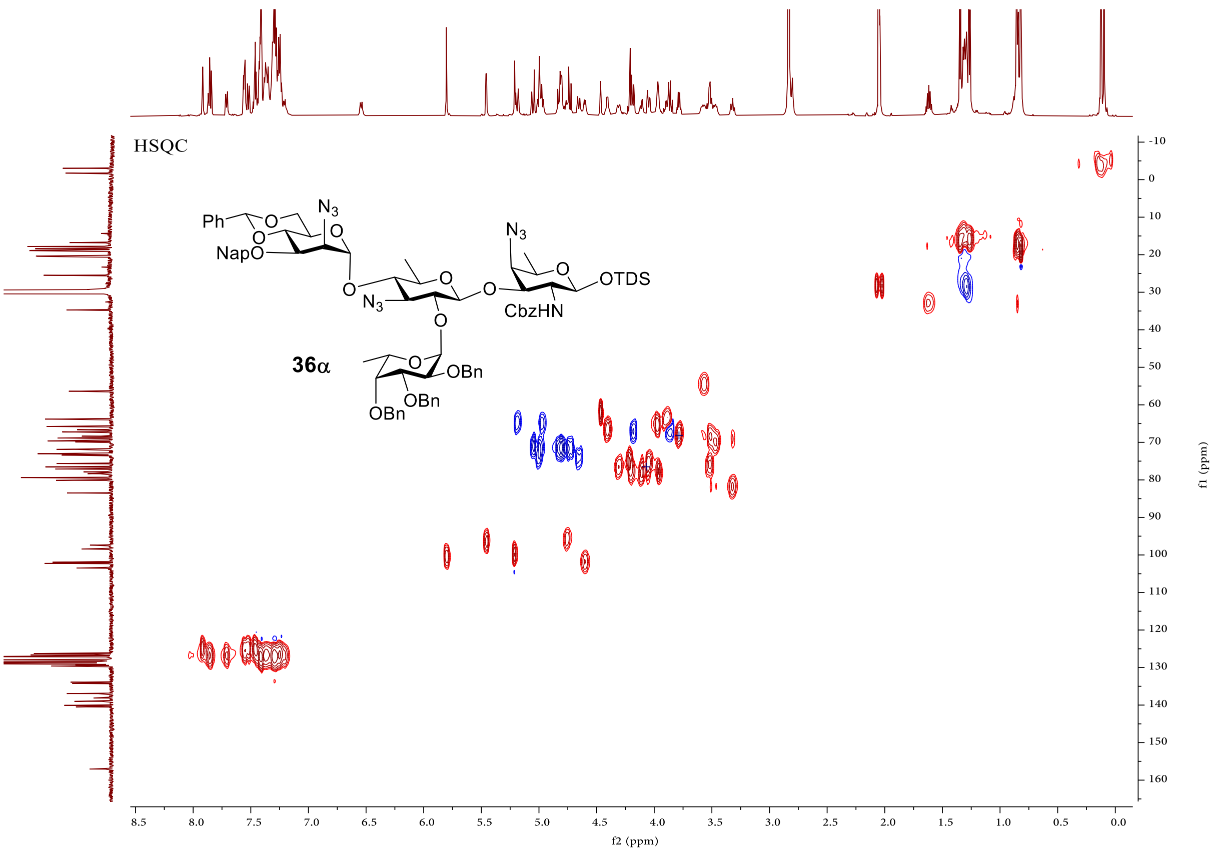
HSQC
Ph
O
O
N3
O
NapO
O
N3
O
O
N3
O
OTDS
CbzHN
O
36α
O
OBn
OBn
OBn
f2 (ppm)
f1 (ppm)

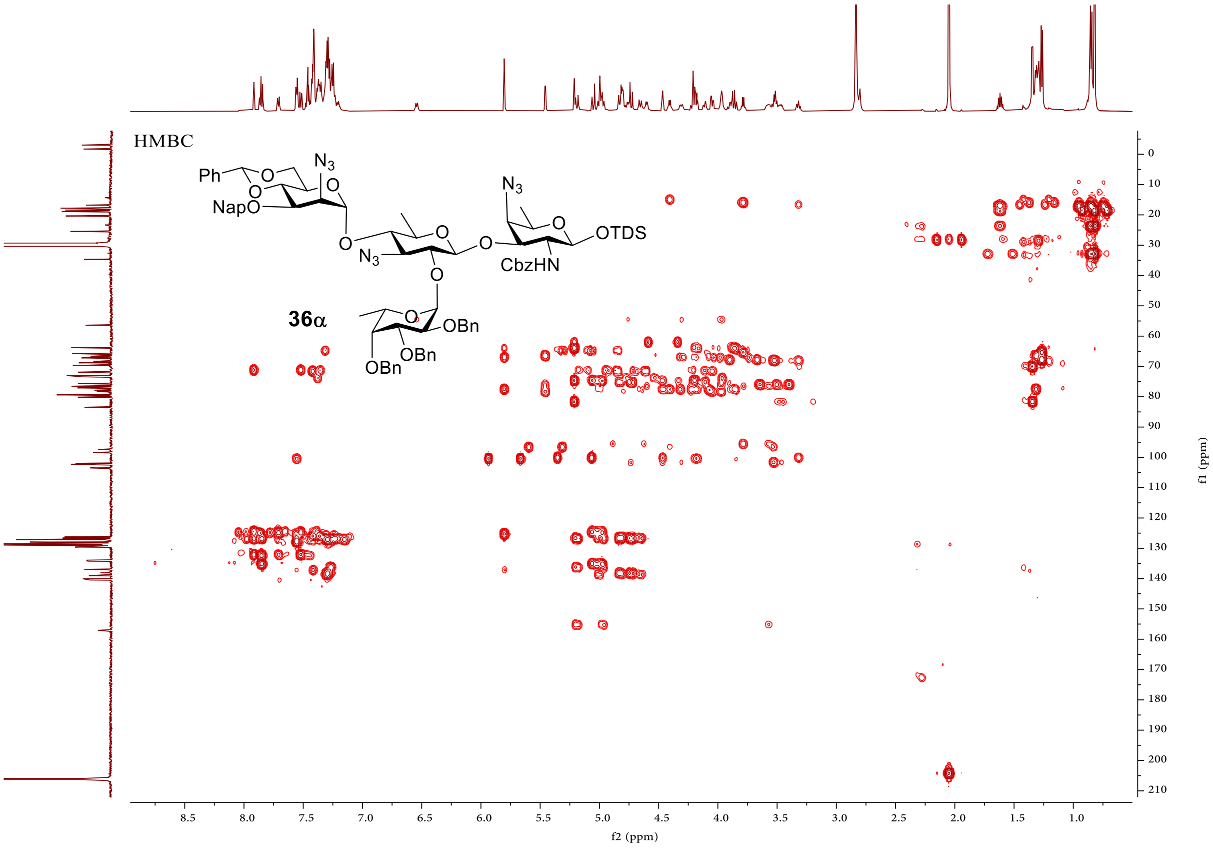
HMBC
36α
Ph
NapO
N3
O
N3
CbzHN
OTDS
OBn
f2 (ppm)
f1 (ppm)

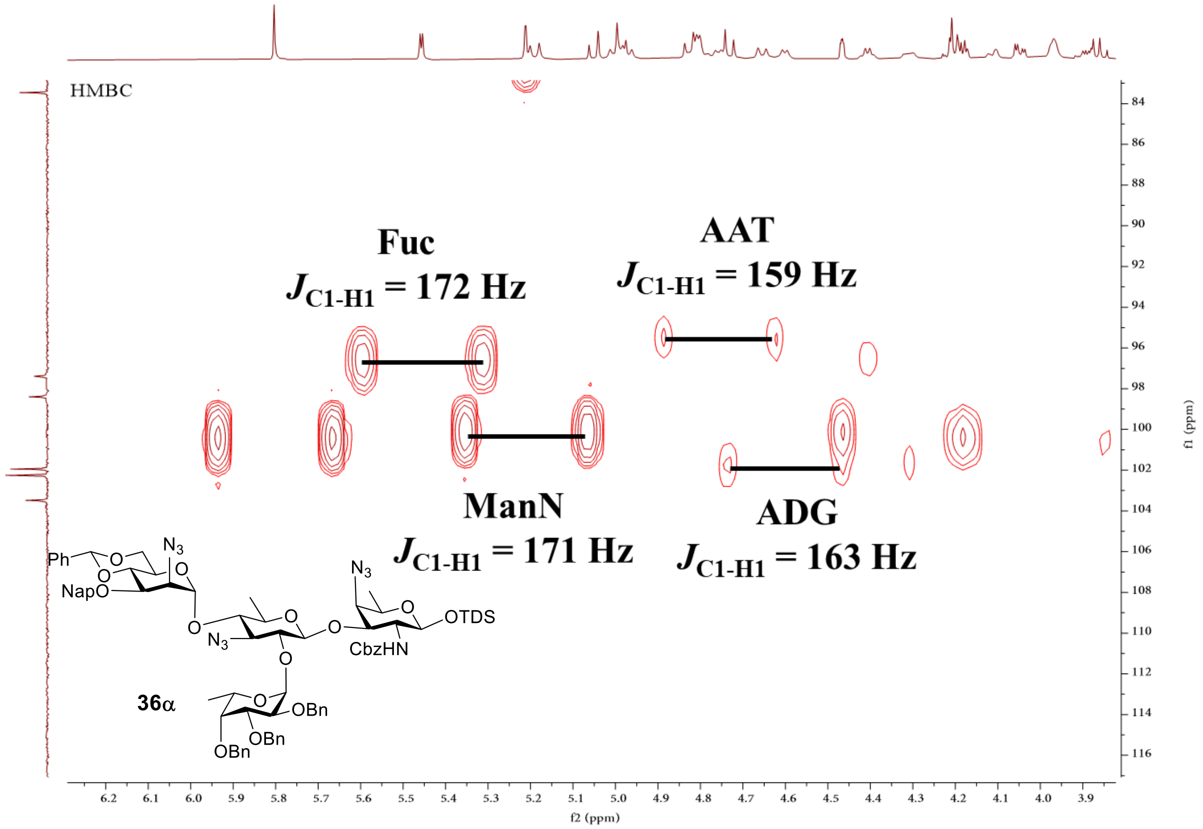
HMBC
Fuc
J_{C1-H1} = 172 Hz
AAT
J_{C1-H1} = 159 Hz
ManN
J_{C1-H1} = 171 Hz
ADG
J_{C1-H1} = 163 Hz
36α
Ph
NapO
N3
O
CbzHN
OTDS
OBn
f2 (ppm)
f1 (ppm)

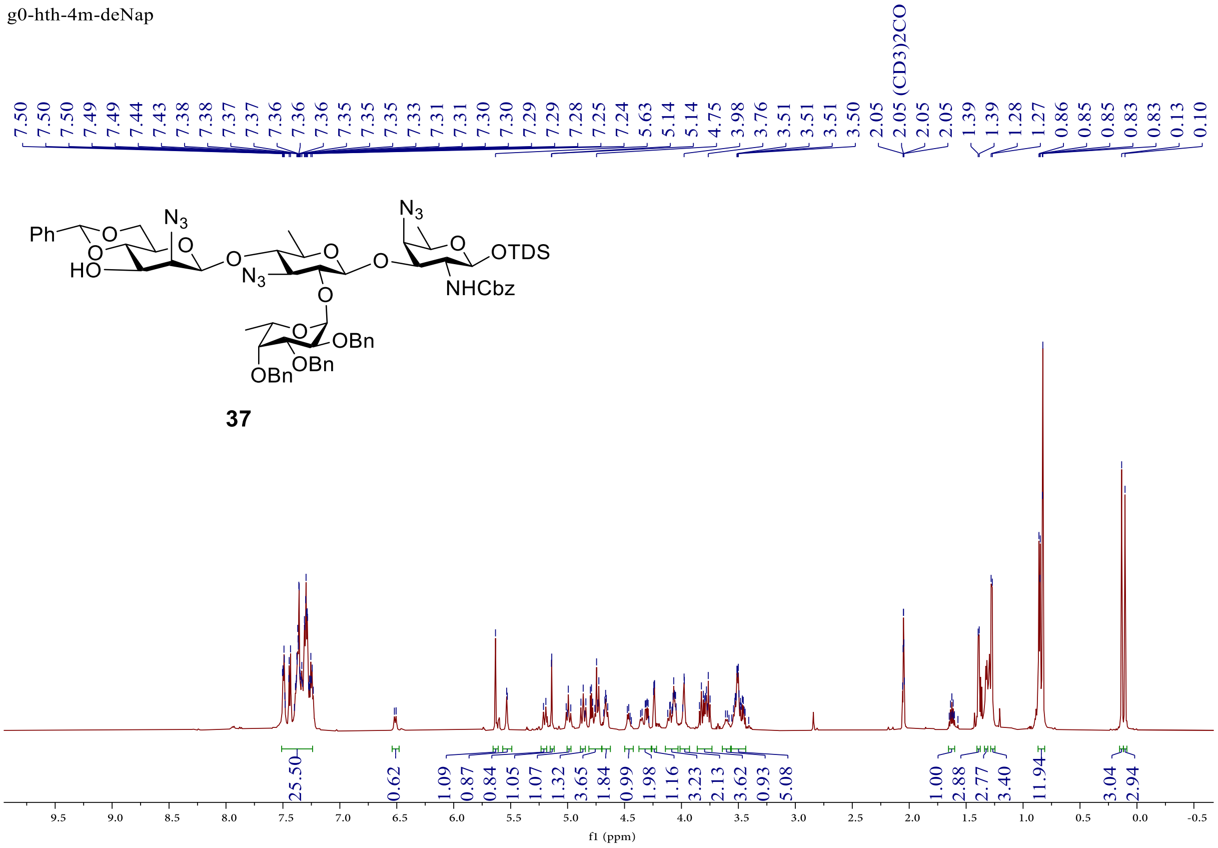
g0-hth-4m-deNap
7.50 7.50 7.50 7.49 7.44 7.43 7.38 7.38 7.37 7.37 7.36 7.36 7.35 7.35 7.35 7.33 7.31 7.31 7.30 7.30 7.29 7.29 7.28 7.25 7.24 5.63 5.14 5.14 4.75 3.98 3.76 3.51 3.51 3.51 3.50 2.05 2.05 (CD3)2CO 2.05 2.05 1.39 1.39 1.28 1.27 0.86 0.85 0.85 0.83 0.83 0.13 0.10
Ph
O
O
HO
N3
O
O
N3
O
O
O
N3
O
OTDS
NHCbz
O
OBn
OBn
OBn
37
25.50 0.62 1.09 0.87 0.84 1.05 1.07 1.32 3.65 1.84 0.99 1.98 1.16 3.23 2.13 3.62 0.93 5.08 1.00 2.88 2.77 3.40 11.94 3.04 2.94
9.5 9.0 8.5 8.0 7.5 7.0 6.5 6.0 5.5 5.0 4.5 4.0 3.5 3.0 2.5 2.0 1.5 1.0 0.5 0.0 -0.5
f1 (ppm)

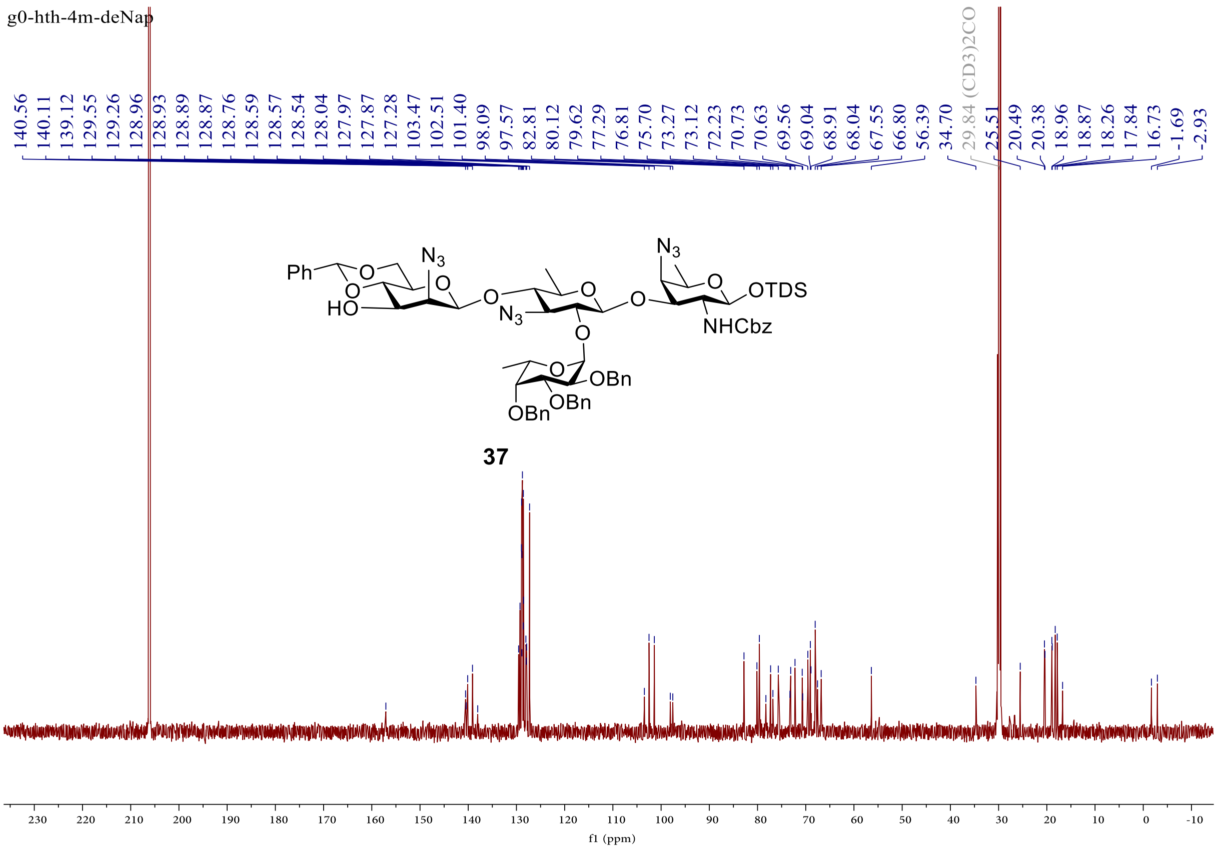
g0-hth-4m-deNap
140.56 140.11 139.12 129.55 129.26 128.96 128.93 128.89 128.87 128.76 128.59 128.57 128.54 128.04 127.97 127.87 127.28 103.47 102.51 101.40 98.09 97.57 82.81 80.12 79.62 77.29 76.81 75.70 73.27 73.12 72.23 70.73 70.63 69.56 69.04 68.91 68.04 67.55 66.80 56.39 34.70 29.84 (CD3)2CO 25.51 20.49 20.38 18.96 18.87 18.26 17.84 16.73 -1.69 -2.93
Ph
O
O
HO
N3
O
O
N3
O
O
O
N3
O
OTDS
NHCbz
O
OBn
OBn
OBn
37
230 220 210 200 190 180 170 160 150 140 130 120 110 100 90 80 70 60 50 40 30 20 10 0 -10
f1 (ppm)

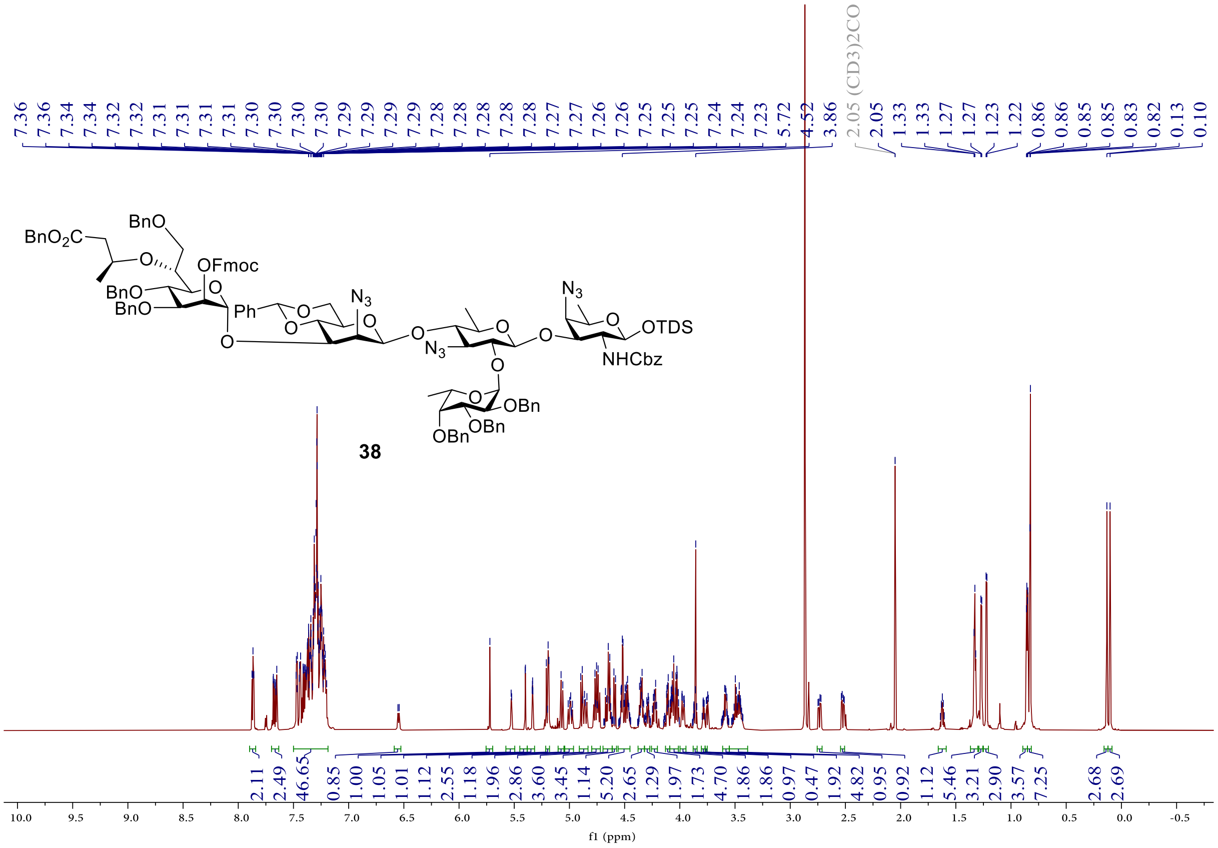
7.36 7.36 7.34 7.34 7.32 7.32 7.31 7.31 7.31 7.31 7.30 7.30 7.30 7.30 7.29 7.29 7.29 7.29 7.28 7.28 7.28 7.28 7.28 7.27 7.27 7.26 7.26 7.25 7.25 7.25 7.24 7.24 7.23 5.72 4.52 3.86 2.05 (CD3)2CO 2.05 1.33 1.33 1.27 1.27 1.23 1.22 0.86 0.86 0.85 0.85 0.83 0.82 0.13 0.10
2.11 2.49 46.65 0.85 1.00 1.05 1.01 1.12 2.55 1.18 1.96 2.86 3.60 3.45 1.14 5.20 2.65 1.29 1.97 1.73 4.70 1.86 0.97 0.47 1.92 4.82 0.95 0.92 1.12 5.46 3.21 2.90 3.57 7.25 2.68 2.69
38
f1 (ppm)

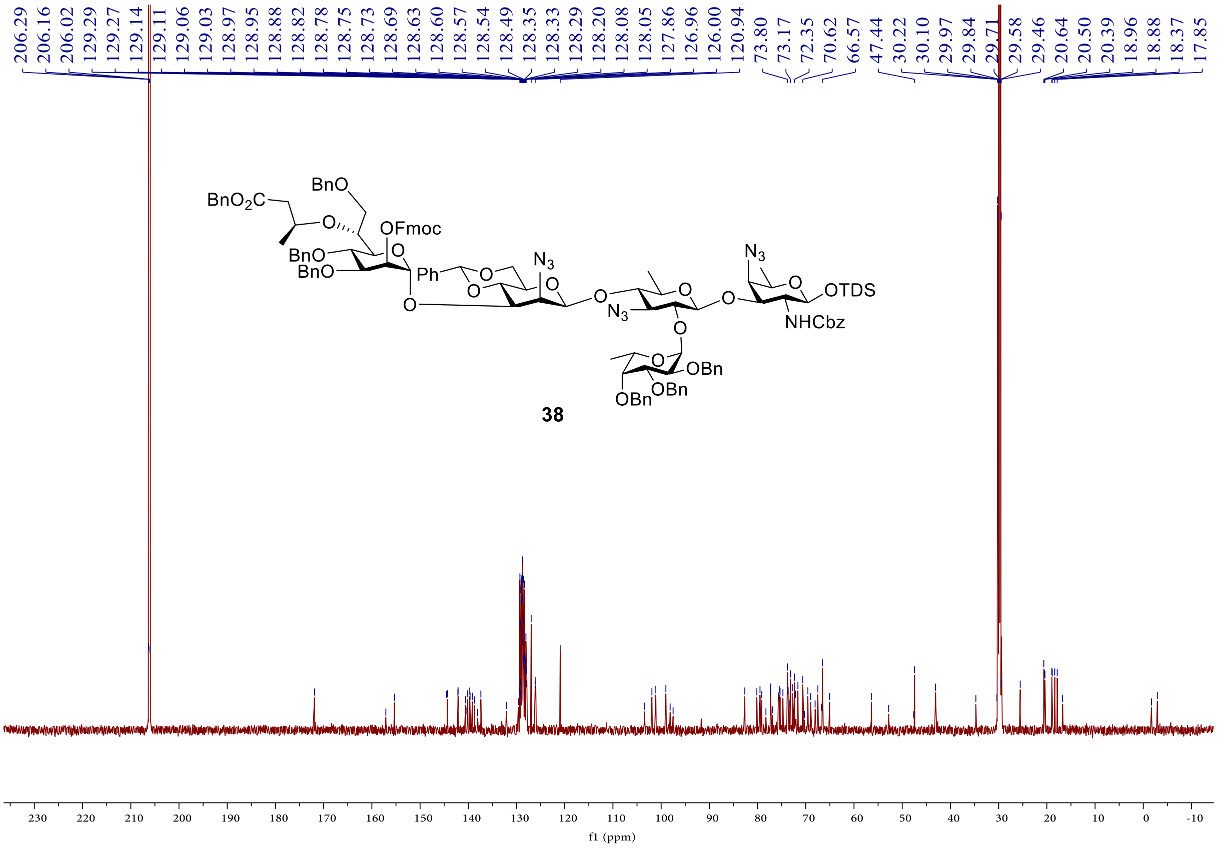
206.29 206.16 206.02 129.29 129.27 129.14 129.11 129.06 129.03 128.97 128.95 128.88 128.82 128.78 128.75 128.73 128.69 128.63 128.60 128.57 128.54 128.49 128.35 128.33 128.29 128.20 128.08 128.05 127.86 126.96 126.00 120.94 73.80 73.17 72.35 70.62 66.57 47.44 30.22 30.10 29.97 29.84 29.71 29.58 29.46 20.64 20.50 20.39 18.96 18.88 18.37 17.85
38
f1 (ppm)

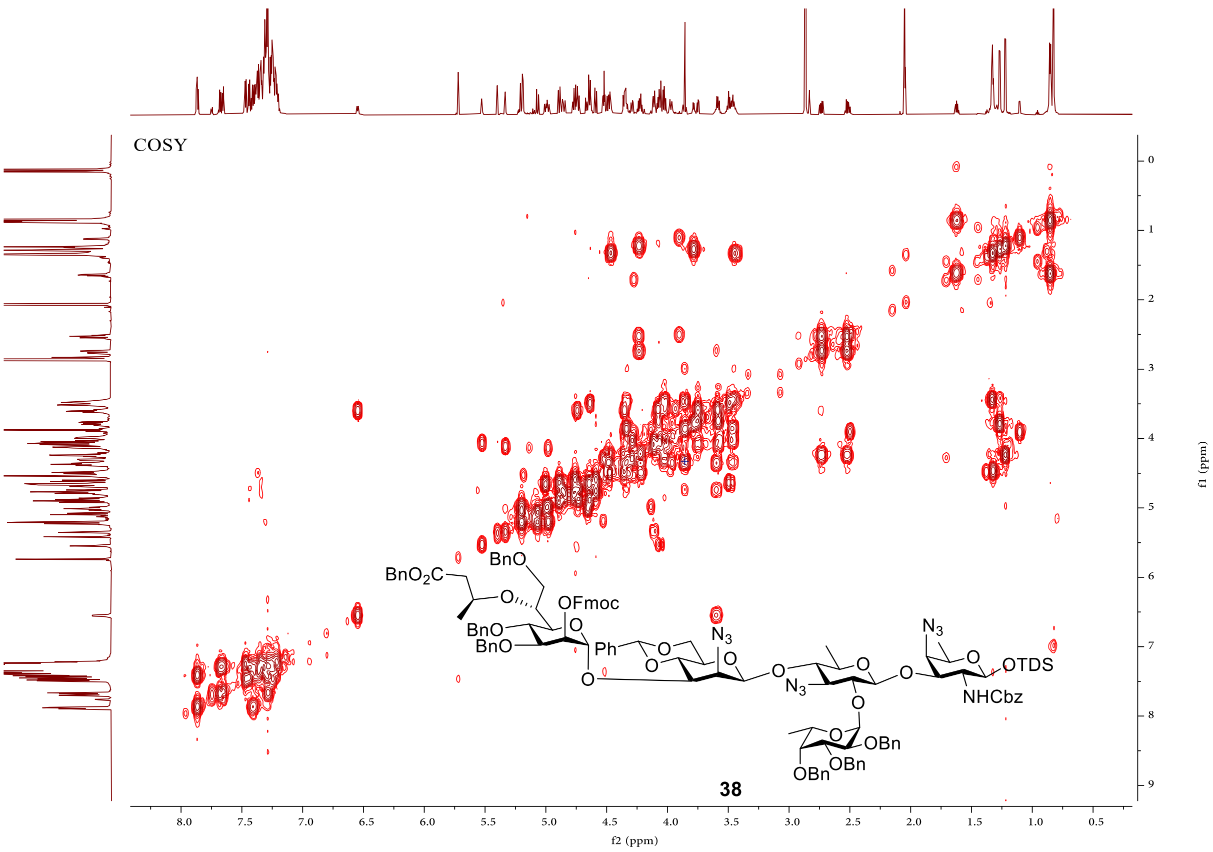
COSY
BnO
BnO$_2$C
OFmoc
BnO
BnO
Ph
O
N$_3$
N$_3$
OTDS
NHCbz
OBn
OBn
OBn
38
f2 (ppm)
f1 (ppm)

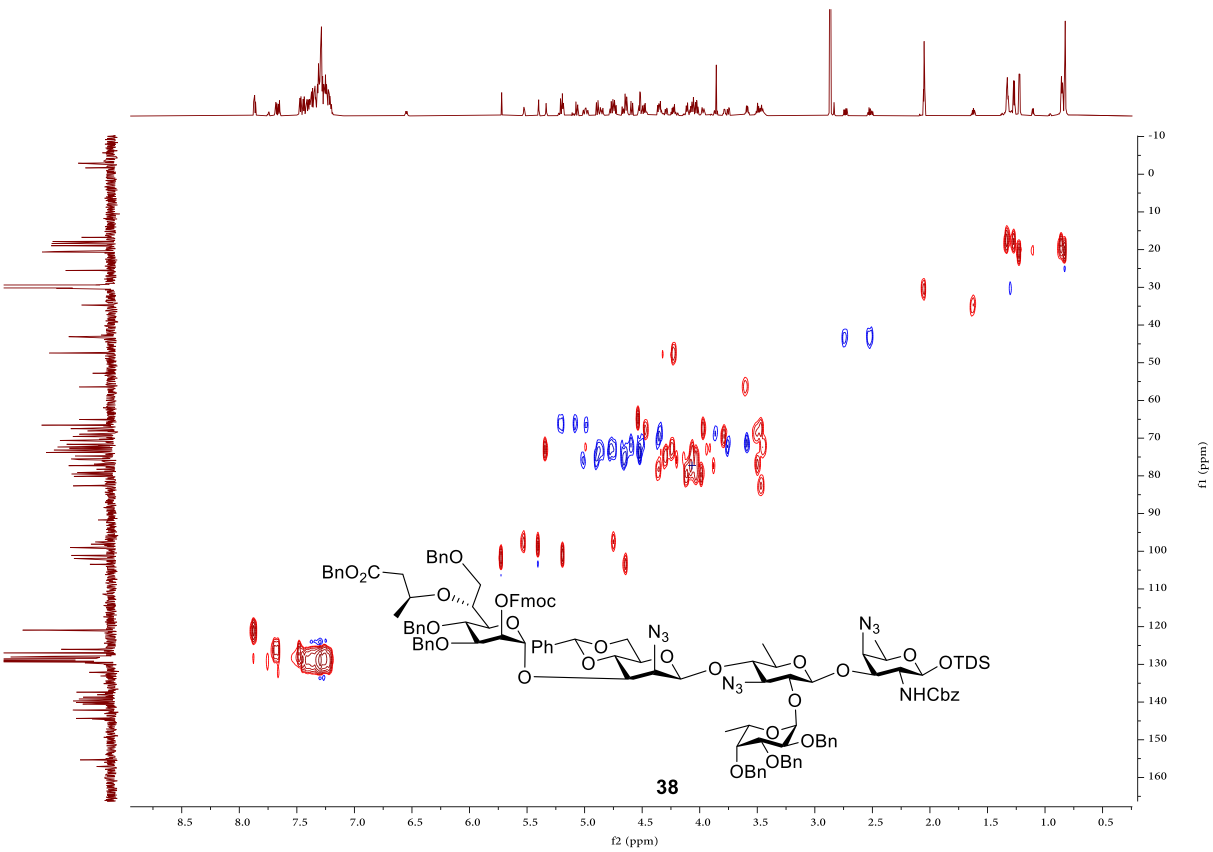
BnO
BnO$_2$C
OFmoc
BnO
BnO
Ph
N$_3$
N$_3$
OTDS
NHCbz
OBn
OBn
OBn
38
f2 (ppm)
f1 (ppm)

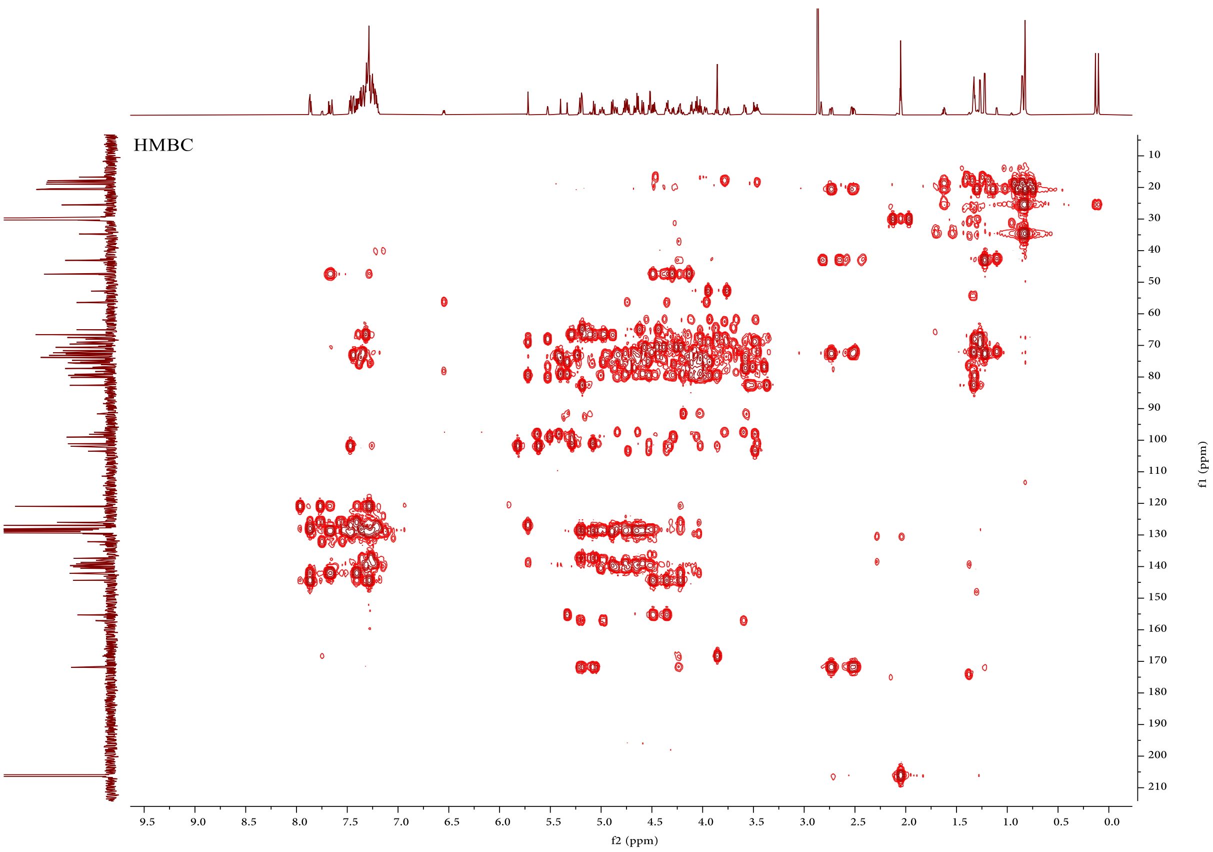
HMBC
f2 (ppm)
f1 (ppm)

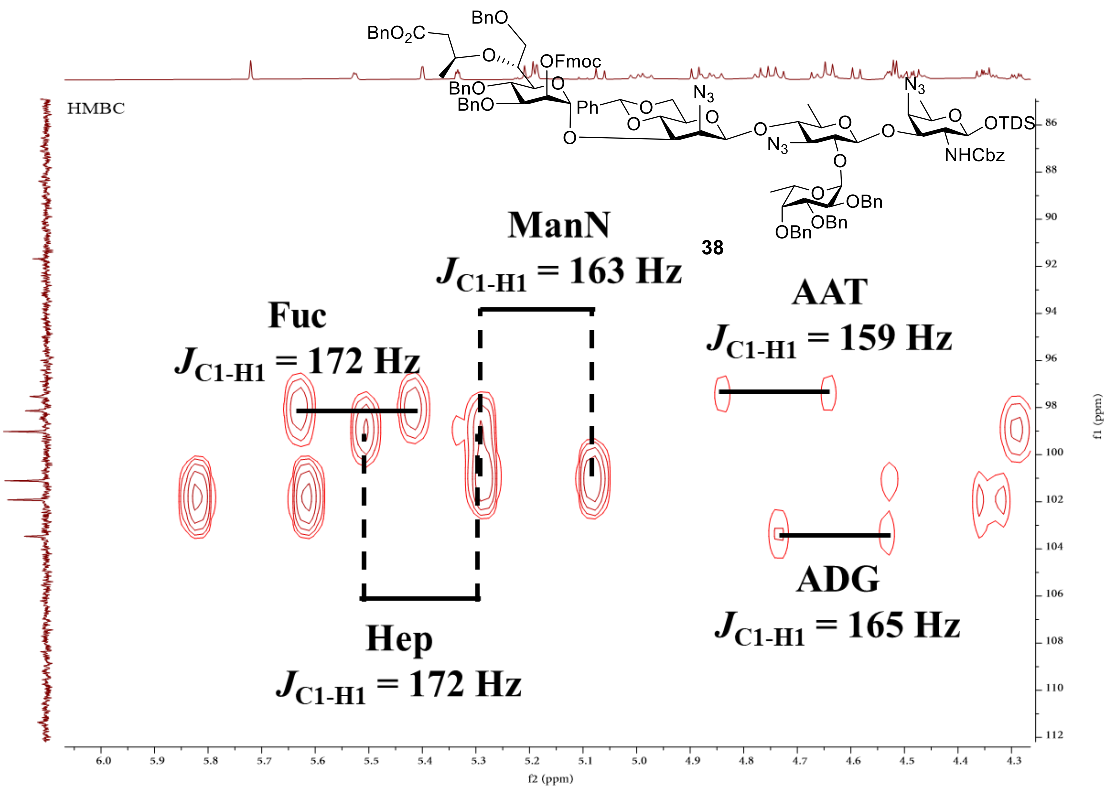
HMBC
BnO
BnO2C
OFmoc
BnO
BnO
Ph
N3
OTDS
NHCbz
OBn
OBn
OBn
38
ManN
JC1-H1 = 163 Hz
Fuc
JC1-H1 = 172 Hz
AAT
JC1-H1 = 159 Hz
ADG
JC1-H1 = 165 Hz
Hep
JC1-H1 = 172 Hz
f2 (ppm)
f1 (ppm)

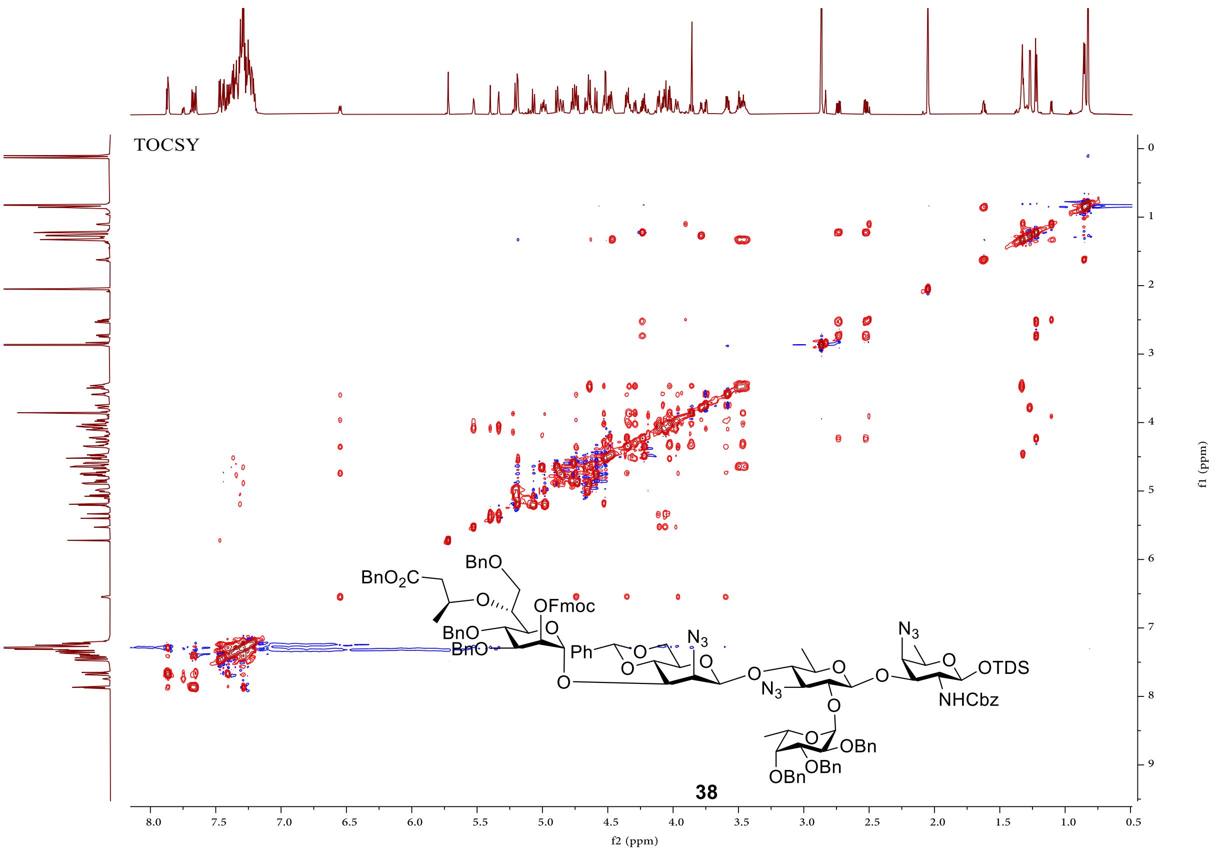
TOCSY
BnO
BnO2C
OFmoc
BnO
BnO
O
Ph
N3
O
O
N3
O
N3
OTDS
NHCbz
OBn
OBn
OBn
38
f2 (ppm)
f1 (ppm)

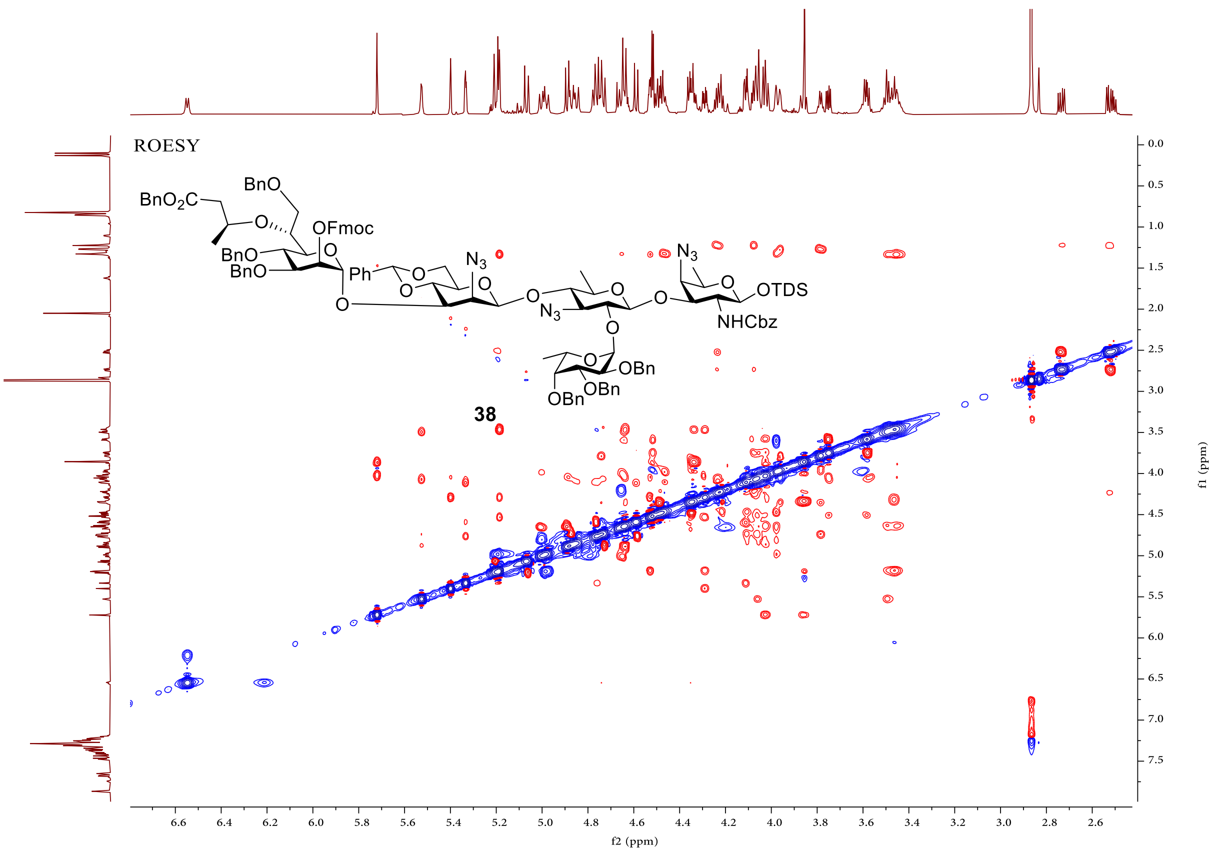
ROESY
BnO
BnO2C
OFmoc
BnO
BnO
Ph
N3
N3
N3
OTDS
NHCbz
OBn
OBn
OBn
38
f2 (ppm)
f1 (ppm)

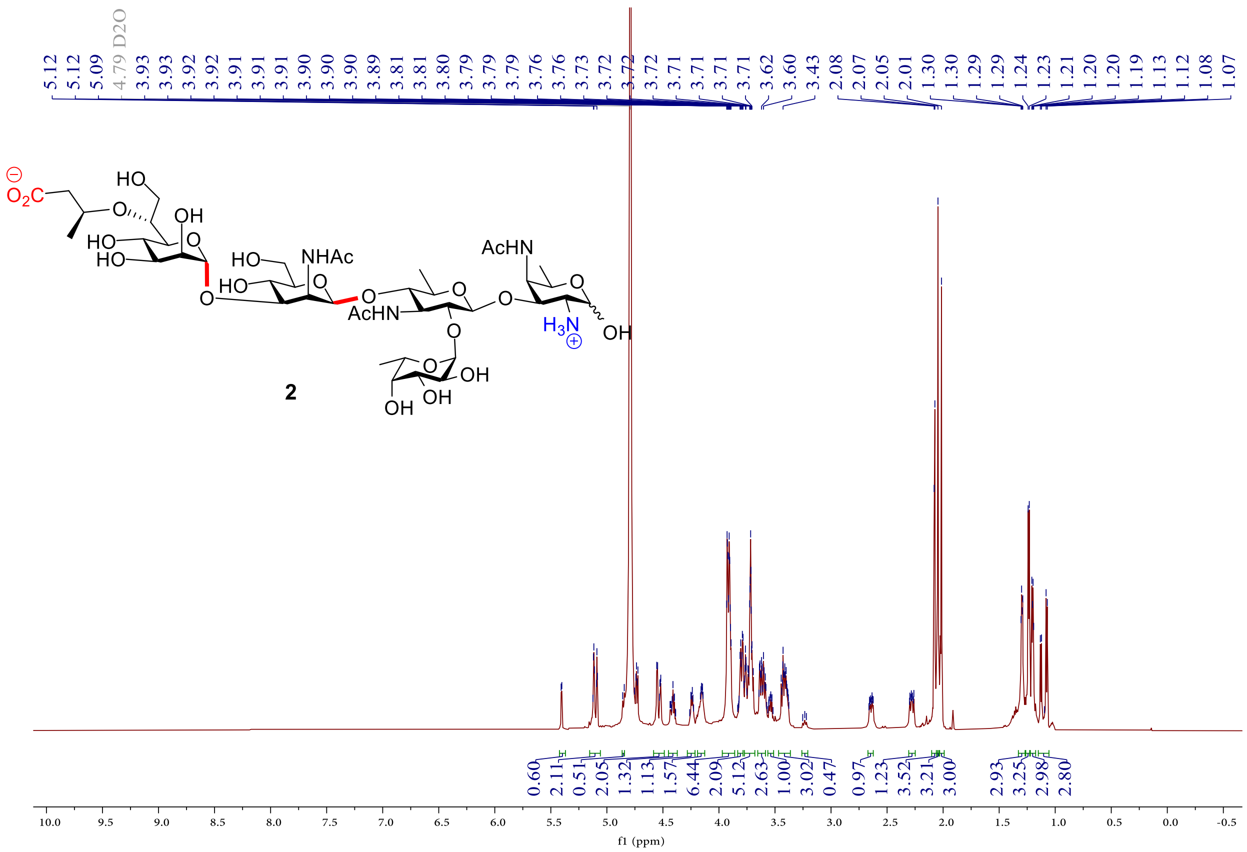
5.12 5.12 5.09 4.79 D2O 3.93 3.93 3.92 3.92 3.91 3.91 3.91 3.90 3.90 3.90 3.89 3.81 3.81 3.80 3.79 3.79 3.79 3.76 3.76 3.73 3.72 3.72 3.72 3.71 3.71 3.71 3.71 3.62 3.60 3.43 2.08 2.07 2.05 2.01 1.30 1.30 1.29 1.29 1.24 1.23 1.21 1.20 1.20 1.19 1.13 1.12 1.08 1.07
2
0.60 2.11 0.51 2.05 1.32 1.13 1.57 6.44 2.09 5.12 2.63 1.00 3.02 0.47 0.97 1.23 3.52 3.21 3.00 2.93 3.25 2.98 2.80
f1 (ppm)

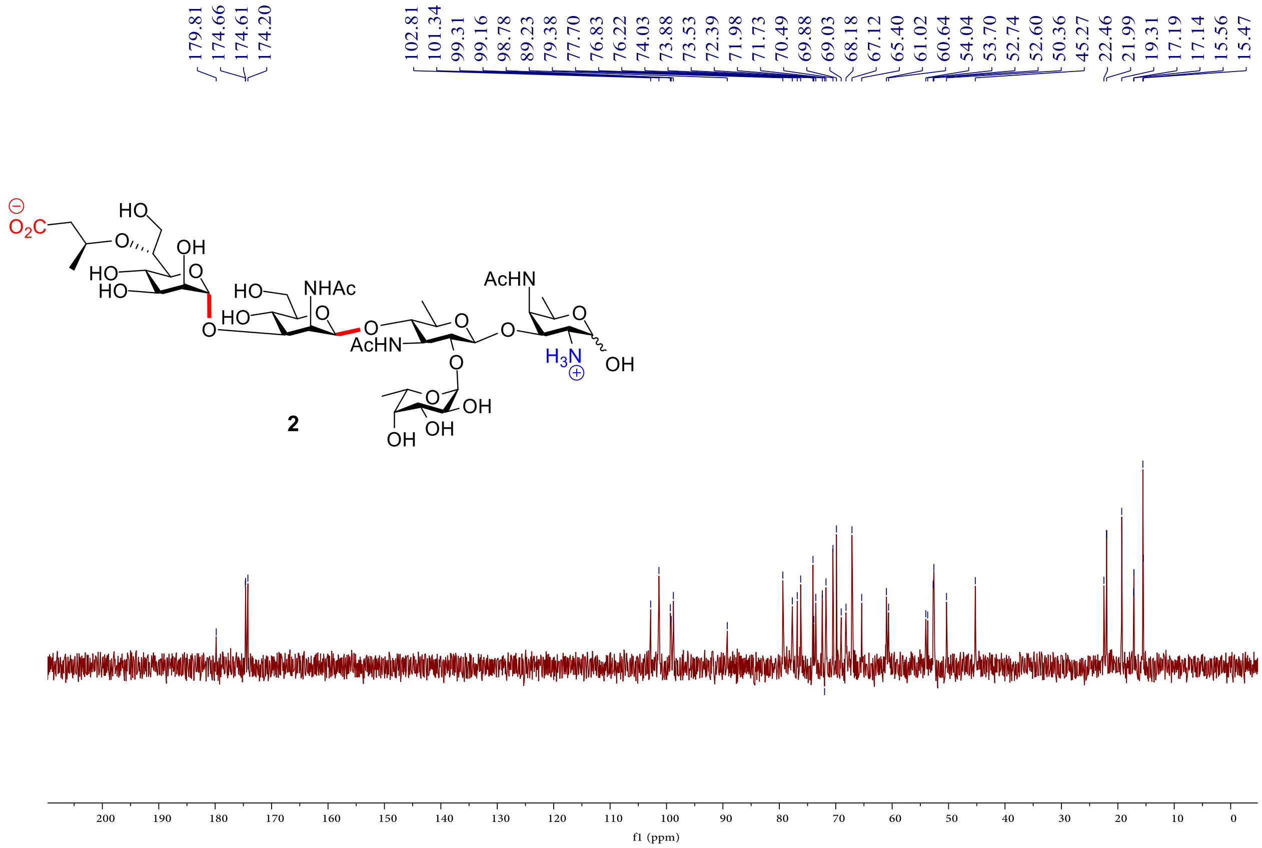
179.81 174.66 174.61 174.20
102.81 101.34 99.31 99.16 98.78 89.23 79.38 77.70 76.83 76.22 74.03 73.88 73.53 72.39 71.98 71.73 70.49 69.88 69.03 68.18 67.12 65.40 61.02 60.64 54.04 53.70 52.74 52.60 50.36 45.27 22.46 21.99 19.31 17.19 17.14 15.56 15.47
2
f1 (ppm)

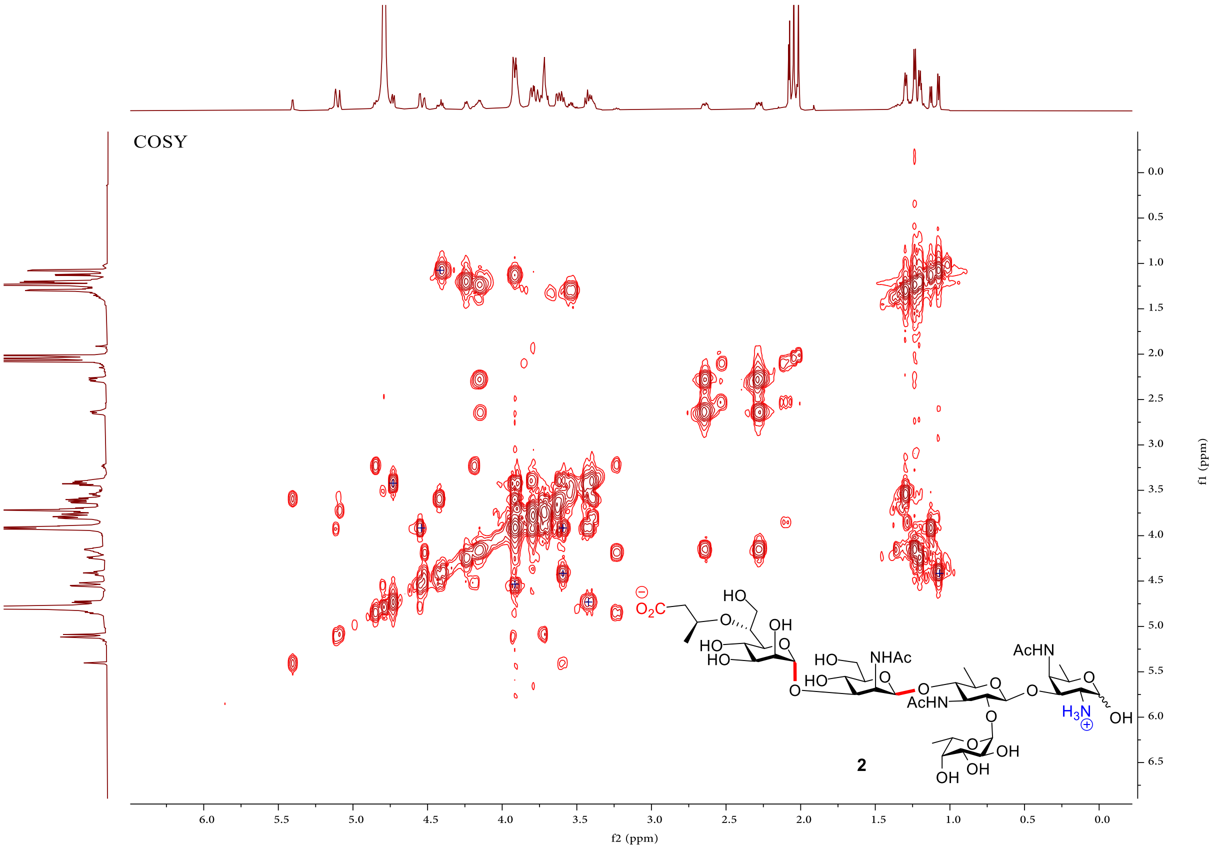
COSY
f2 (ppm)
f1 (ppm)
2

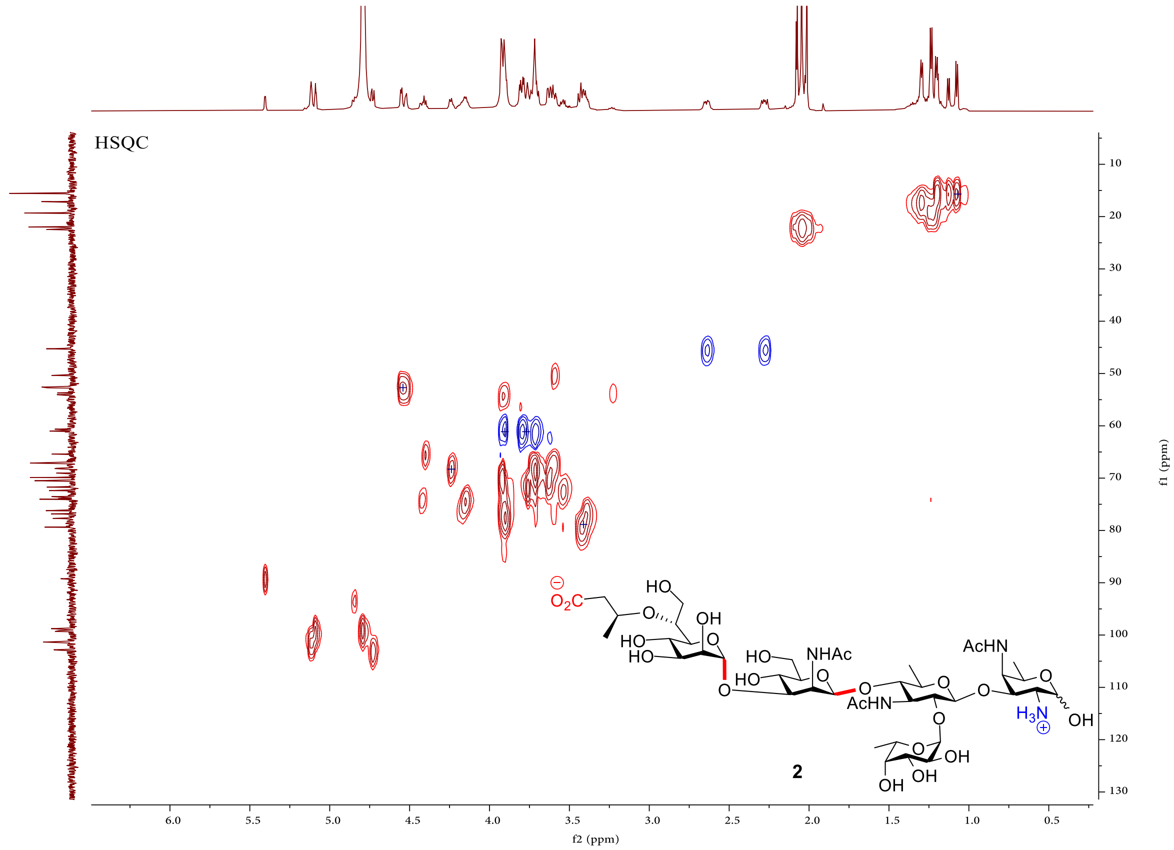
HSQC
f2 (ppm)
f1 (ppm)
2

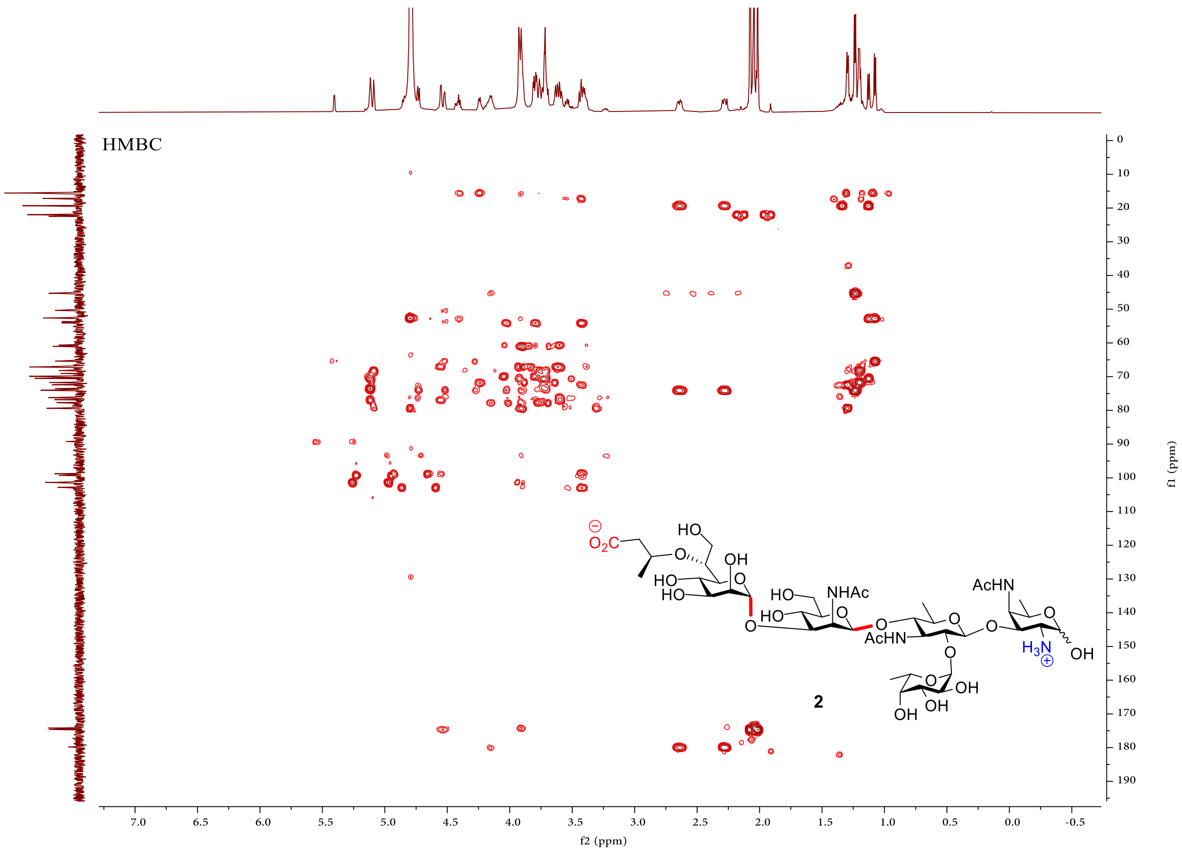
HMBC
f2 (ppm)
f1 (ppm)
2

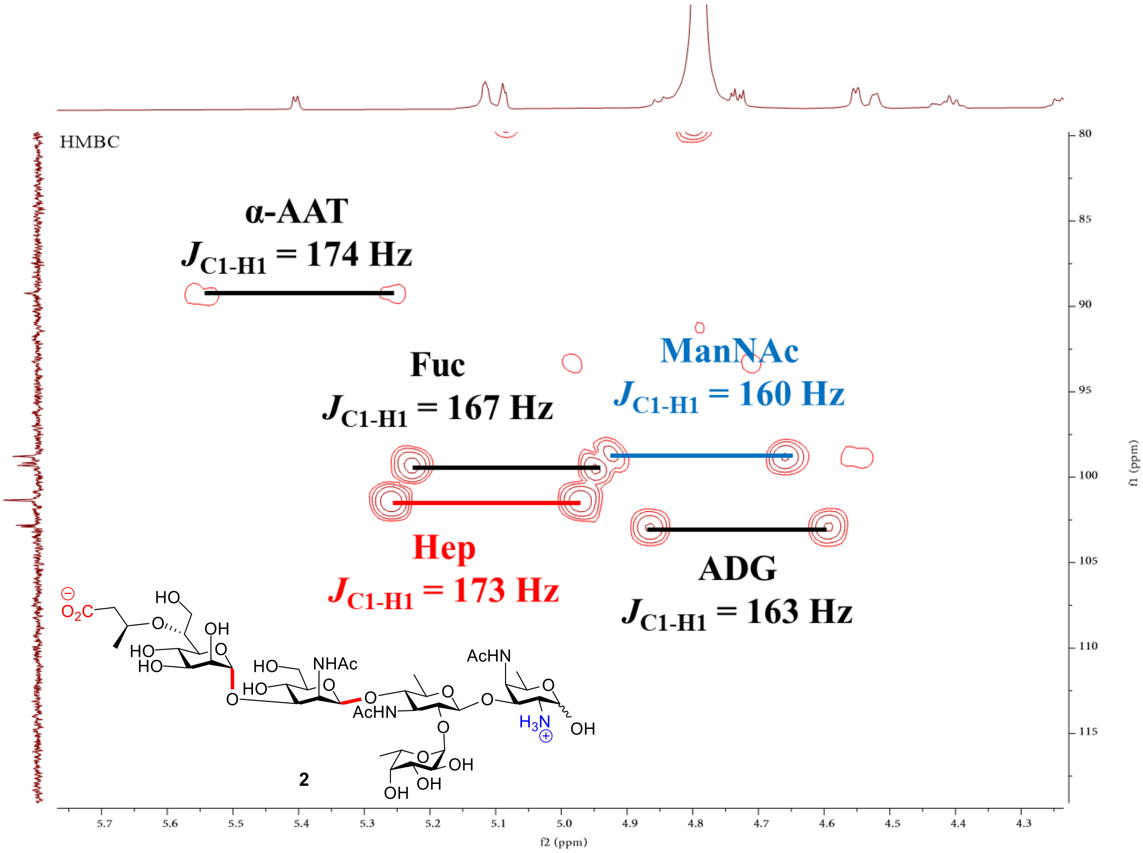
HMBC
α-AAT
J C1-H1 = 174 Hz
Fuc
J C1-H1 = 167 Hz
ManNAc
J C1-H1 = 160 Hz
Hep
J C1-H1 = 173 Hz
ADG
J C1-H1 = 163 Hz
2
f2 (ppm)
f1 (ppm)

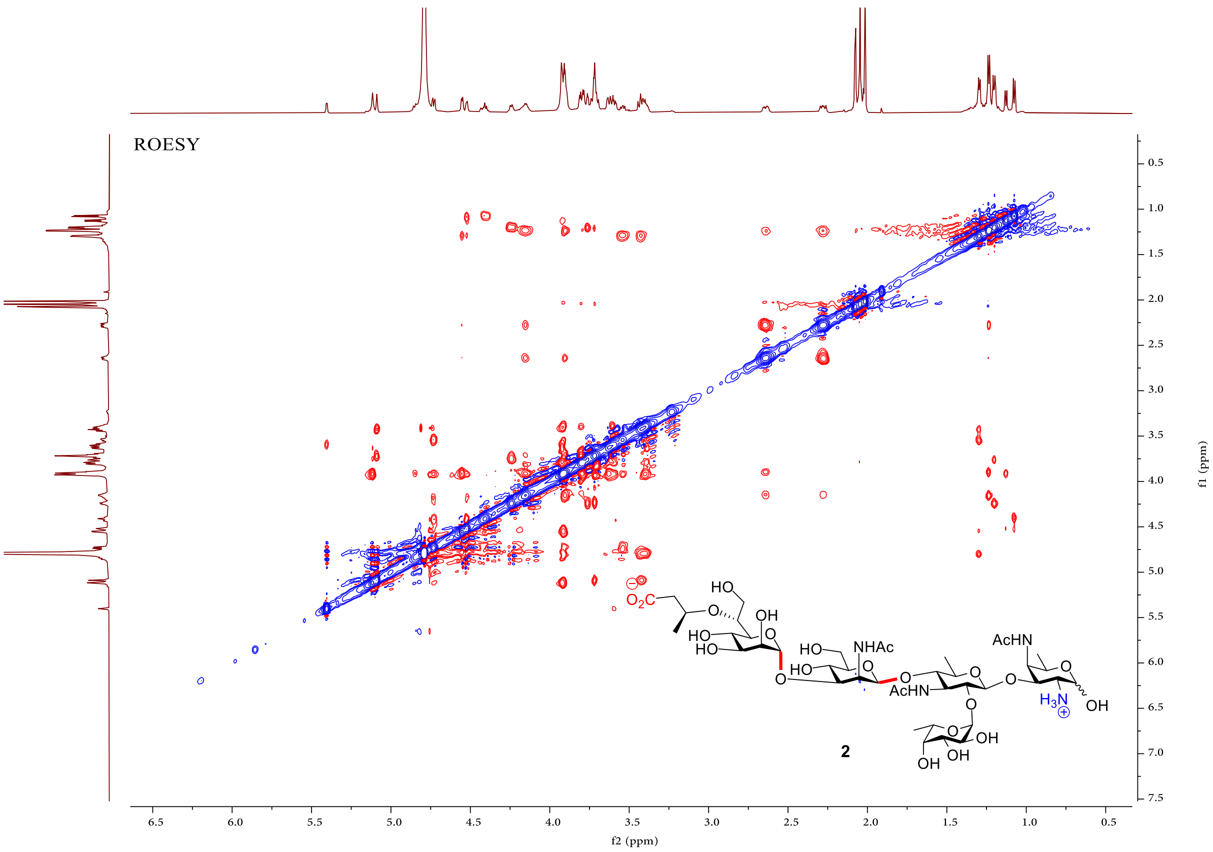
ROESY
f1 (ppm)
f2 (ppm)
HO
OH
HO
HO
HO
NHAc
HO
AcHN
AcHN
H3N
OH
OH
OH
OH
2

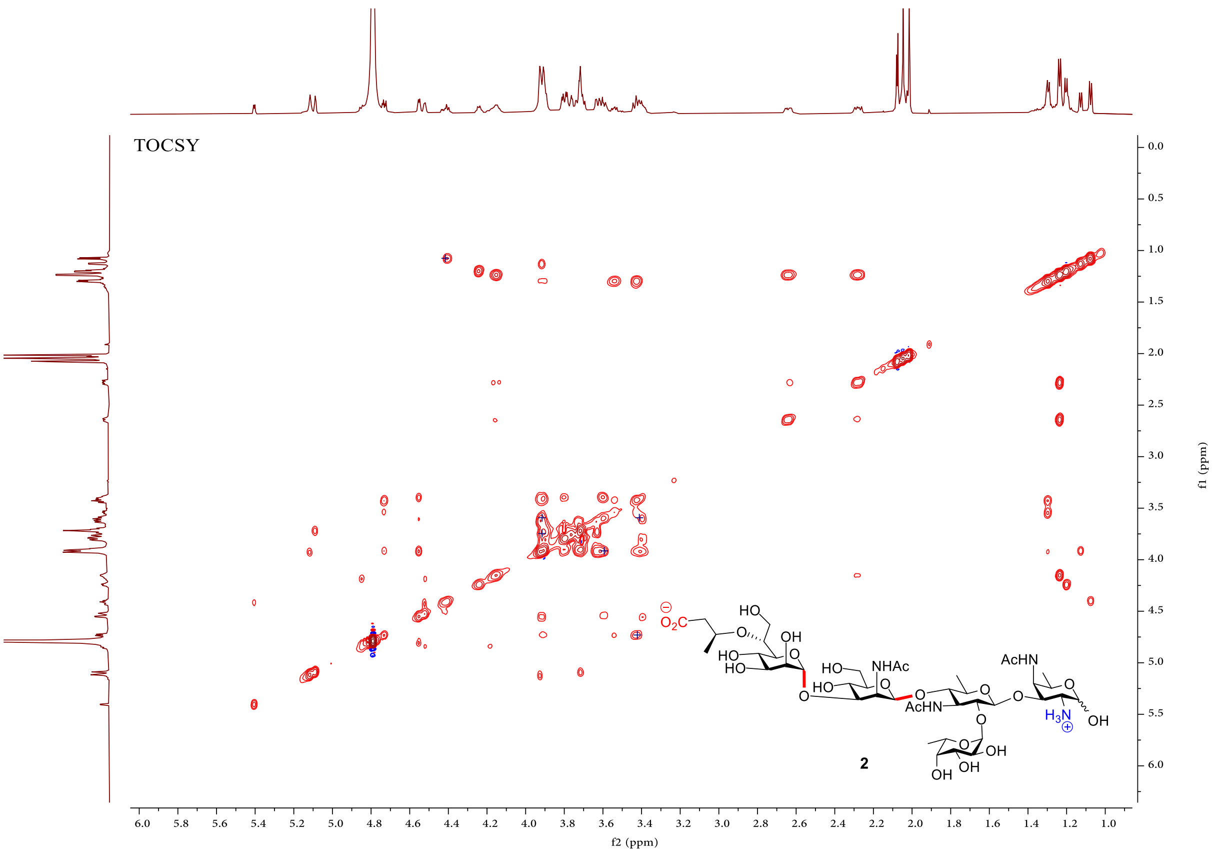
TOCSY
f1 (ppm)
f2 (ppm)
HO
OH
HO
HO
HO
NHAc
HO
AcHN
AcHN
H3N
OH
OH
OH
OH
2

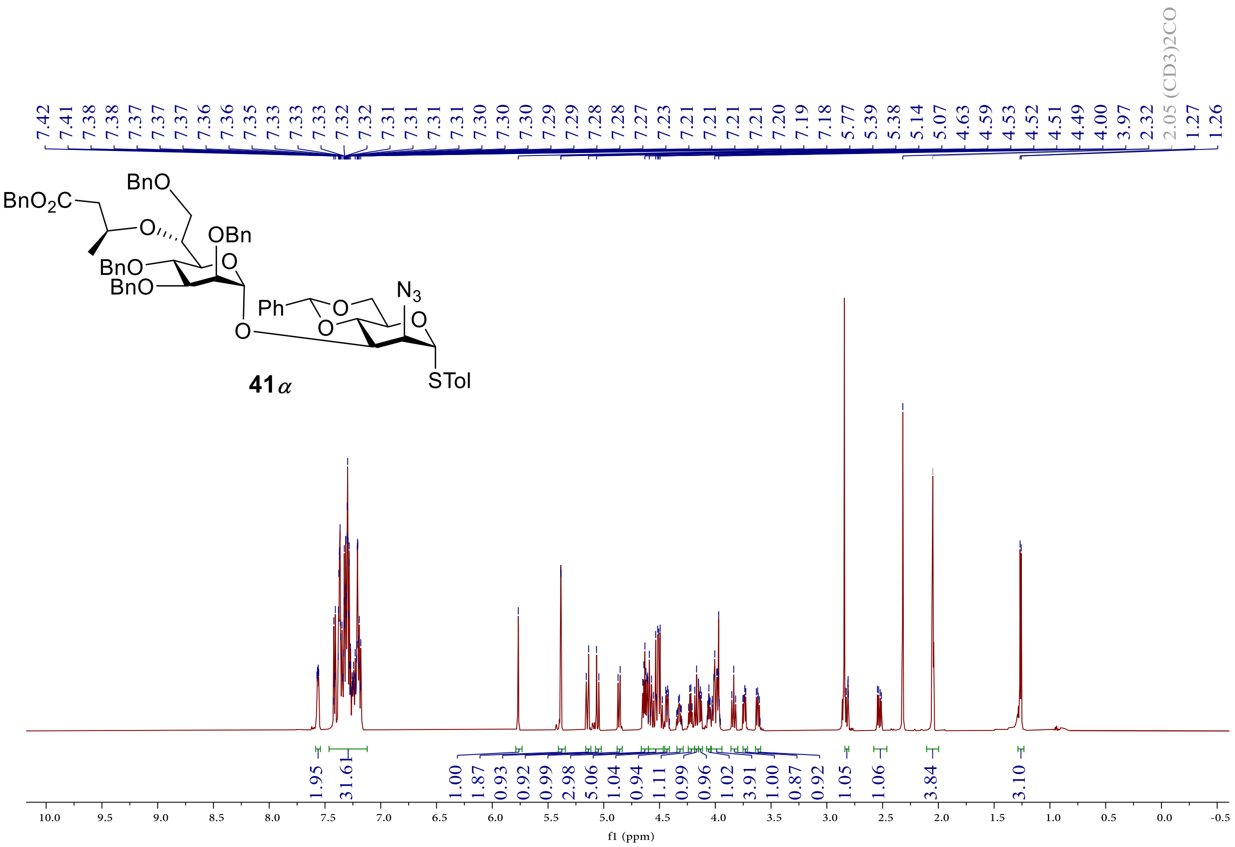
7.42 7.41 7.38 7.38 7.37 7.37 7.37 7.36 7.36 7.35 7.33 7.33 7.33 7.32 7.32 7.31 7.31 7.31 7.30 7.30 7.30 7.29 7.29 7.28 7.28 7.27 7.23 7.21 7.21 7.21 7.21 7.20 7.19 7.18 5.77 5.39 5.38 5.14 5.07 4.63 4.59 4.53 4.52 4.51 4.49 4.00 3.97 2.32 2.05 (CD3)2CO 1.27 1.26
BnO
BnO_2C
O
OBn
BnO
BnO
O
Ph
O
O
N_3
O
O
STol
41α
1.95 31.61 1.00 1.87 0.93 0.92 0.99 2.98 5.06 1.04 0.94 1.11 0.99 0.96 1.02 3.91 1.00 0.87 0.92 1.05 1.06 3.84 3.10
10.0 9.5 9.0 8.5 8.0 7.5 7.0 6.5 6.0 5.5 5.0 4.5 4.0 3.5 3.0 2.5 2.0 1.5 1.0 0.5 0.0 -0.5
f1 (ppm)

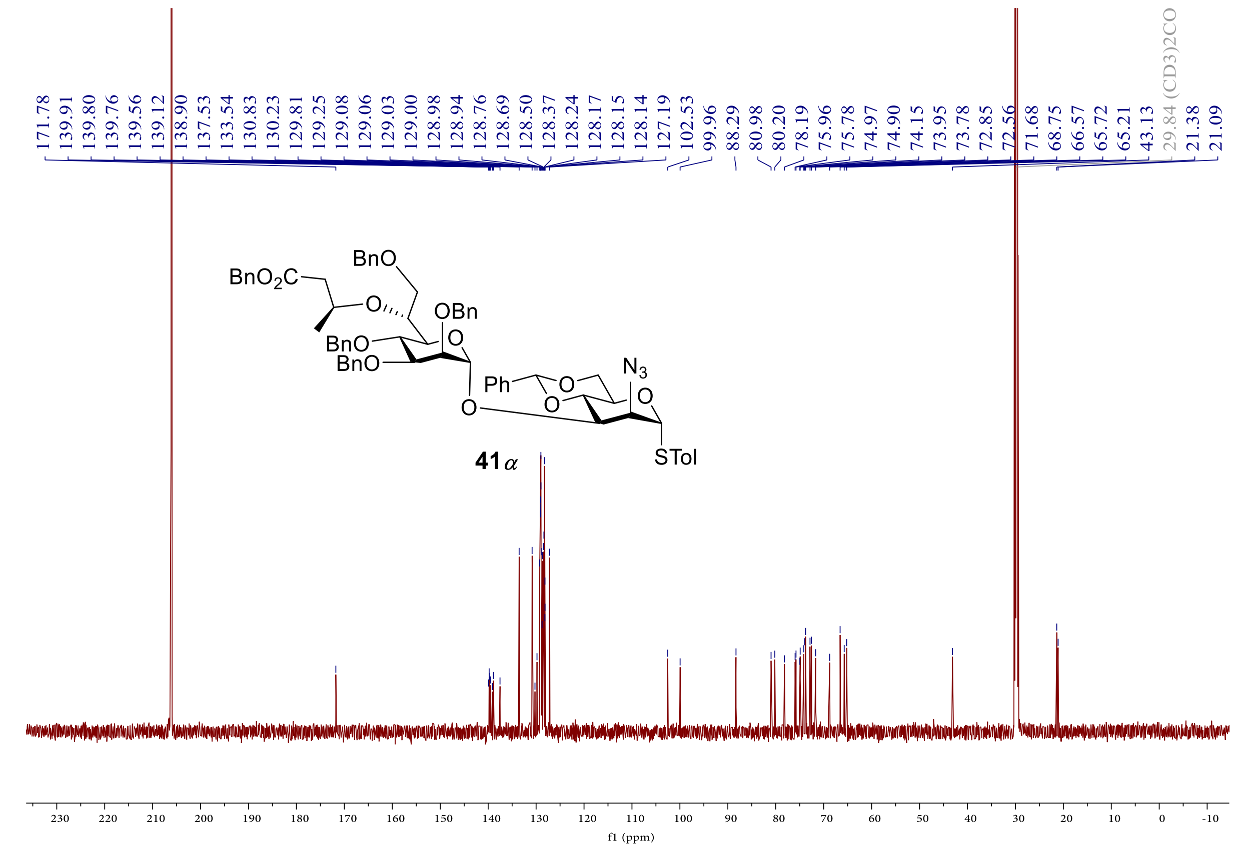
171.78 139.91 139.80 139.76 139.56 139.12 138.90 137.53 133.54 130.83 130.23 129.81 129.25 129.08 129.06 129.03 129.00 128.98 128.94 128.76 128.69 128.50 128.37 128.24 128.17 128.15 128.14 127.19 102.53 99.96 88.29 80.98 80.20 78.19 75.96 75.78 74.97 74.90 74.15 73.95 73.78 72.85 72.56 71.68 68.75 66.57 65.72 65.21 43.13 29.84 (CD3)2CO 21.38 21.09
BnO
BnO_2C
O
OBn
BnO
BnO
O
Ph
O
O
N_3
O
O
STol
41α
230 220 210 200 190 180 170 160 150 140 130 120 110 100 90 80 70 60 50 40 30 20 10 0 -10
f1 (ppm)

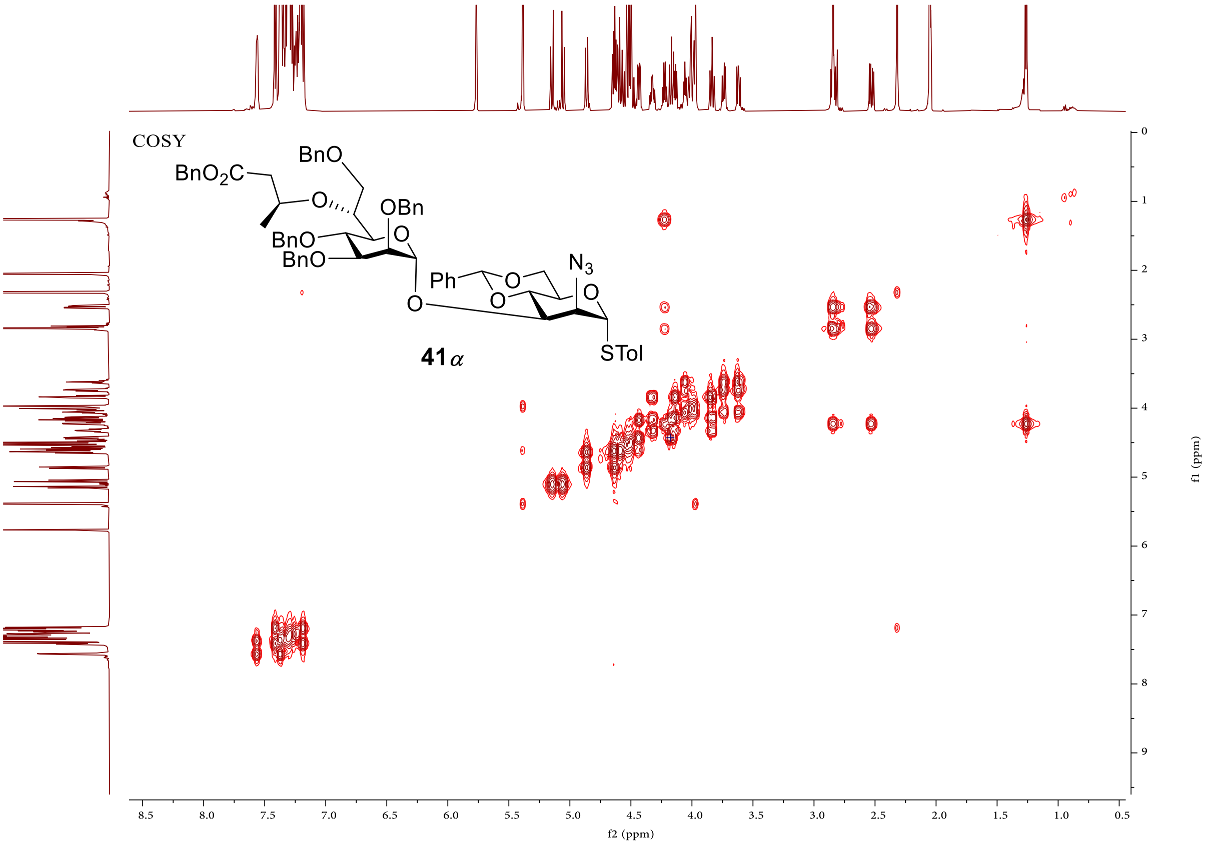
COSY
BnO
BnO2C
OBn
BnO
BnO
Ph
N3
STol
41α
f2 (ppm)
f1 (ppm)

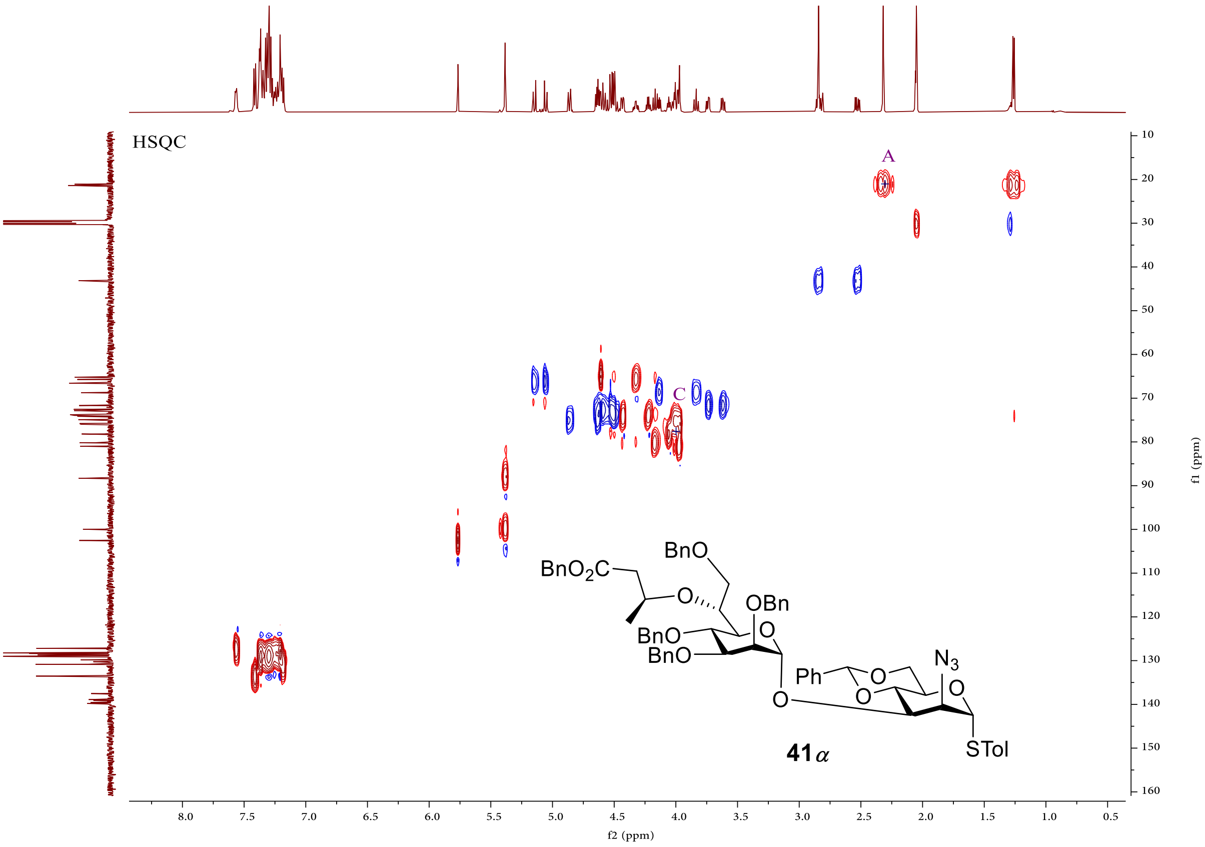
HSQC
A
C
BnO
BnO2C
OBn
BnO
BnO
Ph
N3
STol
41α
f2 (ppm)
f1 (ppm)

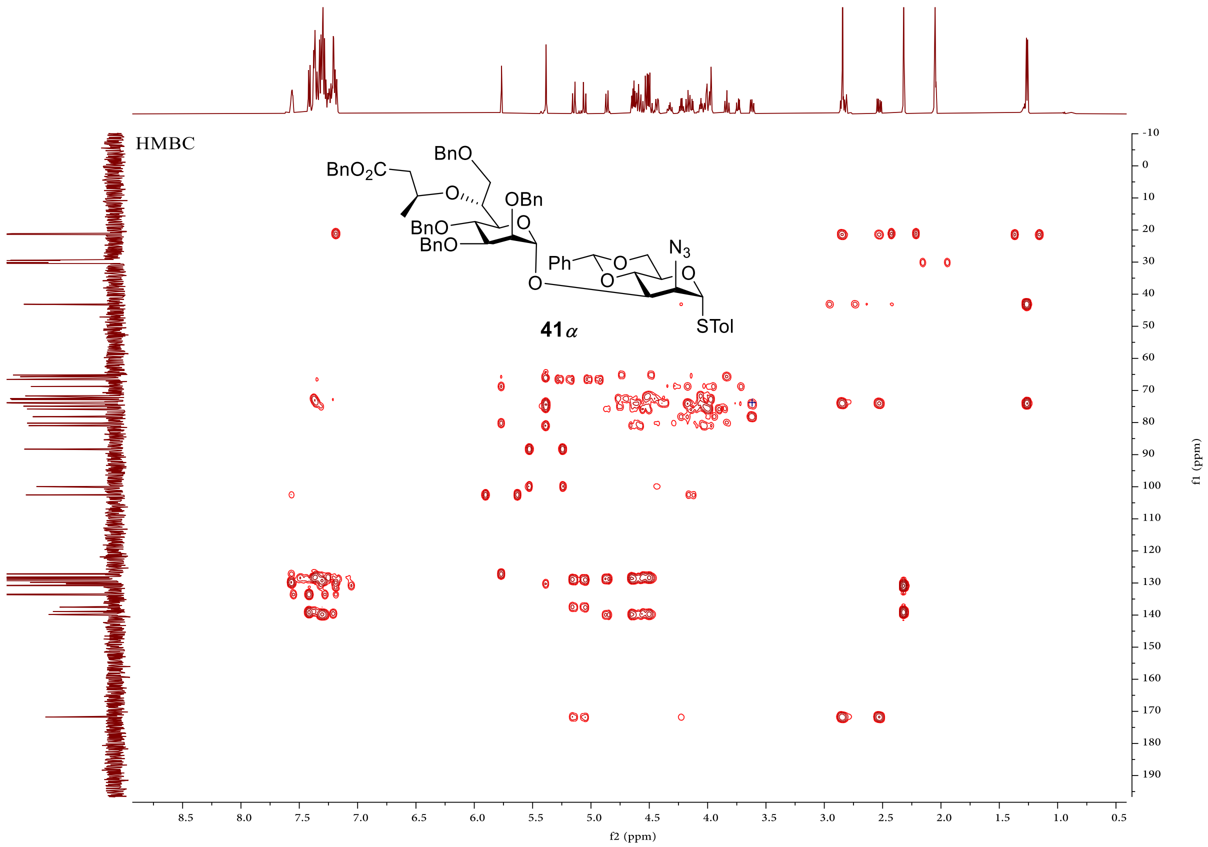

HMBC
BnO
BnO2C
OBn
BnO
BnO
O
Ph
N3
STol
41α
f2 (ppm)
f1 (ppm)

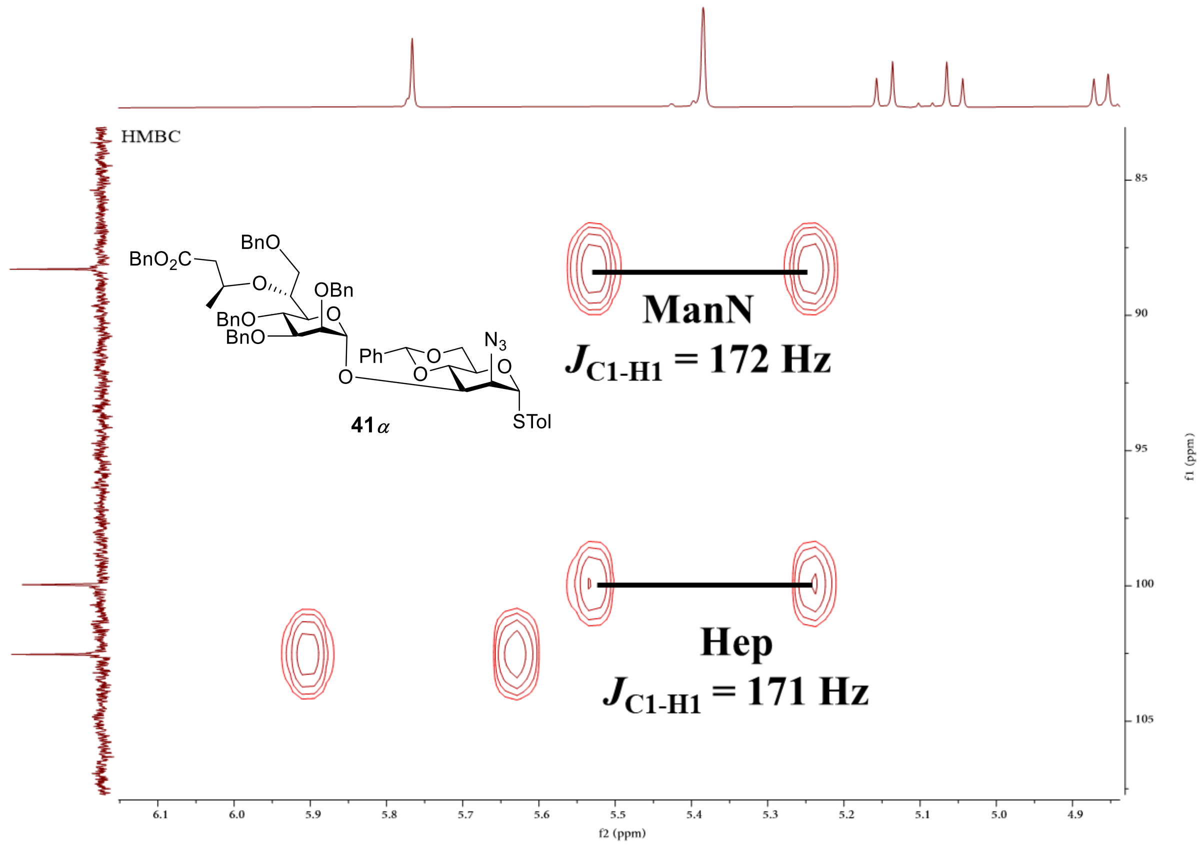

HMBC
BnO
BnO2C
OBn
BnO
BnO
Ph
N3
STol
41α
ManN
J_{C1-H1} = 172 Hz
Hep
J_{C1-H1} = 171 Hz
f2 (ppm)
f1 (ppm)

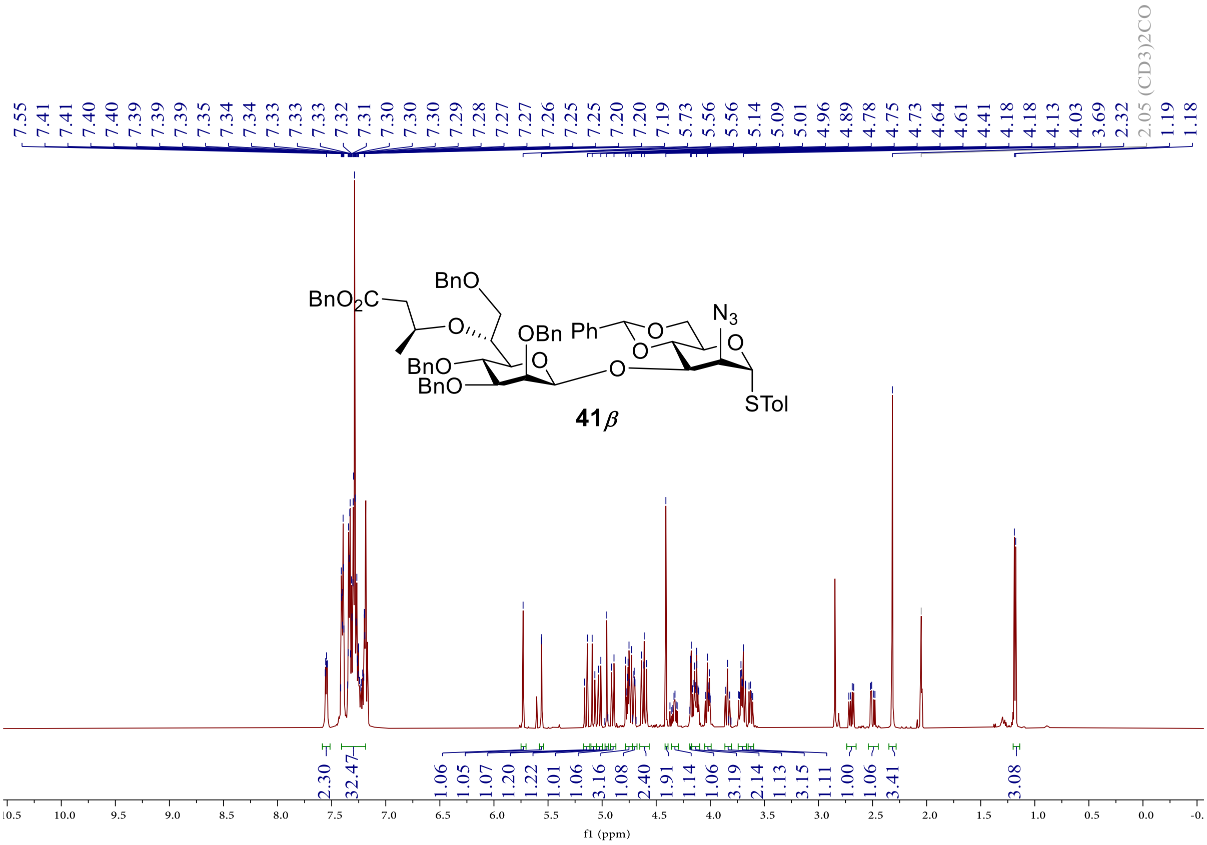
7.55 7.41 7.41 7.40 7.40 7.39 7.39 7.39 7.35 7.34 7.34 7.33 7.33 7.33 7.32 7.31 7.30 7.30 7.29 7.28 7.27 7.27 7.26 7.25 7.25 7.20 7.20 7.19 5.73 5.56 5.56 5.14 5.09 5.01 4.96 4.89 4.78 4.75 4.73 4.64 4.61 4.41 4.18 4.18 4.13 4.03 3.69 2.32 2.05 (CD3)2CO 1.19 1.18
BnO
BnO2C
O
OBn
Ph
O
N3
BnO
BnO
STol
41β
2.30 32.47 1.06 1.05 1.07 1.20 1.22 1.01 1.06 3.16 1.08 2.40 1.91 1.14 1.06 3.19 2.14 1.13 3.15 1.11 1.00 1.06 3.41 3.08
f1 (ppm)

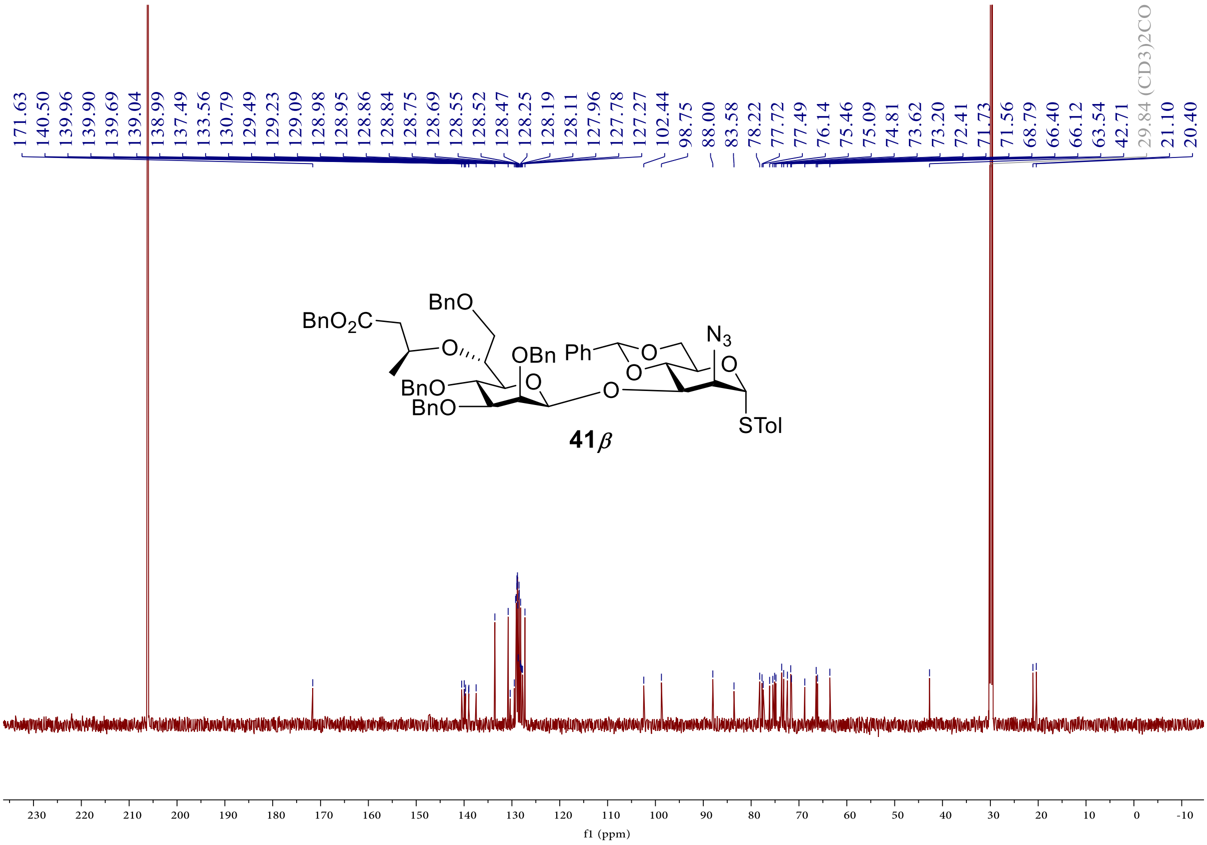
171.63 140.50 139.96 139.90 139.69 139.04 138.99 137.49 133.56 130.79 129.49 129.23 129.09 128.98 128.95 128.86 128.84 128.75 128.69 128.55 128.52 128.47 128.25 128.19 128.11 127.96 127.78 127.27 102.44 98.75 88.00 83.58 78.22 77.72 77.49 76.14 75.46 75.09 74.81 73.62 73.20 72.41 71.73 71.56 68.79 66.40 66.12 63.54 42.71 29.84 (CD3)2CO 21.10 20.40
BnO
BnO2C
O
OBn
Ph
O
N3
BnO
BnO
STol
41β
f1 (ppm)

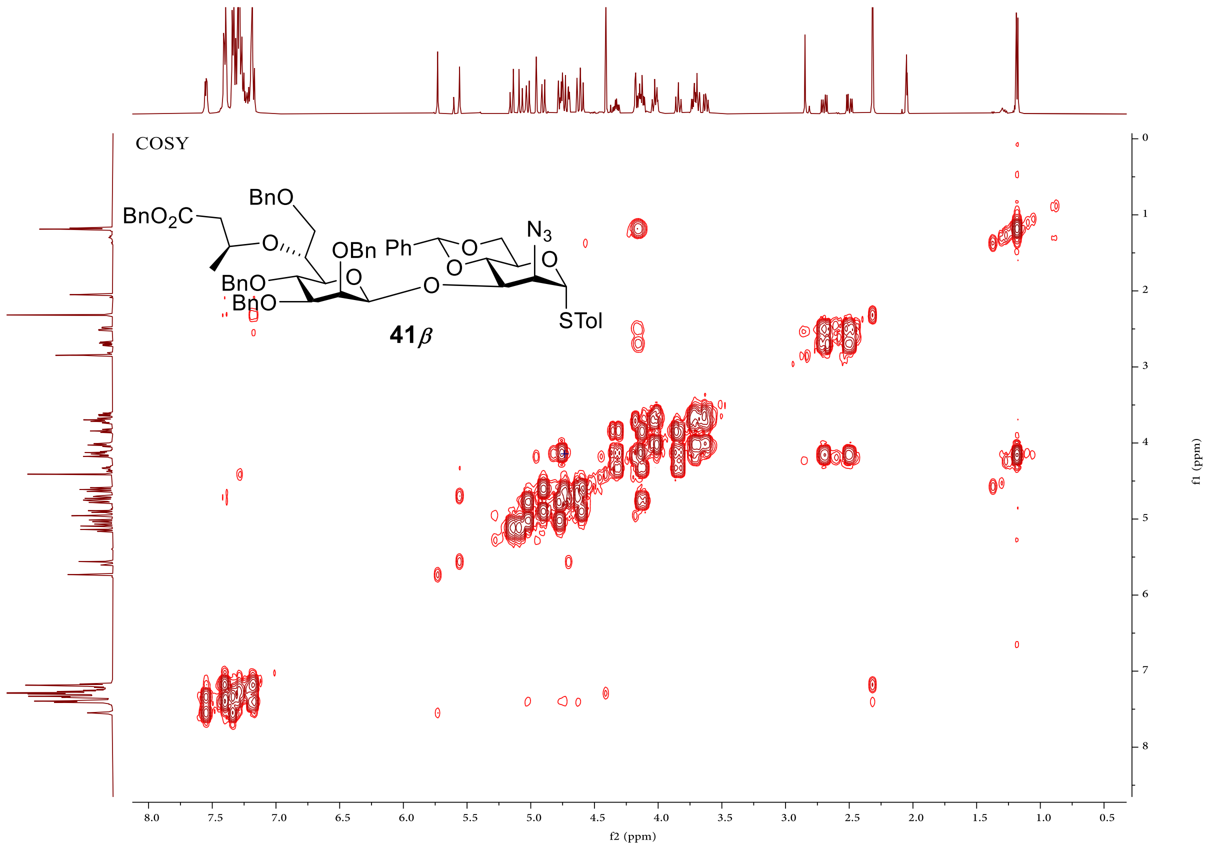
COSY
BnO
BnO_2C
OBn
Ph
N_3
BnO
BnO
O
STol
41β
f2 (ppm)
f1 (ppm)

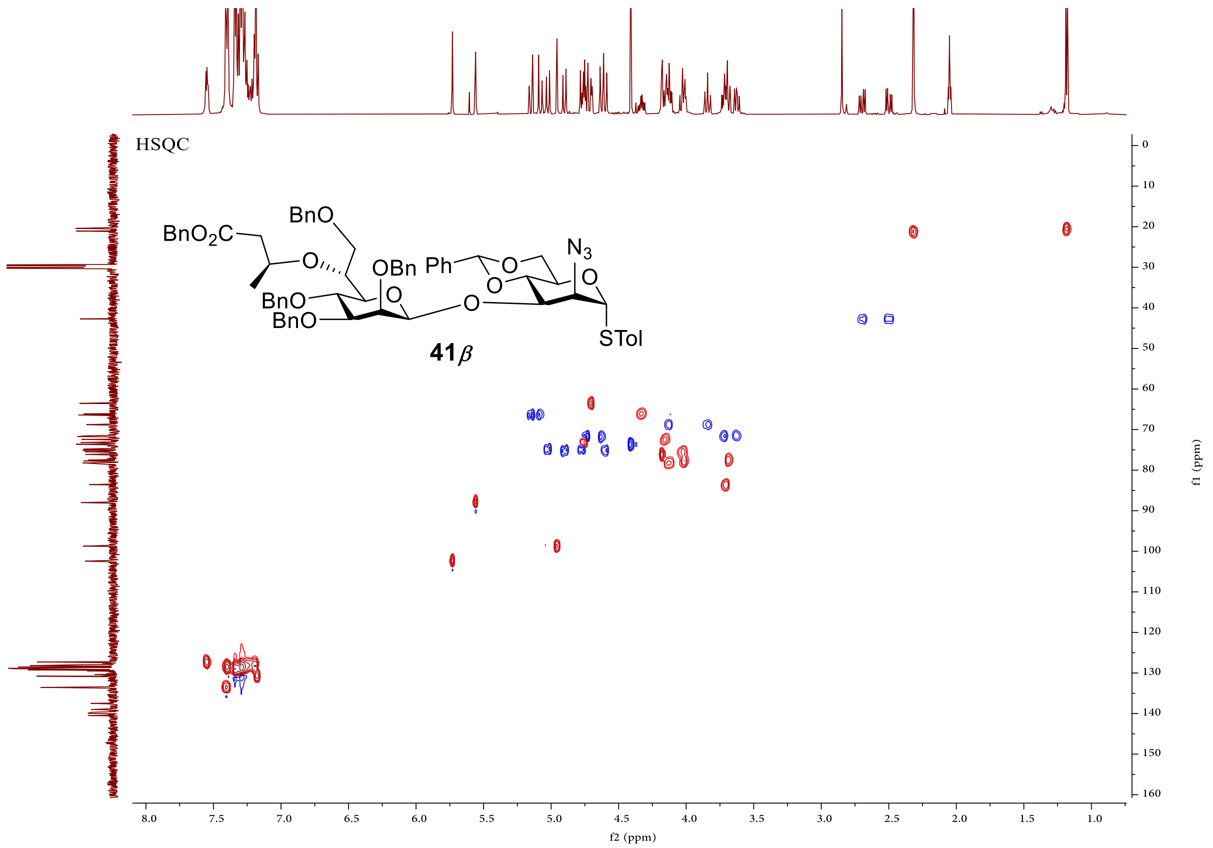
HSQC
BnO
BnO_2C
OBn
Ph
N_3
BnO
BnO
O
STol
41β
f2 (ppm)
f1 (ppm)

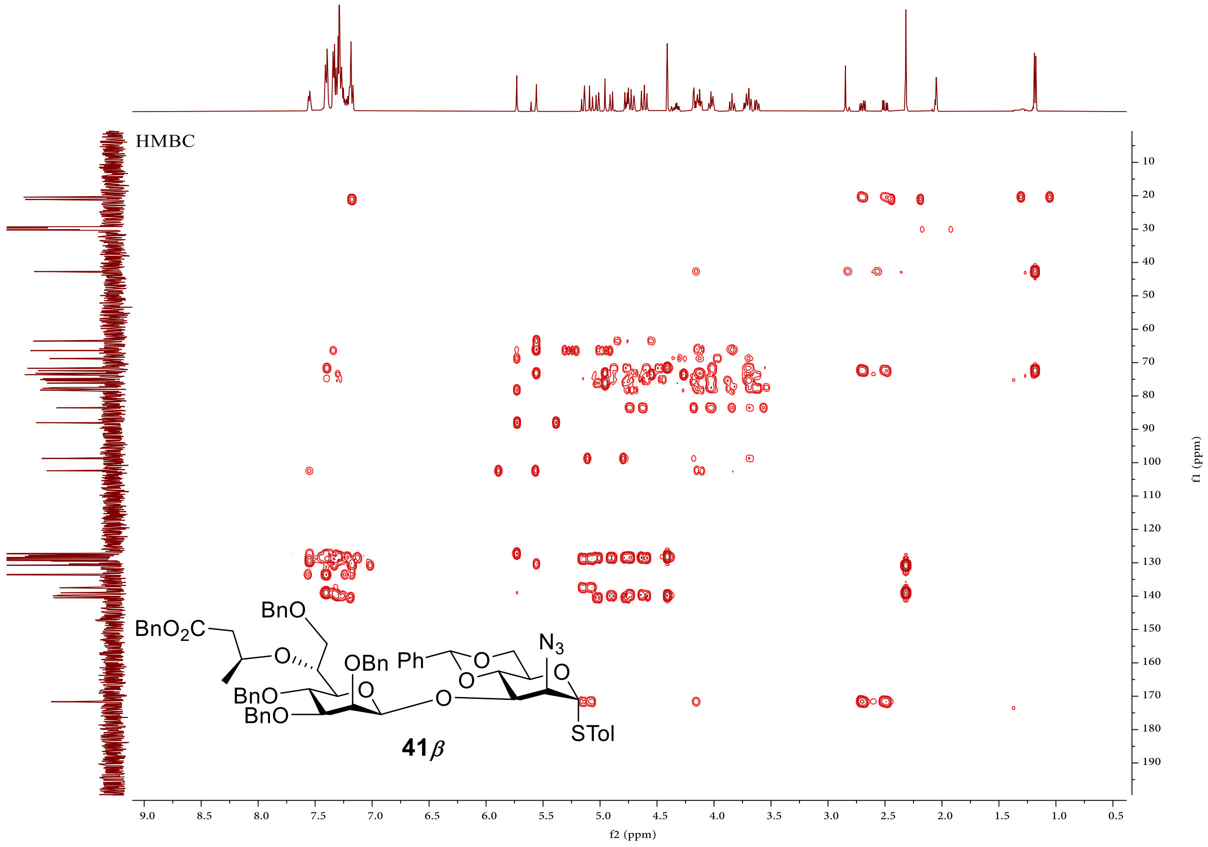
HMBC
BnO
BnO2C
O
OBn
Ph
O
N3
BnO
BnO
41β
STol
f2 (ppm)
f1 (ppm)

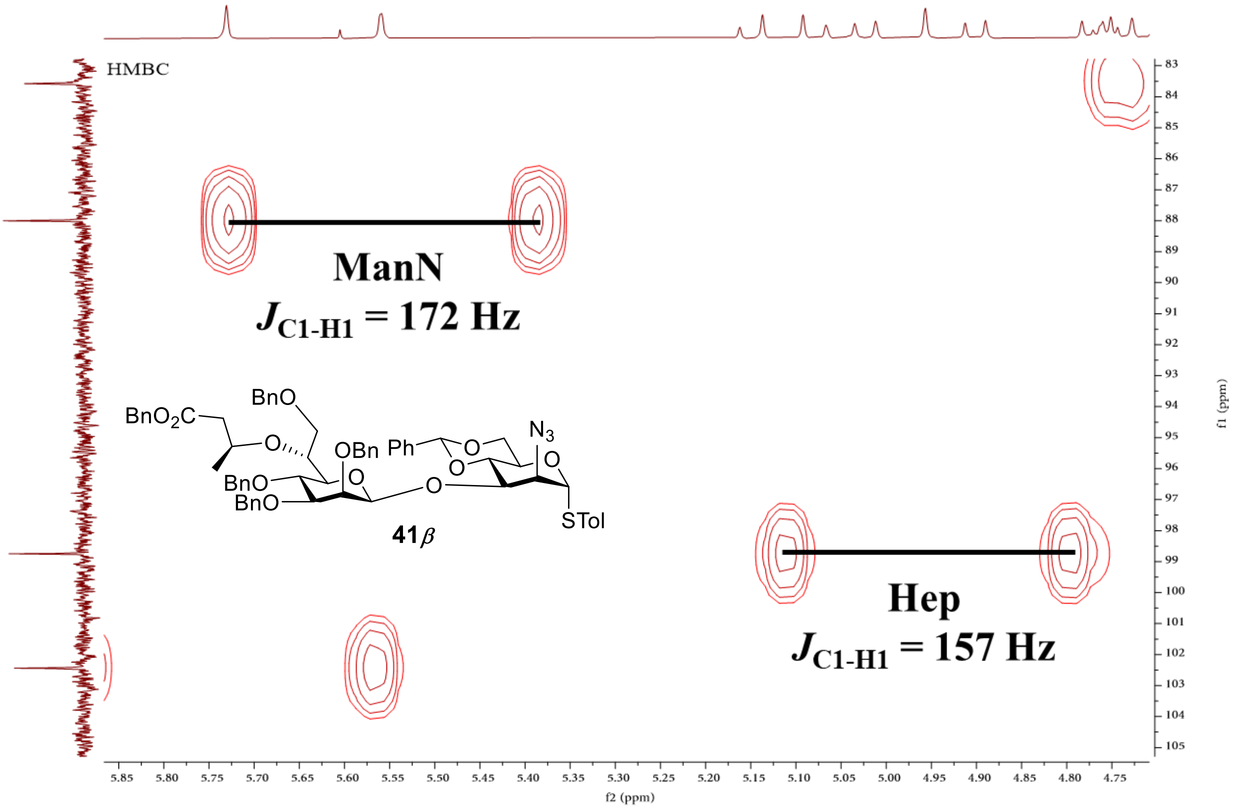
HMBC
ManN
JC1-H1 = 172 Hz
Hep
JC1-H1 = 157 Hz
41β
f2 (ppm)
f1 (ppm)

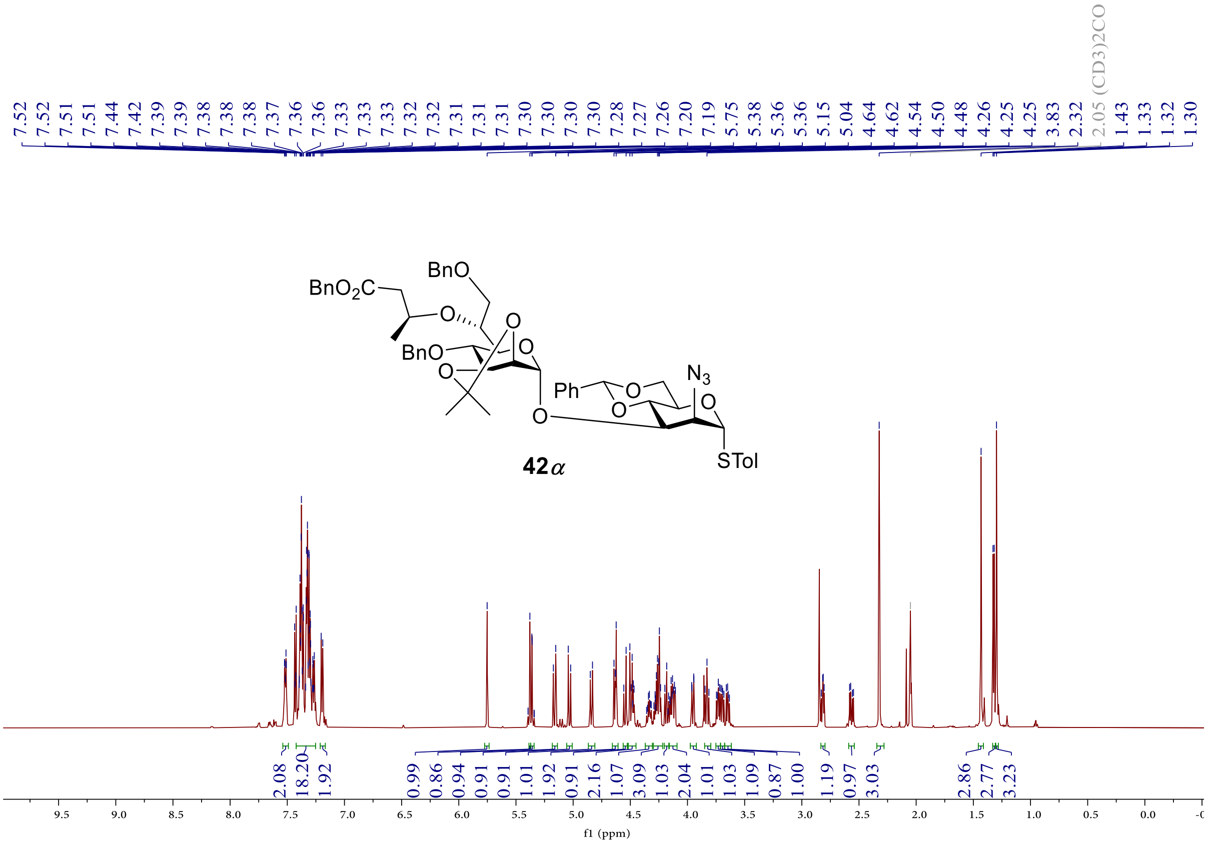
7.52 7.52 7.51 7.51 7.44 7.42 7.39 7.39 7.38 7.38 7.38 7.37 7.36 7.36 7.33 7.33 7.33 7.32 7.32 7.31 7.31 7.31 7.30 7.30 7.30 7.30 7.28 7.27 7.26 7.20 7.19 5.75 5.38 5.36 5.36 5.15 5.04 4.64 4.62 4.54 4.50 4.48 4.26 4.25 4.25 3.83 2.32 2.05 (CD3)2CO 1.43 1.33 1.32 1.30
BnO
BnO2C
BnO
Ph
N3
STol
42α
2.08 18.20 1.92 0.99 0.86 0.94 0.91 0.91 1.01 1.92 0.91 2.16 1.07 3.09 1.03 2.04 1.01 1.03 1.09 0.87 1.00 1.19 0.97 3.03 2.86 2.77 3.23
f1 (ppm)

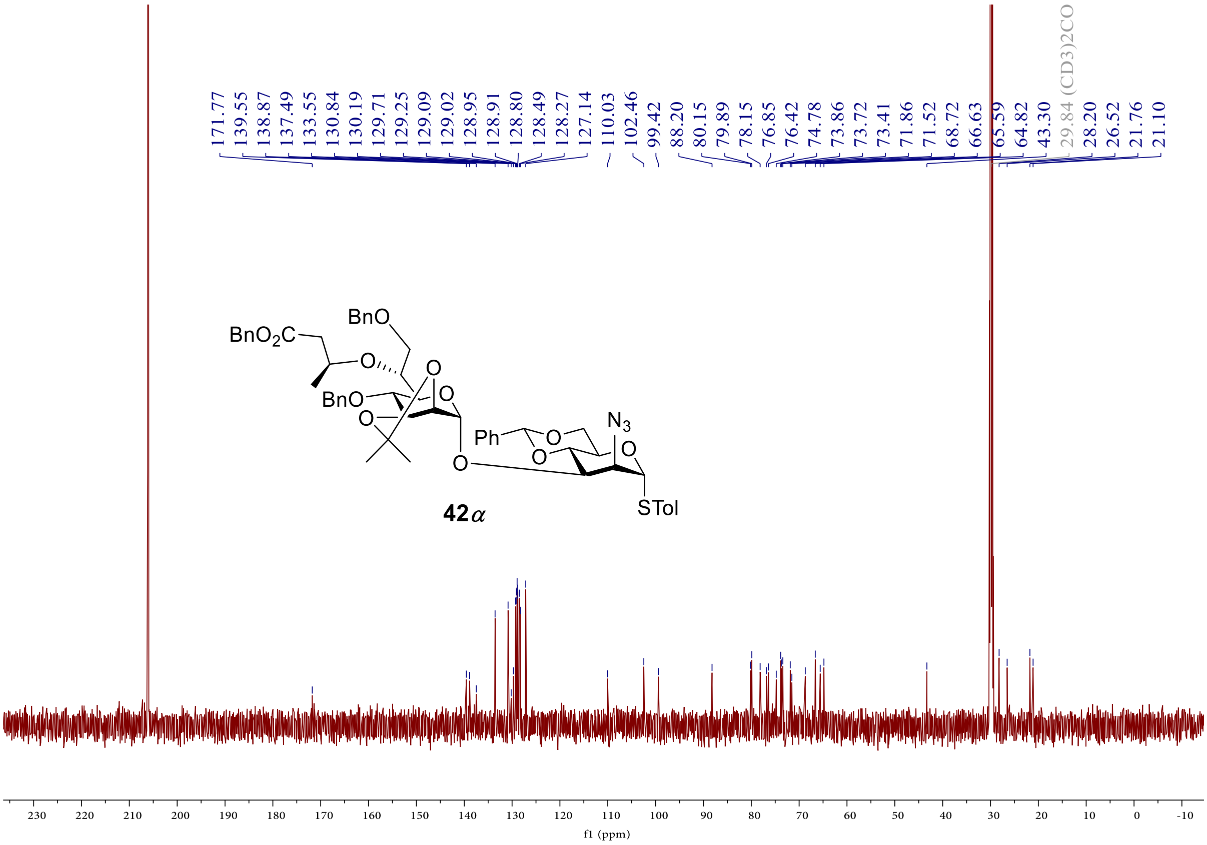
171.77 139.55 138.87 137.49 133.55 130.84 130.19 129.71 129.25 129.09 129.02 128.95 128.91 128.80 128.49 128.27 127.14 110.03 102.46 99.42 88.20 80.15 79.89 78.15 76.85 76.42 74.78 73.86 73.72 73.41 71.86 71.52 68.72 66.63 65.59 64.82 43.30 29.84 (CD3)2CO 28.20 26.52 21.76 21.10
BnO
BnO2C
BnO
Ph
N3
STol
42α
f1 (ppm)

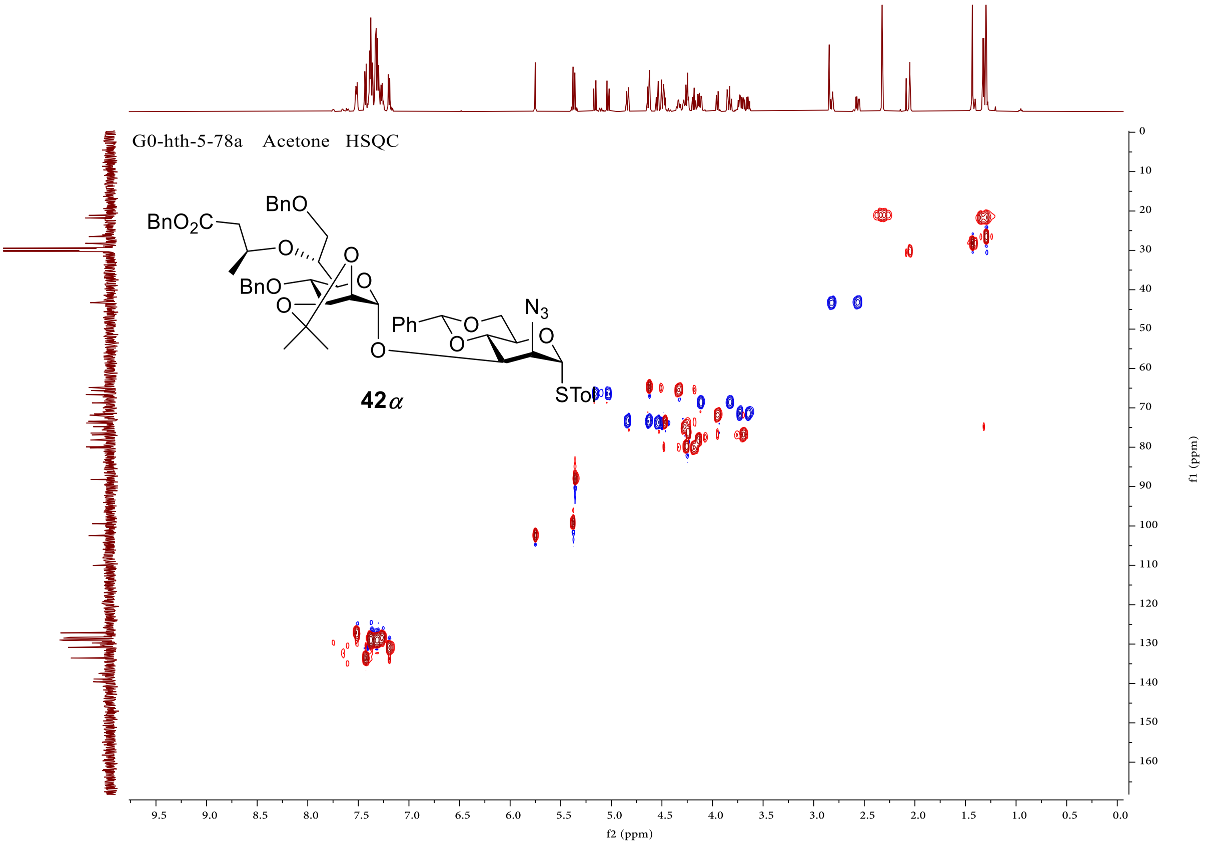
G0-hth-5-78a Acetone HSQC
BnO
BnO2C
O
BnO
Ph
N3
STol
42α
f2 (ppm)
f1 (ppm)

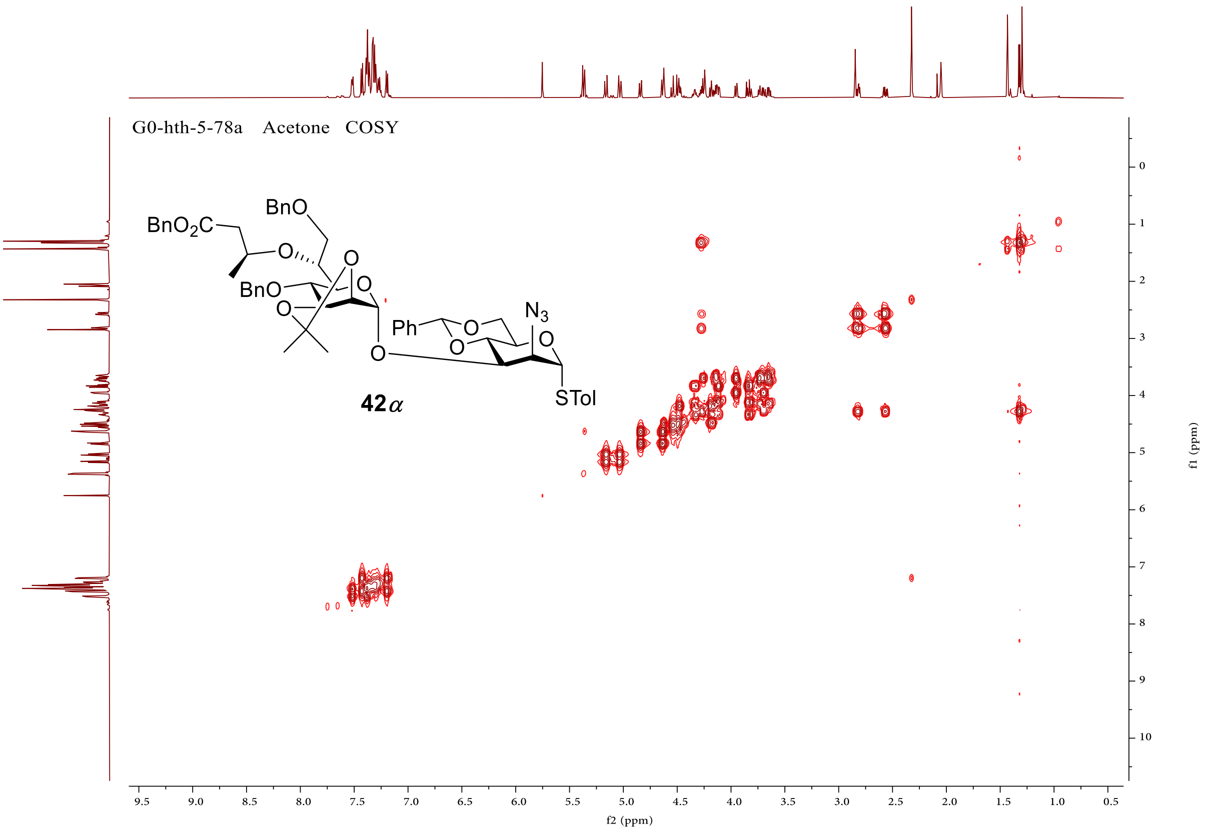
G0-hth-5-78a Acetone COSY
BnO
BnO2C
O
BnO
Ph
N3
STol
42α
f2 (ppm)
f1 (ppm)

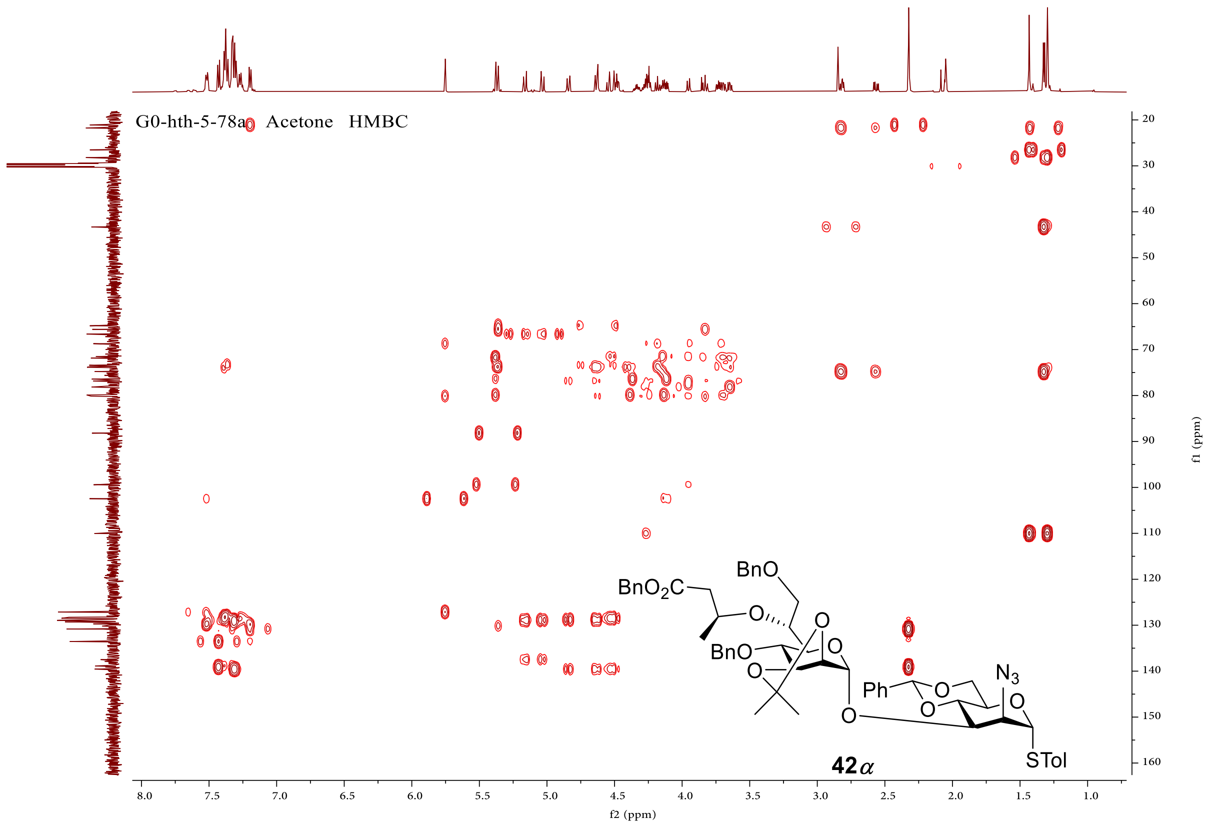
G0-hth-5-78a Acetone HMBC
BnO
BnO2C
O
BnO
O
O
O
Ph
O
O
N3
O
O
STol
42α
f2 (ppm)
f1 (ppm)

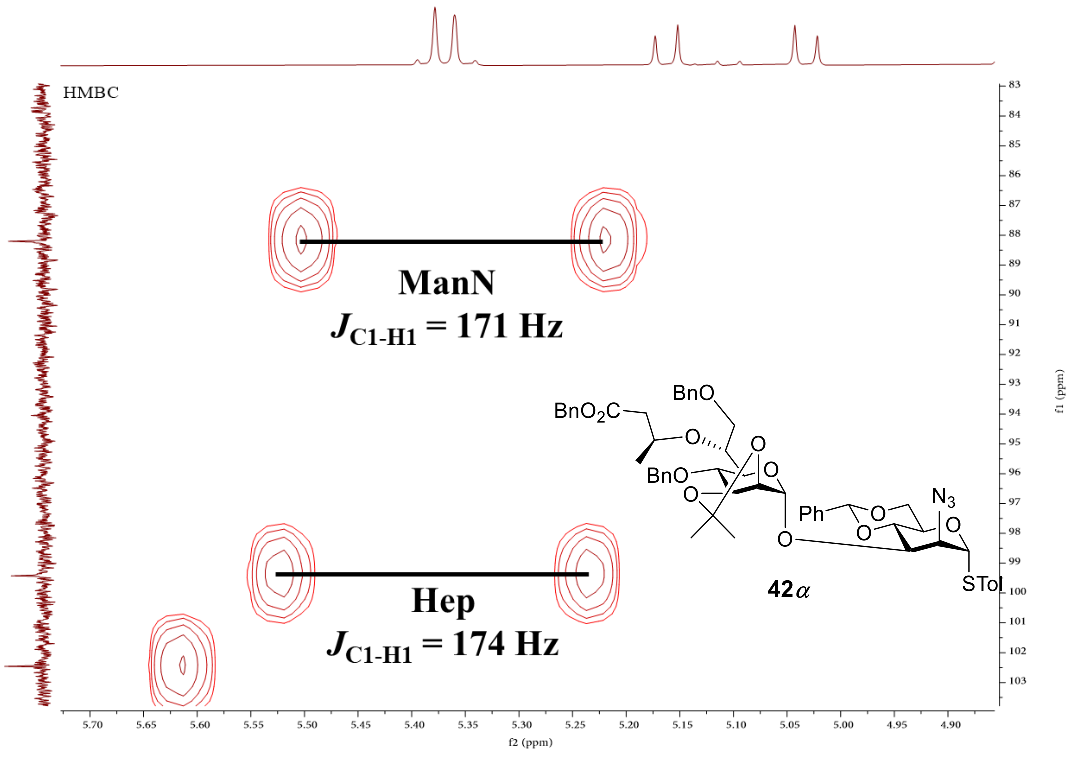
HMBC
ManN
JC1-H1 = 171 Hz
Hep
JC1-H1 = 174 Hz
BnO
BnO2C
BnO
Ph
N3
STol
42α
f2 (ppm)
f1 (ppm)

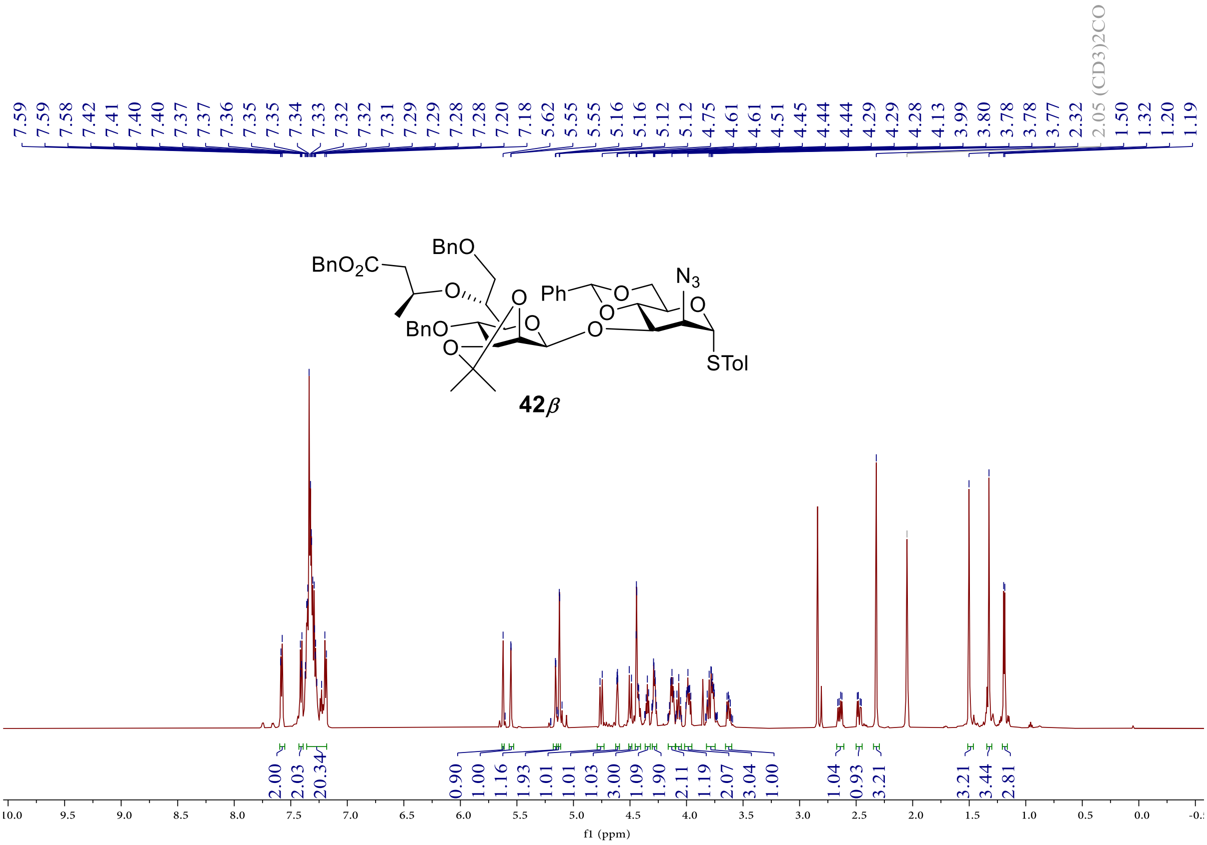
7.59 7.59 7.58 7.42 7.41 7.40 7.37 7.37 7.36 7.35 7.35 7.34 7.33 7.32 7.32 7.31 7.29 7.29 7.28 7.28 7.20 7.18 5.62 5.55 5.55 5.16 5.16 5.12 5.12 4.75 4.61 4.61 4.51 4.45 4.44 4.44 4.29 4.29 4.28 4.13 3.99 3.80 3.78 3.78 3.77 2.32 2.05 (CD3)2CO 1.50 1.32 1.20 1.19
BnO
BnO2C
O
Ph
O
N3
O
BnO
O
O
STol
O
42β
2.00 2.03 20.34 0.90 1.00 1.16 1.93 1.01 1.01 1.03 3.00 1.09 1.90 2.11 1.19 2.07 3.04 1.00 1.04 0.93 3.21 3.21 3.44 2.81
f1 (ppm)

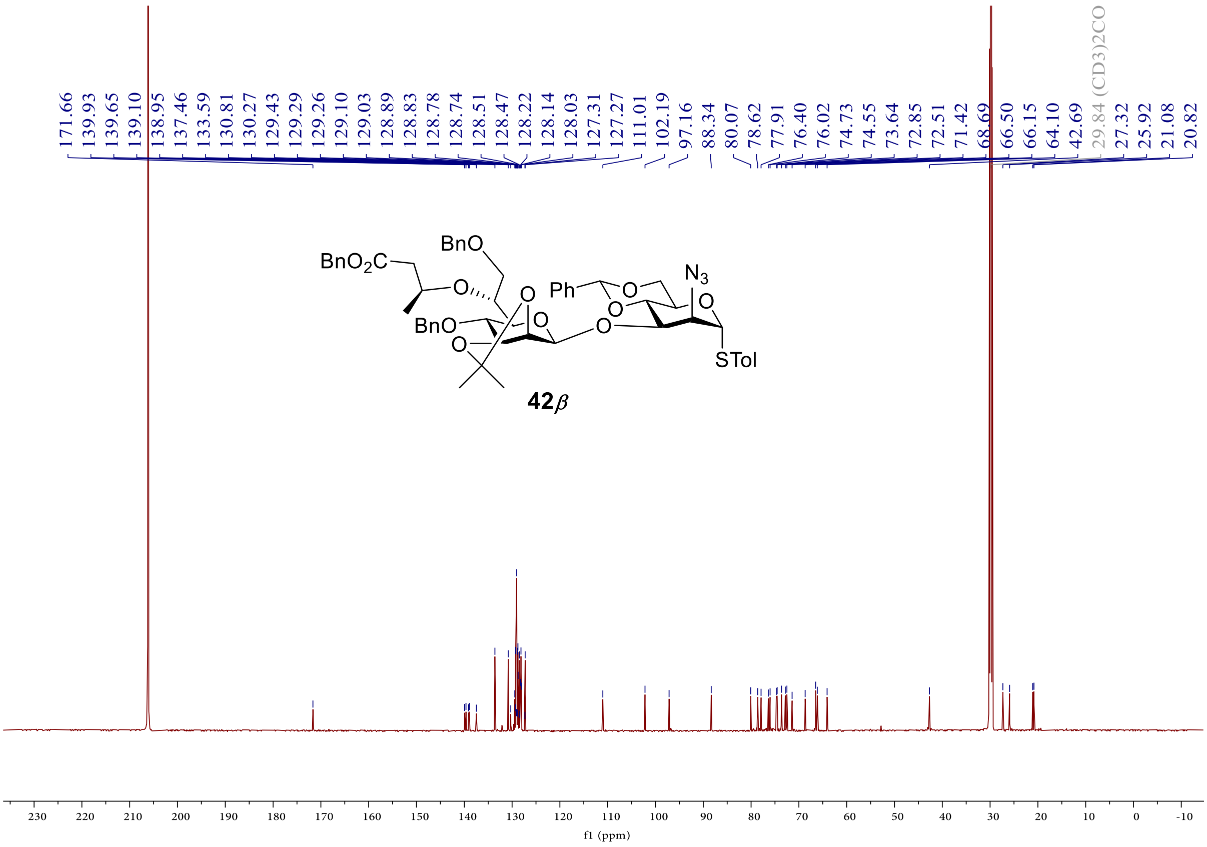
171.66 139.93 139.65 139.10 138.95 137.46 133.59 130.81 130.27 129.43 129.29 129.26 129.10 129.03 128.89 128.83 128.78 128.74 128.51 128.47 128.22 128.14 128.03 127.31 127.27 111.01 102.19 97.16 88.34 80.07 78.62 77.91 76.40 76.02 74.73 74.55 73.64 72.85 72.51 71.42 68.69 66.50 66.15 64.10 42.69 29.84 (CD3)2CO 27.32 25.92 21.08 20.82
BnO
BnO2C
O
Ph
O
N3
O
BnO
O
O
STol
O
42β
f1 (ppm)

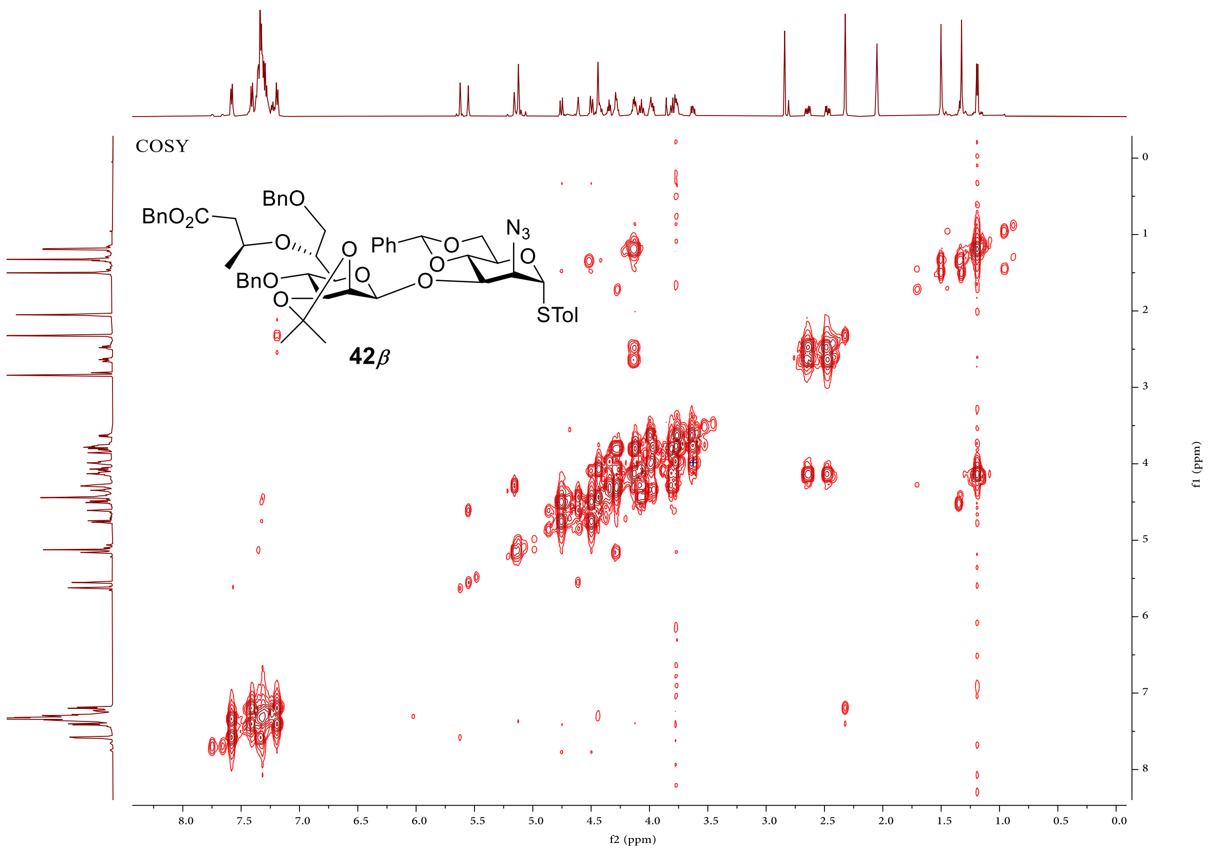
COSY
BnO
BnO$_2$C
O
Ph
O
N$_3$
BnO
O
STol
42β
f2 (ppm)
f1 (ppm)

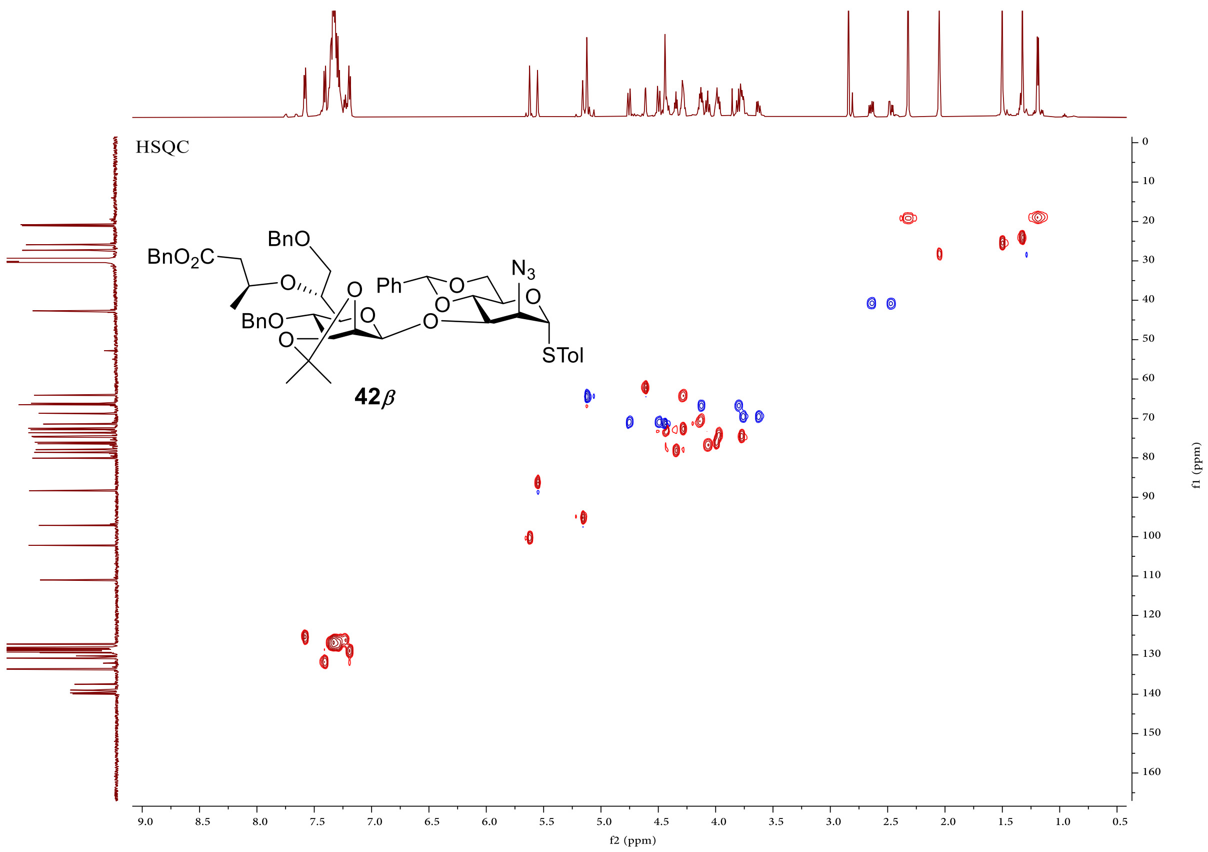
HSQC
BnO
BnO$_2$C
O
Ph
O
N$_3$
BnO
O
STol
42β
f2 (ppm)
f1 (ppm)

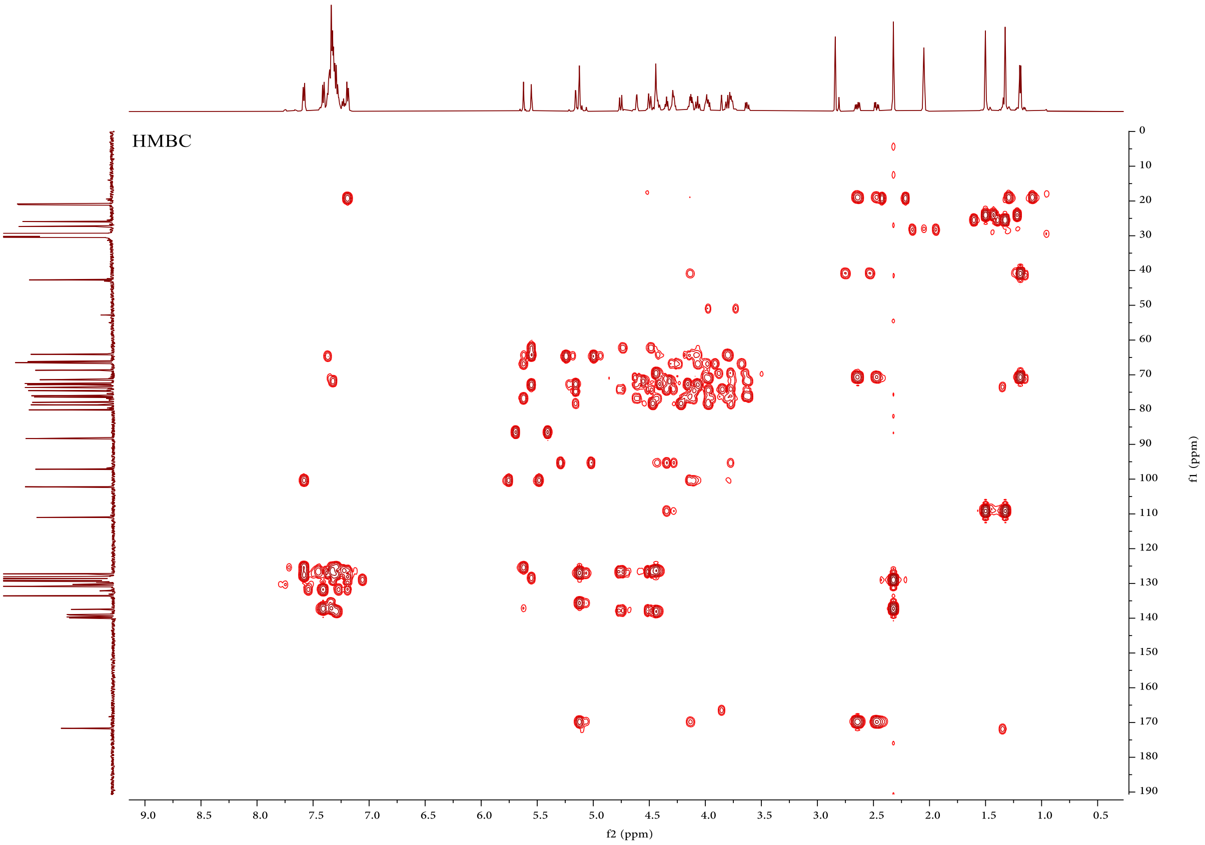
HMBC
f2 (ppm)
f1 (ppm)

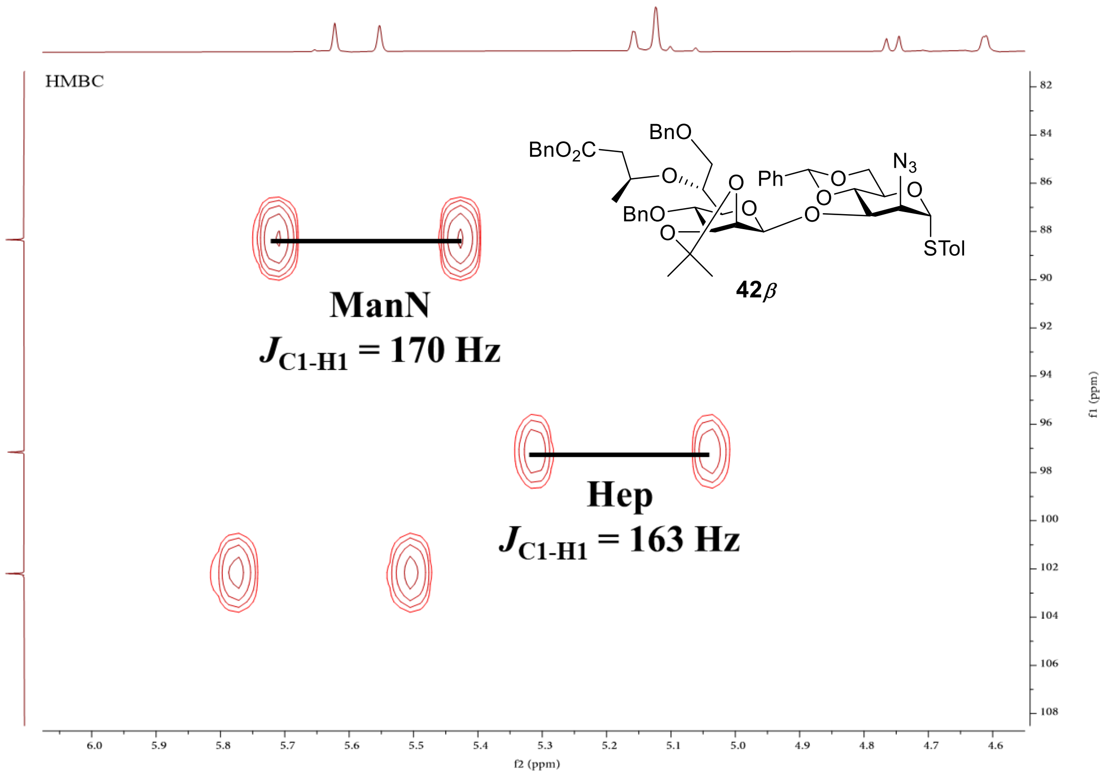
HMBC
ManN
J_{C1-H1} = 170 Hz
Hep
J_{C1-H1} = 163 Hz
BnO
BnO_2C
BnO
Ph
N_3
STol
42*β*
f2 (ppm)
f1 (ppm)

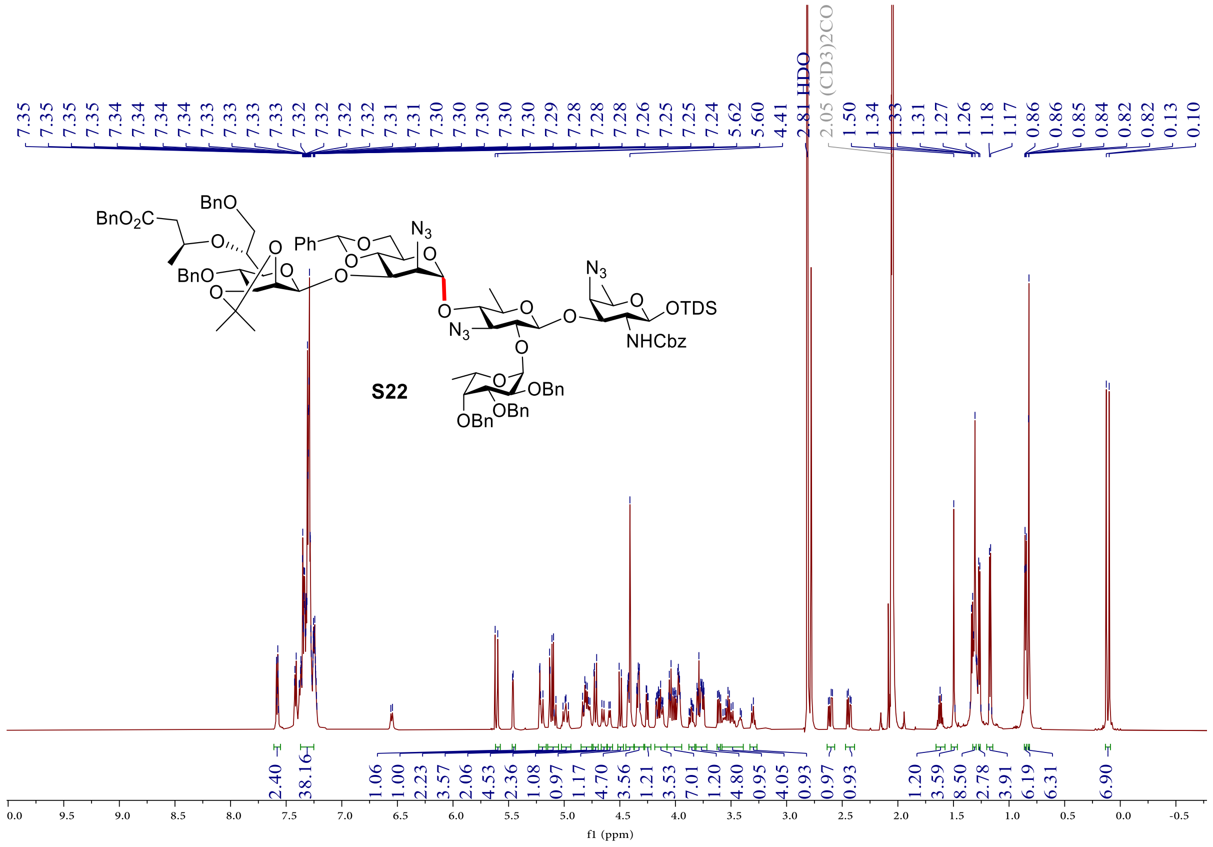

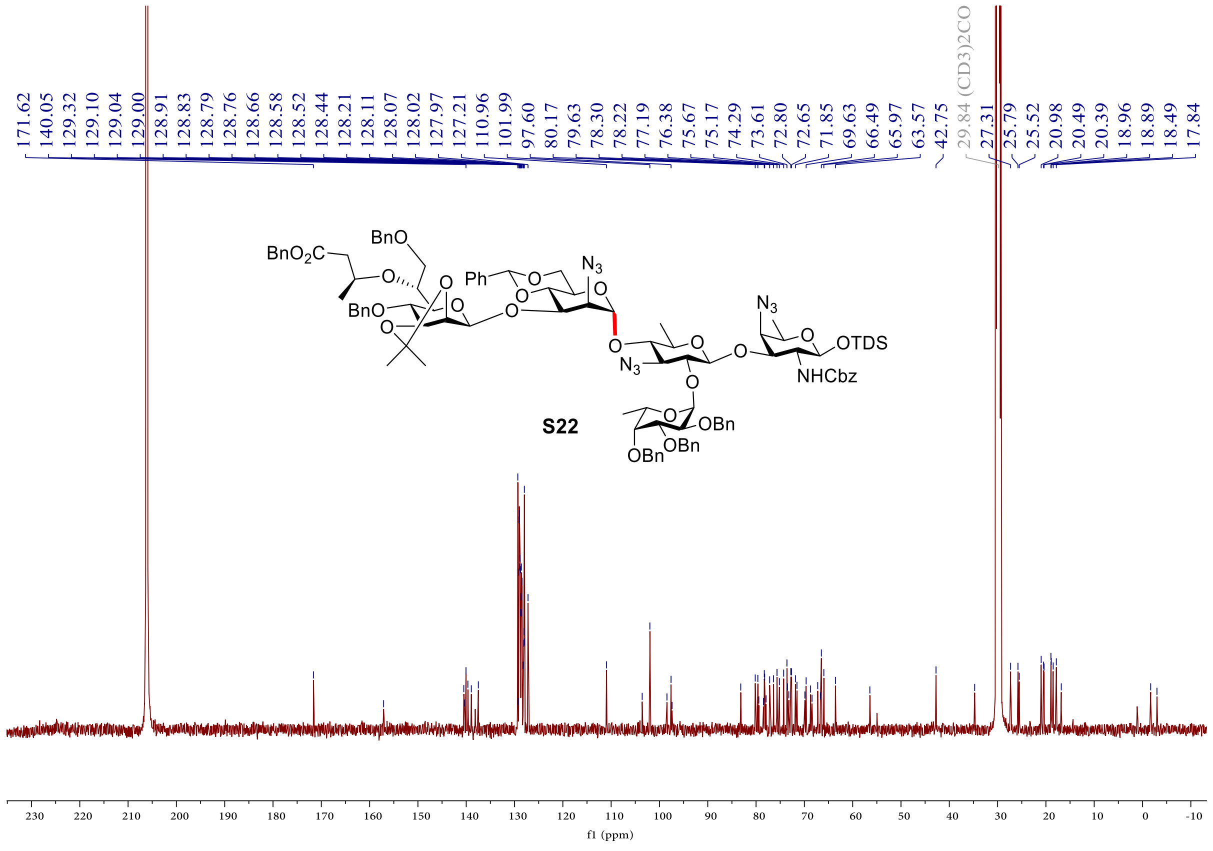

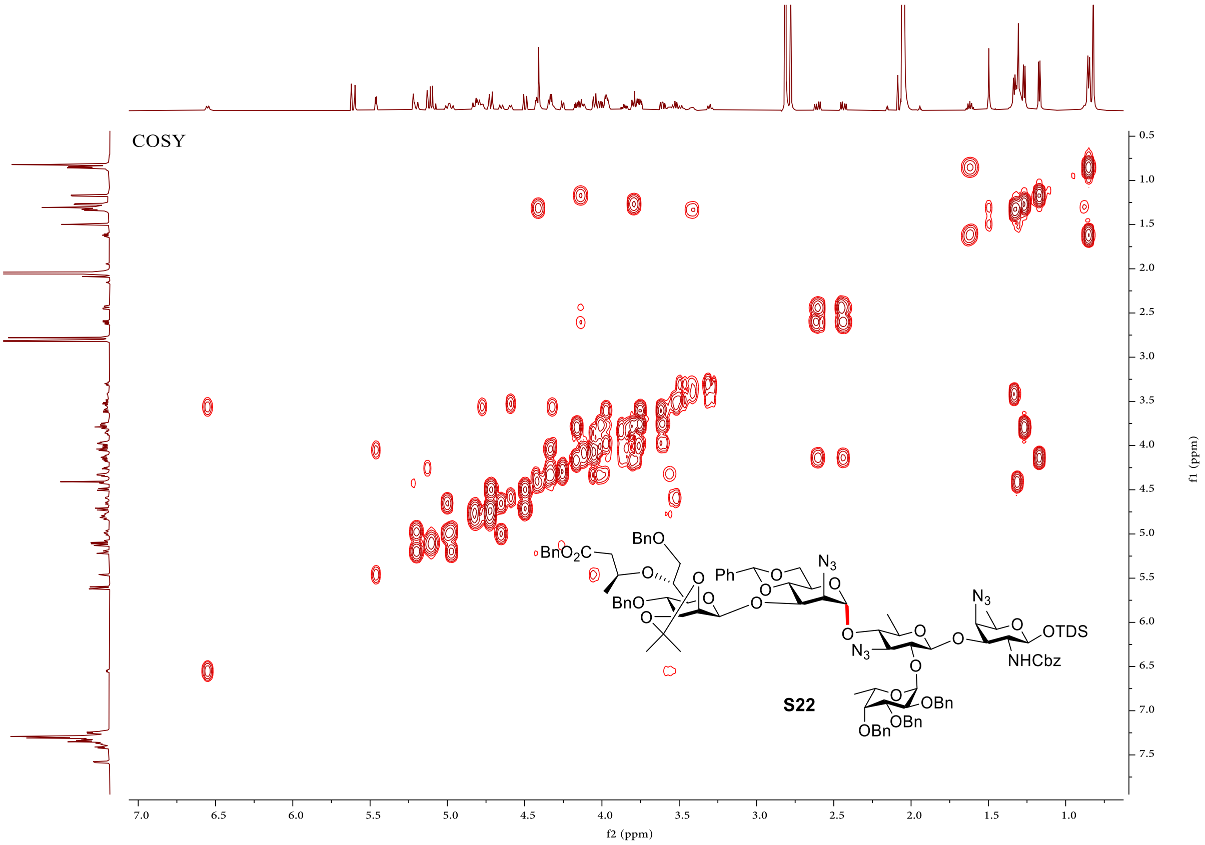
COSY
f2 (ppm)
f1 (ppm)
BnO
BnO2C
O
Ph
N3
BnO
OTDS
NHCbz
OBn
S22

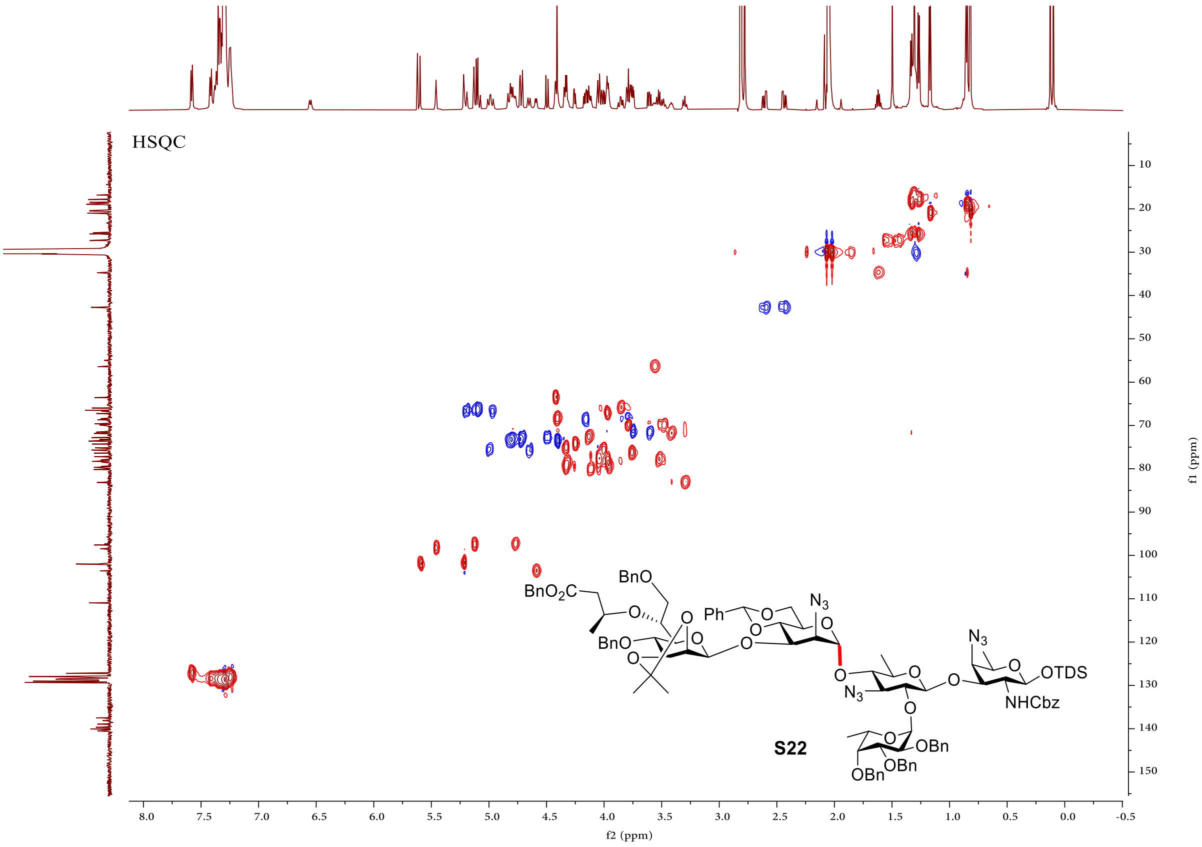
HSQC
f2 (ppm)
f1 (ppm)
BnO
BnO2C
O
Ph
N3
BnO
OTDS
NHCbz
OBn
S22

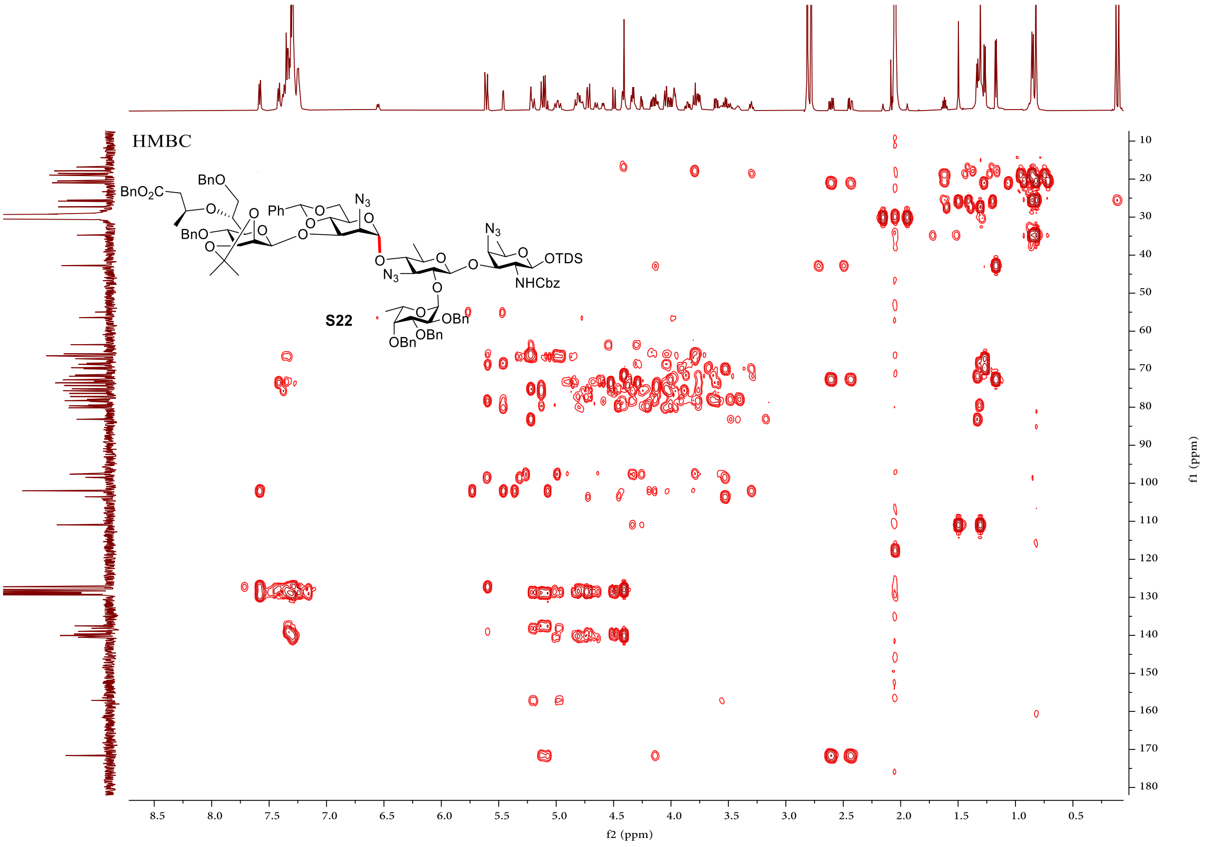
HMBC
S22
f2 (ppm)
f1 (ppm)

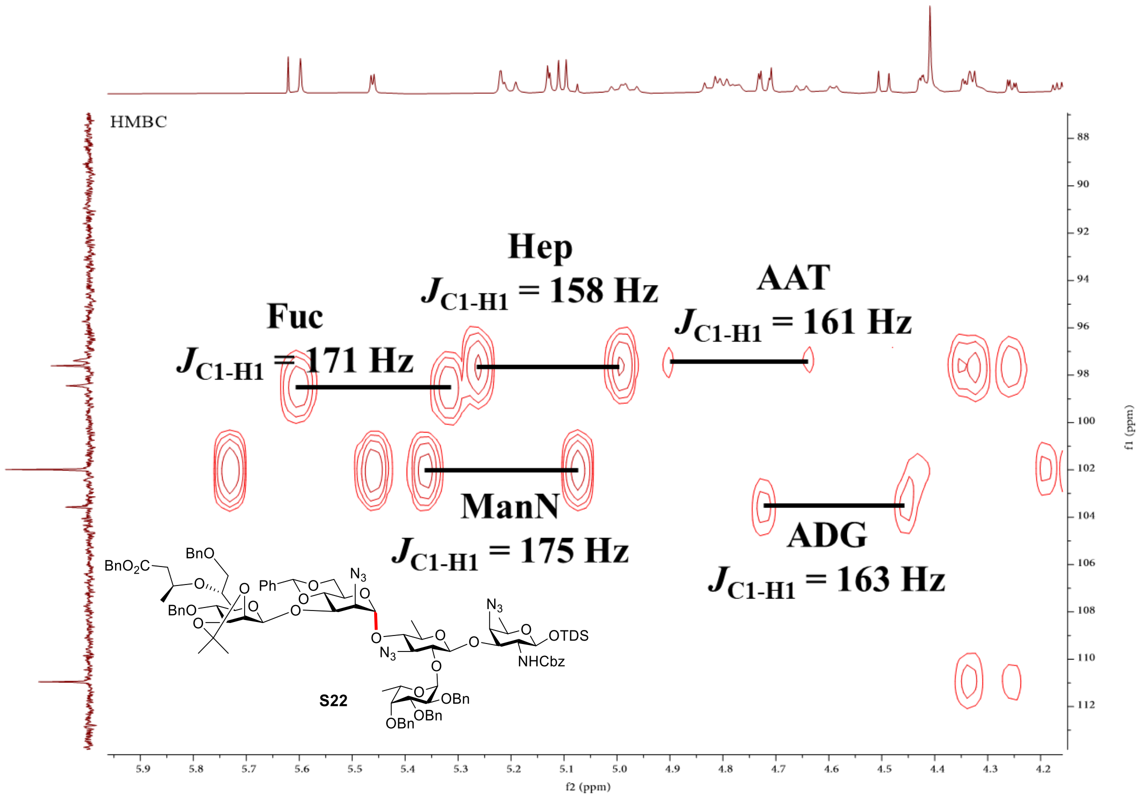
HMBC
Hep
J_{C1-H1} = 158 Hz
AAT
J_{C1-H1} = 161 Hz
Fuc
J_{C1-H1} = 171 Hz
ManN
J_{C1-H1} = 175 Hz
ADG
J_{C1-H1} = 163 Hz
S22
f2 (ppm)
f1 (ppm)

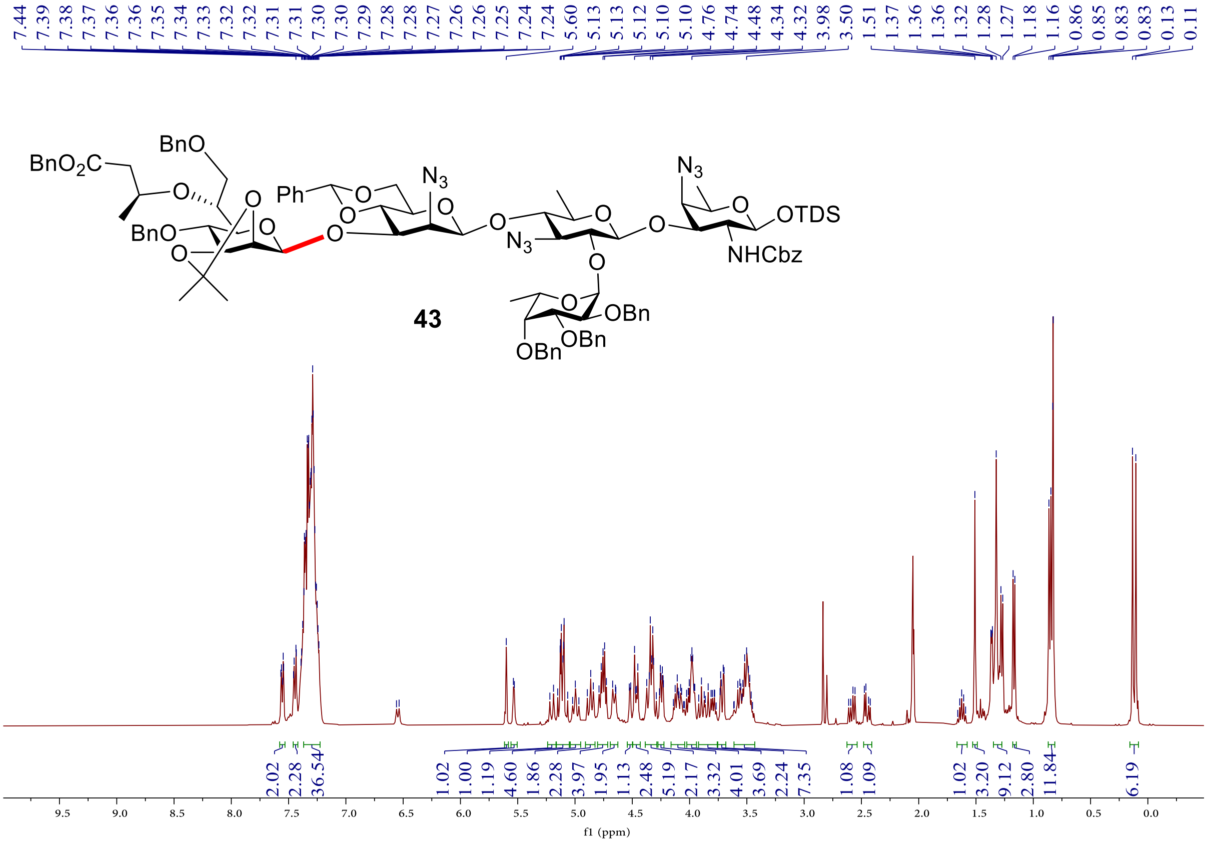
7.44 7.39 7.38 7.37 7.36 7.36 7.35 7.34 7.33 7.32 7.32 7.31 7.31 7.30 7.30 7.29 7.28 7.28 7.27 7.26 7.26 7.25 7.24 7.24 5.60 5.13 5.13 5.12 5.10 5.10 4.76 4.74 4.48 4.34 4.32 3.98 3.50 1.51 1.37 1.36 1.36 1.32 1.28 1.27 1.18 1.16 0.86 0.85 0.83 0.83 0.13 0.11
BnO$_2$C
BnO
BnO
Ph
N$_3$
N$_3$
N$_3$
OTDS
NHCbz
OBn
OBn
OBn
43
2.02 2.28 36.54 1.02 1.00 1.19 4.60 1.86 2.28 3.97 1.95 1.13 2.48 5.19 2.17 3.32 4.01 3.69 2.24 7.35 1.08 1.09 1.02 3.20 9.12 2.80 11.84 6.19
f1 (ppm)

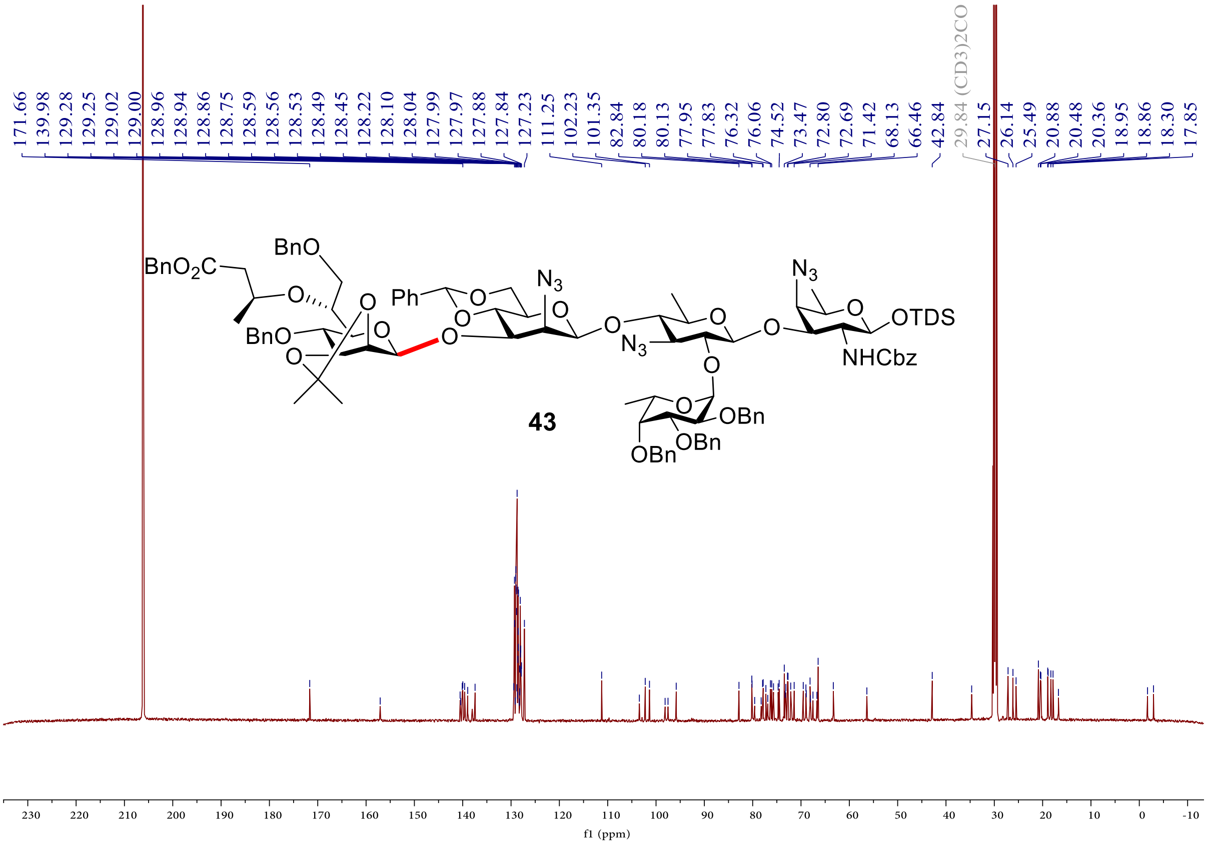
171.66 139.98 129.28 129.25 129.02 129.00 128.96 128.94 128.86 128.75 128.59 128.56 128.53 128.49 128.45 128.22 128.10 128.04 127.99 127.97 127.88 127.84 127.23 111.25 102.23 101.35 82.84 80.18 80.13 77.95 77.83 76.32 76.06 74.52 73.47 72.80 72.69 71.42 68.13 66.46 42.84 29.84 (CD3)2CO 27.15 26.14 25.49 20.88 20.48 20.36 18.95 18.86 18.30 17.85
BnO$_2$C
BnO
BnO
Ph
N$_3$
N$_3$
N$_3$
OTDS
NHCbz
OBn
OBn
OBn
43
f1 (ppm)

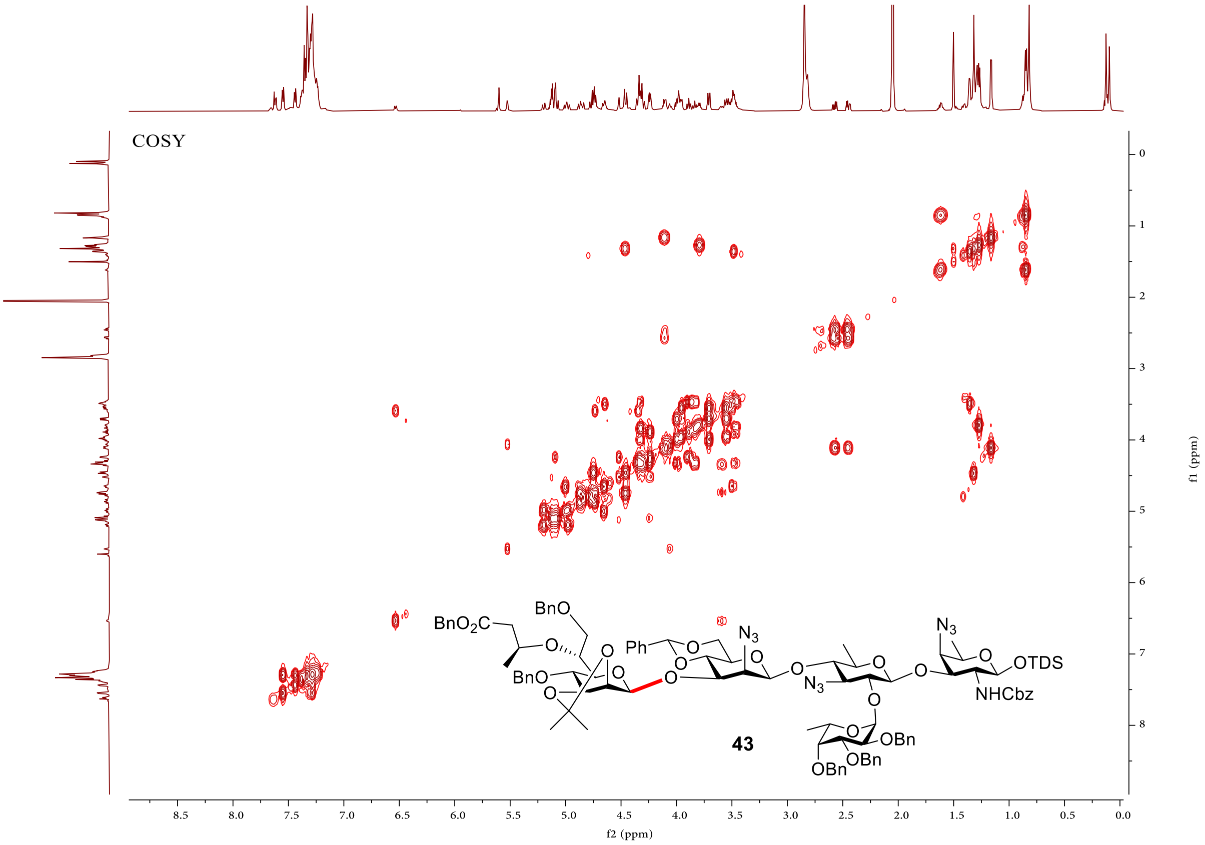
COSY
BnO
BnO2C
O
Ph
N3
OTDS
BnO
NHCbz
OBn
OBn
OBn
43
f2 (ppm)
f1 (ppm)

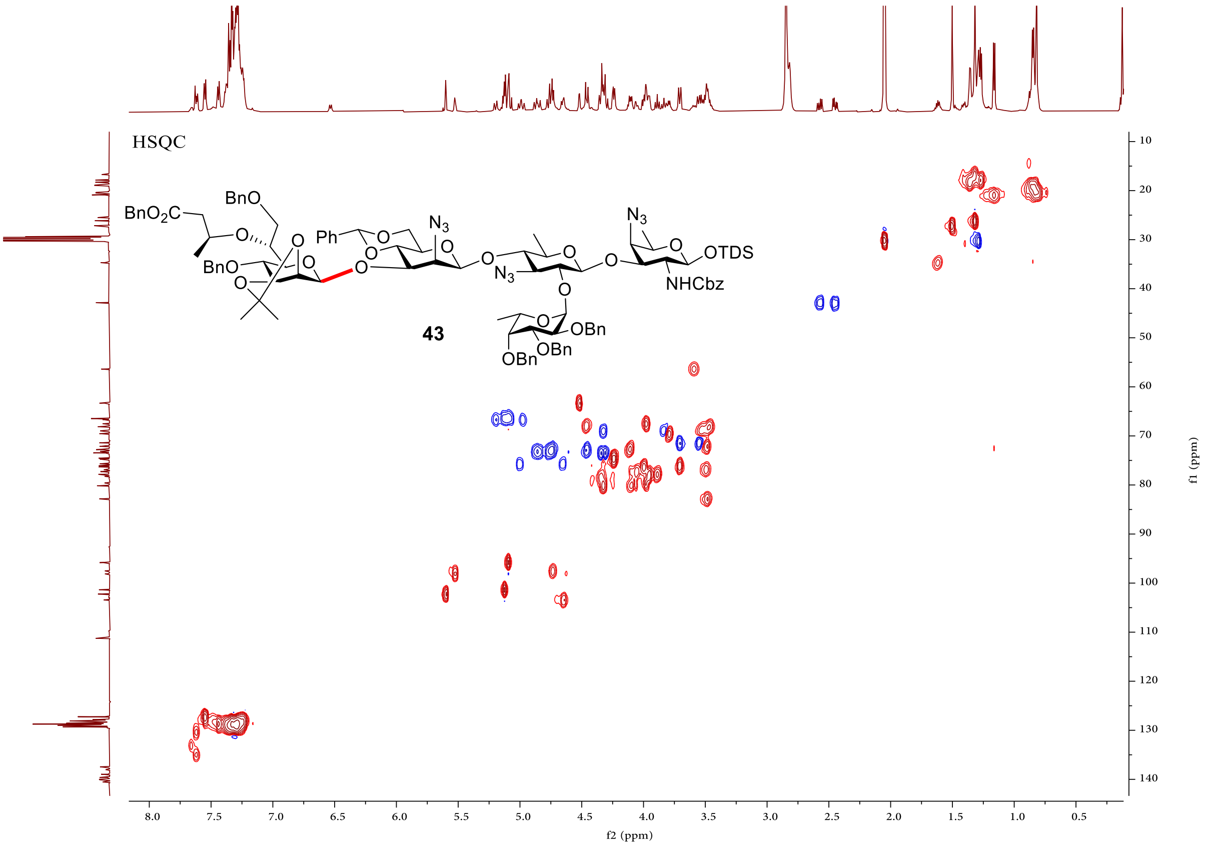
HSQC
BnO
BnO2C
O
Ph
N3
OTDS
BnO
NHCbz
OBn
OBn
OBn
43
f2 (ppm)
f1 (ppm)

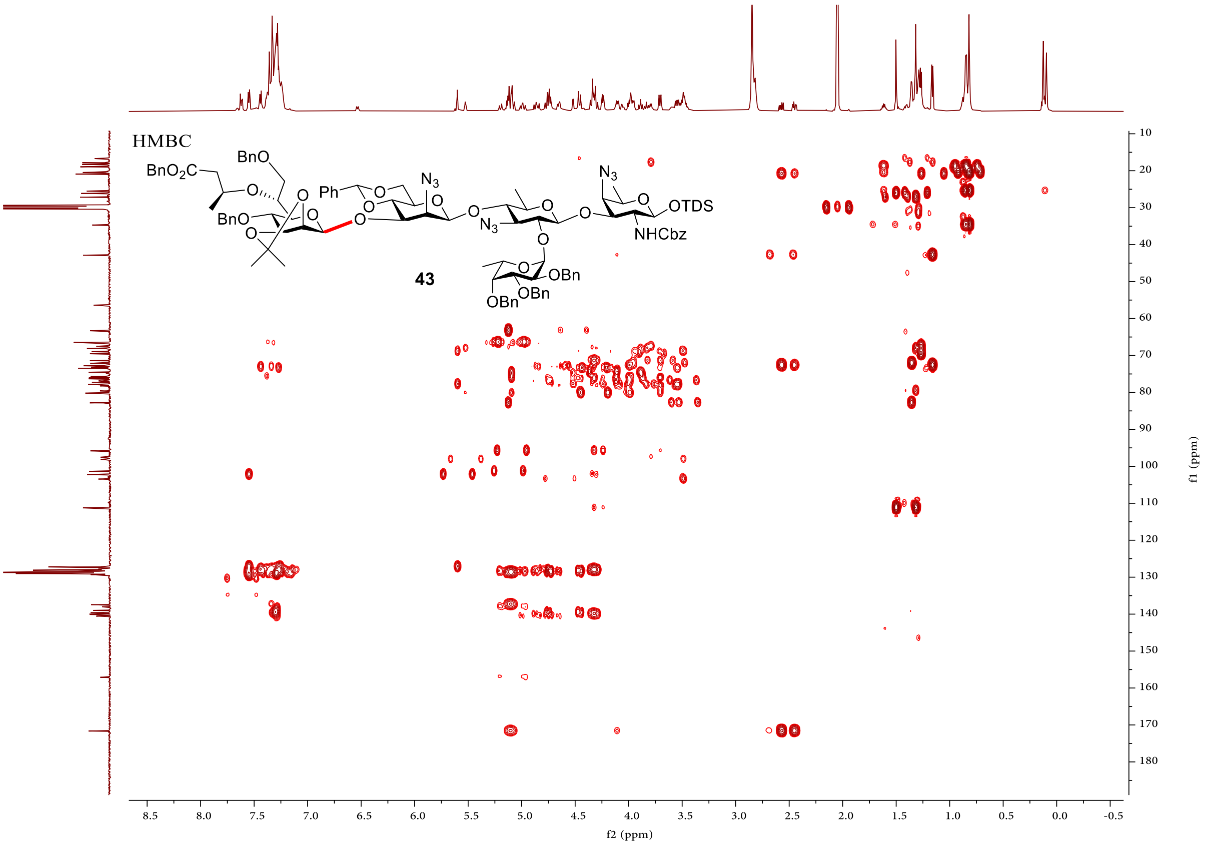
HMBC
BnO
BnO2C
O
Ph
N3
O
OTDS
NHCbz
BnO
43
OBn
OBn
OBn
f1 (ppm)
f2 (ppm)

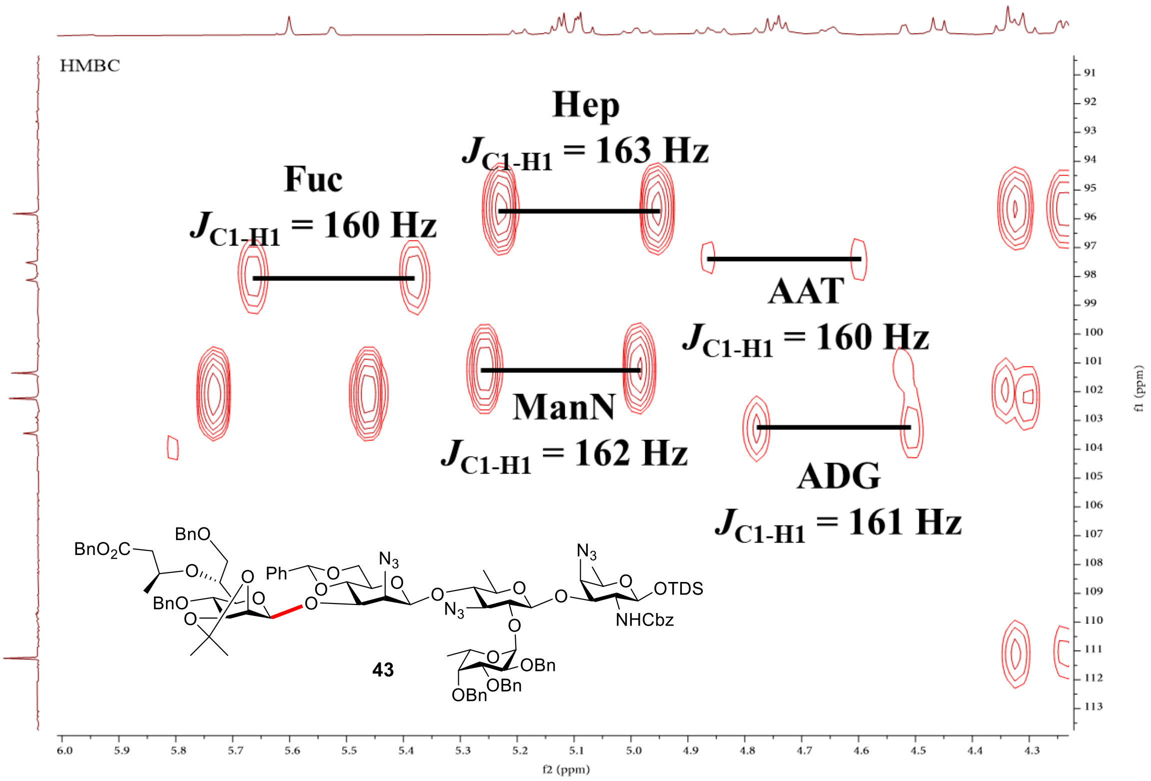
HMBC
Hep
JC1-H1 = 163 Hz
Fuc
JC1-H1 = 160 Hz
AAT
JC1-H1 = 160 Hz
ManN
JC1-H1 = 162 Hz
ADG
JC1-H1 = 161 Hz
BnO
BnO2C
Ph
N3
OTDS
NHCbz
43
OBn
OBn
OBn
f1 (ppm)
f2 (ppm)

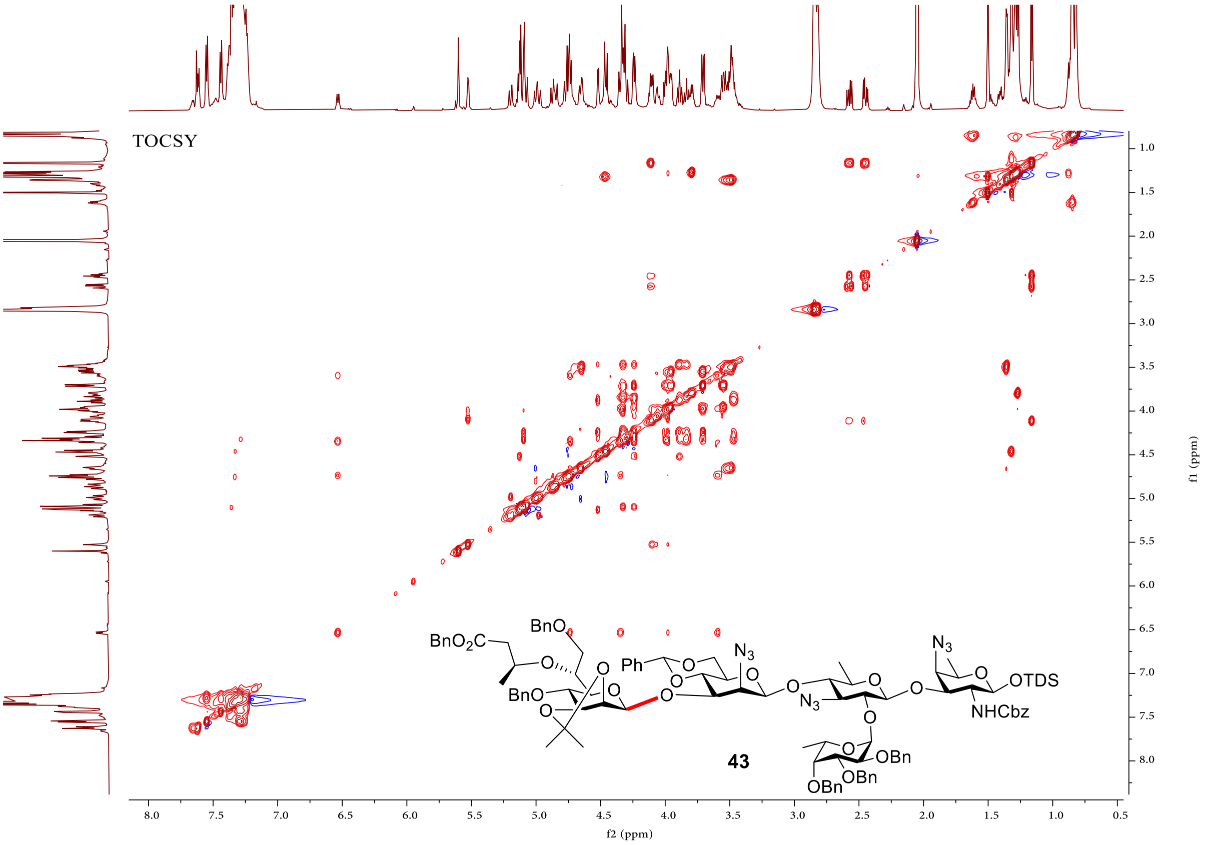
TOCSY
BnO
BnO2C
O
Ph
N3
OTDS
NHCbz
OBn
43
f2 (ppm)
f1 (ppm)

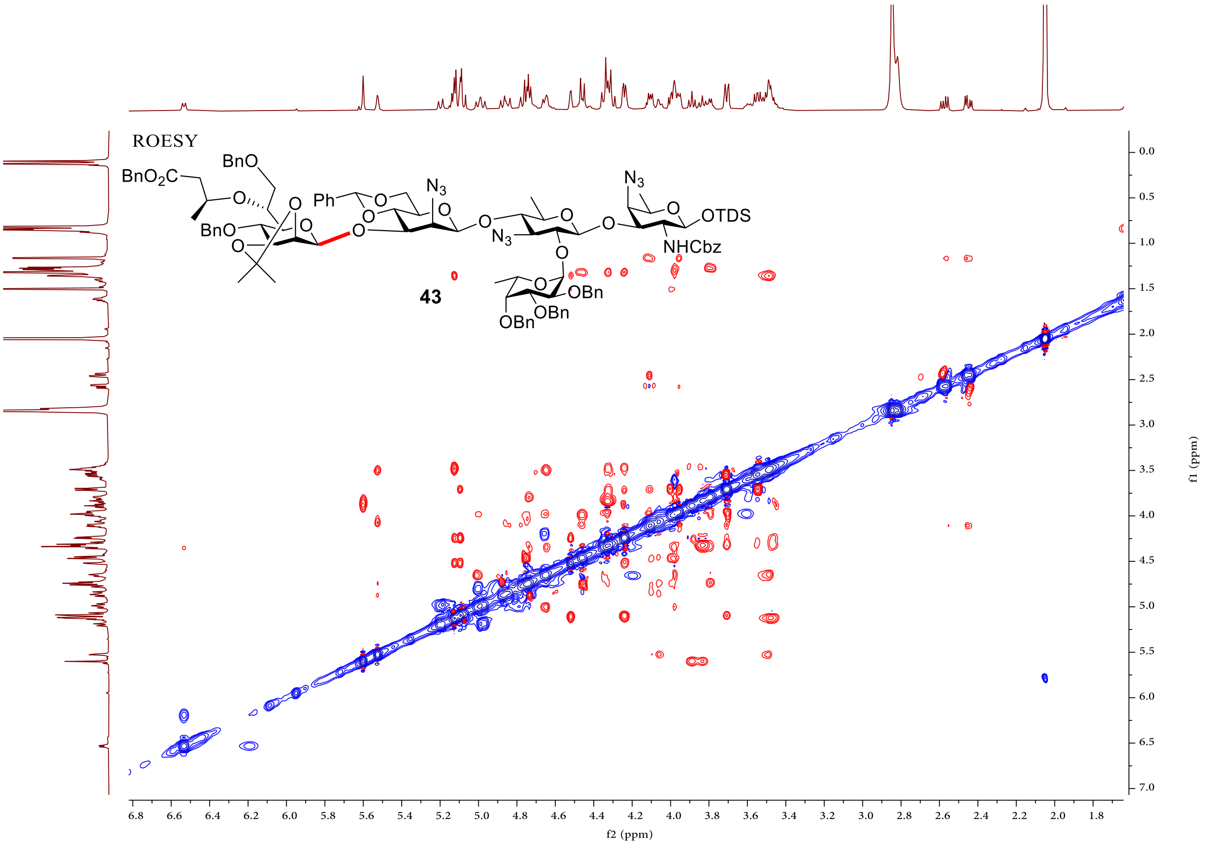
ROESY
BnO
BnO2C
O
Ph
N3
OTDS
NHCbz
OBn
43
f2 (ppm)
f1 (ppm)

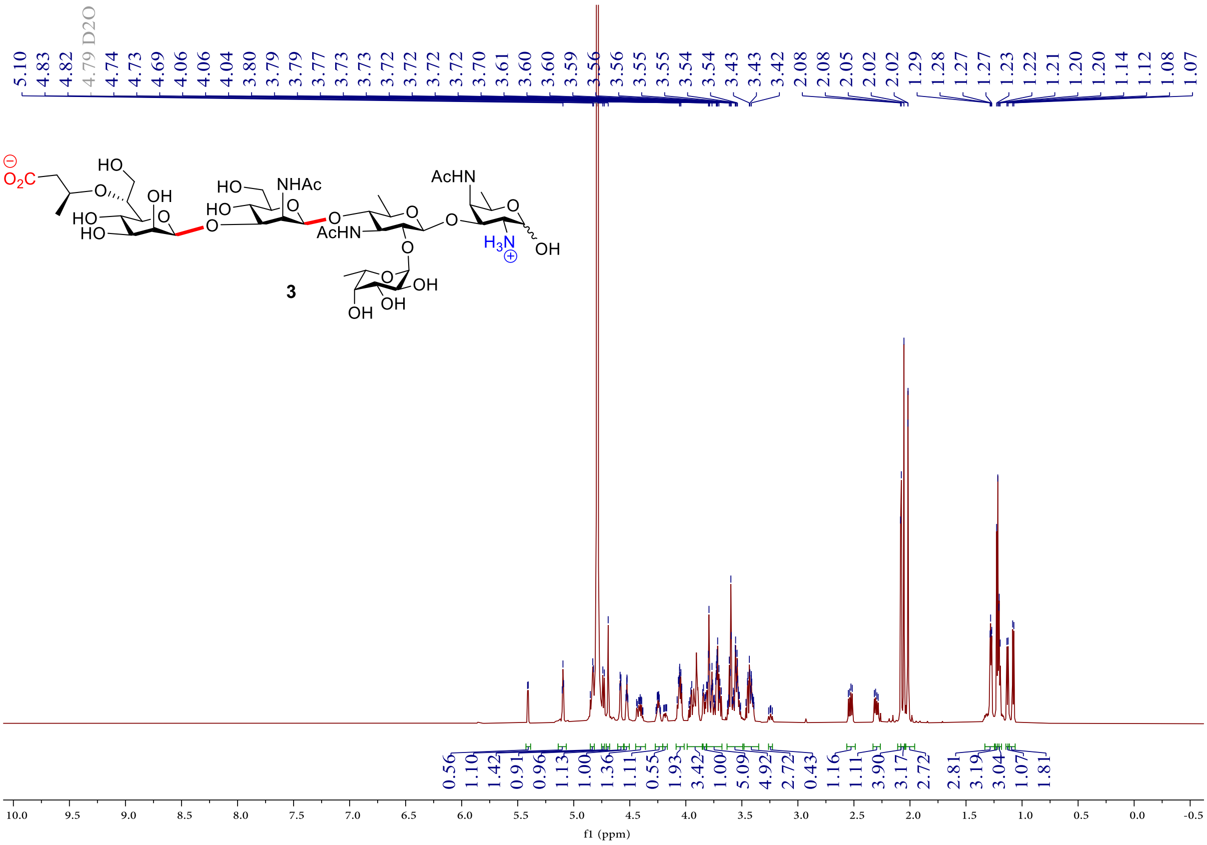
3
f1 (ppm)

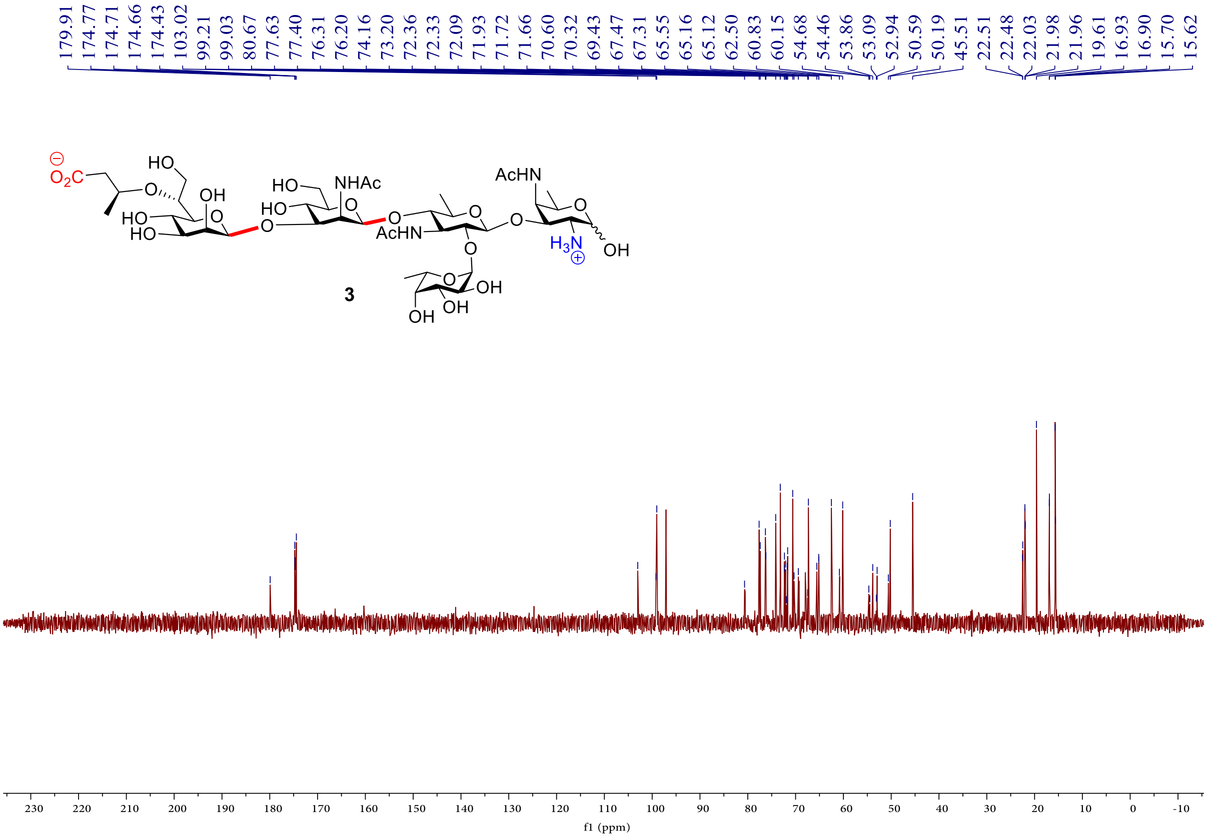
3
f1 (ppm)

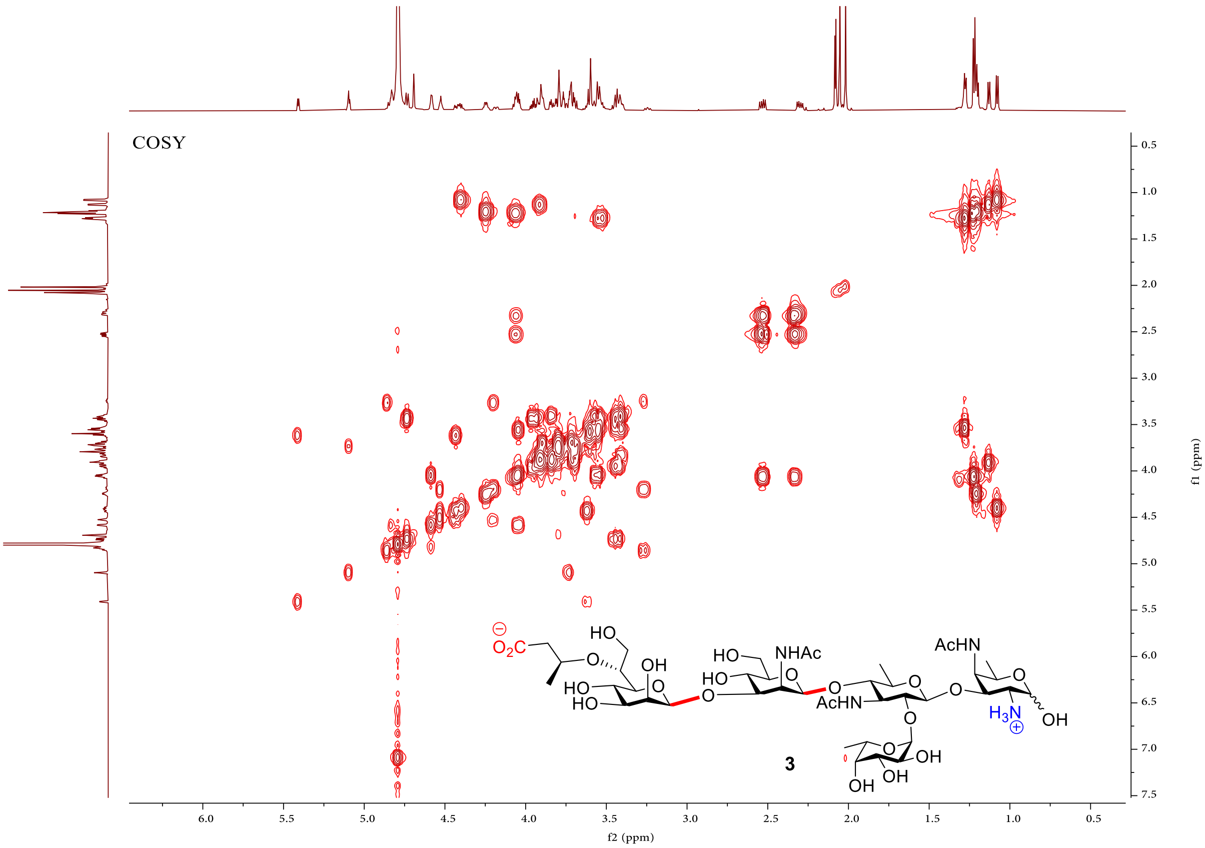
COSY
f2 (ppm)
f1 (ppm)
3

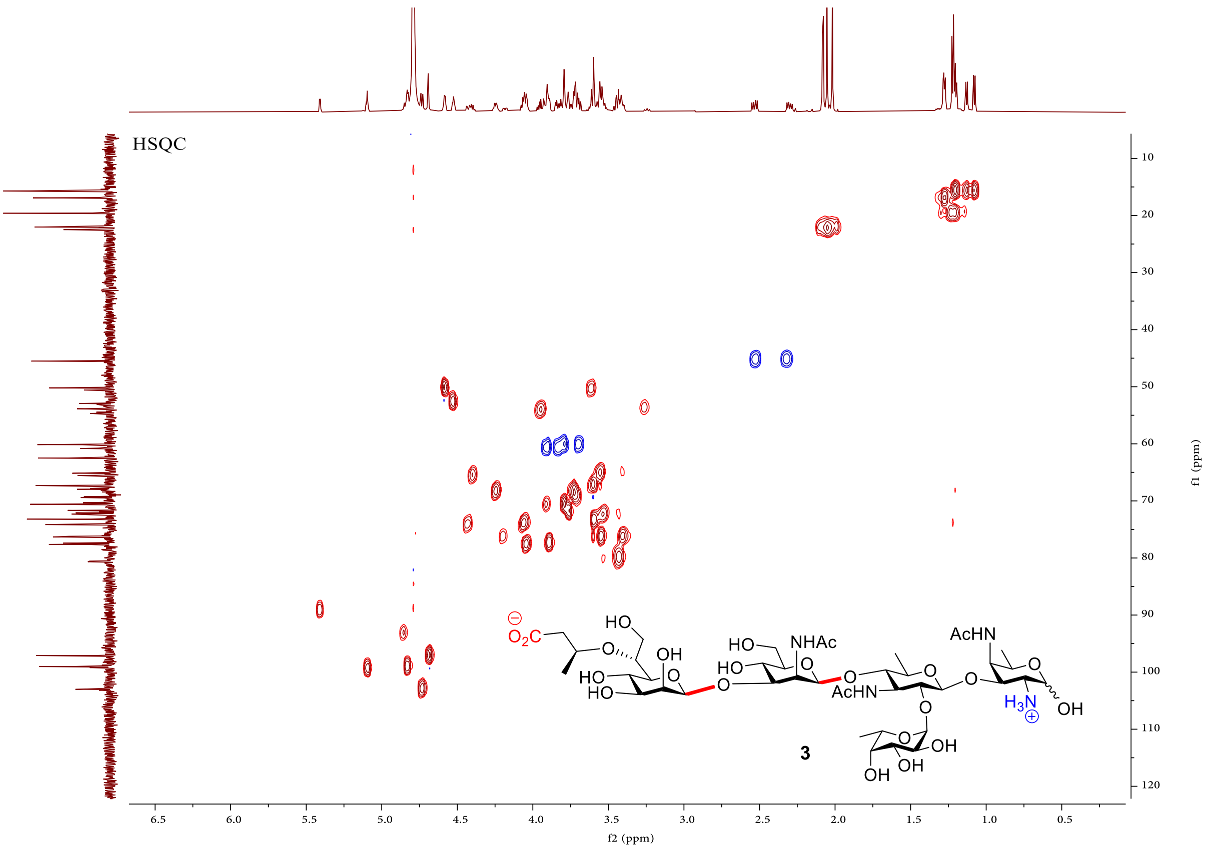
HSQC
f2 (ppm)
f1 (ppm)
3

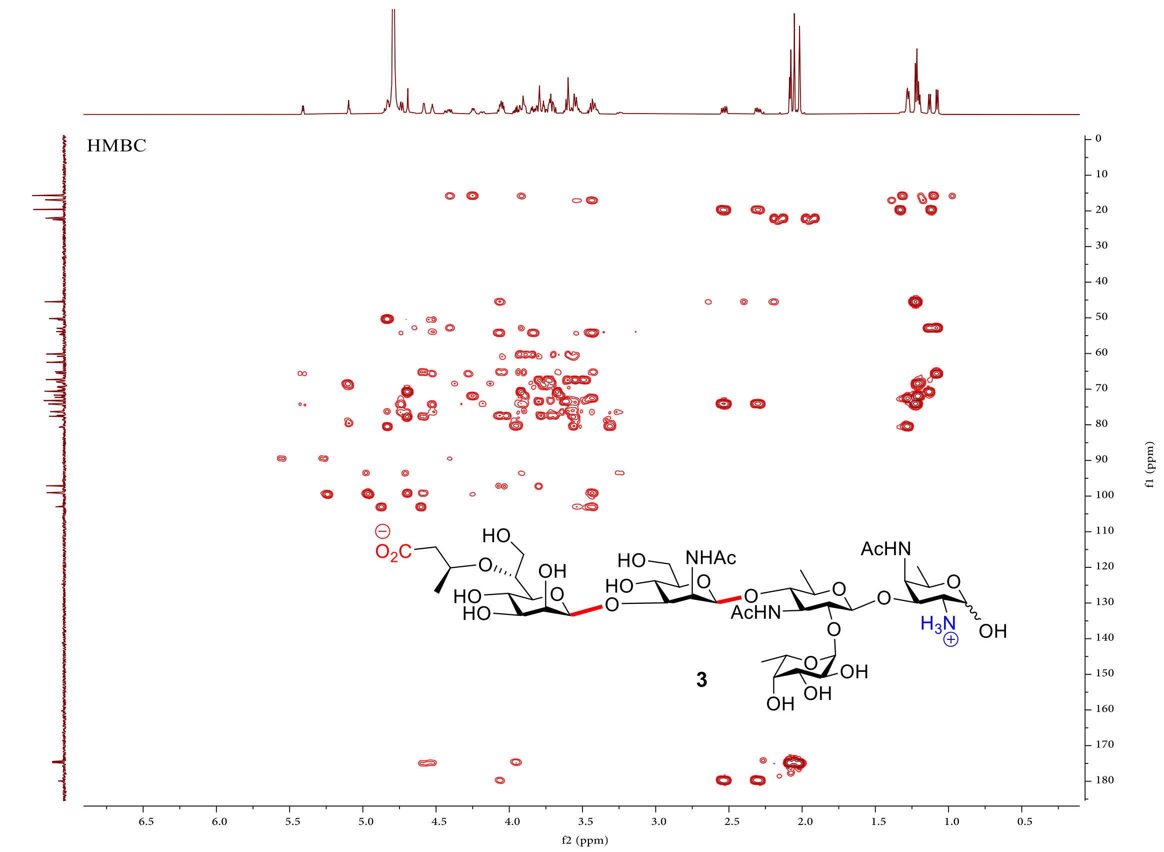
HMBC
3
f2 (ppm)
f1 (ppm)

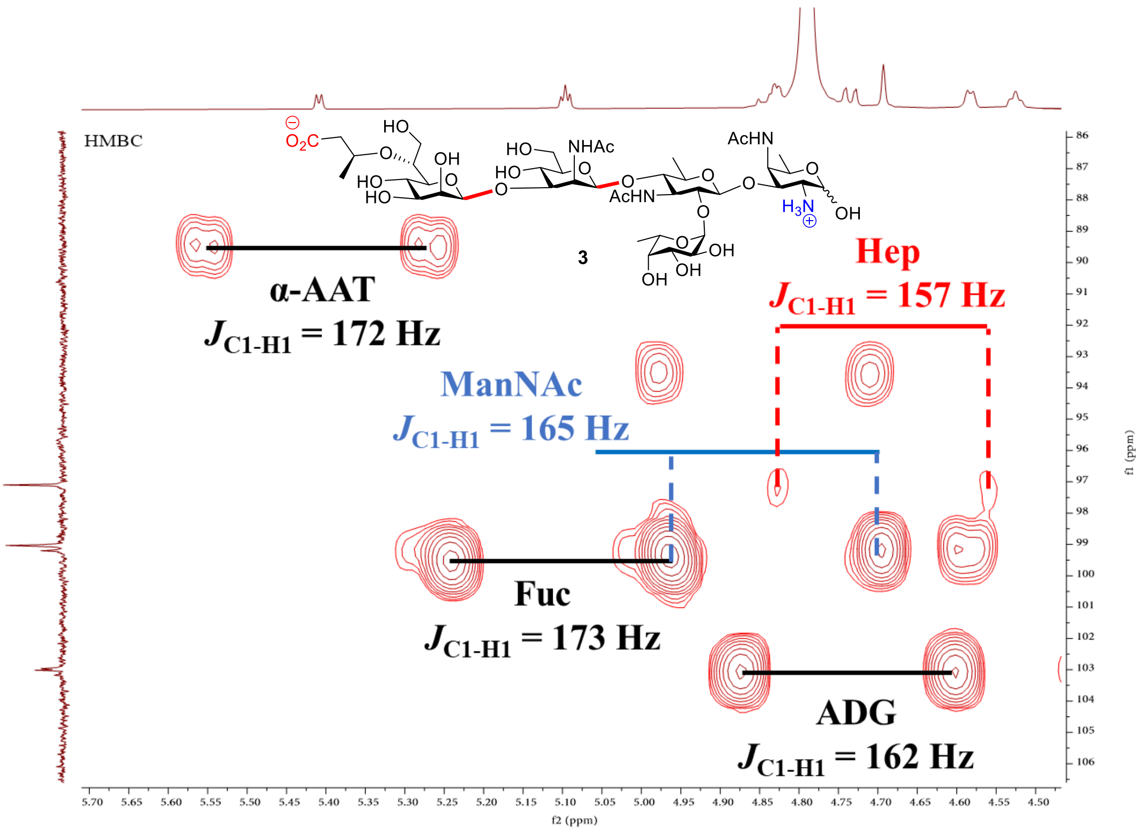
HMBC
3
α-AAT
JC1-H1 = 172 Hz
Hep
JC1-H1 = 157 Hz
ManNAc
JC1-H1 = 165 Hz
Fuc
JC1-H1 = 173 Hz
ADG
JC1-H1 = 162 Hz
f2 (ppm)
f1 (ppm)

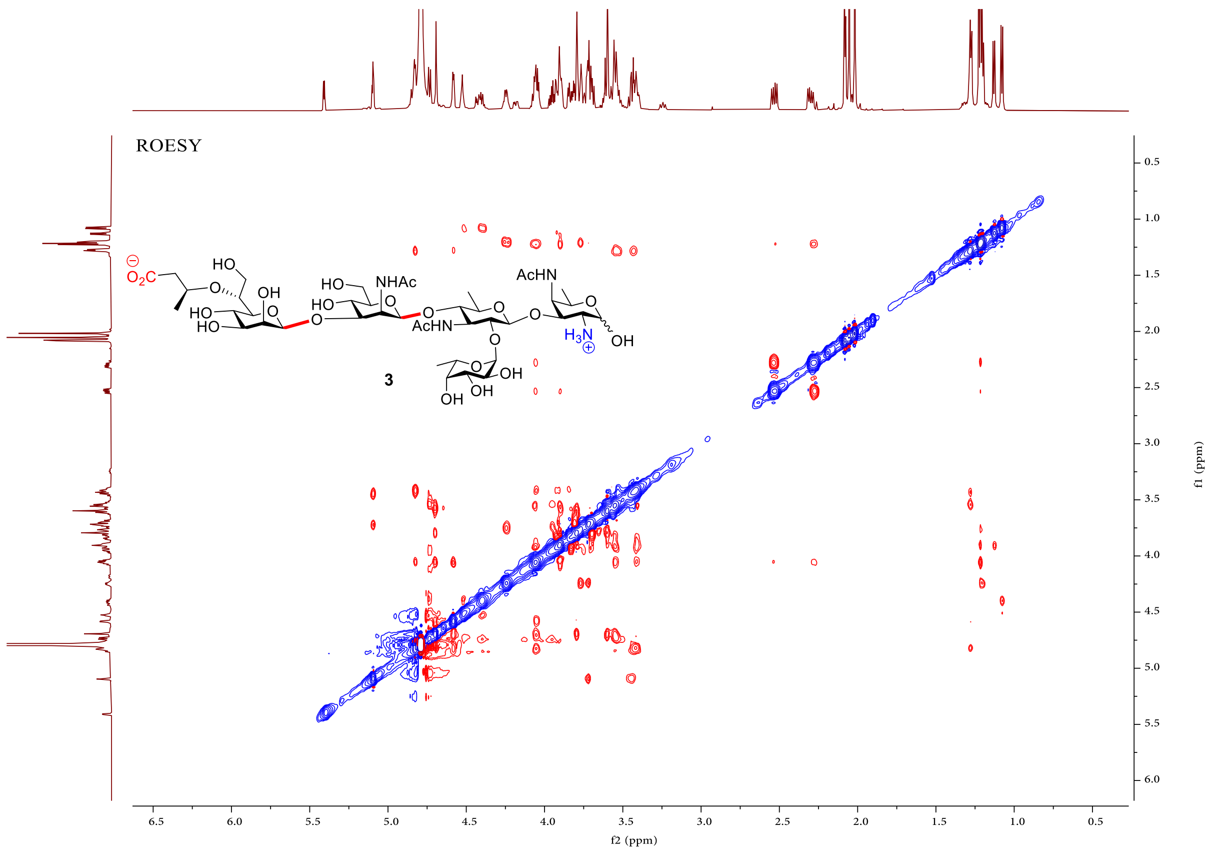
ROESY
3
f1 (ppm)
f2 (ppm)

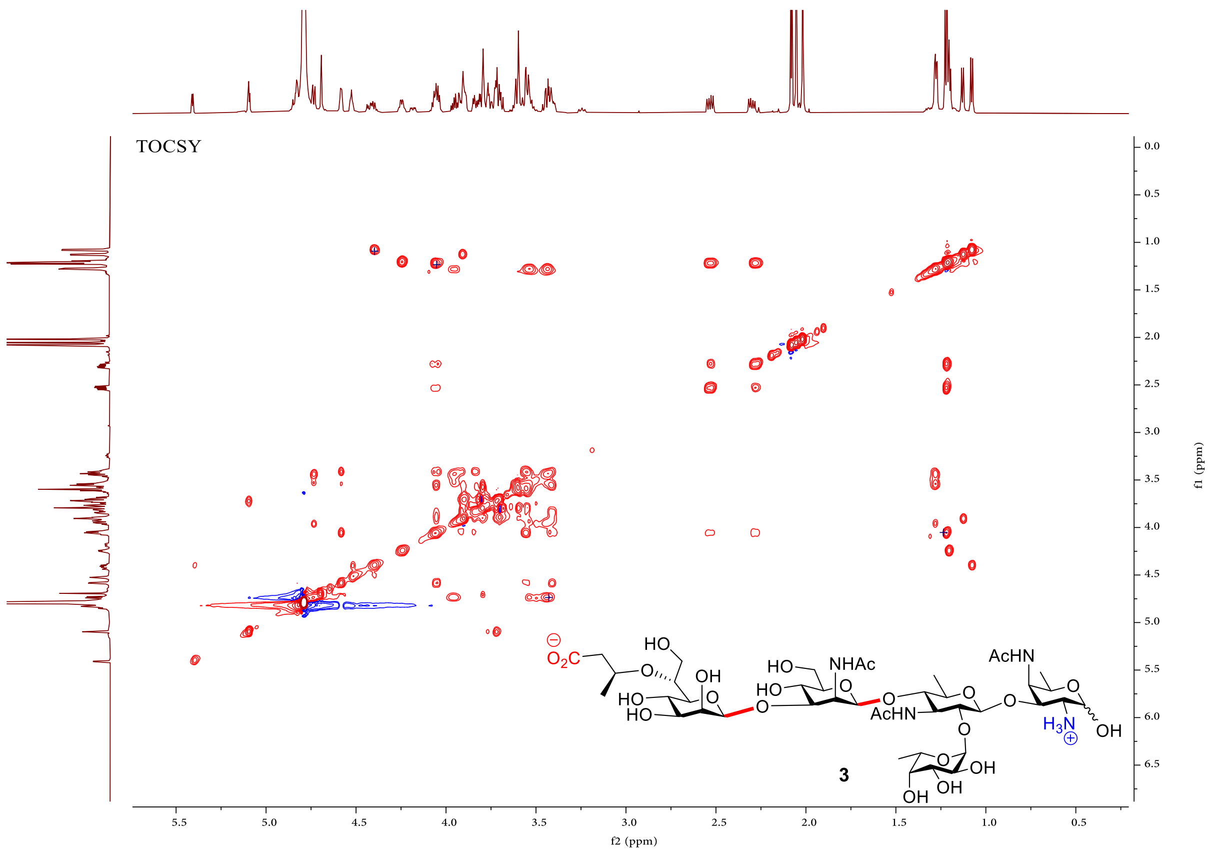
TOCSY
3
f1 (ppm)
f2 (ppm)

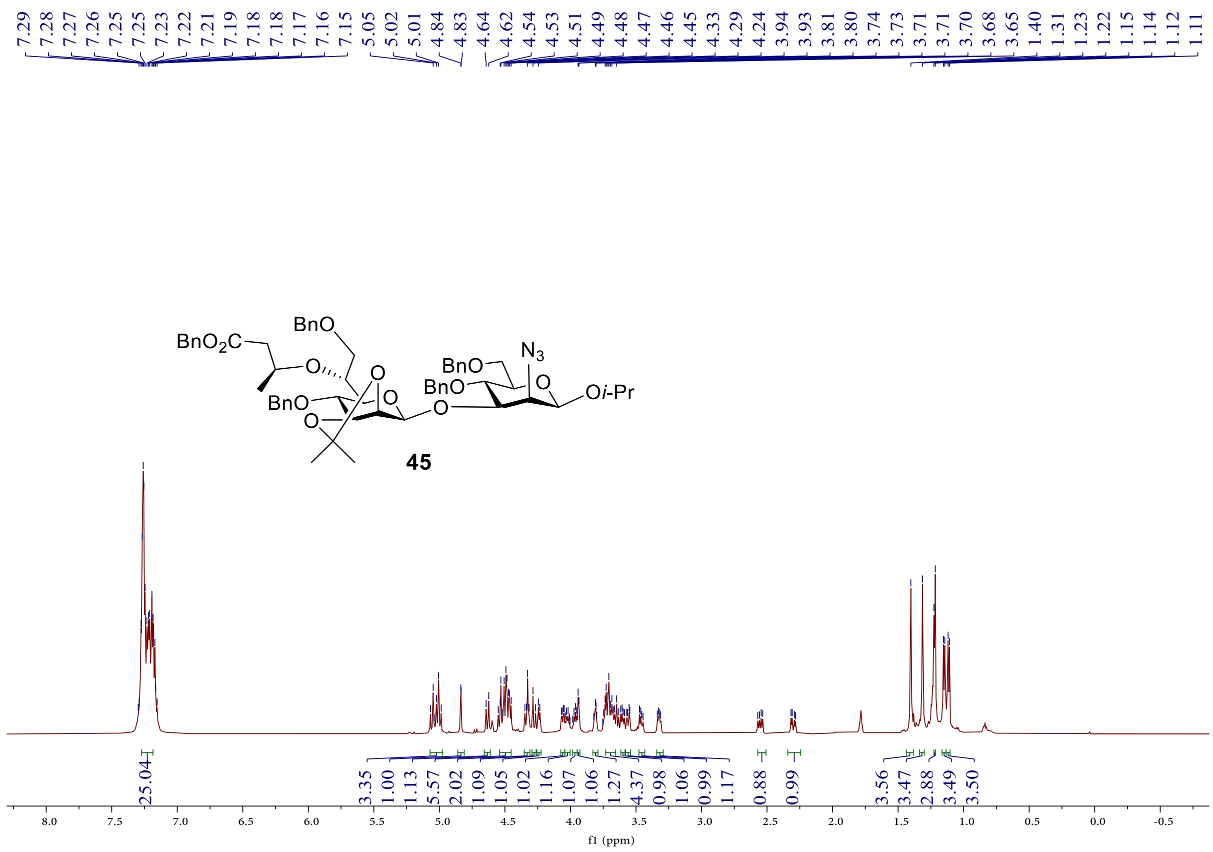

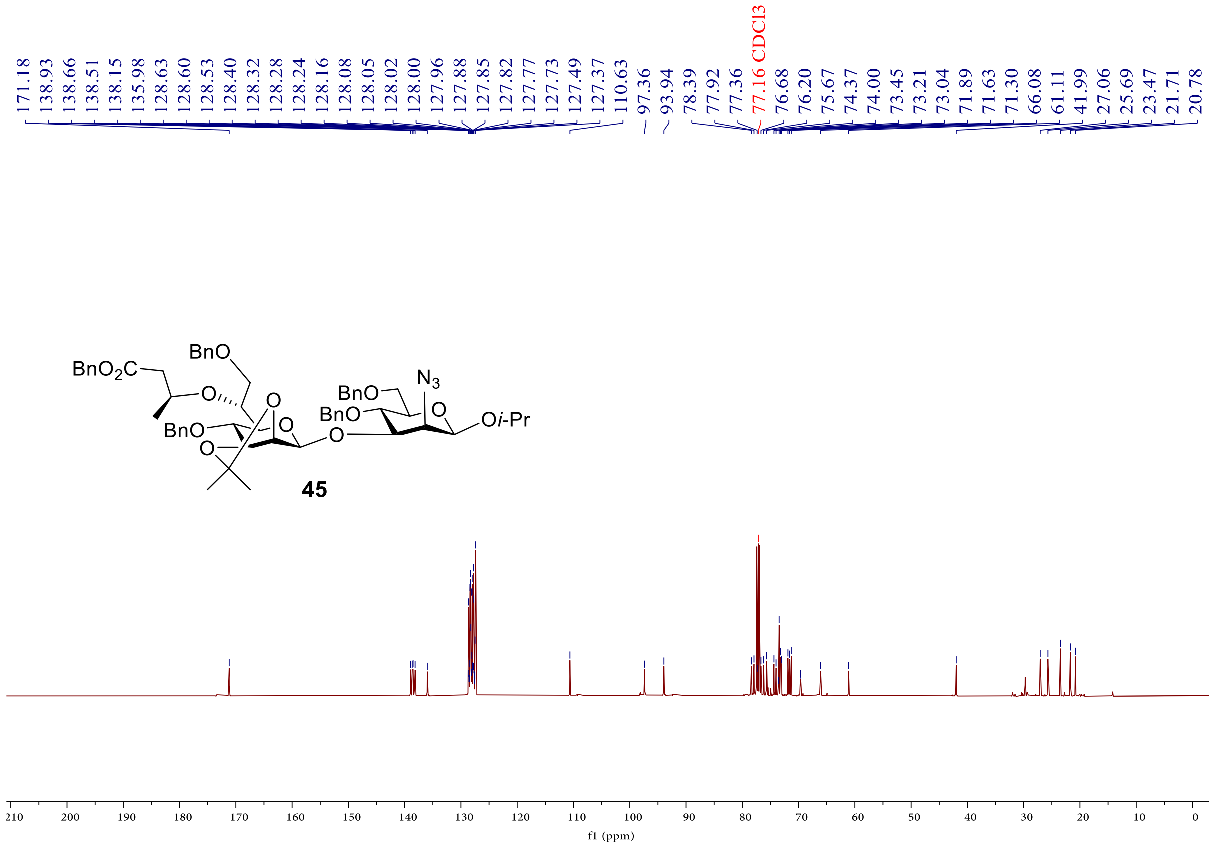

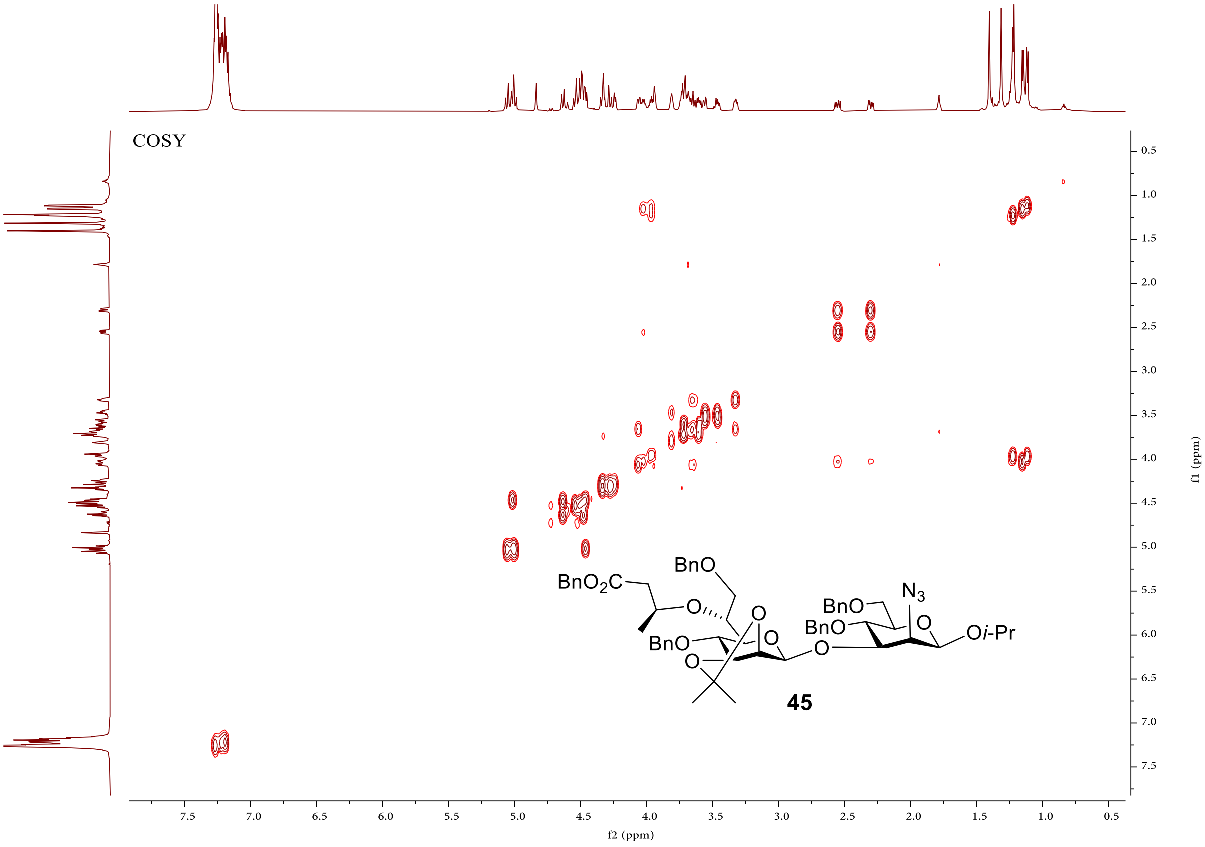
COSY
BnO
BnO2C
O
BnO
N3
BnO
O
O
BnO
O
Oi-Pr
BnO
O
O
45
f2 (ppm)
f1 (ppm)

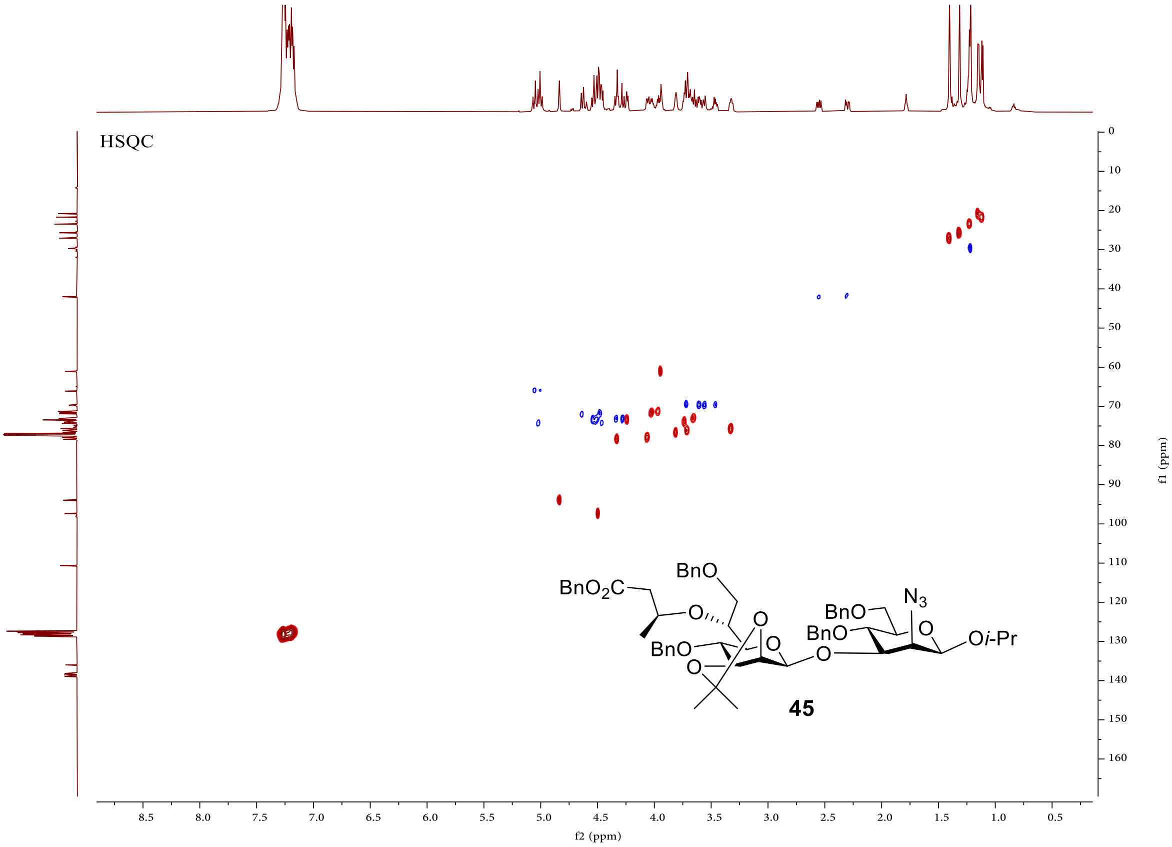
HSQC
BnO
BnO2C
O
BnO
N3
BnO
O
O
BnO
O
Oi-Pr
BnO
O
O
45
f2 (ppm)
f1 (ppm)

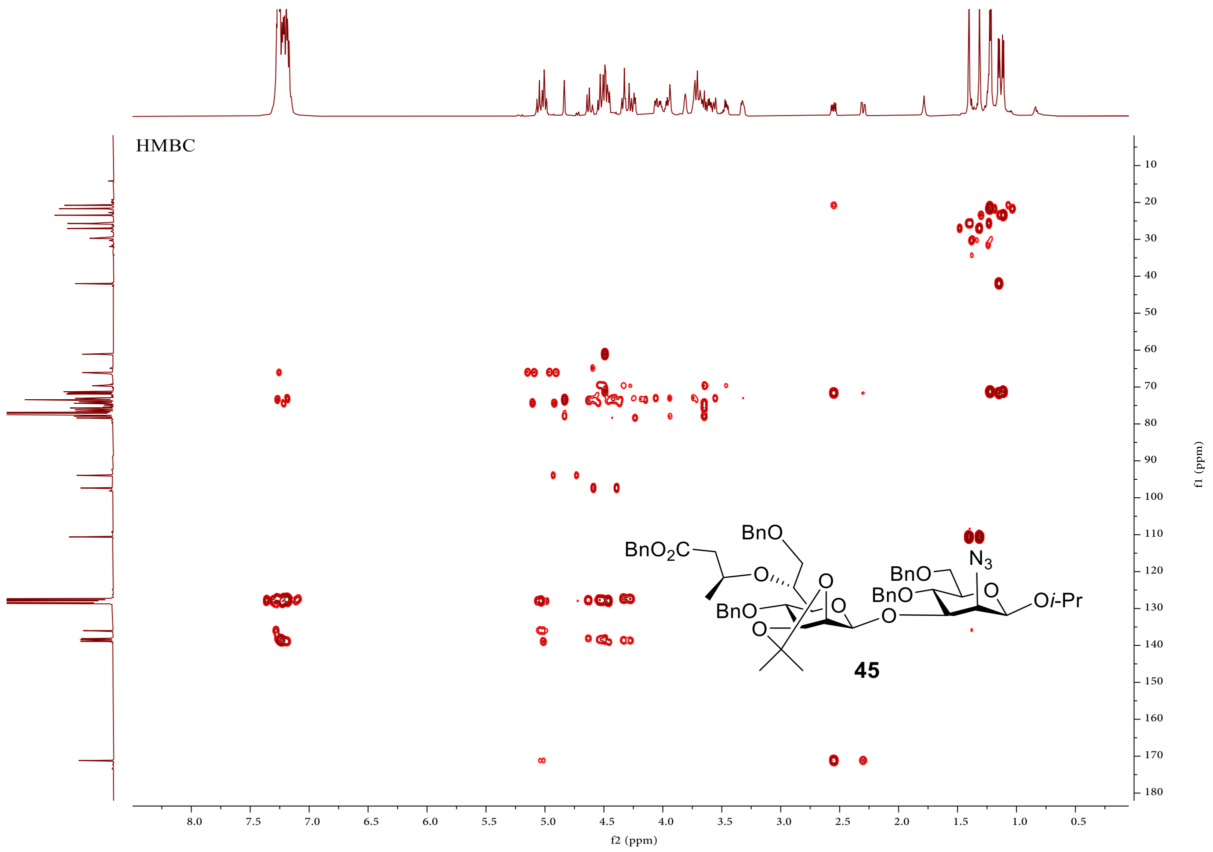
HMBC
BnO
BnO2C
N3
BnO
BnO
O
Oi-Pr
BnO
45
f2 (ppm)
f1 (ppm)

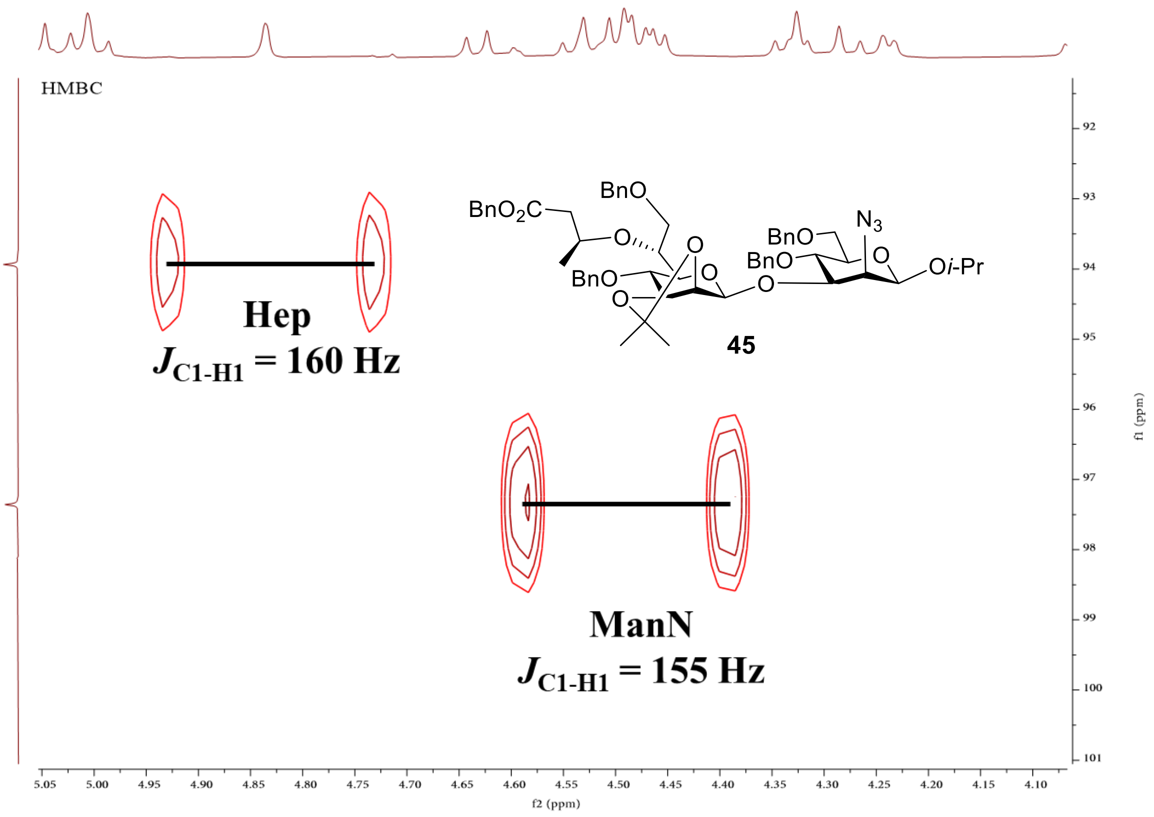
HMBC
Hep
JC1-H1 = 160 Hz
ManN
JC1-H1 = 155 Hz
45
f2 (ppm)
f1 (ppm)

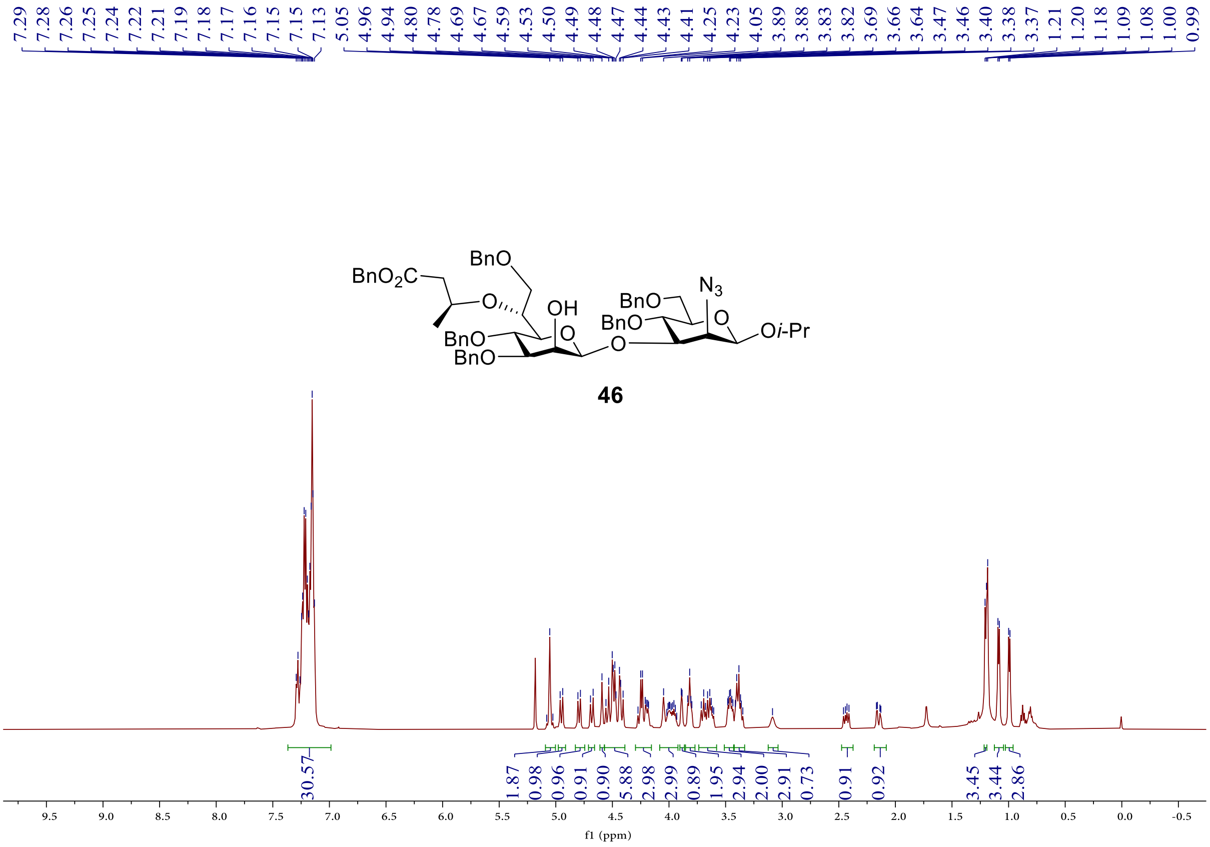
7.29 7.28 7.26 7.25 7.24 7.22 7.21 7.19 7.18 7.17 7.16 7.15 7.15 7.13 5.05 4.96 4.94 4.80 4.78 4.69 4.67 4.59 4.53 4.50 4.49 4.48 4.47 4.44 4.43 4.41 4.25 4.23 4.05 3.89 3.88 3.83 3.82 3.69 3.66 3.64 3.47 3.46 3.40 3.38 3.37 1.21 1.20 1.18 1.09 1.08 1.00 0.99
BnO
BnO_2C
O
OH
O
BnO
BnO
BnO
BnO
N_3
O
O
Oi-Pr
46
30.57
1.87 0.98 0.96 0.91 0.90 5.88 2.98 2.99 0.89 1.95 2.94 2.00 2.91 0.73 0.91 0.92 3.45 3.44 2.86
9.5 9.0 8.5 8.0 7.5 7.0 6.5 6.0 5.5 5.0 4.5 4.0 3.5 3.0 2.5 2.0 1.5 1.0 0.5 0.0 -0.5
f1 (ppm)

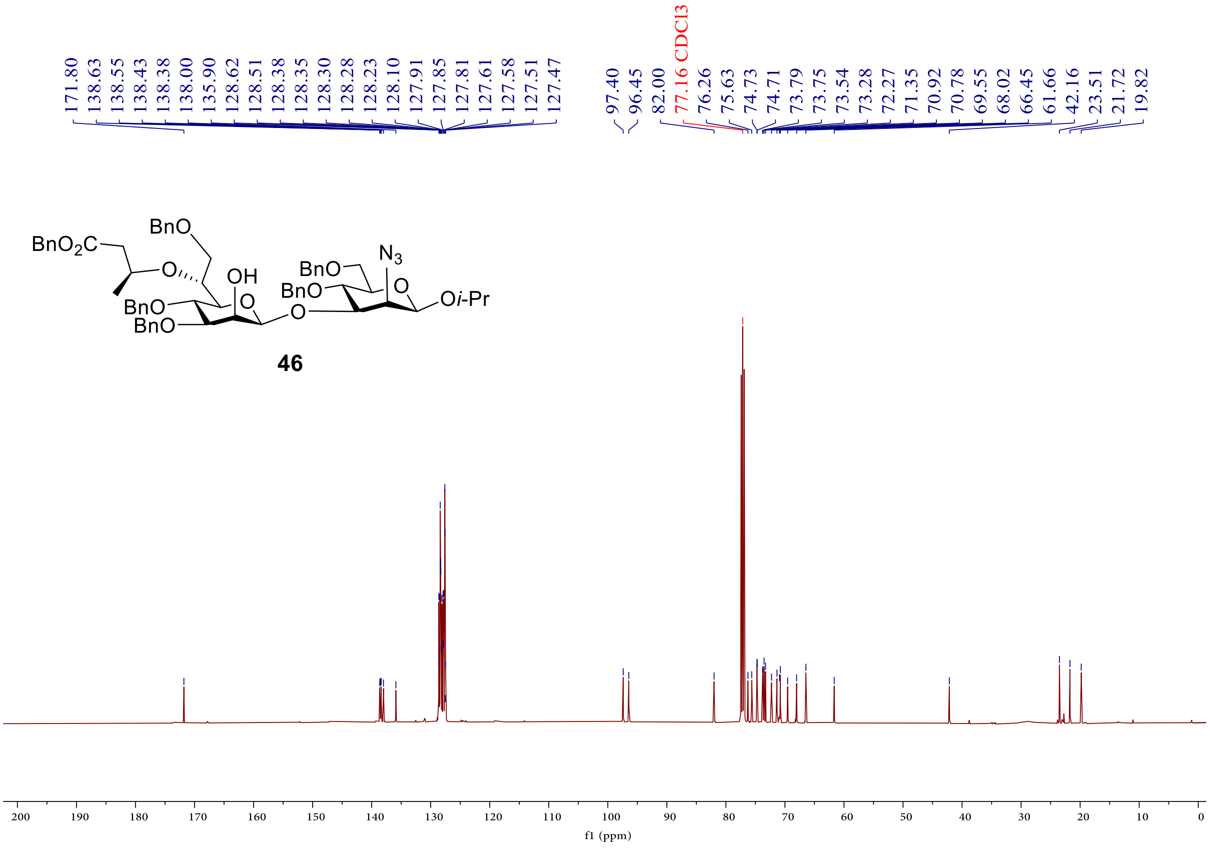
171.80 138.63 138.55 138.43 138.38 138.00 135.90 128.62 128.51 128.38 128.35 128.30 128.28 128.23 128.10 127.91 127.85 127.81 127.61 127.58 127.51 127.47
97.40 96.45 82.00 77.16 CDCl3 76.26 75.63 74.73 74.71 73.79 73.75 73.54 73.28 72.27 71.35 70.92 70.78 69.55 68.02 66.45 61.66 42.16 23.51 21.72 19.82
BnO
BnO_2C
O
OH
O
BnO
BnO
BnO
BnO
N_3
O
O
Oi-Pr
46
200 190 180 170 160 150 140 130 120 110 100 90 80 70 60 50 40 30 20 10 0
f1 (ppm)

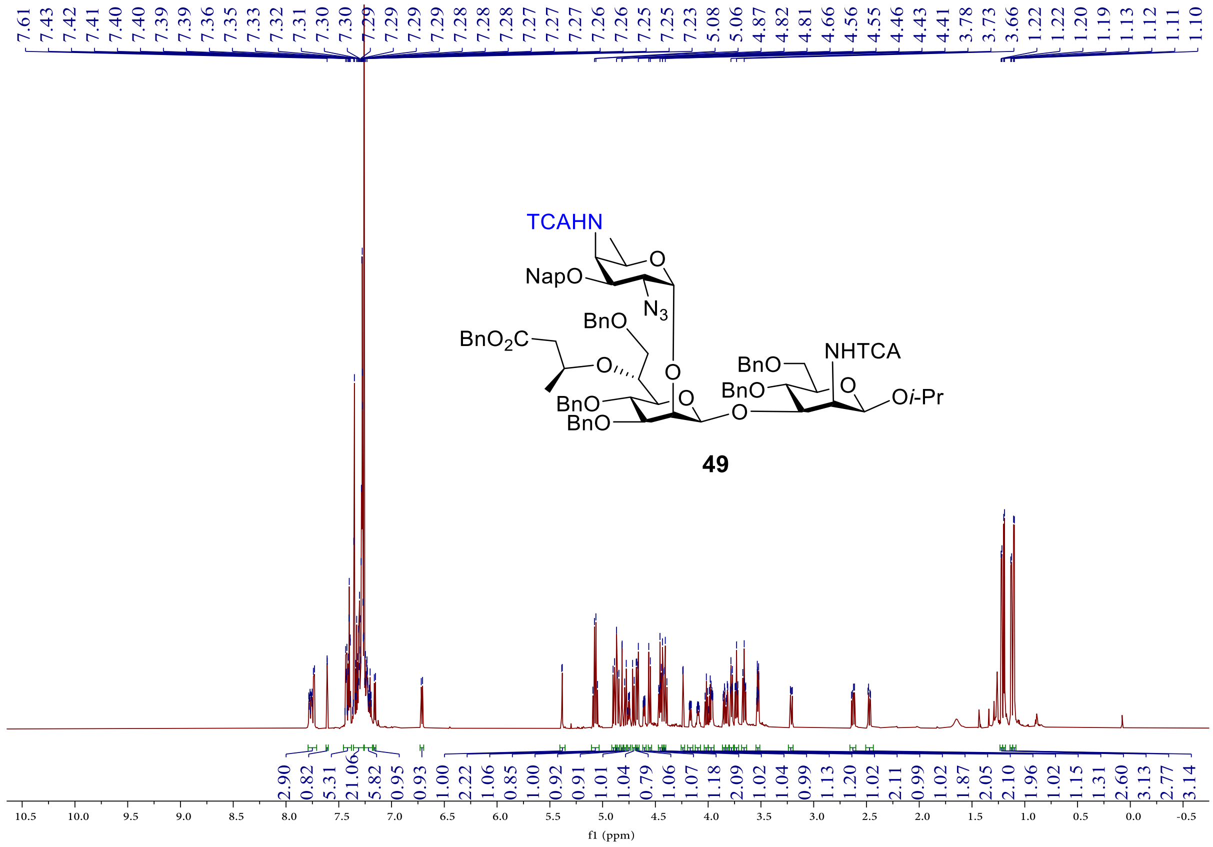

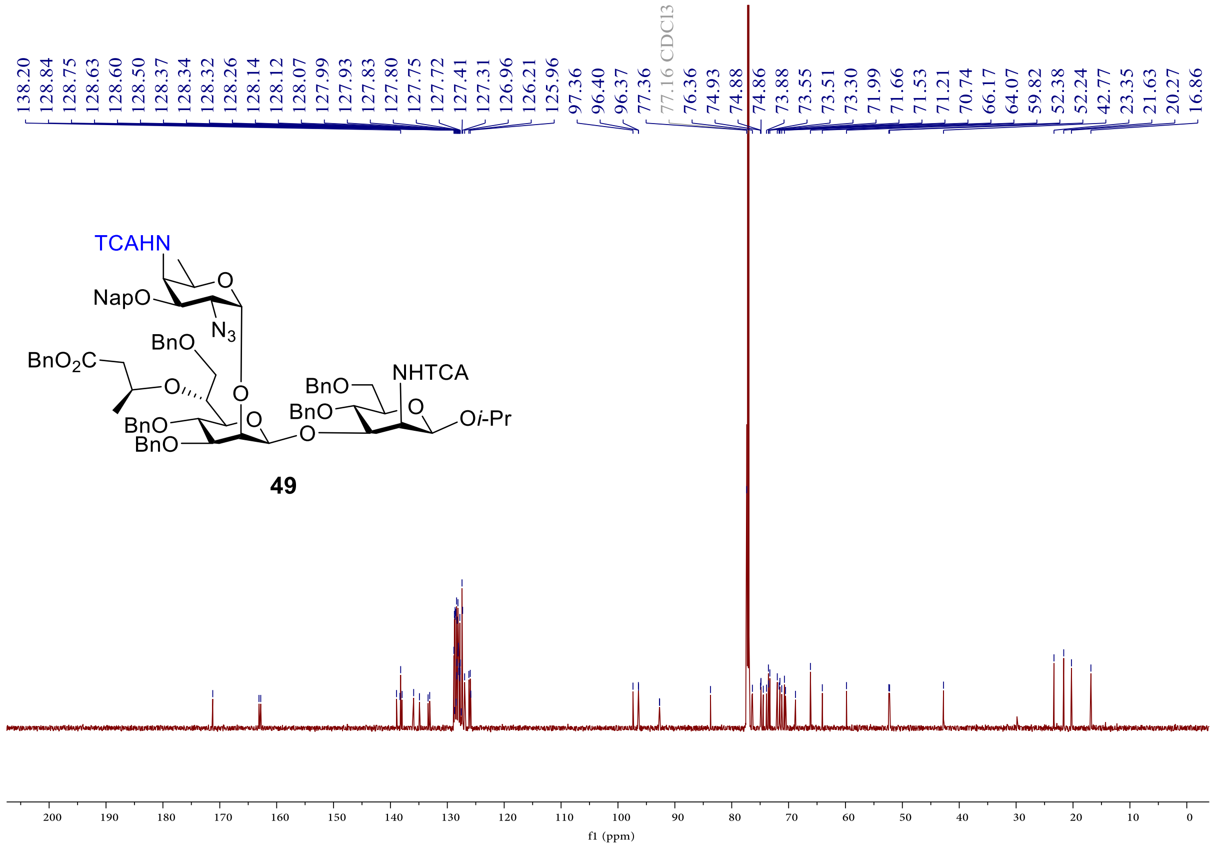

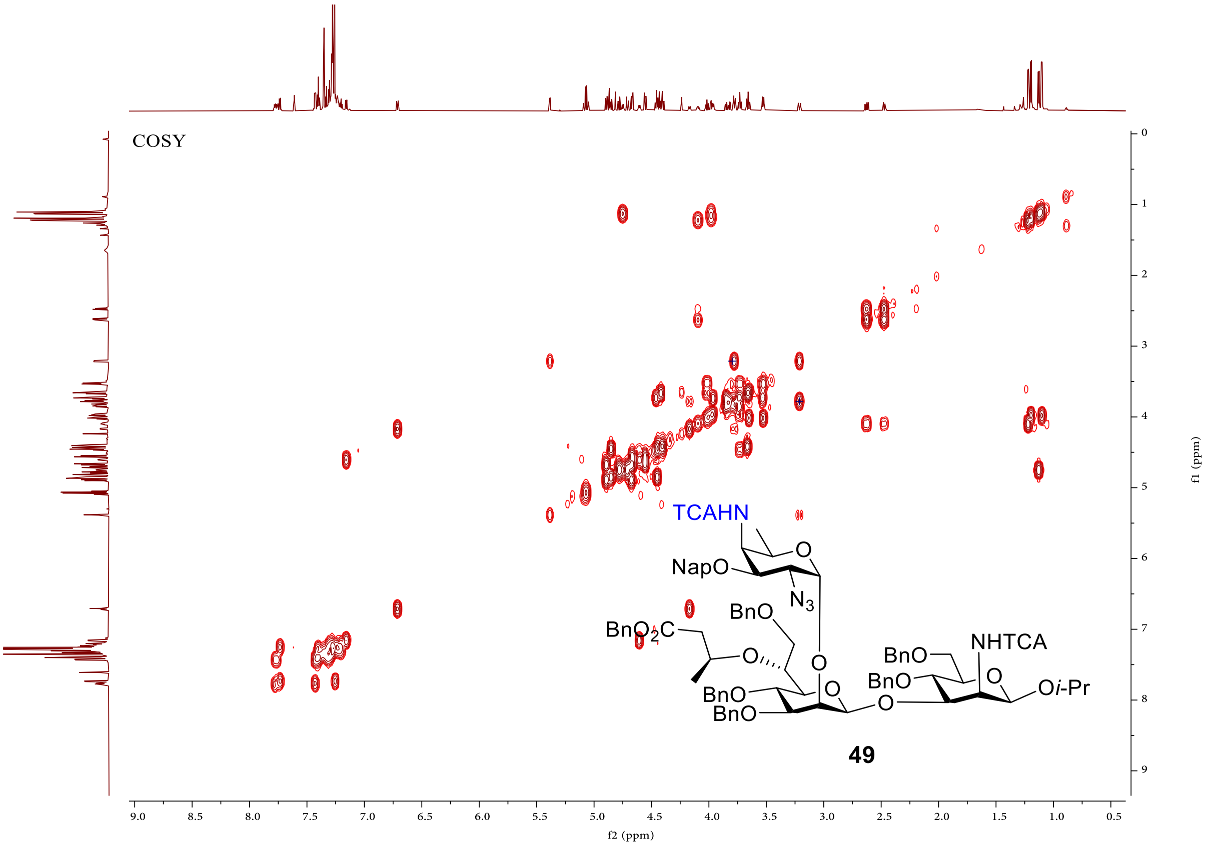
COSY
TCAHN
NapO
N3
BnO
BnO2C
O
BnO
BnO
NHTCA
Oi-Pr
49
f2 (ppm)
f1 (ppm)

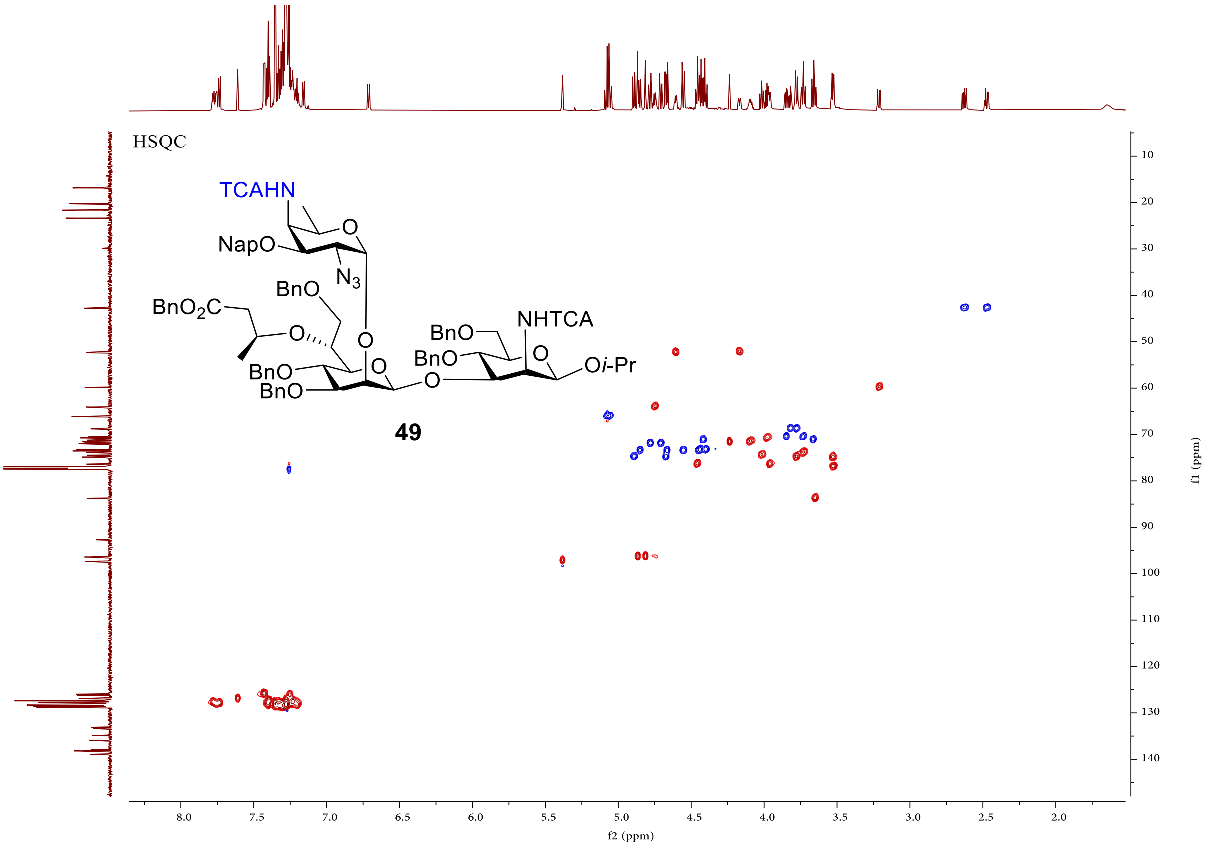
HSQC
TCAHN
NapO
N3
BnO
BnO2C
BnO
BnO
NHTCA
Oi-Pr
49
f2 (ppm)
f1 (ppm)

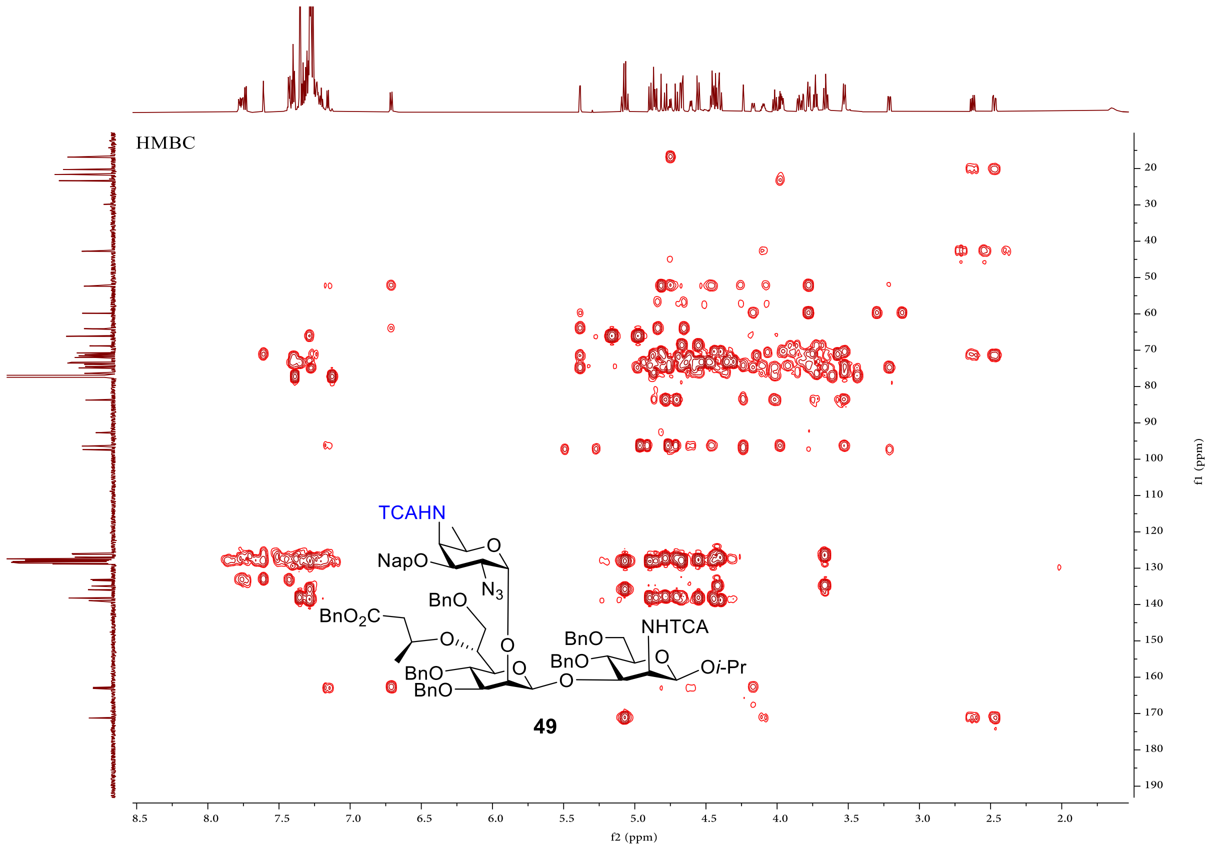
HMBC
TCAHN
NapO
N3
BnO
BnO2C
BnO
BnO
NHTCA
BnO
BnO
Oi-Pr
49
f2 (ppm)
f1 (ppm)

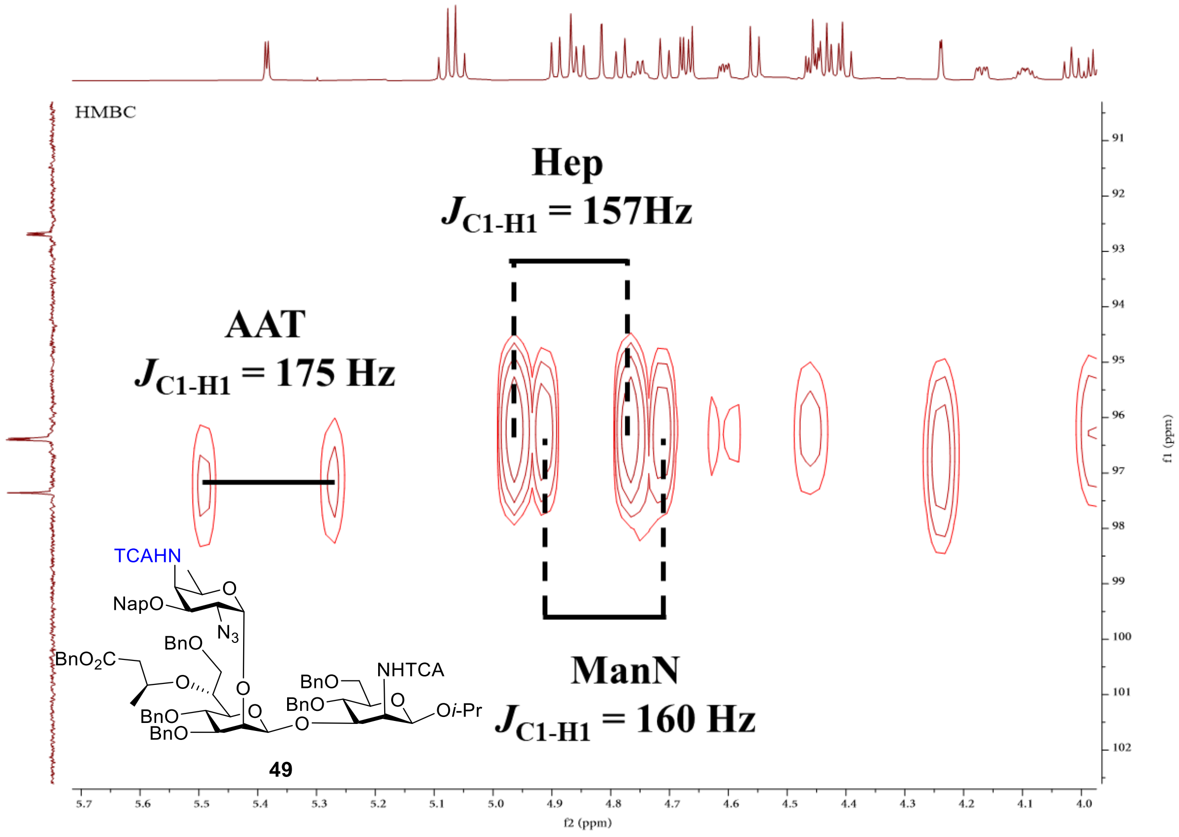
HMBC
Hep
JC1-H1 = 157Hz
AAT
JC1-H1 = 175 Hz
ManN
JC1-H1 = 160 Hz
TCAHN
NapO
N3
BnO
BnO2C
BnO
BnO
NHTCA
BnO
BnO
Oi-Pr
49
f2 (ppm)
f1 (ppm)

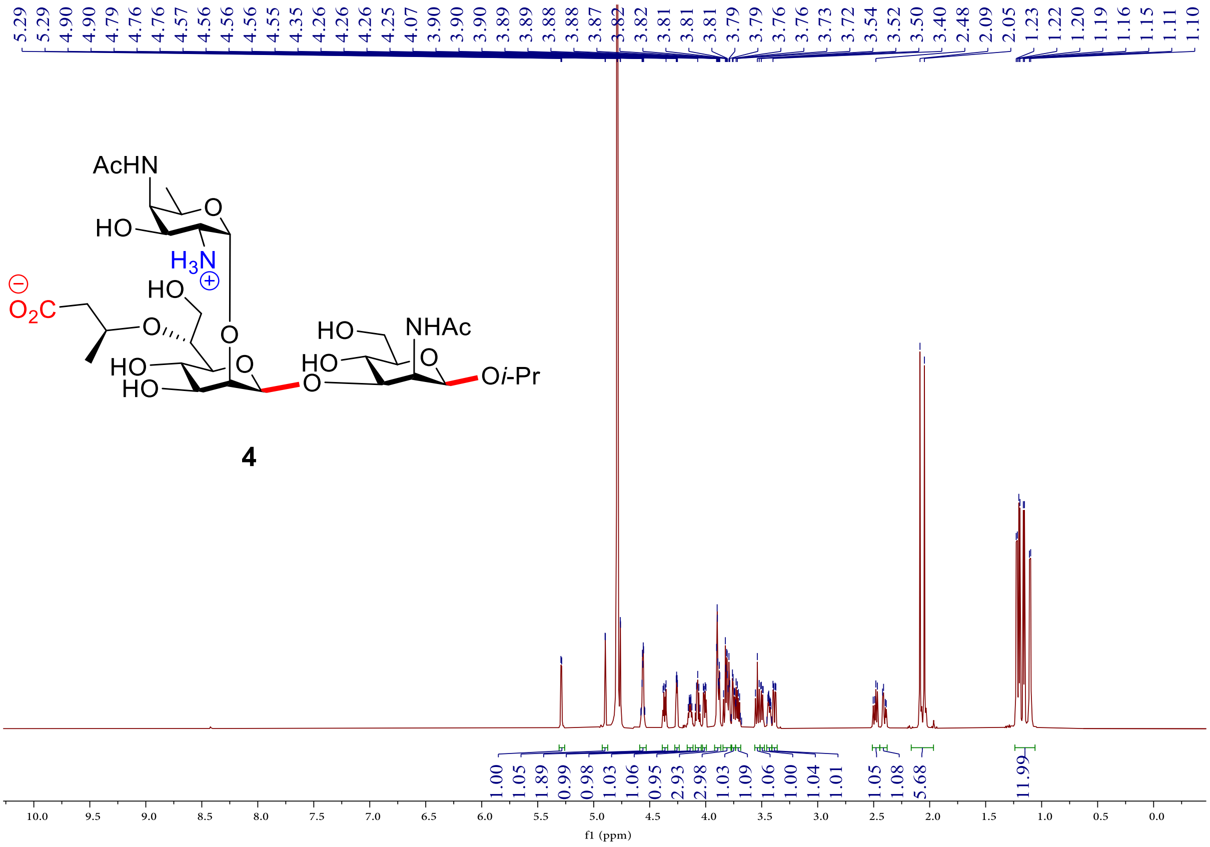
5.29
5.29
4.90
4.90
4.79
4.76
4.76
4.57
4.56
4.56
4.56
4.55
4.35
4.26
4.26
4.26
4.25
4.07
3.90
3.90
3.90
3.89
3.89
3.88
3.88
3.87
3.82
3.82
3.81
3.81
3.81
3.79
3.79
3.76
3.76
3.73
3.72
3.54
3.52
3.50
3.40
2.48
2.09
2.05
1.23
1.22
1.20
1.19
1.16
1.15
1.11
1.10
AcHN
HO
O
H3N
HO
O2C
O
O
HO
NHAc
HO
O
HO
HO
O
Oi-Pr
1.00
1.05
1.89
0.99
0.98
1.03
1.06
0.95
2.93
2.98
1.03
1.09
1.06
1.00
1.04
1.01
1.05
1.08
5.68
11.99
f1 (ppm)

4

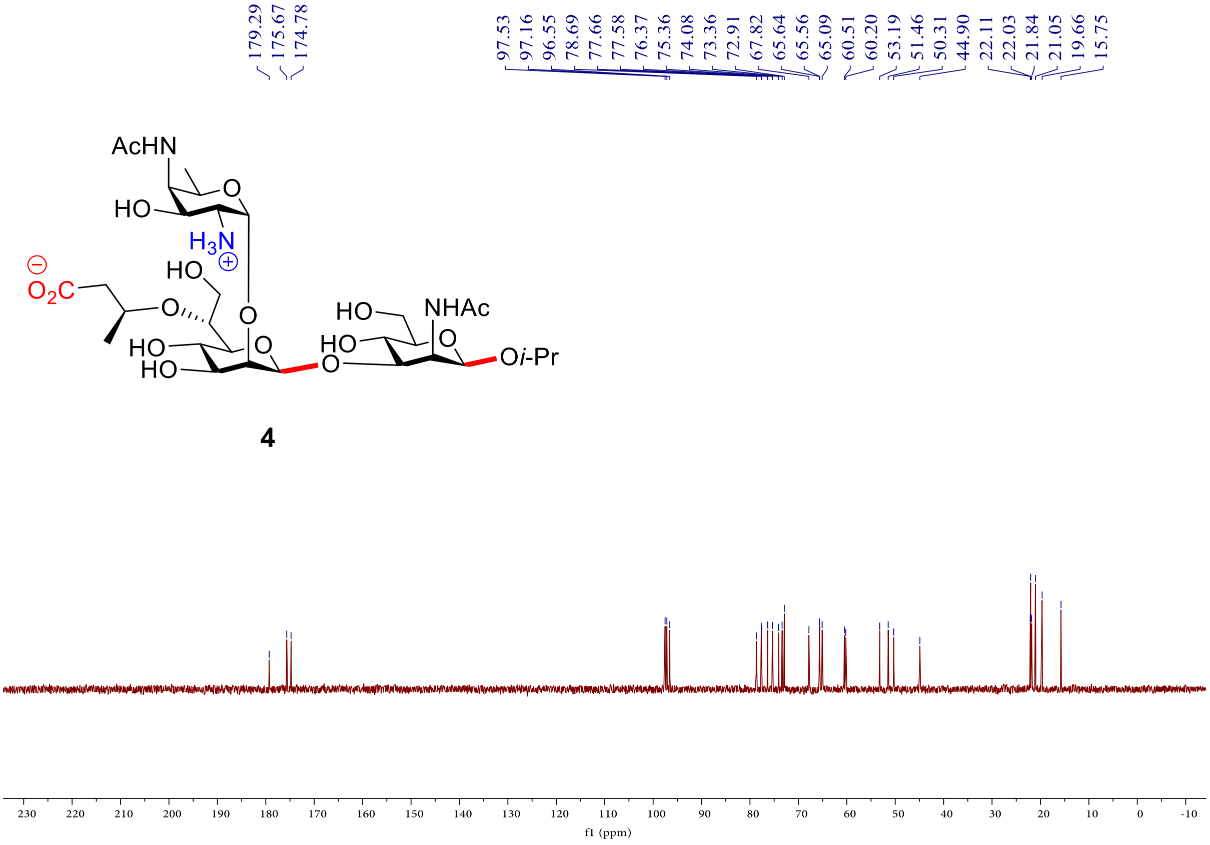
179.29
175.67
174.78
97.53
97.16
96.55
78.69
77.66
77.58
76.37
75.36
74.08
73.36
72.91
67.82
65.64
65.56
65.09
60.51
60.20
53.19
51.46
50.31
44.90
22.11
22.03
21.84
21.05
19.66
15.75
AcHN
HO
O
H3N
HO
O2C
O
O
HO
NHAc
HO
O
HO
HO
O
Oi-Pr
f1 (ppm)

4

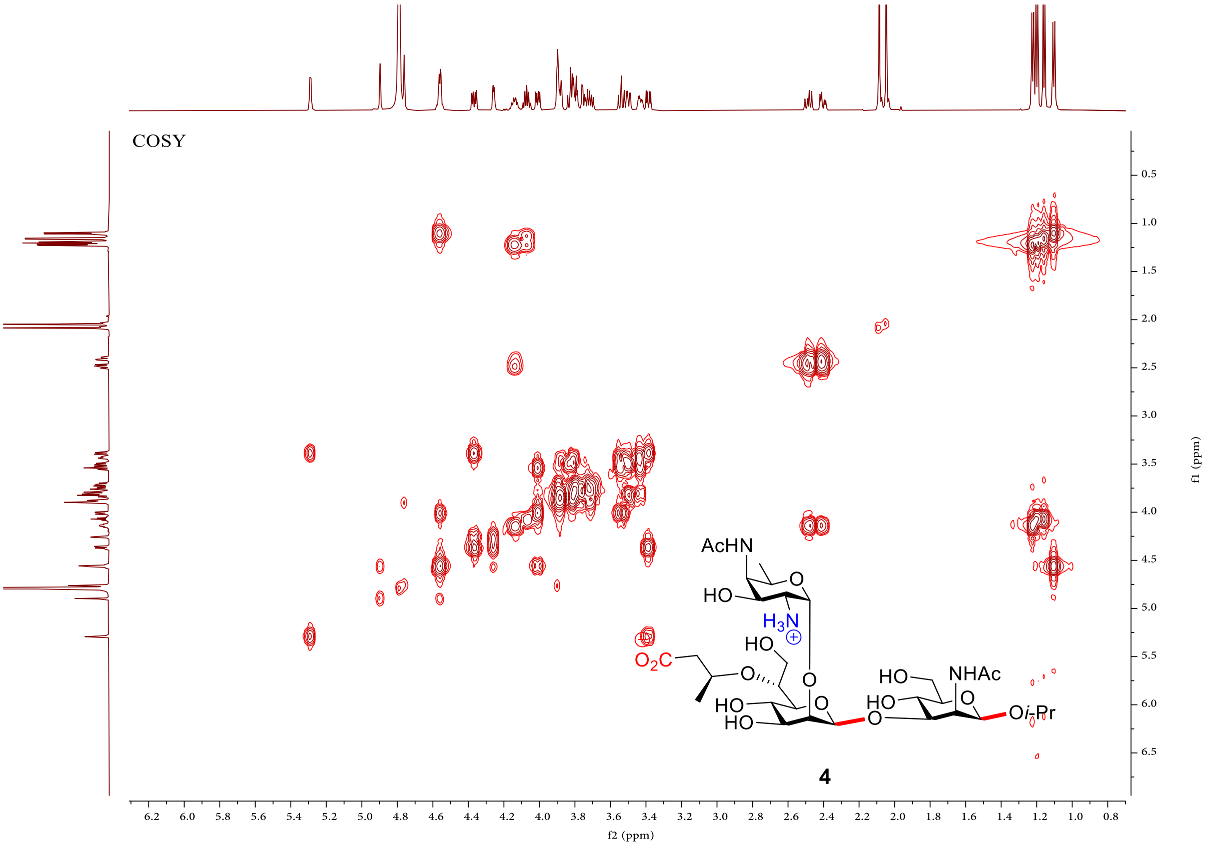
COSY
AcHN
HO
H3N
HO
O2C
HO
HO
HO
NHAc
HO
Oi-Pr
f2 (ppm)
f1 (ppm)

4

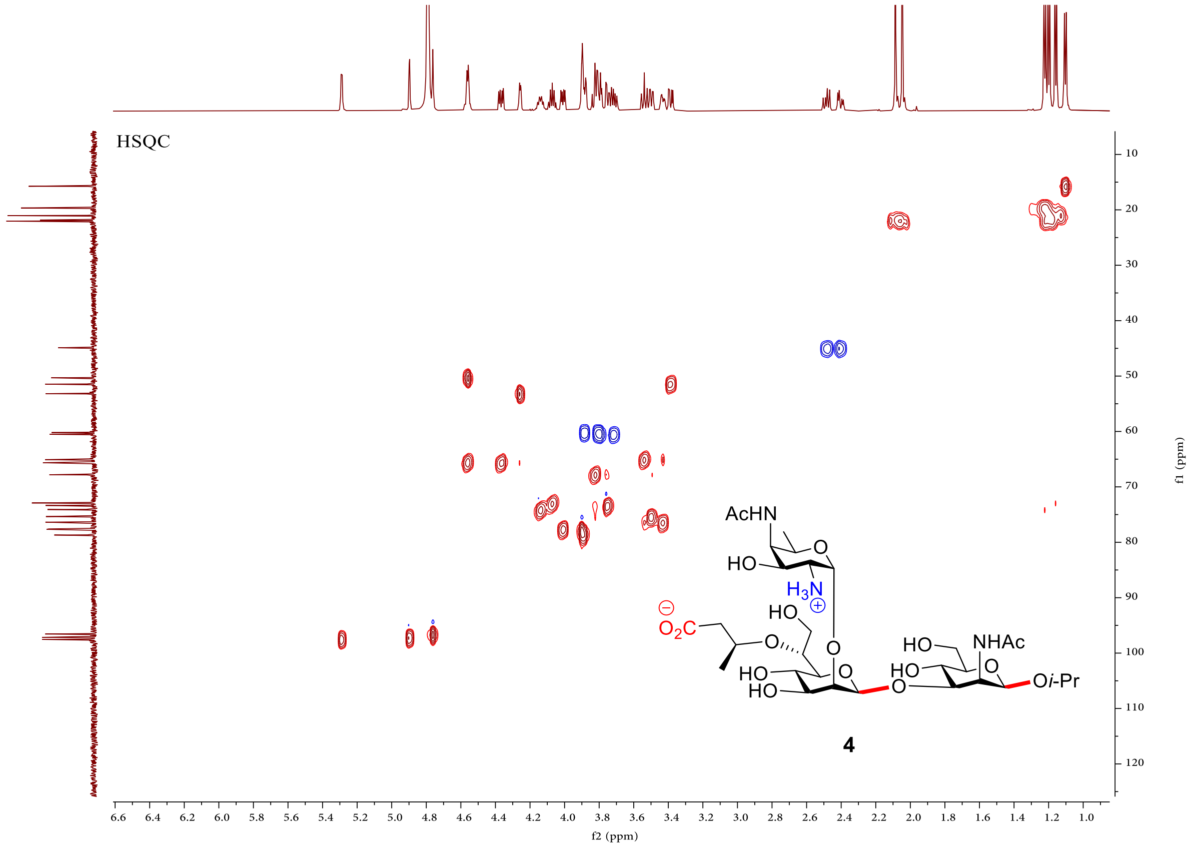
HSQC
AcHN
HO
H3N
HO
O2C
HO
HO
HO
NHAc
HO
Oi-Pr
f2 (ppm)
f1 (ppm)

4

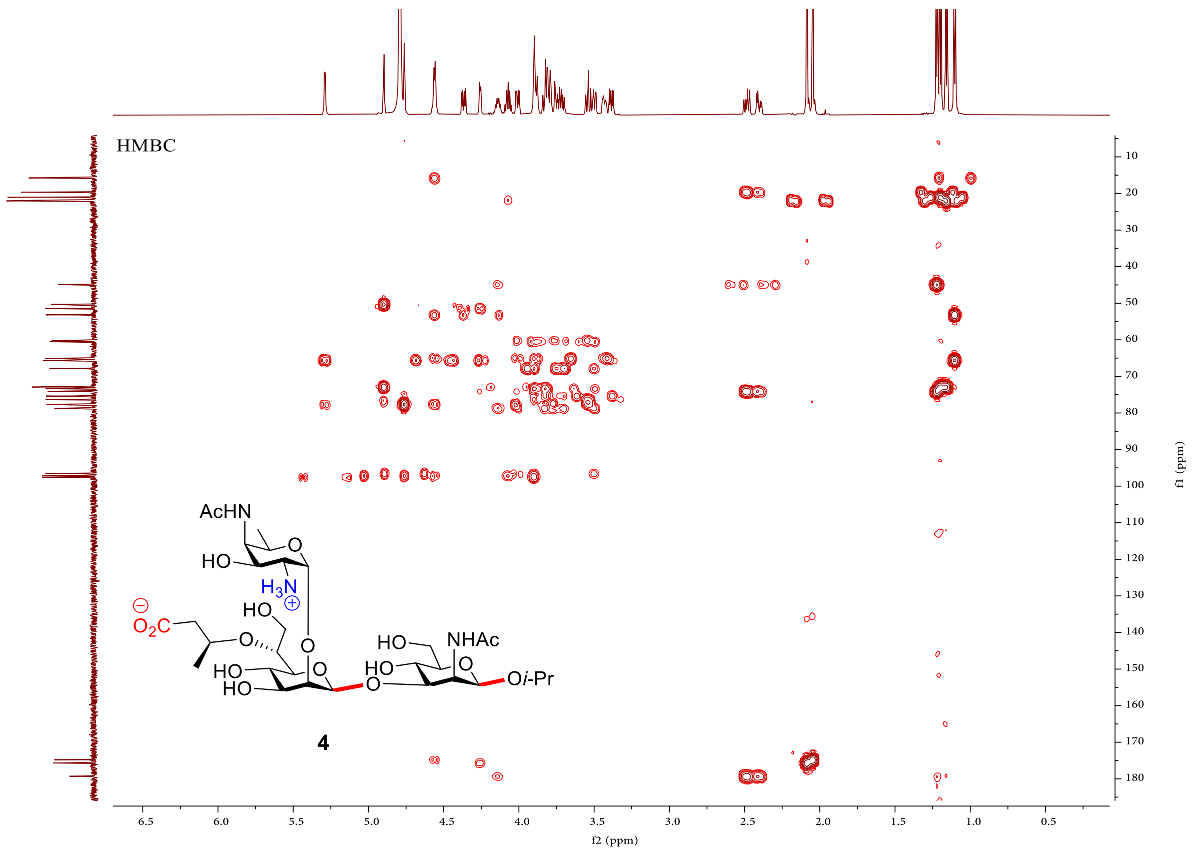
HMBC
AcHN
HO
H3N
HO
O2C
O
O
O
HO
HO
HO
NHAc
HO
O
Oi-Pr
4
f2 (ppm)
f1 (ppm)

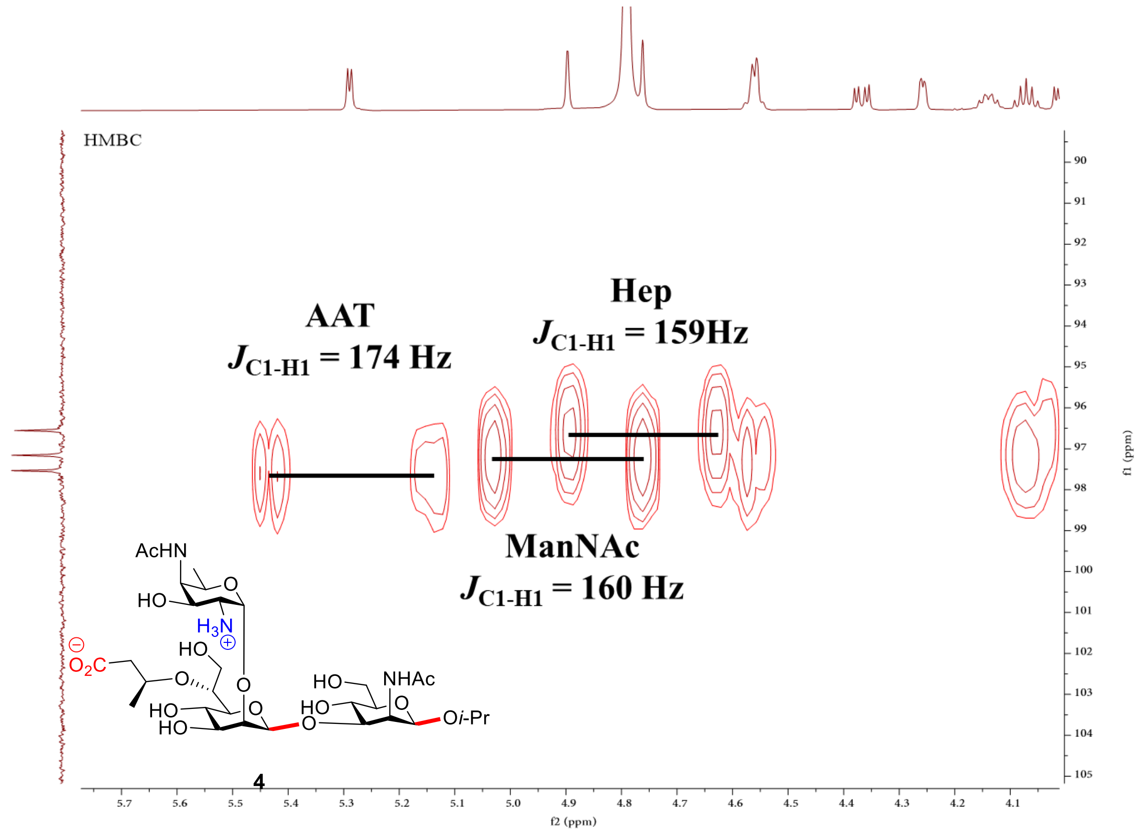
HMBC
AAT
JC1-H1 = 174 Hz
Hep
JC1-H1 = 159Hz
ManNAc
JC1-H1 = 160 Hz
AcHN
HO
H3N
HO
O2C
HO
HO
HO
NHAc
HO
Oi-Pr
4
f2 (ppm)
f1 (ppm)

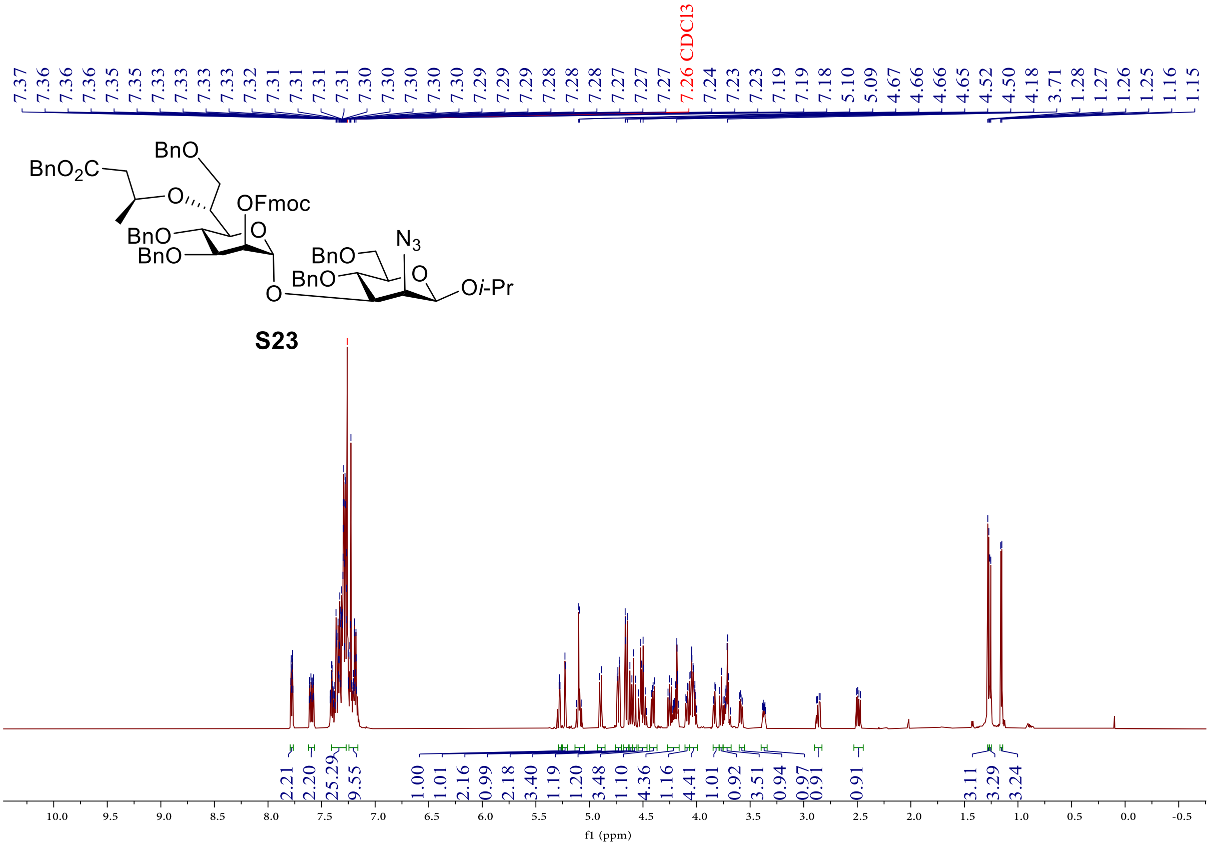
7.37 7.36 7.36 7.36 7.35 7.35 7.33 7.33 7.33 7.33 7.33 7.32 7.31 7.31 7.31 7.31 7.30 7.30 7.30 7.30 7.30 7.29 7.29 7.29 7.28 7.28 7.28 7.27 7.27 7.27 7.26 CDCl3 7.24 7.23 7.23 7.19 7.19 7.18 5.10 5.09 4.67 4.66 4.66 4.65 4.52 4.50 4.18 3.71 1.28 1.27 1.26 1.25 1.16 1.15
BnO
BnO2C
OFmoc
O
BnO
BnO
N3
BnO
BnO
O
Oi-Pr
O
S23
2.21 2.20 25.29 9.55 1.00 1.01 2.16 0.99 2.18 3.40 1.19 1.20 3.48 1.10 4.36 1.16 4.41 1.01 0.92 3.51 0.94 0.97 0.91 0.91 3.11 3.29 3.24
f1 (ppm)

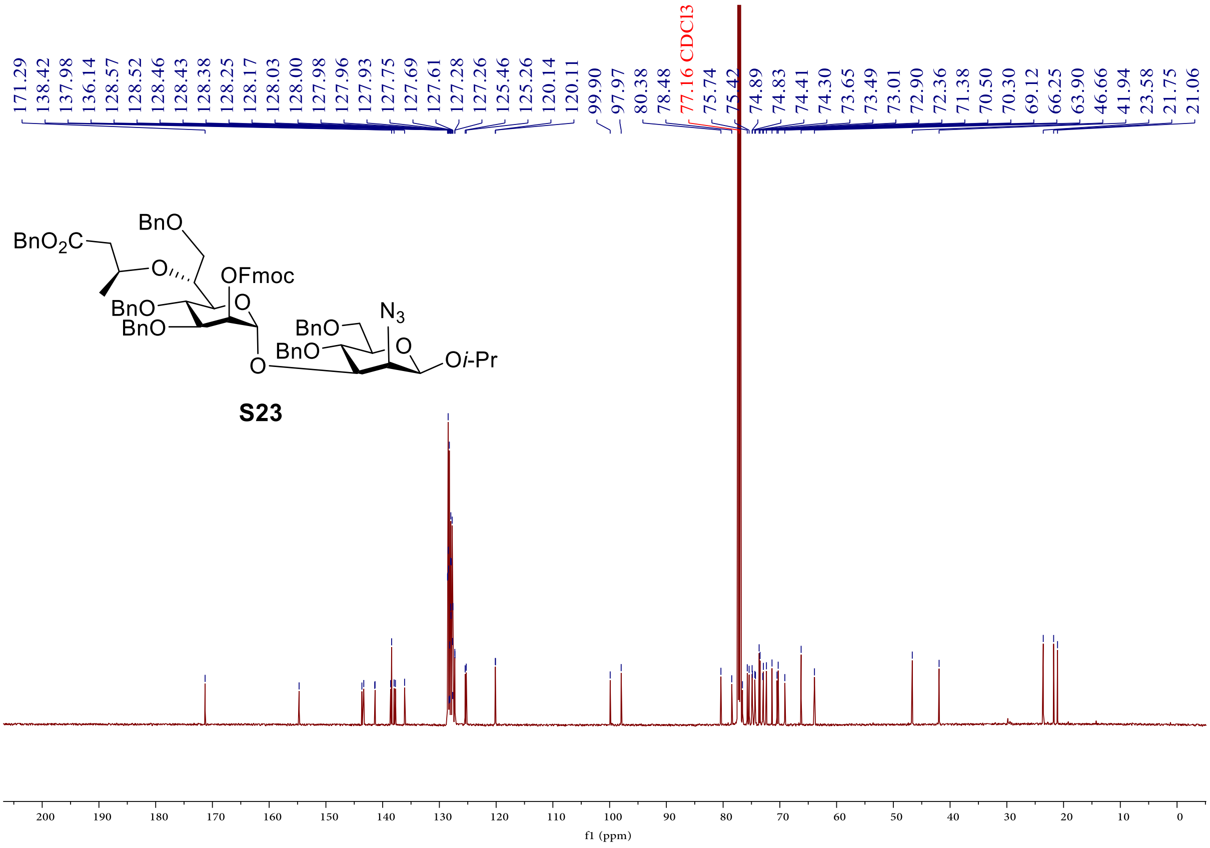
171.29 138.42 137.98 136.14 128.57 128.52 128.46 128.43 128.38 128.25 128.17 128.03 128.00 127.98 127.96 127.93 127.75 127.69 127.61 127.28 127.26 125.46 125.26 120.14 120.11 99.90 97.97 80.38 78.48 77.16 CDCl3 75.74 75.42 74.89 74.83 74.41 74.30 73.65 73.49 73.01 72.90 72.36 71.38 70.50 70.30 69.12 66.25 63.90 46.66 41.94 23.58 21.75 21.06
BnO
BnO2C
OFmoc
O
BnO
BnO
N3
BnO
BnO
O
Oi-Pr
O
S23
f1 (ppm)

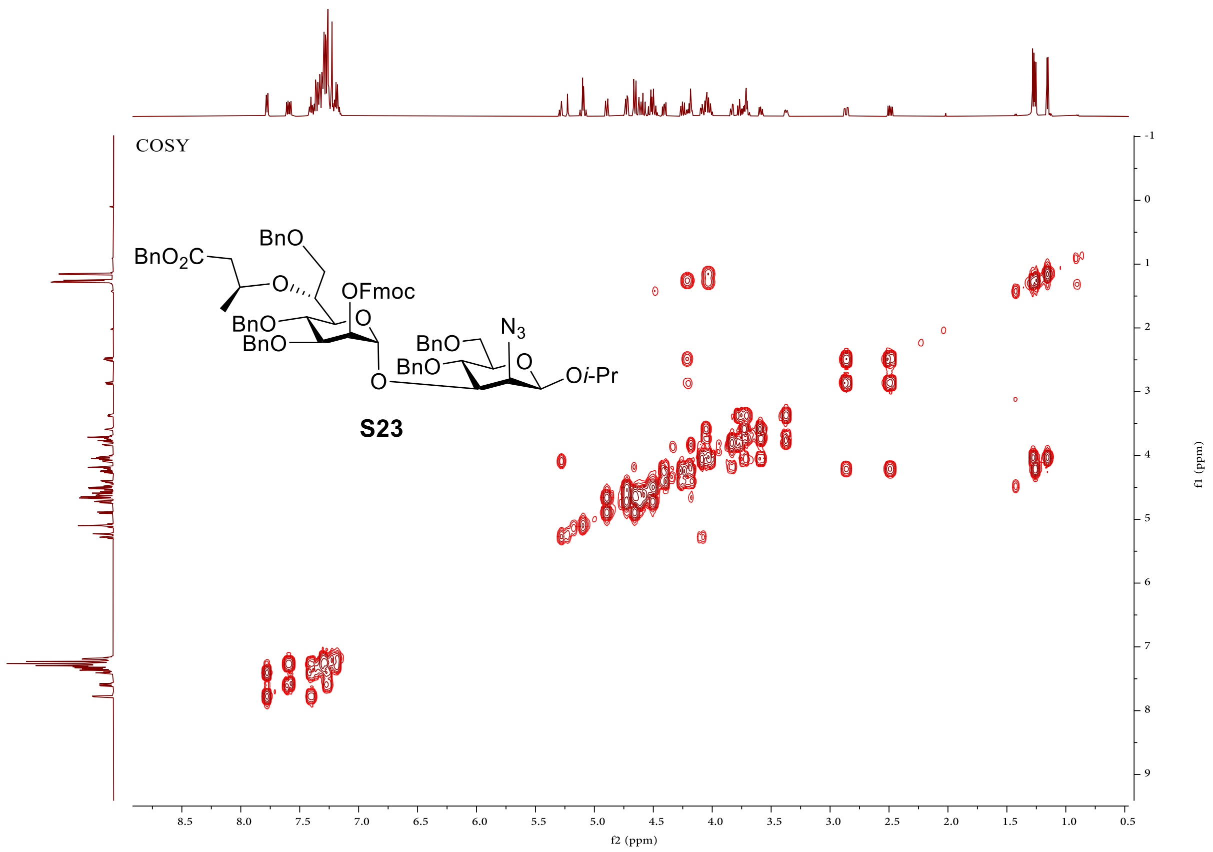
COSY
BnO
BnO2C
O
OFmoc
O
BnO
BnO
BnO
N3
BnO
O
Oi-Pr
O
S23
f2 (ppm)
f1 (ppm)

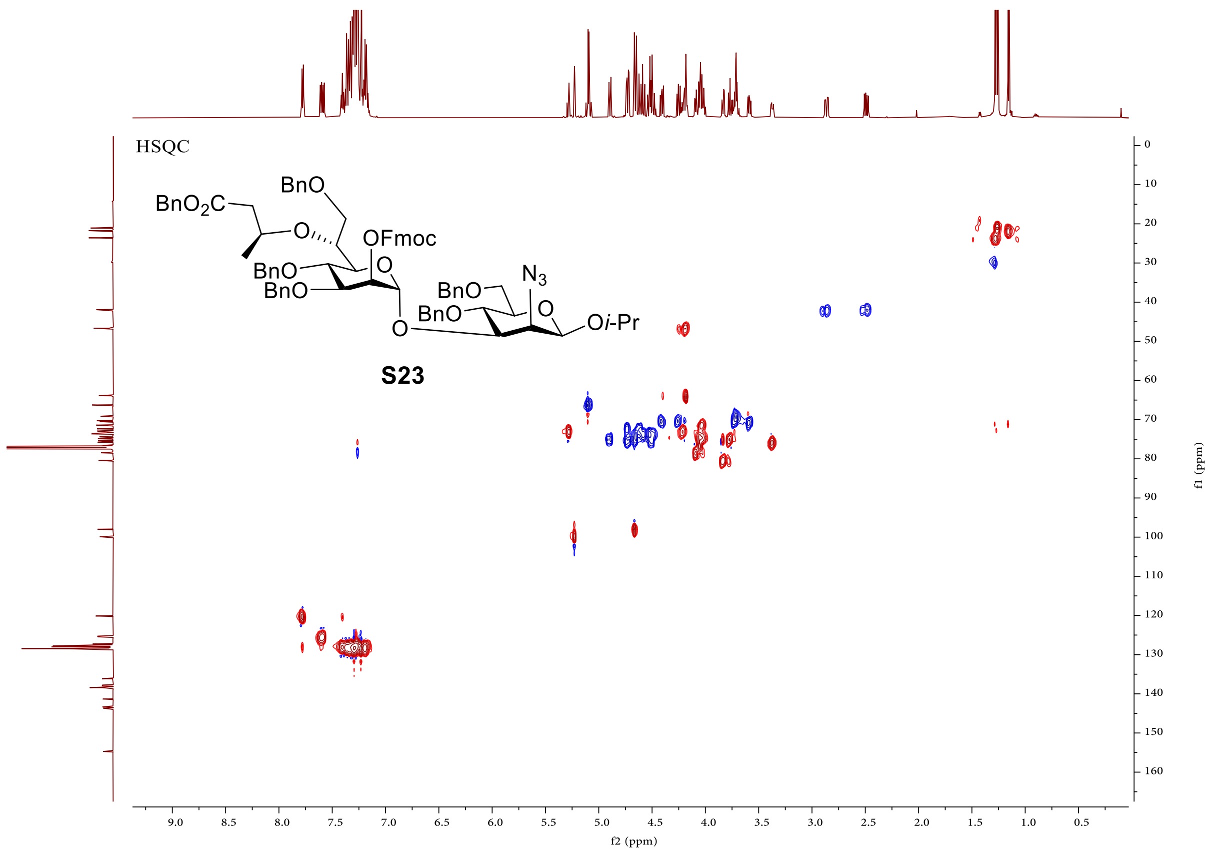
HSQC
BnO
BnO2C
O
OFmoc
O
BnO
BnO
BnO
N3
BnO
O
Oi-Pr
O
S23
f2 (ppm)
f1 (ppm)

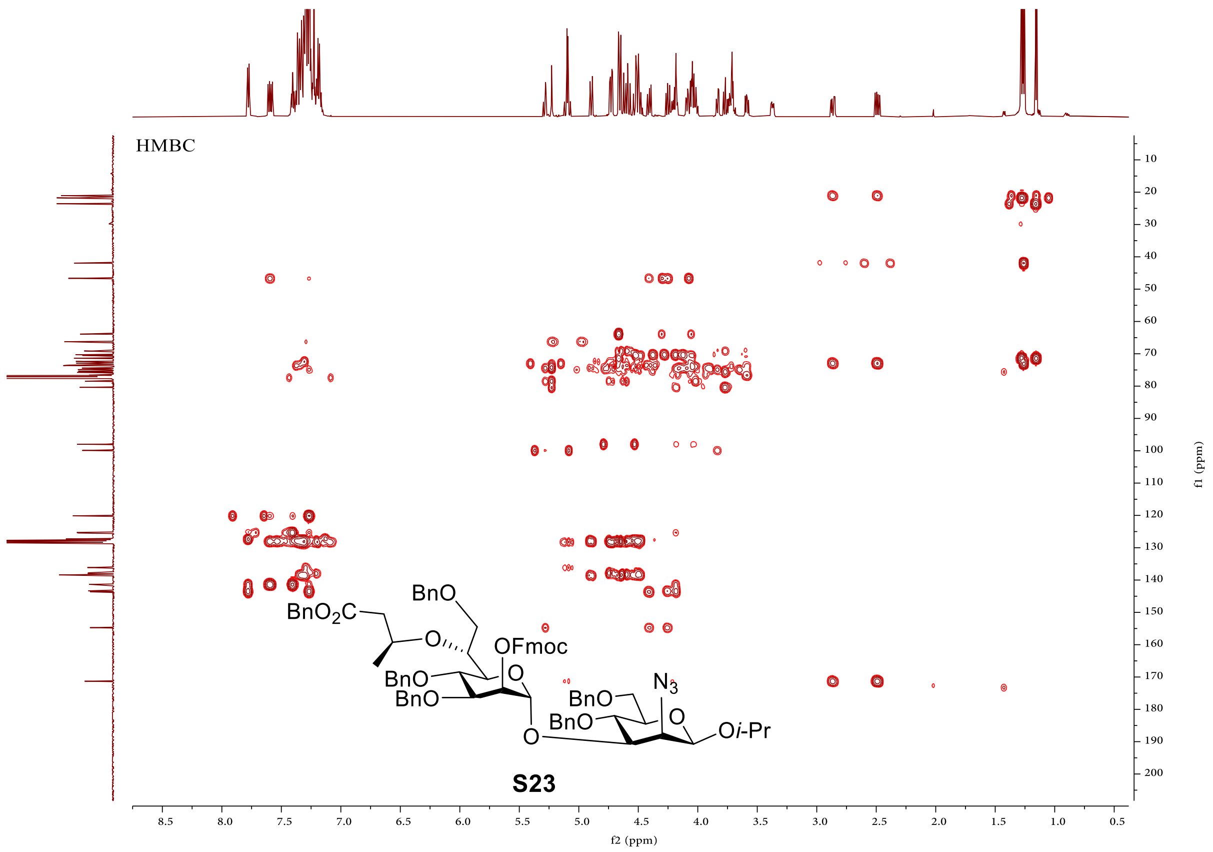
HMBC
BnO
BnO2C
OFmoc
BnO
BnO
O
BnO
N3
BnO
O
Oi-Pr
O
S23
f2 (ppm)
f1 (ppm)

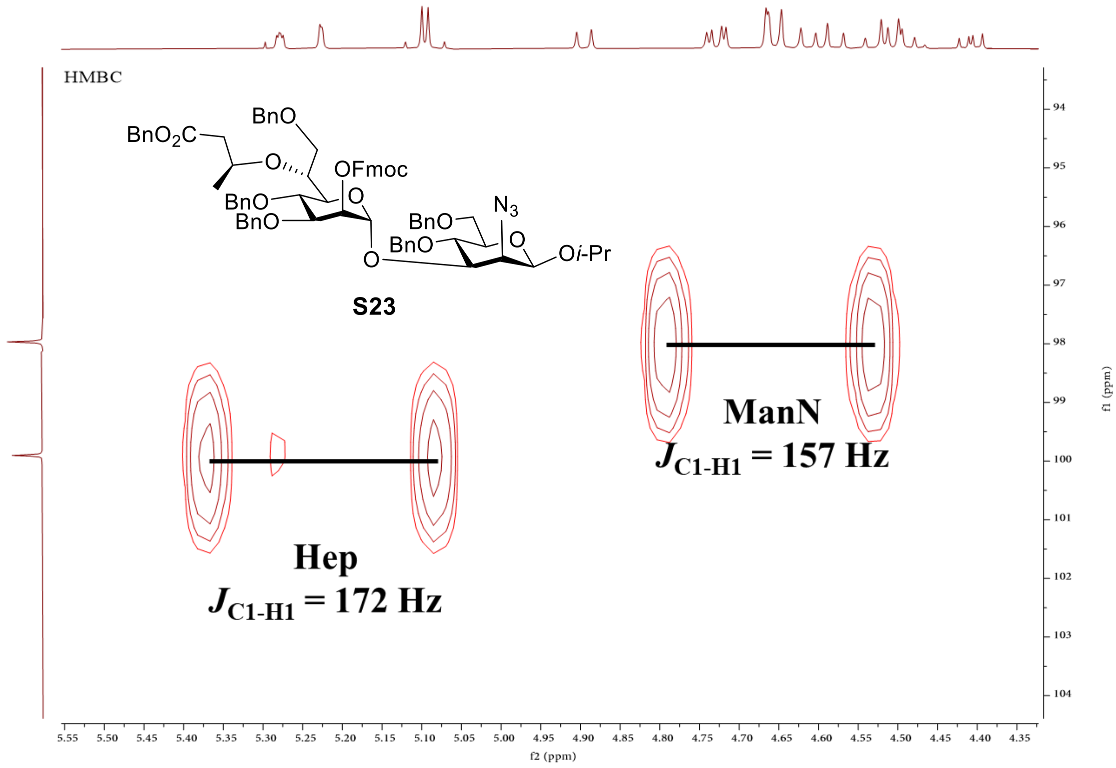
HMBC
BnO
BnO2C
OFmoc
BnO
BnO
O
BnO
N3
BnO
O
Oi-Pr
O
S23
ManN
JC1-H1 = 157 Hz
Hep
JC1-H1 = 172 Hz
f2 (ppm)
f1 (ppm)

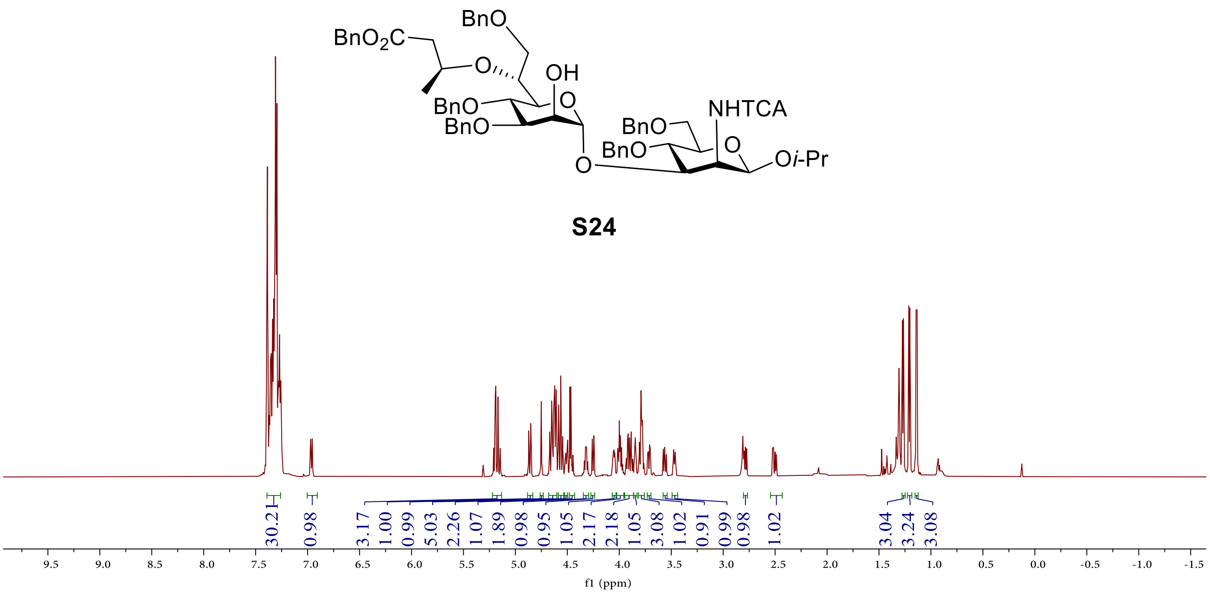

BnO$_2$C
BnO
OH
BnO
BnO
O
BnO
BnO
NHTCA
O
O
O
O*i*-Pr
S24
30.21
0.98
3.17
1.00
0.99
5.03
2.26
1.07
1.89
0.98
0.95
1.05
2.17
2.18
1.05
3.08
1.02
0.91
0.99
0.98
1.02
3.04
3.24
3.08
9.5 9.0 8.5 8.0 7.5 7.0 6.5 6.0 5.5 5.0 4.5 4.0 3.5 3.0 2.5 2.0 1.5 1.0 0.5 0.0 -0.5 -1.0 -1.5
f1 (ppm)

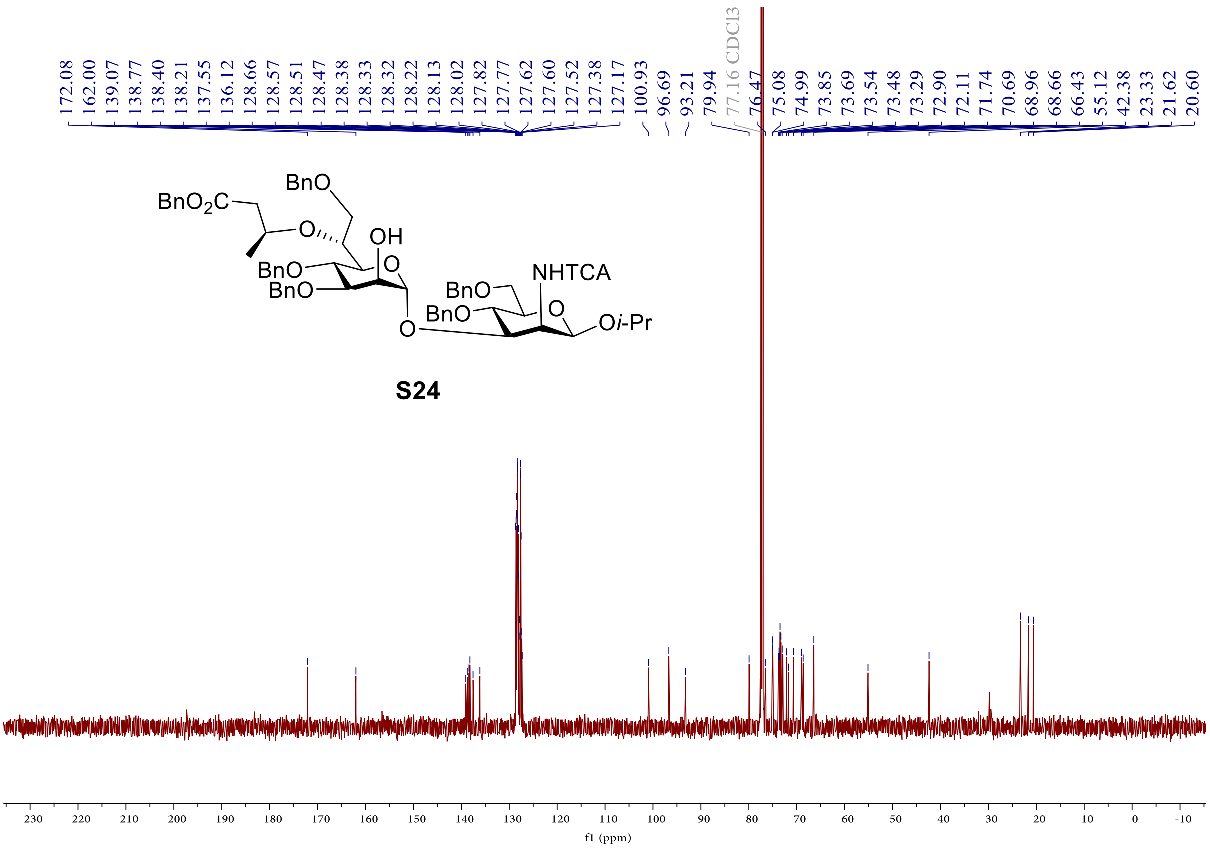

172.08
162.00
139.07
138.77
138.40
138.21
137.55
136.12
128.66
128.57
128.51
128.47
128.38
128.33
128.32
128.22
128.13
128.02
127.82
127.77
127.62
127.60
127.52
127.38
127.17
100.93
96.69
93.21
79.94
77.16 CDCl3
76.47
75.08
74.99
73.85
73.69
73.54
73.48
73.29
72.90
72.11
71.74
70.69
68.96
68.66
66.43
55.12
42.38
23.33
21.62
20.60
BnO$_2$C
BnO
OH
BnO
BnO
O
BnO
BnO
NHTCA
O
O
O
O*i*-Pr
S24
230 220 210 200 190 180 170 160 150 140 130 120 110 100 90 80 70 60 50 40 30 20 10 0 -10
f1 (ppm)

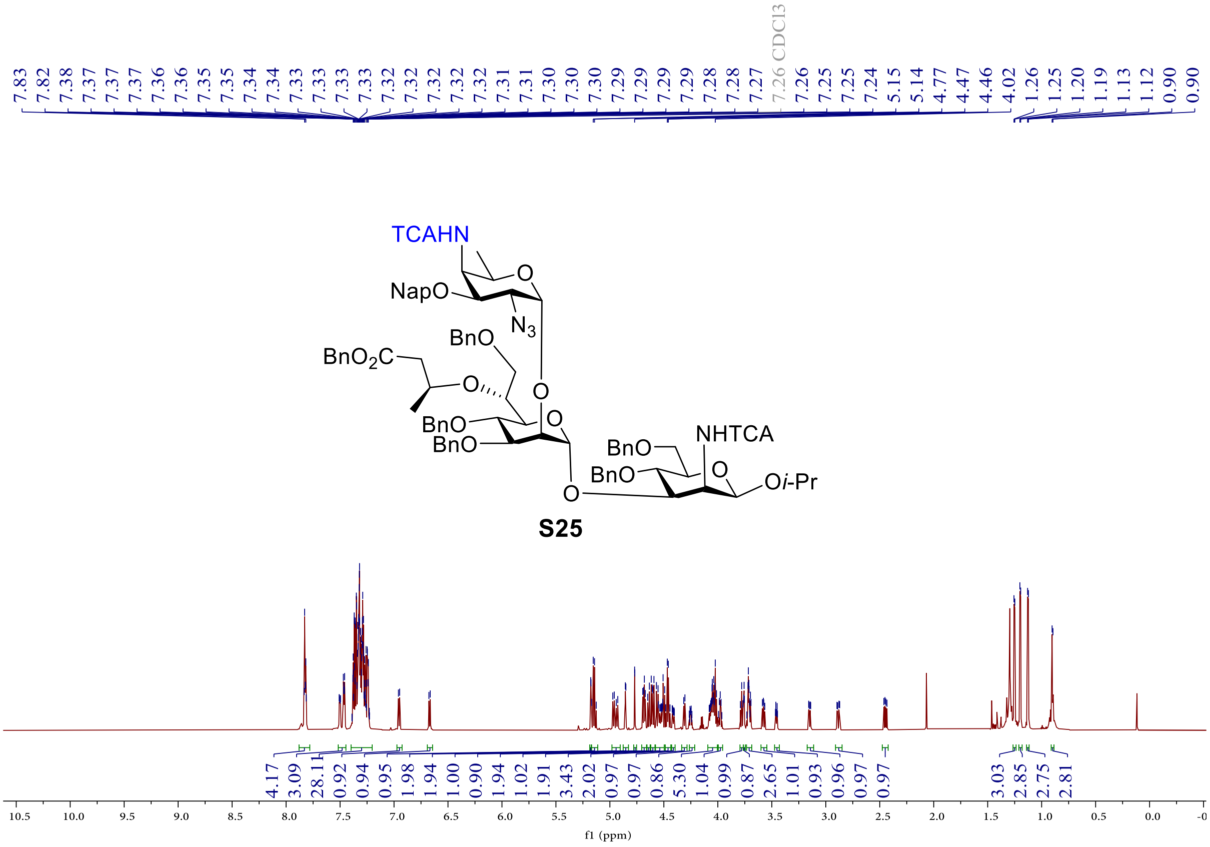
7.83 7.82 7.38 7.37 7.37 7.37 7.36 7.36 7.35 7.35 7.34 7.34 7.33 7.33 7.33 7.33 7.32 7.32 7.32 7.32 7.32 7.31 7.31 7.30 7.30 7.30 7.29 7.29 7.29 7.29 7.28 7.28 7.27 7.26 CDCl3 7.26 7.25 7.25 7.24 5.15 5.14 4.77 4.47 4.46 4.02 1.26 1.25 1.20 1.19 1.13 1.12 0.90 0.90
TCAHN
NapO
N3
BnO
BnO2C
O
BnO
BnO
NHTCA
BnO
BnO
Oi-Pr
S25
4.17 3.09 28.11 0.92 0.94 0.95 1.98 1.94 1.00 0.90 1.94 1.02 1.91 3.43 2.02 0.97 0.97 0.86 5.30 1.04 0.99 0.87 2.65 1.01 0.93 0.96 0.97 0.97 3.03 2.85 2.75 2.81
f1 (ppm)

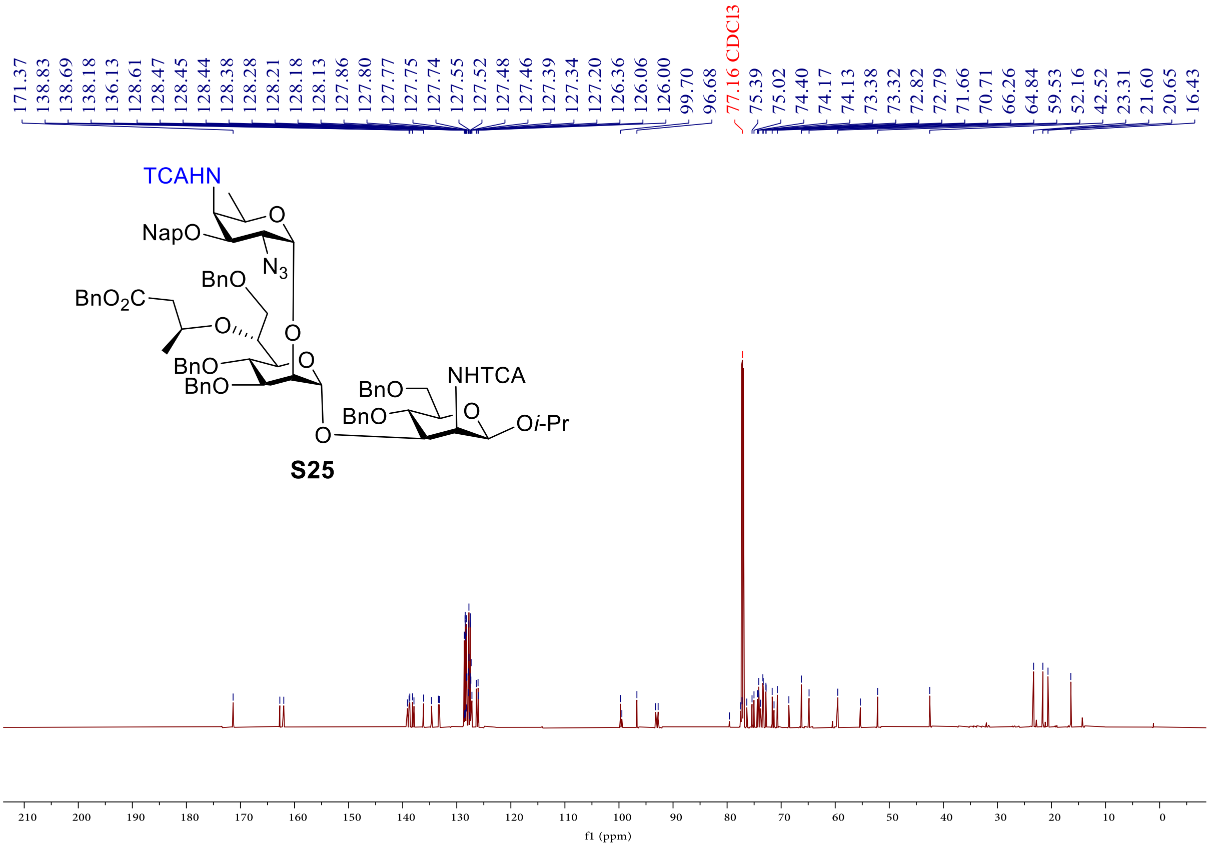
171.37 138.83 138.69 138.18 136.13 128.61 128.47 128.45 128.44 128.38 128.28 128.21 128.18 128.13 127.86 127.80 127.77 127.75 127.74 127.55 127.52 127.48 127.46 127.39 127.34 127.20 126.36 126.06 126.00 99.70 96.68 77.16 CDCl3 75.39 75.02 74.40 74.17 74.13 73.38 73.32 72.82 72.79 71.66 70.71 66.26 64.84 59.53 52.16 42.52 23.31 21.60 20.65 16.43
TCAHN
NapO
N3
BnO
BnO2C
O
BnO
BnO
NHTCA
BnO
BnO
Oi-Pr
S25
f1 (ppm)

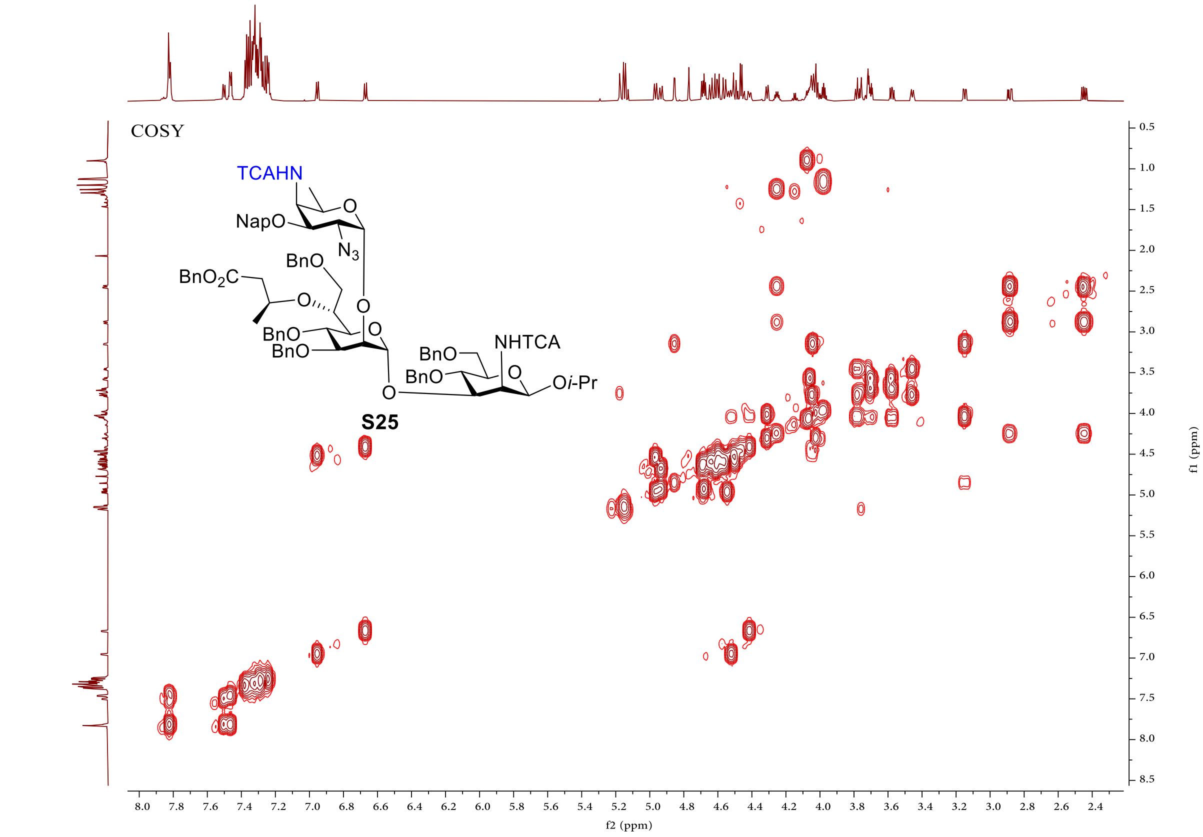
COSY
TCAHN
NapO
N3
BnO
BnO2C
O
BnO
BnO
NHTCA
Oi-Pr
S25
f2 (ppm)
f1 (ppm)

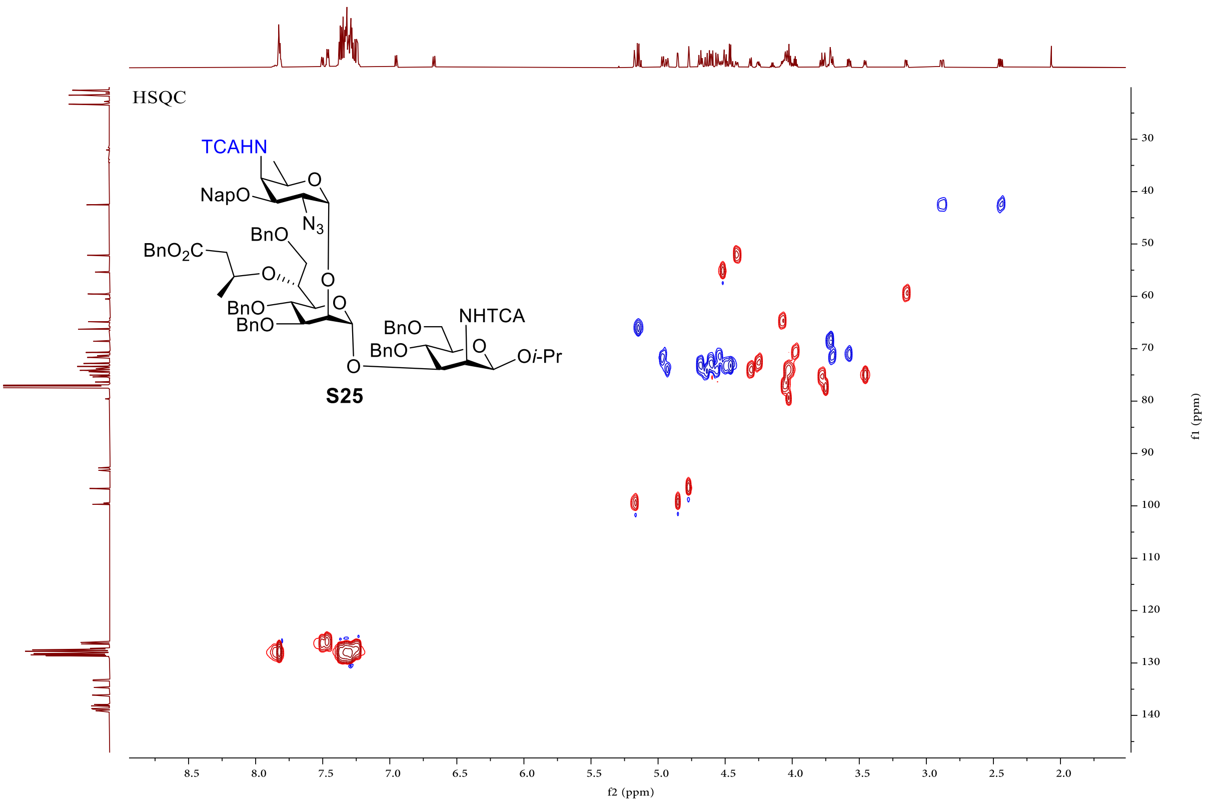
HSQC
TCAHN
NapO
N3
BnO
BnO2C
O
BnO
BnO
NHTCA
Oi-Pr
S25
f2 (ppm)
f1 (ppm)

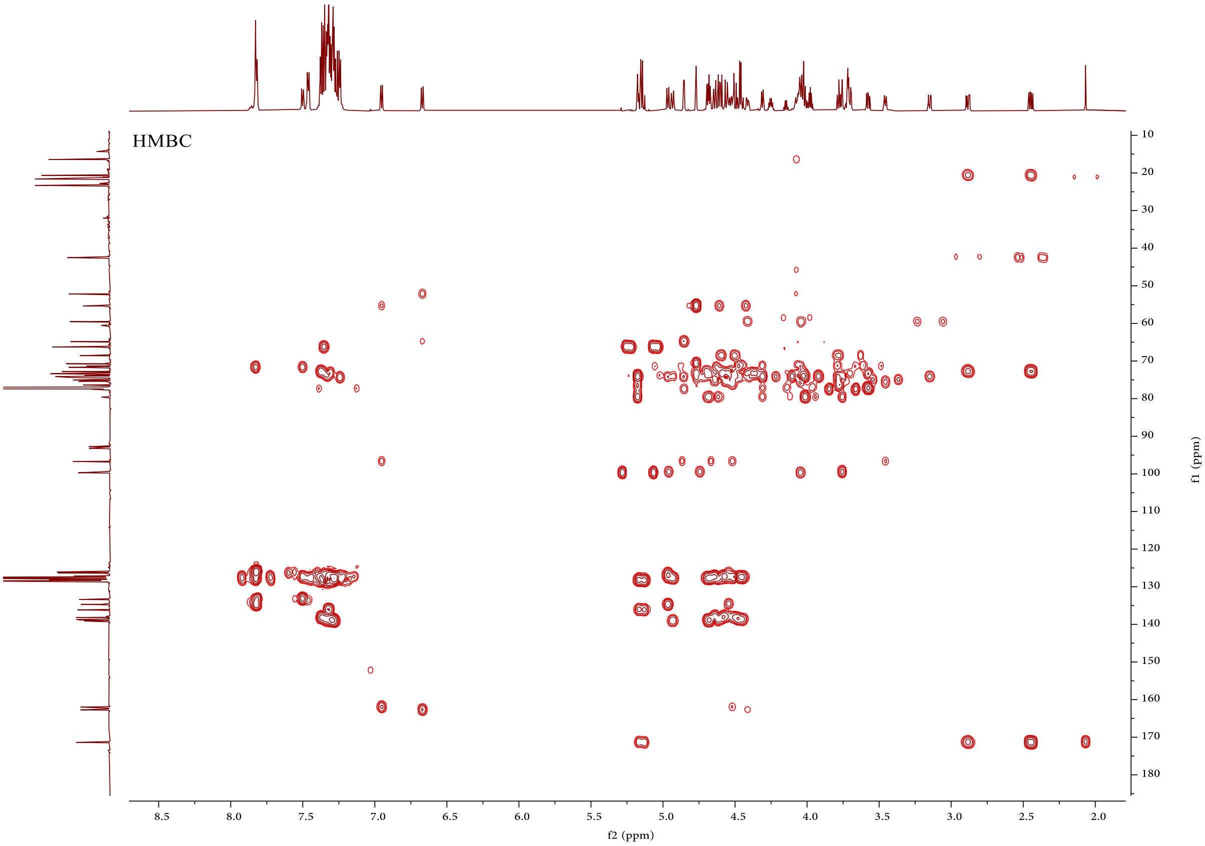
HMBC
f2 (ppm)
f1 (ppm)

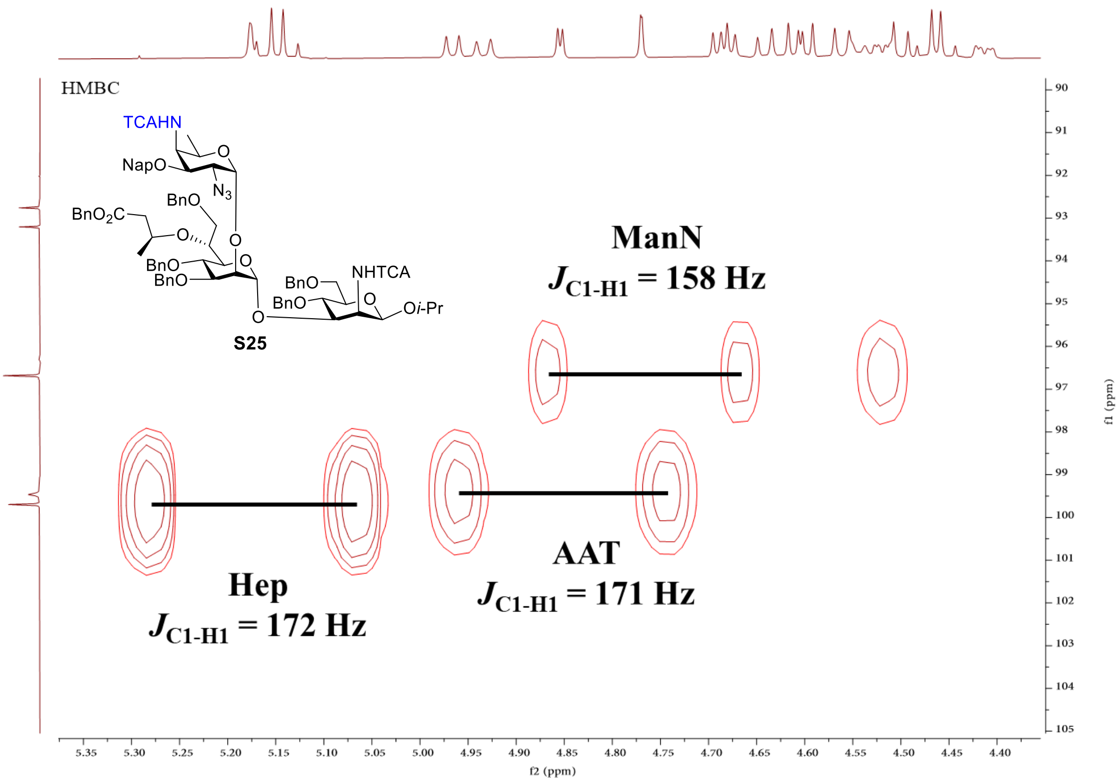
HMBC
TCAHN
NapO
N3
BnO
BnO2C
BnO
BnO
O
NHTCA
Oi-Pr
S25
ManN
JC1-H1 = 158 Hz
AAT
JC1-H1 = 171 Hz
Hep
JC1-H1 = 172 Hz
f2 (ppm)
f1 (ppm)

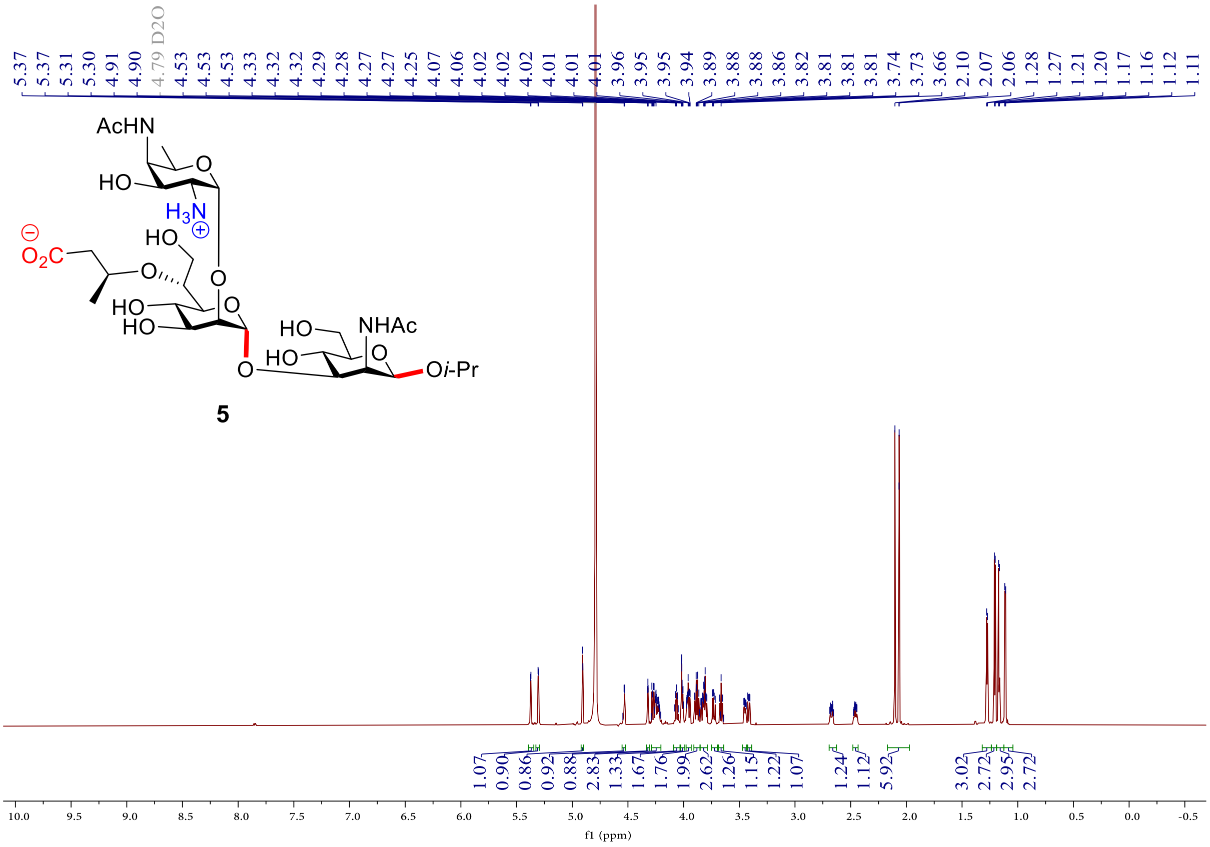

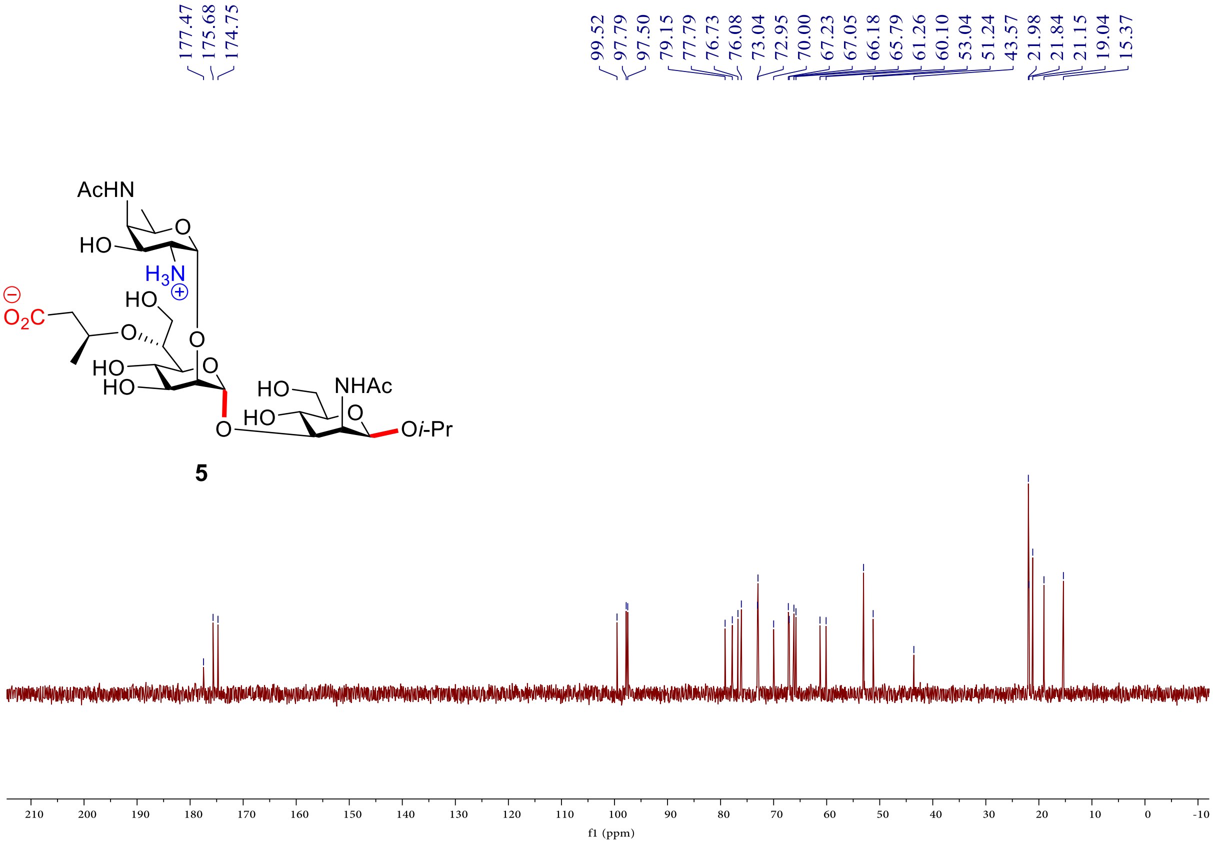

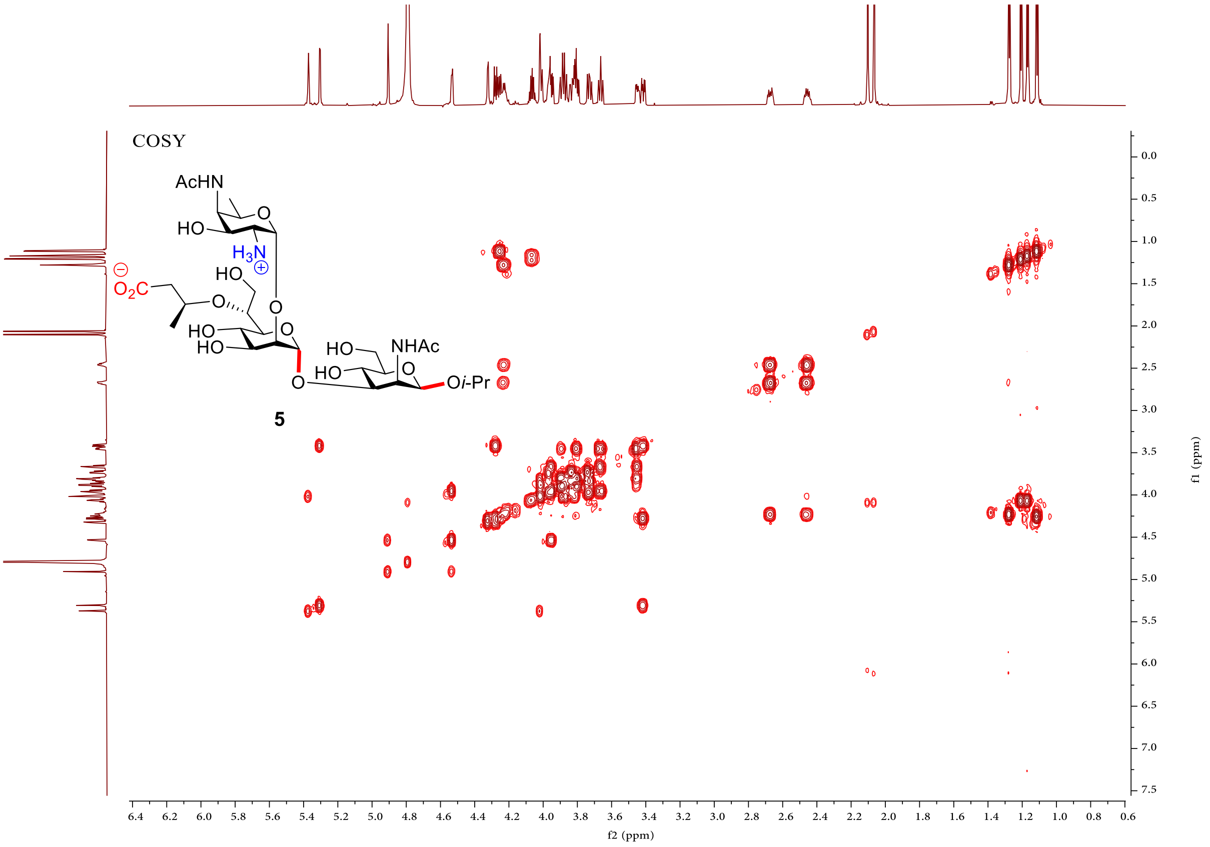
COSY
AcHN
HO
O
H_3N
HO
O_2C
O
O
HO
HO
O
HO
NHAc
HO
O
O
Oi-Pr
5
f2 (ppm)
f1 (ppm)

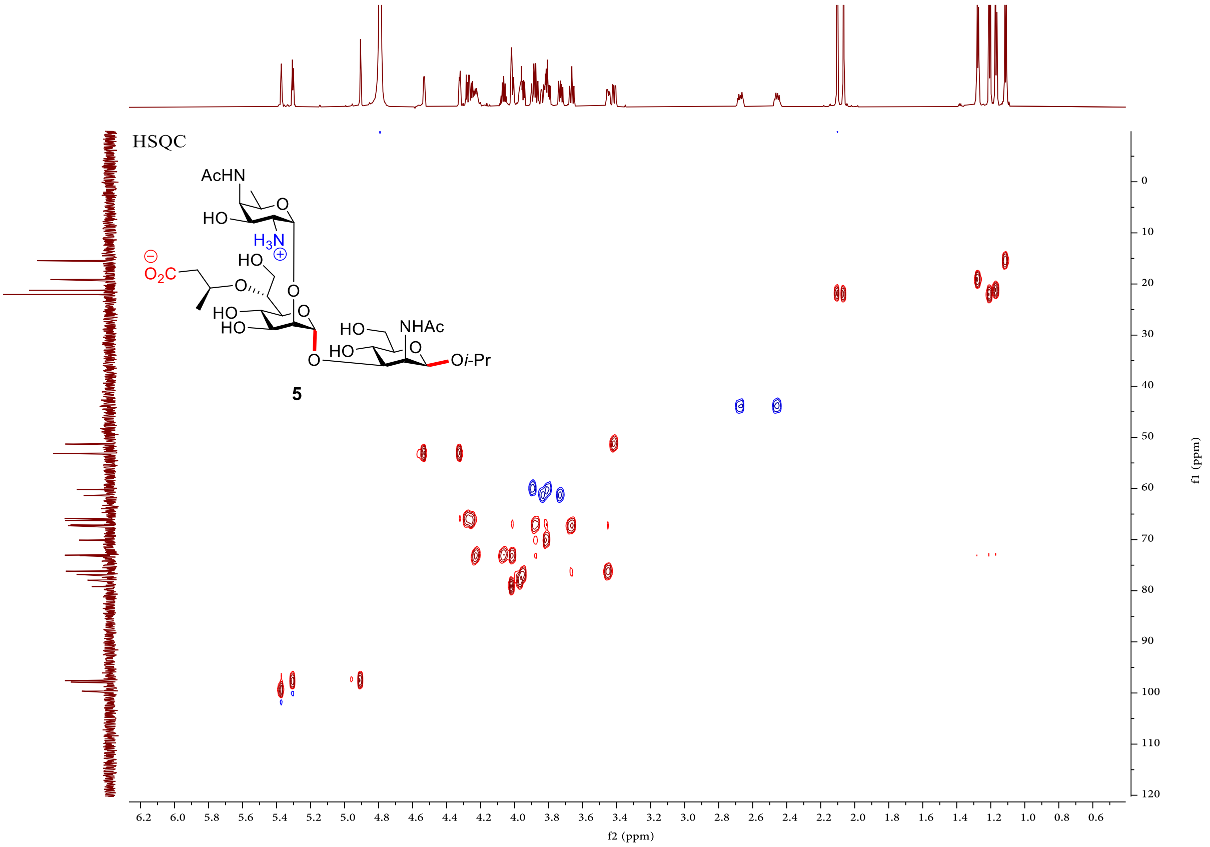
HSQC
AcHN
HO
O
H_3N
HO
O_2C
O
O
HO
HO
O
HO
NHAc
HO
O
O
Oi-Pr
5
f2 (ppm)
f1 (ppm)

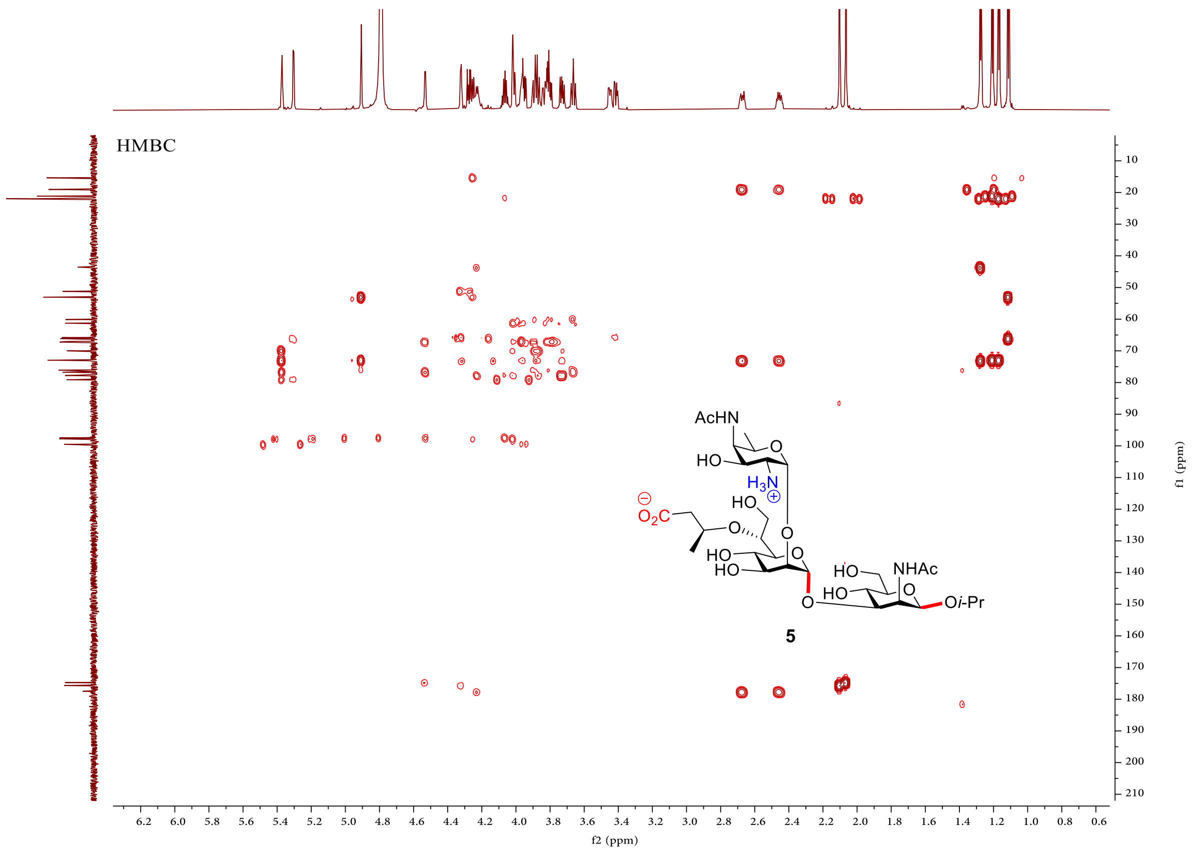
HMBC
AcHN
HO
H_3N
HO
O_2C
O
HO
HO
O
HO
NHAc
HO
O
Oi-Pr
5
f1 (ppm)
f2 (ppm)

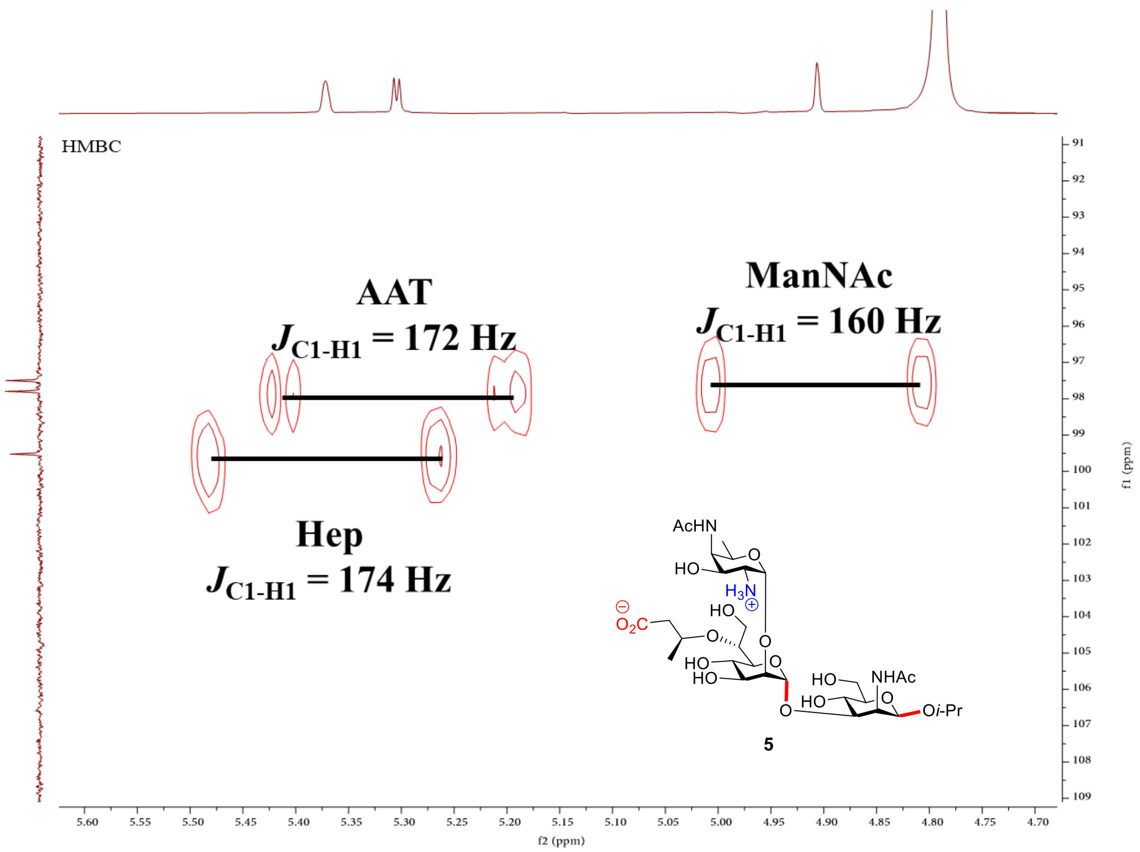
HMBC
AAT
J_{C1-H1} = 172 Hz
ManNAc
J_{C1-H1} = 160 Hz
Hep
J_{C1-H1} = 174 Hz
AcHN
HO
H_3N
HO
O_2C
O
HO
HO
O
HO
NHAc
HO
O
Oi-Pr
5
f1 (ppm)
f2 (ppm)

**Data Filename**	ESIH202402987-1.d	**Sample Name**	G0-G0-hth-4-131
**Sample ID**		**Position**	P1-A1
**Instrument Name**	Agilent 6520 Q-TOF	**Acq Method**	20160322_MS_ESIH_POS_1min.m
**Acquired Time**	5/31/2024 3:34:10 PM	**DA Method**	ESI-HR-20231114.m
**Comment**	ESIH by fangsu		

User Spectra

Fragmentor Voltage	Collision Energy	Ionization Mode
200	0	ESI

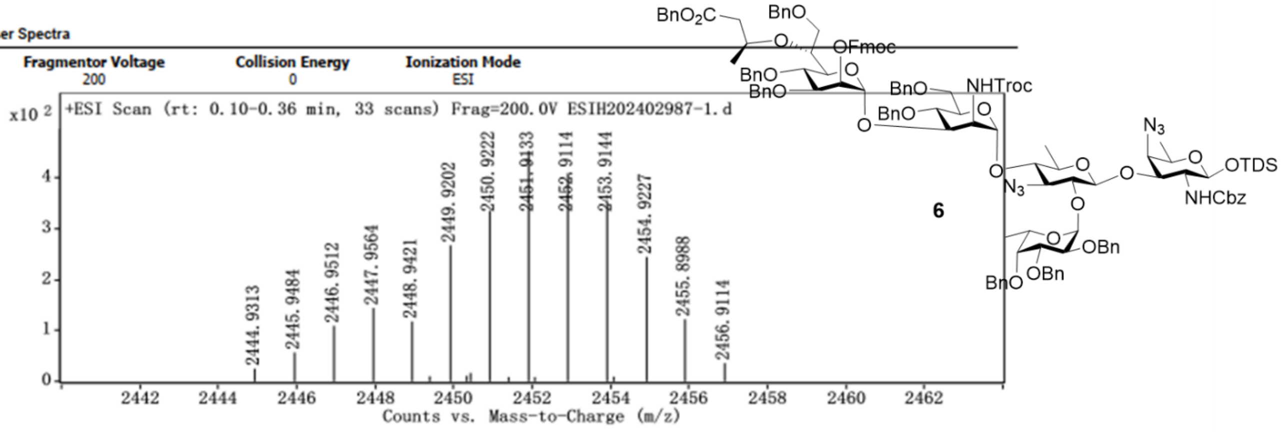

Formula Calculator Results

m/z	Calc m/z	Diff (mDa)	Diff (ppm)	Ion Formula	Ion
2449.9202	2449.9208	0.67	0.27	C132 H149 Cl3 N8 Na O28 Si	(M+Na)+

ESI HRMS of compound 6

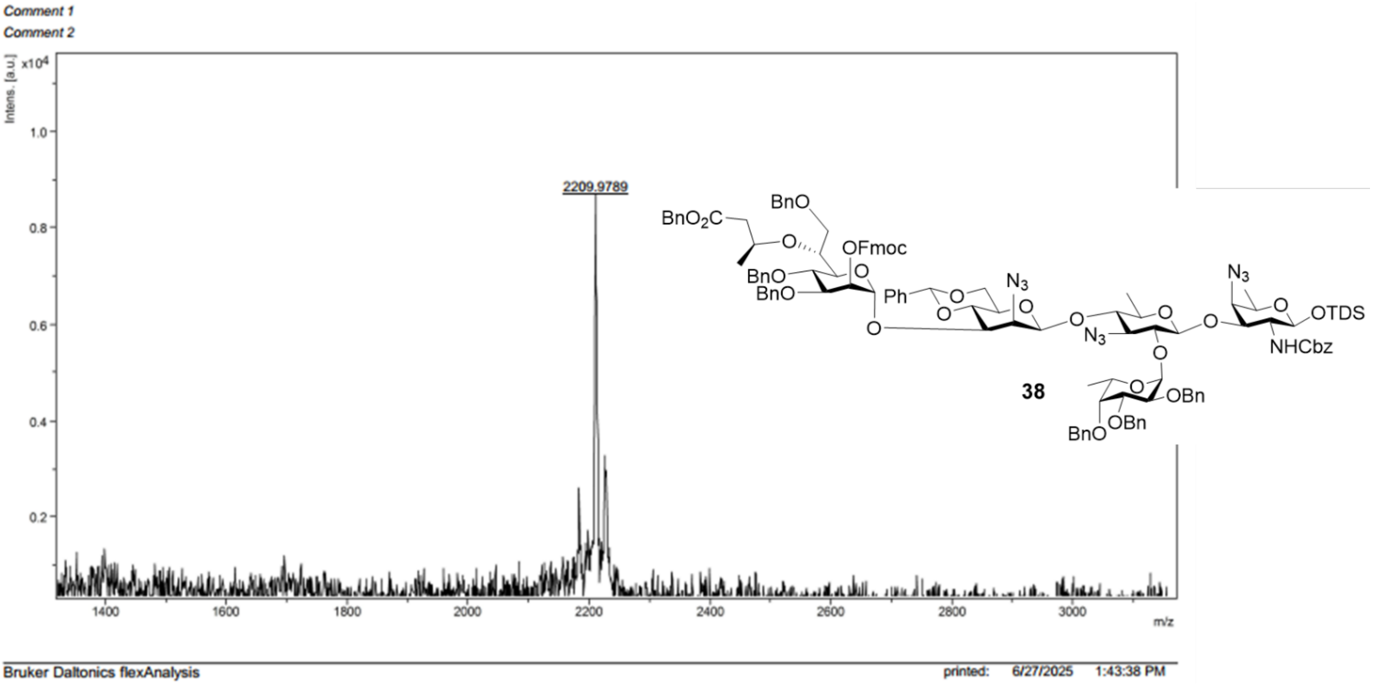

MALDI-TOF MS of compound 38

**Data Filename**	ESIH202500113.d	**Sample Name**	G0-G0-hth-5-101
**Sample ID**		**Position**	P1-A2
**Instrument Name**	Agilent 6520 Q-TOF	**Acq Method**	20160322_MS_ESIH_POS_1min.m
**Acquired Time**	1/9/2025 4:17:08 PM	**DA Method**	ESI-HR-20231114.m
**Comment**	ESIH by fangsu		

User Spectra

Fragmentor Voltage	Collision Energy	Ionization Mode
170	0	ESI

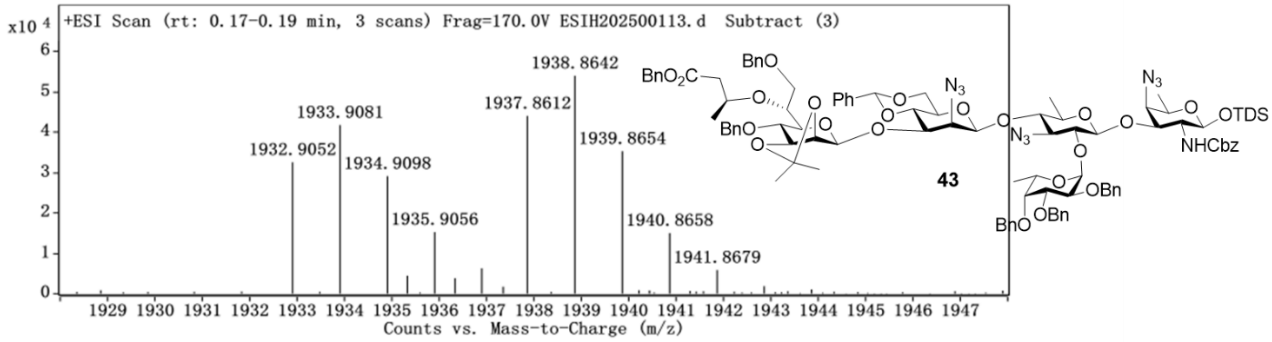

Formula Calculator Results

m/z	Calc m/z	Diff (mDa)	Diff (ppm)	Ion Formula	Ion
1937.8612	1937.8608	-0.45	-0.23	C103 H126 N10 Na O24 Si	(M+Na)+
1932.9052	1932.9054	0.23	0.12	C103 H130 N11 O24 Si	(M+NH4)+

ESI HRMS of compound 43

**Data Filename**	ESIH202403617.d	**Sample Name**	G0-G0-hth-4-0709-22
**Sample ID**		**Position**	P1-A4
**Instrument Name**	Agilent 6520 Q-TOF	**Acq Method**	20160322_MS_ESIH_POS_1min.m
**Acquired Time**	7/12/2024 3:04:06 PM	**DA Method**	ESI-HR-20231114.m
**Comment**	ESIH by fangsu		

User Spectra

Fragmentor Voltage	Collision Energy	Ionization Mode
170	0	ESI

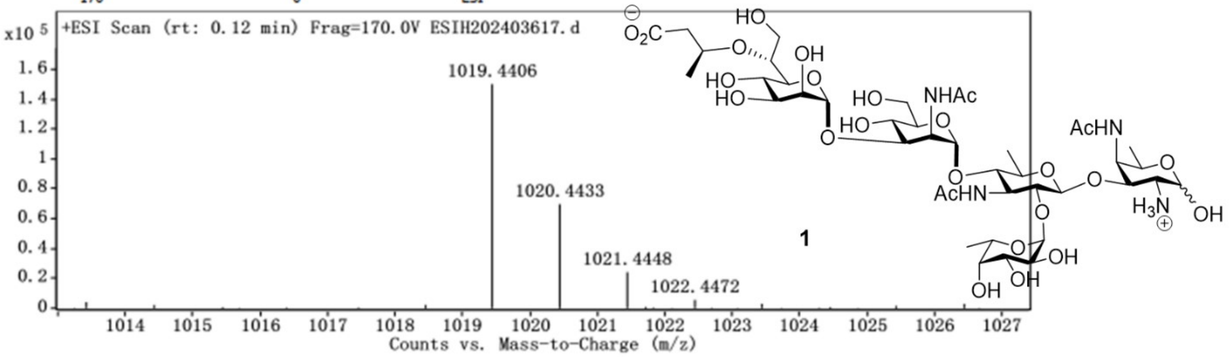

Formula Calculator Results

m/z	Calc m/z	Diff (mDa)	Diff (ppm)	Ion Formula	Ion
1019.4406	1019.4402	-0.39	-0.38	C41 H71 N4 O25	(M+H)+

--- End Of Report ---

ESI HRMS of compound 1

**Data Filename**	ESIH202503545.d	**Sample Name**	G0-G0-hth-5mer-ab
**Sample ID**		**Position**	P1-B1
**Instrument Name**	Agilent 6520 Q-TOF	**Acq Method**	20160322_MS_ESIH_POS_1min.m
**Acquired Time**	6/26/2025 2:17:02 PM	**DA Method**	ESI-HR-20231114.m
**Comment**	ESIH by fangsu		

User Spectra

Fragmentor Voltage	Collision Energy	Ionization Mode
170	0	ESI

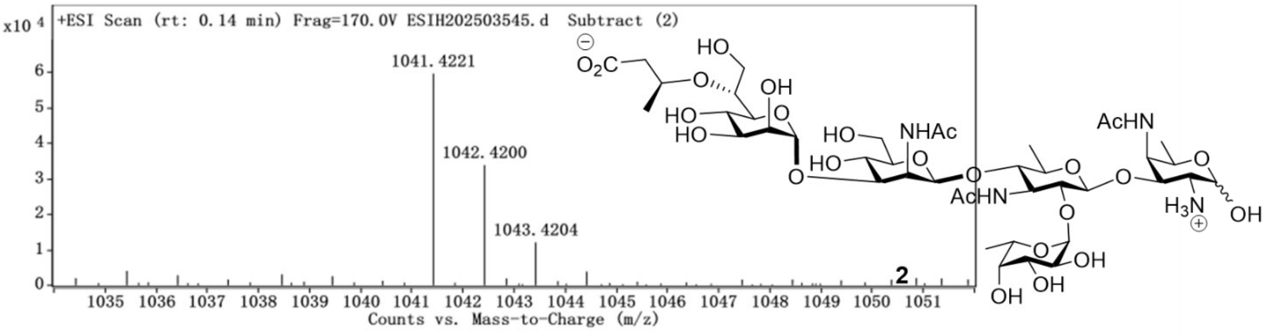

Formula Calculator Results

m/z	Calc m/z	Diff (mDa)	Diff (ppm)	Ion Formula	Ion
1041.4221	1041.4221	0.05	0.05	C41 H70 N4 Na O25	(M+Na)+

--- End Of Report ---

ESI HRMS of compound 2

**Data Filename**	ESIH202503546.d	**Sample Name**	G0-G0-hth-5mer-bb
**Sample ID**		**Position**	P1-B2
**Instrument Name**	Agilent 6520 Q-TOF	**Acq Method**	20160322_MS_ESIH_POS_1min.m
**Acquired Time**	6/26/2025 2:18:20 PM	**DA Method**	ESI-HR-20231114.m
**Comment**	ESIH by fangsu		

User Spectra

Fragmentor Voltage	Collision Energy	Ionization Mode
170	0	ESI

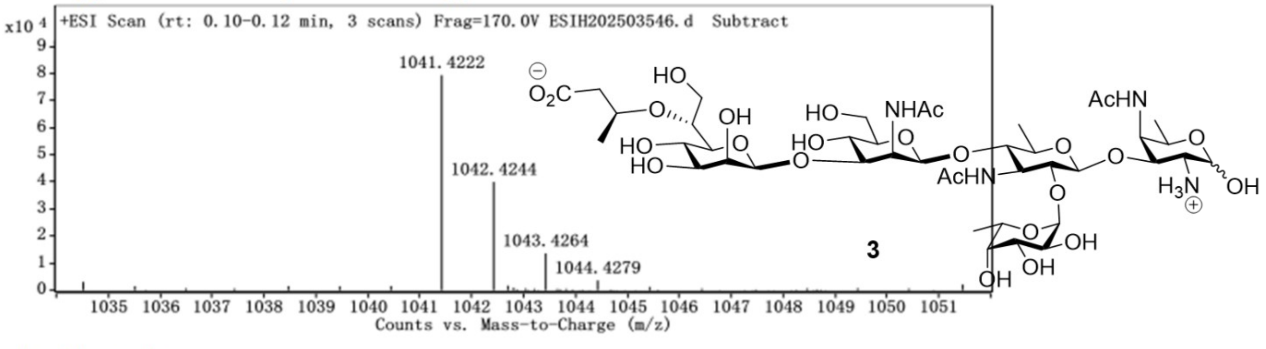

Formula Calculator Results

m/z	Calc m/z	Diff (mDa)	Diff (ppm)	Ion Formula	Ion
1041.4222	1041.4221	-0.11	-0.11	C41 H70 N4 Na O25	(M+Na)+

--- End Of Report ---

ESI HRMS of compound 3

**Data Filename**	ESIH202503572.d	**Sample Name**	G0-G0-hth-5-160
**Sample ID**		**Position**	P1-A7
**Instrument Name**	Agilent 6520 Q-TOF	**Acq Method**	20160322_MS_ESIH_POS_1min.m
**Acquired Time**	6/27/2025 4:04:13 PM	**DA Method**	ESI-HR-20231114.m
**Comment**	ESIH by fangsu		

User Spectra

Fragmentor Voltage	Collision Energy	Ionization Mode
170	0	ESI

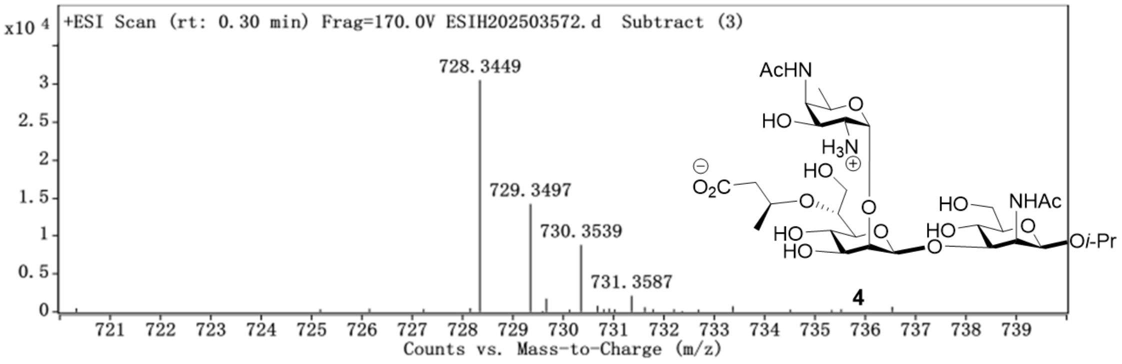

Formula Calculator Results

m/z	Calc m/z	Diff (mDa)	Diff (ppm)	Ion Formula	Ion
728.3449	728.3448	-0.08	-0.11	C30 H54 N3 O17	(M+H)+

ESI HRMS of compound 4

**Data Filename**	N_ESIH202501549.d	**Sample Name**	G0-G0-hth-5-152
**Sample ID**		**Position**	P1-B4
**Instrument Name**	Agilent 6520 Q-TOF	**Acq Method**	20160324_MS_ESIH_NEG_1min.m
**Acquired Time**	3/7/2025 2:16:35 PM	**DA Method**	ESI-HR-20231114.m
**Comment**	ESIH by fangsu		

User Spectra

Fragmentor Voltage	Collision Energy	Ionization Mode
170	0	ESI

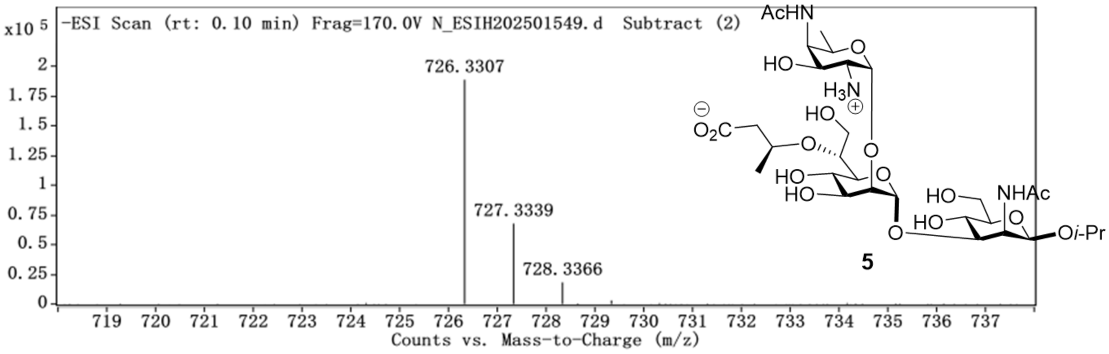

Formula Calculator Results

m/z	Calc m/z	Diff (mDa)	Diff (ppm)	Ion Formula	Ion
726.3307	726.3302	-0.44	-0.6	C30 H52 N3 O17	(M-H)-

ESI HRMS of compound 5
